# Pharmacogenomics of coronary artery response to intravenous gamma globulin in kawasaki disease

**DOI:** 10.1038/s41525-024-00419-7

**Published:** 2024-05-30

**Authors:** Sadeep Shrestha, Howard W. Wiener, Sabrina Chowdhury, Hidemi Kajimoto, Vinodh Srinivasasainagendra, Olga A. Mamaeva, Ujval N. Brahmbhatt, Dolena Ledee, Yung R. Lau, Luz A. Padilla, Jake Y. Chen, Nagib Dahdah, Hemant K. Tiwari, Michael A. Portman

**Affiliations:** 1 Department of Epidemiology, School of Public Health, University of Alabama at Birmingham, Birmingham, AL USA; 2 Division of Cardiology, Seattle Children’s and University of Washington Department of Pediatrics, Seattle, WA USA; 3 Department of Biostatistics, School of Public Health, University of Alabama at Birmingham, Birmingham, AL USA; 4 Division of Pediatric Cardiology, Heersink School of Medicine, University of Alabama at Birmingham, Birmingham, AL USA; 5 Department of Biomedical Informatics and Data Science, Heersink School of Medicine, University of Alabama at Birmingham, Birmingham, AL USA; 6 Division of Pediatric Cardiology, CHU Ste-Justine, Universite de Montreal, Montreal, QC Canada

**Keywords:** Risk factors, DNA sequencing, Sequence annotation

## Abstract

Kawasaki disease (KD) is a multisystem inflammatory illness of infants and young children that can result in acute vasculitis. The mechanism of coronary artery aneurysms (CAA) in KD despite intravenous gamma globulin (IVIG) treatment is not known. We performed a Whole Genome Sequencing (WGS) association analysis in a racially diverse cohort of KD patients treated with IVIG, both using AHA guidelines. We defined coronary aneurysm (CAA) (*N* = 234) as coronary z ≥ 2.5 and large coronary aneurysm (CAA/L) (N = 92) as z ≥ 5.0. We conducted logistic regression models to examine the association of genetic variants with CAA/L during acute KD and with persistence >6 weeks using an additive model between cases and 238 controls with no CAA. We adjusted for age, gender and three principal components of genetic ancestry. The top significant variants associated with CAA/L were in the intergenic regions (rs62154092 *p* < 6.32E–08 most significant). Variants in *SMAT4, LOC100127*, *PTPRD, TCAF2* and *KLRC2* were the most significant non-intergenic SNPs. Functional mapping and annotation (FUMA) analysis identified 12 genomic risk loci with eQTL or chromatin interactions mapped to 48 genes. Of these *NDUFA5* has been implicated in KD CAA and *MICU* and *ZMAT4* has potential functional implications. Genetic risk score using these 12 genomic risk loci yielded an area under the receiver operating characteristic curve (AUC) of 0.86. This pharmacogenomics study provides insights into the pathogenesis of CAA/L in IVIG-treated KD and shows that genomics can help define the cause of CAA/L to guide management and improve risk stratification of KD patients.

## Introduction

Kawasaki disease (KD) is a life-threatening acute vasculitis that diffusely affects multiple organ systems in children. Coronary artery dilatations and aneurysms can occur and represent the most serious KD complications^1,2^. For most patients, KD is self-limited and lacks the chronic nature of other autoimmune diseases; however, the pathological walls of afflicted vessels show a propensity for forming thrombosis and aneurysms^3^. If untreated or treatment fails, the vasculitis can lead to coronary aneurysm or thrombosis in 20–25% of cases, potentially resulting in ischemic heart disease, myocardial infarction, or death^4,5^. Clinical trials conducted in the 1980s and 1990s showed that IVIG treatment dramatically reduced occurrence of persistent CAA defined, primarily by Japanese Ministry of Health criteria^6,7^. These criteria stated that coronary artery diameters ≥3 mm in children <5 years and ≥4 mm in children ≥5 years were classified as abnormal. Echocardiographic detection and definition of significant CAA has dramatically improved over the past 2 to 3 decades. A 2007 study by the National Heart Lung and Blood Institute (NHLBI) showed that approximately 18 to 20% of patients had persistent CAA determined using coronary artery z scores^8,9^. The 2017 AHA guidelines adjusted the definition for CAA as z score ≥ 2.5 and ≥5 defines a medium to large CAA^1^. Although these coronary abnormalities show higher prevalence in IVIG refractory patients, they can still occur in patients seemingly responsive and showing fever resolution. Most of these children with large aneurysms require daily lifelong anti-coagulation, often with twice daily painful low molecular weight heparin injections, as warfarin is difficult to maintain within therapeutic range in children.

A study based on a large-scale Japanese cohort reported that coronary events did not occur in patients with small CAA; however, 5% of patients with medium CAA and 35% of patients with large CAA had coronary events^10^. North American studies support these data from Japan^11^. The most severe form, giant coronary artery aneurysm (GCA), has been shown to be associated with complications such as luminal narrowing, thrombosis, and major cardiac events^11,12^, and substantially alters quality of life for KD patients. Children with GCA require lifelong anti-coagulation, exercise restrictions, and often treatment for ischemic heart disease such as coronary stenting and/or bypass grafting^1^. The majority of patients with GCA develop clinically important stenosis from intimal hypertrophy during the late convalescent phase^13^. Japanese males with coronary artery involvement have a mortality rate 2.4 times higher than expected in general population^14^, but the overall impact on KD patients in the U.S. still requires definition. A recent systematic review showed that mid- to large-sized CAA provided the most significant risk factor for reducing survival of patients with KD^15^. Mid- to large-sized CAA showed a slower recovery with worse prognosis than smaller CAA^16^. Another study reported that the high persistence probability of mid- to large-sized CAA significantly increased the cardiovascular risk at 1 year after KD onset, when approximately two-thirds of the acute myocardial infarction cases occur^17^.

Predicting increased risk for persistent large (medium to giant) size coronary aneurysms despite IVIG treatment is clinically important for intensification of treatment and disease management. Algorithms combining clinical and lab data in Japanese populations predict risk with respect to persistent CAA^18^. However, the Japanese algorithms show poor predictive value for risk in North American and European cohorts^8,19^, and fail even in some Asian populations^20,21^. Thus, no universal biomarker or algorithm accurately predicts risk for persistent CAA in North America^22^.

A single U.S center retrospectives study showed that presence of early coronary artery dilation is moderately useful in predicting persistent dilation. However, that study did not specifically evaluate for larger higher risk aneurysms^23^. Currently available data indicate that KD susceptibility and treatment response depend on an individual patient’s genetic background^24–33^. Discrepancies among races or ethnicities also suggest that pathogenesis of KD might vary^34,35^. To date, very few studies have focused on genetic risk factors for CAA development in KD patients. We performed the first Whole Genome Sequencing (WGS) association analysis in a cohort of KD patients in a racially diverse North American population exhibiting differences in artery aneurysm formation. We identified multiple loci associated with CAA formation among a pediatric KD population receiving IVIG that can inform on risk stratification; potentially serve as treatment response predictors and guidance toward new therapeutic targets.

## Results

### Genetic association analysis between individual SNPs and the risk of large (medium/giant) aneurysm

**Fig. 1:**
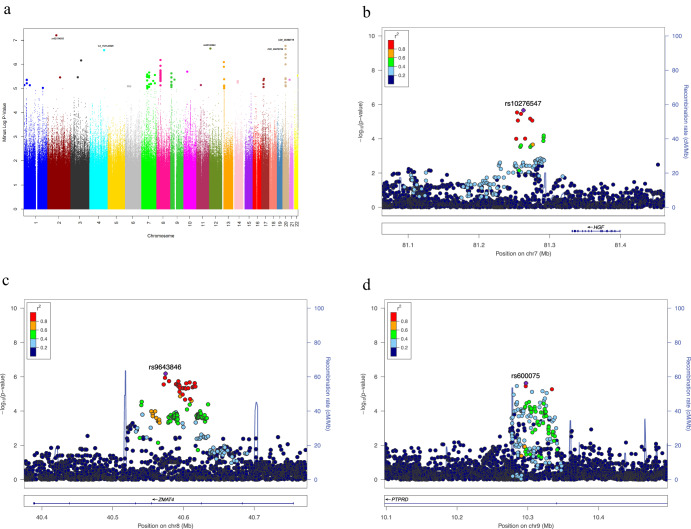
Overall and regional association results. **a** Manhattan Plot displaying Whole Genome Sequence Association results with—Large (medium/giant) Coronary Aneurysm (*N* = 92) vs no Coronary Artery Aneurysm (*N* = 276). Negative log_10_-transformed *P* values from the logistic regression model (additive model) are plotted on the *y-*axis and the SNP genomic locations on the x-axis (colors representing different autosomal chromosomes). Locus Zoom plots for selected gene regions in (**b**) Chromosome 7 with rs10276547 the most significant SNP in the region, (**c**) chromosome 8 with rs9643846 the most significant SNP in the region, (**d**) Chromosome 9 with rs600075 the most significant SNP in the region. Vertical axis (on the left) is the –log_10_ of the p-value, the horizontal axis is the chromosomal position. Each dot represents a SNP tested for association with large coronary aneurysm. Linkage disequilibrium between the most significant SNP, listed at the top of each plot, and the other SNPs in the plot is shown by the r^2^ legend in each plot. Vertical axis (on the right) is the recombination—the site and rate are represented by red curves.

**Fig. 2:**
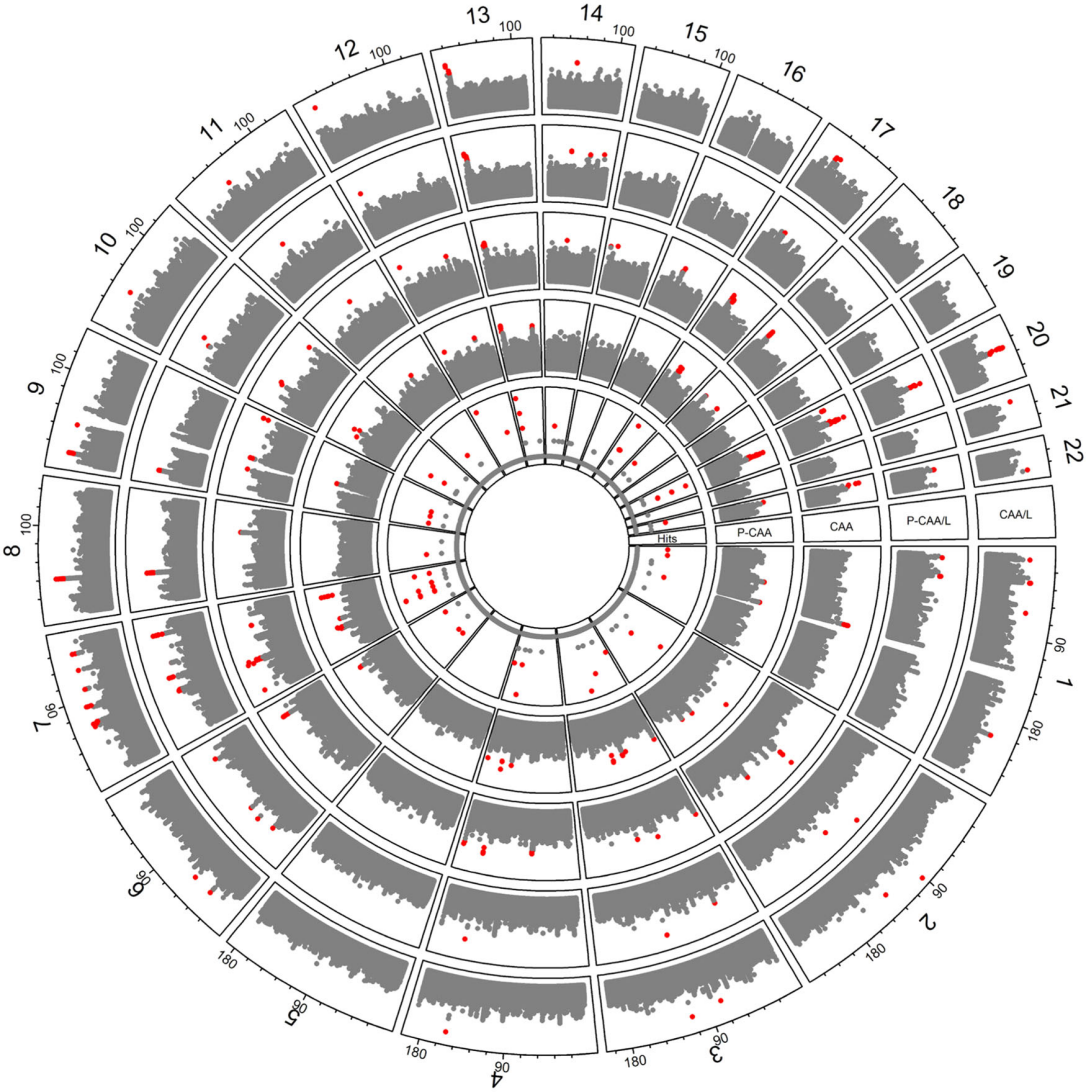
Circo plot summarizing whole genome sequence associations. The outer plot is the association of large aneurysm (*N* = 92), the next inner plot is the association of persistent large aneurysm (*N* = 79), the next inner plot is the association of any coronary aneurysm (*N* = 233) and the fourth inner plot is the association of persistent any coronary aneurysm vs no coronary aneurysm (for each outcome). The innermost plot indicates if SNPs were associated in 1–4 outcomes with *p* < 1.0E–05.

To identify SNPs associated with KD-associated large (medium/giant) coronary aneurysm (CCA/L), clinical data were linked in KD patients with whole genome sequencing data, nested in a clinical cohort as previously described^36^. Basic demographics of the study population with CCA/L (*N* = 91) and no aneurysm (*N* = 278) are described in Table 1. Principal component analysis confirmed a good match between KD patients with CCA/L and those without any aneurysm. However, 3 PC were adjusted for in the analyses, as estimated in previous study^36^. A quantile-quantile plot indicated that population stratification had negligible effects on the statistical results (λ genomic control = 0.955). There were several SNPs that exhibited suggestive statistical significance (*p* < 10^–5^) in the additive genetic model, as shown in the Manhattan plot (Fig. 1). Of all the SNPs examined, rs62154092 in the intragenic region (nearest gene *ACTR3BP2*) was the most statistically significant (6.32E–08). Among the overall top 10 most significant SNPs, 5 SNPs (all intergenic rs1424006606, rs1396081550, rs1258107032, rs1379390981, rs1424309393) were in chromosome 20, although in different regions. All SNPs statistically significant at *p* < 10^–4^ are listed in Supplementary Table S1. Among the non-intergenic SNPs, rs28730284 upstream of *KLRC2* gene was the most statistically significant (2.20 E-07) and among the top 15 non-intergenic, they were mostly intronic (rs9643846, rs9643847, rs57504215, rs60545202, rs59556769, rs73677451, rs12676292, rs4332118, rs6988966) located in *SMAT4* and others in *LOC100127* (non-coding RNA rs10276547, rs10280266), *PTPRD* (intronic rs600075, rs5896385) and *TCAF2* (intronic rs1218424730) genes. The most significant exonic SNPs (rs11259953 and rs11259954) were in *WHAMM* gene (Supplementary Table S1). Regional association plots with cluster of SNPs in LD in chromosomes 7, 8 and 9 are shown in (Fig. 1b–d). Results of corresponding single SNP association with developing any CAA (*N* = 233), any persistent CAA (*N* = 145) for 2 years, and P-CCA/L for 2 years (*N* = 79) vs no aneurysm (*N* = 276) are shown in Fig. 2 and Supplementary Table S1.

**Table 1:** Kawasaki Disease Patients With (medium/large, any) and Without Coronary Aneurysm included in the whole genome sequencing analyses

	Medium/Giant Coronary Aneurysm Z ≥ 5	Persistent Medium/Giant Coronary Aneurysm	Any Coronary Aneurysm Z ≥ 2.5	Persistent Coronary Aneurysm	No Coronary Aneurysm Z < 2.5
N	91	79	233	145	278
Median Age (months)	20	21.5	27	26	36
Gender Male Female	58 34	48 31	154 79	95 50	157 119

### Gene mapping

**Fig. 3:**
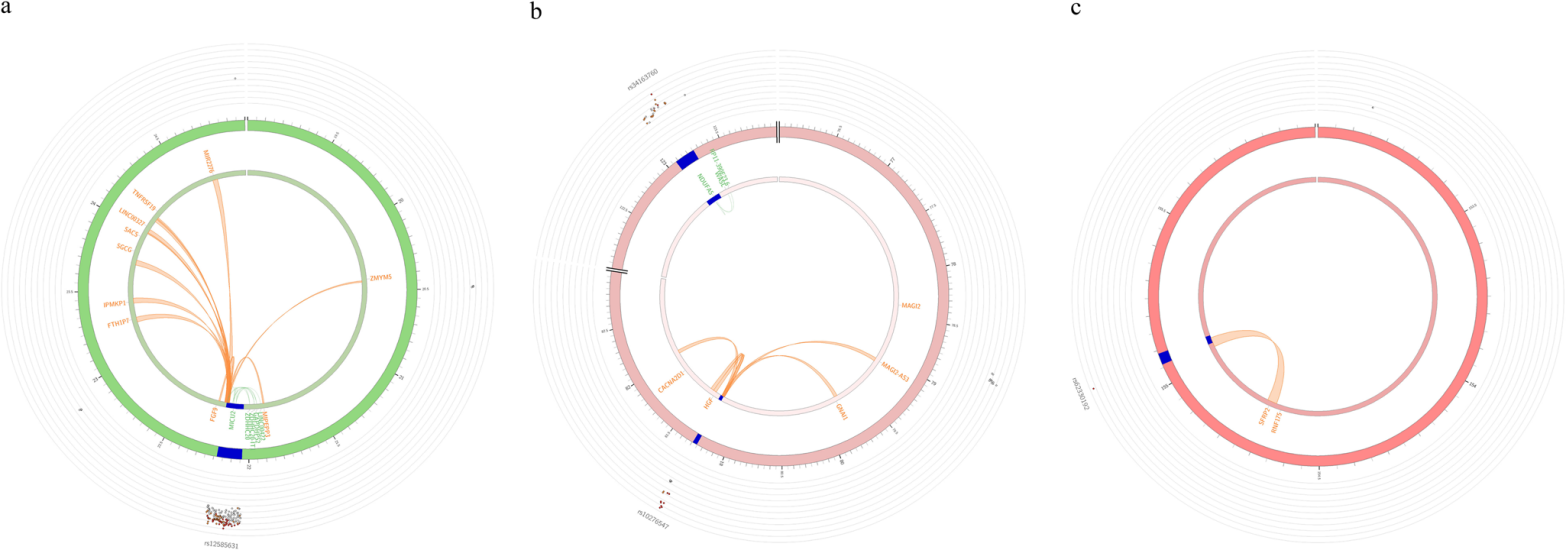
FUMA circos plots of mapped genes in genomic risk loci. The most outer layer is the Manhattan plot (only SNPs with *P* < 0.05 are displayed). Genomic risk loci are highlighted in blue and the strength of linkage disequilibrium r^2^ between each SNP to the lead SNP is given by the following color code: red (r^2^ > 0.8), orange (r^2^ > 0.6), green (r^2^ > 0.4), blue (r^2^ > 0.2) and gray (r^2^ ≤ 0.2). Genes are mapped by 3-D chromatin interaction (orange) or eQTLs (green), or both (red). **a** Circos plot for Chromosome 13 with lead SNP rs12585631, (**b**) Circos plot for Chromosome 7 with lead SNP rs10276547, and (**c**) Circos plot for Chromosome 4 with lead SNP rs62330192.

Using three gene mapping strategies (position mapping, eQTL mapping and chromatin interaction mapping) in FUMA, we mapped the significant association variants (*P* < 10^–5^) to genes and identified 12 genomic risk loci (Supplementary Table S2) and 48 mapped genes associated with CCA/L (Fig. 3, Supplementary Table S3). None of the genes were mapped by all three strategies. Three genes *NDUFA5*, *ZMAT4* and *MICU2* were mapped by physical and eQTL - *NDUFA5* is located at the chromosome 7, and its lead SNP rs34163760 is located in the intron of the gene (*P* = 4.84E-06). An eQTL analysis showed that with the increasing number of risk alleles of rs34163760, there was a higher mRNA level of *NDUFA5* in the Esophagus. The CADD score of rs34163760 is 14.39 indicating a deleterious mutation. *ZMAT4* is located in chromosome 8, and its lead SNP rs9643846 is located in the intron of the gene (*P* = 6.57E-07). An eQTL analysis showed that with the decreasing number of risk alleles of rs9643846, there was a higher mRNA level of *ZMAT4* in thyroid. CADD score of rs9643846 is 15 indicating a deleterious mutation. The third gene *MICU2* is located in chromosome 13, and its lead SNP rs12585631 is located in the intron of the gene (*P* = 4.11E-06). An eQTL analysis showed that with the decreasing number of risk alleles of rs12585631, there was a higher mRNA level of *MICU2* in thyroid. The CADD score of rs12585631 is 15.22 indicating a deleterious mutation. Several genes in chromosomes 4, 7 and 13 were also identified to interact with the chromatin at those sites (Fig. 3, Supplementary Table S3).

Expression patterns of the 48 prioritized genes were estimated in 54 different tissues (Supplementary Table S4 Supplementary Fig. S1). Several of these genes show high expression in aorta and coronary artery tissues.

### Genetic risk score (GRS)

**Fig. 4:**
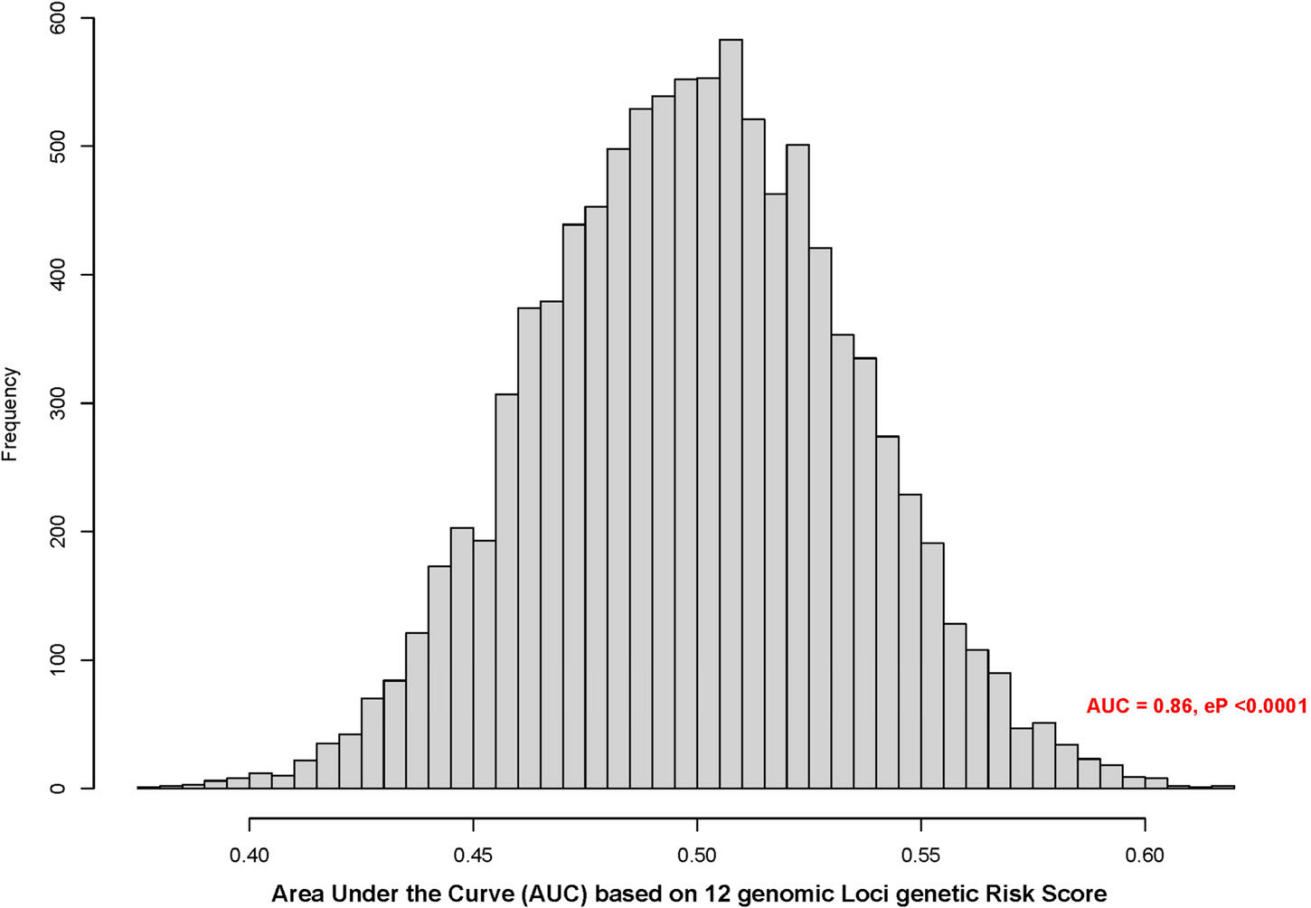
Empirical curve based on the area under the curve (AUC) from 10,000 permutation. X-axis is the AUC value after a random permutation of the outcome variable (medium to large coronary aneurysm, z > 5.0) based on the 12 genomic loci from FUMA in predicting the risk score of having a large aneurysm and Y-axis is the frequency. Empirical P-value (eP) is proportion of permutations resulting in a larger AUC than original data.

Twelve genomic risk loci identified from FUMA yielded an AUC of 0.86. As shown in Fig. 4, based on the empirical distribution of the AUC from the permutation test, eP was <0.0001, suggesting highly significant genetic risk score from the 12 genomic risk loci. For sensitivity analyses, when GRS for CCA/L was conducted separately in four specific races, AUC of 0.83, 0.78, 0.81 and 0.97 were obtained among White, Asian, Hispanic and African American KD patients.

## Discussion

We analyzed a relatively large North American KD cohort using whole genome sequencing. Treatment with IVIG during the acute phase was an inclusion criterion, so lack of treatment was not a confounding factor. Additionally, persistent coronary artery aneurysm (P-CAA or P-CCA/L) should be considered a failure of IVIG therapy. As noted in Table 1, the vast majority of medium to giant coronary aneurysms persisted. We identified for the most part novel gene loci that appear to have a relationship with coronary artery aneurysm formation and persistence in KD patients. We have used this same WGS strategy to identify genes related to IVIG refractoriness as defined by AHA guidelines^1^. Prior studies searching for CAA genetic risk variants have used either hypothesis driven strategies or genome-wide association strategy with their inherent limitations. In this study, we specifically used the newest AHA classifications, and sought to determine genetic associations with CCA/L (Z ≥ 5) as these show a lower chance for early regression than do smaller aneurysms (Z ≥ 2.5, but < 5). However, we also found that statistical genetic associations were consistent among all coronary phenotype groups as shown in Table 2 and Fig. 2. This suggests that pathobiology or at least genetic risk is consistent regardless of size of the aneurysm or propensity for regression.

**Table 2:** Top genes associated with large (medium/giant) coronary aneurysm (CCA/L) (z ≥ 5), persistent CCA/L (P-CCA/L), any coronary artery aneurysm (CAA) (z ≥ 2.5), and persistent CAA (P-CAA)

Overall Top 10 Hits									
	Genes and gene regions	Large Aneurysm		Persistent Large Aneurysm		Any Aneurysm		Persistent Any Aneurysm	
SNP*	Closest gene	OR (95% CI)	*P* value	OR (95% CI)	*P* value	OR (95% CI)	*P* value	OR (95% CI)	*P* value
**rs62154092 (T/c)**	*ACTR3BP2;NONE*	0.17 [0.09, 0.33]	6.32E–08	0.16, [0.08, 0.30]	3.22E–08	0.23, [0.14, 0.39]	3.25E–08	0.20, [0.12, 0.36]	3.28E–08
**rs1424006606 (C/t)**	*FRG1DP;FRG2EP*	0.15 [0.08, 0.31]	1.71E–07	0.19, [0.09, 0.38]	3.37E–06	0.40, [0.26, 0.59]	6.78E–06	0.29, [0.18, 0.48]	1.53E–06
**rs28730284 (G/a)**	*KLRC2*	0.18 [0.10, 0.35]	2.20E–07	0.20, [0.10, 0.39]	1.96E–06	0.37, [0.25, 0.55]	6.34E–07	0.35, [0.22, 0.55]	6.86E–06
**rs1396081550 (C/t)**	*FAM242B;MIR4477A*	0.24 [0.14, 0.41]	2.28E–07	0.22, [0.13, 0.39]	1.97E–07	0.35, [0.24, 0.52]	3.01E–07	0.35, [0.22, 0.54]	4.14E–06
**rs62330192 (A/g)**	*SFRP2;DCHS2*	0.19 [0.10, 0.36]	2.59E–07	0.20, [0.10, 0.38]	1.09E–06	0.38, [0.25, 0.56]	1.14E–06	0.31, [0.19, 0.49]	7.82E–07
**rs1258107032 (T/a)**	*FRG1CP;FRG1DP*	5.76 [2.93, 11.32]	3.83E–07	6.11, [2.96, 12.63]	1.04E–06	2.70, [1.82, 4.00]	7.60E–07	3.44, [2.13, 5.57]	4.80E–07
**rs1379390981 (T/g)**	*FAM242B;MIR4477A*	0.21 [0.11, 0.38]	3.86E–07	0.20, [0.11, 0.38]	8.74E–07	0.27, [0.17, 0.44]	6.23E–08	0.24, [0.14, 0.41]	1.93E–07
**rs1424309393 (A/t)**	*FRG1CP;FRG1DP*	5.64 [2.87, 11.08]	5.37E–07	5.97, [2.88, 12.34]	1.45E–06	2.74, [1.84, 4.07]	6.02E–07	3.41, [2.11, 5.53]	5.86E–07
**rs9643846 (G/t)**	*ZMAT4*	4.12 [2.36, 7.20]	6.57E–07	4.16, [2.35, 7.39]	1.10E–06	2.30, [1.54, 3.44]	5.33E–05	2.04, [1.30, 3.20]	1.97E–03
**rs377109159 (T/c)**	*MIR4445;NECTIN3-AS1*	4.60 [2.52, 8.40]	6.86E–07	5.24, [2.80, 9.80]	2.23E–07	2.83, [1.79, 4.47]	8.01E–06	3.49, [2.11, 5.79]	1.25E–06

Top 5 exonic SNPs									
SNP	Gene	OR (95% CI)	*P* value	OR (95% CI)	*P* value	OR (95% CI)	*P* value	OR (95% CI)	*P* value
**rs11259953 (A/c)**	*WHAMM*	3.30(1.90, 5.74)	2.25E–05	3.34(1.88, 5.93)	3.74E–05	1.98(1.29, 3.02)	1.65E–03	2.01(1.24, 3.24)	4.33E–03
**rs11259954 (C/g)**	*WHAMM*	3.30(1.9, 5.74)	2.25E–05	3.34(1.88, 5.93)	3.74E–05	1.98(1.29, 3.02)	1.65E–03	2.01(1.24, 3.24)	4.33E–03
**rs1055510 (A/g)**	*MADD-AS1*	0.42(0.27, 0.65)	8.76E–05	0.36(0.23, 0.58)	2.16E–05	0.59(0.43, 0.79)	4.24E–04	0.51(0.36, 0.72)	1.13E–04
**rs1052373 (C/t)**	*MYBPC3*	0.44(0.29, 0.68)	1.85E–04	0.39(0.25, 0.61)	5.28E–05	0.60(0.45, 0.81)	7.69E–04	0.53(0.37, 0.74)	2.18E–04
**rs77868901 (G/c)**	*TMEM259*	9.00(2.76, 29.31)	2.66E–04	8.57(2.62, 28.05)	3.86E–04	3.60(1.21, 10.71)	2.13E–02	4.69(1.51, 14.54)	7.46E–03

Top 10 non-exonic SNPs in the gene									
SNP	Gene	OR (95% CI)	*P* value	OR (95% CI)	*P* value	OR (95% CI)	*P* value	OR (95% CI)	*P* value
**rs28730284 (A/g)**	*Upstream KLRC2*	0.18(0.10, 0.35)	2.20E–07	0.20(0.1, 0.39)	1.96E–06	0.37(0.25, 0.55)	6.34E–07	0.35(0.22, 0.55)	6.86E–06
**rs9643846 (T/g)**	*Intronic ZMAT4*	4.12(2.36, 7.2)	6.57E–07	4.16(2.35, 7.39)	1.10E–06	2.3(1.54, 3.44)	5.33E–05	2.04(1.3, 3.2)	1.97E–03
**rs9643847 (G/a)**	*Intronic ZMAT4*	3.95(2.27, 6.86)	1.14E–06	3.86(2.19, 6.82)	3.19E–06	2.14(1.44, 3.2)	1.89E–04	1.99(1.27, 3.12)	2.62E–03
**rs57504215 (G/a)**	*Intronic ZMAT4*	3.88(2.23, 6.78)	1.78E–06	3.90(2.2, 6.92)	3.04E–06	2.18(1.45, 3.27)	1.61E–04	1.9(1.21, 2.98)	5.60E–03
**rs60545202 (A/g)**	*Intronic ZMAT4*	3.91(2.23, 6.84)	1.82E–06	3.96(2.22, 7.06)	2.97E–06	2.13(1.41, 3.23)	3.47E–04	1.98(1.26, 3.14)	3.37E–03
**rs59556769 (G/a)**	*Intronic ZMAT4*	3.73(2.16, 6.42)	2.11E–06	3.87(2.21, 6.77)	2.18E–06	2.01(1.35, 2.99)	6.39E–04	1.98(1.27, 3.08)	2.67E–03
**rs10276547 (C/t)**	*ncRNA intronic LOC100128317*	5.55(2.73, 11.27)	2.17E–06	5.93(2.88, 12.2)	1.34E–06	2.74(1.56, 4.81)	4.31E–04	3.88(2.09, 7.22)	1.78E–05
**rs73677451 (G/t)**	*Intronic ZMAT4*	3.70(2.15, 6.38)	2.36E–06	3.72(2.13, 6.52)	4.14E–06	2.08(1.39, 3.09)	3.34E–04	1.98(1.27, 3.09)	2.50E–03
**rs600075 (G/a)**	*Intronic PTPRD*	3.59(2.11, 6.09)	2.36E–06	3.55(2.05, 6.16)	6.45E–06	2.30(1.57, 3.37)	1.79E–05	2.41(1.56, 3.74)	8.24E–05
**rs5896385 (-/a)**	*Intronic PTPRD*	3.59(2.11, 6.09)	2.36E–06	3.55(2.05, 6.16)	6.45E–06	2.30(1.57, 3.37)	1.79E–05	2.41(1.56, 3.74)	8.24E–05

*First allele is the major allele and the second is the minor allele. Associations statistics (OR and *p* values) are based on the minor allele.

The most significant SNP related to moderate to giant aneurysm was located in the intergenic region with the closest gene being *ACTR3BP2*, a pseudogene with unknown function. However, among the top SNPs in the gene region, rs28730284 was located just upstream of the *KLRC2* gene and has intriguing potential biological relevance to KD. The *KLRC2* gene encodes the C-type lectin NKG2C (Killer cell lectin receptor-2). Natural Killer (NK) cells mediate innate immune responses against virally infected and malignant cells^37–39^. NK cell function such as production of proinflammatory cytokines, depends on a balance between activating and inhibiting signals triggered by multiple surface receptors, including NKG2C^40^. Polymorphisms in *KLCR2* have been shown to influence both function and expression of NK cells. Furthermore, SNPs in *KLRC2* are associated with microvascular inflammation during renal graft transplant rejection. Importantly, all type NK cell (CD56^++^CD16^+−^, CD56^+^CD16^+^, CD56^−^CD16^+^) expression is reduced in KD patients compared to febrile or non-febrile controls, while CD56 − CD16 + NK cell expression was significantly lower in IVIG-resistant patients than in the IVIG-responsive^41^.

Multiple intronic SNPs were found in *ZMAT4* gene, which encodes the Zinc Finger Matrin-Type 4 protein. This gene was also identified by FUMA. SNPs within ZMAT4 are associated with diseases such as Spinocerebellar Ataxia and Myopia^42^, and copy number variations are associated with hematological malignancies^43^. Function of this particular Zinc-Finger protein remains undefined, so a potential biological role in KD would be unclear. The top two exonic SNPs were in the *WHAMM* gene. This gene encodes a protein nucleation-promoting factor that regulates the Actin-related protein 2/3 complex, but any biological relevance to KD would be highly speculative. Additionally, we found SNP (rs1052373) within the *MYBPC3* exon region as marginally significant. MYBPC3 (myosin binding protein c3) function is well established and mutations are involved in the pathology of hypertrophic cardiomyopathy^44,45^. This particular SNP has also been associated with athletic endurance^46^. However, any suggestion of biological relevance for these exonic SNPs to KD would be highly speculative.

Multiple SNPs were also found in regions near Facioscapulohumeral muscular dystrophy (FSHD) region-1(*FRG1DP*). FRG1 acts on upstream of FGF2, which signals activation of the AKT/ERK signaling axis in endothelial cells. Interestingly, we previously reported that this gene is associated with IVIG response in KD patients. *FRG1DP* has been linked to angiogenesis and retinal vasculpathy in FSHD patients including development of micro aneurysms. Additionally, altered expression for FRG-1 protein leads altered angiogenesis in human umbilical vein endothelial cells (HUVECs).

Using FUMA we found several genes potentially related to CAA/L in IVIG-treated KD patients. *MICU2* is a calcium sensitive regulatory subunit of the mitochondrial calcium uniporter and is important for reducing oxidative stress particularly in endothelial cells. MICU 2 −/− mice exhibit abnormal cardiac diastolic relaxation but also develop abdominal aortic aneurysms, which spontaneously rupture with only modest increases in blood pressure. *NDUFA5* encodes NADH dehydrogenase [ubiquinone] 1 alpha subcomplex subunit 5, a critical component of mitochondrial respiratory complex 1, which facilitates the translocation of protons across to the mitochondrial inner membrane. A SNP in *NDUFA5* was also a top hit in a small Taiwanese KD study evaluating genetic risk for CAA formation^47^. Thus, our findings for these two genes regulating oxidative stress response suggest that mitochondria play a role in development of CAA and should be a target for future research^48^.

We previously published a WGS pharmacogenomics analyses of IVIG response in KD using “persistent or recurrent fever” as the benchmark for IVIG resistance^36^. Data suggests that IVIG resistance or refractoriness is a risk factor for persistent CAA. However, we did not find numbers of variants that were associated with both coronary aneurysms and IVIG resistance. This lack of genetic association uniformity between these two different outcomes suggests that their biological pathways and mechanisms may be different. However, the list of novel genes provides new insights into the pathogenesis of KD.

Genetic analyses of coronary aneurysms in KD are complicated by multiple potential confounding factors. Prior studies, which have identified numerous significant variants associated with coronary aneurysms, did not account for the IVIG therapeutic effect^49–51^. Clinical trials clearly show that IVIG reduces the risk of CAA^52^. However, those previous genetic studies for the most part do not clarify whether study participants received appropriate IVIG treatment. Thus those prior cohorts could and probably do include patients whom did not receive timely IVIG. We used strict criteria for IVIG treatment in our study subjects in accordance with pharmacogenomics design. Accordingly, our results using different design and methodology did not replicate findings from prior studies such as associations with coronary aneurysms for variants in *ITPKC*^51^, *KCNN2*^50^, *NEBL* and *TUBA3C*^47^, *SLC8A1*^53^, and the matrix metalloproteinase (*MMP*) gene family^54^. Likewise, Huang et al^55^ reported TET mRNA levels associated with IVIG and DMNT1 mRNA levels with CAA; however, there were no SNPs in these gene regions that were statistically significant in our study.

Two other studies indicated association of TIFAB^56^ and PLCB1^57^ genes. Although the same SNPs were not replicated in our study, we found several SNPs in these gene regions that were associated with CAA in our study (Table S1). Unlike studies predominantly using Asian populations, our cohort included 4 races (Whites, Asians, African-Americans, and Hispanics). We had fewer cases of African-Americans and Hispanics and we were underpowered to conduct race-specific analyses (Supplementary Table S5). However, in the main combined cohort, we adjusted for three principal components which resulted in λ genomic control = 0.955, suggesting no major confounding factors (Supplementary Figure S2).

We also used 12 genomic loci from FUMA to test for overall prediction of risk for developing CAA/L, a genetic predictive risk score. The AUC based on these markers is promising and the empirical risk models based on 10,000 simulated cases and controls had considerably higher AUC than theoretically achievable. Although sample size is small, all race-specific analyses also showed similar trends with high AUC. These specific markers need to be validated in different IVIG-treated KD populations; however, there is potential clinical utility in developing a point-of-care assay based on panels of genetic risk markers to predict who will develop CAA/L and/or other sequalae. Accurate prediction can assist in developing a treatment plan during the acute KD phase^58^.

The main limitation for this study is the lack of a validation cohort. This is a common limitation of clinical trials and pharmacogenomics studies of rare diseases, although our cohort is the largest reported to date with a clear IVIG treatment phenotype. Further validations as well as functional studies of these variants will be needed in the future.

In summary, using WGS we have identified several novel genes and loci, which could have a functional impact on coronary artery response to IVIG in KD. Additionally, these loci could be used in identifying new personalized therapeutic avenues as well as developing an important predictive risk score for persistence of coronary artery aneurysms despite IVIG treatment.

## Methods

### Study populations and primary outcome

We performed whole genome sequencing in 504 KD patients who were diagnosed and treated with IVIG (2 g/Kg on a single infusion) and aspirin^36^, both using the American Heart Association (AHA) criteria^1,59^. All patients included in the study had echocardiography data that assessed for coronary artery aneurysm (CAA) (212 Whites, 75 Asians, 50 Hispanics and 32 Blacks). Coronary artery internal diameters in the left main coronary artery (LMCA), left anterior descending artery (LAD), and right coronary artery (RCA) obtained by echocardiography. Coronary artery dimensions were normalized for body surface area and converted to z- scores (SDs from a predicted normal mean) based on nonlinear regression equations derived from a normal nonfebrile population^60^. Echocardiographic data were collected at baseline, at 2 weeks, and 5–6 weeks or after following fever onset. The > 5 -6 week echocardiograms were assessed for persistence of the CAAs. We used the Boston z -score model^1^ and as defined by AHA, coronary abnormality or aneurysm was considered if z ≥ 2.5. According to AHA guidelines, we categorized positive or negative coronary artery aneurysm (CAA) occurring at any time point with z score ≥ 2.5 as “CAA”. Persistent CAA (P-CAA) was defined as any aneurysm z ≥ 2.5 for upto 5–6 weeks. We also categorized large CAA (CAA/L) as medium to giant coronary aneurysm for a z score ≥5.0 at any time; and then persistent CCA/L (P-CCA/L) if aneurysm z ≥ 5.0 remained for upto 5–6 weeks. Genomic comparisons were made for each of the 4 categories versus those without any aneurysm (z < 2.5).

The parent cohort/study and this pharmacogenomic study conformed to the procedures for informed consent (parental permission) approved by institutional review boards at all sponsoring organizations .The pharmacogenomic data management and analysis procedure was approved by the University of Alabama at Birmingham Institutional Review Board (IRB). The study was conducted in accordance with the local legislation and institutional requirements following the ethical guidelines of the Declaration of Helsinki. Written informed consent for minor participation (children) in this study was provided by the participants’ legal guardians/next of kin.

### Whole genome sequencing and variant calling

With consent from the parents or legal guardian, whole blood or saliva was collected to extract genomic DNA, as previously described^36^. PCR-free libraries were generated using the BGI DNBSEQ True PCR-Free platform (Beijing Genomics Institute; Guangdong, Shenzhen, China) and whole genome sequencing was performed on the MGISEQ-2000 instrument (Beijing Genomics Institute; Guangdong, Shenzhen, China) to generate 100 bp paired-end reads, as previously described^36^. All reads that passed were aligned to the human reference genome (hg38) using Burrows-Wheeler aligner (BWA) v 0.7.17. The average sequencing depth was 30x per individual. Broad Institute’s Genome Analysis Tool Kit (GATK) best practices workflow was used for quality control and informatics pre-processing of the data. Variant-level QC was performed using the Variant Quality Score Recalibration tool (VQSR) from the Genome Analysis Toolkit (GATK), using the recommended threshold of 99% sensitivity for the “true” variant. As we previously reported, we included 5 duplicate samples, which showed overall high SNP genotype concordance, with a kinship coefficient estimate, Φ > 0.497 between duplicates. For the SNPs we report association, the concordant genotypes were confirmed between all duplicate samples.

### Whole genome sequencing (WGS) association—single-variant analysis

Intensive quality control of the genetic data including minor allele frequency (MAF), call rate (CR), and *p* values of Hardy-Weinberg equilibrium (HWE), were applied to filter uncertain SNPs as described previously^36^, resulting in 46,718,826 variants (21,675,492 singletons) and 25,043,334 polymorphic SNPs were included in the analysis. Logistic regressions models were conducted using PLINK 1.90 to examine the association of individual autosomal SNPs using an additive model in a case/control design, for the main outcome (medium/giant aneurysm, 91 cases (52 W, 3AA, 20His, 16As) and 278 controls (160 W, 29AA, 30His, 59As) and the three secondary outcomes (Table 1). Age, gender and three principal components (PCAs) of genetic ancestry were adjusted in the models. Quantile-quantile (QQ) plots and Manhattan plots were produced with the qqman package in R. The crude and adjusted odds ratios (ORs) and 95% confidence intervals (CIs) were also calculated for the top hits using unconditional univariate logistic regression analysis to evaluate the associations between genotypes and medium/giant aneurysm.

### Identification of genes and their roles using FUMA

Functional annotation was conducted in Functional Mapping and Annotation (FUMA) v1.3.0^61^, using variants of interest from the WGS association analysis (*p* < 1.0 × 10^−5^ and all variants in *r*^2^ < 0.6 with them). Lead SNPs were defined from these independent statistically significant SNPs if pairwise SNPs had r^2^ < 0.1. The maximum distance between LD blocks to merge into a genomic locus was 250 kb. The genetic data of mixed population in 1000 G phase3 were used as reference to estimate LD. Three methods were used to map SNPs to genes: (a) physical distance (within a 10-kb window) from known protein-coding genes in the human reference assembly, (b) expression quantitative trait loci (eQTL) variant mapping using^62^, and (c) 3D chromatin interaction mapping (Hi-C)^63^. Combined Annotation-Dependent Depletion (CADD) analysis^64^ with a minimum score of >12.37 (considered to be suggestive deleterious) was used to filter the variants. Annotation of enhancers^65^, tissue-specific expression of genes identified via Hi-C and eQTL mapping^62^ were queried in FUMA tool and Genotype Tissue Expression (GTEx) database (https://gtexportal.org/home/).

### Genetic risk score computation

All genomic risk loci from FUMA were used to estimate gene risk score (GRS) by a simple risk alleles count method. Discriminative power attributable to the GRS was estimated and compared by plotting receiver operating characteristic (ROC) curves and calculating the area under the curve (AUC) for the case-control samples. The AUC compares the rates of true positives (sensitivity) and false positives (1—specificity) and assesses the overall performance of genetic risk score models. Next, case and control status was randomly permuted 10,000 times and AUC was estimated with each pseudo case-control status. An empirical p-value (eP), which is the proportion of AUC based on randomization distribution of cases and controls that are more extreme than our observed AUC from the actual case (medium/giant aneurysm) and control (no aneurysm) status, was then calculated.

### Reporting summary

Further information on research design is available in the Nature Research Reporting Summary linked to this article.

### Supplementary information

Supplemental Material

Reporting Summary

## Data Availability

The data have been deposited with links to BioProject accession number PRJNA1055092 in the NCBI BioProject database (https://www.ncbi.nlm.nih.gov/bioproject/). The datasets used and/or analyzed in this study are also available from the corresponding author on reasonable request.

## Code availability

Analytical codes will be available upon request from the Corresponding author.
