# Supplementary material for: Pharmacogenomics of coronary artery response to intravenous gamma globulin in kawasaki disease
Source: NPJ Genom Med. 2024 May 30;9:34. doi: 10.1038/s41525-024-00419-7 (PMC11139870; doi:10.1038/s41525-024-00419-7)
Supplement: Supplementary file 1 — Supplemental Material [file 41525_2024_419_MOESM1_ESM.pdf]

Supplementary Figures

Supplementary Figure S1. Heat map of 48 prioritized genes based on FUMA related to 12 genomic risk loci in 54 different tissues. The tissue-specific expression patterns are based on GTEx v6 expression data. Cells in red represent a higher expression, compared to the cells in blue. Gene expression comparisons between tissues (horizontal comparison) within a gene (y axis) are comparable but not those of different genes within a tissue (vertical comparison).

Supplementary Figure S2 QQ plot of whole genome sequence association analysis of large aneurysm in IVIG-treated Kawasaki Disease. Q-Q plot shows observed distributions of association statistics (-log(p)) in y-axis against those expected (-log(p)) in x-axis under the fitted model.

Supplementary Tables

Supplementary Table S1. Independent SNPs (r2 < 0.6) associated with large (medium/giant) aneurysm

Supplementary Table S2. Genomic risk loci of interest from large (medium/giant) aneurysm WGS Association

Supplementary Table S3. All genes mapped in SNP-based (FUMA) for large (medium/giant) aneurysm

Supplementary Table S4. Expression values of the genes prioritized by FUMA for large (medium/giant) aneurysm in 54 tissues

Supplementary Table S5. Minor allele frequencies of cases and control design for 4 CAA phenotype

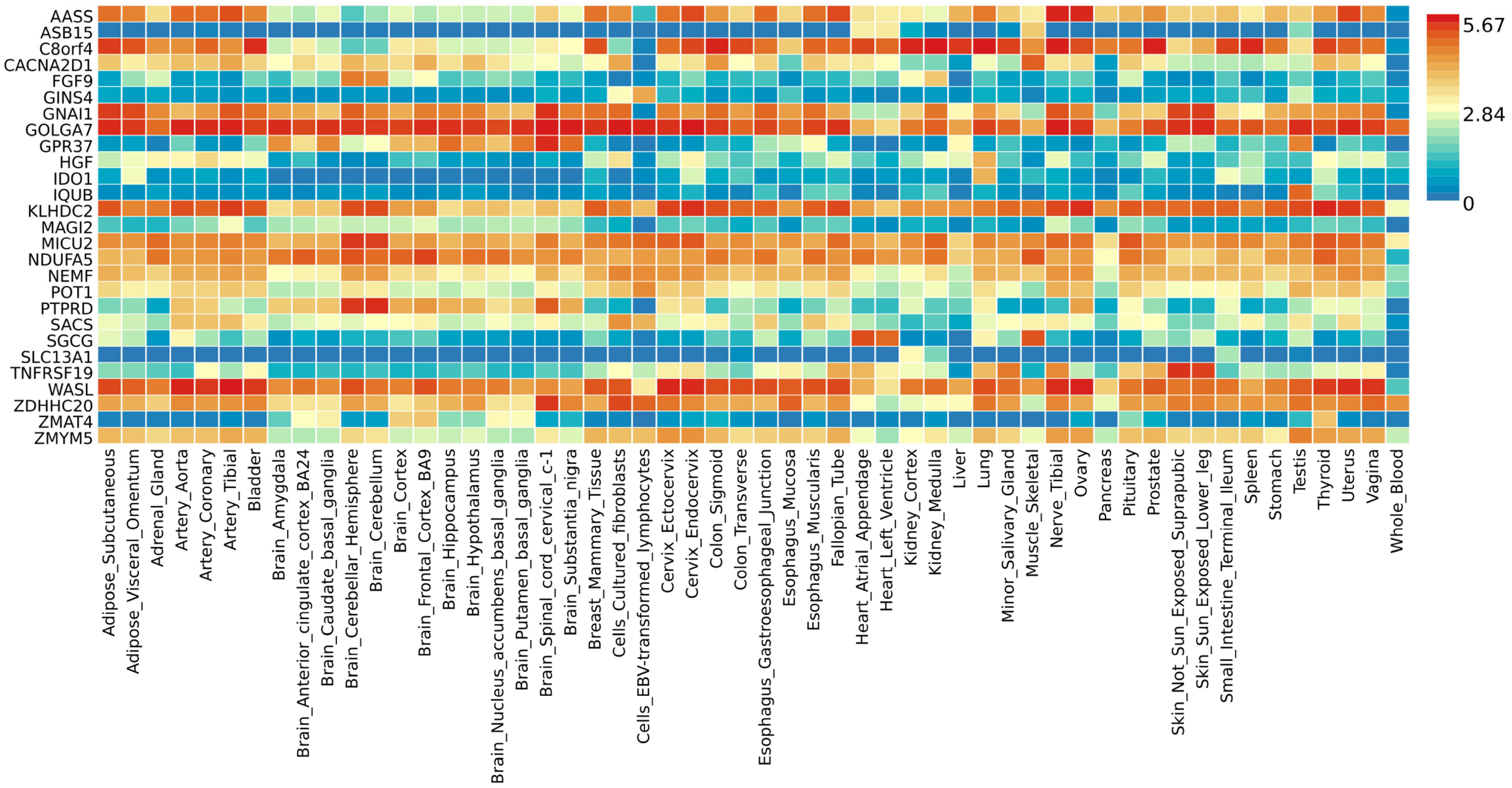

Supplementary Figure S1. Heat map of 48 prioritized genes based on FUMA related to 12 genomic risk loci in 54 different tissues. The tissue-specific expression patterns are based on GTEx v6 expression data. Cells in red represent a higher expression, compared to the cells in blue. Gene expression comparisons between tissues (horizontal comparison) within a gene (y axis) are comparable but not those of different genes within a tissue (vertical comparison).

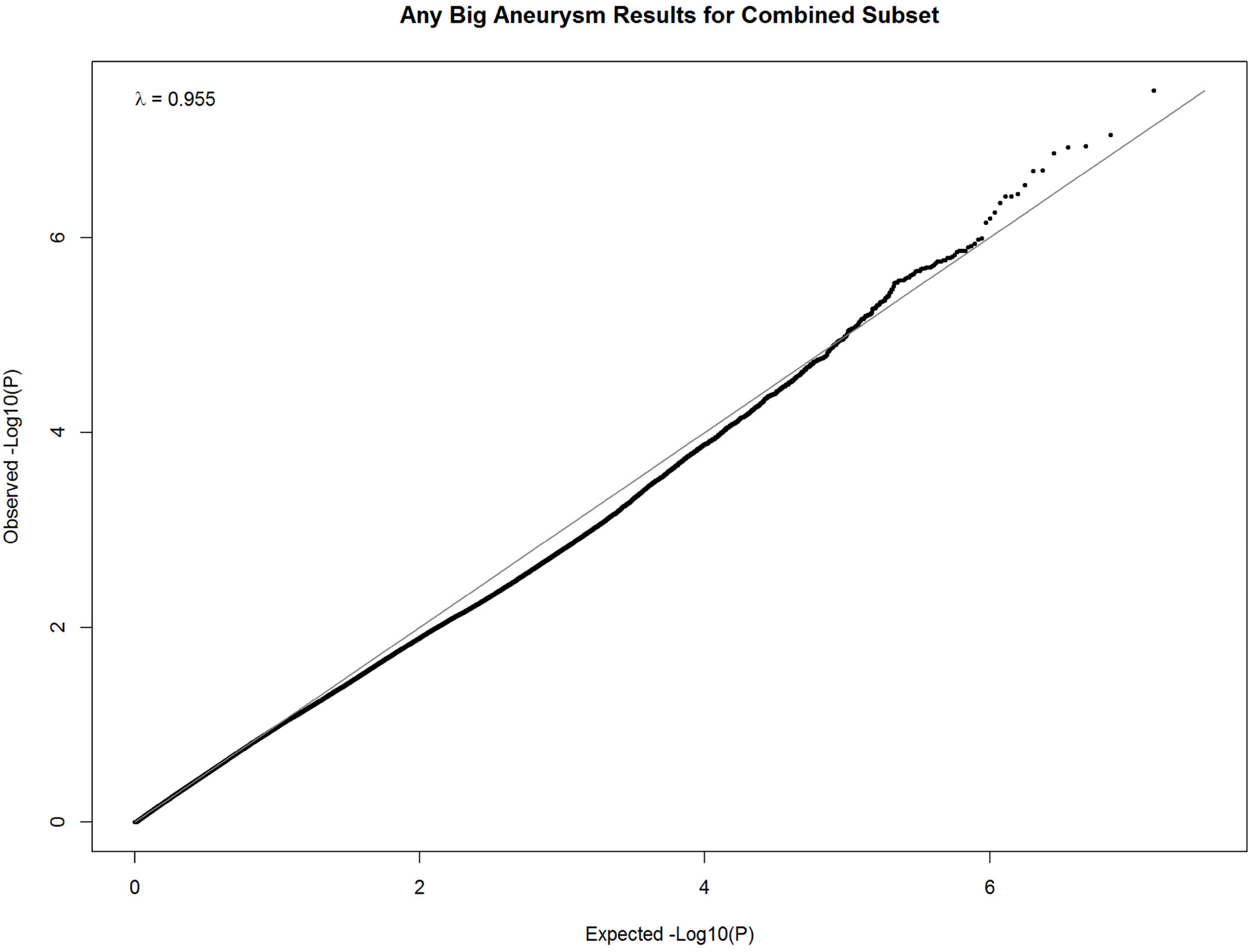

Supplementary Figure S2 QQ plot of whole genome sequence association analysis of large aneurysm in IVIG-treated Kawasaki Disease. Q-Q plot shows observed distributions of association statistics (-log(p)) in y-axis against those expected (-log(p)) in x-axis under the fitted model.

Supplementary Table S1. Independent SNPs (r2 < 0.6) associated with large (medium/giant) aneurysm

Chromosome	Hg38 Genomic Position	SNP	Medium/Big Anewrysm (beta) - Z >5.0	Medium/Big Anewrysm (p-value) - Z >5.0	Persistent Medium/Big Anewrysm (beta) - Z >5.0	Persistent Medium/Big Anewrysm (p-value) - Z >5.0	Any Anewrysm (beta) - Z >2.5	Any Anewrysm (p-value) - Z >2.5	Persistent Any Anewrysm (beta) - Z >2.5	Persistent Any Anewrysm (p-value) - Z >2.5	Func.refGene	Gene/Nearest Gene	Hg19 Genomic Position	Reference Allele in 1000 Genome	Alt Allele in 1000 Genome	Overall MAF	East Asiant MAF	Admixed Americans MAF	African MAF	Europeans MAF	South Asians MAF
2	92065500	rs62154092	-1.751	6.32e-08	-1.853	3.22e-08	-1.45	3.25e-08	-1.587	3.28e-08	intergenic	ACTR3BP2;NONE	NA	NA	NA	NA	NA	NA	NA	NA	NA
20	29360719	rs1424006606	-1.868	1.71e-07	-1.671	3.37e-06	-0.9284	6.78e-06	-1.225	1.53e-06	intergenic	FRG1DP;FRG2EP	NA	NA	NA	NA	NA	NA	NA	NA	NA
12	10436048	rs28730284	-1.695	2.2e-07	-1.607	1.96e-06	-0.9965	6.34e-07	-1.052	6.86e-06	upstream	KLRC2	NA	NA	NA	NA	NA	NA	NA	NA	NA
20	29679725	rs1396081550	-1.42	2.28e-07	-1.508	1.97e-07	-1.045	3.01e-07	-1.061	4.14e-06	intergenic	M242B;MIR447	NA	NA	NA	NA	NA	NA	NA	NA	NA
4	154148395	rs62330192	-1.636	2.59e-07	-1.627	1.09e-06	-0.9791	1.14e-06	-1.182	7.82e-07	intergenic	SFRP2;DCHS2	155069547	G	A	0.000798722	0	0	0.003	0	0
20	28616821	rs1258107032	1.751	3.83e-07	1.81	1.04e-06	0.9922	7.6e-07	1.236	4.8e-07	intergenic	FRG1CP;FRG1DP	NA	NA	NA	NA	NA	NA	NA	NA	NA
20	29821717	rs1379390981	-1.579	3.86e-07	-1.591	8.74e-07	-1.293	6.23e-08	-1.413	1.93e-07	intergenic	M242B;MIR447	NA	NA	NA	NA	NA	NA	NA	NA	NA
20	28616815	rs1424309393	1.729	5.37e-07	1.786	1.45e-06	1.007	6.02e-07	1.228	5.86e-07	intergenic	FRG1CP;FRG1DP	NA	NA	NA	NA	NA	NA	NA	NA	NA
8	40717160	rs9643846	1.416	6.57e-07	1.426	1.1e-06	0.8328	5.33e-05	0.713	0.001965	intronic	ZMAT4	40574679	T	G	0.24381	0.378	0.2594	0.3026	0.0686	0.1953
3	110637278	rs377109159	1.526	6.86e-07	1.656	2.23e-07	1.04	8.01e-06	1.25	1.25e-06	intergenic	R4445;NECTIN3-	NA	NA	NA	NA	NA	NA	NA	NA	NA
3	110637283	rs796814369	1.526	6.86e-07	1.656	2.23e-07	1.041	7.95e-06	1.231	1.89e-06	intergenic	R4445;NECTIN3-	NA	NA	NA	NA	NA	NA	NA	NA	NA
13	18725871	rs1240713470	-1.631	8e-07	-1.668	1.05e-06	-1.191	6.28e-06	-1.4	1.48e-06	intergenic	M230C;LINC004	NA	NA	NA	NA	NA	NA	NA	NA	NA
20	29078317	rs1205686479	1.365	9.91e-07	1.341	4.43e-06	0.844	1.39e-05	0.9336	2.77e-05	intergenic	FRG1CP;FRG1DP	NA	NA	NA	NA	NA	NA	NA	NA	NA
8	40717476	rs9643847	1.373	1.14e-06	1.351	3.19e-06	0.763	0.0001894	0.6896	0.00262	intronic	ZMAT4	40574995	G	A	0.23742	0.378	0.2594	0.2776	0.0696	0.1953
13	18725870	rs1202400626	-1.616	1.24e-06	-1.641	1.87e-06	-1.206	6.08e-06	-1.399	1.89e-06	intergenic	M230C;LINC004	NA	NA	NA	NA	NA	NA	NA	NA	NA
8	40723183	rs57504215	1.357	1.78e-06	1.362	3.04e-06	0.7797	0.0001609	0.6398	0.005597	intronic	ZMAT4	40580702	G	A	0.24401	0.379	0.2579	0.3033	0.0686	0.1953
8	40736410	rs60545202	1.363	1.82e-06	1.377	2.97e-06	0.7582	0.000347	0.6856	0.003374	intronic	ZMAT4	40593929	A	G	0.209065	0.3829	0.2579	0.1679	0.0686	0.1953
10	38635837	rs375244917	1.373	2.01e-06	1.528	9.58e-07	0.7872	4.71e-05	1.115	1.33e-06	intergenic	NC00999;ACTR3B	NA	NA	NA	NA	NA	NA	NA	NA	NA
8	40752382	rs59556769	1.316	2.11e-06	1.353	2.18e-06	0.6968	0.0006387	0.6806	0.002665	intronic	ZMAT4	40609901	G	A	0.217053	0.38	0.2579	0.1884	0.0686	0.2117
7	81633584	rs10276547	1.713	2.17e-06	1.78	1.34e-06	1.009	0.0004307	1.357	1.78e-05	ncRNA_intronic	LOC100128317	81262900	C	T	0.081869	0.0278	0.0591	0.1732	0.0497	0.0634
8	40759531	rs73677451	1.309	2.36e-06	1.315	4.14e-06	0.7301	0.0003341	0.6836	0.002503	intronic	ZMAT4	40617050	G	T	0.217851	0.381	0.2579	0.1906	0.0686	0.2117
9	10297519	rs600075	1.277	2.36e-06	1.267	6.45e-06	0.8336	1.79e-05	0.8799	8.24e-05	intronic	PTPRD	10297519	G	A	0.254992	0.2133	0.3242	0.3593	0.0716	0.2965
9	10298389	rs5896385	1.277	2.36e-06	1.267	6.45e-06	0.8336	1.79e-05	0.8799	8.24e-05	intronic	PTPRD	10298389	C	CA	0.25619	0.2143	0.3242	0.3608	0.0716	0.2996
8	40747480	rs12676292	1.331	2.42e-06	1.345	3.92e-06	0.7368	0.0004134	0.6936	0.002612	intronic	ZMAT4	40604999	C	T	0.21266	0.38	0.2579	0.1785	0.0686	0.2025
7	60921543	rs1327573902	-1.543	2.61e-06	-1.616	5.18e-06	-0.9498	3.46e-06	-1.112	5.37e-06	intergenic	NONE;NONE	NA	NA	NA	NA	NA	NA	NA	NA	NA
8	40732960	rs4332118	1.344	2.68e-06	1.384	2.74e-06	0.7737	0.0002607	0.6614	0.004792	intronic	ZMAT4	40590479	C	T	0.209864	0.381	0.2594	0.171	0.0686	0.1963
7	142457900	rs1218424730	1.315	2.76e-06	1.497	4.47e-07	0.8564	2.61e-05	1.199	2.47e-07	intronic	TCAF2	NA	NA	NA	NA	NA	NA	NA	NA	NA
8	40715671	rs6988966	1.316	2.78e-06	1.351	3.19e-06	0.7157	0.0004386	0.6265	0.006265	intronic	ZMAT4	40573190	G	A	0.23762	0.374	0.2594	0.2784	0.0686	0.2004
22	48342321	rs35056946	1.34	2.92e-06	1.238	3.24e-05	0.7595	0.0002046	0.8774	0.000213	intergenic	MIR3201;TAFA5	48738133	T	G	0.217053	0.3343	0.1254	0.292	0.1193	0.1605
7	81625474	rs10280266	1.682	2.93e-06	1.777	1.36e-06	0.9986	0.0004874	1.346	2.08e-05	ncRNA_intronic	LOC100128317	81254790	A	T	0.0816693	0.0278	0.0591	0.174	0.0477	0.0634
8	40737645	rs12677604	1.329	2.98e-06	1.372	2.94e-06	0.7574	0.0003499	0.6633	0.004504	intronic	ZMAT4	40595164	G	A	0.220647	0.3839	0.2579	0.1989	0.0686	0.2117
8	40735098	rs61320655	1.336	2.99e-06	1.377	2.97e-06	0.7645	0.0003235	0.6665	0.004435	intronic	ZMAT4	40592617	T	G	0.210463	0.3829	0.2579	0.1725	0.0686	0.1963
8	40736329	rs12541699	1.336	2.99e-06	1.377	2.97e-06	0.7444	0.0004492	0.6665	0.004435	intronic	ZMAT4	40593848	A	T	0.209265	0.3829	0.2579	0.1679	0.0686	0.1963
7	57683789	rs77925841	-1.384	3.1e-06	-1.326	1.94e-05	-1.053	1.13e-07	-1.063	4.52e-06	intergenic	ZNF716;NONE	NA	NA	NA	NA	NA	NA	NA	NA	NA
7	60921548	rs1396220338	-1.516	3.27e-06	-1.565	7.82e-06	-0.9831	1.85e-06	-1.17	2.17e-06	intergenic	NONE;NONE	NA	NA	NA	NA	NA	NA	NA	NA	NA
7	60921547	rs1441248959	-1.508	3.34e-06	-1.56	7.77e-06	-0.9147	7.14e-06	-1.107	5.77e-06	intergenic	NONE;NONE	NA	NA	NA	NA	NA	NA	NA	NA	NA
8	40738114	rs60311345	1.318	3.41e-06	1.357	3.51e-06	0.7228	0.000583	0.6556	0.004872	intronic	ZMAT4	40595633	G	A	0.217652	0.381	0.2579	0.1899	0.0686	0.2117
3	75853862	rs10451979	-1.277	3.42e-06	-1.241	1.59e-05	-0.93	2.56e-06	-1.01	7.32e-06	intergenic	ZNF717;ROBO2	NA	NA	NA	NA	NA	NA	NA	NA	NA
9	10285669	rs193014517	2.259	3.43e-06	2.255	4.8e-06	1.515	7.78e-05	1.699	7.5e-05	intronic	PTPRD	10285669	G	A	0.11262	0.0407	0.2363	0.1974	0.006	0.0941
2	131644399	rs1342924635	-1.339	3.47e-06	-1.494	1.71e-06	-0.8132	2.69e-05	-0.9198	5.2e-05	ncRNA_intronic	LINC01087	NA	NA	NA	NA	NA	NA	NA	NA	NA
7	81631122	rs10264538	1.79	3.52e-06	1.843	2.67e-06	1.165	0.0001765	1.516	7.75e-06	ncRNA_intronic	LOC100128317	81260438	C	G	0.0800719	0.0179	0.0591	0.174	0.0497	0.0634
9	10298344	rs647328	1.246	3.52e-06	1.267	6.45e-06	0.8263	2.09e-05	0.8672	0.0001017	intronic	PTPRD	10298344	G	A	0.25639	0.2143	0.3242	0.3608	0.0716	0.3006
8	40736611	rs62497418	1.322	3.55e-06	1.362	3.58e-06	0.7401	0.0004785	0.6861	0.003539	intronic	ZMAT4	40594130	T	C	0.221246	0.3829	0.2594	0.2126	0.0686	0.1963
8	40758138	rs6988685	1.284	3.72e-06	1.315	4.14e-06	0.7177	0.0004246	0.6665	0.003249	intronic	ZMAT4	40615657	C	T	0.217452	0.3819	0.2579	0.1884	0.0686	0.2117
8	40758619	rs4341150	1.284	3.72e-06	1.315	4.14e-06	0.6822	0.0008377	0.6339	0.005512	intronic	ZMAT4	40616138	A	C	0.218051	0.3819	0.2579	0.1906	0.0686	0.2117
8	40760499	rs12549678	1.284	3.72e-06	1.315	4.14e-06	0.7177	0.0004246	0.6665	0.003249	intronic	ZMAT4	40618018	G	A	0.217452	0.3819	0.2579	0.1884	0.0686	0.2117
20	28616805	rs1301447577	1.459	3.76e-06	1.468	1.04e-05	0.9211	3.46e-06	1.207	5.99e-07	intergenic	FRG1CP;FRG1DP	NA	NA	NA	NA	NA	NA	NA	NA	NA
17	26719834	rs1157832314	-1.289	3.95e-06	-1.307	8.46e-06	-0.9226	7.59e-06	-1.052	7.14e-06	intergenic	ONE;LOC1053717	NA	NA	NA	NA	NA	NA	NA	NA	NA
8	40756547	rs12678434	1.28	4.05e-06	1.311	4.46e-06	0.6978	0.0006676	0.6332	0.005689	intronic	ZMAT4	40614066	C	T	0.217452	0.3819	0.2579	0.1884	0.0686	0.2117
20	29078321	rs1262415727	1.268	4.09e-06	1.225	2.02e-05	0.8246	2.2e-05	0.8882	6.42e-05	intergenic	FRG1CP;FRG1DP	NA	NA	NA	NA	NA	NA	NA	NA	NA
13	21531367	rs12585631	-0.9647	4.11e-06	-1.045	3.69e-06	-0.6562	5.28e-06	-0.7247	1.28e-05	intronic	MICU2	22105506	T	A	0.287939	0.3036	0.3357	0.059	0.4215	0.41
9	43186651	rs1463676990	1.255	4.3e-06	1.132	7.63e-05	0.9248	3.44e-06	0.8113	0.000363	intergenic	C102724904;NO	NA	NA	NA	NA	NA	NA	NA	NA	NA
21	7948712	rs1329708049	-1.554	4.32e-06	-1.369	5.87e-05	-0.6551	0.0009612	-0.9456	9.78e-05	intergenic	NE1B;LOC100507	NA	NA	NA	NA	NA	NA	NA	NA	NA
1	30360799	rs634288	1.267	4.38e-06	1.281	5.83e-06	0.8194	9.62e-05	1.005	2.12e-05	intergenic	INC01648;MATN	30833646	A	G	0.811701	0.8413	0.8156	0.6906	0.8956	0.8558
1	30355994	rs1754156	1.284	4.45e-06	1.299	5.94e-06	0.842	7.61e-05	1.005	2.97e-05	intergenic	INC01648;MATN	30828841	G	A	0.807508	0.8413	0.8112	0.6762	0.8956	0.8569
17	21715387	rs1480649071	1.366	4.56e-06	1.366	1.06e-05	0.8869	0.0001585	1.082	3.43e-05	intergenic	KCNJ18;UBBP4	NA	NA	NA	NA	NA	NA	NA	NA	NA
8	40738554	rs56847872	1.294	4.58e-06	1.334	4.66e-06	0.7188	0.0005897	0.639	0.005839	intronic	ZMAT4	40596073	T	G	0.217652	0.381	0.2579	0.1899	0.0686	0.2117
8	40742744	rs7832204	1.294	4.58e-06	1.334	4.66e-06	0.7308	0.0004434	0.6634	0.003935	intronic	ZMAT4	40600263	C	T	0.217452	0.381	0.2579	0.1899	0.0686	0.2106
8	40743012	rs7831620	1.294	4.58e-06	1.334	4.66e-06	0.7389	0.0003882	0.6634	0.003935	intronic	ZMAT4	40600531	G	A	0.217652	0.381	0.2594	0.1899	0.0686	0.2106
8	40745809	rs12679157	1.294	4.58e-06	1.334	4.66e-06	0.7181	0.0005514	0.6634	0.003935	intronic	ZMAT4	40603328	G	A	0.217452	0.381	0.2579	0.1891	0.0686	0.2117
8	40750391	rs58008678	1.294	4.58e-06	1.334	4.66e-06	0.7181	0.0005514	0.6634	0.003935	intronic	ZMAT4	40607910	C	T	0.217452	0.38	0.2579	0.1899	0.0686	0.2117
7	60921542	rs1444241839	-1.505	4.65e-06	-1.576	8.87e-06	-0.8841	1.4e-05	-1.071	1.19e-05	intergenic	NONE;NONE	NA	NA	NA	NA	NA	NA	NA	NA	NA
7	123570563	rs34163760	1.304	4.84e-06	1.248	3.79e-05	0.6764	0.001613	0.9077	0.0001191	intronic	NDUFA5	123210617	G	A	0.164736	0.0714	0.0994	0.2315	0.1421	0.2403
8	40746986	rs6995809	1.29	4.88e-06	1.33	4.96e-06	0.7152	0.000582	0.6602	0.004113	intronic	ZMAT4	40604505	C	G	0.217851	0.381	0.2579	0.1906	0.0686	0.2117
8	40746988	rs6995810	1.29	4.88e-06	1.33	4.96e-06	0.7152	0.000582	0.6602	0.004113	intronic	ZMAT4	40604507	C	A	0.217851	0.381	0.2579	0.1906	0.0686	0.2117
14	49838862	rs117885021	1.803	4.93e-06	1.877	3.94e-06	1.213	0.0003642	1.528	2.82e-05	intronic	NEMF	50305580	G	A	0.034345	0.002	0.0288	0.0038	0.0616	0.0849
9	10335382	rs1590347	1.181	5.29e-06	1.232	5.47e-06	0.8076	2.49e-05	0.859	8.37e-05	intronic	PTPRD	10335382	C	T	0.277556	0.1964	0.3213	0.4675	0.0736	0.2832
20	29360731	rs1479209115	-1.463	5.62e-06	-1.3	6.41e-05	-0.6844	0.000552	-0.9961	3.77e-05	intergenic	FRG1DP;FRG2EP	NA	NA	NA	NA	NA	NA	NA	NA	NA
14	49775737	rs143665261	1.787	5.7e-06	1.856	4.77e-06	1.201	0.0004088	1.507	3.46e-05	intronic	KLHDC2	50242455	G	A	0.0339457	0.002	0.0274	0.0038	0.0616	0.0838
1	30349321	rs377203	1.27	6.05e-06	1.287	7.93e-06	0.8258	0.0001081	1.009	3.03e-05	intergenic	INC01648;MATN	30822168	T	C	0.824281	0.8413	0.8184	0.736	0.8956	0.8569
7	142457899	rs1276772253	1.255	6.1e-06	1.432	9.91e-07	0.8365	3.66e-05	1.156	5.66e-07	intronic	TCAF2	NA	NA	NA	NA	NA	NA	NA	NA	NA
7	100950922	rs79702292	1.854	6.4e-06	2.405	9.68e-06	0.9506	1.45e-05	1.041	8.46e-05	intronic	MUC3A	NA	NA	NA	NA	NA	NA	NA	NA	NA
7	100950935	rs78451619	1.697	6.57e-06	1.891	1.07e-05	0.8422	6.38e-05	0.9107	0.0002852	intronic	MUC3A	NA	NA	NA	NA	NA	NA	NA	NA	NA
17	21709814	rs1422393541	1.274	6.61e-06	1.25	2.43e-05	0.8734	6e-06	0.8409	0.000168	intergenic	KCNJ18;UBBP4	NA	NA	NA	NA	NA	NA	NA	NA	NA
7	81644107	rs10276241	1.629	6.76e-06	1.693	4.4e-06	0.9324	0.001148	1.312	3.61e-05	ncRNA_intronic	LOC100128317	81273423	C	T	0.0816693	0.0278	0.0591	0.1725	0.0497	0.0634
9	10321993	rs12003726	2.159	6.83e-06	2.173	8.71e-06	1.25	0.0004335	1.543	0.000198	intronic	PTPRD	10321993	A	G	0.117212	0.0466	0.2464	0.2156	0.007	0.0787
1	5673103	rs72863358	1.307	7.23e-06	1.383	5.04e-06	0.7758	0.0006379	0.8988	0.0003949	intergenic	AJAP1;MIR4689	NA	NA	NA	NA	NA	NA	NA	NA	NA
11	45718866	rs36228357	1.234	7.24e-06	1.403	8.59e-07	0.6532	0.002224	0.8398	0.0004212	intergenic	7154;LOC100507	45740416	CCACTCCT	C	0.168331	0.1885	0.2853	0.1747	0.1024	0.1237
1	63209243	rs1328589	-1.013	7.33e-06	-1.055	1.15e-05	-0.4107	0.003004	-0.607	0.0003025	ncRNA_intronic	LINC00466	63674914	T	G	0.555711	0.5079	0.5908	0.5499	0.6759	0.4642

20	29502968	rs1467766855	-1.245	7.47e-06	-1.205	3.47e-05	-0.7523	0.0003291	-0.8072	0.0006768	intergenic	FRG1EP;FAM242I	NA	NA	NA	NA	NA	NA	NA	NA	NA
8	40738067	rs12677682	1.267	7.48e-06	1.302	8.06e-06	0.7312	0.0005482	0.6343	0.006639	intronic	ZMAT4	40595586	G	A	0.214856	0.381	0.2579	0.1793	0.0686	0.2117
9	10307194	rs35433273	2.01	7.51e-06	2.051	7.07e-06	1.255	0.0002578	1.574	6.4e-05	intronic	PTPRD	10307194	CA	C	0.150958	0.0446	0.245	0.3464	0.006	0.0787
13	21529155	rs12869776	-0.9602	7.58e-06	-1.045	6.23e-06	-0.6538	9.37e-06	-0.7183	2.7e-05	intronic	MICU2	22103294	A	G	0.294928	0.3046	0.3415	0.087	0.4115	0.4131
7	60921539	rs1231228837	-1.471	7.85e-06	-1.542	1.43e-05	-0.9052	1.05e-05	-1.088	1.02e-05	intergenic	NONE;NONE	NA	NA	NA	NA	NA	NA	NA	NA	NA
13	21596136	rs11148172	-0.9446	7.95e-06	-1.019	7.59e-06	-0.6376	9.8e-06	-0.671	5.25e-05	intronic	MICU2	22170275	C	T	0.3127	0.3036	0.3372	0.1536	0.4175	0.4121
6	29956382	rs41265804	1.054	8.05e-06	1.083	9.93e-06	0.6769	4.92e-05	0.7879	3.44e-05	intergenic	HLA-A;HCG9	29924159	C	G	0.226238	0.2688	0.2118	0.2194	0.1978	0.2311
20	30865049	rs1980848880	-1.819	8.1e-06	-1.804	2.89e-05	-1.12	9.36e-07	-1.505	4.95e-07	intergenic	FRG1BP;DEFB115	NA	NA	NA	NA	NA	NA	NA	NA	NA
6	53726346	rs114644621	1.812	8.21e-06	1.924	3.1e-06	1.41	3.58e-05	1.324	0.0003521	intergenic	KLHL31;LRRC1	53591144	A	G	0.0299521	0	0.0663	0.0023	0.0944	0.0061
13	21497559	rs2025243	-0.97	8.35e-06	-1.065	6.02e-06	-0.6218	2.75e-05	-0.6942	5.39e-05	intronic	MICU2	22071698	A	G	0.283946	0.3026	0.3343	0.053	0.4115	0.41
7	81647015	rs10236154	1.604	8.65e-06	1.693	4.4e-06	0.9264	0.001225	1.303	4.06e-05	ncRNA_intronic	LOC100128317	81276331	G	A	0.0814696	0.0268	0.0591	0.1725	0.0497	0.0634
7	81626687	rs10247514	1.604	8.67e-06	1.694	4.4e-06	0.9725	0.000727	1.303	4.1e-05	ncRNA_intronic	LOC100128317	81256003	C	T	0.0820687	0.0278	0.0576	0.1755	0.0477	0.0644
9	10327894	rs1951785	2.258	8.69e-06	2.221	1.55e-05	1.365	0.0001937	1.634	0.0001625	intronic	PTPRD	10327894	T	C	0.120807	0.0476	0.2478	0.2118	0.007	0.1002
7	62309248	rs138547582	-1.264	8.74e-06	-1.327	8.67e-06	-1.034	1.79e-06	-1.017	3.25e-05	intergenic	NONE;ZNF733P	61753499	G	A	0.000199681	0	0	0	0.001	0
17	21320260	rs79263592	1.253	8.77e-06	1.304	1.29e-05	0.9059	2.92e-06	0.9581	2.23e-05	intergenic	MAP2K3;KCNJ12	NA	NA	NA	NA	NA	NA	NA	NA	NA
1	200689016	rs151019013	-1.109	9.51e-06	-1.091	3.45e-05	-0.4421	0.003095	-0.7403	6.41e-05	ncRNA_intronic	DDX59-AS1	NA	NA	NA	NA	NA	NA	NA	NA	NA
13	21523314	rs60181111	-0.9454	9.67e-06	-1.03	7.91e-06	-0.6345	1.56e-05	-0.694	4.46e-05	intronic	MICU2	22097453	A	G	0.295927	0.3046	0.3415	0.0908	0.4145	0.41
13	21528837	rs10492455	-0.9454	9.67e-06	-1.03	7.91e-06	-0.6407	1.38e-05	-0.7047	3.73e-05	intronic	MICU2	22102976	A	G	0.296126	0.3046	0.3415	0.0908	0.4125	0.4131
13	21511520	rs718426	-0.956	9.81e-06	-1.067	5.42e-06	-0.6335	1.9e-05	-0.7087	3.64e-05	intronic	MICU2	22085659	A	G	0.290535	0.3036	0.3372	0.0749	0.4125	0.41
7	62309258	rs113910059	-1.273	9.99e-06	-1.366	7.35e-06	-1.117	1.72e-07	-1.163	1.57e-06	intergenic	NONE;ZNF733P	NA	NA	NA	NA	NA	NA	NA	NA	NA
2	161044292	rs10490562	1.05	1e-05	1.053	2.79e-05	0.7582	1.15e-05	0.9148	5.13e-06	intergenic	RBMS1;TANK	161900803	T	C	0.174521	0.247	0.1052	0.1641	0.2038	0.1329
13	21549132	rs4769167	-0.9271	1e-05	-1.034	5.63e-06	-0.6167	1.99e-05	-0.6767	4.83e-05	intronic	MICU2	22123271	T	C	0.301717	0.3204	0.3372	0.1104	0.3956	0.4192
1	24970252	rs10751776	-1.057	1.01e-05	-1.093	1.34e-05	-0.5493	0.0003254	-0.8134	2.28e-05	intergenic	RUNX3;MIR4425	25296743	A	C	0.69988	0.6865	0.7651	0.8094	0.5467	0.6769
13	21532104	rs2798266	-0.921	1.01e-05	-0.9959	8.89e-06	-0.5735	5.8e-05	-0.6806	4.41e-05	intronic	MICU2	22106243	T	C	0.325679	0.3016	0.3458	0.211	0.4036	0.411
4	154148459	rs1207442361	-1.307	1.02e-05	-1.302	3.25e-05	-0.9685	1.49e-06	-1.065	6.7e-06	intergenic	SFRP2;DCHS2	NA	NA	NA	NA	NA	NA	NA	NA	NA
13	21486902	rs34946673	-0.95	1.03e-05	-1.042	7.4e-06	-0.6472	1.07e-05	-0.7126	2.95e-05	intergenic	ZDHHC20;MICU2	22061041	C	T	0.289537	0.3026	0.3256	0.0961	0.3907	0.408
13	21560307	rs147058896	-0.9233	1.03e-05	-1.003	8.55e-06	-0.5409	0.0001335	-0.6259	0.0001448	intronic	MICU2	22134446	CAACTGCACTAGT	A	0.320687	0.3006	0.3444	0.2179	0.3817	0.4008
7	142457894	rs1215719281	1.218	1.06e-05	1.398	1.67e-06	0.8044	6.39e-05	1.112	1.3e-06	intronic	TCAF2	NA	NA	NA	NA	NA	NA	NA	NA	NA
8	40742461	rs60297253	1.246	1.07e-05	1.316	6.29e-06	0.6934	0.0008671	0.6484	0.004825	intronic	ZMAT4	40599980	G	A	0.217452	0.381	0.2579	0.1899	0.0686	0.2106
7	100950924	rs78630534	1.724	1.1e-05	2.172	1.12e-05	0.8937	3.73e-05	0.9459	0.0002562	intronic	MUC3A	NA	NA	NA	NA	NA	NA	NA	NA	NA
9	10281159	rs74345088	2.137	1.1e-05	2.15	1.36e-05	1.545	7.25e-05	1.76	6.72e-05	intronic	PTPRD	10281159	C	G	0.115615	0.0377	0.2378	0.2095	0.006	0.0951
10	119020181	rs504162	0.9261	1.1e-05	0.8565	9.1e-05	0.4436	0.00211	0.5732	0.0007729	intergenic	CACUL1;NANOS1	120779693	A	G	0.56849	0.497	0.6888	0.4758	0.6481	0.6002
13	21499835	rs111475357	0.8908	1.1e-05	0.9596	9.25e-06	0.4783	0.000773	0.617	0.0001835	intronic	MICU2	22073974	CCAA	C	0.48143	0.6627	0.513	0.3411	0.4175	0.5276
1	200630823	rs7527539	-1.06	1.11e-05	-0.9877	7.07e-05	-0.426	0.003686	-0.6575	0.0002074	intergenic	KIF14;DDX59	200599951	C	T	0.255791	0.2827	0.2363	0.1135	0.4056	0.2802
9	63804060	rs78491863	1.314	1.14e-05	1.353	1.96e-05	0.8735	8.34e-06	1.005	1.72e-05	intergenic	100996643;LINC0	NA	NA	NA	NA	NA	NA	NA	NA	NA
22	49774617	rs2269626	2.818	1.16e-05	2.847	1.05e-05	1.56	0.003293	2.019	0.0004735	intronic	BRD1	50168265	G	A	0.0405351	0.0675	0.0187	0.0295	0.007	0.0777
7	100950903	rs79510797	2.02	1.17e-05	2.542	3.4e-05	0.8439	0.0001468	0.9912	0.0002738	intronic	MUC3A	NA	NA	NA	NA	NA	NA	NA	NA	NA
1	124980023	rs1451276664	-1.251	1.2e-05	-1.239	3.83e-05	-0.8433	1.38e-05	-0.9846	1.53e-05	intergenic	NONE;NONE	NA	NA	NA	NA	NA	NA	NA	NA	NA
13	21519685	rs17622791	-0.9281	1.24e-05	-1.01	1.02e-05	-0.6429	1.17e-05	-0.7037	3.5e-05	intronic	MICU2	22093824	T	A	0.287939	0.3046	0.3242	0.0862	0.3926	0.41
1	124980016	rs1236116259	-1.238	1.25e-05	-1.217	4.43e-05	-0.8234	2.09e-05	-0.9555	2.42e-05	intergenic	NONE;NONE	NA	NA	NA	NA	NA	NA	NA	NA	NA
13	18725868	rs2801763	-1.492	1.25e-05	-1.498	2.45e-05	-1.163	1.92e-05	-1.343	7.97e-06	intergenic	AM230C;LINC004	NA	NA	NA	NA	NA	NA	NA	NA	NA
1	200680036	rs4072553	-1.04	1.29e-05	-0.951	0.0001134	-0.4367	0.002783	-0.6704	0.0001475	ncRNA_intronic	DDX59-AS1	200649164	T	C	0.263778	0.2827	0.2363	0.1445	0.4076	0.2771
6	151613479	rs11155800	-1.357	1.29e-05	-1.446	2.12e-05	-0.9659	9.86e-07	-1.123	3.22e-06	intronic	CCDC170	151934614	A	G	0.170727	0.0218	0.1614	0.171	0.2256	0.274
13	21595685	rs2798262	-0.9225	1.29e-05	-0.9945	1.2e-05	-0.5375	0.0001694	-0.6303	0.0001559	intronic	MICU2	22169824	T	C	0.32508	0.3016	0.3444	0.2247	0.3877	0.407
7	57683806	rs74600664	-1.79	1.31e-05	-1.623	8.14e-05	-1.164	5.49e-07	-1.248	9.67e-06	intergenic	ZNF716;NONE	NA	NA	NA	NA	NA	NA	NA	NA	NA
13	21543806	rs71093329	-0.9146	1.33e-05	-1.009	8.59e-06	-0.5419	0.000136	-0.6565	8.06e-05	intronic	MICU2	22117945	G	GAAAA,GAAAT	0.639177,0.3314	0.6944,0.3036	0.6297,0.3617	0.6747,0.2209	0.5915,0.4085	0.59,0.409
13	21502352	rs2315545	-0.9358	1.34e-05	-1.027	9.69e-06	-0.6053	3.7e-05	-0.6676	8.58e-05	intronic	MICU2	22076491	C	T	0.284744	0.3363	0.317	0.0522	0.3926	0.4121
8	40738905	rs4430080	1.167	1.35e-05	1.183	1.95e-05	0.5639	0.00417	0.5553	0.01237	intronic	ZMAT4	40596424	G	A	0.727037	0.619	0.7291	0.6082	0.9304	0.7883
1	200652107	rs12041320	-1.045	1.36e-05	-0.9754	8.31e-05	-0.4187	0.004241	-0.6482	0.0002462	intronic	DDX59	200621235	T	C	0.25599	0.2827	0.2363	0.115	0.4056	0.2791
1	200655372	rs139391672	-1.045	1.36e-05	-0.9754	8.31e-05	-0.4187	0.004241	-0.6482	0.0002462	intronic	DDX59	200624500	CCT	C	0.25639	0.2827	0.2363	0.1157	0.4066	0.2791
7	142457890	rs1278498984	1.19	1.36e-05	1.371	2.08e-06	0.789	7.96e-05	1.106	1.26e-06	intronic	TCAF2	NA	NA	NA	NA	NA	NA	NA	NA	NA
13	21507238	rs12865749	-0.9456	1.36e-05	-1.037	1.01e-05	-0.6142	3.31e-05	-0.6813	7.12e-05	intronic	MICU2	22081377	A	G	0.29353	0.3393	0.3357	0.059	0.4115	0.4121
13	21518623	rs12861578	-0.9256	1.38e-05	-1.03	7.91e-06	-0.6269	1.98e-05	-0.694	4.46e-05	intronic	MICU2	22092762	T	G	0.299521	0.3056	0.3401	0.1059	0.4125	0.41
9	63893835	rs62545564	-1.218	1.39e-05	-1.235	2.96e-05	-0.8393	1.28e-05	-0.9284	3.64e-05	intergenic	FRG1JP;FLJ43315	NA	NA	NA	NA	NA	NA	NA	NA	NA
7	79191714	rs17152038	1.728	1.4e-05	1.759	1.81e-05	1.002	0.0002153	1.037	0.001292	intronic	MAGI2	78821030	T	C	0.127796	0.1508	0.0908	0.3056	0.0119	0.0092
13	21473266	rs2874196	-0.9557	1.4e-05	-1.071	7.5e-06	-0.6959	3.25e-06	-0.7332	2.67e-05	intergenic	ZDHHC20;MICU2	22047405	C	T	0.263978	0.2599	0.3127	0.0492	0.3748	0.41
7	156266214	rs201042550	-1.271	1.41e-05	-1.21	7.43e-05	-0.7503	0.0001813	-0.7346	0.001396	intergenic	C389602;LOC285	NA	NA	NA	NA	NA	NA	NA	NA	NA
14	101079491	rs76540573	1.789	1.41e-05	1.804	1.76e-05	1.333	0.0001711	1.477	0.0001019	intergenic	MEG9;LINC0228	101545828	C	A	0.0267572	0	0.0317	0.0038	0.0895	0.0174
13	21538714	rs2798280	-0.9069	1.43e-05	-0.981	1.25e-05	-0.5479	0.0001254	-0.6372	0.0001275	intronic	MICU2	22112853	T	C	0.332468	0.3016	0.3631	0.2262	0.4076	0.409
22	17026434	rs1441960448	1.22	1.43e-05	1.262	2.04e-05	0.6769	0.0005397	0.8599	0.000134	intergenic	GAB4;CECR7	NA	NA	NA	NA	NA	NA	NA	NA	NA
13	21576070	rs10870842	-0.9095	1.51e-05	-0.9864	1.29e-05	-0.6275	1.66e-05	-0.6574	8e-05	intronic	MICU2	22150209	A	C	0.278355	0.3036	0.3184	0.0545	0.3887	0.4131
13	21530239	rs2181751	0.8801	1.52e-05	1.029	2.47e-06	0.48	0.0009672	0.6724	5.47e-05	intronic	MICU2	22104378	C	T	0.570487	0.2847	0.5749	0.7171	0.7058	0.5245
1	200638338	rs10919992	-1.042	1.56e-05	-0.9727	9.34e-05	-0.4108	0.005132	-0.6421	0.0002943	intergenic	KIF14;DDX59	200607466	A	G	0.25639	0.2837	0.2378	0.1135	0.4066	0.2802
1	124980005	rs1249616454	-1.222	1.6e-05	-1.201	5.49e-05	-0.8412	1.43e-05	-0.9379	3.36e-05	intergenic	NONE;NONE	NA	NA	NA	NA	NA	NA	NA	NA	NA
7	62309251	rs77189369	-1.204	1.63e-05	-1.284	1.19e-05	-0.967	5.12e-06	-0.9427	8.51e-05	intergenic	NONE;ZNF733P	61753502	G	A	0.00239617	0	0	0.0083	0	0.001
7	142457919	rs371146758	1.249	1.63e-05	1.36	6.13e-06	0.9489	1.51e-05	1.16	1.99e-06	intronic	TCAF2	NA	NA	NA	NA	NA	NA	NA	NA	NA
13	21541683	rs11616720	-0.9081	1.64e-05	-0.9851	1.4e-05	-0.6063	2.88e-05	-0.6527	9.03e-05	intronic	MICU2	22115822	A	G	0.288738	0.3046	0.3372	0.0711	0.4085	0.409
15	82837194	rs56108482	1.221	1.64e-05	1.232	2.7e-05	0.7035	0.001275	0.7303	0.002964	intergenic	WHAMM;HOMER	83505946	C	T	0.133586	0.1915	0.1916	0.1256	0.1203	0.0573
13	21524074	rs17622810	-0.9091	1.66e-05	-0.9903	1.34e-05	-0.616	2.36e-05	-0.6652	7.87e-05	intronic	MICU2	22098213	C	T	0.28774	0.3046	0.3242	0.0862	0.3926	0.409
12	94565535	rs7306104	-0.9796	1.68e-05	-0.9616	5.74e-05	-0.7029	6.84e-06	-0.8495	5.08e-06	intergenic	MIR5700;TMCC3	94959311	G	A	0.221845	0.0804	0.2594	0.1278	0.3469	0.3395
7	35926870	rs796838579	-1.237	1.73e-05	-1.183	7.94e-05	-0.7167	0.0001936	-0.9203	5.11e-05	intergenic	TIN7;LOC101928	NA	NA	NA	NA	NA	NA	NA	NA	NA
15	82834411	rs28698473	1.218	1.74e-05	1.206	3.74e-05	0.6453	0.002889	0.7044	0.003981	UTR3	WHAMM	83503163	G	T	0.133586	0.1915	0.1916	0.1256	0.1203	0.0573
7	100950897	rs76849309	1.983	1.75e-05	2.506	4.47e-05	0.8052	0.0002979	0.9514	0.0004864	intronic	MUC3A	NA	NA	NA	NA	NA	NA	NA	NA	NA
7	100950900	rs77075135	1.983	1.75e-05	2.506	4.47e-05	0.8052	0.0002979	0.9514	0.0004864	intronic	MUC3A	NA	NA	NA	NA	NA	NA	NA	NA	NA
7	100950920	rs75915790	1.766	1.75e-05	2.316	2.03e-05	0.8646	8.3e-05	0.9564	0.0003181	intronic	MUC3A	NA	NA	NA	NA	NA	NA	NA	NA	NA
13	21509207	rs9634371	1.038	1.76e-05	1.238	2.44e-06	0.5547	0.001253	0.8429	1.79e-05	intronic	MICU2	22083346	G	A	0.355232	0.6944	0.3545	0.1823	0.166	0.4346
20	30179906	rs1266698617	-1.363	1.77e-05	-1.402	2.07e-05	-1.103	9.1e-06	-1.348	9.35e-07	intergenic	IR4477A;LINC015	NA	NA	NA	NA	NA	NA	NA	NA	NA
13	21468051	rs11148117	-0.9808	1.78e-05	-1.004	3.07e-05	-0.7018	4.14e-06	-0.7016	7.04e-05	intergenic	ZDHHC20;MICU2	22042190	T	A	0.262979	0.2589	0.3112	0.0477	0.3738	0.41
13	21475043	rs1744909	-0.9389	1.78e-05	-1.049	1.01e-05	-0.6098	3.28e-05	-0.6895	6.42e-05	intergenic	ZDHHC20;MICU2	22049182	T	C	0.693091	0.7123	0.6744	0.8079	0.6262	0.6002
1	200679441	rs10800720	-1.024	1.8e-05	-0.951	0.0001134	-0.4302	0.003243	-0.6613	0.0001838	ncRNA_intronic	DDX59-AS1	200648569	T	A	0.263978	0.2827	0.2378	0.1445	0.4076	0.2771
1	200682202	rs10920000	-1.024	1.8e-05	-0.951	0.0001134	-0.4302	0.003243	-0.6613	0.0001838	ncRNA_intronic	DDX59-AS1	200651330	C	G	0.255791	0.2827	0.2349	0.115	0.4076	0.2771
1	200682420	rs10920001	-1.024	1.8e-05	-0.951	0.0001134	-0.435	0.002946	-0.6718	0.0001547	ncRNA_intronic	DDX59-AS1	200651548	C	T	0.254792	0.2788	0.2349	0.1142	0.4076	0.2771
13	21538788	rs2476637	-0.8918	1.81e-05	-0.9651	1.58e-05	-0.545	0.0001281	-0.6243	0.0001645	intronic	MICU2	22112927	G	A	0.330471	0.3006	0.3631	0.2201	0.4076	0.408
13	21539044	rs11403747	-0.8926	1.82e-05	-0.964	1.61e-05	-0.5518	0.0001193	-0.629	0.0001502	intronic	MICU2	22113183	T	TG	0.332069	0.3056	0.3617	0.2247	0.4076	0.4059
9	10325352	rs78806274	2.057	1.83e-05	2.065	2.49e-05	1.219	0.0006135	1.487	0.0003526	intronic	PTPRD	10325352	A	G	0.106829	0.0466	0.2378	0.1891	0.006	0.0685
13	21546527	rs12584577	-0.8996	1.84e-05	-0.9764	1.56e-05	-0.6219	1.78e-05	-0.6709	5.98e-05	intronic	MICU2	22120666	C	T	0.282149	0.3056	0.3228	0.0688	0.3897	0.407
1	200674277	rs12076877	-1.025	1.87e-05	-0.951	0.0001181	-0.4361	0.002966	-0.6787	0.0001387	ncRNA_intronic	DDX59-AS1	200643405	C	G	0.255192	0.2817	0.2349	0.1142	0.4056	0.2781
20	29046308	rs77639110	1.188	1.87e-05	1.094	0.000158	0.8093	7.6e-05	0.9452	5.05e-05	intergenic	FRG1CP;FRG1DP	NA	NA	NA	NA	NA	NA	NA	NA	NA
14	79001429	rs76832499	1.154	1.9e-05	1.243	7.38e-06	0.6307	0.001759	0.8404	0.0001698	intronic	NRXN3	79467772	C	T	0.0980431	0.1171	0.0965	0.0749	0.1034	0.1053
20	29060896	rs1173817094	1.218	1.93e-05	1.196	5.49e-05	0.646	0.003076	0.8956	0.0002427	intergenic	FRG1CP;FRG1DP	NA	NA	NA	NA	NA	NA	NA	NA	NA
13	21575197	rs12860947	-0.8949	1.97e-05	-0.9713	1.67e-05	-0.6142	2.14e-05	-0.6468	0.0001008	intronic	MICU2	22149336	A	G	0.280551	0.3046	0.3199	0.0598	0.3887	0.4151
19	8895310	rs201022734	1.835	1.97e-05	1.727	0.0001173	1.281	0.0006637	1.261	0.001914	intronic	MUC16	NA	NA	NA	NA	NA	NA	NA	NA	NA
13	21543809	rs56204467	-0.8954	1.98e-05	-0.9683	1.78e-05	-0.5392	0.0001573	-0.6245	0.0001712	intronic	MICU2	NA	NA	NA	NA	NA	NA	NA	NA	NA
13	21549889	rs12877509	-0.8843	1.98e-05	-0.9533	1.82e-05	-0.5572	7.98e-05	-0.6368	0.0001091	intronic	MICU2	22124028	G	C	0.35004	0.3056	0.3545	0.3094	0.3897	0.407
8	40753031	rs12676234	1.209	1.99e-05	1.233	2.44e-05	0.6088	0.003637	0.5776	0.01298	intronic	ZMAT4	40610550	C	T	0.217252	0.38	0.2565	0.1906	0.0676	0.2117
13	21489030	rs9550739	0.8616	1.99e-05	0.9304	1.62e-05	0.4829	0.0006809	0.6265	0.0001533	intergenic	ZDHHC20;MICU2	22063169	A	G	0.499201	0.6627	0.5346	0.379	0.4374	0.5317
13	21470018	rs1199895	-0.9231	2.03e-05	-1.155	2.21e-06	-0.6435	1.01e-05	-0.739	2.05e-05	intergenic	ZDHHC20;MICU2	22044157	T	G	0.670327	0.7411	0.6599	0.739	0.5994	0.5849
13	21531995	rs2798264	-0.8849	2.04e-05	-0.9579	1.75e-05	-0.5409	0.0001469	-0.646	0.0001023	intronic	MICU2	22106134	A	T	0.327077	0.3016	0.3458	0.2163	0.4026	0.4121
17	26722844	rs1324240969	-2.154	2.04e-05	-2.182	2.42e-05	-1.262	0.005985	-1.703	0.0003448	intergenic	ONE;LOC1053717	NA	NA	NA	NA	NA	NA	NA	NA	NA

7	61065596	rs1454766342	1.217	2.07e-05	1.349	5.68e-06	0.8807	4.79e-05	0.8898	0.0002933	intergenic	NONE;NONE	NA	NA	NA	NA	NA	NA	NA	NA	NA
7	100950926	rs75133091	1.599	2.1e-05	1.969	1.68e-05	0.8026	0.0001609	0.8526	0.0007522	intronic	MUC3A	NA	NA	NA	NA	NA	NA	NA	NA	NA
13	21545840	rs71202426	-0.8907	2.12e-05	-0.9669	1.8e-05	-0.6123	2.28e-05	-0.6612	7.3e-05	intronic	MICU2	22119979	AACT	A	0.28155	0.3046	0.3228	0.0688	0.3887	0.4059
13	21550782	rs66508249	-0.8907	2.12e-05	-0.9669	1.8e-05	-0.6123	2.28e-05	-0.6612	7.3e-05	intronic	MICU2	22124921	TACA	T	0.281949	0.3056	0.3228	0.0688	0.3887	0.407
8	40745916	rs58574561	1.21	2.14e-05	1.246	2.25e-05	0.6148	0.00311	0.5585	0.0166	intronic	ZMAT4	40603435	A	C	0.226438	0.381	0.2579	0.2231	0.0686	0.2117
13	21518822	rs4770170	-0.8959	2.14e-05	-0.9965	1.24e-05	-0.6103	2.84e-05	-0.6687	7.34e-05	intronic	MICU2	22092961	T	C	0.293331	0.3046	0.3256	0.1051	0.3926	0.411
13	21577986	rs150371490	-0.8908	2.15e-05	-0.9671	1.82e-05	-0.6048	2.81e-05	-0.6384	0.0001228	intronic	MICU2	22152125	AAAT	A	0.278754	0.3036	0.3184	0.0613	0.3887	0.4059
16	33042173	rs796322145	-1.212	2.18e-05	-1.18	8.39e-05	-0.6058	0.001603	-0.756	0.0006959	intergenic	LC6A10P;TP53TG	NA	NA	NA	NA	NA	NA	NA	NA	NA
16	33042175	rs796931543	-1.212	2.18e-05	-1.18	8.39e-05	-0.6058	0.001603	-0.756	0.0006959	intergenic	LC6A10P;TP53TG	NA	NA	NA	NA	NA	NA	NA	NA	NA
16	21542735	rs1183557141	1.411	2.2e-05	1.288	0.000202	0.8924	0.0005417	1.061	0.000234	intergenic	'A5P2;LOC10192	NA	NA	NA	NA	NA	NA	NA	NA	NA
15	82833313	rs11259953	1.195	2.25e-05	1.206	3.74e-05	0.6807	0.001652	0.6975	0.004333	exonic	WHAMM	83502065	A	C	0.135383	0.1944	0.1916	0.1256	0.1203	0.0634
15	82833314	rs11259954	1.195	2.25e-05	1.206	3.74e-05	0.6807	0.001652	0.6975	0.004333	exonic	WHAMM	83502066	C	G	0.134984	0.1935	0.1902	0.1256	0.1203	0.0634
13	21577737	rs61952264	0.8577	2.3e-05	0.9514	1.25e-05	0.4606	0.001124	0.5674	0.0005442	intronic	MICU2	22151876	A	G	0.457069	0.6964	0.5144	0.2012	0.4414	0.5317
7	142767845	rs1212860291	-1.405	2.32e-05	-1.494	3.21e-05	-0.6181	0.002451	-0.8649	0.0004924	intronic	TCAF2	NA	NA	NA	NA	NA	NA	NA	NA	NA
7	123571518	rs12706538	1.214	2.33e-05	1.143	0.0001887	0.6509	0.002898	0.8704	0.000276	intronic	NDUFA5	123211572	T	C	0.134585	0.0694	0.0937	0.1641	0.1421	0.183
3	36108755	rs10652766	0.9349	2.35e-05	1.048	7.62e-06	0.4261	0.01193	0.5932	0.001745	intergenic	ARPP21;STAC	36150247	A	AATT	0.158746	0.0734	0.2032	0.1097	0.2753	0.1616
11	125288088	rs61917767	3.033	2.35e-05	2.937	9.21e-05	1.56	0.003827	2.045	0.0004468	intronic	PKNOX2	125157984	G	A	0.0367412	0.0923	0.0043	0.0015	0.007	0.0808
4	154148464	rs1243908229	-1.253	2.47e-05	-1.265	5.49e-05	-0.968	1.73e-06	-1.057	8.57e-06	intergenic	SFRP2;DCHS2	NA	NA	NA	NA	NA	NA	NA	NA	NA
8	40754910	rs4631453	1.126	2.49e-05	1.166	2.37e-05	0.5521	0.004445	0.5455	0.0126	intronic	ZMAT4	40612429	G	C	0.729633	0.622	0.7277	0.615	0.9314	0.7894
6	29953458	rs9260532	0.8989	2.51e-05	0.9125	3.07e-05	0.6058	0.0002235	0.6834	0.0001424	intergenic	HLA-A;HCG9	29921235	C	T	0.232428	0.2867	0.2277	0.2383	0.164	0.2423
9	10323957	rs7851619	0.9202	2.52e-05	0.8924	6.85e-05	0.569	0.0004437	0.6686	0.0002414	intronic	PTPRD	10323957	G	C	0.318291	0.2004	0.3948	0.5363	0.1759	0.2372
13	21554484	rs1200047	-0.8784	2.55e-05	-0.9495	2.29e-05	-0.537	0.0001526	-0.6206	0.0001761	intronic	MICU2	22128623	C	T	0.32528	0.3056	0.3473	0.2194	0.3887	0.408
13	21465961	rs7339080	-0.9111	2.56e-05	-1.008	1.69e-05	-0.6349	1.15e-05	-0.6881	5.41e-05	intergenic	ZDHHC20;MICU2	22040100	G	A	0.327077	0.255	0.3415	0.2557	0.3976	0.4151
13	21492472	rs11148128	-0.8855	2.57e-05	-1.032	8.46e-06	-0.6187	2.1e-05	-0.6753	6.09e-05	downstream	MICU2	22066611	A	T	0.308107	0.3373	0.3271	0.1362	0.3926	0.41
13	21564552	rs4770174	-0.8787	2.59e-05	-0.9534	2.2e-05	-0.595	3.78e-05	-0.6264	0.0001529	intronic	MICU2	22138691	T	C	0.282548	0.3056	0.3213	0.0703	0.3887	0.409
13	21594354	rs7335880	-0.8746	2.59e-05	-0.944	2.34e-05	-0.5386	0.000149	-0.6105	0.0002241	intronic	MICU2	22168493	G	A	0.320887	0.3036	0.3415	0.208	0.3877	0.408
1	24978666	rs6600248	-0.9811	2.63e-05	-0.989	4.44e-05	-0.5075	0.0007224	-0.7072	0.0001441	intergenic	RUNX3;MIR4425	25305157	A	G	0.60004	0.6726	0.7262	0.5257	0.5467	0.591
20	29731114	rs1255950384	-1.159	2.63e-05	-1.15	6.36e-05	-0.7908	6.61e-05	-0.9504	2.96e-05	intergenic	AM242B;MIR447	NA	NA	NA	NA	NA	NA	NA	NA	NA
15	82862073	rs201965193	1.227	2.64e-05	1.219	5.69e-05	0.7242	0.001096	0.7926	0.00172	intronic	HOMER2	NA	NA	NA	NA	NA	NA	NA	NA	NA
3	85190237	rs72909180	-1.768	2.65e-05	-2.08	4.27e-05	-0.6466	0.001501	-1.421	3.48e-06	intronic	CADM2	85239387	A	G	0.277157	0.1875	0.1066	0.6755	0.1024	0.1319
15	82862194	rs56686717	1.281	2.66e-05	1.268	6.15e-05	0.7483	0.001061	0.7861	0.002748	intronic	HOMER2	83530946	T	C	0.127995	0.2103	0.17	0.149	0.0646	0.0501
8	19799137	rs7008703	0.8729	2.67e-05	0.9032	5.49e-05	0.4622	0.0009147	0.4928	0.002261	intergenic	GALNACT1;INTS	19656648	C	T	0.521166	0.6111	0.5389	0.4977	0.4006	0.5716
1	161996330	rs73021247	1.855	2.69e-05	1.976	1.53e-05	1.163	0.000366	1.525	5.44e-05	intronic	OLFML2B	161966120	G	A	0.0848642	0.119	0.0663	0.1437	0.0219	0.0481
13	21554264	rs1200049	-0.8715	2.71e-05	-0.9405	2.44e-05	-0.5248	0.0002108	-0.5979	0.0002807	intronic	MICU2	22128403	T	C	0.325479	0.3046	0.3473	0.2209	0.3887	0.408
13	21554356	rs1200048	-0.8715	2.71e-05	-0.9405	2.44e-05	-0.5248	0.0002108	-0.5979	0.0002807	intronic	MICU2	22128495	T	C	0.325479	0.3046	0.3473	0.2209	0.3887	0.408
13	21557021	rs1200046	-0.8715	2.71e-05	-0.9405	2.44e-05	-0.5334	0.0001727	-0.6281	0.0001493	intronic	MICU2	22131160	C	A	0.32508	0.3046	0.3473	0.2201	0.3887	0.407
13	21564245	rs2093804	-0.8715	2.71e-05	-0.9405	2.44e-05	-0.5297	0.0001868	-0.6071	0.000235	intronic	MICU2	22138384	T	G	0.32528	0.3046	0.3473	0.2209	0.3887	0.407
13	21565152	rs17679490	-0.8715	2.71e-05	-0.9405	2.44e-05	-0.536	0.0001554	-0.6187	0.0001793	intronic	MICU2	22139291	C	T	0.327077	0.3046	0.3473	0.2269	0.3887	0.408
13	21568961	rs2040059	-0.8715	2.71e-05	-0.9405	2.44e-05	-0.5248	0.0002108	-0.5979	0.0002807	intronic	MICU2	22143100	C	G	0.326078	0.3046	0.3487	0.2216	0.3887	0.409
13	21482099	rs1744916	-0.8462	2.72e-05	-0.897	2.76e-05	-0.501	0.000401	-0.6067	0.0002181	intergenic	ZDHHC20;MICU2	22056238	C	G	0.466054	0.3016	0.4553	0.5371	0.5437	0.4673
1	24977972	rs2370233	-0.9619	2.74e-05	-0.9686	4.75e-05	-0.5248	0.0004357	-0.7632	3.95e-05	intergenic	RUNX3;MIR4425	25304463	G	C	0.622604	0.6756	0.7262	0.5499	0.5427	0.6748
1	24977973	rs2370234	-0.9619	2.74e-05	-0.9686	4.75e-05	-0.5248	0.0004357	-0.7632	3.95e-05	intergenic	RUNX3;MIR4425	25304464	C	T	0.623602	0.6776	0.7277	0.5507	0.5447	0.6738
1	150958450	rs114908993	2.102	2.74e-05	2.096	4.46e-05	1.416	0.002068	1.62	0.0006792	intronic	SETDB1	150930926	C	T	0.0119808	0	0.0231	0.0008	0.0358	0.0072
13	21571802	rs12429479	-0.8715	2.76e-05	-0.9406	2.49e-05	-0.5296	0.0001878	-0.5969	0.0002939	intronic	MICU2	22145941	C	T	0.321885	0.3036	0.3444	0.2095	0.3887	0.408
13	21571866	rs12430902	-0.8715	2.76e-05	-0.9406	2.49e-05	-0.5296	0.0001878	-0.5969	0.0002939	intronic	MICU2	22146005	T	C	0.322684	0.3036	0.3444	0.2126	0.3887	0.408
13	21573374	rs2096263	-0.8715	2.76e-05	-0.9406	2.49e-05	-0.5296	0.0001878	-0.5969	0.0002939	intronic	MICU2	22147513	T	C	0.322484	0.3036	0.3429	0.2103	0.3887	0.411
13	21574147	rs2761916	-0.8715	2.76e-05	-0.9406	2.49e-05	-0.5296	0.0001878	-0.5969	0.0002939	intronic	MICU2	22148286	A	C	0.323482	0.3046	0.3429	0.2103	0.3887	0.4151
13	21589887	rs67982666	-0.8715	2.76e-05	-0.9406	2.49e-05	-0.5296	0.0001878	-0.5969	0.0002939	intronic	MICU2	22164026	GA	G	0.321486	0.3036	0.3444	0.2095	0.3887	0.4059
13	21595184	rs35067197	-0.8715	2.76e-05	-0.9406	2.49e-05	-0.5296	0.0001878	-0.5969	0.0002939	intronic	MICU2	22169323	C	T	0.321086	0.3036	0.3415	0.2088	0.3877	0.408
2	91869221	rs4059845	1.454	2.77e-05	1.402	9.83e-05	1.011	2e-06	1.089	2.01e-05	intergenic	GGT8P;ACTR3BP	NA	NA	NA	NA	NA	NA	NA	NA	NA
13	21559590	rs2476638	-0.869	2.79e-05	-0.9377	2.52e-05	-0.5237	0.0002166	-0.5962	0.0002886	intronic	MICU2	22133729	T	C	0.325479	0.3046	0.3473	0.2209	0.3887	0.408
17	21645618	rs62049740	-1.156	2.82e-05	-1.163	5.7e-05	-0.9457	1.26e-06	-1.08	2.22e-06	intergenic	INC02693;KCNJ1	NA	NA	NA	NA	NA	NA	NA	NA	NA
1	24978623	rs6600247	-0.9759	2.84e-05	-0.983	4.82e-05	-0.4996	0.0008636	-0.7011	0.0001622	intergenic	RUNX3;MIR4425	25305114	T	C	0.605631	0.6716	0.7277	0.5484	0.5457	0.59
1	161997370	rs10918414	1.952	2.84e-05	2.079	1.67e-05	1.193	0.0008666	1.386	0.0004471	intronic	OLFML2B	161967160	G	A	0.0541134	0.123	0.0576	0.0303	0.0209	0.047
13	21571469	rs1963168	0.8441	2.84e-05	0.9379	1.51e-05	0.4461	0.001528	0.5546	0.0006875	intronic	MICU2	22145608	G	A	0.457268	0.6964	0.5144	0.202	0.4414	0.5317
13	21573272	rs66941223	0.8441	2.84e-05	0.9379	1.51e-05	0.4461	0.001528	0.5546	0.0006875	intronic	MICU2	22147411	ATTTTTTTGTTTT	A	0.45647	0.6954	0.5144	0.2012	0.4414	0.5297
8	40684613	rs16889855	1.079	2.86e-05	1.094	3.68e-05	0.6639	0.0006822	0.6973	0.001279	intronic	ZMAT4	40542132	C	T	0.236222	0.3938	0.268	0.2194	0.0716	0.2434
13	21557758	rs2798269	-0.8682	2.86e-05	-0.9371	2.57e-05	-0.5328	0.00017	-0.6153	0.0001931	intronic	MICU2	22131897	C	T	0.32528	0.3046	0.3473	0.2201	0.3887	0.408
13	21562626	rs2798271	-0.8682	2.86e-05	-0.9371	2.57e-05	-0.5328	0.00017	-0.6153	0.0001931	intronic	MICU2	22136765	C	T	0.325479	0.3056	0.3473	0.2194	0.3887	0.409
13	21562627	rs2798272	-0.8682	2.86e-05	-0.9371	2.57e-05	-0.5328	0.00017	-0.6153	0.0001931	intronic	MICU2	22136766	C	G	0.325479	0.3056	0.3473	0.2194	0.3887	0.409
13	21562991	rs2761914	-0.8682	2.86e-05	-0.9371	2.57e-05	-0.5217	0.0002301	-0.5946	0.0003015	intronic	MICU2	22137130	T	A	0.32528	0.3046	0.3473	0.2201	0.3887	0.408
13	21563604	rs11148158	-0.8682	2.86e-05	-0.9371	2.57e-05	-0.5217	0.0002301	-0.5946	0.0003015	intronic	MICU2	22137743	C	T	0.32528	0.3046	0.3473	0.2201	0.3887	0.408
13	21597650	rs7321318	-0.8774	2.87e-05	-0.9522	2.47e-05	-0.601	3.18e-05	-0.6308	0.0001471	intronic	MICU2	22171789	G	A	0.277955	0.3026	0.3184	0.0598	0.3877	0.4059
13	21557551	rs2798268	-0.8665	2.9e-05	-0.9354	2.6e-05	-0.5443	0.0001252	-0.6347	0.0001257	intronic	MICU2	22131690	A	G	0.325479	0.3056	0.3473	0.2201	0.3887	0.408
9	10303042	rs628731	0.8945	2.91e-05	0.8871	6.2e-05	0.6253	7.59e-05	0.7037	7.12e-05	intronic	PTPRD	10303042	A	G	0.365016	0.253	0.3963	0.5749	0.1899	0.3548
8	19796163	rs58556504	0.8648	2.92e-05	0.9233	3.13e-05	0.4701	0.0006388	0.5139	0.001332	intergenic	GALNACT1;INTS	19653674	A	G	0.510184	0.6042	0.5447	0.4834	0.4066	0.5317
1	117639417	rs55969585	1.671	2.99e-05	1.517	0.0002954	1.354	5.18e-05	1.341	0.0002094	intergenic	TENT5C;GDAP2	118182039	G	T	0.0467252	0.0258	0.0403	0.0386	0.0805	0.0491
13	21500146	rs72183497	0.8465	3e-05	0.9353	1.74e-05	0.4708	0.0009726	0.5975	0.0003001	intronic	MICU2	22074285	AGCATCAT	A	0.440695	0.6627	0.4986	0.1959	0.4155	0.5276
7	60921552	rs1460221574	-1.325	3.02e-05	-1.366	5.98e-05	-0.8518	2.5e-05	-1.041	1.9e-05	intergenic	NONE;NONE	NA	NA	NA	NA	NA	NA	NA	NA	NA
9	10286018	rs16925703	1.88	3.02e-05	1.888	3.65e-05	1.091	0.001227	1.348	0.0005319	intronic	PTPRD	10286018	G	A	0.150359	0.0407	0.2435	0.3366	0.006	0.0941
9	10286040	rs76139064	1.88	3.02e-05	1.888	3.65e-05	1.091	0.001227	1.348	0.0005319	intronic	PTPRD	10286040	A	T	0.150359	0.0407	0.2435	0.3366	0.006	0.0941
13	21482528	rs9509757	0.8422	3.05e-05	0.9123	2.35e-05	0.4918	0.0005358	0.6174	0.000193	intergenic	ZDHHC20;MICU2	22056667	C	G	0.485024	0.6984	0.5303	0.2874	0.4543	0.5317
13	21507697	rs9550741	1	3.05e-05	1.241	2.5e-06	0.554	0.001292	0.8431	1.91e-05	intronic	MICU2	22081836	G	A	0.355831	0.6944	0.3559	0.1838	0.167	0.4335
13	21515927	rs1044090	-0.873	3.05e-05	-1.041	6.81e-06	-0.5749	6.84e-05	-0.6366	0.0001427	intronic	MICU2	22090066	A	C	0.29373	0.3065	0.33	0.1051	0.3926	0.408
14	78993068	rs7150552	1.12	3.06e-05	1.208	1.19e-05	0.607	0.002121	0.8114	0.0002157	intronic	NRXN3	79459411	A	G	0.108626	0.1171	0.0994	0.1014	0.1054	0.1196
1	24978681	rs6600250	-0.9751	3.08e-05	-0.9834	5.11e-05	-0.4979	0.0009379	-0.7008	0.0001707	intergenic	RUNX3;MIR4425	25305172	C	T	0.599641	0.6716	0.7262	0.525	0.5467	0.591
6	151615079	rs6914432	-1.291	3.09e-05	-1.406	3.29e-05	-0.9708	1.16e-06	-1.109	5.33e-06	intronic	CCDC170	151936214	C	G	0.170927	0.0218	0.1542	0.202	0.2147	0.2495
17	10617383	rs8079868	1.022	3.09e-05	1.058	4.04e-05	0.3518	0.04563	0.5315	0.00787	ncRNA_intronic	MYHAS	10520700	T	C	0.26278	0.2619	0.2305	0.4917	0.1252	0.1186
13	21597019	rs4769169	-0.869	3.14e-05	-0.9942	1.35e-05	-0.5789	6.16e-05	-0.6031	0.0002612	intronic	MICU2	22171158	A	G	0.301917	0.3036	0.3343	0.1135	0.4175	0.4131
8	19800506	rs4577973	0.8675	3.16e-05	0.9285	3.34e-05	0.4836	0.0005041	0.5123	0.001525	intergenic	GALNACT1;INTS	19658017	T	C	0.521965	0.6111	0.5432	0.4985	0.4006	0.5716
11	69985545	rs61885141	1.176	3.19e-05	1.037	0.0004781	0.5745	0.009052	0.8841	0.000273	upstream	ANO1	69831651	C	G	0.0692891	0.1319	0.0735	0.0083	0.1113	0.0409
13	21562441	rs34097809	-0.8645	3.19e-05	-0.9301	3.02e-05	-0.5426	0.0001304	-0.6169	0.0001856	intronic	MICU2	22136580	AT	A	0.327476	0.3046	0.3501	0.2269	0.3887	0.408
10	74474679	rs4745747	1.007	3.21e-05	0.9322	0.0001727	0.4844	0.002521	0.6198	0.0009208	intronic	ADK	76234437	G	T	0.517772	0.372	0.6297	0.3707	0.7575	0.5409
9	10318490	rs587791	0.9295	3.22e-05	0.9041	8.04e-05	0.5407	0.001247	0.707	0.0001649	intronic	PTPRD	10318490	G	T	0.313698	0.1677	0.3905	0.5356	0.161	0.2669
1	161997025	rs12045457	1.824	3.23e-05	1.976	1.53e-05	1.156	0.0003909	1.517	5.88e-05	intronic	OLFML2B	161966815	C	T	0.0854633	0.119	0.0663	0.1467	0.0219	0.047
3	85214628	rs7630177	-1.749	3.23e-05	-2.058	5.03e-05	-0.6509	0.001537	-1.402	4.6e-06	intronic	CADM2	85263778	A	C	0.252196	0.1885	0.098	0.5825	0.1024	0.135
4	116176401	rs11939549	1.188	3.23e-05	1.283	1.24e-05	0.7583	0.0004651	0.9442	0.0001373	intergenic	NDST4;MIR1973	117097557	T	C	0.252396	0.0913	0.1902	0.6346	0.1074	0.0951
3	42120424	rs139585936	1.373	3.24e-05	1.247	0.0002863	0.8016	0.001817	0.8461	0.003108	intronic	TRAK1	42161916	GGGCC	G	0.0585064	0.0516	0.0504	0.0242	0.1064	0.0685
13	21522472	rs1359190	-0.8682	3.25e-05	-1.03	7.91e-06	-0.6061	3.26e-05	-0.6621	8.28e-05	intronic	MICU2	22096611	T	G	0.29992	0.3056	0.3401	0.1059	0.4125	0.4121
7	79191273	rs10485934	1.705	3.28e-05	1.699	5.26e-05	1.001	0.0004675	1.033	0.002355	intronic	MAGI2	78820589	C	T	0.127596	0.1508	0.0879	0.3064	0.0119	0.0092
10	74096149	rs59287851	0.9804	3.28e-05	0.9457	9.08e-05	0.5583	0.0005035	0.6422	0.000541	intronic	VCL	NA	NA	NA	NA	NA	NA	NA	NA	NA
15	82822904	rs58528072	1.115	3.28e-05	1.117	5.9e-05	0.5455	0.007618	0.6206	0.00772	intronic	WHAMM	83491656	TTACA	T	0.161542	0.1875	0.1974	0.2239	0.1243	0.0634
18	54537838	rs1439531635	-1.169	3.3e-05	-1.13	0.0001322	-0.6269	0.001323	-0.7442	0.0009887	intergenic	C18orf54;DYNAP	NA	NA	NA	NA	NA	NA	NA	NA	NA
3	85305864	rs10440085	-1.647	3.32e-05	-1.838	5.18e-05	-0.5731	0.003716	-1.21	2.13e-05	intronic	CADM2	85355014	G	A	0.251997	0.1815	0.1009	0.5817	0.1004	0.1421
13	21536326	rs34967503	-0.8535	3.36e-05	-0.99	1.18e-05	-0.5992	3.07e-05	-0.641	0.0001058	intronic	MICU2	22110465	A	AT	0.281749	0.3016	0.3256	0.0703	0.3887	0.4059
10	74503503	rs3862535	1.007	3.38e-05	0.9528	0.0001337	0.5272	0.001259	0.644	0.0006831	intronic	ADK	76263261	G	A	0.617412	0.3631	0.6671	0.7504	0.7624	0.5153
17	26722843	rs1278369239	-2.008	3.38e-05	-2.035	4.13e-05	-1.14	0.009335	-1.567	0.0005778	intergenic	ONE;LOC1053717	NA	NA	NA	NA	NA	NA	NA	NA	NA
3	85311506	rs1985716	-1.626	3.39e-05	-1.801	5.92e-05	-0.5787	0.003477	-1.221	1.7e-05	intronic	CADM2	85360656	A	G	0.22524	0.1825	0.0908	0.4887	0.0994	0.138
13	21560752	rs11618032	-0.8601	3.43e-05	-0.9282	3.13e-05	-0.5372	0.000153	-0.6137	0.0002008	intronic	MICU2	22134891	T	C	0.32488	0.3046	0.3473	0.2186	0.3887	0.408

13	21473209	rs9552432	0.9706	3.45e-05	1.153	6.32e-06	0.5459	0.0009965	0.8308	1.57e-05	intergenic	ZDHHC20;MICU2	22047348	A	G	0.411941	0.7391	0.3948	0.2965	0.2087	0.4519
13	21549680	rs7990030	-0.8622	3.46e-05	-0.932	3.03e-05	-0.5307	0.0001841	-0.6112	0.0002212	intronic	MICU2	22123819	T	C	0.326478	0.3056	0.3473	0.2239	0.3887	0.408
9	10299478	rs629243	0.8933	3.47e-05	0.8794	8.29e-05	0.5992	0.0001479	0.6984	8.8e-05	intronic	PTPRD	10299478	T	A	0.347843	0.254	0.3876	0.5212	0.1789	0.3558
15	82946522	rs1002881	1.113	3.49e-05	1.142	3.99e-05	0.7452	0.0003101	0.783	0.0007964	intronic	HOMER2	83615274	G	A	0.115815	0.1716	0.183	0.0983	0.0885	0.0624
6	15350224	rs11753952	1.455	3.52e-05	1.416	0.0001037	1.023	0.0004101	1.329	2.98e-05	intronic	JARID2	15350455	G	A	0.0890575	0.0615	0.0533	0.1392	0.0447	0.1207
7	79181396	rs115916112	1.728	3.53e-05	1.712	6.41e-05	1.033	0.0004465	0.9551	0.005052	intronic	MAGI2	78810712	T	G	0.0834665	0.1518	0.0893	0.1377	0.0119	0.0092
13	21566243	rs9552453	0.9793	3.54e-05	1.182	4.71e-06	0.5089	0.002543	0.786	5.48e-05	intronic	MICU2	22140382	A	G	0.366613	0.6944	0.3703	0.1899	0.1879	0.4489
6	153894824	rs62434386	-1.642	3.56e-05	-1.807	7.85e-06	-1.139	0.0006853	-1.193	0.0009993	intergenic	RGS17;OPRM1	NA	NA	NA	NA	NA	NA	NA	NA	NA
13	21523491	rs12428749	-0.8582	3.56e-05	-0.9965	1.24e-05	-0.5974	3.7e-05	-0.6374	0.000134	intronic	MICU2	22097630	C	T	0.28774	0.3046	0.3242	0.0862	0.3926	0.409
22	16454781	rs796806674	1.158	3.59e-05	1.376	6.33e-06	0.7501	9.24e-05	0.9803	1.5e-05	intergenic	DUXAP8;CCT8L2	NA	NA	NA	NA	NA	NA	NA	NA	NA
13	21560903	rs2761913	-0.8578	3.6e-05	-0.9253	3.31e-05	-0.5322	0.000174	-0.611	0.0002139	intronic	MICU2	22135042	C	T	0.325479	0.3046	0.3473	0.2186	0.3887	0.411
9	10323737	rs12000959	0.9199	3.62e-05	0.8888	0.0001038	0.5736	0.0004667	0.6757	0.0002709	intronic	PTPRD	10323737	C	T	0.327476	0.2004	0.3977	0.5461	0.1769	0.2679
4	116227844	rs1506741	1.229	3.64e-05	1.318	1.62e-05	0.7943	0.0002554	1.035	5.33e-05	intergenic	NDST4;MIR1973	117149000	G	C	0.227835	0.0903	0.17	0.4554	0.1054	0.229
3	36124533	rs7636488	0.9139	3.65e-05	0.9968	1.8e-05	0.3944	0.01969	0.5577	0.00314	intergenic	ARPP21;STAC	36166025	T	C	0.158147	0.0734	0.2032	0.1074	0.2753	0.1616
9	10284947	rs12001726	1.854	3.66e-05	1.888	3.65e-05	1.081	0.001337	1.336	0.0005912	intronic	PTPRD	10284947	G	T	0.150359	0.0407	0.2435	0.3366	0.006	0.0941
9	10284969	rs12005579	1.854	3.66e-05	1.888	3.65e-05	1.081	0.001337	1.336	0.0005912	intronic	PTPRD	10284969	A	G	0.150359	0.0407	0.2435	0.3366	0.006	0.0941
9	10323575	rs12000957	0.934	3.67e-05	0.9193	7.53e-05	0.5935	0.0003665	0.694	0.0002151	intronic	PTPRD	10323575	G	C	0.324681	0.2004	0.3948	0.5386	0.174	0.2689
9	63880149	rs1440658387	1.179	3.7e-05	1.263	3.03e-05	0.8343	6.72e-05	1.003	2.66e-05	intergenic	FRG1JP;FLJ43315	NA	NA	NA	NA	NA	NA	NA	NA	NA
10	74488630	rs6143984	1.001	3.75e-05	0.9477	0.0001464	0.5272	0.00127	0.6374	0.0007815	intronic	ADK	76248388	A	ATTTTTCTCCTC	0.617212	0.3651	0.6686	0.7481	0.7614	0.5153
10	74548214	rs10740439	1.001	3.75e-05	0.9477	0.0001464	0.5272	0.00127	0.6374	0.0007815	intronic	ADK	76307972	C	T	0.616813	0.3621	0.6671	0.7496	0.7614	0.5153
3	36090214	rs1545481	0.9175	3.77e-05	1.028	1.29e-05	0.3975	0.0195	0.5774	0.002467	intergenic	ARPP21;STAC	36131706	T	C	0.159145	0.0734	0.2061	0.1097	0.2763	0.1605
3	110637310	rs536855860	1.203	3.8e-05	1.307	1.8e-05	0.8889	3.4e-05	1.073	9.01e-06	intergenic	R4445;NECTIN3-A	110356157	G	A	0.024361	0.0109	0.0418	0.0242	0.0229	0.0276
13	21510360	rs2296977	0.8331	3.8e-05	0.9203	2.2e-05	0.4545	0.001442	0.5814	0.0004259	intronic	MICU2	22084499	G	C	0.448882	0.6964	0.4986	0.1982	0.4165	0.5307
8	19801023	rs3866469	0.8499	3.81e-05	0.9089	4.05e-05	0.4746	0.0006073	0.509	0.001507	intergenic	GALNACT1;INTS1	19658534	T	C	0.522364	0.6111	0.5432	0.4985	0.4006	0.5736
20	30356038	rs62198872	-1.117	3.84e-05	-1.199	3.02e-05	-0.9975	4.31e-07	-1.243	7.77e-08	intergenic	INC01597;FRG1B	NA	NA	NA	NA	NA	NA	NA	NA	NA
9	10285341	rs12002159	1.848	3.89e-05	1.882	3.84e-05	1.073	0.001457	1.328	0.0006411	intronic	PTPRD	10285341	G	A	0.150359	0.0407	0.2435	0.3366	0.006	0.0941
9	10285347	rs12006022	1.848	3.89e-05	1.882	3.84e-05	1.073	0.001457	1.328	0.0006411	intronic	PTPRD	10285347	A	T	0.150359	0.0407	0.2435	0.3366	0.006	0.0941
18	14266737	rs200156232	1.486	3.89e-05	1.418	0.0001731	1.039	0.0003141	1.29	4.7e-05	intergenic	KRD20A5P;CYP4F	NA	NA	NA	NA	NA	NA	NA	NA	NA
20	29046315	rs75488419	1.119	3.89e-05	1.053	0.0002032	0.7304	0.0002131	0.8297	0.0002477	intergenic	FRG1CP;FRG1DP	NA	NA	NA	NA	NA	NA	NA	NA	NA
7	62425241	rs1316125038	-1.447	3.9e-05	-1.741	2.04e-05	-0.9028	2.92e-05	-1.111	2.72e-05	intergenic	NONE;ZNF733P	NA	NA	NA	NA	NA	NA	NA	NA	NA
20	29791086	rs1440702293	1.157	3.9e-05	1.235	3.55e-05	0.6223	0.001131	0.7325	0.0009675	intergenic	AM242B;MIR447	NA	NA	NA	NA	NA	NA	NA	NA	NA
3	36125229	rs35344316	0.9104	3.95e-05	0.9937	1.94e-05	0.388	0.02162	0.5519	0.003451	intergenic	ARPP21;STAC	36166721	G	GAT	0.158147	0.0734	0.2032	0.1074	0.2753	0.1616
3	156562622	rs55637018	2.941	3.95e-05	2.867	7.94e-05	2.342	0.0002708	2.629	6.55e-05	intergenic	SSR3;TIPARP-AS1	156280411	G	A	0.00878594	0	0.0187	0.0008	0.0268	0.0031
3	85322801	rs818216	-1.608	3.96e-05	-1.782	6.8e-05	-0.5551	0.004938	-1.158	3.83e-05	intronic	CADM2	85371951	T	A	0.246406	0.1815	0.098	0.5628	0.1004	0.1411
7	51641608	rs10263016	1.304	3.96e-05	1.221	0.0003358	0.6716	0.007547	0.8192	0.005786	intergenic	07986794;POM1	51709304	T	G	0.118411	0	0.0764	0.292	0.0805	0.0746
13	21494086	rs1199938	-0.8051	3.98e-05	-0.8455	4.36e-05	-0.4682	0.0007669	-0.588	0.0002593	intronic	MICU2	22068225	T	C	0.44988	0.3373	0.4409	0.4637	0.5358	0.4652
1	112823300	rs147142092	2.269	3.99e-05	2.084	0.0002758	1.337	0.007501	1.579	0.002496	ncRNA_intronic	LINC01356	113365922	G	A	0.0133786	0	0.0173	0.0008	0.0229	0.0317
12	94563958	rs2128896	-0.9618	4.02e-05	-0.9794	7.22e-05	-0.6719	2.72e-05	-0.8468	1.02e-05	intergenic	MIR5700;TMCC3	94957734	C	T	0.752596	0.9236	0.7392	0.7375	0.663	0.6984
8	40778385	rs7004361	1.104	4.04e-05	1.156	2.96e-05	0.5511	0.006062	0.642	0.004094	intronic	ZMAT4	40635904	G	C	0.248403	0.4395	0.2349	0.2632	0.0686	0.226
17	57321218	rs72830848	1.636	4.04e-05	1.732	2.04e-05	0.4673	0.1552	0.839	0.01989	intronic	MSI2	55398579	C	T	0.0161741	0	0.0159	0	0.0606	0.0092
13	21495681	rs1199942	-0.8063	4.06e-05	-0.8462	4.49e-05	-0.4603	0.001007	-0.5866	0.0002811	intronic	MICU2	22069820	G	A	0.449481	0.3026	0.4553	0.466	0.5557	0.4652
1	29576845	rs28533195	1.148	4.07e-05	1.093	0.0001363	0.5557	0.005393	0.7507	0.001192	intergenic	NC01756;LINC016	30049692	G	A	0.124002	0.002	0.196	0.0817	0.2654	0.1104
8	19795539	rs6586867	0.8508	4.07e-05	0.9085	4.43e-05	0.46	0.0008821	0.4972	0.001936	intergenic	GALNACT1;INTS1	19653050	A	G	0.522764	0.6062	0.5447	0.497	0.4076	0.5746
15	82900670	rs7174216	1.183	4.08e-05	1.181	7.94e-05	0.7286	0.0008792	0.7768	0.001846	intronic	HOMER2	83569422	T	C	0.179113	0.2163	0.1916	0.3116	0.0775	0.0573
9	10285076	rs12005969	1.841	4.1e-05	1.876	4.04e-05	1.065	0.001611	1.319	0.0006913	intronic	PTPRD	10285076	A	C	0.150359	0.0407	0.2435	0.3366	0.006	0.0941
12	98155089	rs78924855	2.26	4.11e-05	2.423	1.09e-05	1.306	0.007654	1.682	0.00101	intergenic	MIR4303;SLC9A7P	98548867	G	A	0.0385383	0.0317	0.0173	0.0658	0.0129	0.0501
20	30179876	rs878937828	-1.339	4.14e-05	-1.371	4.81e-05	-1.333	4.44e-07	-1.477	2.67e-07	intergenic	IR4477A;LINC015	NA	NA	NA	NA	NA	NA	NA	NA	NA
8	40752427	rs4736878	0.9167	4.15e-05	0.9581	3.27e-05	0.4182	0.009207	0.5237	0.003989	intronic	ZMAT4	40609946	C	G	0.65615	0.619	0.6527	0.5915	0.8082	0.6278
13	21601944	rs7330859	0.8164	4.18e-05	0.8822	3.21e-05	0.4891	0.0004682	0.5394	0.0007759	intronic	MICU2	22176083	C	A	0.527356	0.6895	0.5461	0.4554	0.4414	0.5327
4	29740951	rs73215339	2.738	4.19e-05	2.764	3.74e-05	1.994	0.0004351	2.256	0.0002503	intergenic	LINC02472;PCDH	29742573	T	C	0.0299521	0	0.0274	0.0658	0.0378	0.0061
18	15344674	rs77889827	1.282	4.22e-05	1.253	0.0001376	0.8075	5.47e-05	0.7772	0.000834	intergenic	LOC644669;NON	NA	NA	NA	NA	NA	NA	NA	NA	NA
1	24977085	rs10903122	-0.9582	4.25e-05	-0.974	6.79e-05	-0.4919	0.00108	-0.7488	7.58e-05	intergenic	RUNX3;MIR4425	25303576	A	G	0.638978	0.6756	0.7421	0.5923	0.5447	0.6881
4	116211311	rs28437135	1.19	4.25e-05	1.287	1.61e-05	0.7798	0.0003678	0.9922	8.18e-05	intergenic	NDST4;MIR1973	117132467	T	A	0.238818	0.0903	0.1844	0.5893	0.1064	0.093
14	18294828	rs28803325	1.229	4.28e-05	1.309	2.72e-05	0.7711	0.0009782	0.8374	0.00146	intergenic	NONE;OR11H12	NA	NA	NA	NA	NA	NA	NA	NA	NA
3	156538384	rs9985499	2.911	4.3e-05	2.867	7.94e-05	2.333	0.000285	2.618	6.96e-05	UTR3	KCNAB1	156256173	A	G	0.00858626	0	0.0159	0.0008	0.0268	0.0041
3	156561017	rs112231209	2.911	4.3e-05	2.867	7.94e-05	2.321	0.0003156	2.604	8.06e-05	intergenic	SSR3;TIPARP-AS1	156278806	G	A	0.00958466	0	0.0187	0.0008	0.0288	0.0051
10	42264603	rs28482394	1.567	4.34e-05	1.419	0.0002358	0.8046	0.0001713	0.8865	0.0004893	intergenic	NONE;LOC44166	NA	NA	NA	NA	NA	NA	NA	NA	NA
17	26950006	rs73312311	1.177	4.35e-05	1.195	8.73e-05	0.9056	3.59e-06	1.102	2.52e-06	intergenic	ONE;LOC1053717	NA	NA	NA	NA	NA	NA	NA	NA	NA
7	142457914	rs189593833	1.174	4.37e-05	1.286	1.63e-05	0.8752	5.74e-05	1.097	5.64e-06	intronic	TCAF2	NA	NA	NA	NA	NA	NA	NA	NA	NA
1	30348644	rs428978	1.161	4.39e-05	1.165	6.43e-05	0.8223	0.0002251	0.9452	0.0001349	intergenic	LINC01648;MATN	30821491	C	T	0.872204	0.8413	0.8357	0.9085	0.8966	0.8558
10	31789700	rs7921490	1.459	4.4e-05	1.602	9.41e-06	0.841	0.004215	1.013	0.001436	intergenic	ZEB1;ARHGAP12	32078628	T	C	0.033746	0.003	0.0778	0.0265	0.0656	0.0112
1	117640622	rs79003536	1.595	4.41e-05	1.463	0.0003732	1.285	7.92e-05	1.289	0.0002678	intergenic	TENT5C;GDAP2	118183244	A	G	0.047524	0.0258	0.0403	0.0401	0.0805	0.0511
3	85312394	rs6775792	-1.599	4.42e-05	-1.773	7.44e-05	-0.559	0.004573	-1.189	2.7e-05	intronic	CADM2	85361544	G	C	0.244808	0.1815	0.098	0.5567	0.1004	0.1411
15	82818461	rs55994896	1.132	4.43e-05	1.143	7.23e-05	0.6278	0.00323	0.68	0.004809	intronic	WHAMM	83487213	G	T	0.148962	0.1845	0.196	0.1861	0.1213	0.0573
16	33039848	rs138208513	-1.181	4.43e-05	-1.298	1.66e-05	-0.6701	0.001661	-0.8746	0.0003204	intergenic	LC6A10P;TP53TG	33051169	C	T	0.0419329	0.006	0.0274	0.0113	0.1014	0.0695
8	40683990	rs57243936	1.066	4.46e-05	1.078	5.83e-05	0.69	0.0005215	0.7039	0.001376	intronic	ZMAT4	40541509	C	T	0.228634	0.3929	0.2709	0.1891	0.0716	0.2444
1	24963243	rs72657048	-0.9373	4.51e-05	-1.011	3.32e-05	-0.4116	0.004863	-0.6641	0.0002949	intronic	RUNX3	25289734	C	G	0.510982	0.6845	0.7046	0.1868	0.5437	0.5992
3	85284069	rs12486846	-1.602	4.51e-05	-1.778	7.51e-05	-0.5491	0.005602	-1.189	2.9e-05	intronic	CADM2	85333219	G	A	0.244609	0.1825	0.1009	0.5567	0.0974	0.1401
1	200686558	rs7542840	-0.966	4.54e-05	-0.9275	0.0001709	-0.4124	0.005341	-0.6699	0.0001984	ncRNA_intronic	DDX59-AS1	200655686	A	T	0.256989	0.2917	0.2219	0.1483	0.3827	0.2638
20	29821707	rs1175391584	-1.15	4.54e-05	-1.081	0.0002436	-0.9035	2.1e-05	-0.9833	4.38e-05	intergenic	AM242B;MIR447	NA	NA	NA	NA	NA	NA	NA	NA	NA
4	29719638	rs11939705	2.151	4.55e-05	2.159	5.14e-05	1.253	0.001959	1.775	0.000194	intergenic	LINC02472;PCDH	29721260	A	G	0.0656949	0	0.0274	0.2027	0.0368	0.0051
22	30234267	rs9608853	1.933	4.55e-05	1.884	0.0001316	1.138	0.003559	1.418	0.0007978	intergenic	HORMAD2;LIF-AS	30630256	G	A	0.0179712	0	0.0259	0.0061	0.0586	0.0051
7	79189830	rs75588042	1.7	4.59e-05	1.682	8.27e-05	1.044	0.0003396	0.9299	0.006109	intronic	MAGI2	78819146	A	G	0.0848642	0.1528	0.0879	0.143	0.0119	0.0092
11	69697906	rs1192929	0.9265	4.59e-05	1	4.54e-05	0.6358	0.0001112	0.741	8.26e-05	downstream	FGF19	69512674	A	G	0.365216	0.8462	0.2781	0.1611	0.2445	0.3313
13	21549669	rs7983473	0.9742	4.59e-05	1.17	6.86e-06	0.507	0.002901	0.7805	6.66e-05	intronic	MICU2	22123808	G	A	0.356829	0.6935	0.3545	0.1785	0.168	0.4468
6	62892800	rs13205352	-0.9133	4.6e-05	-0.8413	0.0002868	-0.6554	1.33e-05	-0.7656	2.03e-05	intergenic	KHDRBS2;LGSN	63602705	C	T	0.666134	0.7718	0.7233	0.5885	0.5875	0.7025
10	74176853	rs10824095	0.9663	4.6e-05	0.9114	0.0001654	0.5234	0.001268	0.6394	0.0006565	UTR5	ADK	75936611	C	T	0.630192	0.3869	0.6787	0.7852	0.7584	0.5051
10	38729483	rs201771919	1.131	4.61e-05	1.18	4.96e-05	0.6824	0.0004095	0.7937	0.0003994	intergenic	ACTR3BP5;NONE	NA	NA	NA	NA	NA	NA	NA	NA	NA
7	142767847	rs1586021376	-1.33	4.63e-05	-1.403	6.48e-05	-0.6563	0.001375	-0.8133	0.000972	intronic	TCAF2	NA	NA	NA	NA	NA	NA	NA	NA	NA
22	16454789	rs796527088	1.14	4.63e-05	1.355	8.57e-06	0.7458	0.0001019	0.9916	1.27e-05	intergenic	DUXAP8;CCT8L2	NA	NA	NA	NA	NA	NA	NA	NA	NA
1	150578780	rs2737821	1.947	4.64e-05	1.941	7.61e-05	1.338	0.001979	1.467	0.001237	intronic	MCL1	150551256	G	A	0.0113818	0	0.0216	0.0008	0.0348	0.0061
13	21518121	rs9552442	0.9779	4.64e-05	1.177	6.59e-06	0.5101	0.002765	0.7886	5.48e-05	intronic	MICU2	22092260	A	G	0.365016	0.6944	0.3689	0.1861	0.1869	0.4479
15	82854635	rs2306428	1.177	4.64e-05	1.154	0.0001174	0.7236	0.001213	0.7616	0.002441	intronic	HOMER2	83523387	C	T	0.126398	0.2024	0.1758	0.146	0.0736	0.0409
3	85308893	rs6780644	-1.592	4.68e-05	-1.782	6.8e-05	-0.5686	0.003906	-1.195	2.37e-05	intronic	CADM2	85358043	C	T	0.258986	0.1915	0.1081	0.5885	0.1123	0.1411
3	85325291	rs818218	-1.592	4.68e-05	-1.782	6.8e-05	-0.5231	0.007311	-1.149	4.34e-05	intronic	CADM2	85374441	G	A	0.246805	0.1825	0.098	0.5635	0.1004	0.1411
3	85327490	rs818221	-1.592	4.68e-05	-1.782	6.8e-05	-0.552	0.005045	-1.149	4.34e-05	intronic	CADM2	85376640	T	A	0.244209	0.1825	0.0994	0.553	0.1004	0.1411
8	15794597	rs76560807	1.384	4.74e-05	1.391	9.3e-05	0.7755	0.008363	0.8194	0.01077	intergenic	TUSC3;MSR1	15652106	T	C	0.0373403	0.0198	0.0461	0.003	0.0706	0.0613
5	95766787	rs3756708	-0.9169	4.75e-05	-0.9584	5.22e-05	-0.3454	0.0186	-0.5021	0.004178	intronic	RHOBTB3	95102491	C	T	0.240815	0.1895	0.5043	0.0212	0.4394	0.1994
13	21542665	rs2761909	-0.8342	4.75e-05	-0.9582	1.92e-05	-0.5128	0.000289	-0.5826	0.0003728	intronic	MICU2	22116804	T	C	0.332668	0.3046	0.3617	0.2247	0.4085	0.409
13	21574559	rs9509802	0.8105	4.77e-05	0.9419	1.38e-05	0.4433	0.001589	0.543	0.0008329	intronic	MICU2	22148698	A	G	0.455871	0.6954	0.5144	0.2012	0.4414	0.5266
17	21715368	rs1397234999	1.123	4.82e-05	1.131	9.1e-05	0.8727	2.33e-05	0.9869	2.74e-05	intergenic	KCNJ18;UBBP4	NA	NA	NA	NA	NA	NA	NA	NA	NA
3	36112821	rs1449991	0.8806	4.83e-05	0.9494	2.94e-05	0.4099	0.009946	0.5762	0.001457	intergenic	ARPP21;STAC	36154313	A	C	0.28774	0.0794	0.2608	0.531	0.2992	0.181
10	74164847	rs7894143	0.963	4.83e-05	0.9079	0.0001735	0.5214	0.001304	0.6372	0.0006764	intronic	ADK	75924605	G	C	0.629992	0.3859	0.6772	0.7859	0.7584	0.5051
10	74182976	rs4745741	0.963	4.83e-05	0.9079	0.0001735	0.5214	0.001304	0.6372	0.0006764	intronic	ADK	75942734	C	T	0.629792	0.3859	0.6772	0.7852	0.7584	0.5051
14	99808434	rs7151803	1.126	4.84e-05	1.315	4.69e-06	0.7573	0.0002373	0.9656	2.13e-05	intronic	EML1	100274771	G	A	0.212859	0.1498	0.1556	0.4402	0.1272	0.0992
4	29708078	rs115793168	2.508	4.86e-05	2.523	5.13e-05	1.479	0.001604	1.954	0.000284	intergenic	LINC02472;PCDH	29709700	G	T	0.0257588	0	0.0202	0.056	0.0358	0.0051
9	10327162	rs1323495	2.121	4.86e-05	2.096	7.81e-05	1.576	0.0001313	1.672	0.0002946	intronic	PTPRD	10327162	T	C	0.101038	0.0476	0.2363	0.1672	0.006	0.0685
15	82875832	rs17158194	1.185	4.86e-05	1.196	8.48e-05	0.6894	0.001965	0.7401	0.003366	intronic	HOMER2	83544584	T	C	0.134984	0.2153	0.1787	0.1566	0.0746	0.0542
7	55638621	rs10233947	0.8113	4.87e-05	0.8043	0.0001066	0.3791	0.005433	0.4915	0.001791	intergenic	VOPP1;FKBP9P1	55706314	C	T	0.50639	0.3105	0.3444	0.8222	0.5517	0.3497

6	62882194	rs4084782	-0.9205	4.88e-05	-0.826	0.000395	-0.5513	0.0002034	-0.6526	0.0002132	intergenic	KHDRBS2;LGSN	63592099	C	T	0.672524	0.7778	0.7219	0.5976	0.5915	0.7137
12	64985593	rs1822929	-0.8355	4.89e-05	-0.697	0.001037	-0.389	0.004507	-0.4231	0.007488	ncRNA_intronic	LINC02231	65379373	G	C	0.511981	0.4315	0.5807	0.497	0.6173	0.4581
13	21602297	rs35747298	-0.836	4.9e-05	-0.9671	1.82e-05	-0.585	4.55e-05	-0.6089	0.0002164	intronic	MICU2	22176436	C	T	0.279553	0.3105	0.3184	0.0598	0.3867	0.407
6	29955646	rs9260639	0.9758	4.92e-05	0.9768	8.14e-05	0.6512	0.0002392	0.7229	0.0002784	intergenic	HLA-A;HCG9	29923423	C	T	0.208466	0.2431	0.2104	0.2141	0.159	0.2147
4	116192135	rs11724265	1.174	4.97e-05	1.279	1.74e-05	0.8433	0.000103	0.9957	6.27e-05	intergenic	NDST4;MIR1973	117113291	A	C	0.707069	0.9087	0.8026	0.2579	0.8877	0.8528
4	116193889	rs1532909	1.174	4.97e-05	1.279	1.74e-05	0.8433	0.000103	0.9957	6.27e-05	intergenic	NDST4;MIR1973	117115045	A	T	0.707668	0.9107	0.804	0.2579	0.8877	0.8528
4	116171995	rs1827949	1.154	5.01e-05	1.251	1.9e-05	0.7557	0.000405	0.9413	0.000128	intergenic	NDST4;MIR1973	117093151	C	G	0.271965	0.0913	0.2017	0.7012	0.1093	0.0951
13	21542932	rs1539562	-0.8309	5.02e-05	-0.9547	2.02e-05	-0.5097	0.0003148	-0.5794	0.0003997	intronic	MICU2	22117071	A	G	0.332069	0.3046	0.3617	0.2224	0.4085	0.409
1	24977414	rs2063112	-0.9447	5.03e-05	-0.9549	8.04e-05	-0.4724	0.00165	-0.7224	0.0001262	intergenic	RUNX3;MIR4425	25303905	G	A	0.626997	0.6746	0.7392	0.5492	0.5447	0.6881
1	24977643	rs2063110	-0.9447	5.03e-05	-0.9549	8.04e-05	-0.479	0.001409	-0.734	9.58e-05	intergenic	RUNX3;MIR4425	25304134	A	G	0.625599	0.6756	0.7392	0.5499	0.5437	0.68
1	124979997	rs1402049450	-1.136	5.03e-05	-1.106	0.0001719	-0.7875	4.67e-05	-0.908	5.75e-05	intergenic	NONE;NONE	NA	NA	NA	NA	NA	NA	NA	NA	NA
1	124980039	rs1460900320	-1.159	5.03e-05	-1.147	0.0001371	-0.8486	1.32e-05	-0.9641	2.61e-05	intergenic	NONE;NONE	NA	NA	NA	NA	NA	NA	NA	NA	NA
3	42104454	rs113712414	1.327	5.04e-05	1.184	0.0005005	0.7354	0.003066	0.7424	0.008731	intronic	TRAK1	42145946	C	T	0.101637	0.0516	0.0605	0.1982	0.0865	0.0675
15	82820879	rs60686632	1.121	5.04e-05	1.136	7.92e-05	0.6313	0.002936	0.6745	0.005104	intronic	WHAMM	83489631	A	C	0.151957	0.1935	0.196	0.1861	0.1213	0.0634
1	124980055	rs1383193086	-1.177	5.05e-05	-1.207	8.09e-05	-0.9208	2.93e-06	-1.056	6.37e-06	intergenic	NONE;NONE	NA	NA	NA	NA	NA	NA	NA	NA	NA
17	21715381	rs1248958341	1.141	5.06e-05	1.126	0.0001301	0.9183	1.83e-05	1.03	2.17e-05	intergenic	KCNJ18;UBBP4	NA	NA	NA	NA	NA	NA	NA	NA	NA
10	74513537	rs10824194	0.9862	5.07e-05	0.934	0.0001899	0.5045	0.002079	0.6148	0.00124	ncRNA_intronic	LOC102723439	76273295	G	A	0.61222	0.3621	0.6657	0.7511	0.7624	0.4898
19	14489751	rs10422158	-0.8325	5.07e-05	-0.921	2.6e-05	-0.4786	0.0006768	-0.5615	0.0007099	intronic	GIPC1	14600563	T	C	0.539537	0.2619	0.585	0.6619	0.499	0.6697
4	29703325	rs80181466	2.5	5.08e-05	2.523	5.13e-05	1.473	0.001664	1.95	0.0002925	intergenic	INC02472;PCDH	29704947	G	A	0.0239617	0	0.0202	0.0492	0.0358	0.0051
4	29703894	rs74431596	2.5	5.08e-05	2.523	5.13e-05	1.473	0.001664	1.95	0.0002925	intergenic	INC02472;PCDH	29705516	G	A	0.0239617	0	0.0202	0.0492	0.0358	0.0051
3	85269145	rs72919209	-1.586	5.14e-05	-1.761	8.48e-05	-0.5796	0.003645	-1.177	3.19e-05	intronic	CADM2	85318295	T	G	0.245008	0.1835	0.0994	0.5582	0.0974	0.1401
4	29756703	rs7683757	1.987	5.14e-05	1.967	8.69e-05	1.647	8.09e-05	1.587	0.0004951	intergenic	INC02472;PCDH	29758325	C	A	0.0425319	0	0.0331	0.09	0.0567	0.0143
6	143948864	rs2064495	0.8994	5.15e-05	0.8171	0.0005581	0.3831	0.01473	0.5692	0.002025	intronic	PLAGL1	144270001	C	T	0.786542	0.5615	0.8487	0.9644	0.7863	0.7342
13	21537660	rs9316443	0.812	5.15e-05	0.9388	1.63e-05	0.4673	0.0009758	0.5832	0.0003812	intronic	MICU2	22111799	A	G	0.448283	0.6984	0.4971	0.1906	0.4215	0.5317
13	21544163	rs11840168	-0.858	5.17e-05	-0.9602	2.54e-05	-0.478	0.0008324	-0.5723	0.0005276	intronic	MICU2	22118302	A	G	0.333067	0.3046	0.3617	0.2262	0.4085	0.409
9	2030570	rs77582903	1.096	5.18e-05	1.016	0.0002278	0.5554	0.007185	0.7696	0.0007902	intronic	SMARCA2	2030570	G	C	0.150559	0.122	0.2493	0.2474	0.0765	0.0552
3	85333058	rs818226	-1.583	5.2e-05	-1.773	7.44e-05	-0.5365	0.006613	-1.134	5.55e-05	intronic	CADM2	85382208	G	A	0.246605	0.1815	0.098	0.5635	0.1004	0.1411
13	21536337	rs1200039	-0.8284	5.21e-05	-0.9584	1.92e-05	-0.5178	0.0002502	-0.5929	0.0003078	intronic	MICU2	22110476	A	C	0.326478	0.2996	0.3501	0.2277	0.3887	0.407
4	29751467	rs7685135	2.722	5.22e-05	2.733	5.34e-05	1.991	0.0004675	2.247	0.0002915	intergenic	INC02472;PCDH	29753089	A	G	0.0277556	0	0.0259	0.0582	0.0378	0.0061
20	29868538	rs1433249736	1.196	5.22e-05	1.189	0.0001414	0.9819	9.82e-07	0.9774	3.12e-05	intergenic	M242B;MIR447	NA	NA	NA	NA	NA	NA	NA	NA	NA
4	29750060	rs7677718	2.722	5.24e-05	2.733	5.38e-05	1.982	0.0004924	2.234	0.0003133	intergenic	INC02472;PCDH	29751682	A	G	0.0277556	0	0.0259	0.0582	0.0378	0.0061
4	29755967	rs147069769	2.722	5.24e-05	2.733	5.38e-05	1.982	0.0004924	2.234	0.0003133	intergenic	INC02472;PCDH	29757589	T	C	0.0275559	0	0.0259	0.0575	0.0378	0.0061
4	29707495	rs28758230	2.135	5.25e-05	2.147	5.86e-05	1.202	0.002507	1.744	0.0002273	intergenic	INC02472;PCDH	29709117	G	A	0.0686901	0	0.0288	0.2133	0.0368	0.0051
1	5673324	rs72863359	1.144	5.27e-05	1.193	6.29e-05	0.7778	6.44e-05	0.8928	7.84e-05	intergenic	AJAP1;MIR4689	5733384	C	T	0.00299521	0.001	0.0014	0.0098	0	0
4	116212385	rs10028392	1.171	5.31e-05	1.287	1.61e-05	0.7703	0.0004276	0.976	0.0001042	intergenic	NDST4;MIR1973	117133541	A	G	0.238818	0.0903	0.1844	0.5893	0.1064	0.093
3	85287409	rs7637876	-1.586	5.32e-05	-1.778	7.51e-05	-0.5446	0.006008	-1.181	3.27e-05	intronic	CADM2	85336559	G	A	0.245208	0.1845	0.1023	0.5552	0.0994	0.1401
3	85306334	rs994237	-1.586	5.32e-05	-1.778	7.51e-05	-0.5734	0.004292	-1.181	3.27e-05	intronic	CADM2	85355484	C	T	0.23123	0.1815	0.0951	0.5098	0.0994	0.138
3	85316164	rs59880141	-1.589	5.37e-05	-1.765	8.7e-05	-0.5307	0.007491	-1.176	3.61e-05	intronic	CADM2	85365314	A	C	0.225639	0.1825	0.0908	0.4902	0.0994	0.138
4	29749758	rs10517181	2.953	5.37e-05	2.964	5.24e-05	2.264	0.0003849	2.485	0.0002773	intergenic	INC02472;PCDH	29751380	C	G	0.0219649	0	0.0216	0.0386	0.0378	0.0061
13	21501952	rs1199949	-0.7858	5.38e-05	-0.8239	5.96e-05	-0.4384	0.001536	-0.548	0.0005961	intronic	MICU2	22076091	T	C	0.450679	0.3373	0.4409	0.4652	0.5358	0.4673
20	25904756	rs62211122	1.126	5.4e-05	1.2	4.43e-05	0.7567	0.0002318	0.9412	5.98e-05	intergenic	01926935;LINC0	NA	NA	NA	NA	NA	NA	NA	NA	NA
3	85326303	rs818220	-1.58	5.41e-05	-1.755	8.77e-05	-0.5158	0.008483	-1.171	4.35e-05	intronic	CADM2	85375453	T	A	0.246805	0.1825	0.098	0.5635	0.1004	0.1411
20	29791089	rs1242772574	1.13	5.42e-05	1.235	3.55e-05	0.6138	0.001312	0.7189	0.001197	intergenic	M242B;MIR447	NA	NA	NA	NA	NA	NA	NA	NA	NA
4	29745046	rs73215341	2.107	5.44e-05	2.164	4.35e-05	1.779	5.51e-05	1.707	0.0004055	intergenic	INC02472;PCDH	29746668	A	T	0.0427316	0	0.0331	0.0908	0.0567	0.0143
1	200687859	rs7524025	-0.9519	5.47e-05	-0.9139	0.0002027	-0.4062	0.005952	-0.663	0.0002247	ncRNA_intronic	DDX59-AS1	200656987	G	A	0.257388	0.2917	0.2219	0.1483	0.3817	0.2669
10	74096084	rs4746172	0.9511	5.47e-05	0.947	0.0001027	0.5693	0.0004426	0.6457	0.0005702	intronic	VCL	75855842	C	T	0.613818	0.377	0.67	0.7352	0.7584	0.5051
13	21481826	rs7336891	0.8171	5.48e-05	0.8863	4.23e-05	0.4668	0.0009707	0.5941	0.0003374	intergenic	ZDHHC20;MICU2	22055965	A	C	0.485224	0.6984	0.5288	0.2897	0.4523	0.5327
17	21656630	rs1196203882	1.125	5.48e-05	1.126	0.0001087	0.7451	0.0002254	0.9679	3.3e-05	intergenic	INC02693;KCNJ1	NA	NA	NA	NA	NA	NA	NA	NA	NA
4	116215168	rs1395438	1.169	5.5e-05	1.285	1.68e-05	0.7726	0.0004089	0.9757	0.0001046	intergenic	NDST4;MIR1973	117136324	C	T	0.238818	0.0903	0.1844	0.5893	0.1064	0.093
14	76846834	rs34159247	0.7972	5.52e-05	0.8513	4.72e-05	0.4949	0.000894	0.5292	0.001463	intronic	LRRC74A	77313177	A	AC	0.650559	0.8651	0.6124	0.528	0.6451	0.6278
9	10297439	rs633062	0.8702	5.53e-05	0.8355	0.0001868	0.5743	0.0002813	0.6798	0.0001365	intronic	PTPRD	10297439	G	A	0.348243	0.253	0.389	0.5219	0.1799	0.3558
13	21553655	rs145031574	-0.8522	5.54e-05	-0.925	4.56e-05	-0.4831	0.0007524	-0.5695	0.0006478	intronic	MICU2	22127794	A	C	0.331869	0.3046	0.3617	0.2216	0.4085	0.409
1	150836640	rs10305695	1.919	5.58e-05	1.941	7.61e-05	1.23	0.004852	1.455	0.001344	intronic	ARNT	150809116	C	T	0.0117812	0	0.0231	0.0008	0.0358	0.0061
11	40365468	rs67842779	1.17	5.58e-05	1.189	6.68e-05	0.769	0.0005041	0.8329	0.0007969	intronic	LRRC4C	40387018	C	T	0.140974	0.255	0.0951	0.0628	0.1103	0.1933
13	21558276	rs9552451	0.946	5.58e-05	1.182	4.71e-06	0.4949	0.003225	0.7577	8.78e-05	intronic	MICU2	22132415	C	T	0.366613	0.6944	0.3703	0.1906	0.1879	0.4479
15	63350744	rs35255119	0.9338	5.6e-05	0.9748	5.92e-05	0.3202	0.07134	0.4014	0.04463	intronic	CA12	63642943	GT	G	0.192891	0.0149	0.3026	0.2625	0.2087	0.1881
4	189988487	rs7440565	-1.096	5.61e-05	-1.246	1.89e-05	-0.721	0.0001808	-0.7578	0.0006232	intergenic	FRG1;FRG2	NA	NA	NA	NA	NA	NA	NA	NA	NA
20	25914628	rs62212269	-1.106	5.61e-05	-1.134	8.01e-05	-0.8779	1.44e-05	-1.07	3.5e-06	intergenic	01926935;LINC0	NA	NA	NA	NA	NA	NA	NA	NA	NA
4	29744828	rs16882211	2.71	5.64e-05	2.73	5.49e-05	1.968	0.0005433	2.221	0.0003426	intergenic	INC02472;PCDH	29746450	C	T	0.0339457	0	0.0259	0.0817	0.0378	0.0061
9	10303934	rs609528	0.8532	5.64e-05	0.8514	0.0001076	0.6306	5.79e-05	0.6778	0.0001118	intronic	PTPRD	10303934	G	A	0.383187	0.251	0.3991	0.646	0.1879	0.3538
10	31789372	rs11598961	1.427	5.64e-05	1.565	1.29e-05	0.7789	0.006901	0.9743	0.001922	intergenic	ZEB1;ARHGAP12	32078300	A	G	0.0499201	0.003	0.085	0.0832	0.0666	0.0112
3	156877051	rs75207295	2.031	5.66e-05	1.885	0.0003328	1.373	0.001681	1.435	0.002117	intronic	LEKR1	156594840	A	C	0.0131789	0	0.0187	0.0023	0.0457	0.0041
20	29773940	rs1345229444	1.132	5.68e-05	1.054	0.0003595	0.6703	0.001792	0.669	0.006056	intergenic	M242B;MIR447	NA	NA	NA	NA	NA	NA	NA	NA	NA
9	10310942	rs605257	0.8709	5.75e-05	0.8398	0.0001864	0.588	0.0002197	0.6976	0.0001002	intronic	PTPRD	10310942	A	T	0.346246	0.253	0.3934	0.5129	0.1839	0.3507
1	18261005	rs16861762	3.199	5.78e-05	3.031	0.0002073	1.175	0.01163	1.269	0.01693	intronic	IGSF21	18587499	C	G	0.0353435	0.1607	0.0101	0	0	0.0082
7	79179233	rs76403905	1.664	5.81e-05	1.679	8.3e-05	1.034	0.000391	0.9119	0.007126	intronic	MAGI2	78808549	T	C	0.0848642	0.1518	0.0922	0.1415	0.0119	0.0092
10	74094038	rs10824069	0.932	5.81e-05	0.8538	0.0002993	0.5061	0.001444	0.5877	0.001374	intronic	VCL	75853796	C	T	0.610423	0.376	0.6715	0.7337	0.7495	0.499
6	151648191	rs3020302	-1.319	5.83e-05	-1.484	4.88e-05	-0.8882	1.26e-05	-1.119	1.2e-05	intergenic	CCDC170;ESR1	151969326	A	G	0.167532	0.0298	0.1398	0.2035	0.1978	0.2495
10	74157673	rs10762577	0.9554	5.83e-05	0.8995	0.0002071	0.5226	0.001449	0.6462	0.0006804	intronic	ADK	75917431	A	G	0.63119	0.3849	0.6772	0.7912	0.7584	0.5051
7	61065601	rs1330767619	1.154	5.88e-05	1.282	1.77e-05	0.8542	7.73e-05	0.8409	0.0006392	intergenic	NONE;NONE	NA	NA	NA	NA	NA	NA	NA	NA	NA
9	10317731	rs76584673	0.8605	5.88e-05	0.8414	0.0001374	0.5511	0.0006229	0.6314	0.0004492	intronic	PTPRD	10317731	C	A	0.339856	0.1994	0.3934	0.6097	0.161	0.2658
13	21537382	rs11841562	-0.8643	5.9e-05	-0.9395	5.24e-05	-0.6245	2.49e-05	-0.655	0.0001503	intronic	MICU2	22111521	C	A	0.277756	0.3006	0.3184	0.0582	0.3897	0.407
1	24978769	rs6600251	-0.9942	5.92e-05	-1.065	5.07e-05	-0.5128	0.001371	-0.7247	0.0002309	intergenic	RUNX3;MIR4425	25305260	T	C	0.763578	0.7014	0.8156	0.8865	0.672	0.7188
3	63542373	rs1039631	0.8002	5.92e-05	0.8401	5.68e-05	0.3983	0.003939	0.5656	0.0004541	ncRNA_intronic	SYNPR-AS1	63528049	G	A	0.373802	0.2817	0.4135	0.5053	0.3588	0.2781
3	42101624	rs6806570	1.306	5.95e-05	1.189	0.0004694	0.7441	0.002402	0.7374	0.009076	intronic	TRAK1	42143116	A	C	0.102835	0.0516	0.0605	0.202	0.0875	0.0675
3	42104777	rs13322778	1.306	5.95e-05	1.189	0.0004694	0.7441	0.002402	0.7374	0.009076	intronic	TRAK1	42146269	T	C	0.103035	0.0516	0.0605	0.2027	0.0875	0.0675
2	4198872	rs2118380	1.118	5.98e-05	1.074	0.0001665	0.6651	0.001425	0.7454	0.00195	intergenic	05373394;LINC0	4246462	C	A	0.757588	0.9881	0.8588	0.4175	0.8101	0.8538
7	123560687	rs1589877	1.131	5.98e-05	1.07	0.0002787	0.4774	0.01984	0.8458	0.0002423	intronic	NDUFA5	123200741	C	G	0.188299	0.0565	0.1138	0.3139	0.1421	0.2546
1	24972913	rs7555518	-0.9345	6e-05	-0.9438	9.61e-05	-0.4751	0.001518	-0.7199	0.0001268	intergenic	RUNX3;MIR4425	25299404	T	A	0.636781	0.6746	0.745	0.5908	0.5437	0.6789
1	24972941	rs12407074	-0.9345	6e-05	-0.9438	9.61e-05	-0.4751	0.001518	-0.7199	0.0001268	intergenic	RUNX3;MIR4425	25299432	T	A	0.636981	0.6746	0.745	0.5915	0.5437	0.6789
1	24973146	rs7550635	-0.9345	6e-05	-0.9438	9.61e-05	-0.4751	0.001518	-0.7199	0.0001268	intergenic	RUNX3;MIR4425	25299637	C	G	0.636581	0.6746	0.745	0.5908	0.5427	0.6789
1	24973257	rs34115476	-0.9345	6e-05	-0.9438	9.61e-05	-0.4751	0.001518	-0.7199	0.0001268	intergenic	RUNX3;MIR4425	25299748	CCA	C	0.636981	0.6746	0.745	0.5923	0.5437	0.6779
1	24973325	rs7542123	-0.9345	6e-05	-0.9438	9.61e-05	-0.4751	0.001518	-0.7199	0.0001268	intergenic	RUNX3;MIR4425	25299816	G	A	0.637181	0.6746	0.745	0.5923	0.5437	0.6789
1	24973345	rs7513156	-0.9345	6e-05	-0.9438	9.61e-05	-0.4751	0.001518	-0.7199	0.0001268	intergenic	RUNX3;MIR4425	25299836	T	C	0.637181	0.6746	0.745	0.5923	0.5437	0.6789
1	24974141	rs12036815	-0.9345	6e-05	-0.9438	9.61e-05	-0.4751	0.001518	-0.7199	0.0001268	intergenic	RUNX3;MIR4425	25300632	C	T	0.639177	0.6756	0.7464	0.5976	0.5437	0.68
1	24974986	rs7545113	-0.9345	6e-05	-0.9438	9.61e-05	-0.4751	0.001518	-0.7199	0.0001268	intergenic	RUNX3;MIR4425	25301477	T	A	0.63758	0.6756	0.745	0.5923	0.5437	0.68
1	24975378	rs1609996	-0.9345	6e-05	-0.9438	9.61e-05	-0.4751	0.001518	-0.7199	0.0001268	intergenic	RUNX3;MIR4425	25301869	T	C	0.63758	0.6756	0.745	0.5923	0.5437	0.68
7	79174856	rs11982534	1.658	6.01e-05	1.672	8.62e-05	1.001	0.0005844	0.9102	0.007242	intronic	MAGI2	78804172	T	A	0.0850639	0.1478	0.0937	0.1445	0.0119	0.0092
11	40365244	rs11035806	1.119	6.02e-05	1.154	5.66e-05	0.7101	0.0009677	0.7895	0.001026	intronic	LRRC4C	40386794	T	C	0.142971	0.255	0.0965	0.0719	0.1103	0.1902
6	62885058	rs1538086	-0.9013	6.05e-05	-0.8228	0.0004123	-0.5433	0.0002482	-0.6421	0.0002599	intergenic	KHDRBS2;LGSN	63594963	C	T	0.673123	0.7778	0.7233	0.5983	0.5924	0.7137
3	30226755	rs9864657	-0.9414	6.07e-05	-0.9936	7.41e-05	-0.4642	0.002082	-0.5881	0.001192	intergenic	BMS3;LINC0198	30268246	T	C	0.427716	0.2252	0.3761	0.7262	0.4225	0.2751
10	74188662	rs10762581	0.9506	6.07e-05	0.8935	0.0002217	0.5223	0.001303	0.645	0.0006147	intronic	ADK	75948420	C	A	0.629792	0.3859	0.6772	0.7852	0.7584	0.5051
13	21549622	rs11452621	-0.8178	6.08e-05	-0.9405	2.44e-05	-0.5174	0.0002387	-0.5898	0.0003101	intronic	MICU2	22123761	C	CA	0.32528	0.3056	0.3473	0.2194	0.3887	0.408
13	21557662	rs2761911	-0.8178	6.08e-05	-0.9405	2.44e-05	-0.5065	0.0003207	-0.5695	0.0004773	intronic	MICU2	22131801	T	G	0.325479	0.3046	0.3473	0.2209	0.3887	0.408
13	21559898	rs2761912	-0.8178	6.08e-05	-0.9405	2.44e-05	-0.5065	0.0003207	-0.5695	0.0004773	intronic	MICU2	22134037	C	T	0.32508	0.3046	0.3473	0.2194	0.3887	0.408
13	21563127	rs2476639	-0.8178	6.08e-05	-0.9405	2.44e-05	-0.5065	0.0003207	-0.5695	0.0004773	intronic	MICU2	22137266	T	G	0.324281	0.3036	0.3473	0.2201	0.3887	0.4039
13	21490421	rs9550740	0.9277	6.09e-05	1.085	1.53e-05	0.4659	0.005511	0.7544	9.66e-05	intergenic	ZDHHC20;MICU2	22064560	T	C	0.412939	0.6617	0.3977	0.3676	0.1998	0.4479
15	81212029	rs4614682	-1.277	6.11e-05	-1.285	0.0001477	-0.5378	0.004363	-0.6833	0.003188	intronic	IL16	81504370	G	A	0.232428	0.378	0.2882	0.1188	0.0974	0.3354
1	112851814	rs11587645	1.196	6.12e-05	1.054	0.000783	0.4562	0.05659	0.6591	0.0122	intergenic	C01356;MIR113	113394436	T	C	0.0628994	0.0506	0.0879	0.0015	0.1203	0.0818

20	29639245	rs1487731939	1.425	6.12e-05	1.586	1.23e-05	0.9511	0.001065	1.102	0.0005409	intergenic	AM242B;MIR447	NA	NA	NA	NA	NA	NA	NA	NA	NA
22	17026441	rs1241084219	1.111	6.12e-05	1.113	0.0001251	0.608	0.002168	0.7606	0.0007686	intergenic	GAB4;CECR7	NA	NA	NA	NA	NA	NA	NA	NA	NA
1	200688020	rs7526365	-0.9443	6.15e-05	-0.907	0.0002231	-0.409	0.005503	-0.6558	0.0002564	ncRNA_intronic	DDX59-AS1	200657148	G	C	0.257388	0.2927	0.2233	0.1483	0.3827	0.2638
1	200689851	rs10920005	-0.9443	6.15e-05	-0.907	0.0002231	-0.3988	0.006813	-0.6558	0.0002564	ncRNA_intronic	DDX59-AS1	200658979	T	A	0.257388	0.2917	0.2233	0.149	0.3827	0.2638
1	200689889	rs10920006	-0.9443	6.15e-05	-0.907	0.0002231	-0.3988	0.006813	-0.6558	0.0002564	ncRNA_intronic	DDX59-AS1	200659017	C	T	0.257188	0.2917	0.2233	0.1483	0.3827	0.2638
1	200690727	rs7545177	-0.9443	6.15e-05	-0.907	0.0002231	-0.3988	0.006813	-0.6558	0.0002564	ncRNA_intronic	DDX59-AS1	200659855	T	C	0.257188	0.2927	0.2233	0.1475	0.3827	0.2638
1	200692968	rs7519383	-0.9443	6.15e-05	-0.907	0.0002231	-0.3988	0.006813	-0.6558	0.0002564	ncRNA_intronic	DDX59-AS1	200662096	A	G	0.257388	0.2927	0.2233	0.1483	0.3827	0.2638
1	200694546	rs10920008	-0.9443	6.15e-05	-0.907	0.0002231	-0.3988	0.006813	-0.6558	0.0002564	downstream	DDX59-AS1	200663674	A	G	0.257388	0.2927	0.2233	0.1483	0.3827	0.2638
10	31790296	rs7909082	1.424	6.15e-05	1.546	1.66e-05	0.7677	0.007828	0.9596	0.002292	intergenic	ZEB1;ARHGAP12	32079224	G	A	0.0497204	0.002	0.085	0.0832	0.0666	0.0112
18	58671446	rs7506913	0.8047	6.15e-05	0.7043	0.0006514	0.4612	0.0007714	0.576	0.0002737	ncRNA_intronic	LOC101927322	56338678	A	C	0.634784	0.4891	0.6081	0.975	0.4751	0.5082
9	20305588	rs78354010	1.517	6.16e-05	1.518	7.43e-05	0.6185	0.05313	0.9405	0.006683	intergenic	SLC24A2;MLLT3	20305586	A	G	0.0383387	0.001	0.0692	0.0499	0.0497	0.0276
13	21462677	rs1830940	-0.8116	6.17e-05	-0.8705	5e-05	-0.5488	0.0001066	-0.5823	0.0003323	intergenic	ZDHHC20;MICU2	22036816	A	G	0.366214	0.2411	0.3876	0.3593	0.4473	0.4059
15	82831567	rs17158257	1.104	6.18e-05	1.096	0.000124	0.6222	0.003156	0.6359	0.00782	intronic	WHAMM	83500319	A	G	0.152356	0.1954	0.196	0.1861	0.1213	0.0634
10	74507125	rs6480739	0.9625	6.21e-05	0.9477	0.0001464	0.5176	0.001522	0.6207	0.001025	ncRNA_intronic	LOC102723439	76266883	A	G	0.618211	0.3631	0.67	0.7519	0.7624	0.5153
13	21572274	rs12430739	-0.8176	6.22e-05	-0.9406	2.49e-05	-0.5113	0.000286	-0.5684	0.0005001	intronic	MICU2	22146413	A	T	0.321685	0.3036	0.3429	0.2095	0.3887	0.408
20	29804896	rs1295395553	-1.103	6.22e-05	-1.237	2.05e-05	-0.6148	0.002027	-0.9247	5.42e-05	intergenic	AM242B;MIR447	NA	NA	NA	NA	NA	NA	NA	NA	NA
1	30348467	rs623746	1.129	6.23e-05	1.131	9.19e-05	0.7878	0.0003621	0.905	0.0002246	intergenic	LINC01648;MATN	30821314	A	G	0.872204	0.8413	0.8357	0.9085	0.8966	0.8558
3	150650034	rs1879808	0.8216	6.23e-05	0.9544	1.49e-05	0.4324	0.00213	0.5965	0.0003364	intergenic	SELENOT;ERICH6	150367821	T	A	0.421126	0.4117	0.5562	0.2542	0.5487	0.4294
3	150650036	rs1879807	0.8216	6.23e-05	0.9544	1.49e-05	0.4324	0.00213	0.5965	0.0003364	intergenic	SELENOT;ERICH6	150367823	C	A	0.421326	0.4097	0.5562	0.2534	0.5497	0.4325
15	82901080	rs17841162	1.16	6.23e-05	1.154	0.0001291	0.7121	0.001288	0.7194	0.004159	intronic	HOMER2	83569832	C	G	0.134784	0.2163	0.1816	0.1498	0.0765	0.0573
22	36825816	rs6000429	0.9869	6.23e-05	0.9227	0.0003616	0.5235	0.008163	0.7234	0.0008187	intergenic	PVALB;NCF4-AS1	37221860	G	A	0.0914537	0.0724	0.098	0.0991	0.1262	0.0603
9	10296904	rs597087	0.8628	6.27e-05	0.8511	0.0001397	0.6111	0.000123	0.6771	0.0001426	intronic	PTPRD	10296904	G	T	0.353035	0.252	0.389	0.5635	0.1789	0.3262
13	21537556	rs9316442	0.8103	6.3e-05	0.8938	3.87e-05	0.4404	0.00197	0.5533	0.0008045	intronic	MICU2	22111695	G	A	0.448283	0.6984	0.4971	0.1906	0.4215	0.5317
13	21548338	rs17679426	-0.8174	6.32e-05	-0.9415	2.51e-05	-0.5143	0.000266	-0.5874	0.0003347	intronic	MICU2	22122477	A	T	0.324281	0.3006	0.3473	0.2201	0.3887	0.407
8	4605028	rs2617101	-0.7817	6.35e-05	-0.8049	0.0001001	-0.4167	0.001647	-0.5335	0.0006081	intronic	CSMD1	4462550	G	C	0.561901	0.4871	0.4568	0.8669	0.4026	0.4652
1	24973551	rs10903121	-0.9259	6.36e-05	-0.9438	9.61e-05	-0.4742	0.001541	-0.7183	0.0001289	intergenic	RUNX3;MIR4425	25300042	G	A	0.63738	0.6756	0.745	0.5923	0.5437	0.6789
1	24973765	rs11249218	-0.9259	6.36e-05	-0.9438	9.61e-05	-0.4636	0.002009	-0.7183	0.0001289	intergenic	RUNX3;MIR4425	25300256	A	G	0.63758	0.6756	0.745	0.5923	0.5437	0.68
6	62885046	rs4579334	-0.8973	6.37e-05	-0.8191	0.0004289	-0.5412	0.0002607	-0.6391	0.000274	intergenic	KHDRBS2;LGSN	63594951	G	A	0.673722	0.7778	0.7233	0.6006	0.5924	0.7137
13	21466418	rs1148976	-0.787	6.37e-05	-0.8353	5.9e-05	-0.5212	0.0001906	-0.5969	0.0002366	intergenic	ZDHHC20;MICU2	22040557	A	G	0.438498	0.255	0.4366	0.4879	0.5288	0.4693
1	200688529	rs7526853	-0.936	6.4e-05	-0.9265	0.0001796	-0.4057	0.006073	-0.6575	0.0002474	ncRNA_intronic	DDX59-AS1	200657657	G	A	0.257188	0.2917	0.2233	0.1483	0.3827	0.2638
20	30372471	rs77916748	1.227	6.4e-05	1.128	0.0005085	0.8202	0.00109	0.7782	0.00578	intergenic	LINC01597;FRG1B	NA	NA	NA	NA	NA	NA	NA	NA	NA
6	62880207	rs7773896	-0.9122	6.45e-05	-0.8165	0.0005051	-0.5527	0.0002151	-0.6443	0.0002779	intergenic	KHDRBS2;LGSN	63590112	A	C	0.67492	0.7778	0.7233	0.6021	0.5934	0.7168
6	62880507	rs9343307	-0.9122	6.45e-05	-0.8165	0.0005051	-0.5527	0.0002151	-0.6443	0.0002779	intergenic	KHDRBS2;LGSN	63590412	A	C	0.673722	0.7778	0.7233	0.5976	0.5934	0.7168
11	40358628	rs35188639	1.116	6.45e-05	1.132	7.48e-05	0.7492	0.0003373	0.8141	0.0006188	intronic	LRRC4C	40380178	G	A	0.189297	0.253	0.1124	0.239	0.1113	0.1912
4	116224589	rs2389142	1.202	6.47e-05	1.288	3.05e-05	0.7855	0.0003241	1.029	6.46e-05	intergenic	NDST4;MIR1973	117145745	A	T	0.222644	0.0903	0.1686	0.4365	0.1054	0.229
1	30345613	rs415965	1.13	6.48e-05	1.128	0.0001004	0.8214	0.0002342	0.9283	0.0001729	intergenic	LINC01648;MATN	30818460	T	C	0.874002	0.8413	0.8401	0.9115	0.8966	0.8579
1	200692534	rs7539646	-0.9404	6.48e-05	-0.9029	0.0002342	-0.4023	0.006425	-0.6653	0.0002238	ncRNA_intronic	DDX59-AS1	200661662	G	A	0.257388	0.2927	0.2233	0.1483	0.3827	0.2638
3	36138277	rs13086896	0.8794	6.5e-05	0.9873	2.23e-05	0.3829	0.02358	0.5498	0.003651	intergenic	ARPP21;STAC	36179769	A	G	0.158546	0.0734	0.2032	0.1074	0.2763	0.1626
7	61026567	rs1202291219	-1.093	6.52e-05	-0.942	0.0009743	-0.7718	9.67e-05	-0.8946	9.18e-05	intergenic	NONE;NONE	NA	NA	NA	NA	NA	NA	NA	NA	NA
11	47287512	rs61896050	-0.881	6.52e-05	-1.024	1.73e-05	-0.551	0.0002985	-0.6949	8.09e-05	intronic	MADD	47309063	T	C	0.480431	0.7262	0.3372	0.5166	0.3072	0.4581
22	11338752	rs1378082003	3.039	6.53e-05	3.139	3.76e-05	2.016	0.002673	2.479	0.0003847	intergenic	51FP;LOC102723	NA	NA	NA	NA	NA	NA	NA	NA	NA
7	81663222	rs17490715	1.754	6.54e-05	1.778	7.02e-05	1.035	0.005836	1.283	0.001492	ncRNA_intronic	LOC100128317	81292538	G	A	0.0628994	0.006	0.0504	0.1528	0.0348	0.0378
6	62872346	rs7739641	-0.9071	6.57e-05	-0.8151	0.0004897	-0.5166	0.0004559	-0.6058	0.0005107	intergenic	KHDRBS2;LGSN	63582251	C	T	0.698283	0.7778	0.7262	0.6853	0.5994	0.7157
7	151066329	rs34740196	0.8035	6.57e-05	0.8513	5.02e-05	0.44	0.003238	0.495	0.003088	intronic	SLC4A2	150763416	C	G	0.224441	0.3165	0.2363	0.0575	0.3519	0.2157
6	53695730	rs74317643	1.658	6.58e-05	1.855	1.03e-05	1.407	7.92e-05	1.338	0.0004511	intergenic	KLHL31;LRRC1	53560528	C	T	0.0297524	0	0.0591	0.003	0.0964	0.0072
4	116196188	rs1395436	1.152	6.6e-05	1.278	1.82e-05	0.8467	0.0001006	0.9953	6.55e-05	intergenic	NDST4;MIR1973	117117344	G	T	0.707468	0.9087	0.8026	0.2595	0.8877	0.8528
10	74504349	rs4561146	0.9611	6.63e-05	0.9039	0.000262	0.517	0.001515	0.6194	0.00105	intronic	ADK	76264107	A	C,G	51901,0.0005990	0.3631,0	0.6715,0	0.7504,0.0023	0.7624,0	0.5204,0
16	28277922	rs4635350	0.8269	6.63e-05	0.8512	0.0001001	0.4253	0.002561	0.683	4.81e-05	intergenic	XPO6;SBK1	28289243	C	T	0.465655	0.7054	0.4352	0.2368	0.4771	0.5378
13	21509859	rs3814795	0.8024	6.64e-05	0.9306	2.03e-05	0.4444	0.001885	0.5649	0.0006227	intronic	MICU2	22083998	A	C	0.448882	0.6954	0.5	0.1989	0.4165	0.5297
13	21552161	rs75565407	0.8154	6.64e-05	0.897	4.37e-05	0.4447	0.00183	0.5449	0.001007	intronic	MICU2	22126300	C	G	0.44988	0.6944	0.4986	0.1989	0.4215	0.5317
13	21575158	rs68090535	0.9385	6.65e-05	1.171	6.04e-06	0.5042	0.002762	0.7606	8.37e-05	intronic	MICU2	22149297	TAC	T	0.364816	0.6935	0.3703	0.1899	0.1879	0.4407
10	110176913	rs17127151	1.087	6.66e-05	1.085	0.0001239	0.7042	0.0002702	0.7633	0.0004977	intergenic	OC105378479;M	111936671	G	A	0.238419	0.3948	0.1023	0.1747	0.1203	0.3814
13	21526810	rs35261213	0.8119	6.67e-05	0.8712	5.79e-05	0.4636	0.0012	0.5703	0.0005861	intronic	MICU2	22100949	GA	G	0.516174	0.3085	0.4827	0.6868	0.5666	0.4714
10	125619829	rs189758	1.612	6.68e-05	1.723	3.37e-05	1.013	0.0008522	1.113	0.0009581	intronic	TEX36	127308398	A	G	0.0972444	0.1161	0.0749	0.0703	0.0447	0.184
10	125624328	rs297253	1.612	6.68e-05	1.723	3.37e-05	1.013	0.0008522	1.113	0.0009581	intronic	TEX36	127312897	G	A	0.0970447	0.1161	0.0749	0.0696	0.0447	0.184
16	33042154	rs796644552	-1.149	6.75e-05	-1.13	0.0001979	-0.6515	0.0007126	-0.8099	0.0003245	intergenic	LC6A10P;TP53TG	NA	NA	NA	NA	NA	NA	NA	NA	NA
1	161991307	rs12044824	1.684	6.76e-05	1.833	3.01e-05	1.09	0.0006376	1.415	0.0001157	intergenic	OLFML2B	161961097	C	A	0.0838658	0.121	0.0677	0.1384	0.0219	0.047
2	64621942	rs13395792	-0.8039	6.77e-05	-0.7094	0.0006714	-0.3781	0.00527	-0.5023	0.001498	intergenic	NC02579;SERTAI	64849076	T	G	0.552117	0.502	0.4942	0.705	0.3996	0.5951
2	109019981	rs17034808	1.224	6.77e-05	1.255	7.46e-05	0.6475	0.004975	0.9636	0.0002392	intergenic	EDAR;SH3RF3-AS	109636437	G	A	0.16234	0.1429	0.0821	0.3487	0.0706	0.0818
20	30175364	rs1290414647	-1.181	6.8e-05	-1.25	4.86e-05	-0.8966	5.61e-05	-0.8858	0.0004978	intergenic	IR4477A;LINC01S	NA	NA	NA	NA	NA	NA	NA	NA	NA
10	74140704	rs7896966	0.9417	6.81e-05	0.859	0.0003649	0.4918	0.00235	0.6111	0.001083	intronic	AP3M1	75900462	C	A	0.629593	0.3819	0.6772	0.792	0.7565	0.501
4	29744676	rs17735113	2.073	6.89e-05	2.133	5.36e-05	1.769	5.99e-05	1.691	0.0004544	intergenic	LINC02472;PCDH	29746298	A	G	0.0427316	0	0.0331	0.0908	0.0567	0.0143
4	29697898	rs11943301	2.115	6.92e-05	2.128	7.68e-05	1.151	0.003448	1.712	0.0002962	intergenic	LINC02472;PCDH	29699520	A	G	0.0742812	0	0.0317	0.233	0.0368	0.0051
6	62879936	rs7739136	-0.899	6.92e-05	-0.8198	0.0004646	-0.5543	0.0001965	-0.6416	0.0002763	intergenic	KHDRBS2;LGSN	63589841	T	A	0.673722	0.7778	0.7233	0.5983	0.5924	0.7168
16	33042153	rs74770893	-1.087	6.94e-05	-1.179	4.82e-05	-0.7171	0.0002172	-0.7555	0.0007257	intergenic	LC6A10P;TP53TG	NA	NA	NA	NA	NA	NA	NA	NA	NA
15	82828837	rs17158267	1.105	6.98e-05	1.108	0.0001238	0.6118	0.004349	0.6371	0.008648	intronic	WHAMM	83497589	G	A	0.146166	0.1716	0.196	0.1861	0.1203	0.0573
13	21549537	rs4770172	-0.8112	7.02e-05	-0.9315	2.97e-05	-0.5146	0.0002594	-0.5852	0.0003473	intronic	MICU2	22123676	A	G	0.327276	0.3065	0.3473	0.2239	0.3887	0.411
13	21567448	rs2249901	-0.7825	7.02e-05	-0.9018	2.29e-05	-0.4272	0.001788	-0.5072	0.001223	intronic	MICU2	22141587	C	G	0.349641	0.2917	0.4006	0.2231	0.4533	0.4376
1	24979116	rs6690896	-0.9855	7.03e-05	-1.067	5.42e-05	-0.484	0.002356	-0.7509	0.0001442	intergenic	RUNX3;MIR4425	25305607	G	A	0.779952	0.7083	0.8156	0.9281	0.667	0.7444
1	24979182	rs6659811	-0.9855	7.03e-05	-1.067	5.42e-05	-0.4733	0.002867	-0.7337	0.0001949	intergenic	RUNX3;MIR4425	25305673	T	G	0.778754	0.7103	0.8127	0.9168	0.669	0.7515
9	10306103	rs614638	0.8638	7.08e-05	0.8465	0.00017	0.5933	0.0001949	0.7014	9.35e-05	intronic	PTPRD	10306103	C	T	0.347444	0.251	0.3934	0.5166	0.1849	0.3528
4	123863431	rs3113396	0.8375	7.09e-05	0.8894	5.3e-05	0.4223	0.003839	0.5887	0.0004857	ncRNA_intronic	LINC01091	124784586	G	A	0.660543	0.5992	0.7248	0.643	0.6302	0.7331
1	200687819	rs7524010	-0.9325	7.12e-05	-0.896	0.0002529	-0.4091	0.005508	-0.6506	0.0002803	ncRNA_intronic	DDX59-AS1	200656947	G	A	0.257588	0.2917	0.2219	0.1483	0.3827	0.2669
16	33739450	rs76495562	-1.704	7.14e-05	-1.565	0.0002792	-1.046	8.27e-06	-1.206	3.26e-05	intergenic	C390705;ENPP7F	NA	NA	NA	NA	NA	NA	NA	NA	NA
9	80006326	rs17729164	1.517	7.15e-05	1.538	8.02e-05	0.8698	0.005545	1.005	0.003391	ncRNA_intronic	LINC01507	82621241	C	T	0.0349441	0	0.0836	0.0053	0.0915	0.0184
1	200687904	rs7548530	-0.9335	7.18e-05	-0.8959	0.0002571	-0.3844	0.009001	-0.6434	0.0003198	ncRNA_intronic	DDX59-AS1	200657032	A	G	0.257188	0.2927	0.2219	0.1467	0.3837	0.2648
11	69984577	rs75130418	1.029	7.2e-05	0.9225	0.0005989	0.5274	0.008676	0.7811	0.0004847	intergenic	FGF3;ANO1	69830683	C	A	0.0778754	0.1667	0.0793	0.0083	0.1153	0.0409
5	127991198	rs145175012	1.883	7.23e-05	1.79	0.0001764	1.298	0.0007941	1.245	0.002972	ncRNA_intronic	LINC01184	127326890	C	T	0.0417332	0.0119	0.0317	0.0045	0.0626	0.1084
7	62336478	rs78413752	-1.307	7.23e-05	-1.218	0.0003561	-0.891	1.58e-05	-1.04	3.75e-05	intergenic	NONE;ZNF733P	NA	NA	NA	NA	NA	NA	NA	NA	NA
1	124980057	rs1369301042	-1.153	7.27e-05	-1.184	0.0001124	-0.8867	6.85e-06	-1.03	1.09e-05	intergenic	NONE;NONE	NA	NA	NA	NA	NA	NA	NA	NA	NA
6	62884578	rs6940961	-0.8956	7.3e-05	-0.8166	0.0004847	-0.5392	0.0002852	-0.6367	0.0003061	intergenic	KHDRBS2;LGSN	63594483	G	A	0.672923	0.7778	0.7248	0.5968	0.5924	0.7137
1	200689919	rs10920007	-0.9342	7.31e-05	-0.893	0.0002763	-0.3955	0.00729	-0.6501	0.0002896	ncRNA_intronic	DDX59-AS1	200659047	C	T	0.257188	0.2917	0.2233	0.1483	0.3827	0.2638
7	159056130	rs3793217	-1.535	7.32e-05	-1.625	0.0001468	-0.8177	0.0001377	-0.9679	0.0002523	intronic	VIPR2	158848821	A	G	0.0920527	0.1409	0.085	0.0166	0.1431	0.0961
9	24904992	rs974861	-1.006	7.35e-05	-0.9716	0.0002325	-0.5117	0.00126	-0.6924	0.0003055	intergenic	IZUMO3;TUSC1	24904990	C	T	0.580871	0.7212	0.781	0.1346	0.7237	0.7505
10	24541142	rs10828669	0.7714	7.36e-05	0.763	0.0001898	0.517	0.0002298	0.5953	0.0002848	intronic	KIAA1217	24830071	A	G	0.375599	0.3135	0.3847	0.4887	0.3499	0.3067
22	12290021	rs1293276571	1.345	7.36e-05	1.185	0.0005339	0.7501	0.0002295	0.8907	0.0002747	intergenic	OC102723769;NO	NA	NA	NA	NA	NA	NA	NA	NA	NA
7	79135792	rs9886174	1.54	7.37e-05	1.533	0.0001209	0.7993	0.00376	0.8458	0.009613	intronic	MAGI2	78765108	T	C	0.119409	0.1458	0.1023	0.2723	0.0119	0.0082
10	74473690	rs4338470	0.9537	7.38e-05	0.9	0.0002739	0.4754	0.002943	0.5774	0.002001	intronic	ADK	76233448	G	A	0.557308	0.376	0.6441	0.5287	0.7555	0.5174
19	14485359	rs1187810	-0.8129	7.38e-05	-0.8835	4.53e-05	-0.5002	0.0003584	-0.586	0.0004204	intronic	GIPC1	14596171	A	T	0.564097	0.2589	0.5764	0.7625	0.502	0.6656
1	24976004	rs2135755	-0.9224	7.39e-05	-0.9304	0.0001191	-0.5123	0.0007734	-0.7298	0.0001151	intergenic	RUNX3;MIR4425	25302495	G	A	0.636981	0.6756	0.7421	0.5915	0.5437	0.68
15	82818558	rs17841165	1.088	7.39e-05	1.099	0.0001189	0.5843	0.005628	0.6344	0.007938	intronic	WHAMM	83487310	T	C	0.151558	0.1915	0.196	0.1861	0.1213	0.0634
1	200620685	rs3806361	-0.9615	7.45e-05	-0.8771	0.000451	-0.4176	0.006419	-0.565	0.001757	UTR5	KIF14	200589813	C	A	0.234625	0.246	0.2262	0.0741	0.3996	0.2761
4	116182690	rs12509032	1.154	7.47e-05	1.249	2.99e-05	0.7603	0.0005137	0.9262	0.0002395	intergenic	NDST4;MIR1973	117103846	G	A	0.234425	0.0903	0.183	0.5734	0.1064	0.093
22	36840586	rs113621236	1.019	7.47e-05	0.8606	0.001466	0.497	0.01418	0.6515	0.003381	intergenic	PVALB;NCF4-AS1	37236629	T	TA	0.0786741	0.0754	0.1023	0.0166	0.1332	0.093
9	10325044	rs9987798	0.8634	7.55e-05	0.8558	0.0001558	0.6342	6.04e-05	0.6678	0.0001812	intronic	PTPRD	10325044	T	C	0.361422	0.2569	0.4006	0.5431	0.2028	0.3589
20	29804907	rs1385333560	-1.081	7.57e-05	-1.202	2.87e-05	-0.5981	0.002624	-0.8187	0.0003053	intergenic	AM242B;MIR447	NA	NA	NA	NA	NA	NA	NA	NA	NA
7	61065621	rs1405193000	1.127	7.58e-05	1.248	2.42e-05	0.8293	0.000124	0.7961	0.001218	intergenic	NONE;NONE	NA	NA	NA	NA	NA	NA	NA	NA	NA
21	30304714	rs8132185	0.8241	7.6e-05	0.8124	0.0001862	0.2046	0.1508	0.4265	0.01063	intergenic	TAP25-1;KRTAP2	31677032	C	A	0.364018	0.2688	0.3732	0.2436	0.4742	0.5051
4	116190608	rs34496746	1.147	7.66e-05	1.241	3.06e-05	0.765	0.0004556	0.9351	0.0002043	intergenic	NDST4;MIR1973	117111764	G	GA	0.229233	0.0903	0.1816	0.5522	0.1064	0.0961

10	74119753	rs703258	0.9373	7.68e-05	0.8537	0.0004108	0.5108	0.001598	0.6048	0.001235	UTR3	VCL	75879511	T	A	0.625399	0.3829	0.6758	0.7761	0.7555	0.502
10	74133146	rs7919821	0.9373	7.68e-05	0.8537	0.0004108	0.5108	0.001598	0.6048	0.001235	intronic	AP3M1	75892904	A	G	0.624601	0.3819	0.6758	0.7746	0.7555	0.501
6	72246009	rs35512671	2.412	7.69e-05	2.458	6.5e-05	1.673	0.00142	2.041	0.0001842	intronic	RIMS1	72955712	A	G	0.014377	0	0.0418	0	0.0318	0.0112
10	110176313	rs4918483	1.035	7.7e-05	1.042	0.0001262	0.7237	9.1e-05	0.6875	0.001106	intergenic	OC105378479;MX	111936071	C	T	0.28754	0.4048	0.1196	0.351	0.1243	0.3681
13	21594867	rs9509810	0.7885	7.71e-05	0.9163	2.38e-05	0.4193	0.002713	0.5248	0.001231	intronic	MICU2	22169006	C	T	0.455072	0.6855	0.5144	0.2027	0.4404	0.5317
4	94140595	rs1472983	1.3	7.75e-05	1.33	0.0001347	0.3626	0.1056	0.549	0.03291	ncRNA_intronic	LOC101929210	95061746	G	C	0.126198	0.2024	0.1974	0.1316	0.0139	0.1053
4	94144498	rs150981711	1.3	7.75e-05	1.33	0.0001347	0.3217	0.1521	0.5765	0.02596	ncRNA_intronic	LOC101929210	95065649	CTA	C	0.11901	0.2034	0.196	0.1051	0.0129	0.1053
4	94147829	rs151080514	1.3	7.75e-05	1.33	0.0001347	0.3301	0.1407	0.549	0.03291	ncRNA_intronic	LOC101929210	95068980	C	T	0.11901	0.2034	0.196	0.1051	0.0129	0.1053
13	21533995	rs7322862	0.7883	7.76e-05	0.9137	2.43e-05	0.4403	0.00178	0.5434	0.0008906	intronic	MICU2	22108134	C	T	0.454273	0.6984	0.5115	0.1906	0.4414	0.5317
13	21549461	rs7985023	0.7883	7.76e-05	0.9137	2.43e-05	0.4294	0.002255	0.5256	0.001255	intronic	MICU2	22123600	A	G	0.453674	0.6944	0.513	0.1906	0.4414	0.5317
4	116210721	rs12509875	1.149	7.77e-05	1.243	3.12e-05	0.759	0.0005135	0.9655	0.0001268	intergenic	NDST4;MIR1973	117131877	A	C	0.232628	0.0903	0.1816	0.5673	0.1064	0.093
13	21469383	rs2874195	0.9692	7.81e-05	1.164	1.3e-05	0.5655	0.0009582	0.853	1.86e-05	intergenic	ZDHHC20;MICU2	22043522	A	T	0.421526	0.7391	0.4006	0.329	0.2087	0.453
6	62862313	rs6453827	-0.902	7.84e-05	-0.8092	0.000572	-0.5127	0.0005193	-0.6006	0.0005951	intergenic	KHDRBS2;LGSN	63572218	G	A	0.698682	0.7788	0.7262	0.6861	0.5994	0.7157
1	200688025	rs7526367	-0.9137	7.86e-05	-0.8711	0.0002993	-0.4049	0.00576	-0.6359	0.0003468	ncRNA_intronic	DDX59-AS1	200657153	G	A	0.258786	0.2917	0.2233	0.1483	0.3887	0.2658
11	69991148	rs61885149	1.083	7.86e-05	0.9461	0.0009816	0.5018	0.01931	0.7882	0.0008418	intronic	ANO1	69837254	G	A	0.0694888	0.1329	0.0735	0.0083	0.1113	0.0409
11	69991563	rs61885150	1.083	7.86e-05	0.9461	0.0009816	0.5018	0.01931	0.7882	0.0008418	intronic	ANO1	69837669	G	A	0.0694888	0.1329	0.0735	0.0083	0.1113	0.0409
11	69993064	rs17160523	1.083	7.86e-05	0.9461	0.0009816	0.5018	0.01931	0.7882	0.0008418	intronic	ANO1	69839170	G	A	0.0696885	0.1329	0.0735	0.0083	0.1123	0.0409
9	10311000	rs605318	0.8557	7.88e-05	0.8398	0.0001864	0.5879	0.0002197	0.6982	9.81e-05	intronic	PTPRD	10311000	G	A	0.346046	0.253	0.3934	0.5113	0.1849	0.3507
17	54892483	rs2332303	-0.8537	7.88e-05	-0.6815	0.002179	-0.5511	0.0001757	-0.6156	3e-04	intergenic	KIF2B;TOM1L1	52969844	T	C	0.476238	0.4435	0.4726	0.6649	0.4095	0.3262
12	38082388	rs1168078881	1.096	7.89e-05	1.135	0.000103	0.8099	9.39e-05	0.8109	0.0005842	intergenic	NONE;ALG10B	NA	NA	NA	NA	NA	NA	NA	NA	NA
1	24973111	rs7550552	-0.9223	7.9e-05	-0.9333	0.0001204	-0.4676	0.001838	-0.7093	0.0001661	intergenic	RUNX3;MIR4425	25299602	C	T	0.636781	0.6746	0.745	0.5908	0.5437	0.6789
1	24974050	rs11249221	-0.9223	7.9e-05	-0.9333	0.0001204	-0.4676	0.001838	-0.7093	0.0001661	intergenic	RUNX3;MIR4425	25300541	T	C	0.63758	0.6756	0.745	0.5923	0.5437	0.68
9	10291546	rs12554683	1.958	7.92e-05	1.925	0.0001397	1.503	0.0003919	1.578	0.000519	intronic	PTPRD	10291546	T	C	0.063099	0.0456	0.2118	0.0197	0.006	0.093
15	82839647	rs17158253	1.082	7.92e-05	1.096	0.0001213	0.6292	0.002932	0.6299	0.0008428	intergenic	VHAMM;HOMER	83508399	A	T	0.152556	0.1964	0.196	0.1861	0.1203	0.0644
18	58643954	rs78418867	1.237	7.92e-05	1.151	0.0003764	0.526	0.01878	0.8657	0.0008114	intergenic	PK2;LOC1019273	56311186	C	T	0.125399	0.2708	0.0663	0.0113	0.1044	0.1933
13	21476259	rs1475154	-0.7697	7.96e-05	-0.8149	7.76e-05	-0.445	0.001182	-0.5243	0.0009761	intergenic	ZDHHC20;MICU2	22050398	G	C	0.44389	0.3036	0.4366	0.4758	0.5288	0.4632
15	82900860	rs17841163	1.138	7.96e-05	1.13	0.0001662	0.6848	0.001855	0.7	0.005111	intronic	HOMER2	83569612	T	C	0.138978	0.2163	0.1816	0.1657	0.0765	0.0573
9	124443690	rs116898749	2.324	7.97e-05	2.193	0.0002069	1.49	0.00457	1.829	0.000746	intergenic	PSMB7;NR5A1	127205969	C	T	0.0175719	0.001	0.0216	0.0015	0.0298	0.0409
11	49822985	rs377682406	-1.186	8.02e-05	-1.162	0.0002145	-0.8521	2.11e-05	-0.9989	2.7e-05	intergenic	OC440040;OR4C:	NA	NA	NA	NA	NA	NA	NA	NA	NA
8	40748534	rs10092049	0.88	8.03e-05	0.92	6.36e-05	0.4051	0.01196	0.5045	0.005668	intronic	ZMAT4	40606053	T	C	0.654752	0.619	0.6499	0.5877	0.8082	0.6278
9	10287340	rs16925713	1.967	8.03e-05	1.937	0.0001395	1.472	0.0005884	1.583	0.0005559	intronic	PTPRD	10287340	G	A	0.0577077	0.0397	0.2075	0.0068	0.005	0.093
8	6341591	rs2920655	0.9557	8.06e-05	1.022	4.35e-05	0.475	0.009138	0.691	0.0007259	intergenic	VID1;LOC100287	6199112	T	A	0.160942	0.1032	0.1297	0.2163	0.173	0.1554
9	10304610	rs612312	0.8532	8.07e-05	0.8347	0.0001984	0.5809	0.0002442	0.7006	8.98e-05	intronic	PTPRD	10304610	T	C	0.35024	0.253	0.3948	0.5219	0.1869	0.3548
1	200685659	rs6427840	-0.9178	8.09e-05	-0.907	0.0002231	-0.3941	0.007423	-0.6463	0.0002987	ncRNA_intronic	DDX59-AS1	200654787	A	G	0.256989	0.2917	0.2219	0.1483	0.3827	0.2638
1	200688131	rs34899833	-0.9178	8.09e-05	-0.907	0.0002231	-0.4063	0.005784	-0.6463	0.0002987	ncRNA_intronic	DDX59-AS1	200657259	T	TC	0.257388	0.2917	0.2233	0.1483	0.3827	0.2648
1	200688842	rs11386451	-0.9178	8.09e-05	-0.907	0.0002231	-0.3941	0.007423	-0.6463	0.0002987	ncRNA_intronic	DDX59-AS1	200657970	C	CT	0.257188	0.2917	0.2233	0.149	0.3827	0.2628
1	200693389	rs12141497	-0.9178	8.09e-05	-0.907	0.0002231	-0.3941	0.007423	-0.6463	0.0002987	ncRNA_intronic	DDX59-AS1	200662517	C	A	0.257388	0.2927	0.2233	0.1483	0.3827	0.2638
1	200693397	rs12126708	-0.9178	8.09e-05	-0.907	0.0002231	-0.3941	0.007423	-0.6463	0.0002987	ncRNA_intronic	DDX59-AS1	200662525	G	A	0.257188	0.2917	0.2233	0.1483	0.3827	0.2638
10	74513821	rs10824195	0.965	8.1e-05	0.8885	0.0003881	0.4847	0.00283	0.5978	0.001567	ncRNA_intronic	LOC102723439	76273579	G	A	0.550719	0.3621	0.6412	0.5318	0.7614	0.4898
4	116177534	rs2135574	1.113	8.14e-05	1.232	2.42e-05	0.7354	0.0005434	0.9125	0.0001891	intergenic	NDST4;MIR1973	117098690	T	C	0.272963	0.0913	0.2017	0.705	0.1093	0.0951
22	16676777	rs73159831	1.197	8.15e-05	1.239	8.44e-05	0.6402	0.008285	0.7537	0.005705	intergenic	i2P1-PARP4P3;LII	NA	NA	NA	NA	NA	NA	NA	NA	NA
9	24908450	rs1461322	-1.015	8.16e-05	-0.987	0.0002409	-0.5128	0.001486	-0.6645	0.0005992	intergenic	IZUMO3;TUSC1	24908448	G	C	0.58766	0.748	0.7824	0.1354	0.7237	0.7556
1	112827386	rs61819968	1.294	8.17e-05	1.152	0.0009147	0.6048	0.02763	0.8566	0.003706	ncRNA_intronic	LINC01356	113370008	C	A	0.0425319	0	0.0648	0.0015	0.0954	0.0716
7	123518500	rs12706536	1.231	8.18e-05	1.188	0.0002855	0.7055	0.003034	1.005	0.0001331	intronic	IQUB	123158554	T	C	0.076877	0.0228	0.0807	0.0076	0.1412	0.1575
1	30350490	rs364949	1.109	8.23e-05	1.106	0.0001276	0.7986	0.0003168	0.9053	0.0002299	intergenic	.INC01648;MATN	30823337	T	C	0.870407	0.8363	0.8357	0.907	0.8956	0.8548
1	30352534	rs625892	1.109	8.23e-05	1.106	0.0001276	0.7863	0.0003956	0.8856	0.0003212	intergenic	.INC01648;MATN	30825381	G	C	0.872404	0.8413	0.8357	0.9092	0.8966	0.8558
1	30356836	rs585572	1.109	8.23e-05	1.106	0.0001276	0.7986	0.0003168	0.9053	0.0002299	intergenic	.INC01648;MATN	30829683	A	G	0.872604	0.8413	0.8357	0.9092	0.8966	0.8569
1	30359187	rs35857070	1.109	8.23e-05	1.106	0.0001276	0.7986	0.0003168	0.9053	0.0002299	intergenic	.INC01648;MATN	30832034	AG	A	0.871605	0.8413	0.8357	0.9085	0.8966	0.8528
1	30361693	rs1754157	1.109	8.23e-05	1.106	0.0001276	0.7986	0.0003168	0.9053	0.0002299	intergenic	.INC01648;MATN	30834540	G	C	0.871805	0.8413	0.8357	0.9092	0.8966	0.8528
13	18725864	rs1259164930	-1.47	8.24e-05	-1.518	8.78e-05	-1.32	1.27e-05	-1.542	3.03e-06	intergenic	\M230C;LINC004	NA	NA	NA	NA	NA	NA	NA	NA	NA
4	116206708	rs10032121	1.145	8.25e-05	1.239	3.31e-05	0.752	0.0005816	0.9606	0.0001378	intergenic	NDST4;MIR1973	117127864	C	T	0.234225	0.0903	0.1844	0.5719	0.1064	0.093
4	116211783	rs2135577	1.145	8.25e-05	1.239	3.31e-05	0.7557	0.0005495	0.9673	0.0001257	intergenic	NDST4;MIR1973	117132939	G	A	0.233826	0.0903	0.183	0.5703	0.1074	0.093
9	10295001	rs16925748	1.952	8.25e-05	1.92	0.0001438	1.491	0.0004388	1.572	0.0005468	intronic	PTPRD	10295001	A	G	0.0626997	0.0446	0.2118	0.0197	0.005	0.093
9	10305780	rs613283	0.8534	8.29e-05	0.8375	0.000195	0.5841	0.0002401	0.6868	0.0001271	intronic	PTPRD	10305780	C	T	0.347244	0.251	0.3934	0.5166	0.1849	0.3517
20	30402333	rs62196406	1.062	8.31e-05	1.162	5.14e-05	0.8506	9.51e-06	0.9948	8.51e-06	ncRNA_intronic	FRG1BP	NA	NA	NA	NA	NA	NA	NA	NA	NA
14	27346327	rs8009161	0.7802	8.37e-05	0.761	0.0002188	0.4644	0.001715	0.4673	0.005231	ncRNA_intronic	LOC728755	27815533	A	C	0.270168	0.128	0.3862	0.0651	0.4851	0.3906
11	69989146	rs143881512	1.078	8.4e-05	0.9416	0.001038	0.4967	0.02059	0.7833	0.0009081	intronic	ANO1	69835252	TC	T	0.0692891	0.1319	0.0735	0.0083	0.1113	0.0409
1	24973945	rs11249220	-0.9765	8.42e-05	-1.058	6.47e-05	-0.4785	0.002725	-0.7605	0.0001286	intergenic	RUNX3;MIR4425	25300436	T	C	0.79353	0.7024	0.8271	0.9773	0.667	0.7454
1	24976267	rs2135754	-0.9765	8.42e-05	-1.058	6.47e-05	-0.5109	0.001486	-0.7605	0.0001286	intergenic	RUNX3;MIR4425	25302758	G	A	0.792732	0.7024	0.8242	0.9758	0.667	0.7454
13	21514594	rs2012875	0.9594	8.44e-05	1.142	1.51e-05	0.5277	0.002204	0.8025	5.17e-05	intronic	MICU2	22088733	C	T	0.34385	0.6944	0.3602	0.1112	0.1869	0.4468
20	30179901	rs1191020582	-1.249	8.44e-05	-1.317	5.99e-05	-1.043	3.4e-05	-1.241	6.95e-06	intergenic	IR4477A;LINC015	NA	NA	NA	NA	NA	NA	NA	NA	NA
3	42142681	rs9872588	1.287	8.45e-05	1.151	0.0007174	0.7858	0.002244	0.8208	0.003729	intronic	TRAK1	42184173	T	G	0.0595048	0.0506	0.0533	0.0242	0.1093	0.0695
6	62866770	rs59775654	-0.9	8.45e-05	-0.8068	0.0006129	-0.5186	0.0004754	-0.5978	0.0006506	intergenic	KHDRBS2;LGSN	63576675	T	C	0.698283	0.7778	0.7262	0.6853	0.5994	0.7157
1	200688310	rs7526482	-0.9139	8.5e-05	-0.9035	0.0002317	-0.3907	0.007949	-0.6419	0.0003248	ncRNA_intronic	DDX59-AS1	200657438	C	T	0.257188	0.2917	0.2233	0.1483	0.3827	0.2638
2	97151125	rs201532466	1.273	8.51e-05	1.185	0.0003772	0.8473	3.72e-05	1.011	4.6e-05	intronic	ANKRD36	NA	NA	NA	NA	NA	NA	NA	NA	NA
7	61065612	rs1171699091	1.12	8.52e-05	1.243	2.66e-05	0.8423	9.44e-05	0.8201	0.000822	intergenic	NONE;NONE	NA	NA	NA	NA	NA	NA	NA	NA	NA
10	118838988	rs56120810	1.997	8.58e-05	2.105	4.55e-05	1.466	0.001457	1.695	0.0004184	intergenic	CACUL1;NANOS1	120598500	T	C	0.0091853	0	0.0058	0	0.0388	0.0031
7	81663053	rs10280857	1.701	8.59e-05	1.724	9.31e-05	1.083	0.003307	1.334	0.0008578	ncRNA_intronic	LOC100128317	81292369	G	A	0.0650958	0.006	0.0504	0.1619	0.0348	0.0368
16	32409858	rs1284938622	1.671	8.59e-05	1.725	7.84e-05	0.7294	0.05384	1.22	0.002245	intergenic	90705;LOC10536	NA	NA	NA	NA	NA	NA	NA	NA	NA
4	29717122	rs192297812	2.439	8.6e-05	2.454	9.16e-05	1.639	0.001817	1.973	0.0006108	intergenic	.INC02472;PCDH	29718744	T	C	0.0189696	0.003	0.0144	0.031	0.0358	0.0051
18	57547111	rs73453635	2.312	8.61e-05	1.861	0.004267	1.478	0.002454	1.535	0.005197	UTR3	FECH	55214343	T	C	0.0385383	0.0724	0.0144	0.053	0.0219	0.0184
18	57550290	rs3760612	2.5	8.62e-05	2.046	0.003302	1.517	0.003868	1.728	0.002698	UTR3	FECH	55217522	A	T	0.0299521	0.0913	0.0159	0.0068	0.0199	0.0184
16	78959509	rs74032454	1.08	8.65e-05	1.06	0.0001909	0.6128	0.003521	0.6949	0.002995	intronic	WWOX	78993406	A	G	0.151757	0.3591	0.1268	0.0741	0.0606	0.1544
7	79174533	rs75636051	1.59	8.67e-05	1.578	0.0001445	0.9573	0.0008375	0.8639	0.009676	intronic	MAGI2	78803849	C	A	0.0898562	0.1468	0.0951	0.1626	0.0119	0.0092
11	69987721	rs61885142	1.076	8.73e-05	0.9398	0.001067	0.4764	0.02657	0.7819	0.000931	intronic	ANO1	69833827	T	A	0.0694888	0.1329	0.0735	0.0083	0.1113	0.0409
1	30361005	rs682703	1.105	8.74e-05	1.102	0.0001352	0.7945	0.0003398	0.9008	0.0002471	intergenic	.INC01648;MATN	30833852	C	G	0.872005	0.8413	0.8357	0.9085	0.8956	0.8558
3	78455996	rs9866630	0.8856	8.74e-05	0.9211	0.0001165	0.3107	0.04369	0.5007	0.007017	intergenic	.INC02077;ROBO	78505146	G	A	0.596446	0.8532	0.6254	0.4175	0.4771	0.6759
3	78456063	rs9866490	0.8856	8.74e-05	0.9211	0.0001165	0.2967	0.0531	0.4748	0.01002	intergenic	.INC02077;ROBO	78505213	C	A	0.596446	0.8532	0.6254	0.4175	0.4771	0.6759
11	112825364	rs10891455	1.045	8.75e-05	1.201	1.12e-05	0.5396	0.008069	0.7628	0.0005516	intergenic	02764;LOC10192	112696087	A	G	0.0978435	0.1508	0.062	0.0734	0.1074	0.092
7	123567555	rs12538107	1.108	8.76e-05	1.039	0.0004337	0.4968	0.01717	0.8571	0.0002248	intronic	NDUFA5	123207609	A	G	0.164736	0.0565	0.1023	0.233	0.1402	0.2536
11	47270686	rs1055510	-0.8664	8.76e-05	-1.017	2.16e-05	-0.5359	0.0004243	-0.6795	0.0001133	ncRNA_exonic	MADD-AS1	47292237	A	G	0.480431	0.7272	0.3372	0.5166	0.3072	0.4571
11	69990409	rs61885146	1.076	8.76e-05	0.9398	0.001071	0.5204	0.01547	0.8099	0.0006328	intronic	ANO1	69836515	G	A	0.0694888	0.1329	0.0735	0.0083	0.1113	0.0409
6	151648227	rs11155807	-1.266	8.81e-05	-1.399	9.74e-05	-0.8571	2.33e-05	-1.074	2.19e-05	intergenic	CCDC170;ESR1	151969362	T	A	0.16274	0.0298	0.1398	0.1853	0.1978	0.2495
3	36128450	rs7642528	0.8686	8.83e-05	0.9317	5.59e-05	0.4051	0.0147	0.5043	0.006561	intergenic	ARPP21;STAC	36169942	A	G	0.169329	0.0734	0.2075	0.1165	0.2992	0.1789
7	79171862	rs7799239	1.587	8.84e-05	1.575	0.0001463	0.9725	0.0007088	0.8955	0.00761	intronic	MAGI2	78801178	A	G	0.0900559	0.1478	0.0951	0.1626	0.0119	0.0092
18	57549535	rs201540657	2.493	8.84e-05	2.047	0.003289	1.509	0.004066	1.726	0.00275	UTR3	FECH	NA	NA	NA	NA	NA	NA	NA	NA	NA
1	143538429	rs78949682	1.137	8.85e-05	1.154	0.0001545	0.8448	5.41e-05	0.9339	0.0001066	intergenic	.SP1P5;RNVU1-1	NA	NA	NA	NA	NA	NA	NA	NA	NA
17	21656632	rs1458739994	1.095	8.85e-05	1.079	0.000208	0.7838	0.0001289	0.9506	5.23e-05	intergenic	.INC02693;KCNJ1	NA	NA	NA	NA	NA	NA	NA	NA	NA
8	138237886	rs4909409	0.7598	8.9e-05	0.8186	5.94e-05	0.4849	0.0006331	0.6765	3.79e-05	intergenic	FAM135B	139250129	A	C	0.362819	0.2917	0.3963	0.5416	0.3608	0.1728
18	57549090	rs78359676	2.494	8.93e-05	2.047	0.003307	1.507	0.0041	1.72	0.002861	UTR3	FECH	55216322	A	G	0.0301518	0.0923	0.0159	0.0068	0.0199	0.0184
9	41239266	rs1166759388	-1.172	8.99e-05	-1.21	0.00015	-0.6828	0.0005388	-0.8802	0.0001718	intergenic	1477A;LOC10798	NA	NA	NA	NA	NA	NA	NA	NA	NA
2	86866229	rs62146895	-1.083	9e-05	-1.046	0.0002961	-0.6203	0.001179	-0.6457	0.003557	ncRNA_intronic	ANAPC1P1	NA	NA	NA	NA	NA	NA	NA	NA	NA
13	21504274	rs2025242	0.9182	9e-05	1.099	1.53e-05	0.4824	0.004498	0.736	0.0001393	intronic	MICU2	22078413	C	T	0.352835	0.6617	0.3631	0.1838	0.1789	0.4346
10	51528427	rs34889204	0.8146	9.02e-05	0.7886	0.0002679	0.3181	0.03698	0.3156	0.06825	intronic	PRKG1	NA	NA	NA	NA	NA	NA	NA	NA	NA
1	124980048	rs1356379875	-1.122	9.05e-05	-1.132	0.0001684	-0.9212	2.81e-06	-1.039	8.36e-06	intergenic	NONE;NONE	NA	NA	NA	NA	NA	NA	NA	NA	NA
7	55637606	rs10248239	0.7687	9.05e-05	0.8043	0.0001066	0.3719	0.006306	0.4915	0.001791	intergenic	VOPP1;FKBP9P1	55705299	T	A	0.507188	0.3115	0.3444	0.823	0.5537	0.3497
3	42142869	rs9852952	1.281	9.08e-05	1.145	0.0007624	0.7792	0.002446	0.8146	0.004	intronic	TRAK1	42184361	C	T	0.0579073	0.0496	0.0519	0.0242	0.1054	0.0675
7	147980040	rs2888541	-0.8608	9.1e-05	-0.8003	0.0003967	-0.494	0.001038	-0.5664	0.00126	intronic	CNTNAP2	147677132	G	A	0.536941	0.7609	0.5216	0.562	0.4006	0.4233
16	33739449	rs74716198	-1.677	9.1e-05	-1.536	0.0003547	-1.028	1.18e-05	-1.184	4.46e-05	intergenic	C390705;ENPP7I	NA	NA	NA	NA	NA	NA	NA	NA	NA

12	128709229	rs12319911	-0.8425	9.17e-05	-0.7824	0.0004638	-0.5241	0.0002215	-0.5149	0.0018	intergenic	MEM132C;SLC15	129193774	G	T	0.275559	0.3621	0.304	0.1316	0.3211	0.3139
4	49563362	rs3207277	1.916	9.19e-05	1.989	5.97e-05	1.475	0.0002762	1.699	0.0001171	intergenic	CWH43;NONE	NA	NA	NA	NA	NA	NA	NA	NA	NA
11	81239461	rs35071790	1.139	9.19e-05	1.091	0.0002779	0.7215	0.001813	0.8877	0.0003121	intergenic	C02720;MIR4300	80950504	A	G	0.0595048	0.0476	0.0288	0.0401	0.0855	0.093
11	81242925	rs1013042	1.139	9.19e-05	1.091	0.0002779	0.7215	0.001813	0.8877	0.0003121	intergenic	C02720;MIR4300	80953968	C	A	0.0599042	0.0476	0.0288	0.0408	0.0855	0.0941
11	81246828	rs76126162	1.139	9.19e-05	1.091	0.0002779	0.7215	0.001813	0.8877	0.0003121	intergenic	C02720;MIR4300	80957871	C	G	0.0593051	0.0456	0.0288	0.0408	0.0855	0.093
10	16834053	rs79010915	-1.2	9.23e-05	-1.151	0.000367	-0.5581	0.002814	-0.6736	0.002456	intronic	CUBN	16876052	G	T	0.158546	0.2907	0.281	0.028	0.1243	0.1472
1	200623235	rs7521550	-0.911	9.3e-05	-0.8074	0.0007784	-0.3692	0.01209	-0.5674	0.00126	intergenic	KIF14;DDX59	200592363	C	G	0.254792	0.2827	0.2378	0.0779	0.4245	0.3027
1	200627217	rs10919991	-0.911	9.3e-05	-0.8074	0.0007784	-0.3779	0.01018	-0.5841	0.0009241	intergenic	KIF14;DDX59	200596345	C	A	0.252995	0.2837	0.2349	0.0726	0.4235	0.3027
9	62813926	rs1338876698	1.687	9.3e-05	1.704	0.0001115	1.091	0.003566	1.482	0.0001938	downstream	LINC01410	NA	NA	NA	NA	NA	NA	NA	NA	NA
4	94159632	rs73832505	1.278	9.32e-05	1.33	0.0001347	0.3459	0.1219	0.5411	0.03541	ncRNA_intronic	LOC101929210	95080783	T	C	0.126398	0.2034	0.1974	0.1316	0.0139	0.1053
18	8837442	rs8091169	0.7907	9.32e-05	0.7759	0.000304	0.3859	0.005742	0.4731	0.004281	intergenic	MTCL1;NDUFV2	8837440	A	G	0.461262	0.7579	0.3847	0.2882	0.4344	0.4714
20	29791098	rs1281742105	1.094	9.32e-05	1.216	2.97e-05	0.8277	7.17e-05	0.9265	8.84e-05	intergenic	AM242B;MIR447	NA	NA	NA	NA	NA	NA	NA	NA	NA
1	200621724	rs17450217	-0.9192	9.33e-05	-0.835	0.0005616	-0.3694	0.01222	-0.5716	0.001222	upstream	KIF14	200590852	A	T	0.254393	0.2847	0.2363	0.0779	0.4205	0.3037
10	74454517	rs10824183	0.9411	9.33e-05	0.8851	0.0003346	0.4991	0.002213	0.5899	0.001877	intronic	ADK	76214275	G	T	0.659345	0.3998	0.6772	0.8828	0.7565	0.5123
13	21567387	rs11148159	-0.8017	9.33e-05	-0.8599	9.06e-05	-0.5759	6.28e-05	-0.5903	0.0003284	intronic	MICU2	22141526	T	A	0.289936	0.3065	0.3256	0.0749	0.3976	0.4274
6	151618492	rs6904261	-1.171	9.35e-05	-1.382	4.32e-05	-0.9431	2.22e-06	-1.037	1.51e-05	UTR3	CCDC170	151939627	G	A	0.161741	0.0198	0.1513	0.1672	0.2147	0.2536
8	73231740	rs1833676	1.13	9.35e-05	1.119	0.0002398	0.8519	0.0002809	0.895	0.0004181	intergenic	SBSPON;C8orf89	74143975	C	T	0.0742812	0.1577	0.0634	0.0045	0.1074	0.0562
13	21589786	rs35010533	-0.799	9.35e-05	-0.9188	3.93e-05	-0.5071	0.0003354	-0.5643	0.0005773	intronic	MICU2	22163925	TAGTG	T	0.321486	0.3036	0.3429	0.2095	0.3877	0.408
19	14482529	rs10401146	-0.8889	9.36e-05	-1.019	4.37e-05	-0.5428	0.0004173	-0.5826	0.001291	intronic	GIPC1	14593341	G	A	0.634385	0.7986	0.6196	0.5885	0.6143	0.5583
20	25914616	rs62212267	-1.072	9.41e-05	-1.125	0.0001016	-0.8445	3.07e-05	-1.048	5.89e-06	intergenic	01926935;LINC0	NA	NA	NA	NA	NA	NA	NA	NA	NA
20	25914620	rs62212268	-1.072	9.41e-05	-1.125	0.0001016	-0.8203	5.16e-05	-1.013	1.19e-05	intergenic	01926935;LINC0	NA	NA	NA	NA	NA	NA	NA	NA	NA
11	48855576	rs1034311287	1.315	9.43e-05	1.163	0.001107	0.9799	0.0003591	1.067	0.0003911	intergenic	OR4A47;TRIM49	NA	NA	NA	NA	NA	NA	NA	NA	NA
8	7089760	rs149946349	-1.502	9.47e-05	-1.359	0.000443	-0.912	2.95e-05	-1.141	2.91e-05	intergenic	FA5;LOC101928	6947282	A	G	0.00858626	0.0188	0.0058	0.0144	0	0.001
9	10293685	rs10511541	1.931	9.47e-05	1.925	0.0001397	1.487	0.0004453	1.567	0.0005698	intronic	PTPRD	10293685	A	G	0.0632987	0.0456	0.2118	0.0197	0.006	0.0941
9	10293756	rs10511542	1.931	9.47e-05	1.925	0.0001397	1.487	0.0004453	1.567	0.0005698	intronic	PTPRD	10293756	C	T	0.0632987	0.0456	0.2118	0.0197	0.006	0.0941
9	10294095	rs16925743	1.931	9.47e-05	1.925	0.0001397	1.487	0.0004453	1.567	0.0005698	intronic	PTPRD	10294095	T	C	0.0632987	0.0456	0.2118	0.0197	0.006	0.0941
1	200687911	rs7526136	-0.9066	9.48e-05	-0.8959	0.0002571	-0.3796	0.0098	-0.6338	0.0003728	ncRNA_intronic	DDX59-AS1	200657039	C	T	0.257388	0.2927	0.2219	0.1467	0.3837	0.2658
7	123537261	rs35275680	1.13	9.54e-05	1.052	0.0005299	0.5973	0.006357	0.9322	0.0001099	UTR3	NDUFA5	123177315	T	A	0.0990415	0.0536	0.0836	0.0424	0.1402	0.1912
4	130576690	rs1531319	-0.8971	9.55e-05	-0.9643	8.7e-05	-0.5316	0.0007965	-0.9156	3.82e-06	intergenic	INC02479;SNHG2	131497845	C	T	0.207069	0.0962	0.3818	0.0552	0.4125	0.1912
7	79181713	rs2191812	1.552	9.55e-05	1.558	0.0001408	0.9333	0.0007969	0.8271	0.01101	intronic	MAGI2	78811029	T	C	0.102835	0.1518	0.098	0.2065	0.0119	0.0092
16	33042125	rs796228554	-1.234	9.56e-05	-1.127	0.0005541	-0.6692	0.0006933	-0.8311	0.0004308	intergenic	LC6A10P;TP53TG	NA	NA	NA	NA	NA	NA	NA	NA	NA
9	10284150	rs16925695	1.94	9.57e-05	1.937	0.0001395	1.489	0.0004763	1.571	0.0006075	intronic	PTPRD	10284150	G	T	0.0617013	0.0397	0.2118	0.0189	0.005	0.0941
7	79132720	rs57205028	1.512	9.58e-05	1.53	0.0001228	0.7873	0.004171	0.8523	0.00906	intronic	MAGI2	78762036	A	G	0.119609	0.1468	0.1023	0.2723	0.0119	0.0082
7	79132721	rs58800062	1.512	9.58e-05	1.53	0.0001228	0.7873	0.004171	0.8523	0.00906	intronic	MAGI2	78762037	C	A	0.119808	0.1468	0.1037	0.2723	0.0119	0.0082
11	69988369	rs61885143	1.065	9.58e-05	0.9461	0.0009816	0.4959	0.02067	0.7802	0.0009409	intronic	ANO1	69834475	T	A	0.0692891	0.1319	0.0735	0.0083	0.1113	0.0409
2	4196708	rs1866121	1.095	9.6e-05	1.077	0.0001734	0.6523	0.001883	0.6805	0.004688	intergenic	05373394;LINC0	4244298	C	T	0.770367	0.9881	0.8617	0.4561	0.8231	0.8517
6	166469188	rs117259014	1.598	9.6e-05	1.607	0.00012	0.8708	0.01061	1.092	0.003329	intronic	RPS6KA2	166882676	C	T	0.0161741	0	0.0317	0.003	0.0467	0.0082
17	14312129	rs76000899	1.653	9.63e-05	1.775	4.24e-05	1.082	0.001083	1.299	0.0003163	intronic	HS3ST3B1	14215446	T	C	0.058107	0.0903	0.0245	0.0356	0.0477	0.09
4	49563368	rs1356286708	1.908	9.66e-05	1.983	6.21e-05	1.434	0.0004346	1.69	0.0001243	intergenic	CWH43;NONE	NA	NA	NA	NA	NA	NA	NA	NA	NA
9	20319887	rs73649750	1.346	9.67e-05	1.362	9.83e-05	0.5144	0.0733	0.8452	0.007165	intergenic	SLC24A2;MLLT3	20319885	A	G	0.0625	0.001	0.0836	0.1362	0.0527	0.0215
10	74473238	rs10824190	0.9725	9.67e-05	0.8901	0.000505	0.4093	0.01181	0.5604	0.003449	intronic	ADK	76232996	T	C	0.477037	0.3641	0.6066	0.2799	0.7495	0.4877
23	2839192	rs5982877	0.7688	9.67e-05	0.7466	0.0002356	0.3409	0.01177	0.489	0.001391	intronic	GYG2	NA	NA	NA	NA	NA	NA	NA	NA	NA
1	161990551	rs77531925	1.6	9.7e-05	1.717	5.1e-05	1.055	0.0007707	1.397	0.0001122	intronic	OLFML2B	161960341	T	TA	0.0848642	0.126	0.0692	0.1377	0.0219	0.047
1	161990773	rs12046108	1.6	9.7e-05	1.717	5.1e-05	1.026	0.001028	1.334	0.0001895	intronic	OLFML2B	161960563	A	G	0.0850639	0.126	0.0692	0.1377	0.0229	0.047
1	161990909	rs12044683	1.6	9.7e-05	1.717	5.1e-05	1.026	0.001028	1.334	0.0001895	intronic	OLFML2B	161960699	C	T	0.0848642	0.126	0.0692	0.1377	0.0219	0.047
7	62336448	rs144061615	-1.087	9.7e-05	-1.079	0.0002526	-0.8109	4.01e-05	-0.9567	3.1e-05	intergenic	NONE;ZNF733P	NA	NA	NA	NA	NA	NA	NA	NA	NA
1	110182462	rs2484684	-0.8205	9.71e-05	-0.7386	0.0007133	-0.4658	0.001093	-0.6164	0.0003224	intronic	SLC6A17	110725084	C	T	0.577875	0.506	0.6643	0.5023	0.5954	0.6748
6	62859697	rs6930829	-0.8946	9.71e-05	-0.801	0.0006941	-0.5262	0.0003853	-0.5991	0.0006466	intergenic	KHDRBS2;LGSN	63569602	G	A	0.702077	0.7778	0.7406	0.6868	0.6044	0.7178
17	8722692	rs7219141	0.7888	9.73e-05	0.8121	0.0001343	0.3392	0.01426	0.49	0.002452	intergenic	MYH10;CCDC42	8626010	A	C	0.682308	0.631	0.7032	0.5923	0.7455	0.7771
10	74473309	rs7476181	0.9728	9.74e-05	0.8908	0.0005074	0.4068	0.01226	0.5599	0.003493	intronic	ADK	76233067	G	A	0.476837	0.3641	0.6066	0.2799	0.7495	0.4867
13	21574294	rs2185884	-0.7588	9.75e-05	-0.7951	0.0001085	-0.4245	0.002162	-0.5157	0.001203	intronic	MICU2	22148433	A	G	0.434704	0.3046	0.4395	0.4349	0.5268	0.4703
15	82781969	rs191364511	1.923	9.78e-05	2.12	1.95e-05	1.431	0.000919	1.581	0.0005815	intronic	FSD2	83450721	C	T	0.0147764	0	0.0173	0.0015	0.0487	0.0112
1	22216875	rs78416576	1.147	9.81e-05	1.273	3.08e-05	0.4826	0.04567	0.809	0.001872	intergenic	WNT4;MIR4418	22543368	C	T	0.0628994	0	0.1527	0.0053	0.1064	0.0971
11	81243026	rs1902862	1.134	9.82e-05	1.085	0.0002962	0.7165	0.001948	0.8825	0.0003369	intergenic	C02720;MIR4300	80954069	A	G	0.0599042	0.0476	0.0288	0.0408	0.0855	0.0941
11	81243115	rs1902863	1.134	9.82e-05	1.085	0.0002962	0.7165	0.001948	0.8825	0.0003369	intergenic	C02720;MIR4300	80954158	A	T	0.0599042	0.0476	0.0288	0.0408	0.0855	0.0941
7	81636937	rs9656518	1.375	9.84e-05	1.44	6.4e-05	0.7263	0.009281	1.093	0.0004595	ncRNA_intronic	LOC100128317	81266253	A	G	0.086262	0.0278	0.0605	0.1891	0.0497	0.0634
20	30175361	rs1250159068	-1.141	9.84e-05	-1.221	6.12e-05	-0.8896	4.96e-05	-0.8646	0.0005517	intergenic	IR4477A;LINC015	NA	NA	NA	NA	NA	NA	NA	NA	NA
9	10292763	rs1886000	1.925	9.87e-05	1.92	0.0001438	1.488	0.0004481	1.574	0.0005454	intronic	PTPRD	10292763	G	C	0.0626997	0.0456	0.2118	0.0197	0.006	0.091
9	10295010	rs117538995	1.925	9.87e-05	1.92	0.0001438	1.482	0.0004703	1.561	0.0006004	intronic	PTPRD	10295010	T	C	0.0626997	0.0446	0.2118	0.0197	0.005	0.093
1	200687916	rs7526265	-0.9033	9.93e-05	-0.8922	0.0002695	-0.381	0.009423	-0.6307	0.0003946	ncRNA_intronic	DDX59-AS1	200657044	G	A	0.257388	0.2927	0.2219	0.1467	0.3837	0.2658
20	29502985	rs1225233808	-1.087	9.93e-05	-1.132	0.0001144	-0.6827	0.001039	-0.7276	0.00236	intergenic	FRG1EP;FAM242	NA	NA	NA	NA	NA	NA	NA	NA	NA
6	62880792	rs9343308	-0.8833	9.94e-05	-0.8052	0.0006175	-0.5414	0.0002907	-0.6277	0.0003962	intergenic	KHDRBS2;LGSN	63590697	T	C	0.673722	0.7788	0.7233	0.5976	0.5924	0.7168
11	47319098	rs7120957	-0.8549	9.95e-05	-0.9701	3.67e-05	-0.5043	0.001065	-0.6218	0.0004383	intronic	MADD	47340649	A	G	0.479433	0.7262	0.3357	0.5136	0.3072	0.4581
6	143947515	rs2076682	0.9918	9.96e-05	0.9431	0.0004978	0.4263	0.01606	0.6557	0.001691	intronic	PLAGL1	144268652	G	A	0.17472	0.4375	0.1081	0.003	0.1272	0.2321
9	10291955	rs12553386	1.927	9.97e-05	1.921	0.0001454	1.458	0.000598	1.564	0.0005835	intronic	PTPRD	10291955	G	A	0.063099	0.0456	0.2118	0.0197	0.006	0.093
19	14483213	rs12974884	-0.879	9.98e-05	-1.008	4.51e-05	-0.5325	0.0005227	-0.5848	0.001203	intronic	GIPC1	14594025	C	T	0.632388	0.7976	0.6196	0.587	0.6074	0.5583
7	81624332	rs56347829	1.467	0.0001009	1.608	2.01e-05	0.8916	0.002673	1.248	0.0001438	ncRNA_intronic	LOC100128317	81253648	T	C	0.0694888	0.0218	0.0331	0.1762	0.0437	0.0266
17	43854842	rs34462326	1.004	0.0001012	1.17	1.44e-05	0.4178	0.03851	0.5652	0.01097	intronic	CD300LG	41932210	T	C	0.122404	0	0.1081	0.1831	0.1938	0.1033
1	191482297	rs139598681	-1.395	0.0001015	-1.569	0.0001068	-0.8726	7.79e-05	-1.116	3.97e-05	intergenic	INC01680;RGS1	NA	NA	NA	NA	NA	NA	NA	NA	NA
14	90938344	rs1286119	0.9397	0.0001016	1.045	3.53e-05	0.6456	0.0003762	0.6951	0.0008468	intronic	RPS6KA5	91404688	T	C	0.820088	0.9812	0.7406	0.7511	0.7893	0.8354
14	90943561	rs1286116	0.9397	0.0001016	1.045	3.53e-05	0.6509	0.0003465	0.6951	0.0008468	intronic	RPS6KA5	91409905	C	T	0.820288	0.9812	0.7378	0.7519	0.7893	0.8374
20	28784589	rs1210597027	-1.195	0.0001016	-1.235	0.0001631	-1.01	8.23e-07	-1.14	3.83e-06	intergenic	FRG1CP;FRG1DP	NA	NA	NA	NA	NA	NA	NA	NA	NA
8	40767160	rs7836926	1.01	0.0001019	1.097	4.62e-05	0.4642	0.01458	0.5685	0.008818	intronic	ZMAT4	40624679	G	A	0.30651	0.6171	0.2594	0.3328	0.0696	0.228
1	91632067	rs11588083	-0.8526	0.0001022	-0.8925	0.0001254	-0.5672	0.0001093	-0.6938	7.07e-05	intergenic	CDC7;TGFBR3	92097624	C	G	0.591653	0.5565	0.5533	0.6293	0.7048	0.4877
13	21514222	rs1041021	0.9443	0.0001025	1.13	1.77e-05	0.5208	0.002456	0.7942	5.89e-05	intronic	MICU2	22088361	G	A	0.34365	0.6944	0.3602	0.1104	0.1869	0.4468
4	116186016	rs10001754	1.125	0.0001028	1.221	4.08e-05	0.7316	0.0007566	0.902	0.0003205	intergenic	NDST4;MIR1973	117107172	C	T	0.234425	0.0903	0.183	0.5734	0.1064	0.093
4	116186057	rs10024106	1.125	0.0001028	1.221	4.08e-05	0.7316	0.0007566	0.902	0.0003205	intergenic	NDST4;MIR1973	117107213	A	G	0.234425	0.0903	0.183	0.5734	0.1064	0.093
4	116189170	rs1404982	1.125	0.0001028	1.221	4.08e-05	0.7353	0.0007125	0.9095	0.0002877	intergenic	NDST4;MIR1973	117110326	G	C	0.235024	0.0903	0.183	0.5756	0.1064	0.093
4	116189174	rs1404983	1.125	0.0001028	1.221	4.08e-05	0.7353	0.0007125	0.9095	0.0002877	intergenic	NDST4;MIR1973	117110330	A	G	0.235024	0.0903	0.183	0.5756	0.1064	0.093
4	116189220	rs1404984	1.125	0.0001028	1.221	4.08e-05	0.7353	0.0007125	0.9095	0.0002877	intergenic	NDST4;MIR1973	117110376	A	G	0.235024	0.0903	0.183	0.5756	0.1064	0.093
4	116194376	rs17048369	1.125	0.0001028	1.221	4.08e-05	0.7285	0.0007906	0.9309	0.0001977	intergenic	NDST4;MIR1973	117115532	T	A	0.234225	0.0903	0.1844	0.5719	0.1064	0.093
4	116194389	rs72905580	1.125	0.0001028	1.221	4.08e-05	0.7285	0.0007906	0.9309	0.0001977	intergenic	NDST4;MIR1973	117115545	T	G	0.234225	0.0903	0.1844	0.5719	0.1064	0.093
4	116200655	rs6827033	1.125	0.0001028	1.221	4.08e-05	0.7285	0.0007906	0.9309	0.0001977	intergenic	NDST4;MIR1973	117121811	C	T	0.233227	0.0903	0.183	0.5688	0.1064	0.093
4	116190107	rs5861268	1.118	0.0001029	1.236	3.14e-05	0.7283	0.0007604	0.8992	0.0003189	intergenic	NDST4;MIR1973	117111263	A	AT	0.75	0.9097	0.8156	0.3684	0.8936	0.907
16	33042131	rs796484268	-1.223	0.0001032	-1.121	0.0005754	-0.7087	0.0003233	-0.8101	0.0005507	intergenic	LC6A10P;TP53TG	NA	NA	NA	NA	NA	NA	NA	NA	NA
4	29747878	rs6816461	1.919	0.0001037	1.971	8.44e-05	1.612	0.0001175	1.53	0.0008469	intergenic	INC02472;PCDH	29749500	C	A	0.0427316	0	0.0331	0.0908	0.0567	0.0143
4	29748443	rs6822726	1.919	0.0001037	1.971	8.44e-05	1.612	0.0001175	1.53	0.0008469	intergenic	INC02472;PCDH	29750065	G	A	0.0427316	0	0.0331	0.0908	0.0567	0.0143
4	116187647	rs10005583	1.125	0.0001037	1.239	3.31e-05	0.7487	0.0006123	0.922	0.0002573	intergenic	NDST4;MIR1973	117108803	C	T	0.234225	0.0903	0.183	0.5726	0.1064	0.093
4	116193092	rs6814137	1.125	0.0001037	1.239	3.31e-05	0.7488	0.0006089	0.9576	0.0001443	intergenic	NDST4;MIR1973	117114248	A	G	0.234225	0.0903	0.1844	0.5719	0.1064	0.093
4	116195518	rs12506192	1.125	0.0001037	1.239	3.31e-05	0.7452	0.0006431	0.9512	0.0001575	intergenic	NDST4;MIR1973	117116674	G	A	0.234225	0.0903	0.1844	0.5719	0.1064	0.093
4	116202093	rs6533960	1.125	0.0001037	1.239	3.31e-05	0.7452	0.0006431	0.9512	0.0001575	intergenic	NDST4;MIR1973	117123249	C	T	0.234225	0.0903	0.1844	0.5719	0.1064	0.093
11	69984464	rs61885140	1.003	0.0001038	0.9128	0.000692	0.538	0.007486	0.7532	0.0007504	intergenic	FGF3;ANO1	69830570	C	T	0.0814696	0.1825	0.0793	0.0083	0.1153	0.0429
3	85321244	rs818214	-1.47	0.000104	-1.637	0.000147	-0.5292	0.006777	-1.133	5.26e-05	intronic	CADM2	85370394	T	C	0.199481	0.1825	0.0836	0.3926	0.0994	0.1411
13	21517014	rs1199950	-0.7496	0.0001045	-0.8289	5.96e-05	-0.4311	0.001873	-0.5298	0.0008979	intronic	MICU2	22091153	A	G	0.451877	0.3056	0.451	0.4917	0.5358	0.4632
13	21466609	rs1886866	-0.819	0.000105	-0.975	2.79e-05	-0.644	1.13e-05	-0.6574	0.0001158	intergenic	ZDHHC20;MICU2	22040748	G	T	0.279952	0.255	0.3127	0.1142	0.3738	0.41
17	57342002	rs4573986	1.517	0.000105	1.613	5.36e-05	0.4255	0.1852	0.8139	0.02105	intronic	MSI2	55419363	G	A	0.0217652	0	0.0216	0.0008	0.0716	0.0215
3	85212235	rs9813787	-1.558	0.0001052	-1.611	0.0002532	-0.5712	0.00506	-1.205	3.88e-05	intronic	CADM2	85261385	A	T	0.251797	0.1875	0.0965	0.584	0.1004	0.135
1	143540806	rs143547868	-2.436	0.0001056	-2.409	0.000189	-2.048	0.0002166	-2.325	5.34e-05	intergenic	SP1P5;RNVU1-1	NA	NA	NA	NA	NA	NA	NA	NA	NA
1	200626241	rs10399790	-0.9075	0.0001072	-0.8181	0.00068	-0.3836	0.009384	-0.5668	0.001323	intergenic	KIF14;DDX59	200595369	A	T	0.253195	0.2837	0.2349	0.0734	0.4235	0.3027

7	123537075	rs34418989	1.09	0.0001075	1.039	0.0004337	0.4912	0.01839	0.8493	0.0002535	UTR3	NDUFA5	123177129	A	T	0.164137	0.0556	0.1009	0.2315	0.1412	0.2536
14	90961810	rs1286095	0.9274	0.0001075	1.006	5.8e-05	0.6301	0.000485	0.7109	0.0006265	intronic	RPS6KA5	91428154	A	G	0.814097	0.9812	0.7392	0.7284	0.7903	0.8354
11	47327151	rs3781622	-0.8398	0.0001085	-0.9751	3.12e-05	-0.5527	0.0002731	-0.6828	9.21e-05	intronic	MADD	47348702	T	C	0.483427	0.7262	0.3386	0.525	0.3072	0.4611
13	21608434	rs60074257	-0.8154	0.0001085	-0.923	5.28e-05	-0.5407	0.0002111	-0.5886	0.0004946	intergenic	MICU2;FGF9	22182573	G	A	0.253594	0.3036	0.3256	0.0234	0.3628	0.3497
3	85236934	rs9681960	-1.491	0.0001095	-1.679	0.0001457	-0.6221	0.002454	-1.262	1.62e-05	intronic	CADM2	85286084	T	A	0.244209	0.1855	0.0965	0.5575	0.0994	0.135
12	11316857	rs551119637	1.674	0.0001097	1.627	0.0002495	0.7721	0.04054	1.123	0.005522	intergenic	PRB4;PRB1	11469791	A	G	0.0123802	0	0.0187	0.0015	0.0408	0.0061
15	91395484	rs12441301	1.225	0.0001103	1.379	2.82e-05	0.7446	0.001153	0.8545	0.001186	intergenic	SV2B;CRAT37	91938714	A	G	0.136981	0.2937	0.0994	0.1301	0.0755	0.0746
3	85179495	rs1376936	-1.544	0.0001105	-1.615	0.0002399	-0.6531	0.001849	-1.311	1.73e-05	intronic	CADM2	85228645	C	G	0.254193	0.1875	0.098	0.5825	0.0974	0.1513
4	116224190	rs10022336	1.149	0.0001107	1.258	4.04e-05	0.7647	0.0004585	0.9977	0.0001019	intergenic	NDST4;MIR1973	117145346	T	A	0.222644	0.0903	0.1686	0.4365	0.1054	0.229
6	134756060	rs9402642	-0.774	0.0001116	-0.8585	5.71e-05	-0.3407	0.01276	-0.5556	0.0006471	intergenic	101928304;ALDH	135077198	C	T	0.54373	0.5962	0.5778	0.6233	0.4463	0.4581
7	123519096	rs71574748	1.106	0.0001122	1.04	0.0005299	0.5566	0.009993	0.894	0.0001701	intronic	IQUB	123159150	A	G	0.0978435	0.0536	0.0821	0.0431	0.1421	0.183
3	695650	rs2628010	-0.9229	0.000113	-0.9035	0.0002759	-0.7157	8.85e-06	-0.8785	5.79e-06	ncRNA_intronic	LINC01266	737334	T	A	0.582468	0.6101	0.6484	0.3601	0.7525	0.6329
1	200693087	rs7542210	-0.9011	0.0001132	-0.8646	0.0003791	-0.3929	0.007689	-0.6432	0.0003184	ncRNA_intronic	DDX59-AS1	200662215	C	T	0.250799	0.2927	0.2233	0.1483	0.3787	0.2342
3	85328521	rs818222	-1.467	0.0001138	-1.618	0.0001819	-0.5194	0.00819	-1.12	6.84e-05	intronic	CADM2	85377671	T	C	0.199681	0.1825	0.0836	0.3949	0.1004	0.138
13	19914072	rs1413477	0.8712	0.0001138	0.9362	7.63e-05	0.4389	0.007288	0.6873	0.0003411	intergenic	ZMYM5;ZMYM2	20488212	A	G	0.682109	0.8313	0.7176	0.4266	0.7783	0.7495
13	19914381	rs4769917	0.8712	0.0001138	0.9362	7.63e-05	0.4507	0.00588	0.6873	0.0003411	intergenic	ZMYM5;ZMYM2	20488521	T	C	0.680711	0.8313	0.7176	0.4213	0.7783	0.7495
13	19915737	rs7997980	0.8712	0.0001138	0.9362	7.63e-05	0.4389	0.007288	0.6873	0.0003411	intergenic	ZMYM5;ZMYM2	20489877	T	C	0.680312	0.8313	0.7161	0.4191	0.7783	0.7515
3	36072326	rs6788182	0.8262	0.000114	0.9156	4.71e-05	0.3599	0.02583	0.5031	0.005375	intergenic	ARPP21;STAC	36113818	G	T	0.167931	0.0714	0.2075	0.1165	0.2982	0.1748
3	85159417	rs12487792	-1.539	0.0001143	-1.624	0.0002197	-0.645	0.002007	-1.32	1.49e-05	intronic	CADM2	85208567	A	T	0.252196	0.1865	0.0951	0.5772	0.0974	0.1513
13	21473837	rs7982473	0.9239	0.0001144	1.131	1.47e-05	0.5384	0.001522	0.774	6.96e-05	intergenic	ZDHHC20;MICU2	22047976	C	G	0.365415	0.7391	0.379	0.1331	0.2078	0.4468
17	12462812	rs1350704	0.7669	0.0001148	0.875	4.49e-05	0.3889	0.005443	0.4863	0.002678	intergenic	IAP2K4;LINC0067	12366129	G	A	0.614617	0.5446	0.4582	0.6815	0.6004	0.7219
15	30492974	rs200566609	-1.045	0.0001153	-1.015	0.0003398	-0.879	4.92e-06	-0.959	1.7e-05	intergenic	0288203;LOC100	NA	NA	NA	NA	NA	NA	NA	NA	NA
6	135187715	rs17720864	2.568	0.0001155	2.701	5.33e-05	1.724	0.003978	2.233	0.0002848	intronic	MYB	135508853	C	T	0.014976	0.0486	0.0029	0.0008	0.0209	0.002
6	134749639	rs7749965	-0.7674	0.0001157	-0.8541	5.65e-05	-0.3426	0.01144	-0.5527	0.0006355	intergenic	101928304;ALDH	135070777	T	C	0.517173	0.5962	0.5764	0.5174	0.4513	0.4611
11	69990617	rs61885147	1.062	0.000116	0.9183	0.001524	0.4253	0.04981	0.7194	0.002463	intronic	ANO1	69836723	C	T	0.0642971	0.1141	0.0735	0.0061	0.1113	0.0368
20	29027918	rs79236426	-1.346	0.000116	-1.292	0.0003649	-0.9843	7.99e-06	-1.093	3.81e-05	intergenic	FRG1CP;FRG1DP	NA	NA	NA	NA	NA	NA	NA	NA	NA
4	116184243	rs1032943	1.118	0.0001162	1.233	3.71e-05	0.7505	0.0006065	0.9169	0.0002816	intergenic	NDST4;MIR1973	117105399	A	G	0.234625	0.0903	0.183	0.5741	0.1064	0.093
17	32135553	rs796177255	1.065	0.0001165	0.9201	0.001563	0.7664	0.0001619	0.978	2.55e-05	intergenic	C37B;LOC105371	NA	NA	NA	NA	NA	NA	NA	NA	NA
12	3933197	rs11062924	0.9593	0.0001166	0.9246	0.0004258	0.5293	0.004027	0.5619	0.008771	intergenic	ARP11;CCND2-AS	4042363	G	T	0.162939	0.1359	0.1138	0.2163	0.16	0.1564
8	40732872	rs6474273	0.8217	0.0001167	0.8878	5.53e-05	0.349	0.02373	0.4264	0.01604	intronic	ZMAT4	40590391	G	T	0.57508	0.4266	0.634	0.444	0.7952	0.637
1	24974879	rs12130556	-0.9423	0.0001174	-1.035	7.85e-05	-0.4731	0.002823	-0.7126	0.0002773	intergenic	RUNX3;MIR4425	25301370	G	A	0.769968	0.7024	0.8098	0.8971	0.667	0.7454
13	21599043	rs1041020	0.9072	0.0001174	1.083	2.08e-05	0.5472	0.0009212	0.7745	5.13e-05	intronic	MICU2	22173182	C	T	0.384185	0.6885	0.3862	0.2526	0.1889	0.4479
20	28792997	rs1183170692	-1.075	0.0001175	-1.195	5.13e-05	-0.7556	0.0001398	-0.7239	0.001599	intergenic	FRG1CP;FRG1DP	NA	NA	NA	NA	NA	NA	NA	NA	NA
22	16894430	rs73392010	1.09	0.0001176	1.173	9.42e-05	0.5812	0.002643	0.702	0.001805	intergenic	HSFY1P1;GAB4	NA	NA	NA	NA	NA	NA	NA	NA	NA
19	14491036	rs12463167	-0.7813	0.0001181	-0.8499	8.06e-05	-0.4544	0.001212	-0.5531	0.0008093	intronic	GIPC1	14601848	A	G	0.510383	0.2738	0.5	0.5651	0.505	0.6933
11	47316169	rs10555583	-0.8379	0.0001184	-0.9341	6.31e-05	-0.5074	0.0008077	-0.6187	0.0003916	intronic	MADD	47337720	ACG	A	0.479034	0.7272	0.3357	0.5136	0.3072	0.455
3	36125035	rs954694	0.8178	0.0001185	0.8982	5.55e-05	0.3701	0.02131	0.5031	0.005147	intergenic	ARPP21;STAC	36166527	G	C	0.169129	0.0734	0.2075	0.1165	0.2982	0.1789
3	36139314	rs7650036	0.8178	0.0001185	0.8982	5.55e-05	0.3701	0.02131	0.5031	0.005147	intergenic	ARPP21;STAC	36180806	T	A	0.169529	0.0734	0.2075	0.1165	0.2992	0.18
3	46528891	rs9848284	0.7463	0.0001186	0.5752	0.004521	0.4239	0.002143	0.4794	0.002242	intronic	LRRC2	46570381	T	C	0.534345	0.6091	0.5591	0.3472	0.5845	0.6411
1	161990711	rs12041196	1.571	0.0001187	1.717	5.1e-05	1.019	0.001101	1.325	0.0002068	intronic	OLFML2B	161960501	G	T	0.0844649	0.124	0.0692	0.1377	0.0219	0.047
1	161993041	rs12568584	1.571	0.0001187	1.717	5.1e-05	1.019	0.001101	1.325	0.0002068	intronic	OLFML2B	161962831	C	T	0.0850639	0.124	0.0692	0.1399	0.0219	0.047
10	125633253	rs9422902	1.544	0.0001188	1.649	6.63e-05	0.8737	0.00316	1.023	0.00221	intronic	TEX36	127321822	A	G	0.0978435	0.122	0.0605	0.059	0.0437	0.2076
10	125633297	rs2046295	1.544	0.0001188	1.649	6.63e-05	0.8737	0.00316	1.023	0.00221	intronic	TEX36	127321866	A	G	0.0976438	0.122	0.0605	0.0582	0.0437	0.2076
7	51653338	rs60400218	1.584	0.0001189	1.632	0.0001191	0.8367	0.01502	1.026	0.008091	intergenic	07986794;POM1	51721034	AT	TT,A	0259585,0.0636	0,0	0,0.0591	0.0098,0.1384	0,0.0527	0,0.0429
23	3363478	rs806625	0.7001	0.0001189	0.6983	0.0002754	0.4431	0.001261	0.4914	0.001538	intergenic	MXRA5;SNORA48	NA	NA	NA	NA	NA	NA	NA	NA	NA
9	41239130	rs1311778547	1.116	0.0001193	1.15	0.0001652	0.8518	0.0001071	1.031	2.79e-05	intergenic	4477A;LOC10798	NA	NA	NA	NA	NA	NA	NA	NA	NA
22	11567016	rs1208579097	1.741	0.0001197	1.596	0.0004348	0.9475	3.54e-05	1.113	0.0001017	intergenic	51FP;LOC102723	NA	NA	NA	NA	NA	NA	NA	NA	NA
11	45723148	rs10742765	1.19	0.0001198	1.335	2.62e-05	0.7639	0.001422	0.8388	0.001698	ncRNA_intronic	LOC100507384	45744698	C	T	0.126398	0.1855	0.196	0.0772	0.0885	0.1217
17	21412546	rs72846655	-1.157	0.00012	-1.226	0.000101	-0.9215	0.0001017	-1.067	4.73e-05	intronic	KCNJ12	NA	NA	NA	NA	NA	NA	NA	NA	NA
13	21502144	rs139682127	0.8991	0.0001201	1.079	2.07e-05	0.4762	0.004511	0.7358	0.0001215	intronic	MICU2	22076283	TTTC	T	0.358826	0.6597	0.3746	0.1853	0.1988	0.4366
6	151646800	rs115713943	-1.241	0.000121	-1.399	9.74e-05	-0.8495	2.76e-05	-1.062	2.72e-05	intergenic	CCDC170;ESR1	151967935	A	G	0.162939	0.0298	0.1398	0.1853	0.1988	0.2495
7	51639487	rs2191255	1.682	0.000121	1.704	0.0001473	0.9136	0.01004	1.212	0.003461	intergenic	07986794;POM1	51707183	C	T	0.0613019	0	0.0562	0.1263	0.0527	0.0491
3	46528231	rs6791703	0.7457	0.0001221	0.5741	0.004661	0.4274	0.001971	0.4772	0.0024	intronic	LRRC2	46569721	G	T	0.532149	0.6091	0.5591	0.3389	0.5845	0.6411
3	46528487	rs9827559	0.7457	0.0001221	0.5741	0.004661	0.4194	0.002444	0.4772	0.0024	intronic	LRRC2	46569977	C	A	0.526558	0.6091	0.5562	0.3207	0.5825	0.6411
1	75391533	rs1249825	-0.9003	0.0001224	-1.035	3.75e-05	-0.5455	0.0007806	-0.8975	5.96e-06	intronic	SLC44A5	75857218	C	A	0.737021	0.9722	0.6354	0.8684	0.5398	0.592
9	10326236	rs10756025	0.8308	0.0001224	0.8212	0.0002536	0.6161	9.66e-05	0.6675	0.0001812	intronic	PTPRD	10326236	T	C	0.366613	0.2579	0.4107	0.5492	0.2097	0.362
13	21516168	rs34979708	0.8987	0.0001224	1.077	2.11e-05	0.4945	0.003456	0.7507	0.0001145	intronic	MICU2	22090307	T	C	0.36881	0.6954	0.3761	0.1861	0.1988	0.4489
22	11610038	rs71237385	1.125	0.0001225	1.147	0.000173	0.6497	0.003479	0.6773	0.006901	intergenic	51FP;LOC102723	NA	NA	NA	NA	NA	NA	NA	NA	NA
2	164656555	rs34271465	0.7734	0.0001227	0.842	7.76e-05	0.5247	0.0003574	0.6741	5.62e-05	intergenic	GRB14;COBLL1	165513065	CA	C	0.396965	0.0962	0.2594	0.7988	0.4423	0.2147
2	164656581	rs10195252	0.7734	0.0001227	0.842	7.76e-05	0.5247	0.0003574	0.6741	5.62e-05	intergenic	GRB14;COBLL1	165513091	T	C	0.396965	0.0962	0.2594	0.7988	0.4423	0.2147
13	21488120	rs9552435	0.8966	0.0001228	1.074	2.15e-05	0.4601	0.006234	0.6961	0.0002855	intergenic	ZDHHC20;MICU2	22062259	C	T	0.361821	0.6607	0.3761	0.1868	0.1978	0.4489
3	85241284	rs9866167	-1.467	0.000123	-1.64	0.0001799	-0.5777	0.00414	-1.229	2.41e-05	intronic	CADM2	85290434	T	G	0.251198	0.1855	0.0965	0.5832	0.1004	0.135
17	21835781	rs1422997524	-1.03	0.0001233	-1.21	2.56e-05	-0.7917	4.07e-05	-0.7571	0.0006272	intergenic	KCNJ18;UBBP4	NA	NA	NA	NA	NA	NA	NA	NA	NA
11	71674159	rs192400155	1.17	0.0001234	1.23	9.91e-05	0.8319	0.0004488	1.172	9.66e-06	intergenic	RTAP5-11;ALG1L9	NA	NA	NA	NA	NA	NA	NA	NA	NA
18	26765519	rs58701382	-0.787	0.0001255	-0.8593	8.83e-05	-0.3665	0.007121	-0.3747	0.01672	intergenic	PCAT18;AQP4	24345483	T	C	0.464657	0.3829	0.3833	0.652	0.4145	0.4049
3	36067656	rs1451793	0.779	0.0001256	0.873	5.45e-05	0.3377	0.02314	0.4864	0.004002	intergenic	ARPP21;STAC	36109148	C	T	0.600639	0.8859	0.5893	0.295	0.5984	0.7301
1	3975069	rs10799224	0.9389	0.0001259	1.128	1.46e-05	0.4548	0.0108	0.6961	0.0007814	intergenic	NC01346;LINC017	4035129	G	A	0.838059	0.996	0.8602	0.8918	0.6948	0.7342
3	36067492	rs5847908	0.7826	0.000126	0.8731	5.55e-05	0.3347	0.02448	0.4835	0.00427	intergenic	ARPP21;STAC	36108984	CA	C	0.600439	0.8859	0.5893	0.295	0.5984	0.729
6	15327554	rs10589143	1.447	0.0001263	1.426	0.0002367	1.05	0.0008076	1.394	4.25e-05	intronic	JARID2	NA	NA	NA	NA	NA	NA	NA	NA	NA
13	21493542	rs3818314	0.8813	0.0001263	1.068	2.15e-05	0.4721	0.004395	0.7537	7.44e-05	intronic	MICU2	22067681	T	C	0.413139	0.6607	0.3991	0.3676	0.2008	0.4479
4	94137160	rs74878305	1.254	0.0001266	1.308	0.0001725	0.348	0.1219	0.5449	0.03539	ncRNA_intronic	LOC101929210	95058311	T	G	0.126997	0.2044	0.1974	0.1331	0.0139	0.1053
17	75555119	rs62089183	0.7718	0.0001267	0.7218	0.0006222	0.4026	0.004058	0.6004	0.0002575	intronic	LLGL2	73551200	G	A	0.404952	0.4683	0.3746	0.1044	0.5606	0.6074
3	85171216	rs58013523	-1.529	0.0001268	-1.615	0.0002399	-0.6297	0.002583	-1.304	1.91e-05	intronic	CADM2	85220366	G	A	0.253794	0.1865	0.098	0.5817	0.0974	0.1513
6	151631510	rs66585063	-1.163	0.0001268	-1.325	8.02e-05	-0.8454	1.52e-05	-1.01	2.5e-05	intergenic	CCDC170;ESR1	151952645	G	T	0.169928	0.0268	0.1542	0.1959	0.2127	0.2495
3	85214194	rs9882148	-1.464	0.0001269	-1.65	0.0001648	-0.5946	0.003151	-1.248	1.77e-05	intronic	CADM2	85263344	T	G	0.251198	0.1875	0.0965	0.5817	0.1004	0.135
3	85214266	rs9882296	-1.464	0.0001269	-1.65	0.0001648	-0.5946	0.003151	-1.248	1.77e-05	intronic	CADM2	85263416	T	C	0.251198	0.1875	0.0965	0.5817	0.1004	0.135
3	85215861	rs11127883	-1.464	0.0001269	-1.65	0.0001648	-0.5946	0.003151	-1.248	1.77e-05	intronic	CADM2	85265011	T	C	0.251198	0.1875	0.0965	0.5817	0.1004	0.135
3	85258811	rs7635613	-1.464	0.0001269	-1.65	0.0001648	-0.5946	0.003151	-1.248	1.77e-05	intronic	CADM2	85307961	G	A	0.249002	0.1865	0.0951	0.5756	0.0994	0.135
3	85259434	rs6799730	-1.47	0.0001269	-1.645	0.0001839	-0.6054	0.003193	-1.241	2.18e-05	intronic	CADM2	85308584	A	T	0.244409	0.1865	0.098	0.5575	0.0984	0.135
17	72953421	rs8080025	2.987	0.0001276	3.051	0.0001998	1.496	0.01192	2.063	0.002481	intronic	SLC39A11	70949560	T	A	0.0279553	0.0526	0.0101	0.0522	0	0.0112
1	75398319	rs6691590	-0.8775	0.0001278	-0.978	6.78e-05	-0.5374	0.0008254	-0.8738	7.08e-06	intronic	SLC44A5	75864004	C	G	0.260783	0.0288	0.3602	0.1263	0.4602	0.4059
11	47303115	rs4752824	-0.8363	0.0001278	-0.9501	4.79e-05	-0.5292	0.0004759	-0.6678	0.0001322	intronic	MADD	47324666	T	A	0.479233	0.7262	0.3357	0.5129	0.3072	0.4581
22	44121400	rs5764544	0.7413	0.0001282	0.7154	0.0003322	0.4632	0.001019	0.6644	4.79e-05	intronic	PARVB	44517280	C	T	0.444489	0.2976	0.4697	0.7352	0.3678	0.2638
1	200626145	rs10399615	-0.8944	0.0001283	-0.8074	0.0007784	-0.3573	0.01528	-0.5579	0.00154	intergenic	KIF14;DDX59	200595273	G	A	0.254593	0.2837	0.2363	0.0779	0.4235	0.3027
1	200627076	rs10919990	-0.8944	0.0001283	-0.8074	0.0007784	-0.3623	0.01388	-0.5579	0.00154	intergenic	KIF14;DDX59	200596204	G	A	0.258786	0.2837	0.2363	0.0953	0.4235	0.3006
1	200627849	rs7529962	-0.8944	0.0001283	-0.8074	0.0007784	-0.3623	0.01388	-0.5579	0.00154	intergenic	KIF14;DDX59	200596977	T	C	0.264776	0.2837	0.2378	0.1135	0.4235	0.3057
5	176877398	rs2304517	1.004	0.0001283	0.9984	0.0002171	0.3577	0.0593	0.5348	0.01264	intronic	UNC5A	176304399	A	G	0.853035	0.6627	0.8559	0.9508	0.9145	0.8517
4	116190215	rs1027792	1.1	0.0001291	1.23	3.47e-05	0.8195	0.0001435	0.9481	0.0001284	intergenic	NDST4;MIR1973	117111371	G	C	0.6877	0.9087	0.7939	0.1906	0.8857	0.8528
1	200695174	rs7526031	-0.892	0.0001294	-0.8551	0.0004292	-0.383	0.009221	-0.6315	0.0003929	downstream	DDX59-AS1	200664302	A	C	0.250399	0.2927	0.2205	0.1467	0.3787	0.2362
4	116185060	rs6856663	1.105	0.0001294	1.221	4.08e-05	0.7249	0.0008346	0.8926	0.0003644	intergenic	NDST4;MIR1973	117106216	G	A	0.234425	0.0903	0.183	0.5734	0.1064	0.093
7	123611694	rs7797819	1.051	0.0001295	0.9683	0.0008806	0.5594	0.006035	0.8298	0.0001995	intronic	ASB15	123251748	G	A	0.144968	0.0942	0.098	0.1672	0.1451	0.2004
13	21588517	rs9552455	0.9249	0.0001296	1.107	2.35e-05	0.501	0.003366	0.7661	0.0001068	intronic	MICU2	22162656	T	C	0.342851	0.6954	0.3617	0.1044	0.1889	0.4468
6	16255578	rs9383141	2.192	0.0001308	1.91	0.001765	1.109	0.0107	1.224	0.0167	intronic	GMPR	16255809	A	T	0.0491214	0.0685	0.0043	0.1271	0.003	0.0031
3	85229420	rs1839938	-1.466	0.0001309	-1.654	0.0001683	-0.6155	0.002711	-1.249	1.89e-05	intronic	CADM2	85278570	T	C	0.244409	0.1855	0.0965	0.5582	0.0994	0.135
18	58714800	rs59570228	-0.8947	0.0001309	-0.8736	0.0003623	-0.4596	0.001596	-0.8221	8.26e-06	intronic	MALT1	56382032	GGATA	G	0.493411	0.6131	0.6095	0.0915	0.7386	0.5787
12	97932388	rs77222855	2.799	0.0001314	2.996	4.6e-05	1.464	0.01789	2.076	0.001894	intergenic	FAH1B2P2;MIR4	98326166	A	C	0.0259585	0	0.0288	0.0703	0.0169	0
1	200624065	rs2808254	-0.8986	0.0001327	-0.8037	0.000874	-0.3561	0.01553	-0.5607	0.001486	intergenic	KIF14;DDX59	200593193	T	C	0.721446	0.7173	0.7594	0.8328	0.5755	0.6984
3	85189880	rs112349103	-1.46	0.0001331	-1.645	0.0001731	-0.586	0.003634	-1.239	2.06e-05	intronic	CADM2	85239030	TTATCAG	T	0.250599	0.1875	0.098	0.5809	0.1004	0.1319
10	3185212	rs113657344	1.188	0.0001332	1.28	9.05e-05	0.7647	0.000816	0.7884	0.00226	intergenic	RM1;LOC105376	3227404	G	GCCCA,GCCCC	.132987,0.10243	0.0407,0.1726	0.0548,0.0749	0.4085,0.0794	0.0328,0.0596	0.0143,0.1247

16	36095793	rs1486318784	1.894	0.0001333	1.827	0.000414	1.167	0.0119	1.375	0.004386	intergenic	LINC02167;NONE	NA	NA	NA	NA	NA	NA	NA	NA	NA
3	722897	rs9873686	-0.9027	0.0001335	-0.9111	0.0002455	-0.6467	4.52e-05	-0.7756	4.34e-05	ncRNA_intronic	LINC01266	764581	G	C	0.349042	0.3948	0.3285	0.3979	0.2515	0.3507
7	51646419	rs17134366	1.52	0.0001343	1.508	0.0002598	0.6886	0.0275	0.9889	0.007902	intergenic	07986794;POM1	51714115	G	A	0.102236	0	0.0677	0.2806	0.0537	0.0409
4	94133036	rs112402870	1.226	0.0001347	1.272	0.0001976	0.2971	0.1786	0.5065	0.04539	ncRNA_intronic	LOC101929210	95054187	G	A	0.121206	0.2044	0.1945	0.1127	0.0129	0.1063
1	75431114	rs6672579	-0.8758	0.0001352	-0.9764	7.18e-05	-0.5329	0.0009358	-0.8703	8.06e-06	intronic	SLC44A5	75896799	G	A	0.258187	0.0317	0.3617	0.1293	0.4602	0.3845
14	52538492	rs72684248	2.044	0.0001363	1.81	0.001002	0.9811	0.02903	1.108	0.02628	intronic	TXNDC16	53005210	A	C	0.0131789	0	0.0202	0.0008	0.0288	0.0225
13	21571398	rs1963169	0.9197	0.0001367	1.06	4.62e-05	0.524	0.002234	0.8066	5.04e-05	intronic	MICU2	22145537	T	A	0.366613	0.6865	0.3718	0.1989	0.1879	0.4438
6	151617330	rs56681564	-1.169	0.0001368	-1.332	8.67e-05	-0.9236	4.54e-06	-1.008	3.4e-05	intronic	CCDC170	151938465	G	A	0.161142	0.0198	0.1513	0.1672	0.2147	0.2505
11	18964846	rs79351975	-1.093	0.0001368	-1.073	0.0003018	-0.4097	0.05153	-0.5944	0.01324	intergenic	IRGPRX1;MRGPR	NA	NA	NA	NA	NA	NA	NA	NA	NA
8	40768145	rs7841685	0.9841	0.0001375	1.071	6.2e-05	0.4439	0.01887	0.5465	0.01132	intronic	ZMAT4	40625664	G	A	0.307109	0.6171	0.2594	0.3336	0.0696	0.2301
1	75375977	rs4353040	-0.8686	0.0001377	-0.962	7.91e-05	-0.5622	0.000437	-0.8737	6.62e-06	intronic	SLC44A5	75841662	T	C	0.729034	0.9702	0.6398	0.8434	0.5328	0.591
13	21553334	rs9552448	0.9255	0.0001378	1.107	2.59e-05	0.506	0.003324	0.7725	9.82e-05	intronic	MICU2	22127473	C	T	0.336062	0.6944	0.3473	0.1036	0.168	0.4458
6	134751150	rs4896094	-0.7604	0.0001382	-0.8529	6.21e-05	-0.3231	0.01724	-0.5369	0.0009106	intergenic	101928304;ALDH	135072288	G	A	0.546326	0.5962	0.5836	0.6225	0.4543	0.4601
13	21537474	rs78519330	0.9665	0.0001382	1.187	1.92e-05	0.5346	0.0026	0.8265	6.38e-05	intronic	MICU2	NA	NA	NA	NA	NA	NA	NA	NA	NA
2	164645417	rs5835988	0.7436	0.0001383	0.8119	8.2e-05	0.5102	0.0004033	0.6456	7.63e-05	intergenic	GRB14;COBLL1	165501927	T	TG	0.405751	0.122	0.2723	0.8011	0.4404	0.2229
7	123562099	rs1608860	1.098	0.0001393	1.04	0.0006173	0.5635	0.009572	0.8948	0.0001872	intronic	NDUFA5	123202153	C	T	0.0990415	0.0546	0.085	0.0416	0.1392	0.1912
3	75801522	rs140658525	-1.2	0.0001394	-1.465	4.55e-05	-0.8648	2.29e-05	-0.9133	0.0001526	intergenic	ZNF717;ROBO2	NA	NA	NA	NA	NA	NA	NA	NA	NA
18	31788366	rs75763353	3.667	0.0001398	3.811	9.09e-05	1.918	0.02017	2.447	0.00394	intergenic	LC25A52;TRAPPC	29368329	T	C	0.0203674	0.0645	0.0043	0.0083	0.002	0.0215
4	94164517	rs148133858	1.258	0.0001403	1.234	0.0003434	0.3343	0.1363	0.4996	0.05267	ncRNA_intronic	LOC101929210	95085668	ATATAGGACTTT	G	0.123203	0.2014	0.196	0.1218	0.0139	0.1053
8	40767234	rs12675644	0.9848	0.0001403	1.066	6.69e-05	0.4782	0.01271	0.5504	0.01166	intronic	ZMAT4	40624753	A	G	0.294728	0.6121	0.255	0.2965	0.0686	0.226
3	85253323	rs12488308	-1.455	0.0001404	-1.64	0.0001799	-0.5633	0.005024	-1.233	2.26e-05	intronic	CADM2	85302473	T	G	0.251597	0.1895	0.098	0.5817	0.0994	0.135
4	123875733	rs3097934	0.7938	0.0001413	0.86	8.61e-05	0.3804	0.00895	0.5836	0.0005756	ncRNA_intronic	LINC01091	124796888	C	G	0.73143	0.5952	0.7536	0.9032	0.6332	0.7249
22	12193991	rs9282911	-1.042	0.0001414	-1.034	0.0002975	-1.022	3.03e-07	-1.06	4.89e-06	intergenic	C102723769;NO	NA	NA	NA	NA	NA	NA	NA	NA	NA
20	28784615	rs1436718853	-1.176	0.0001417	-1.31	0.0001085	-0.9363	4.63e-06	-1.104	8.11e-06	intergenic	FRG1CP;FRG1DP	NA	NA	NA	NA	NA	NA	NA	NA	NA
11	47251028	rs3758669	-0.8368	0.0001419	-0.9823	3.83e-05	-0.4818	0.001575	-0.6599	0.00019	intronic	NR1H3	47272579	A	G	0.483427	0.7758	0.3357	0.4977	0.2982	0.4581
13	21464064	rs4770162	-0.7906	0.000142	-1.001	1.72e-05	-0.6122	2.21e-05	-0.6388	0.0001562	intergenic	ZDHHC20;MICU2	22038203	A	G	0.670527	0.745	0.6729	0.7073	0.6233	0.591
22	36799011	rs17810287	1.407	0.0001424	1.316	0.0005738	0.6865	0.02848	0.9739	0.003423	intergenic	IFT27;PVALB	37195055	G	A	0.0207668	0	0.0346	0.0038	0.0646	0.0102
7	51650940	rs10261309	1.162	0.0001425	1.086	0.001009	0.5362	0.02776	0.6938	0.01573	intergenic	07986794;POM1	51718636	T	C	0.120407	0	0.0793	0.2905	0.0825	0.0828
20	29042465	rs1216581271	-1.033	0.0001428	-1.03	0.0003074	-0.8034	4.53e-05	-0.9614	2.02e-05	intergenic	FRG1CP;FRG1DP	NA	NA	NA	NA	NA	NA	NA	NA	NA
12	97931454	rs117611216	2.782	0.0001429	2.975	5.01e-05	1.446	0.01913	2.056	0.002054	intergenic	FAH1B2P2;MIR4	98325232	G	A	0.0257588	0	0.0288	0.0696	0.0169	0
2	97151116	rs1313246136	1.431	0.0001433	1.58	3.42e-05	1.159	5.96e-05	1.333	2.36e-05	intronic	ANKRD36	NA	NA	NA	NA	NA	NA	NA	NA	NA
13	21474906	rs11148119	0.9037	0.0001435	1.11	1.8e-05	0.4709	0.005413	0.7246	0.0001852	intergenic	ZDHHC20;MICU2	22049045	G	A	0.364217	0.7272	0.3833	0.1346	0.2078	0.4479
17	63971347	rs2302237	0.7306	0.0001438	0.7433	0.0001983	0.2685	0.05334	0.3949	0.01347	intronic	SCN4A	62048707	C	T	0.252796	0.3065	0.2752	0.1074	0.3688	0.2587
2	161026431	rs4664392	0.9271	0.0001444	0.9206	0.0003761	0.7878	1.81e-05	0.8841	2.79e-05	intergenic	RBMS1;TANK	161882942	C	T	0.127396	0.249	0.0893	0.0121	0.1909	0.1196
16	19590309	rs12325023	-0.9975	0.0001445	-0.9901	0.0003636	-0.3732	0.02845	-0.5861	0.003928	intronic	VPS35L	19601631	G	T	0.13099	0.0317	0.1369	0.0325	0.3241	0.1636
3	85237744	rs72915086	-1.453	0.0001446	-1.626	0.0002067	-0.557	0.005743	-1.209	3.26e-05	intronic	CADM2	85286894	G	A	0.250599	0.1855	0.0965	0.5809	0.1004	0.135
3	85263484	rs6789415	-1.457	0.000145	-1.645	0.0001839	-0.6012	0.003415	-1.234	2.42e-05	intronic	CADM2	85312634	G	T	0.243211	0.1865	0.098	0.5567	0.0974	0.1309
19	14498125	rs6511935	-0.7677	0.000145	-0.8649	5.94e-05	-0.4647	0.0009069	-0.5683	0.0005602	intergenic	GIPC1;DNAJB1	14608937	G	A	0.533946	0.2748	0.5	0.6543	0.505	0.6922
19	14498399	rs7254715	-0.7677	0.000145	-0.8649	5.94e-05	-0.4647	0.0009069	-0.5683	0.0005602	intergenic	GIPC1;DNAJB1	14609211	A	G	0.536142	0.2748	0.5	0.6611	0.505	0.6943
8	40733967	rs4618678	0.8059	0.0001451	0.8556	8.62e-05	0.3949	0.01069	0.473	0.007663	intronic	ZMAT4	40591486	T	A	0.339257	0.5704	0.3415	0.2549	0.1938	0.363
8	40734227	rs4469439	0.8059	0.0001451	0.8556	8.62e-05	0.4069	0.008687	0.473	0.007663	intronic	ZMAT4	40591746	T	G	0.339058	0.5704	0.3415	0.2542	0.1938	0.363
13	21570921	rs4770176	0.9179	0.0001455	1.099	2.66e-05	0.4977	0.003576	0.761	0.000119	intronic	MICU2	22145060	T	C	0.34345	0.6954	0.3617	0.1051	0.1879	0.4499
13	21546946	rs2040060	0.803	0.0001463	0.9256	5.22e-05	0.4948	0.001003	0.6906	8.39e-05	intronic	MICU2	22121085	T	C	0.417532	0.3065	0.4914	0.23	0.6421	0.502
20	28949593	rs1404498476	1.358	0.0001471	1.394	0.0001568	0.7187	0.01816	1.138	0.0004075	intergenic	FRG1CP;FRG1DP	NA	NA	NA	NA	NA	NA	NA	NA	NA
23	86401104	rs4134455	0.7353	0.0001471	0.7916	0.0001494	0.5253	0.0003507	0.7599	4.44e-06	intronic	DACH2	NA	NA	NA	NA	NA	NA	NA	NA	NA
13	19916906	rs9285095	0.8499	0.0001478	0.9355	7.49e-05	0.4339	0.008413	0.6889	0.0003731	intergenic	ZMYM5;ZMYM2	20491046	A	G	0.676318	0.8423	0.7161	0.4138	0.7734	0.7321
6	62901829	rs4710388	-0.8359	0.0001484	-0.7549	0.0009154	-0.5748	9.58e-05	-0.717	5.5e-05	intergenic	KHDRBS2;LGSN	63611734	C	A	0.673922	0.7718	0.7219	0.6225	0.5805	0.7045
6	151632174	rs9784821	-1.149	0.0001485	-1.312	9.29e-05	-0.8315	2.05e-05	-0.9989	3e-05	intergenic	CCDC170;ESR1	151953309	T	C	0.161542	0.0268	0.1513	0.1657	0.2127	0.2495
21	30322643	rs2832797	0.7793	0.0001485	0.7937	0.0001909	0.2217	0.1212	0.4561	0.00643	intergenic	TAP26-1;KRTAP2	31694961	C	T	0.320487	0.2639	0.3458	0.1831	0.4453	0.4182
1	143540827	rs79486674	-1.665	0.0001489	-1.567	0.0005727	-1.374	0.0001866	-1.566	5.36e-05	intergenic	.SP1P5;RNVU1-1	NA	NA	NA	NA	NA	NA	NA	NA	NA
1	24968411	rs10903119	-0.8741	0.0001491	-0.9074	0.0001811	-0.4817	0.001407	-0.7742	4.64e-05	intergenic	RUNX3;MIR4425	25294902	A	G	0.551518	0.6855	0.719	0.2746	0.5437	0.6769
6	151634860	rs73001924	-1.237	0.0001493	-1.393	0.0001225	-0.8179	6.64e-05	-1.051	4.32e-05	intergenic	CCDC170;ESR1	151955995	A	T	0.155751	0.0268	0.1427	0.1581	0.2008	0.2485
6	134758479	rs7749709	-0.7511	0.0001496	-0.8438	6.96e-05	-0.3206	0.01847	-0.5413	0.0008416	intergenic	101928304;ALDH	135079617	G	A	0.544728	0.5972	0.5807	0.6225	0.4483	0.4591
16	36117582	rs1371321543	-1.042	0.0001504	-1.113	0.0001261	-0.7607	8.64e-05	-0.6938	0.001914	intergenic	LINC02167;NONE	NA	NA	NA	NA	NA	NA	NA	NA	NA
2	161040057	rs4664032	0.9288	0.0001505	0.9271	0.0003783	0.8198	1.05e-05	0.9042	2.12e-05	intergenic	RBMS1;TANK	161896568	C	T	0.127196	0.248	0.0893	0.0129	0.1909	0.1186
11	47297661	rs11039167	-0.8712	0.0001505	-1.017	4.86e-05	-0.5872	0.0002551	-0.6992	0.0001447	intronic	MADD	47319212	C	T	0.494808	0.7262	0.3386	0.5703	0.3072	0.4581
13	21605047	rs4770182	-0.7805	0.0001506	-0.8581	9.06e-05	-0.4962	0.000558	-0.5101	0.00201	upstream	MICU2	22179186	T	G	0.330272	0.3125	0.3545	0.1505	0.4612	0.4397
5	10778817	rs75870423	1.699	0.0001509	1.679	0.0002371	0.9231	0.0182	1.185	0.00522	intergenic	DAP;CTNND2	10778929	C	T	0.0227636	0.001	0.0216	0.0356	0.0417	0.0092
20	30221652	rs201128393	1.599	0.0001512	1.721	5.57e-05	1.214	0.0005919	1.257	0.001287	intergenic	IR4477A;LINC015	NA	NA	NA	NA	NA	NA	NA	NA	NA
1	55228515	rs111928762	1.728	0.0001514	1.864	4.26e-05	1.044	0.003382	1.251	0.001745	ncRNA_intronic	MIR4422HG	55694188	A	G	0.0565096	0.0446	0.0331	0.1165	0.0378	0.0235
1	75395787	rs1249814	-0.8628	0.0001517	-0.9561	8.68e-05	-0.5473	0.0006365	-0.8686	7.47e-06	intronic	SLC44A5	75861472	C	A	0.73103	0.9722	0.6383	0.8442	0.5398	0.592
21	32434919	rs199730729	-1.217	0.0001518	-1.186	0.0004162	-1.008	8.32e-05	-1.032	0.0002546	intronic	EVA1C	NA	NA	NA	NA	NA	NA	NA	NA	NA
2	143971312	rs16823382	1.605	0.000152	1.677	9.47e-05	0.7469	0.01463	1.071	0.00232	intronic	GTDC1	144728879	G	A	0.123403	0.1637	0.0922	0.1815	0.0179	0.1339
2	143973618	rs146235644	1.605	0.000152	1.677	9.47e-05	0.8386	0.006316	1.238	0.0005052	intronic	GTDC1	144731185	G	A	0.120607	0.1627	0.0922	0.1747	0.0169	0.1309
23	3364359	rs2376512	0.6984	0.0001535	0.6887	0.0004246	0.4313	0.002103	0.4706	0.003042	intergenic	MXRA5;SNORA48	NA	NA	NA	NA	NA	NA	NA	NA	NA
8	40763574	rs4129325	0.9714	0.0001544	1.051	7.63e-05	0.5101	0.007428	0.6106	0.005	intronic	ZMAT4	40621093	A	G	0.299521	0.6161	0.2579	0.3079	0.0696	0.228
7	123615417	rs4727973	1.034	0.0001552	0.9683	0.0008806	0.5538	0.006513	0.8221	0.0002248	ncRNA_intronic	ASB15-AS1	123255471	G	A	0.145767	0.0942	0.098	0.1672	0.1451	0.2045
18	58762068	rs12961764	-0.867	0.0001556	-0.8487	0.0004011	-0.4879	0.0006792	-0.8157	9.27e-06	intergenic	MALT1;LINC0192	56429300	T	C	0.628594	0.6349	0.6614	0.5635	0.7425	0.5695
2	164651879	rs10184004	0.7567	0.0001558	0.8235	9.94e-05	0.5132	0.0004521	0.6602	7.29e-05	intergenic	GRB14;COBLL1	165508389	C	T	0.399561	0.0962	0.2622	0.7988	0.4433	0.2249
1	55226016	rs66679331	1.724	0.0001561	1.86	4.39e-05	1.049	0.00325	1.259	0.001658	ncRNA_intronic	MIR4422HG	55691689	G	T	0.0567093	0.0446	0.0331	0.1172	0.0378	0.0235
5	128035896	rs10063647	0.7259	0.0001561	0.7609	0.000187	0.5752	3.8e-05	0.537	0.0007479	ncRNA_intronic	LINC01184	127371588	A	G	0.636781	0.7341	0.5677	0.8071	0.4195	0.5787
19	1728999	rs6510621	-0.7883	0.0001562	-0.8007	0.0002495	-0.4501	0.001355	-0.6758	7.52e-05	intergenic	TCF3;ONECUT3	1728998	T	C	0.493411	0.3988	0.4755	0.4183	0.5497	0.6472
22	12615040	rs1373227070	-1.044	0.0001579	-1.067	0.0002209	-1.014	6.94e-07	-0.9735	2.54e-05	intergenic	C102723769;NO	NA	NA	NA	NA	NA	NA	NA	NA	NA
4	116191884	rs1857934	1.091	0.000158	1.186	6.38e-05	0.7062	0.001075	0.9122	0.000258	intergenic	NDST4;MIR1973	117113040	G	A	0.755591	0.9097	0.8156	0.3903	0.8926	0.907
4	116192383	rs1395435	1.091	0.000158	1.186	6.38e-05	0.7096	0.00102	0.9184	0.0002373	intergenic	NDST4;MIR1973	117113539	G	T	0.75619	0.9097	0.8156	0.3911	0.8946	0.907
8	40734306	rs4554450	0.8021	0.0001583	0.8517	9.44e-05	0.3961	0.01059	0.4675	0.008434	intronic	ZMAT4	40591825	A	G	0.345248	0.5704	0.3444	0.2761	0.1938	0.363
8	40734309	rs4526345	0.8021	0.0001583	0.8517	9.44e-05	0.3961	0.01059	0.4675	0.008434	intronic	ZMAT4	40591828	A	C	0.343051	0.5704	0.3444	0.2678	0.1938	0.363
17	21752343	rs1387678001	-1.02	0.0001584	-1.148	6.83e-05	-0.8804	6.17e-06	-0.8995	5.82e-05	intergenic	KCNJ18;UBBP4	NA	NA	NA	NA	NA	NA	NA	NA	NA
22	16438034	rs113636112	1.044	0.000159	1.23	2.43e-05	0.7049	0.0005765	0.792	0.0006455	intergenic	DUXAP8;CCT8L2	NA	NA	NA	NA	NA	NA	NA	NA	NA
4	94166944	rs61236700	1.247	0.0001592	1.223	0.0003842	0.3241	0.1486	0.4905	0.05703	ncRNA_intronic	LOC101929210	95088095	C	T	0.123802	0.2024	0.196	0.1233	0.0139	0.1053
14	27345691	rs12881090	0.7439	0.0001592	0.7225	0.00041	0.4519	0.002185	0.4494	0.007008	ncRNA_intronic	LOC728755	27814897	G	T	0.267572	0.127	0.3862	0.0628	0.4851	0.3814
3	163533268	rs9831104	1.079	0.0001593	1.148	8.2e-05	0.698	0.001267	0.7826	0.001262	intergenic	NC01192;MIR12	163251056	C	A	0.155152	0.121	0.1686	0.174	0.0805	0.2321
8	40685533	rs11989433	0.7496	0.0001598	0.7324	0.0004194	0.5538	9.73e-05	0.581	0.0003262	intronic	ZMAT4	40543052	A	G	0.478235	0.4415	0.4625	0.4962	0.5527	0.4264
13	19918318	rs9508955	0.8142	0.0001599	0.8831	9.37e-05	0.3827	0.01599	0.6299	0.0006572	intergenic	ZMYM5;ZMYM2	20492458	G	A	0.685903	0.8502	0.7161	0.4607	0.7674	0.7157
11	118887380	rs497916	0.8583	0.0001601	0.9019	0.0001708	0.4717	0.003444	0.6056	0.001418	intronic	CXCR5	118758089	C	T	0.259984	0.2312	0.3184	0.3215	0.2704	0.1544
19	14499183	rs8108117	-0.7744	0.0001603	-0.8441	9.83e-05	-0.4287	0.002394	-0.5467	0.001027	intergenic	GIPC1;DNAJB1	14609995	G	T	0.507987	0.2748	0.4928	0.559	0.505	0.6933
11	47323549	rs10838692	-0.8164	0.0001604	-0.951	4.62e-05	-0.5198	0.0005717	-0.6528	0.0001728	intronic	MADD	47345100	T	C	0.479633	0.7262	0.3357	0.5144	0.3072	0.4581
11	47330858	rs4752825	-0.8164	0.0001604	-0.951	4.62e-05	-0.5198	0.0005717	-0.6528	0.0001728	downstream	MADD;MYBPC3	47352409	G	A	0.478235	0.7252	0.3357	0.5098	0.3072	0.4581
6	136157355	rs9376176	2.874	0.0001609	3.196	0.0001907	1.025	0.06869	1.574	0.009028	intronic	PDE7B	136478493	C	G	0.0167732	0.0685	0.0115	0	0.005	0.002
3	85239406	rs58223718	-1.443	0.0001611	-1.626	0.0002076	-0.5658	0.00497	-1.223	2.64e-05	intronic	CADM2	85288556	G	A	0.250599	0.1855	0.0965	0.5809	0.1004	0.135
3	101078194	rs7646803	0.8442	0.0001618	0.9465	5.74e-05	0.4329	0.009561	0.6189	0.0009288	intergenic	ABI3BP;IMPG2	100797038	G	C	0.788938	0.8869	0.7795	0.8283	0.7018	0.7311
1	143540816	rs148478882	-2.028	0.0001628	-2.024	0.0002619	-1.833	8.57e-05	-1.993	4.02e-05	intergenic	.SP1P5;RNVU1-1	149035478	G	A	0.000199681	0	0	0	0	0.001
3	157652056	rs79239055	2.797	0.0001636	3.166	9.34e-05	0.8438	0.1156	1.399	0.01642	intergenic	SLC66A1L;SHOX2	157369845	C	T	0.00998403	0.0417	0.0115	0	0	0
7	123529141	rs4731110	1.032	0.0001646	0.9873	0.0005671	0.4893	0.0172	0.8619	0.000163	intronic	IQUB	123169195	A	C	0.184305	0.0575	0.0994	0.2587	0.1431	0.317
12	11264863	rs145733576	1.543	0.0001654	1.651	8.44e-05	0.8619	0.01698	1.227	0.001421	intergenic	TAS2R42;PRB3	11417796	G	GA	0.013778	0	0.0231	0.0015	0.0447	0.0061
8	15788796	rs62503711	1.259	0.000166	1.243	0.0003341	0.668	0.01872	0.7918	0.01025	intergenic	TUSC3;MSR1	15646305	C	T	0.0493211	0.0278	0.0461	0.0356	0.0795	0.0613
8	15789170	rs73667507	1.259	0.000166	1.243	0.0003341	0.668	0.01872	0.7918	0.01025	intergenic	TUSC3;MSR1	15646679	A	T	0.0491214	0.0278	0.0461	0.0356	0.0795	0.0603
8	40763989	rs4736879	0.9721	0.000166	1.054	7.91e-05	0.4717	0.01333	0.5558	0.0104	intronic	ZMAT4	NA	NA	NA	NA	NA	NA	NA	NA	NA
8	40763999	rs4999665	0.9721	0.000166	1.054	7.91e-05	0.4717	0.01333	0.5558	0.0104	intronic	ZMAT4	40621518	C	T	0.296126	0.6171	0.255	0.2965	0.0686	0.228

8	40764552	rs59936904	0.9721	0.000166	1.054	7.91e-05	0.4717	0.01333	0.5558	0.0104	intronic	ZMAT4	NA	NA	NA	NA	NA	NA	NA	NA	NA
8	40765279	rs57956863	0.9721	0.000166	1.054	7.91e-05	0.4539	0.01689	0.5558	0.0104	intronic	ZMAT4	40622798	TC	T	0.296126	0.6171	0.255	0.2965	0.0686	0.228
8	40765831	rs60495745	0.9721	0.000166	1.054	7.91e-05	0.4539	0.01689	0.5558	0.0104	intronic	ZMAT4	40623350	A	G	0.296326	0.6171	0.255	0.2973	0.0686	0.228
8	40766239	rs6992762	0.9721	0.000166	1.054	7.91e-05	0.4717	0.01333	0.5558	0.0104	intronic	ZMAT4	40623758	T	C	0.296326	0.6171	0.255	0.2965	0.0686	0.229
18	15408881	rs79920853	1.309	0.0001668	1.258	0.0006165	0.7662	0.007016	0.7415	0.0205	intergenic	.OC644669;NONI	NA	NA	NA	NA	NA	NA	NA	NA	NA
20	28875139	rs1328147728	-1.018	0.0001672	-1.032	0.0002958	-0.8414	1.94e-05	-0.9017	7.53e-05	intergenic	FRG1CP;FRG1DP	NA	NA	NA	NA	NA	NA	NA	NA	NA
17	75552782	rs11077793	0.7449	0.0001674	0.6929	0.0008173	0.3784	0.006361	0.5933	0.0002592	intronic	LLGL2	73548863	C	A	0.41254	0.4683	0.3847	0.1271	0.5616	0.6074
4	140081643	rs10519523	1.445	0.0001676	1.611	4.1e-05	1.061	0.0008735	1.273	0.0002524	intronic	MAML3	141002797	T	G	0.0503195	0	0.0331	0.0045	0.1024	0.1227
13	21597492	rs2147125	0.9027	0.0001677	1.117	1.97e-05	0.5014	0.003325	0.7556	0.0001255	intronic	MICU2	22171631	C	A	0.343251	0.6954	0.3617	0.1044	0.1889	0.4489
16	32096589	rs201468572	-1.628	0.0001682	-1.624	0.0004314	-0.9414	7.81e-05	-1.076	0.0002158	intergenic	F267;LOC102723	NA	NA	NA	NA	NA	NA	NA	NA	NA
11	47257366	rs3758673	-0.8276	0.0001687	-0.9666	4.87e-05	-0.4999	0.001156	-0.6658	0.0001805	intronic	NR1H3	47278917	C	T	0.483427	0.7748	0.3357	0.5	0.2972	0.4571
9	63861872	rs62545432	1.463	0.000169	1.741	0.0001301	1.1	3.03e-06	1.333	7.49e-06	intergenic	FRG1JP;FLJ43315	NA	NA	NA	NA	NA	NA	NA	NA	NA
6	15327842	rs7765425	1.319	0.0001691	1.326	0.0002869	0.9911	0.0007182	1.294	5.37e-05	intronic	JARID2	15328073	G	A	0.096845	0.0526	0.0562	0.174	0.0447	0.1207
11	69999599	rs78066979	2.045	0.0001691	2.2	6.43e-05	1.03	0.02126	1.463	0.002075	intronic	ANO1	69845705	A	C	0.0257588	0.0923	0.0072	0.0008	0.0129	0.0174
13	21492795	rs1044069	0.8793	0.0001691	1.055	3.08e-05	0.457	0.00631	0.7316	0.0001391	UTR3	MICU2	22066934	G	A	0.374401	0.6607	0.3847	0.2307	0.1978	0.4479
23	86464163	rs5968928	0.7629	0.0001694	0.7311	0.0007615	0.5458	0.0003616	0.6846	8.75e-05	intronic	DACH2	NA	NA	NA	NA	NA	NA	NA	NA	NA
7	123612569	rs12706542	1.028	0.0001697	0.9633	0.0009354	0.531	0.009151	0.8182	0.0002411	intronic	ASB15	123252623	G	C	0.138978	0.0952	0.098	0.143	0.1451	0.2014
1	200623596	rs2808252	-0.8705	0.0001699	-0.779	0.00106	-0.3575	0.01558	-0.566	0.001416	intergenic	KIF14;DDX59	200592724	T	C	0.720647	0.7173	0.7594	0.8313	0.5755	0.6963
6	62898281	rs13196154	-0.8225	0.0001702	-0.7549	0.0009154	-0.5718	0.0001024	-0.7111	6.06e-05	intergenic	KHDRBS2;LGSN	63608186	G	A	0.673722	0.7718	0.7219	0.6218	0.5805	0.7045
11	47305666	rs1375688	-0.8122	0.0001703	-0.9457	4.96e-05	-0.5296	0.0004577	-0.6698	0.0001225	intronic	MADD	47327217	G	A	0.479433	0.7262	0.3357	0.5136	0.3072	0.4581
1	200623419	rs2808250	-0.8645	0.0001714	-0.7576	0.001391	-0.345	0.01833	-0.5446	0.001837	intergenic	KIF14;DDX59	200592547	A	T	0.721246	0.7173	0.7594	0.8328	0.5755	0.6973
2	164672114	rs1128249	0.754	0.0001715	0.8151	0.0001207	0.5389	0.0002619	0.6731	5.8e-05	intronic	COBLL1	165528624	G	T	0.39397	0.0962	0.2579	0.7988	0.4344	0.2086
13	21528516	rs9316436	0.9442	0.0001716	1.125	3.48e-05	0.4854	0.007379	0.775	0.000178	intronic	MICU2	22102655	C	G	0.30631	0.6944	0.3329	0.0121	0.163	0.4325
3	36138290	rs13067111	0.7926	0.0001717	0.8982	5.55e-05	0.3626	0.02388	0.4924	0.006069	intergenic	ARPP21;STAC	36179782	T	C	0.169529	0.0734	0.2075	0.1165	0.2992	0.18
3	36138931	rs34910777	0.7926	0.0001717	0.8982	5.55e-05	0.364	0.02336	0.4955	0.0058	intergenic	ARPP21;STAC	36180423	G	A	0.169728	0.0734	0.2075	0.1165	0.2992	0.181
7	62301988	rs78898560	1.154	0.0001723	1.303	0.0001069	0.8592	2.02e-05	0.9441	8.61e-05	intergenic	NONE;ZNF733P	NA	NA	NA	NA	NA	NA	NA	NA	NA
1	200689267	rs61825137	-0.9333	0.0001725	-0.8631	0.0007849	-0.3349	0.02592	-0.588	0.001321	ncRNA_intronic	DDX59-AS1	200658395	C	A	0.257188	0.2917	0.2233	0.1483	0.3827	0.2638
1	12163425	rs590314	-0.8767	0.0001727	-0.8739	0.0003407	-0.2666	0.07093	-0.4721	0.008001	intergenic	TNFRSF8;MIR784	12223482	G	A	0.278155	0.2817	0.2651	0.1195	0.4006	0.3722
8	40773556	rs13276178	0.9709	0.0001727	1.049	8.75e-05	0.4374	0.02041	0.5394	0.01259	intronic	ZMAT4	40631075	A	C	0.682708	0.378	0.7378	0.6324	0.9314	0.7699
7	35926890	rs796894942	-1.015	0.0001732	-0.9382	0.0008941	-0.5369	0.005965	-0.6809	0.002372	intergenic	TIN7;LOC101928	NA	NA	NA	NA	NA	NA	NA	NA	NA
8	61375993	rs144878509	4.689	0.0001735	4.693	0.0002588	2.448	0.02516	2.956	0.00909	intronic	CLVS1	62288552	T	A	0.00938498	0.0437	0	0	0	0.0031
2	212453917	rs974968	0.8098	0.0001738	0.8067	0.0003207	0.5505	0.0004034	0.7117	7.54e-05	intronic	ERBB4	213318641	A	C	0.441294	0.129	0.3934	0.7352	0.4423	0.3988
1	212469777	rs11119946	-0.7986	0.0001739	-0.9016	7.69e-05	-0.5769	6.79e-05	-0.6476	0.0001249	intergenic	NENF;LINC0174(	212643119	C	T	0.590855	0.6468	0.5317	0.7602	0.498	0.4417
11	47320669	rs10742801	-0.8243	0.0001745	-0.9598	5.23e-05	-0.5256	0.0005845	-0.6715	0.0001381	intronic	MADD	47342220	A	C	0.496006	0.7262	0.3429	0.5726	0.3072	0.4581
16	18595407	rs7500593	1.017	0.0001748	1.033	0.0003105	0.4812	0.01343	0.6162	0.005688	ncRNA_intronic	ABCC6P1	NA	NA	NA	NA	NA	NA	NA	NA	NA
3	90411506	rs1166666910	-1.099	0.0001752	-1.222	0.0001285	-0.806	4.44e-05	-0.7628	0.0008439	intergenic	EPHA3;NONE	NA	NA	NA	NA	NA	NA	NA	NA	NA
1	225837989	rs2671267	1.112	0.0001753	1.207	7.66e-05	0.8615	9.49e-05	0.89	0.0003441	intronic	EPHX1	226025690	G	A	0.131589	0.1181	0.0677	0.1263	0.1004	0.2301
20	29482553	rs1418717426	-1.068	0.0001753	-1.015	0.0005962	-0.9485	1.79e-06	-1.109	2.86e-06	ncRNA_intronic	FRG1EP	NA	NA	NA	NA	NA	NA	NA	NA	NA
20	30402330	rs62196405	1.021	0.000176	1.146	8.4e-05	0.8056	2.49e-05	0.9449	2.3e-05	ncRNA_intronic	FRG1BP	NA	NA	NA	NA	NA	NA	NA	NA	NA
7	123462775	rs34691578	1.086	0.0001762	1.013	0.0008581	0.5716	0.009076	0.8978	0.0001918	intronic	IQUB	123102829	G	A	0.0886581	0.0526	0.0821	0.0098	0.1421	0.182
10	38755362	rs370698663	1.01	0.0001765	1.141	6.92e-05	0.5824	0.002334	0.5675	0.009917	intergenic	ACTR3BP5;NONE	NA	NA	NA	NA	NA	NA	NA	NA	NA
10	69096699	rs2805907	0.7628	0.0001769	0.8371	9.94e-05	0.5206	0.0002463	0.5629	0.0006424	intronic	SRGN	70856455	G	A	0.450479	0.4851	0.4856	0.1785	0.5895	0.6145
1	75394170	rs1249816	-0.8613	0.0001777	-0.9795	6.29e-05	-0.549	0.000643	-0.8701	8.2e-06	intronic	SLC44A5	75859855	T	A	0.730831	0.9712	0.6383	0.8442	0.5398	0.592
1	75392789	rs1249821	-0.8573	0.0001782	-0.9507	0.0001023	-0.5318	0.0009484	-0.8631	9.25e-06	intronic	SLC44A5	75858474	T	C	0.736821	0.9722	0.6354	0.8684	0.5398	0.591
1	75399034	rs1249780	-0.8573	0.0001782	-0.9507	0.0001023	-0.5318	0.0009484	-0.8631	9.25e-06	intronic	SLC44A5	75864719	C	T	0.738818	0.9722	0.6383	0.8684	0.5398	0.5992
1	75399340	rs1249779	-0.8573	0.0001782	-0.9507	0.0001023	-0.5318	0.0009484	-0.8631	9.25e-06	intronic	SLC44A5	75865025	T	G	0.736621	0.9722	0.6383	0.8623	0.5398	0.5961
1	75410788	rs1249795	-0.8573	0.0001782	-0.9507	0.0001023	-0.5318	0.0009484	-0.8631	9.25e-06	intronic	SLC44A5	75876473	C	T	0.738419	0.9722	0.6398	0.8661	0.5398	0.5992
1	75415345	rs1249801	-0.8573	0.0001782	-0.9507	0.0001023	-0.5318	0.0009484	-0.8631	9.25e-06	intronic	SLC44A5	75881030	T	G	0.738818	0.9722	0.6383	0.8684	0.5398	0.5992
1	75428090	rs1249785	-0.8573	0.0001782	-0.9507	0.0001023	-0.5318	0.0009484	-0.8631	9.25e-06	intronic	SLC44A5	75893775	A	T	0.742013	0.9722	0.6383	0.8684	0.5398	0.6155
6	15354382	rs10949294	1.261	0.0001786	1.219	0.0004948	0.9086	0.0007539	1.185	9.88e-05	intronic	JARID2	15354613	A	T	0.130591	0.0675	0.062	0.2859	0.0447	0.1227
2	212500980	rs5838337	0.9239	0.0001788	0.9688	0.0001385	0.6935	0.0001887	0.8614	5.66e-05	intronic	ERBB4	213365704	G	GA,GGA	340855,0.001397	0.0933,0	0.2608,0	0.7526,0.0053	0.1809,0	0.2607,0
4	6409866	rs4282260	-0.9317	0.0001793	-1.188	2.94e-05	-0.656	8.08e-05	-0.6129	0.001398	intronic	PPP2R2C	6411593	C	T	0.284145	0.6885	0.1585	0.0469	0.2475	0.3149
1	212229182	rs6679796	0.751	0.0001794	0.8151	0.0001052	0.3293	0.02436	0.4591	0.006256	ncRNA_intronic	LINC02608	212402524	T	C	0.29353	0.1984	0.3573	0.2383	0.4066	0.3047
1	212231605	rs6668866	0.751	0.0001794	0.8151	0.0001052	0.3293	0.02436	0.4591	0.006256	ncRNA_intronic	LINC02608	212404947	T	C	0.29373	0.1984	0.3588	0.239	0.4066	0.3037
1	75391524	rs1249826	-0.86	0.0001802	-0.9795	6.27e-05	-0.5484	0.0006491	-0.8724	7.79e-06	intronic	SLC44A5	75857209	G	A	0.730631	0.9722	0.6354	0.8442	0.5398	0.592
14	51752108	rs569699407	-1.471	0.000181	-1.444	0.0004434	-1.071	7.91e-06	-1.249	2.51e-05	intergenic	FRMD6;GNG2	52218826	C	T	0.000199681	0	0	0	0	0.001
14	51752109	rs1321063331	-1.471	0.000181	-1.444	0.0004434	-1.071	7.91e-06	-1.249	2.51e-05	intergenic	FRMD6;GNG2	NA	NA	NA	NA	NA	NA	NA	NA	NA
4	189988450	rs75351524	-1.026	0.0001813	-1.223	2.59e-05	-0.6739	0.0006183	-0.7691	0.0006788	intergenic	FRG1;FRG2	NA	NA	NA	NA	NA	NA	NA	NA	NA
6	70615020	rs1524738	0.7431	0.0001819	0.9739	6.36e-06	0.3891	0.005626	0.4335	0.00664	intergenic	SDHAF4;SMAP1	71324723	G	T	0.609026	0.5188	0.755	0.671	0.5835	0.5409
14	74165117	rs75410415	1.548	0.0001819	1.723	3.83e-05	0.9272	0.009495	1.254	0.0008868	intronic	LIN52	74631820	T	C	0.0233626	0.001	0.0288	0.0015	0.0507	0.044
7	62309235	rs78598581	-1.164	0.0001823	-1.297	5.72e-05	-1.091	5.85e-06	-1.033	0.0001422	intergenic	NONE;ZNF733P	NA	NA	NA	NA	NA	NA	NA	NA	NA
8	40761956	rs60356325	0.9671	0.0001835	1.049	8.81e-05	0.4437	0.01946	0.5492	0.0114	intronic	ZMAT4	40619475	C	T	0.299121	0.6171	0.255	0.3071	0.0696	0.228
17	26953467	rs145252921	1.008	0.0001835	1.029	0.0002706	0.7076	0.0002669	0.9282	3.35e-05	intergenic	ONE;LOC1053717	NA	NA	NA	NA	NA	NA	NA	NA	NA
20	28637994	rs1253464333	1.717	0.0001843	1.552	0.0007353	1.001	4.1e-05	1.307	3.36e-05	intergenic	FRG1CP;FRG1DP	NA	NA	NA	NA	NA	NA	NA	NA	NA
6	134742311	rs7754588	-0.7442	0.0001847	-0.8356	8.74e-05	-0.3154	0.02023	-0.5339	0.001015	intergenic	101928304;ALDH	135063449	G	A	0.546326	0.5962	0.5836	0.6218	0.4553	0.4601
15	91394730	rs79897663	1.186	0.0001849	1.365	3.75e-05	0.692	0.00235	0.8081	0.00214	intergenic	SV2B;CRAT37	91937960	A	C	0.131789	0.2946	0.098	0.112	0.0755	0.0726
2	91787556	rs1255965559	-1.015	0.0001851	-1.131	8.69e-05	-0.9587	8.2e-07	-0.9429	2.42e-05	intergenic	GGT8P;ACTR3BP2	NA	A	NA	NA	NA	NA	NA	NA	NA
1	156784325	rs114955967	1.942	0.0001853	1.892	0.0003595	1.133	0.01648	1.399	0.005019	intronic	PRCC	156754117	G	C	0.014976	0	0.0245	0	0.0308	0.0276
11	47333236	rs1052373	-0.8106	0.0001853	-0.9465	5.28e-05	-0.5081	0.0007693	-0.644	0.0002178	exonic	MYBPC3	47354787	C	T	0.476238	0.7202	0.3329	0.5083	0.3072	0.4571
10	14495297	rs142509510	2.048	0.0001855	2.214	5.23e-05	1.445	0.003483	1.607	0.002054	intergenic	MIR1265;FAM107	14537296	A	G	0.00758786	0	0.0187	0.0008	0.0239	0
16	32409840	rs201874489	1.156	0.0001867	1.235	0.0001138	0.8287	0.0008244	1.059	0.0001045	intergenic	90705;LOC10536	NA	A	NA	NA	NA	NA	NA	NA	NA
6	62899755	rs4440443	-0.8194	0.0001871	-0.7515	0.0009938	-0.5669	0.0001215	-0.7082	6.76e-05	intergenic	KHDRBS2;LGSN	63609660	T	C	0.673722	0.7718	0.7219	0.6218	0.5805	0.7045
18	67295481	rs145048756	2.422	0.0001871	2.557	7.42e-05	1.532	0.008125	1.814	0.002407	intergenic	MIR5011;DSEL	64962718	C	A	0.0071885	0	0.0144	0.0008	0.0189	0.0061
4	6408336	rs11737674	-0.9236	0.0001876	-1.177	3e-05	-0.6527	7.14e-05	-0.662	0.0006511	intronic	PPP2R2C	6410063	C	T	0.326877	0.6915	0.1571	0.2088	0.2366	0.3241
14	27315233	rs396419	0.7375	0.0001878	0.6951	0.000688	0.4986	0.0007873	0.4612	0.005961	intergenic	84307HG;LOC728	27784439	T	G	0.297923	0.1111	0.4035	0.149	0.4791	0.4305
1	5673339	rs72863360	1.04	0.0001879	1.087	0.0002148	0.8036	3.59e-05	0.9181	4.75e-05	intergenic	AJAP1;MIR4689	NA	NA	NA	NA	NA	NA	NA	NA	NA
4	94135549	rs57359435	1.259	0.0001879	1.266	0.0003969	0.3213	0.1568	0.5172	0.04917	ncRNA_intronic	LOC101929210	95056700	A	G	0.120407	0.2054	0.1945	0.1127	0.008	0.1063
6	134754504	rs7765987	-0.7403	0.0001879	-0.8266	9.1e-05	-0.3146	0.02072	-0.528	0.001083	intergenic	101928304;ALDH	135075642	C	A	0.54393	0.5962	0.5807	0.621	0.4463	0.4601
6	166524379	rs3778417	0.7282	0.000188	0.8425	4.8e-05	0.3886	0.005096	0.5525	0.0005726	intronic	RPS6KA2	166937867	G	A	0.391174	0.5476	0.2579	0.3729	0.3976	0.3425
19	56406822	rs182569837	2.487	0.000189	2.509	0.0001846	1.4	0.0191	2.087	0.0008672	intronic	ZNF583	56918191	T	C	0.00738818	0.001	0.0086	0.0038	0.0179	0.0072
13	21502791	rs3843680	0.8643	0.0001892	1.074	2.15e-05	0.474	0.004501	0.7148	0.0001688	intronic	MICU2	22076930	G	C	0.359625	0.6617	0.3761	0.1861	0.1978	0.4376
13	21478244	rs8000137	0.8655	0.0001894	1.01	5.49e-05	0.5039	0.002364	0.7496	8.83e-05	intergenic	ZDHHC20;MICU2	22052383	G	A	0.394768	0.6944	0.3948	0.2708	0.2127	0.4407
11	47229648	rs830085	-0.8227	0.0001897	-0.9681	5.11e-05	-0.4657	0.002219	-0.6022	0.0006145	intergenic	DDB2	47251199	G	A	0.508986	0.2232	0.6571	0.4766	0.6998	0.546
15	63861957	rs2197259	0.8992	0.0001898	0.9887	7.92e-05	0.3577	0.05627	0.5783	0.005992	intergenic	HERC1;MIR422A	64154156	T	C	0.792931	0.9653	0.7637	0.5855	0.838	0.8701
11	47269433	rs1449627	-0.8056	0.00019	-0.939	5.53e-05	-0.4881	0.001211	-0.6272	0.0003233	UTR5	MADD	47290984	T	G	0.480232	0.7262	0.3372	0.5166	0.3072	0.4571
11	47295140	rs11039166	-0.8056	0.00019	-0.939	5.53e-05	-0.5108	0.0007421	-0.6436	0.0002299	intronic	MADD	47316691	A	C	0.48103	0.7262	0.3372	0.5189	0.3072	0.4581
3	75632227	rs75367776	1.966	0.0001904	2.159	4.44e-05	1.067	0.01666	1.567	0.000943	intergenic	MIR1324;FRG2C	NA	NA	NA	NA	NA	NA	NA	NA	NA
1	75457213	rs2799052	-0.8405	0.0001906	-0.9283	0.0001145	-0.5317	0.0008136	-0.8118	1.97e-05	intronic	SLC44A5	75922898	G	A	0.675319	0.9752	0.6124	0.6619	0.5338	0.5746
7	51654301	rs7803437	1.124	0.0001923	1.046	0.001341	0.5226	0.02955	0.6903	0.01434	intergenic	07986794;POM1	51721997	G	T	0.127596	0.0149	0.0793	0.2935	0.0855	0.0971
1	75389905	rs1913124	-0.8708	0.0001928	-1.007	5.76e-05	-0.5148	0.001555	-0.874	9.73e-06	intronic	SLC44A5	75855590	C	T	0.73742	0.9722	0.6383	0.8684	0.5398	0.592
1	200627478	rs2809344	-0.8546	0.0001931	-0.765	0.00117	-0.3525	0.01577	-0.5462	0.001719	intergenic	KIF14;DDX59	200596606	C	A	0.717452	0.7163	0.7594	0.8185	0.5765	0.6973
2	164688863	rs10630427	0.7522	0.0001938	0.8344	9.56e-05	0.5444	0.0002595	0.7022	3.39e-05	intronic	COBLL1	165545373	C	CAA	0.396166	0.0982	0.2579	0.8003	0.4433	0.2065
1	150810763	rs10305752	2.378	0.0001942	2.338	0.0003464	1.743	0.002855	1.928	0.001434	UTR3	ARNT	150783239	A	AG	0.00858626	0	0.0173	0	0.0258	0.0051
11	47313790	rs7940809	-0.8034	0.0001943	-0.9361	5.71e-05	-0.5113	0.0006921	-0.6419	0.0002128	intronic	MADD	47335341	C	T	0.479633	0.7262	0.3357	0.5136	0.3072	0.4591
5	128042602	rs6887276	0.7171	0.0001948	0.7514	0.0002382	0.573	4.16e-05	0.5328	0.0008454	ncRNA_intronic	LINC01184	127378294	C	G	0.636581	0.7351	0.5663	0.8064	0.4195	0.5787
4	116193866	rs1534133	1.072	0.0001949	1.168	7.78e-05	0.6835	0.00144	0.8834	0.0003646	intergenic	NDST4;MIR1973	117115022	T	C	0.75639	0.9117	0.817	0.3903	0.8936	0.907
4	116195227	rs2135576	1.072	0.0001949	1.168	7.78e-05	0.6835	0.00144	0.8834	0.0003646	intergenic	NDST4;MIR1973	117116383	A	G	0.75599	0.9097	0.8156	0.3911	0.8936	0.907
17	26962431	rs76931425	-1.301	0.000195	-1.209	0.0008463	-1.031	4.52e-06	-1.079	5.44e-05	intergenic	ONE;LOC1053717	NA	NA	NA	NA	NA	NA	NA	NA	NA
7	123531788	rs3863952	1.019	0.0001951	0.9762	0.0006512	0.4732	0.02065	0.8529	0.0001832	intronic	IQUB	123171842	A	G	0.184105	0.0575	0.0994	0.2572	0.1431	0.318

8	138237708	rs4909763	0.7268	0.0001952	0.8187	5.93e-05	0.3738	0.009052	0.5999	0.0002597	intronic	FAM135B	139249951	C	T	0.272963	0.2768	0.2839	0.3911	0.2575	0.1176
7	62301560	rs74861477	-1.279	0.0001957	-1.377	8.09e-05	-0.9467	0.0003558	-1.184	5.58e-05	intergenic	NONE;ZNF733P	61745811	C	T	0.00359425	0.001	0	0.0091	0.002	0.0031
10	31703177	rs11008602	2.268	0.0001959	1.99	0.002003	1.979	9.11e-05	2.126	0.0001146	intergenic	ZEB1;ARHGAP12	31992105	C	T	0.0349441	0.0437	0.0086	0	0.0179	0.1094
2	161040273	rs16845493	0.9104	0.0001965	0.9028	0.000516	0.7749	2.5e-05	0.8603	4.67e-05	intergenic	RBMS1;TANK	161896784	C	G	0.127196	0.248	0.0893	0.0129	0.1909	0.1186
7	61065630	rs1161356439	1.06	0.0001975	1.171	7.54e-05	0.6829	0.001651	0.6629	0.007521	intergenic	NONE;NONE	NA	NA	NA	NA	NA	NA	NA	NA	NA
17	41233249	rs796271992	1.03	0.0001975	1.135	9.96e-05	0.5255	0.01054	0.8197	0.0004312	UTR3	KRTAP9-3	NA	NA	NA	NA	NA	NA	NA	NA	NA
13	21475186	rs1744907	0.8633	0.0001982	0.982	7.89e-05	0.4268	0.009763	0.6731	0.0003852	intergenic	ZDHHC20;MICU2	22049325	A	G	0.56889	0.2996	0.5865	0.6089	0.7893	0.5532
7	123451299	rs12539600	1.038	0.0001986	0.9894	0.0007246	0.469	0.02399	0.8193	0.0003839	downstream	IQUB	123091353	G	C	0.15016	0.0556	0.1009	0.1838	0.1421	0.2454
2	9940326	rs10197700	1.065	0.0001987	1.156	9.36e-05	0.4406	0.03535	0.69	0.003958	intergenic	TAF1B;GRHL1	10080455	C	T	0.161741	0.1806	0.049	0.261	0.0815	0.1708
11	48855500	rs140322432	2.716	0.0001988	2.555	0.0007517	1.555	0.01829	1.979	0.005064	intergenic	OR4A47;TRIM49	NA	NA	NA	NA	NA	NA	NA	NA	NA
3	36096348	rs62244982	0.7581	0.0001991	0.8442	7.88e-05	0.2884	0.06225	0.4396	0.01093	intergenic	ARPP21;STAC	36137840	C	T	0.165735	0.0734	0.2089	0.0976	0.3082	0.1759
7	51641597	rs10244860	1.492	0.0001993	1.475	0.000396	0.7759	0.01688	1.001	0.008664	intergenic	07986794;POM1	51709293	C	T	0.0728834	0	0.0591	0.1762	0.0527	0.0389
7	51647610	rs10227253	1.492	0.0001993	1.475	0.000396	0.7759	0.01688	1.001	0.008664	intergenic	07986794;POM1	51715306	C	A	0.0758786	0	0.0591	0.1861	0.0527	0.0409
17	21688395	rs1340370867	1.216	0.0001994	1.146	0.0006557	0.751	0.0002114	0.9633	9.65e-05	intergenic	INC02693;KCNJ1	NA	NA	NA	NA	NA	NA	NA	NA	NA
13	21578900	rs1323891	0.8912	0.0001995	1.107	2.35e-05	0.4897	0.004077	0.7461	0.0001505	intronic	MICU2	22153039	C	T	0.342652	0.6954	0.3617	0.1036	0.1889	0.4468
6	15333861	rs6919296	1.306	0.0002001	1.305	0.0003628	0.9917	0.0006348	1.274	6.77e-05	intronic	JARID2	15334092	T	C	0.0980431	0.0496	0.0562	0.1808	0.0447	0.1207
7	62383062	rs1297477427	1.905	0.0002011	1.835	0.000511	1.07	0.01399	1.371	0.004759	intergenic	NONE;ZNF733P	NA	NA	NA	NA	NA	NA	NA	NA	NA
20	2632433	rs6050703	0.787	0.0002017	0.8125	0.0001805	0.326	0.03765	0.3827	0.03201	intronic	TMC2	2613079	C	A	0.785543	0.8571	0.7997	0.7126	0.7455	0.8415
11	47272151	rs10742799	-0.8022	0.0002021	-1.007	2.22e-05	-0.5282	0.0004809	-0.6559	0.0001649	intronic	MADD	47293702	T	C	0.484625	0.7262	0.3415	0.531	0.3072	0.4571
13	21544662	rs8000741	0.9003	0.0002025	1.079	3.92e-05	0.4895	0.00421	0.7506	0.0001496	intronic	MICU2	22118801	T	C	0.342851	0.6944	0.3617	0.1044	0.1889	0.4479
20	30641457	rs1169606381	-1.056	0.0002034	-1.124	0.0001537	-0.8826	3.25e-05	-1.012	3e-05	intergenic	FRG1BP;DEFB11	NA	NA	NA	NA	NA	NA	NA	NA	NA
3	85153501	rs7642449	-1.393	0.0002041	-1.442	0.0004182	-0.6233	0.002384	-1.237	2.58e-05	intronic	CADM2	85202651	A	G	0.251997	0.1855	0.0951	0.5772	0.0974	0.1513
17	43855461	rs4793041	0.96	0.0002057	1.125	3.06e-05	0.3706	0.06819	0.5377	0.01634	intronic	CD300LG	41932829	C	G	0.111422	0	0.1081	0.1415	0.1938	0.1033
17	43864420	rs231541	0.96	0.0002057	1.125	3.06e-05	0.3706	0.06819	0.5377	0.01634	downstream	CD300LG	41941788	C	G	0.88738	1	0.8919	0.8548	0.8072	0.8947
11	47264739	rs7120118	-0.8119	0.0002067	-0.9535	5.68e-05	-0.4889	0.001388	-0.6571	0.0002025	intronic	NR1H3	47286290	T	C	0.488019	0.7748	0.3401	0.5151	0.2972	0.4571
1	75481584	rs11163468	-0.837	0.0002068	-0.989	4e-05	-0.3646	0.0167	-0.6883	0.0001583	intronic	SLC44A5	75947269	G	A	0.669129	0.9444	0.4236	0.7965	0.4821	0.5798
8	40765000	rs59446867	0.9611	0.0002078	1.044	9.72e-05	0.438	0.02169	0.5376	0.01373	intronic	ZMAT4	40622519	TA	T	0.296326	0.6151	0.255	0.298	0.0696	0.228
13	21577295	rs7994033	0.8681	0.0002079	1.077	2.39e-05	0.5393	0.001104	0.7494	8.52e-05	intronic	MICU2	22151434	A	C	0.385783	0.6954	0.3862	0.2542	0.1889	0.4468
13	21599564	rs4770178	0.8681	0.0002079	1.077	2.39e-05	0.5393	0.001104	0.7494	8.52e-05	intronic	MICU2	22173703	A	C	0.384185	0.6885	0.3862	0.2526	0.1889	0.4479
13	21599656	rs4770179	0.8681	0.0002079	1.077	2.39e-05	0.5393	0.001104	0.7494	8.52e-05	intronic	MICU2	22173795	T	G	0.384185	0.6885	0.3862	0.2526	0.1889	0.4479
13	21600925	rs4770181	0.8681	0.0002079	1.077	2.39e-05	0.5393	0.001104	0.7494	8.52e-05	intronic	MICU2	22175064	T	C	0.385184	0.6885	0.3876	0.2557	0.1879	0.4489
3	85199327	rs9810830	-1.405	0.0002084	-1.582	0.0002641	-0.6261	0.002459	-1.244	2.67e-05	intronic	CADM2	85248477	C	T	0.251797	0.1875	0.098	0.5817	0.1024	0.135
1	75473009	rs1889037	-0.8274	0.0002086	-0.9212	8.27e-05	-0.3819	0.01214	-0.6659	0.0002035	intronic	SLC44A5	75938694	G	C	0.6873	0.9335	0.4236	0.848	0.506	0.59
9	10324342	rs7854874	0.7804	0.0002094	0.7686	0.0004325	0.6203	7e-05	0.6362	0.0002816	intronic	PTPRD	10324342	C	T	0.378794	0.2569	0.4035	0.6135	0.1968	0.3569
12	98172491	rs77561001	1.644	0.0002095	1.841	5.69e-05	1.045	0.006053	1.299	0.001372	intergenic	MIR4303;SLC9A7	98566269	T	A	0.0666933	0.0317	0.1988	0.0643	0.0129	0.0675
8	138234773	rs6577897	0.7129	0.0002096	0.7572	0.0001731	0.4328	0.002053	0.6101	0.0001606	intronic	FAM135B	139247016	G	T	0.317492	0.2401	0.3646	0.4175	0.3748	0.1697
17	10614714	rs72818717	0.9169	0.0002096	0.9368	0.0003166	0.3183	0.07931	0.4809	0.01934	ncRNA_intronic	MYHAS	10518031	C	T	0.229832	0.254	0.2176	0.4236	0.1103	0.0746
17	10614768	rs72818718	0.9169	0.0002096	0.9368	0.0003166	0.3183	0.07931	0.4809	0.01934	ncRNA_intronic	MYHAS	10518085	G	C	0.230032	0.254	0.2176	0.4244	0.1103	0.0746
17	10614785	rs12601954	0.9169	0.0002096	0.9368	0.0003166	0.3183	0.07931	0.4809	0.01934	ncRNA_intronic	MYHAS	10518102	T	C	0.230232	0.254	0.2176	0.4251	0.1103	0.0746
17	10615239	rs7207984	0.9169	0.0002096	0.9368	0.0003166	0.3183	0.07931	0.4809	0.01934	ncRNA_intronic	MYHAS	10518556	A	T	0.229034	0.256	0.2147	0.4206	0.1103	0.0746
6	151634346	rs11759768	-1.16	0.0002098	-1.269	0.000236	-0.7793	8.69e-05	-0.975	7.23e-05	intergenic	CCDC170;ESR1	151955481	C	G	0.157548	0.0258	0.1441	0.1649	0.2008	0.2485
1	89472766	rs6428547	-0.7956	0.0002104	-0.9155	8.03e-05	-0.3006	0.02899	-0.3199	0.04722	intergenic	GBP1P1;LRRC8B	89938325	T	C	0.64976	0.619	0.6628	0.7179	0.6262	0.6043
13	21492933	rs1044045	0.8588	0.0002106	1.069	2.4e-05	0.4636	0.005538	0.706	0.0002073	UTR3	MICU2	22067072	T	C	0.361621	0.6607	0.3775	0.1853	0.1988	0.4479
2	161041482	rs56060127	0.9061	0.0002107	0.8984	0.000549	0.7877	1.91e-05	0.8875	2.89e-05	intergenic	RBMS1;TANK	161897993	TCA	T	0.127196	0.248	0.0893	0.0129	0.1909	0.1186
6	24689083	rs4560629	0.8069	0.0002107	0.9383	4.14e-05	0.473	0.003509	0.5544	0.002704	intronic	ACOT13	24689311	T	C	0.755192	0.8433	0.7939	0.7852	0.659	0.6953
2	164688063	rs10601676	0.7495	0.0002118	0.8344	9.56e-05	0.5433	0.0002671	0.7009	3.54e-05	intronic	COBLL1	165544573	GAA	G	0.395168	0.0982	0.2565	0.7973	0.4433	0.2065
3	101080369	rs9881220	0.8286	0.000212	0.9206	9.61e-05	0.4618	0.005902	0.6124	0.00108	intergenic	ABI3BP;IMPG2	100799213	G	A	0.771965	0.881	0.7723	0.7738	0.6998	0.7311
5	38582063	rs77174713	3.039	0.0002132	3.2	0.0001091	1.18	0.1087	1.811	0.01913	ncRNA_intronic	LIFR-AS1	38582165	T	C	0.0189696	0.0367	0.0014	0.025	0	0.0245
11	39384211	rs1391590	0.9767	0.0002154	1.043	0.0001281	0.5217	0.0093	0.8019	0.0004109	intergenic	INC01493;LRRC4	39405761	C	A	0.792931	0.9325	0.8818	0.6717	0.8052	0.7372
20	29828725	rs1404643062	1.066	0.0002155	1.072	0.00038	0.8713	5.6e-05	0.8323	0.0007732	intergenic	AM242B;MIR447	NA	NA	NA	NA	NA	NA	NA	NA	NA
4	116191038	rs9995163	1.056	0.0002157	1.153	8.68e-05	0.7046	0.0008977	0.8854	0.0003153	intergenic	NDST4;MIR1973	117112194	A	T	0.235224	0.0913	0.1801	0.5477	0.1064	0.1329
2	164672366	rs13389219	0.737	0.0002175	0.7963	0.0001542	0.527	0.0003338	0.6589	7.54e-05	intronic	COBLL1	165528876	C	T	0.396565	0.0962	0.2579	0.7988	0.4354	0.2209
11	47320178	rs10838691	-0.8056	0.0002197	-0.9343	6.98e-05	-0.5309	0.0005285	-0.6611	0.0001681	intronic	MADD	47341729	T	C	0.495008	0.7262	0.3429	0.5688	0.3072	0.4581
7	51644113	rs11975223	1.482	0.00022	1.466	0.0004325	0.7711	0.01752	0.9923	0.009246	intergenic	07986794;POM1	51711809	G	A	0.0790735	0	0.0591	0.1982	0.0547	0.0389
7	98519381	rs57282732	-1.003	0.0002201	-0.8985	0.001238	-0.2748	0.08035	-0.4071	0.02971	intergenic	BAIAP2L1;NPTX2	98148693	G	A	0.228834	0.4256	0.2205	0.0552	0.2117	0.2843
2	161028371	rs4664394	0.904	0.0002202	0.8965	0.0005703	0.7771	2.38e-05	0.8689	3.89e-05	intergenic	RBMS1;TANK	161884882	G	C	0.127396	0.249	0.0893	0.0121	0.1909	0.1196
17	10616028	rs9902806	0.9152	0.000221	0.9349	0.0003353	0.3115	0.08655	0.4767	0.02067	ncRNA_intronic	MYHAS	10519345	A	G	0.240216	0.256	0.2176	0.4607	0.1113	0.0746
4	116191250	rs10018052	1.064	0.0002211	1.18	7.11e-05	0.6999	0.001198	0.9066	0.0002827	intergenic	NDST4;MIR1973	117112406	G	A	0.24401	0.0903	0.1844	0.6089	0.1064	0.093
4	116191380	rs12499869	1.064	0.0002211	1.18	7.11e-05	0.6954	0.001286	0.8976	0.000322	intergenic	NDST4;MIR1973	117112536	A	T	0.245607	0.0903	0.1844	0.615	0.1064	0.093
7	74967509	rs2523310	0.9605	0.0002217	0.9851	0.0002046	0.4804	0.0238	0.6616	0.004239	intronic	CASTOR2	NA	NA	NA	NA	NA	NA	NA	NA	NA
13	21569378	rs4770175	0.9167	0.0002219	1.092	4.66e-05	0.4875	0.006098	0.7622	0.0002241	intronic	MICU2	22143517	A	G	0.315895	0.6944	0.3473	0.0129	0.1839	0.4489
17	11450704	rs59717518	0.7201	0.0002228	0.8014	9.37e-05	0.3906	0.005817	0.5699	0.0004264	intronic	SHISA6	11354021	G	GA	0.3752	0.3681	0.2896	0.4516	0.2913	0.4264
20	29017988	rs1232337536	2.064	0.0002228	2.042	0.0003003	1.797	0.0002401	2.002	9.88e-05	intergenic	FRG1CP;FRG1DP	NA	NA	NA	NA	NA	NA	NA	NA	NA
4	116170142	rs1395429	1.042	0.0002229	1.153	7.49e-05	0.7559	0.0003736	0.8795	0.0002975	intergenic	NDST4;MIR1973	117091298	A	G	0.667532	0.9077	0.7853	0.1225	0.8837	0.8507
4	116171074	rs2048062	1.042	0.0002229	1.153	7.49e-05	0.7559	0.0003736	0.8795	0.0002975	intergenic	NDST4;MIR1973	117092230	T	C	0.667532	0.9077	0.7853	0.1218	0.8847	0.8507
2	161023627	rs59174355	0.901	0.0002236	0.8908	0.0006003	0.7669	2.79e-05	0.8645	4.11e-05	intergenic	RBMS1;TANK	161880138	C	T	0.129992	0.248	0.0922	0.0212	0.1909	0.1196
22	49839876	rs6009898	2.517	0.0002236	2.628	0.0001331	1.267	0.00809	1.901	0.001142	intergenic	BRD1;ZBED4	50233524	C	G	0.052516	0.0139	0.0245	0.1657	0.003	0.0102
11	47304582	rs58917953	-0.7961	0.0002237	-0.9293	6.54e-05	-0.5196	0.0005715	-0.6624	0.0001402	intronic	MADD	47326133	C	CTGTT	0.479233	0.7252	0.3357	0.5136	0.3072	0.4581
10	24505383	rs7907859	-0.753	0.0002249	-0.6545	0.002078	-0.4456	0.001273	-0.7622	7.02e-06	intronic	KIAA1217	24794312	G	A	0.645767	0.5804	0.5461	0.9614	0.5378	0.4683
6	106498915	rs12193525	0.8872	0.000225	0.8017	0.001336	0.245	0.158	0.338	0.09097	intronic	CRYBG1	106946790	G	A	0.247804	0.3998	0.1326	0.3185	0.164	0.1636
1	75375601	rs56996160	-0.8504	0.0002255	-0.9692	7.98e-05	-0.5418	0.0007786	-0.8644	1.01e-05	intronic	SLC44A5	75841286	TTCTATCACTGTT	T	0.735823	0.9722	0.6427	0.8616	0.5388	0.591
1	75388758	rs4949849	-0.8504	0.0002255	-0.9692	7.98e-05	-0.5319	0.0009941	-0.8661	9.77e-06	intronic	SLC44A5	75854443	A	G	0.73742	0.9722	0.6383	0.8684	0.5398	0.592
7	159037236	rs34876733	1.189	0.0002262	1.2	0.0002544	0.5936	0.02245	0.8295	0.003	intronic	VIPR2	158829927	C	T	0.0545128	0.0694	0.062	0.0038	0.0845	0.0716
1	55222870	rs67293523	1.726	0.0002264	1.841	7.52e-05	1.131	0.002438	1.353	0.0008994	ncRNA_intronic	MIR4422HG	55688543	C	A	0.0632987	0.0446	0.0303	0.1225	0.0398	0.0501
1	55223088	rs67175330	1.726	0.0002264	1.841	7.52e-05	1.087	0.003762	1.273	0.002004	ncRNA_intronic	MIR4422HG	55688761	C	T	0.0583067	0.0456	0.0303	0.1225	0.0398	0.0235
1	55223482	rs17131756	1.726	0.0002264	1.841	7.52e-05	1.087	0.003762	1.273	0.002004	ncRNA_intronic	MIR4422HG	55689155	A	T	0.058107	0.0446	0.0303	0.1225	0.0398	0.0235
1	55224574	rs67540256	1.726	0.0002264	1.841	7.52e-05	1.087	0.003762	1.273	0.002004	ncRNA_intronic	MIR4422HG	55690247	G	A	0.058107	0.0446	0.0303	0.1225	0.0398	0.0235
3	36117951	rs17240824	0.7845	0.000228	0.8742	9.4e-05	0.349	0.02993	0.4901	0.006523	intergenic	ARPP21;STAC	36159443	G	A	0.183506	0.0734	0.2133	0.1679	0.2982	0.1789
2	161033481	rs7568166	0.9003	0.000229	0.8905	0.0006074	0.7787	2.3e-05	0.8858	2.99e-05	intergenic	RBMS1;TANK	161889992	A	G	0.132788	0.249	0.0937	0.0303	0.1909	0.1196
1	82407208	rs147335646	3.48	0.0002291	3.38	0.0004585	1.195	0.1003	1.971	0.008147	intergenic	DGRL2;LINC0136	82872891	C	A	0.00698882	0.0337	0	0	0	0.001
2	161032879	rs7605436	0.8979	0.0002292	0.9028	0.000516	0.7774	2.3e-05	0.8659	4e-05	intergenic	RBMS1;TANK	161889390	T	G	0.127396	0.249	0.0893	0.0121	0.1909	0.1196
2	161041403	rs56191094	0.8979	0.0002292	0.9028	0.000516	0.7861	1.93e-05	0.8837	3.02e-05	intergenic	RBMS1;TANK	161897914	TC	T	0.126997	0.247	0.0893	0.0129	0.1909	0.1186
2	161041405	rs56200540	0.8979	0.0002292	0.9028	0.000516	0.7861	1.93e-05	0.8837	3.02e-05	intergenic	RBMS1;TANK	161897916	AT	A	0.126997	0.247	0.0893	0.0129	0.1909	0.1186
2	161042627	rs16845495	0.8979	0.0002292	0.9028	0.000516	0.7774	2.3e-05	0.8659	4e-05	intergenic	RBMS1;TANK	161899138	G	A	0.128395	0.247	0.0893	0.0182	0.1909	0.1186
13	21599398	rs9552458	0.8628	0.0002293	1.072	2.66e-05	0.5303	0.001352	0.7414	0.0001027	intronic	MICU2	22173537	T	A	0.384385	0.6885	0.3862	0.2534	0.1889	0.4479
13	21599952	rs4770180	0.8628	0.0002293	1.072	2.66e-05	0.5182	0.001726	0.7205	0.000156	intronic	MICU2	22174091	C	A	0.383786	0.6865	0.3862	0.2526	0.1889	0.4479
9	43181635	rs1285171929	-1.127	0.0002294	-1.186	0.0001981	-0.6958	0.001669	-1.005	8.01e-05	intergenic	C102724904;NO	NA	NA	NA	NA	NA	NA	NA	NA	NA
8	40764080	rs4288363	0.9467	0.0002303	1.054	7.91e-05	0.4465	0.01867	0.5459	0.01176	intronic	ZMAT4	40621599	C	T	0.296126	0.6171	0.255	0.2965	0.0686	0.228
8	40764264	rs10565040	0.9467	0.0002303	1.054	7.91e-05	0.4465	0.01867	0.5459	0.01176	intronic	ZMAT4	40621783	GAA	G	0.296326	0.6171	0.255	0.2965	0.0696	0.228
8	40766754	rs7008010	0.9467	0.0002303	1.054	7.91e-05	0.4465	0.01867	0.5459	0.01176	intronic	ZMAT4	40624273	C	T	0.296326	0.6181	0.255	0.2965	0.0686	0.228
8	40768287	rs7846312	0.9467	0.0002303	1.054	7.91e-05	0.4643	0.01477	0.5459	0.01176	intronic	ZMAT4	40625806	C	T	0.296526	0.6171	0.255	0.2965	0.0686	0.2301
8	40768617	rs7812360	0.9467	0.0002303	1.054	7.91e-05	0.4643	0.01477	0.5459	0.01176	intronic	ZMAT4	40626136	C	T	0.296526	0.6171	0.255	0.2965	0.0686	0.2301
17	21841309	rs1157112523	-1.148	0.0002303	-1.152	0.0004935	-0.9397	7.32e-06	-0.9664	8.92e-05	intergenic	KCNJ18;UBBP4	NA	NA	NA	NA	NA	NA	NA	NA	NA
4	94302856	rs17021459	1.242	0.0002313	1.282	0.0003876	0.3177	0.1728	0.4928	0.06401	intronic	HPGDS	95224007	A	G	0.0882588	0.2004	0.183	0.0015	0.0129	0.1002
3	85204831	rs7637886	-1.403	0.0002315	-1.579	0.0003056	-0.5422	0.007551	-1.173	5.44e-05	intronic	CADM2	85253981	C	A	0.243211	0.1875	0.0965	0.5522	0.0994	0.135
21	30322146	rs2832794	0.744	0.0002327	0.7544	0.0003348	0.1821	0.198	0.4255	0.01001	intergenic	TAP26-1;KRTAP2	31694464	C	T	0.319489	0.2649	0.3458	0.1762	0.4463	0.4202
10	38635806	rs368957306	1.013	0.0002331	1.171	6.31e-05	0.6242	0.001294	0.9735	1.75e-05	intergenic	NC00999;ACTR3B	NA	NA	NA	NA	NA	NA	NA	NA	NA
7	62301557	rs201592324	-1.208	0.0002336	-1.313	8.55e-05	-0.8773	0.0005511	-1.096	0.0001031	intergenic	NONE;ZNF733P	NA	NA	NA	NA	NA	NA	NA	NA	NA
20	30855980	rs1159436908	0.9956	0.0002336	1.101	0.0001051	0.8679	1.11e-05	0.9886	1.24e-05	intergenic	FRG1BP;DEFB11	NA	NA	NA	NA	NA	NA	NA	NA	NA

20	29810582	rs1466076649	1.028	0.0002339	0.9555	0.00118	0.8854	2.91e-05	0.9408	7.35e-05	intergenic	M242B;MIR447	NA	NA	NA	NA	NA	NA	NA	NA	NA
7	123441352	rs36097906	1.027	0.0002342	0.977	0.0008473	0.456	0.02781	0.8089	0.0004495	intergenic	SLC13A1;IQUB	123081406	A	AG	0.159145	0.0556	0.1009	0.2156	0.1431	0.2474
2	161041514	rs55876040	0.9	0.0002343	0.8931	0.0005956	0.7725	2.68e-05	0.8637	4.31e-05	intergenic	RBMS1;TANK	161898025	A	ACACACACACACAC	0.107428	0.2044	0.0793	0.0166	0.162	0.0941
7	97929234	rs1456793292	2.445	0.0002351	2.614	7.54e-05	1.577	0.006815	1.947	0.001108	ncRNA_intronic	CZ1P-ASNS	NA	NA	NA	NA	NA	NA	NA	NA	NA
7	97929236	rs1239107552	2.445	0.0002351	2.614	7.54e-05	1.654	0.004256	1.947	0.001108	ncRNA_intronic	CZ1P-ASNS	NA	NA	NA	NA	NA	NA	NA	NA	NA
20	47259646	rs11696358	1.284	0.0002354	1.242	0.0006689	1.139	7.85e-05	1.17	0.0001926	intronic	ZMYND8	45888390	C	G	0.0345447	0	0.0562	0.0053	0.1093	0.0174
17	8775093	rs4363889	-0.826	0.0002359	-0.9965	4.43e-05	-0.1966	0.1714	-0.3607	0.03412	intergenic	SPDYE4;MFSD6L	8678411	T	C	0.311701	0.3234	0.3329	0.0772	0.4284	0.4816
1	200623147	rs2808248	-0.8477	0.0002361	-0.7576	0.001391	-0.338	0.02099	-0.5349	0.00224	intergenic	KIF14;DDX59	200592275	T	C	0.721246	0.7173	0.7594	0.8328	0.5755	0.6973
1	200627582	rs34230408	-0.8477	0.0002361	-0.7576	0.001391	-0.338	0.02099	-0.5349	0.00224	intergenic	KIF14;DDX59	200596710	T	TA,TAA	17452,0.000798	0.7143,0	0.7579,0	0.8222,0.003	0.5755,0	0.6963,0
5	128049169	rs1234527	0.7079	0.0002362	0.7479	0.000246	0.5642	4.85e-05	0.5217	0.001025	ncRNA_intronic	LINC01184	127384861	T	C	0.621406	0.7014	0.5634	0.7746	0.4215	0.5787
1	75373613	rs1614816	-0.84	0.0002374	-0.9604	8.13e-05	-0.5514	0.0005752	-0.859	9.78e-06	intronic	SLC44A5	75839298	C	G	0.73123	0.9722	0.6412	0.8434	0.5398	0.592
1	75394691	rs1249815	-0.84	0.0002374	-0.9604	8.13e-05	-0.5405	0.0007538	-0.859	9.78e-06	intronic	SLC44A5	75860376	G	A	0.73103	0.9722	0.6383	0.8442	0.5398	0.592
1	75397404	rs1249811	-0.84	0.0002374	-0.9604	8.13e-05	-0.5405	0.0007538	-0.859	9.78e-06	intronic	SLC44A5	75863089	G	A	0.73143	0.9722	0.6383	0.8442	0.5398	0.5941
19	1729371	rs11320362	-0.7408	0.0002374	-0.7451	0.0004029	-0.448	0.001244	-0.6489	9.45e-05	intergenic	TCF3;ONECUT3	1729370	TG	T	0.477835	0.3819	0.4669	0.4039	0.5278	0.6329
11	69992310	rs61885151	0.9895	0.0002377	0.8466	0.002743	0.3944	0.05737	0.6918	0.002766	intronic	ANO1	69838416	T	C	0.0926518	0.1329	0.0807	0.0923	0.1113	0.0409
11	69992644	rs17160521	0.9895	0.0002377	0.8466	0.002743	0.3944	0.05737	0.6918	0.002766	intronic	ANO1	69838750	T	C	0.0926518	0.1329	0.0807	0.0923	0.1113	0.0409
13	21514062	rs9550742	0.8765	0.0002381	1.049	4.69e-05	0.5023	0.003224	0.7573	0.0001083	intronic	MICU2	22088201	T	C	0.347444	0.6944	0.366	0.112	0.1978	0.4489
14	76848830	rs4903498	0.8203	0.0002386	0.9338	8.42e-05	0.551	0.001343	0.6283	0.001034	intronic	LRRC74A	77315173	C	T	0.239617	0.005	0.1916	0.357	0.2893	0.3057
2	181703745	rs116919931	3.126	0.0002394	3.491	0.0001102	1.349	0.05656	1.951	0.007858	intergenic	NEUROD1;ITPRID	182568472	G	A	0.0155751	0.0585	0.0014	0.0129	0	0.001
7	123751005	rs35590644	1.252	0.0002396	1.248	0.0005558	0.8663	0.001331	1.121	0.0001052	intergenic	WASL;RNU6-1	123391059	C	T	0.0680911	0.0377	0.072	0.0076	0.1113	0.1339
1	200689275	rs61825138	-0.9241	0.0002404	-0.8615	0.0009524	-0.3368	0.02693	-0.6009	0.00127	ncRNA_intronic	DDX59-AS1	200658403	T	C	0.255192	0.2917	0.2233	0.1475	0.3748	0.2628
3	101076735	rs9824042	0.837	0.0002405	0.9445	8.43e-05	0.4401	0.008845	0.5855	0.001956	intergenic	ABI3BP;IMPG2	100795579	C	T	0.751597	0.8869	0.7666	0.7035	0.6918	0.728
6	151639794	rs67413479	-1.15	0.0002413	-1.284	0.0002016	-0.7868	7.59e-05	-0.9707	7.81e-05	intergenic	CCDC170;ESR1	151960929	G	A	0.166334	0.0278	0.147	0.1952	0.2008	0.2485
20	29360732	rs1247866097	-1.02	0.0002414	-1.004	0.000534	-0.7876	4.74e-05	-0.7755	0.0005512	intergenic	FRG1DP;FRG2EP	NA	NA	NA	NA	NA	NA	NA	NA	NA
4	189747117	rs138960177	1.605	0.000242	1.69	0.0001558	0.9452	0.01489	1.05	0.01213	intergenic	INC01262;FRG1-	190668271	C	T	0.0203674	0	0.0159	0.003	0.0477	0.0399
4	189988453	rs79958344	-1.005	0.0002424	-1.193	3.8e-05	-0.6873	0.0004969	-0.7614	0.0007725	intergenic	FRG1;FRG2	NA	NA	NA	NA	NA	NA	NA	NA	NA
9	33799873	rs1010108612	-1.194	0.0002424	-1.073	0.001392	-0.91	2.87e-05	-1.065	5.29e-05	downstream	PRSS3	NA	NA	NA	NA	NA	NA	NA	NA	NA
14	28260444	rs8020736	-0.8039	0.0002426	-0.7818	0.0006193	-0.583	7.78e-05	-0.5738	0.0009183	intergenic	C728755;FOXG1-	28729650	A	G	0.604233	0.748	0.4078	0.8109	0.503	0.4202
14	28260594	rs11383149	-0.8039	0.0002426	-0.7818	0.0006193	-0.583	7.78e-05	-0.5738	0.0009183	intergenic	C728755;FOXG1-	28729800	T	TA	0.604433	0.748	0.4078	0.8109	0.504	0.4202
16	84406705	rs4782958	0.7488	0.000243	0.8074	0.0002207	0.2709	0.05076	0.5056	0.002298	intronic	ATP2C2	84440311	G	C	0.428914	0.38	0.5533	0.1483	0.5606	0.635
1	55225458	rs58472471	1.719	0.0002439	1.834	8.15e-05	1.078	0.004019	1.266	0.002118	ncRNA_intronic	MIR4422HG	55691131	G	A	0.0539137	0.0446	0.0303	0.1067	0.0398	0.0235
11	47256866	rs3758672	-0.8013	0.000244	-0.9411	6.89e-05	-0.4729	0.001919	-0.6426	0.0002694	intronic	NR1H3	47278417	A	G	0.483027	0.7748	0.3357	0.4985	0.2972	0.4571
11	47260229	rs2279239	-0.8013	0.000244	-0.9411	6.89e-05	-0.4729	0.001919	-0.6426	0.0002694	intronic	NR1H3	47281780	T	C	0.483427	0.7748	0.3357	0.5	0.2972	0.4571
11	47265215	rs11372504	-0.8013	0.000244	-0.9411	6.89e-05	-0.4729	0.001919	-0.6426	0.0002694	intronic	NR1H3	47286766	A	AC	0.483626	0.7758	0.3357	0.5	0.2972	0.4571
22	44122009	rs7291712	0.6979	0.000244	0.656	0.0007973	0.4137	0.002883	0.6166	0.0001239	intronic	PARVB	44517889	T	C	0.317692	0.2976	0.4308	0.2784	0.3628	0.2648
11	47293452	rs3781621	-0.793	0.0002453	-0.9251	7.3e-05	-0.5185	0.0005953	-0.6555	0.0001686	intronic	MADD	47315003	T	C	0.480032	0.7252	0.3357	0.5159	0.3082	0.4581
16	50954299	rs1876367	0.8982	0.0002453	0.9437	0.0001979	0.6389	0.0003845	0.8157	5.7e-05	ncRNA_intronic	MIR548AI	50988210	G	A	0.183506	0.2698	0.147	0.0295	0.2087	0.3027
16	50973097	rs41505946	0.8982	0.0002453	0.9437	0.0001979	0.6389	0.0003845	0.8157	5.7e-05	ncRNA_intronic	MIR548AI	51007008	G	A	0.194089	0.2728	0.1427	0.0325	0.2078	0.3538
16	50973448	rs4423423	0.8982	0.0002453	0.9437	0.0001979	0.6389	0.0003845	0.8157	5.7e-05	ncRNA_intronic	MIR548AI	51007359	G	A	0.194089	0.2728	0.1427	0.0325	0.2078	0.3538
15	80425717	rs11462589	2.071	0.0002455	2.03	0.0004661	0.8205	0.03667	1.216	0.00787	intronic	ARNT2	80718059	G	GT	0.0698882	0.0645	0.0101	0.143	0.0159	0.0746
6	151635490	rs12203668	-1.149	0.0002461	-1.283	0.0002046	-0.7859	7.69e-05	-0.9739	7.47e-05	intergenic	CCDC170;ESR1	151956625	A	G	0.166134	0.0268	0.147	0.1952	0.2008	0.2485
11	47302921	rs7127907	-0.8182	0.0002463	-0.9514	6.79e-05	-0.5209	0.0008144	-0.6199	0.0005074	intronic	MADD	47324472	A	C	0.479633	0.7262	0.3372	0.5136	0.3072	0.4581
14	27531767	rs6575694	-0.7938	0.0002463	-0.8738	0.0001534	-0.307	0.02685	-0.2899	0.07247	ncRNA_intronic	LOC728755	28000973	G	C	0.470847	0.3958	0.621	0.2829	0.6183	0.544
20	2638137	rs6076382	0.7705	0.0002465	0.7872	0.0002804	0.3339	0.03331	0.3952	0.02691	intronic	TMC2	2618783	C	T	0.778554	0.8571	0.7983	0.6815	0.7515	0.8425
11	47295017	rs113012334	-0.7926	0.0002467	-0.9208	7.57e-05	-0.5023	0.0008379	-0.6521	0.0001837	intronic	MADD	47316568	G	GT	0.334065	0.4484	0.2334	0.3684	0.2366	0.3415
15	63867905	rs4777370	0.9128	0.0002467	0.9994	0.0001139	0.3942	0.03938	0.6198	0.004085	intergenic	HERC1;MIR422A	64160104	G	A	0.787939	0.9663	0.7622	0.5726	0.838	0.862
23	86421871	rs1894611	0.7357	0.0002467	0.7559	0.0003681	0.4356	0.003463	0.6894	4.29e-05	intronic	DACH2	NA	NA	NA	NA	NA	NA	NA	NA	NA
13	66130683	rs11842950	2.076	0.0002469	2.22	9.07e-05	1.414	0.006279	1.75	0.001119	intergenic	IR548X2;MIR47	66704815	T	A	0.0119808	0	0.0173	0.0083	0.0239	0.0133
19	4222110	rs112335738	2.349	0.000247	2.292	0.0003931	1.448	0.002101	1.948	0.0001884	intronic	ANKRD24	4222107	C	T	0.0682907	0.002	0.0375	0.1815	0.0219	0.0532
15	85842591	rs78723271	0.9412	0.000248	0.843	0.001831	0.2818	0.1353	0.3769	0.08633	intergenic	MIR548AP;AGBL1	86385822	G	T	0.206669	0.2401	0.1585	0.2383	0.2316	0.138
9	33799874	rs1029570877	-1.189	0.0002483	-1.066	0.001448	-0.8654	6.18e-05	-1.075	4.47e-05	downstream	PRSS3	NA	NA	NA	NA	NA	NA	NA	NA	NA
8	133168593	rs117927451	2.611	0.0002494	2.75	9.9e-05	1.974	0.002656	2.275	0.0007035	intergenic	TG;CCN4	134180837	C	T	0.00579073	0	0.0043	0.0008	0.0229	0.002
3	36068283	rs10865869	0.7399	0.0002507	0.8344	0.0001063	0.3305	0.02621	0.4668	0.005538	intergenic	ARPP21;STAC	36109775	T	C	0.549321	0.8859	0.5706	0.1165	0.5964	0.7239
11	47313524	rs10838690	-0.7979	0.0002518	-0.9331	7.49e-05	-0.5034	0.0009115	-0.6534	0.0001917	intronic	MADD	47335075	A	G	0.479233	0.7262	0.3357	0.5129	0.3072	0.4581
8	40762716	rs55671832	0.9418	0.0002538	1.049	8.81e-05	0.4541	0.01706	0.5393	0.01287	intronic	ZMAT4	40620235	T	G	0.299521	0.6181	0.255	0.3071	0.0696	0.229
8	40762837	rs60343283	0.9418	0.0002538	1.049	8.81e-05	0.4363	0.02148	0.5393	0.01287	intronic	ZMAT4	40620356	G	T	0.299121	0.6171	0.255	0.3071	0.0696	0.228
8	40763784	rs62642748	0.9418	0.0002538	1.049	8.81e-05	0.4363	0.02148	0.5393	0.01287	intronic	ZMAT4	40621303	T	C	0.299121	0.6171	0.255	0.3071	0.0696	0.228
8	40763837	rs4430079	0.9418	0.0002538	1.049	8.81e-05	0.4363	0.02148	0.5393	0.01287	intronic	ZMAT4	40621356	G	A	0.299121	0.6171	0.255	0.3071	0.0696	0.228
13	66134687	rs11838913	2.071	0.000254	2.215	9.35e-05	1.406	0.006603	1.747	0.001147	intergenic	IR548X2;MIR47	66708819	T	C	0.0119808	0	0.0173	0.0083	0.0239	0.0133
15	82819949	rs145451188	1.827	0.0002544	2.021	5.68e-05	1.294	0.003005	1.325	0.005205	intronic	WHAMM	83488701	G	A	0.0123802	0	0.0144	0.0008	0.0437	0.0072
13	66068808	rs73201719	2.068	0.0002555	2.215	9.35e-05	1.411	0.00644	1.76	0.001069	intergenic	IR548X2;MIR47	66642940	C	T	0.0125799	0	0.0187	0.0083	0.0258	0.0133
13	66098549	rs144191935	2.068	0.0002555	2.215	9.35e-05	1.406	0.00665	1.75	0.001143	intergenic	IR548X2;MIR47	66672681	ATTGT	A	0.0123802	0	0.0173	0.0083	0.0258	0.0133
4	6407773	rs56217739	-0.8943	0.0002565	-1.132	4.46e-05	-0.6639	5.2e-05	-0.6566	0.0006874	intronic	PPP2R2C	6409500	A	G	0.342851	0.6905	0.1628	0.2799	0.2346	0.3088
4	116170867	rs6818257	1.029	0.0002565	1.159	7.01e-05	0.7487	0.0004202	0.8816	0.0002964	intergenic	NDST4;MIR1973	117092023	G	A	0.667532	0.9077	0.7853	0.1218	0.8847	0.8507
22	12044459	rs1313570056	-1.669	0.000258	-1.909	0.0004153	-1.166	5.56e-06	-1.067	0.0003934	intergenic	C102723769;NO	NA	NA	NA	NA	NA	NA	NA	NA	NA
23	42732394	rs62588241	0.8038	0.0002584	0.7884	0.0006492	0.3723	0.01442	0.5545	0.001472	intergenic	CASK;PPP1R2C	NA	NA	NA	NA	NA	NA	NA	NA	NA
6	151649694	rs12206977	-1.169	0.0002586	-1.298	0.0002635	-0.8437	3.8e-05	-0.9932	7.8e-05	intergenic	CCDC170;ESR1	151970829	G	T	0.166334	0.0298	0.1369	0.2133	0.1879	0.2423
2	107240789	rs115836693	1.142	0.0002587	1.011	0.001813	0.548	0.04206	0.6156	0.03337	ncRNA_intronic	MIR548AU	107857245	C	T	0.0375399	0.001	0.0562	0.0023	0.0845	0.0613
6	151638114	rs10872677	-1.142	0.0002589	-1.277	0.0002134	-0.788	7.34e-05	-0.9709	7.71e-05	intergenic	CCDC170;ESR1	151959249	C	T	0.157947	0.0268	0.1441	0.1657	0.2008	0.2485
4	116179781	rs1567091	1.036	0.000259	1.149	8.62e-05	0.77	0.0003179	0.8961	0.0002535	intergenic	NDST4;MIR1973	117100937	A	G	0.667931	0.9077	0.7839	0.1233	0.8857	0.8507
20	29087113	rs1239021955	-0.9913	0.0002594	-1.114	9.6e-05	-0.7877	7.08e-05	-0.7212	0.001456	ncRNA_intronic	FRG1DP	NA	NA	NA	NA	NA	NA	NA	NA	NA
8	140008599	rs6988665	0.8455	0.0002598	0.9441	9.09e-05	0.5875	0.0007238	0.7032	0.0003307	intronic	TRAPPC9	141018696	T	C	0.324681	0.0913	0.2262	0.7443	0.2127	0.183
2	161031196	rs7600920	0.8886	0.0002601	0.8908	0.0006003	0.7609	3.16e-05	0.8564	4.72e-05	intergenic	RBMS1;TANK	161887707	T	A	0.130192	0.249	0.0922	0.0212	0.1909	0.1196
17	43862992	rs231543	0.9177	0.0002602	1.074	4.7e-05	0.3404	0.07096	0.4814	0.02522	UTR3	CD300LG	41940360	G	T	0.783746	0.999	0.8602	0.5	0.7913	0.8834
2	131156	rs78041081	2.262	0.0002606	2.38	0.0001042	1.518	0.005272	1.791	0.001414	intergenic	FAM110C;SH3YL1	131156	A	G	0.00838658	0	0.0115	0.0008	0.0308	0.002
2	269819	rs2952781	2.262	0.0002606	2.38	0.0001042	1.518	0.005272	1.791	0.001414	intronic	ACP1	269819	C	T	0.00678914	0	0.0101	0	0.0258	0.001
13	21557304	rs9552450	0.907	0.0002616	1.083	5.5e-05	0.4674	0.008466	0.7333	0.0003577	intronic	MICU2	22131443	A	C	0.316094	0.6944	0.3473	0.0136	0.1849	0.4479
10	38666388	rs1348428265	-1.334	0.0002625	-1.692	0.0001071	-0.9603	2.86e-05	-0.9654	0.0003504	intergenic	NC00999;ACTR3B	NA	NA	NA	NA	NA	NA	NA	NA	NA
7	123513057	rs1525621	1.047	0.0002629	0.9736	0.001224	0.5297	0.0143	0.8581	0.0003081	intronic	IQUB	123153111	T	C	0.0980431	0.0536	0.0821	0.0439	0.1421	0.183
6	151633524	rs11753383	-1.055	0.0002642	-1.267	0.0001153	-0.7735	5.37e-05	-0.8762	0.0001349	intergenic	CCDC170;ESR1	151954659	G	A	0.172724	0.0268	0.1729	0.1959	0.2127	0.2505
19	1010397	rs77868901	2.197	0.000266	2.148	0.0003862	1.281	0.02129	1.545	0.007461	exonic	TMEM259	1010396	G	C	0.0309505	0.0169	0.0058	0.0136	0.0457	0.0716
15	85842573	rs74024931	0.9362	0.0002671	0.8389	0.001947	0.2714	0.148	0.3445	0.1145	intergenic	MIR548AP;AGBL1	86385804	G	C	0.20647	0.2391	0.1585	0.2383	0.2316	0.138
14	27528973	rs2154408	-0.8291	0.0002672	-0.8437	0.0003883	-0.2868	0.04421	-0.2629	0.1108	ncRNA_intronic	LOC728755	27998179	A	C	0.544928	0.3958	0.6729	0.5068	0.6402	0.5613
1	5673113	rs113255040	1.394	0.0002677	1.606	3.49e-05	1.028	0.001655	1.161	0.0009688	intergenic	AJAP1;MIR4689	NA	NA	NA	NA	NA	NA	NA	NA	NA
3	36118839	rs6550394	0.7749	0.0002677	0.8602	0.0001183	0.3317	0.03879	0.4624	0.01012	intergenic	ARPP21;STAC	36160331	A	T	0.175719	0.0734	0.2089	0.1407	0.2982	0.1789
17	21695920	rs1243865479	-0.9948	0.0002689	-0.9399	0.0009365	-0.7077	0.0002225	-0.9504	2.46e-05	intronic	KCNJ18	NA	NA	NA	NA	NA	NA	NA	NA	NA
2	212491611	rs10206719	0.8899	0.0002694	0.9566	0.0001599	0.6852	0.0002285	0.8397	7.87e-05	intronic	ERBB4	213356335	C	T	0.322484	0.0942	0.255	0.6876	0.1789	0.2597
13	112920393	rs9577400	3.763	0.0002707	3.947	0.0001928	1.534	0.07706	2.066	0.02263	intergenic	TP11A;MCF2L-AS	113574707	G	A	0.0103834	0.0516	0	0	0	0
13	21527552	rs9552445	0.902	0.0002715	1.117	3.49e-05	0.4704	0.008301	0.7506	0.0002401	intronic	MICU2	22101691	A	G	0.314896	0.6944	0.3473	0.0129	0.1829	0.4448
11	45717812	rs11038621	1.034	0.0002722	1.182	5.07e-05	0.5373	0.01264	0.6573	0.006733	intergenic	7154;LOC100507	45739362	C	A	0.155152	0.1875	0.2219	0.1755	0.0974	0.1063
19	14018096	rs73519619	1.253	0.0002722	1.392	8.6e-05	0.5921	0.02556	0.8904	0.002529	intergenic	RFX1;RLN3	14128908	A	C	0.19988	0.0536	0.0764	0.5741	0.0497	0.0869
20	2635910	rs910271	0.7614	0.0002726	0.8348	0.0001241	0.3429	0.0289	0.3977	0.02589	intronic	TMC2	2616556	T	C	0.786542	0.8571	0.7997	0.711	0.7515	0.8425
17	26950028	rs75028706	1.1	0.0002728	1.144	0.0003776	0.7517	0.0001638	0.9936	3.9e-05	intergenic	ONE;LOC1053717	NA	NA	NA	NA	NA	NA	NA	NA	NA
21	30299104	rs2832772	0.723	0.0002734	0.7245	0.0004451	0.2093	0.1296	0.4427	0.006449	intergenic	TAP25-1;KRTAP2	31671422	G	A	0.342652	0.3869	0.353	0.1596	0.4483	0.4284
21	30300047	rs8134219	0.723	0.0002734	0.7245	0.0004451	0.2093	0.1296	0.4427	0.006449	intergenic	TAP25-1;KRTAP2	31672365	C	T	0.342851	0.3869	0.353	0.1604	0.4483	0.4284
19	254714	rs200745972	-1.128	0.0002737	-1.016	0.001467	-0.5802	0.003292	-0.9767	7.07e-05	intergenic	LINC01002;PLPP2	NA	NA	NA	NA	NA	NA	NA	NA	NA
1	212468923	rs73088566	-0.7659	0.0002741	-0.8692	0.0001196	-0.6106	2.75e-05	-0.6671	8.04e-05	intergenic	NENF;LINC01740	212642265	T	A	0.600639	0.6885	0.5317	0.7602	0.502	0.4448
22	37331759	rs62237460	1.478	0.0002749	1.471	0.0004737	0.8708	0.0128	0.9566	0.0133	intergenic	TH4;LOC1005062	37727800	G	T	0.0239617	0	0.0461	0.0045	0.0616	0.0204
11	47304522	rs12575456	-0.7691	0.0002752	-0.8964	8.18e-05	-0.4928	0.0009148	-0.6281	0.0002459	intronic	MADD	47326073	G	A	0.479433	0.7262	0.3357	0.5136	0.3072	0.4581

13	21491388	rs9552437	0.8595	0.0002758	0.992	9.63e-05	0.4218	0.01421	0.7189	0.0002974	intergenic	ZDHHC20;MICU2	22065527	T	C	0.363219	0.6607	0.3761	0.1944	0.1958	0.4479
17	10615518	rs7207894	0.8997	0.0002773	0.9199	0.0004121	0.2947	0.104	0.4638	0.02415	ncRNA_intronic	MYHAS	10518835	C	T	0.236821	0.256	0.2161	0.4493	0.1103	0.0746
20	28784603	rs1285684358	-1.213	0.0002782	-1.122	0.001171	-0.9819	6.1e-06	-1.022	7.63e-05	intergenic	FRG1CP;FRG1DP	NA	NA	NA	NA	NA	NA	NA	NA	NA
2	161045437	rs4664397	0.8846	0.0002786	0.8912	0.0005195	0.8017	9.62e-06	0.9032	1.46e-05	intergenic	RBMS1;TANK	161901948	G	A	0.127596	0.25	0.0865	0.0091	0.1958	0.1207
11	47322404	rs10769252	-0.7769	0.0002787	-0.9704	3.52e-05	-0.5127	0.0006538	-0.6331	0.0002468	intronic	MADD	47343955	A	G	0.483427	0.7262	0.3372	0.5242	0.3072	0.4632
1	3975101	rs10799225	0.8831	0.0002798	1.064	3.6e-05	0.422	0.01774	0.6539	0.001526	intergenic	NC01346;LINC017	4035161	G	C	0.836462	0.996	0.8559	0.8903	0.6918	0.7342
6	70617353	rs35481020	0.7258	0.0002802	0.9507	1.13e-05	0.3473	0.01286	0.4006	0.01173	intergenic	SDHAF4;SMAP1	71327056	CA	C	0.664337	0.5198	0.7723	0.8434	0.5825	0.5787
6	71937148	rs9350445	0.7699	0.0002804	0.7728	0.0005836	0.6199	9.96e-05	0.7754	2.56e-05	intronic	RIMS1	72646851	A	C	0.800519	0.9891	0.7622	0.8495	0.6292	0.7434
1	75473113	rs1867352	-0.8088	0.0002807	-0.9119	0.0001001	-0.3903	0.01054	-0.6657	0.0001974	intronic	SLC44A5	75938798	G	A	0.670128	0.9296	0.4078	0.8298	0.4702	0.5787
11	71526049	rs1034377547	0.9846	0.0002812	1.141	6.93e-05	0.3317	0.1018	0.4504	0.05055	intergenic	ADSYN1;KRTAP5-	NA	NA	NA	NA	NA	NA	NA	NA	NA
14	90864310	rs8005327	0.8008	0.0002816	0.8974	8.41e-05	0.5097	0.00222	0.6674	0.0004532	UTR3	RPS6KA5	91330654	T	G	0.188299	0.1458	0.2968	0.1377	0.2237	0.1871
11	47254331	rs35879051	-0.7959	0.0002819	-0.9348	8.11e-05	-0.4761	0.001834	-0.6429	0.0002761	intronic	NR1H3	47275882	C	G	0.483427	0.7748	0.3357	0.5	0.2972	0.4571
20	28869722	rs1198223752	1.189	0.0002825	1.346	6.49e-05	0.8992	0.0007793	1.211	2.67e-05	intergenic	FRG1CP;FRG1DP	NA	NA	NA	NA	NA	NA	NA	NA	NA
14	28260394	rs9672035	-0.7898	0.0002835	-0.7818	0.0006193	-0.5791	8.51e-05	-0.5679	0.001013	intergenic	C728755;FOXG1-	28729600	G	A	0.603834	0.748	0.4078	0.8101	0.502	0.4202
4	6406996	rs12642310	-0.8878	0.0002836	-1.101	7.01e-05	-0.6669	4.8e-05	-0.6639	0.0006323	intronic	PPP2R2C	6408723	T	G	0.347444	0.6974	0.1599	0.2897	0.2346	0.3139
4	116174867	rs6857562	1.02	0.0002839	1.153	7.49e-05	0.7623	0.0003388	0.8687	0.0003452	intergenic	NDST4;MIR1973	117096023	C	T	0.667532	0.9077	0.7853	0.1218	0.8847	0.8507
4	116174880	rs6834686	1.02	0.0002839	1.153	7.49e-05	0.7481	0.0004212	0.8687	0.0003452	intergenic	NDST4;MIR1973	117096036	T	A	0.667532	0.9077	0.7853	0.1218	0.8847	0.8507
11	47256101	rs4752822	-0.7948	0.0002839	-0.9354	7.97e-05	-0.4631	0.002409	-0.6361	0.0003181	intronic	NR1H3	47277652	T	C	0.483427	0.7748	0.3357	0.5	0.2972	0.4571
4	68806458	rs114604606	-1.019	0.0002843	-1.147	0.0001247	-0.8133	2.79e-05	-0.9321	4.19e-05	intergenic	JGT2B15;UGT2B1	NA	NA	NA	NA	NA	NA	NA	NA	NA
14	27463132	rs1954668	-0.79	0.0002855	-0.8669	0.000187	-0.2787	0.04729	-0.2821	0.08238	ncRNA_intronic	LOC728755	27932338	A	G	0.458666	0.3929	0.6225	0.2315	0.6342	0.5368
6	151635755	rs11155806	-1.135	0.0002868	-1.269	0.000236	-0.7717	0.0001023	-0.9626	8.93e-05	intergenic	CCDC170;ESR1	151956890	C	A	0.157748	0.0268	0.1441	0.1649	0.2008	0.2485
6	151639470	rs12197785	-1.135	0.0002868	-1.269	0.000236	-0.7717	0.0001023	-0.9626	8.93e-05	intergenic	CCDC170;ESR1	151960605	C	G	0.155351	0.0268	0.1427	0.1566	0.2008	0.2485
19	1013348	rs112496473	2.18	0.0002869	2.136	0.000404	1.273	0.02198	1.533	0.007824	intronic	TMEM259	1013347	C	A	0.0317492	0.0169	0.0072	0.0151	0.0467	0.0716
1	75445428	rs2347218	-0.8326	0.0002872	-0.9538	9.85e-05	-0.5229	0.00119	-0.8496	1.34e-05	intronic	SLC44A5	75911113	A	G	0.72524	0.9722	0.6354	0.8033	0.5398	0.6196
16	84208754	rs2042409	0.7258	0.0002901	0.8746	4.89e-05	0.2444	0.07784	0.5598	0.0006811	intergenic	ADAD2;KCNG4	84242360	C	T	0.436102	0.5278	0.5086	0.4274	0.3748	0.365
11	102805406	rs634607	0.7191	0.0002904	0.7687	0.0002541	0.3253	0.02328	0.4618	0.004414	ncRNA_intronic	WTAPP1	102676137	A	T	0.372204	0.3224	0.4481	0.2595	0.4205	0.4724
7	51640420	rs10240369	1.459	0.000291	1.441	0.000581	0.7123	0.02675	0.9509	0.01257	intergenic	07986794;POM1	51708116	C	T	0.0782748	0	0.0591	0.1974	0.0517	0.0389
11	69968723	rs7926926	1.023	0.0002918	0.9202	0.001638	0.2761	0.1872	0.5643	0.01982	intergenic	FGF3;ANO1	69814829	T	C	0.844649	0.9028	0.915	0.6286	0.9095	0.9601
21	30298764	rs34176987	0.7195	0.0002922	0.7211	0.0004733	0.2043	0.1393	0.4379	0.007055	intergenic	TAP25-1;KRTAP2	31671082	GT	G	0.34345	0.3869	0.353	0.1611	0.4483	0.4305
11	47238721	rs2013867	-0.7909	0.0002924	-0.9303	8.3e-05	-0.4975	0.001152	-0.6498	0.0002319	intronic	DDB2	47260272	T	C	0.50599	0.7768	0.3473	0.5734	0.3022	0.4581
1	75493802	rs10874314	-0.8195	0.0002928	-0.9472	7.75e-05	-0.3488	0.02261	-0.6716	0.000241	intronic	SLC44A5	75959487	A	G	0.672324	0.9444	0.428	0.8026	0.4841	0.5828
1	3974186	rs11581573	0.8812	0.000293	1.063	3.75e-05	0.4167	0.01915	0.6498	0.001636	intergenic	NC01346;LINC017	4034246	A	G	0.836462	0.996	0.8559	0.8903	0.6918	0.7342
1	3975613	rs10799227	0.8812	0.000293	1.063	3.75e-05	0.4167	0.01915	0.6498	0.001636	intergenic	NC01346;LINC017	4035673	A	G	0.836661	0.996	0.8559	0.8911	0.6918	0.7342
11	102804758	rs519806	0.7053	0.0002931	0.7514	0.000267	0.2913	0.03504	0.4001	0.01098	ncRNA_intronic	WTAPP1	102675489	A	G	0.402157	0.4117	0.4611	0.2542	0.4503	0.501
10	56024998	rs36025969	1.776	0.0002935	1.817	0.0002506	1.091	0.0137	1.418	0.002477	intergenic	PCDH15;ZWINT	57784759	G	A	0.0271565	0.002	0.0159	0.0023	0.0258	0.0961
1	75483686	rs2779489	-0.8185	0.0002942	-0.9456	7.84e-05	-0.351	0.02188	-0.6722	0.000238	intronic	SLC44A5	75949371	C	T	0.670527	0.9435	0.4265	0.798	0.4841	0.5818
17	43851023	rs139009564	0.9417	0.0002953	1.11	4.37e-05	0.4135	0.04361	0.5511	0.01488	intronic	CD300LG	41928391	T	C	0.109625	0	0.1052	0.1392	0.1918	0.1012
17	43854367	rs12453611	0.922	0.0002973	1.083	5.24e-05	0.3712	0.05058	0.4761	0.02895	intronic	CD300LG	41931735	A	G	0.230831	0.001	0.1383	0.5711	0.1988	0.1063
1	75390225	rs1249827	-0.8297	0.0002987	-0.9507	0.0001023	-0.5333	0.0009633	-0.8495	1.32e-05	intronic	SLC44A5	75855910	G	A	0.73742	0.9722	0.6383	0.8684	0.5398	0.592
1	75392019	rs1249824	-0.8297	0.0002987	-0.9507	0.0001023	-0.5218	0.001209	-0.8525	1.27e-05	intronic	SLC44A5	75857704	G	A	0.737021	0.9722	0.6354	0.8684	0.5398	0.592
1	75403000	rs1268530	-0.8297	0.0002987	-0.9507	0.0001023	-0.5218	0.001206	-0.8495	1.32e-05	intronic	SLC44A5	75868685	G	C	0.739217	0.9722	0.6383	0.8684	0.5398	0.6012
1	75408625	rs1249789	-0.8297	0.0002987	-0.9507	0.0001023	-0.5218	0.001206	-0.8495	1.32e-05	intronic	SLC44A5	75874310	A	G	0.738818	0.9722	0.6383	0.8684	0.5398	0.5992
1	75417690	rs1778456	-0.8297	0.0002987	-0.9507	0.0001023	-0.5218	0.001206	-0.8495	1.32e-05	intronic	SLC44A5	75883375	T	A	0.739018	0.9722	0.6383	0.8684	0.5398	0.6002
1	212234220	rs11119887	0.7238	0.0002989	0.7851	0.000182	0.3182	0.02989	0.4343	0.009787	ncRNA_intronic	LINC02608	212407562	C	T	0.286941	0.1984	0.3559	0.2148	0.4066	0.3037
19	1018716	rs11672604	2.179	3e-04	2.13	0.0004347	1.274	0.02194	1.531	0.00795	intronic	TMEM259	1018715	T	C	0.0311502	0.0159	0.0058	0.0136	0.0467	0.0726
12	3936470	rs113915304	1.987	0.0003001	1.962	0.0004047	1.073	0.02774	1.415	0.005363	intergenic	ARP11;CCND2-AS	4045636	A	G	0.0183706	0	0.0101	0.0023	0.0467	0.0358
22	16404475	rs374869632	1.266	0.0003009	1.192	0.001001	0.9188	3.45e-05	1.017	0.0001432	intergenic	DUXAP8;CCT8L2	NA	NA	NA	NA	NA	NA	NA	NA	NA
11	47328736	rs2305280	-0.8382	0.0003012	-0.9812	9.98e-05	-0.5996	0.0001676	-0.7452	5.14e-05	intronic	MADD	47350287	G	A	0.424521	0.6657	0.3026	0.4939	0.2495	0.3487
17	22047452	rs73313398	-1.775	0.0003013	-1.871	0.0005467	-1.146	1.1e-05	-1.645	7.25e-06	intergenic	KCNJ18;UBBP4	NA	NA	NA	NA	NA	NA	NA	NA	NA
6	129536972	rs2812489	0.7626	0.0003014	0.8141	0.0002569	0.4872	0.0009957	0.6701	0.0001074	intergenic	LAMA2;ARHGAP1	129858117	C	T	0.271765	0.4444	0.2421	0.1384	0.3082	0.2577
4	130563566	rs11099064	-0.8274	0.0003029	-0.857	0.0004069	-0.459	0.003374	-0.8465	1.69e-05	intergenic	INC02479;SNHG2	131484721	A	C	0.21865	0.0982	0.3689	0.1218	0.3936	0.1871
13	21603271	rs1200040	0.8426	0.0003029	1.04	4.03e-05	0.5233	0.001525	0.7156	0.0001655	intronic	MICU2	22177410	G	A	0.382987	0.6855	0.3862	0.2526	0.1899	0.4438
2	161026520	rs56363172	0.8958	0.0003037	0.9028	0.000516	0.7794	2.51e-05	0.8742	3.47e-05	intergenic	RBMS1;TANK	161883031	C	CCATTCT	0.127396	0.249	0.0893	0.0121	0.1909	0.1196
11	47316833	rs7109147	-0.7889	0.0003038	-0.9161	9.92e-05	-0.5297	0.0005464	-0.6497	0.0002217	intronic	MADD	47338384	C	T	0.495208	0.7262	0.3429	0.5696	0.3072	0.4581
9	7642053	rs114963968	1.446	0.0003039	1.522	0.0002112	0.6548	0.04044	1.128	0.001339	intergenic	KDM4C;DMAC1	7642053	G	A	0.0644968	0.0942	0.0187	0.0726	0.0169	0.1043
13	21584128	rs9550748	0.8887	0.0003039	1.099	4.14e-05	0.4815	0.006591	0.7347	0.0003291	intronic	MICU2	22158267	G	A	0.315296	0.6944	0.3473	0.0136	0.1859	0.4427
13	21485186	rs9550738	0.8828	0.000304	1.036	8.27e-05	0.4916	0.004685	0.6932	0.0004638	intergenic	ZDHHC20;MICU2	22059325	C	T	0.347843	0.6964	0.3689	0.1097	0.1978	0.4499
2	186216	rs116792475	2.226	0.0003042	2.38	0.0001042	1.506	0.005611	1.776	0.001537	intergenic	FAM110C;SH3YL1	186216	C	G	0.00539137	0	0.0115	0	0.0169	0.002
4	116205302	rs28839198	1.039	0.000305	1.178	8.08e-05	0.6685	0.001645	0.8312	0.0008318	intergenic	NDST4;MIR1973	NA	NA	NA	NA	NA	NA	NA	NA	NA
14	27778090	rs2333106	-0.8473	0.0003053	-0.9662	0.0001589	-0.6178	9.93e-05	-0.4545	0.01032	intergenic	C728755;FOXG1-	28247296	C	G	0.523962	0.5169	0.7334	0.2247	0.7406	0.5644
13	21526751	rs9552444	0.8572	0.000307	1.024	6.35e-05	0.4613	0.006467	0.7273	0.0001798	intronic	MICU2	22100890	T	C	0.347444	0.6944	0.3674	0.1104	0.1988	0.4489
20	29656699	rs1182212945	1.011	0.000309	0.9358	0.001294	0.5989	0.001737	0.8082	0.0003189	intergenic	AM242B;MIR447	NA	NA	NA	NA	NA	NA	NA	NA	NA
1	5673794	rs1015306553	1.434	0.0003092	1.528	0.0001662	0.8841	0.008279	0.9817	0.00754	intergenic	AJAP1;MIR4689	NA	NA	NA	NA	NA	NA	NA	NA	NA
21	30366259	rs1888444	0.7165	0.0003098	0.7339	0.0003706	0.1754	0.2095	0.4176	0.01027	intergenic	TAP23-1;KRTAP1	31738577	G	A	0.319289	0.2698	0.3473	0.1702	0.4503	0.4172
20	11443518	rs17189752	1.181	0.00031	1.026	0.003369	0.61	0.02046	0.5092	0.09638	intergenic	C339593;LINC00	11424166	G	T	0.0491214	0.0893	0.0418	0.0106	0.0517	0.0624
10	88509810	rs2765440	0.7216	0.0003103	0.746	0.0004211	0.1103	0.4424	0.2848	0.07715	intronic	RNLS	90269567	A	G	0.3125	0.4921	0.3631	0.0885	0.3241	0.3824
11	47265115	rs6485746	-0.7896	0.0003123	-0.9284	8.99e-05	-0.4689	0.002113	-0.6357	0.0003174	intronic	NR1H3	47286666	A	G	0.483626	0.7748	0.3372	0.5	0.2972	0.4571
3	77784348	rs75041597	-1.597	0.0003136	-1.766	8.96e-05	-0.5269	0.157	-0.7072	0.08143	intergenic	ROBO2;LINC0207	NA	NA	NA	NA	NA	NA	NA	NA	NA
7	62309232	rs796840421	-1.127	0.0003136	-1.304	4.54e-05	-1.092	6.99e-06	-1.033	0.0001586	intergenic	NONE;ZNF733P	NA	NA	NA	NA	NA	NA	NA	NA	NA
1	143540950	rs372004242	-1.024	0.0003138	-1.092	0.0003132	-0.7969	4.77e-05	-0.8073	0.0004383	intergenic	LSP1P5;RNVU1-1	149035612	G	A	0.000199681	0	0	0.0008	0	0
6	151616868	rs12210699	-1.131	0.0003139	-1.242	0.0003301	-0.9172	1.04e-05	-0.997	6.86e-05	intronic	CCDC170	151938003	C	T	0.148762	0.0198	0.1398	0.1604	0.1928	0.227
3	38532746	rs6802071	0.6917	0.000314	0.6395	0.001295	0.2028	0.1278	0.2683	0.08041	intergenic	EXOG;SCN5A	38574237	C	T	0.582668	0.5575	0.5893	0.8472	0.4254	0.408
21	30325590	rs7279882	0.7267	0.0003145	0.7437	0.0003815	0.1848	0.1919	0.4217	0.01053	intergenic	TAP26-1;KRTAP2	31697908	A	C	0.320887	0.2639	0.3473	0.1778	0.4513	0.4202
22	37330933	rs62235158	1.381	0.0003168	1.371	0.0005533	0.71	0.02488	0.9448	0.008639	intergenic	TH4;LOC1005062	37726974	T	C	0.0389377	0	0.049	0.0567	0.0656	0.0204
1	75456062	rs1936357	-0.8134	0.000318	-0.9283	0.0001145	-0.5218	0.001038	-0.7982	2.79e-05	intronic	SLC44A5	75921747	C	T	0.673922	0.9722	0.6124	0.6611	0.5338	0.5716
14	27464996	rs2333000	-0.7807	0.0003184	-0.8575	0.0002077	-0.2711	0.0533	-0.2652	0.1025	ncRNA_intronic	LOC728755	27934202	A	T	0.458466	0.3909	0.6239	0.2307	0.6352	0.5368
17	26989729	rs80314289	1.289	0.0003184	1.245	0.0008357	0.8844	4.45e-05	1.096	5.14e-05	intergenic	ONE;LOC1053717	NA	NA	NA	NA	NA	NA	NA	NA	NA
1	75389462	rs1249830	-0.8253	0.0003191	-0.9464	0.0001093	-0.5021	0.001906	-0.8239	2.58e-05	intronic	SLC44A5	75855147	C	T	0.73742	0.9722	0.6383	0.8684	0.5398	0.592
1	75413144	rs2972006	-0.8253	0.0003191	-0.9464	0.0001093	-0.5187	0.001296	-0.8455	1.43e-05	intronic	SLC44A5	75878829	T	G	0.739018	0.9722	0.6383	0.8684	0.5398	0.6002
2	230906189	rs2245749	0.7892	0.0003199	0.7828	0.0007129	0.6509	7.72e-05	0.6637	0.0004433	intergenic	GCSIR;GPR55	231770904	C	T	0.298922	0.129	0.232	0.5582	0.2505	0.2209
5	95724540	rs2545638	-0.7479	0.0003202	-0.6752	0.001709	-0.2961	0.04149	-0.3524	0.03838	intronic	SPATA9	95060244	C	T	0.604034	0.9097	0.4741	0.5129	0.4553	0.6575
16	50975287	rs16949179	0.8762	0.000321	0.9437	0.0001979	0.6495	0.0002988	0.8106	5.88e-05	ncRNA_intronic	MIR548AI	51009198	T	C	0.199481	0.2976	0.1427	0.0325	0.2087	0.3548
19	1013817	rs112513985	2.167	0.0003218	2.119	0.0004635	1.263	0.02317	1.521	0.008404	intronic	TMEM259	1013816	C	T	0.0317492	0.0169	0.0072	0.0151	0.0467	0.0716
7	62269014	rs372186635	0.9875	0.0003238	1.021	0.0004174	0.8859	5.27e-06	0.9753	1.94e-05	intergenic	NONE;ZNF733P	NA	NA	NA	NA	NA	NA	NA	NA	NA
22	36830165	rs5756364	0.9064	0.0003238	0.8261	0.001887	0.4484	0.02632	0.6284	0.004262	intergenic	PVALB;NCF4-AS1	37226208	G	A	0.0652955	0.0724	0.0879	0.0129	0.1252	0.0511
20	2631740	rs2422807	0.7631	0.0003239	0.8187	0.0001931	0.291	0.06572	0.3706	0.03884	intronic	TMC2	2612386	C	A	0.784345	0.8571	0.7983	0.711	0.7435	0.8405
1	64563771	rs12118994	0.9807	0.0003242	1.11	0.0001231	0.4467	0.03516	0.7017	0.003164	intronic	CACHD1	65029454	A	G	0.10603	0.1141	0.1095	0.1278	0.0934	0.0787
5	81177716	rs114859703	2.012	0.0003248	2.112	0.0001576	0.9755	0.03817	1.46	0.002737	intronic	RASGRF2	80473535	C	T	0.0135783	0	0.0115	0.0106	0.0398	0.0061
7	51651576	rs10243862	1.415	0.0003266	1.398	0.0006347	0.7566	0.0172	0.9214	0.01389	intergenic	07986794;POM1	51719272	C	T	0.0764776	0	0.0591	0.1868	0.0527	0.0429
14	76839101	rs76970624	1.273	0.0003272	1.442	7.74e-05	0.9149	0.001916	1.002	0.00133	intronic	LRRC74A	77305444	G	A	0.0401358	0	0.0461	0.0053	0.0616	0.1022
2	136614156	rs13428643	1.185	0.0003308	1.373	4.08e-05	0.5517	0.03828	0.9681	0.001012	intergenic	CXCR4;THSD7B	137371726	T	G	0.161941	0.0337	0.0634	0.4062	0.0537	0.1452
1	75467926	rs35678703	-0.8167	0.000331	-0.933	0.0001164	-0.5455	0.0007131	-0.8568	1.03e-05	intronic	SLC44A5	75933611	A	AC	0.685903	0.9752	0.6138	0.6626	0.5398	0.6207
1	200623726	rs10919987	-0.8278	0.0003317	-0.8013	0.0008503	-0.3253	0.02703	-0.5584	0.001641	intergenic	KIF14;DDX59	200592854	T	C	0.720647	0.7173	0.7594	0.8313	0.5755	0.6963
4	6407453	rs56333666	-0.8776	0.000332	-1.115	5.83e-05	-0.6433	8.63e-05	-0.6378	0.0009643	intronic	PPP2R2C	6409180	T	G	0.329473	0.6905	0.1628	0.2292	0.2346	0.3088
4	6407778	rs55678743	-0.8776	0.000332	-1.115	5.83e-05	-0.6433	8.63e-05	-0.6378	0.0009643	intronic	PPP2R2C	6409505	A	G	0.330272	0.6905	0.1614	0.233	0.2346	0.3088
8	27642567	rs12549671	0.7882	0.0003326	0.8865	0.0001182	0.2587	0.1039	0.3878	0.03069	intronic	SCARA3	27500084	A	G	0.128594	0.1339	0.1988	0.0121	0.2694	0.0859
19	24114923	rs148838694	-0.9769	0.0003338	-1.127	9.77e-05	-0.7424	0.0001292	-0.8792	9.73e-05	intronic	ZNF254	NA	NA	NA	NA	NA	NA	NA	NA	NA
17	43858261	rs746742	0.9153	0.0003345	1.075	6.03e-05	0.3516	0.06372	0.4636	0.0334	intronic	CD300LG	41935629	A	G	0.231829	0.001	0.1383	0.5741	0.1988	0.1074
10	79314368	rs1782652	-0.7498	0.0003349	-0.9338	4.64e-05	-0.3556	0.01034	-0.5599	0.0007457	UTR3	ZMIZ1	81074125	T	A	0.534545	0.4484	0.5677	0.708	0.3777	0.5266

19	1010904	rs11665815	2.152	0.0003349	2.125	0.000447	1.264	0.02303	1.52	0.008379	intronic	TMEM259	1010903	G	A	0.0309505	0.0169	0.0058	0.0136	0.0457	0.0716
19	1013011	rs113886504	2.152	0.0003349	2.125	0.000447	1.264	0.02303	1.52	0.008379	intronic	TMEM259	1013010	C	T	0.0317492	0.0169	0.0072	0.0151	0.0467	0.0716
23	125978017	rs459987	0.7847	0.0003356	0.8521	0.0001411	0.3139	0.07161	0.6478	0.0005849	intergenic	(13C;LOC101928	NA	NA	NA	NA	NA	NA	NA	NA	NA
9	40919226	rs202131704	-1.001	0.0003362	-1.035	0.0004772	-0.7514	0.000106	-0.9015	8.06e-05	intergenic	102724580;MIR1	NA	NA	NA	NA	NA	NA	NA	NA	NA
7	55750796	rs35121537	0.9363	0.0003366	0.8466	0.002473	0.5659	0.006861	0.7941	0.0007854	intergenic	KBP9P1;SEPTIN1	55818489	G	GA	0.138578	0.0446	0.1628	0.1846	0.1451	0.1493
22	36825808	rs55889103	0.9023	0.0003371	0.8194	0.001999	0.462	0.02245	0.6358	0.003874	intergenic	PVALB;NCF4-AS1	37221852	T	C	0.0654952	0.0724	0.0879	0.0129	0.1262	0.0511
22	36826192	rs17810645	0.9023	0.0003371	0.8194	0.001999	0.462	0.02245	0.6358	0.003874	intergenic	PVALB;NCF4-AS1	37222236	C	A	0.0654952	0.0724	0.0879	0.0129	0.1262	0.0511
22	36827622	rs5756356	0.9023	0.0003371	0.8194	0.001999	0.4642	0.02188	0.6396	0.003712	intergenic	PVALB;NCF4-AS1	37223665	T	C	0.0654952	0.0724	0.0879	0.0129	0.1262	0.0511
22	36827630	rs5756357	0.9023	0.0003371	0.8194	0.001999	0.462	0.02245	0.6358	0.003874	intergenic	PVALB;NCF4-AS1	37223673	A	G	0.0654952	0.0724	0.0879	0.0129	0.1262	0.0511
22	36827798	rs5756358	0.9023	0.0003371	0.8194	0.001999	0.4454	0.02789	0.6358	0.003874	intergenic	PVALB;NCF4-AS1	37223841	A	C	0.0654952	0.0724	0.0879	0.0129	0.1262	0.0511
22	36828288	rs5756359	0.9023	0.0003371	0.8194	0.001999	0.462	0.02245	0.6358	0.003874	intergenic	PVALB;NCF4-AS1	37224331	T	C	0.0654952	0.0724	0.0879	0.0129	0.1262	0.0511
22	36828314	rs5750318	0.9023	0.0003371	0.8194	0.001999	0.462	0.02245	0.6358	0.003874	intergenic	PVALB;NCF4-AS1	37224357	C	G	0.0654952	0.0724	0.0879	0.0129	0.1262	0.0511
22	36828345	rs78308144	0.9023	0.0003371	0.8194	0.001999	0.4454	0.02789	0.6358	0.003874	intergenic	PVALB;NCF4-AS1	37224388	AC	A	0.0654952	0.0724	0.0879	0.0129	0.1262	0.0511
22	36828505	rs5750319	0.9023	0.0003371	0.8194	0.001999	0.4454	0.02789	0.6358	0.003874	intergenic	PVALB;NCF4-AS1	37224548	A	C	0.0654952	0.0724	0.0879	0.0129	0.1262	0.0511
22	36829446	rs5756363	0.9023	0.0003371	0.8194	0.001999	0.462	0.02245	0.6358	0.003874	intergenic	PVALB;NCF4-AS1	37225489	G	T	0.0654952	0.0724	0.0879	0.0129	0.1262	0.0511
7	58116102	rs1233249921	-1.182	0.0003376	-1.193	0.0006432	-0.9277	1.75e-05	-1.194	1.06e-05	intergenic	ZNF716;NONE	NA	NA	NA	NA	NA	NA	NA	NA	NA
19	1020244	rs11670929	2.161	0.0003382	2.113	0.0004848	1.265	0.02284	1.529	0.008024	intronic	TMEM259	1020243	G	A	0.0313498	0.0159	0.0058	0.0129	0.0467	0.0746
21	30377620	rs11702725	0.7212	0.0003383	0.74	0.0003971	0.1681	0.2316	0.4166	0.01112	intergenic	IIR4327;KRTAP13	31749938	C	T	0.319289	0.2688	0.3473	0.1702	0.4483	0.4202
13	40460536	rs7323870	1.668	0.0003397	1.823	9.66e-05	1.082	0.002024	1.385	0.000619	ncRNA_intronic	LINC00598	41034673	T	C	0.100839	0.0466	0.0677	0.2821	0.0209	0.0174
13	21587458	rs9552454	0.8819	0.00034	1.092	4.66e-05	0.4664	0.008431	0.7212	0.0004211	intronic	MICU2	22161597	C	G	0.315495	0.6954	0.3473	0.0129	0.1849	0.4448
11	71526043	rs1394797419	0.9698	0.0003415	1.125	8.59e-05	0.3386	0.09335	0.4608	0.04455	intergenic	IADSYN1;KRTAP5	NA	NA	NA	NA	NA	NA	NA	NA	NA
21	30336010	rs2832809	0.713	0.0003417	0.727	0.0004509	0.167	0.233	0.4158	0.0109	intergenic	TAP26-1;KRTAP2	31708328	C	T	0.320887	0.2639	0.3473	0.1778	0.4503	0.4213
4	91794132	rs77705061	2.301	0.0003419	2.266	0.0004878	2.026	8.03e-05	1.992	0.0002926	intergenic	CSER1;LNCPRESS	92715283	A	G	0.0427316	0.122	0.1081	0.0008	0.003	0.0123
8	107342983	rs76914295	1.638	0.0003419	1.684	0.0002983	0.5321	0.1946	1.11	0.008363	intronic	ANGPT1	108355211	G	A	0.014377	0	0.0418	0.0008	0.0318	0.0102
18	14265386	rs62082007	0.9892	0.0003422	1.009	0.0004373	0.4963	0.01319	0.6129	0.007054	intergenic	KRD20A5P;CYP4F	14265385	C	T	0.209265	0.4266	0.1816	0.2277	0.0298	0.1646
21	30368739	rs79541361	0.7096	0.0003438	0.7247	0.0004435	0.1745	0.213	0.4148	0.01088	intergenic	TAP23-1;KRTAP1	31741057	A	C	0.31889	0.2688	0.3473	0.1679	0.4493	0.4202
23	42769891	rs5918405	0.6854	0.0003451	0.6506	0.001163	0.2368	0.1079	0.3818	0.02187	intergenic	CASK;PPP1R2C	NA	NA	NA	NA	NA	NA	NA	NA	NA
11	130928717	rs77491995	1.394	0.0003468	1.399	0.0004815	0.567	0.09477	0.8726	0.01455	intergenic	SNX19;NTM	130798612	T	C	0.0529153	0.0585	0.0274	0.0038	0.0507	0.1339
1	75446315	rs2799050	-0.8089	0.0003477	-0.9238	0.000126	-0.5144	0.001239	-0.791	3.35e-05	intronic	SLC44A5	75912000	A	G	0.674521	0.9722	0.6124	0.6611	0.5338	0.5746
3	694543	rs1586218	-0.8423	0.0003486	-0.867	0.0005028	-0.6756	2.46e-05	-0.8038	2.46e-05	ncRNA_intronic	LINC01266	736227	A	G	0.582668	0.6101	0.6499	0.3548	0.7555	0.637
15	63863335	rs7183958	0.8943	0.0003486	0.9583	0.0002139	0.4655	0.01748	0.6571	0.002689	intergenic	HERC1;MIR422A	64155534	G	C	0.170727	0.0337	0.2219	0.289	0.1561	0.1309
1	75446084	rs2587031	-0.8075	0.0003493	-0.9223	0.0001265	-0.5288	0.0008895	-0.7911	3.24e-05	intronic	SLC44A5	75911769	G	A	0.686901	0.9722	0.6196	0.7057	0.5348	0.5716
1	75374582	rs1249805	-0.8204	0.0003495	-0.941	0.0001203	-0.5237	0.001153	-0.8394	1.69e-05	intronic	SLC44A5	75840267	C	T	0.737819	0.9722	0.6412	0.8684	0.5398	0.592
7	51658982	rs1157273	1.192	0.0003509	1.168	0.0007405	0.5082	0.04966	0.7707	0.01021	intergenic	07986794;POM1	51726678	G	A	0.126198	0	0.0994	0.2935	0.0895	0.0869
3	157807086	rs75896011	2.655	0.0003529	2.915	0.0001969	0.8224	0.14	1.251	0.04154	intergenic	SLC66A1L;SHOX2	157524875	C	A	0.0101837	0.0446	0.0086	0	0	0
1	75458428	rs11163435	-0.8074	0.0003533	-0.922	0.0001281	-0.5301	0.0008727	-0.7928	3.16e-05	intronic	SLC44A5	75924113	A	G	0.6877	0.9752	0.6196	0.7057	0.5348	0.5726
4	6407821	rs16838740	-0.8625	0.0003533	-1.068	8.84e-05	-0.6664	4.67e-05	-0.6385	0.0008837	intronic	PPP2R2C	6409548	G	A	0.351637	0.6915	0.1671	0.3101	0.2346	0.3088
7	51648988	rs10234700	1.404	0.0003538	1.398	0.0006347	0.7517	0.01789	0.9176	0.01427	intergenic	07986794;POM1	51716684	C	A	0.0798722	0	0.0591	0.1989	0.0537	0.0429
7	51652168	rs7784099	1.404	0.0003538	1.398	0.0006347	0.7517	0.01789	0.9176	0.01427	intergenic	07986794;POM1	51719864	G	A	0.0744808	0.001	0.0591	0.1785	0.0527	0.0429
7	51653620	rs10245335	1.404	0.0003538	1.398	0.0006347	0.7517	0.01789	0.9176	0.01427	intergenic	07986794;POM1	51721316	A	C	0.076877	0	0.0591	0.1884	0.0527	0.0429
7	51656453	rs58194529	1.404	0.0003538	1.398	0.0006347	0.7517	0.01789	0.9176	0.01427	intergenic	07986794;POM1	51724149	G	A	0.0800719	0	0.0591	0.1997	0.0537	0.0429
13	21608877	rs36087356	0.8767	0.0003542	1.021	9.14e-05	0.5364	0.001761	0.7564	0.000155	intergenic	MICU2;FGF9	22183016	C	CGT	0.370008	0.6885	0.3631	0.2549	0.1561	0.4223
6	32174106	rs408359	1.109	0.0003551	1.134	0.0004907	0.9322	5.09e-05	0.8291	0.001671	intronic	AGPAT1	32141883	G	A	0.15655	0.1518	0.098	0.1876	0.0855	0.2342
20	30584908	rs1206684635	-1.63	0.0003553	-1.671	0.0006905	-1.043	3.33e-05	-1.317	4.89e-05	intergenic	FRG1BP;DEFB115	NA	NA	NA	NA	NA	NA	NA	NA	NA
1	241258555	rs10926442	1.057	0.0003558	1.091	0.0003628	0.8803	5.83e-05	0.9574	0.0001423	intronic	RGS7	241421855	A	G	0.111821	0.1369	0.1427	0.1014	0.1133	0.0767
1	3972313	rs6426410	0.8623	0.0003559	1.041	4.68e-05	0.3985	0.024	0.6274	0.002156	intergenic	NC01346;LINC017	4032373	G	C	0.837061	0.996	0.8573	0.8911	0.6918	0.7352
23	2842935	rs5982883	0.6234	0.0003564	0.6349	0.0005748	0.279	0.02524	0.4199	0.003212	intronic	GYG2	NA	NA	NA	NA	NA	NA	NA	NA	NA
10	38666417	rs61840770	-1.176	0.0003566	-1.318	0.0001043	-1.108	1.31e-05	-1.105	0.0001352	intergenic	NC00999;ACTR3B	38959548	C	G	0.000399361	0	0	0.0015	0	0
1	75463289	rs4949859	-0.8039	0.0003572	-0.9166	0.000132	-0.4783	0.002369	-0.753	5.93e-05	intronic	SLC44A5	75928974	A	G	0.670128	0.9742	0.6124	0.6445	0.5348	0.5716
16	34118387	rs749057303	-1.626	0.0003579	-1.482	0.001169	-1.087	2.3e-05	-1.206	0.0001441	intergenic	NPP7P13;MIR99(	NA	NA	NA	NA	NA	NA	NA	NA	NA
4	82482064	rs12650444	-0.7288	0.0003582	-0.7576	0.0004233	-0.4683	0.0008989	-0.6903	5e-05	intergenic	NOPH1;TMEM15(	83403217	T	C	0.695088	0.5516	0.7378	0.9463	0.5288	0.6442
2	161039142	rs56239078	0.8585	0.0003589	0.8463	0.0009367	0.7321	4.82e-05	0.8177	8.11e-05	intergenic	RBMS1;TANK	161895653	A	G	0.144169	0.249	0.0937	0.0741	0.1909	0.1186
21	30318453	rs2832790	0.7179	0.0003606	0.7275	0.0005144	0.1558	0.2689	0.3988	0.01537	downstream	KRTAP26-1	31690771	C	T	0.321086	0.2639	0.3473	0.1778	0.4513	0.4213
8	15114042	rs17609284	2.204	0.0003608	1.907	0.003682	0.9601	0.04274	0.9793	0.064	intronic	SGCZ	14971551	C	A	0.0347444	0.1349	0	0	0.006	0.0327
2	161077148	rs4664035	0.8423	0.0003612	0.848	0.0007008	0.736	4.7e-05	0.7015	0.000572	intergenic	RBMS1;TANK	161933659	A	G	0.120008	0.2173	0.1037	0.0068	0.1889	0.1135
7	123490498	rs4731108	0.9776	0.0003627	0.9267	0.001246	0.4646	0.02378	0.8284	0.0002891	intronic	IQUB	123130552	T	G	0.178914	0.0575	0.0994	0.2383	0.1431	0.317
4	94256724	rs185800909	1.189	0.0003636	1.201	0.0007032	0.3134	0.1739	0.459	0.08276	intronic	SMARCAD1	95177875	A	C	0.091254	0.2054	0.1844	0.0068	0.0129	0.1022
3	174932013	rs12488154	0.8556	0.0003645	0.7805	0.002404	0.14	0.4157	0.4138	0.0354	intronic	NAALADL2	174649803	G	T	0.263778	0.4583	0.3617	0.0817	0.162	0.3446
6	154470903	rs117458369	1.849	0.0003657	1.996	0.0001258	0.9879	0.03308	1.389	0.003789	intronic	CNKSR3	154792037	T	C	0.00439297	0	0.0029	0.0008	0.0149	0.0041
20	30584911	rs1350854619	-1.627	0.0003672	-1.668	0.0007164	-0.9804	8.31e-05	-1.226	0.0001096	intergenic	FRG1BP;DEFB115	NA	NA	NA	NA	NA	NA	NA	NA	NA
7	58116074	rs1316549251	-0.9775	0.0003674	-1.011	0.0004694	-0.801	3.31e-05	-0.9772	1.75e-05	intergenic	ZNF716;NONE	NA	NA	NA	NA	NA	NA	NA	NA	NA
13	21500434	rs7990069	0.7883	0.0003682	0.894	0.0001457	0.4768	0.002509	0.6805	0.0001726	intronic	MICU2	NA	NA	NA	NA	NA	NA	NA	NA	NA
1	4757448	rs11591221	1.778	0.0003692	1.927	0.000123	0.6991	0.1206	1.207	0.0115	intronic	AJAP1	4817508	C	T	0.0447284	0.0228	0.0086	0.0257	0.0179	0.1462
21	30329744	rs2832800	0.7091	0.0003694	0.7232	0.000484	0.1617	0.2487	0.4103	0.01204	intergenic	TAP26-1;KRTAP2	31702062	A	C	0.321486	0.2639	0.3473	0.18	0.4503	0.4213
7	123495711	rs34986055	0.9787	0.0003703	0.9157	0.001422	0.4492	0.02819	0.816	0.0003386	intronic	IQUB	123135765	A	T	0.178115	0.0575	0.0994	0.2352	0.1431	0.317
7	123505522	rs71574746	0.9787	0.0003703	0.9157	0.001422	0.4536	0.02672	0.8261	0.0002927	intronic	IQUB	123145576	C	T	0.184305	0.0575	0.0994	0.2587	0.1431	0.317
7	57645272	rs78970401	-1.019	0.0003727	-1.145	0.000217	-0.7452	0.0001252	-0.957	3.58e-05	intergenic	ZNF716;NONE	NA	NA	NA	NA	NA	NA	NA	NA	NA
6	151649219	rs3020310	-1.107	0.0003755	-1.234	0.0003242	-0.8616	2.29e-05	-0.9798	7.58e-05	intergenic	CCDC170;ESR1	151970354	A	G	0.171925	0.0298	0.1441	0.2141	0.1988	0.2536
16	13428906	rs142498720	1.597	0.0003768	1.741	0.0001079	0.9238	0.01907	1.376	0.0007035	intergenic	SHISA9;ERCC4	13522763	C	G	0.0185703	0	0.0159	0.003	0.0427	0.0358
2	181654626	rs117657300	3.486	0.00038	3.853	0.0001865	1.701	0.04101	2.225	0.009619	intronic	CERKL	182519353	T	C	0.00738818	0.0367	0	0	0	0
21	30371262	rs35136544	0.7068	0.0003802	0.7265	0.0004371	0.1691	0.2272	0.4093	0.012	downstream	KRTAP13-2	31743580	C	G	0.318291	0.2688	0.3473	0.1679	0.4493	0.4172
21	30371809	rs877346	0.7068	0.0003802	0.7265	0.0004371	0.1691	0.2272	0.4093	0.012	exonic	KRTAP13-2	31744127	A	T	0.318091	0.2688	0.3473	0.1679	0.4483	0.4172
4	94196988	rs76769048	1.165	0.0003807	1.167	0.0007634	0.3197	0.1647	0.4625	0.07875	ncRNA_intronic	LOC101929210	95118139	A	G	0.103235	0.2024	0.1902	0.0514	0.0129	0.1022
10	114406042	rs74565943	1.405	0.0003814	1.346	0.000979	0.475	0.1163	0.6536	0.05704	intergenic	AFAP1L2;ABLIM1	116165801	C	CA	0.0553115	0.1667	0.0331	0.0038	0.0229	0.0593
9	41117483	rs145377242	-1.559	0.0003836	-1.699	0.0001371	-1.39	0.000146	-1.651	2.88e-05	ncRNA_intronic	LOC440896	NA	NA	NA	NA	NA	NA	NA	NA	NA
21	30332164	rs2832802	0.7086	0.0003842	0.727	0.0004509	0.1647	0.2396	0.4123	0.01162	intergenic	TAP26-1;KRTAP2	31704482	C	T	0.321086	0.2639	0.3473	0.1785	0.4503	0.4213
21	30332241	rs2832803	0.7086	0.0003842	0.727	0.0004509	0.1647	0.2396	0.4123	0.01162	intergenic	TAP26-1;KRTAP2	31704559	G	A	0.321486	0.2639	0.3473	0.1793	0.4513	0.4213
21	30335200	rs2832808	0.7086	0.0003842	0.727	0.0004509	0.1647	0.2396	0.4123	0.01162	intergenic	TAP26-1;KRTAP2	31707518	A	T	0.321286	0.2639	0.3473	0.1793	0.4503	0.4213
21	30337373	rs2244485	0.7086	0.0003842	0.727	0.0004509	0.1647	0.2396	0.4123	0.01162	exonic	KRTAP27-1	31709691	G	A	0.321286	0.2639	0.3473	0.1793	0.4503	0.4213
21	30340902	rs2832812	0.7086	0.0003842	0.727	0.0004509	0.1647	0.2396	0.4123	0.01162	intergenic	TAP27-1;KRTAP2	31713220	A	G	0.321286	0.2639	0.3473	0.1793	0.4503	0.4213
21	30345145	rs2409369	0.7086	0.0003842	0.727	0.0004509	0.1647	0.2396	0.4123	0.01162	intergenic	TAP27-1;KRTAP2	31717463	T	G	0.321486	0.2639	0.3473	0.1793	0.4513	0.4213
4	94273361	rs3733400	1.18	0.0003849	1.215	0.0006248	0.3214	0.1688	0.5017	0.0605	intronic	SMARCAD1	95194512	A	G	0.0894569	0.2024	0.1844	0.0023	0.0129	0.1022
22	21023669	rs929671	0.7566	0.0003853	0.8377	0.0001542	0.3483	0.02637	0.4177	0.01817	intronic	P2RX6	21377958	A	C	0.807508	0.9544	0.7968	0.8222	0.6342	0.8221
22	21024136	rs3046030	0.7566	0.0003853	0.8377	0.0001542	0.3483	0.02637	0.4177	0.01817	intronic	P2RX6	NA	NA	NA	NA	NA	NA	NA	NA	NA
10	38951003	rs1350106841	-1.933	0.0003886	-2.104	0.0006681	-1.334	2.63e-06	-1.534	3.15e-05	intergenic	ACTR3BP5;NONE	NA	NA	NA	NA	NA	NA	NA	NA	NA
23	125957424	rs5930527	1.076	0.0003896	1.135	0.0002074	0.4949	0.06406	0.7572	0.006701	intergenic	(13C;LOC101928	NA	NA	NA	NA	NA	NA	NA	NA	NA
4	68806459	rs1304075760	-0.9959	0.00039	-1.125	0.0001672	-0.7946	4.2e-05	-0.9126	6.02e-05	intergenic	IGT2B15;UGT2B1	NA	NA	NA	NA	NA	NA	NA	NA	NA
2	176542952	rs11363495	-0.7137	0.0003911	-0.6871	0.001034	-0.4826	0.0007155	-0.6901	4.03e-05	intergenic	MTX2;MIR1246	177407680	TA	T	0.498203	0.7827	0.585	0.3268	0.4185	0.4571
7	51673072	rs146254117	1.323	0.0003917	1.301	0.0007888	0.5786	0.0519	0.7276	0.0368	intergenic	07986794;POM1	51740768	C	T	0.0810703	0	0.0821	0.1755	0.0676	0.0501
21	30308852	rs2832777	0.7195	0.0003918	0.7169	0.0007179	0.141	0.3115	0.3692	0.02453	intergenic	TAP25-1;KRTAP2	31681170	G	A	0.383986	0.2688	0.3847	0.2988	0.4891	0.5092
14	40612245	rs2415626	0.7181	0.0003924	0.7385	0.0005478	0.5958	8.68e-05	0.5712	0.0007942	intergenic	BXO33;LINC0231	41081450	T	G	0.722444	0.752	0.6124	0.8427	0.5527	0.7822
9	41117441	rs143165272	-1.034	0.0003937	-1.151	0.0001363	-0.9629	1.5e-05	-1.008	4.91e-05	ncRNA_intronic	LOC440896	NA	NA	NA	NA	NA	NA	NA	NA	NA
3	156592989	rs13322059	0.7141	0.000394	0.7536	0.0003461	0.6688	1.51e-05	0.7259	2.31e-05	intergenic	SSR3;TIPARP-AS1	156310778	A	C	0.225839	0.1766	0.2767	0.2141	0.2505	0.2311
15	78662834	rs78642871	-0.982	0.0003945	-0.9353	0.001175	-0.5538	0.00442	-0.8936	0.0001014	intergenic	HRNB4;LOC6469	NA	NA	NA	NA	NA	NA	NA	NA	NA
9	10335857	rs7849722	0.7422	0.0003953	0.7122	0.001113	0.6245	6.58e-05	0.7124	5.72e-05	intronic	PTPRD	10335857	A	C	0.380791	0.3244	0.4294	0.5318	0.2485	0.3364
20	8346053	rs17347458	1.038	0.0003958	1.179	9.46e-05	0.7232	0.001672	0.9787	9.98e-05	intronic	PLCB1	8326700	A	T	0.0732827	0	0.0648	0.0484	0.1859	0.0726
16	46412671	rs796570172	1.112	0.0003963	1.272	7.03e-05	0.6283	0.01127	0.9193	0.0007657	intergenic	NONE;ANKRD26P	NA	NA	NA	NA	NA	NA	NA	NA	NA
6	57858700	rs77101783	-1.079	0.0003971	-1.124	0.0005335	-0.2109	0.2699	-0.4196	0.06235	intergenic	PRIM2;GUSBP4	NA	NA	NA	NA	NA	NA	NA	NA	NA
13	21607168	rs1200043	0.853	0.0003977	1.028	5.85e-05	0.5345	0.001629	0.7371	0.0001749	intergenic	MICU2;FGF9	22181307	G	A	0.377396	0.6905	0.3631	0.2814	0.1541	0.4243

12	97992616	rs77561654	2.932	0.0003978	3.066	0.0001801	1.968	0.0117	2.303	0.004061	intergenic	MIR4495;MIR430	98386394	C	A	0.0139776	0	0.0303	0.0265	0.0139	0
18	7444320	rs12607083	0.8335	0.0003992	0.7887	0.001312	0.257	0.1495	0.3433	0.08483	intergenic	RC30;LOC112577	7444318	C	T	0.142372	0.3383	0.1643	0.0076	0.1252	0.1247
13	63029540	rs372203378	-0.9597	0.0003998	-0.9015	0.001457	-0.8302	1.66e-05	-0.7879	0.000386	intergenic	NC00448;LINC003	NA	NA	NA	NA	NA	NA	NA	NA	NA
12	91580962	rs117191314	2.483	0.0004027	2.654	0.0001963	1.883	0.003099	2.177	0.0008211	intergenic	DCN;LINC01619	91974739	T	C	0.0133786	0.001	0.0144	0	0.0427	0.0133
2	161077339	rs4664404	0.8508	0.0004037	0.8447	0.0009018	0.7409	6.12e-05	0.7226	0.0005713	intergenic	RBMS1;TANK	161933850	A	G	0.117212	0.2153	0.1009	0.0068	0.1809	0.1115
21	30307943	rs7280018	0.7136	0.0004042	0.7275	0.0005144	0.1534	0.2764	0.3951	0.01638	intergenic	TAP25-1;KRTAP2	31680261	T	C	0.321286	0.2639	0.3487	0.1778	0.4513	0.4213
21	30312473	rs7277640	0.7136	0.0004042	0.7275	0.0005144	0.1534	0.2764	0.3951	0.01638	intergenic	TAP25-1;KRTAP2	31684791	T	C	0.320887	0.2639	0.3473	0.1778	0.4503	0.4213
21	30312550	rs13051859	0.7136	0.0004042	0.7275	0.0005144	0.1534	0.2764	0.3951	0.01638	intergenic	TAP25-1;KRTAP2	31684868	A	G	0.320887	0.2639	0.3473	0.1778	0.4503	0.4213
3	34530787	rs6771535	-1.491	0.0004055	-1.341	0.0015	-0.9652	3.04e-05	-1.184	9.96e-05	intergenic	D6IP;LOC101928	34572279	G	A	0.207069	0.1815	0.1023	0.3313	0.0875	0.2628
17	72859575	rs8070819	-0.6807	0.0004059	-0.8092	9.11e-05	-0.2792	0.03495	-0.4822	0.001863	intronic	SLC39A11	70855714	G	A	0.555312	0.6706	0.4597	0.6467	0.4105	0.5297
7	62347850	rs74195110	-1.027	0.0004066	-1.05	0.0006317	-0.8214	3.23e-05	-0.8934	0.0001157	intergenic	NONE;ZNF733P	NA	NA	NA	NA	NA	NA	NA	NA	NA
21	30339039	rs2226643	0.7056	0.0004072	0.7242	0.0004755	0.1658	0.2374	0.4075	0.01265	intergenic	TAP27-1;KRTAP2	31711357	G	A	0.321685	0.2639	0.3473	0.1793	0.4513	0.4223
4	6409692	rs7439007	-0.8538	0.0004084	-1.071	8.86e-05	-0.5979	0.0001857	-0.5929	0.001704	intronic	PPP2R2C	6411419	A	G	0.386581	0.7004	0.1816	0.4085	0.2495	0.32
17	63969831	rs11656511	0.6829	0.00041	0.7239	0.0003233	0.2518	0.07443	0.3858	0.01657	intronic	SCN4A	62047191	C	T	0.244209	0.3036	0.2507	0.0915	0.3688	0.2566
17	21325293	rs1657664	0.9574	0.0004105	1.027	0.000319	0.4771	0.01744	0.5967	0.009046	intergenic	MAP2K3;KCNJ12	NA	NA	NA	NA	NA	NA	NA	NA	NA
6	151618938	rs11155801	-1.044	0.0004109	-1.186	0.0002642	-0.8101	3.41e-05	-0.8537	0.0002388	UTR3	CCDC170	151940073	C	T	0.182109	0.0198	0.1542	0.2421	0.2157	0.2536
18	14257670	rs62081994	0.9893	0.0004114	1.002	0.0005686	0.4498	0.02714	0.6144	0.007299	intergenic	RD20A5P;CYP4F	14257669	G	A	0.198682	0.4167	0.1801	0.2012	0.0298	0.1575
6	166509806	rs395475	0.6412	0.0004126	0.7779	6.96e-05	0.4235	0.001317	0.5085	0.0007832	intronic	RPS6KA2	166923294	G	A	0.473043	0.4276	0.6153	0.2776	0.5706	0.5828
21	30331590	rs2832801	0.7047	0.0004141	0.7232	0.000484	0.1594	0.2556	0.4068	0.01282	intergenic	TAP26-1;KRTAP2	31703908	T	C	0.321286	0.2639	0.3473	0.1793	0.4503	0.4213
21	30342381	rs2832815	0.7047	0.0004141	0.7232	0.000484	0.1552	0.2683	0.4002	0.01437	intergenic	TAP27-1;KRTAP2	31714699	T	C	0.321286	0.2639	0.3473	0.1793	0.4503	0.4213
4	6406590	rs11323274	-0.8619	0.0004143	-1.075	0.0001023	-0.6567	6.43e-05	-0.6365	0.001021	intronic	PPP2R2C	6408317	GA	G	0.149161	0.3175	0.0648	0.1309	0.1014	0.1094
3	77784362	rs77979900	-1.64	0.0004152	-1.784	0.0001468	-0.5707	0.1439	-0.7553	0.0771	intergenic	ROBO2;LINC0207	NA	NA	NA	NA	NA	NA	NA	NA	NA
17	43854007	rs12453522	0.8928	0.000416	1.083	5.24e-05	0.3619	0.05626	0.4627	0.03341	exonic	CD300LG	41931375	A	G	0.230232	0.001	0.1383	0.5688	0.1988	0.1063
9	82612128	rs66662330	-2.062	0.0004165	-2.651	0.000636	-0.3334	0.1425	-1.205	0.0004173	intergenic	SPATA31D1;RASE	85227043	C	T	0.158546	0.4048	0.0288	0.0129	0.0517	0.3037
2	107217625	rs74432250	1.107	0.0004167	1.011	0.001813	0.5214	0.05338	0.6242	0.0311	ncRNA_intronic	MIR548AU	107834081	C	T	0.0371406	0.001	0.0562	0.0023	0.0835	0.0603
1	75366316	rs1249847	-0.7986	0.0004173	-0.903	0.0001711	-0.537	0.0007581	-0.8117	2.32e-05	intronic	SLC44A5	75832001	C	T	0.730032	0.9722	0.6383	0.8449	0.5338	0.592
7	98303291	rs143692552	2.95	0.0004177	2.947	0.0005189	1.684	0.03474	2.141	0.009537	intronic	BAIAP2L1	97932603	C	T	0.00299521	0.001	0.0086	0	0.008	0
10	51590808	rs72797304	0.934	0.0004193	1.024	0.0001665	0.5597	0.009912	0.5913	0.01205	intronic	PRKG1	53350568	C	T	0.0507188	0.0347	0.0706	0.0091	0.1193	0.0389
16	50954839	rs17228232	0.8576	0.0004201	0.899	0.000352	0.6128	0.0005558	0.8055	6.26e-05	ncRNA_intronic	MIR548AI	50988750	G	A	0.213658	0.2688	0.1571	0.1399	0.2087	0.3016
5	11371193	rs6891903	1.188	0.0004212	0.9807	0.006604	0.4369	0.063	0.4211	0.1168	intronic	CTNND2	11371305	A	G	0.138179	0.2312	0.1816	0.1301	0.0209	0.1431
2	161037918	rs12473985	0.8456	0.0004215	0.8463	0.0009367	0.726	5.47e-05	0.8093	9.34e-05	intergenic	RBMS1;TANK	161894429	A	T	0.144968	0.249	0.0937	0.0772	0.1909	0.1186
2	161043027	rs10490561	0.8456	0.0004215	0.8463	0.0009367	0.726	5.47e-05	0.8093	9.34e-05	intergenic	RBMS1;TANK	161899538	T	C	0.144569	0.247	0.0937	0.0772	0.1909	0.1186
2	161043444	rs56325411	0.8456	0.0004215	0.8463	0.0009367	0.726	5.47e-05	0.8093	9.34e-05	intergenic	RBMS1;TANK	161899955	C	T	0.145567	0.246	0.0937	0.0817	0.1909	0.1186
3	719937	rs4686097	-0.8068	0.0004215	-0.8939	0.0002897	-0.6554	3.06e-05	-0.8094	1.75e-05	ncRNA_intronic	LINC01266	761621	A	G	0.427117	0.4018	0.3545	0.646	0.2614	0.3793
11	47254799	rs59663860	-0.7625	0.0004222	-0.9473	6.22e-05	-0.4666	0.002134	-0.6497	0.0002343	intronic	NR1H3	47276350	G	C	0.483427	0.7748	0.3357	0.5	0.2972	0.4571
9	63858422	rs2633117	-0.9588	0.0004223	-1.183	6.29e-05	-0.6044	0.00152	-0.7701	0.0005091	ncRNA_intronic	FRG1JP	NA	NA	NA	NA	NA	NA	NA	NA	NA
8	130366179	rs7005271	1.677	0.000423	1.841	0.000128	0.8434	0.03843	1.33	0.002128	intronic	ASAP1	131378425	G	A	0.0417332	0	0.0259	0.1135	0.0328	0.0082
17	63970378	rs4968680	0.6827	0.0004238	0.7241	0.000333	0.249	0.07646	0.397	0.01324	intronic	SCN4A	62047738	A	G	0.238019	0.3075	0.2464	0.0666	0.3688	0.2577
2	161022179	rs12373609	0.8321	0.0004242	0.8271	0.0009701	0.7743	1.35e-05	0.85	3.37e-05	intergenic	RBMS1;TANK	161878690	C	T	0.191094	0.248	0.1239	0.236	0.1909	0.1196
14	27783869	rs4128099	-0.8256	0.0004242	-0.9363	0.000237	-0.6298	7.7e-05	-0.4581	0.01011	intergenic	728755;FOXG1-	28253075	T	C	0.532748	0.5179	0.7392	0.2519	0.7406	0.5675
2	161071472	rs16845589	0.8458	0.000425	0.8401	0.0009448	0.7499	5.13e-05	0.7145	0.000639	intergenic	RBMS1;TANK	161927983	G	A	0.11861	0.2222	0.1009	0.0068	0.1809	0.1115
2	161072179	rs1541716	0.8458	0.000425	0.8401	0.0009448	0.7317	7.54e-05	0.7145	0.000639	intergenic	RBMS1;TANK	161928690	A	C	0.118211	0.2173	0.1009	0.0076	0.1829	0.1115
2	161077204	rs4664403	0.8458	0.000425	0.8401	0.0009448	0.7401	6.19e-05	0.7145	0.000639	intergenic	RBMS1;TANK	161933715	T	C	0.117212	0.2153	0.1009	0.0076	0.1799	0.1115
2	161090417	rs7420380	0.8458	0.000425	0.8401	0.0009448	0.7401	6.19e-05	0.7145	0.000639	intergenic	RBMS1;TANK	161946928	G	T	0.117013	0.2163	0.1009	0.0068	0.1799	0.1104
6	24669717	rs2223588	0.7828	0.0004251	0.9011	0.0001078	0.4212	0.01167	0.5554	0.003373	intronic	ACOT13	24669945	T	C	0.805511	0.8651	0.8372	0.9304	0.661	0.7014
6	24681193	rs75208363	0.7828	0.0004251	0.9011	0.0001078	0.4335	0.009468	0.5554	0.003373	intronic	ACOT13	24681421	A	G	0.805911	0.8651	0.8386	0.9312	0.661	0.7014
6	24687537	rs926529	0.7828	0.0004251	0.9011	0.0001078	0.4212	0.01167	0.5554	0.003373	UTR5	ACOT13	24687765	A	G	0.802516	0.8651	0.8386	0.9297	0.661	0.6861
6	24688755	rs1885210	0.7828	0.0004251	0.9011	0.0001078	0.4335	0.009468	0.5554	0.003373	intronic	ACOT13	24688983	A	G	0.80611	0.8651	0.8386	0.9319	0.661	0.7014
5	14879703	rs835150	-0.7007	0.0004253	-0.6304	0.002305	-0.5629	7.13e-05	-0.691	3.73e-05	intergenic	ANKH;LINC02149	14879812	C	T	0.659744	0.6716	0.572	0.7995	0.5557	0.6278
7	123498280	rs56408669	0.965	0.0004268	0.9157	0.001422	0.4443	0.02982	0.8099	0.0003725	intronic	IQUB	123138334	C	T	0.184105	0.0575	0.0994	0.2579	0.1431	0.317
6	62929176	rs4368793	-0.7547	0.0004275	-0.6677	0.002584	-0.5637	0.0001186	-0.6928	8.35e-05	intergenic	KHDRBS2;LGSN	63639081	G	C	0.670727	0.7381	0.7233	0.618	0.5875	0.7209
17	21427441	rs145292775	1.345	0.0004296	1.478	0.0001288	0.9166	0.004813	1.23	0.0003474	intergenic	CNJ12;LINC0269	NA	NA	NA	NA	NA	NA	NA	NA	NA
1	231874283	rs61836971	0.6808	0.0004299	0.6565	0.001072	0.2331	0.08347	0.5442	0.0005568	ncRNA_intronic	TSNAX-DISC1	232010029	G	A	0.403554	0.5427	0.4207	0.2905	0.3926	0.4121
3	85166605	rs9832814	-1.294	0.00043	-1.458	0.000453	-0.5822	0.004765	-1.177	5.45e-05	intronic	CADM2	85215755	A	G	0.254593	0.1855	0.0994	0.5832	0.0984	0.1524
18	58719688	rs7230967	-0.8056	0.0004314	-0.8081	0.0007445	-0.4123	0.004058	-0.7365	4.66e-05	intronic	MALT1	56386920	T	G	0.420927	0.3849	0.3617	0.6014	0.2584	0.4233
2	161038582	rs16845489	0.8463	0.0004323	0.8318	0.001141	0.7119	7.3e-05	0.8043	0.0001045	intergenic	RBMS1;TANK	161895093	C	T	0.147764	0.249	0.0965	0.0862	0.1909	0.1186
21	30312115	rs2832784	0.7098	0.0004334	0.7238	0.000549	0.1483	0.2931	0.3898	0.0179	intergenic	TAP25-1;KRTAP2	31684433	T	C	0.321086	0.2639	0.3487	0.1778	0.4503	0.4213
3	150649694	rs12695959	0.7056	0.0004363	0.8299	0.0001183	0.3912	0.005401	0.5357	0.001216	intergenic	SELENOT;ERICH6	150367481	A	C	0.440895	0.4097	0.5735	0.2579	0.5795	0.4836
15	63853510	rs6494440	0.8563	0.0004364	0.957	0.0001664	0.3393	0.07123	0.5294	0.01252	intergenic	HERC1;MIR422A	64145709	C	T	0.783347	0.9673	0.7579	0.5545	0.836	0.8671
2	161097705	rs4664037	0.8442	0.0004369	0.8392	0.0009607	0.738	6.95e-05	0.723	0.000565	intergenic	RBMS1;TANK	161954216	C	T	0.116813	0.2153	0.1023	0.0068	0.1799	0.1094
7	63288568	rs145724731	-1.003	0.000437	-1.016	0.0006745	-0.868	2.52e-05	-0.8404	0.0003663	intergenic	NONE;ZNF733P	NA	NA	NA	NA	NA	NA	NA	NA	NA
2	181181214	rs113419077	1.828	0.0004386	1.814	0.0008256	0.9253	0.02858	1.13	0.01455	ncRNA_intronic	LINC01934	182045941	G	A	0.0479233	0.0615	0.0173	0.1135	0	0.0164
14	27482642	rs11622410	-0.7504	0.0004386	-0.8478	0.0002226	-0.2869	0.0392	-0.2747	0.08839	ncRNA_intronic	LOC728755	27951848	C	G	0.46266	0.3909	0.6268	0.2489	0.6312	0.5358
1	150498680	rs34542152	1.439	0.0004389	1.2	0.007946	1.386	6.97e-05	1.489	9.92e-05	intronic	TARS2	150471156	C	T	0.0405351	0.001	0.0331	0.09	0.0457	0.0143
12	98070538	rs77697520	2.817	0.0004404	3.012	0.0001662	2.2	0.004478	2.639	0.001165	intergenic	IR4303;SLC9A7F	98464316	T	C	0.0257588	0	0.0317	0.0703	0.0139	0
1	885007	rs1390215126	1.34	0.0004424	1.286	0.001186	0.5643	0.08149	0.899	0.009045	intergenic	AM41C;LINC0259	NA	NA	NA	NA	NA	NA	NA	NA	NA
2	161029121	rs16845440	0.8531	0.0004425	0.8496	0.00105	0.7659	2.99e-05	0.8476	5.75e-05	intergenic	RBMS1;TANK	161885632	A	G	0.117212	0.2331	0.0893	0.0121	0.1909	0.0838
10	14547787	rs72768915	1.594	0.0004431	1.763	0.000122	1.031	0.008261	1.153	0.006942	intronic	FAM107B	14589786	A	T	0.0125799	0	0.0274	0.0008	0.0427	0
11	71526065	rs74236613	0.9509	0.0004432	1.098	0.0001233	0.4096	0.04193	0.4972	0.03049	intergenic	ADSYN1;KRTAP5	NA	NA	NA	NA	NA	NA	NA	NA	NA
6	163968996	rs607643	-0.7121	0.0004434	-0.7506	0.0004786	-0.5337	0.0001982	-0.7089	2.87e-05	intergenic	C102724152;ME	164390028	A	G	0.663538	0.5903	0.5778	0.9448	0.5378	0.5491
15	99169163	rs2602014	0.834	0.0004445	0.9847	7.36e-05	0.6096	0.0006382	0.8344	4.56e-05	intronic	TTC23	99709368	C	T	0.208466	0.0982	0.232	0.3109	0.1819	0.1943
13	18724299	rs79631953	-1.132	0.0004448	-1.327	0.0002265	-0.8206	8.81e-05	-1.007	7.92e-05	intergenic	AM230C;LINC004	NA	NA	NA	NA	NA	NA	NA	NA	NA
22	25754222	rs114159181	2.523	0.0004453	2.783	0.0002026	0.8297	0.1305	1.573	0.007152	intronic	MYO18B	26150189	G	A	0.0231629	0.0536	0.0029	0.0401	0	0.0072
6	51517599	rs10498788	1.308	0.0004468	1.313	0.0005151	0.8836	0.00272	1.182	0.0001845	intergenic	TFAP2B;PKHD1	51382397	T	C	0.0623003	0	0.0476	0.1036	0.0875	0.0552
12	37456023	rs1281780347	-1.002	0.0004486	-0.9504	0.001362	-0.7058	0.0002938	-0.9236	6.69e-05	intergenic	NONE;ALG10B	NA	NA	NA	NA	NA	NA	NA	NA	NA
1	231873697	rs72632842	0.6797	0.0004488	0.6577	0.001029	0.2217	0.09934	0.544	0.0005493	ncRNA_intronic	TSNAX-DISC1	232009443	G	A	0.401358	0.5337	0.4207	0.2905	0.3926	0.41
10	125895299	rs5019520	0.9813	0.00045	1.002	0.0006822	0.8478	1.57e-05	0.9443	3.63e-05	intergenic	DHX32;FANK1	NA	NA	NA	NA	NA	NA	NA	NA	NA
6	24683924	rs9461049	0.785	0.0004508	0.925	7.94e-05	0.428	0.01067	0.5527	0.003676	intronic	ACOT13	24684152	G	C	0.805711	0.8651	0.8386	0.9312	0.661	0.7004
9	100746559	rs11506959	1.193	0.0004516	1.161	0.000879	0.5577	0.05386	0.8676	0.005164	intergenic	CAVIN4;PLPPR1	103508841	T	C	0.029353	0	0.0519	0.0159	0.0825	0.0072
4	12759257	rs73105453	1.32	0.0004518	1.424	0.0001951	0.571	0.05382	1.069	0.00125	intergenic	LINC02270;RAB28	12760881	T	C	0.086861	0.0169	0.0548	0.2277	0.0229	0.0573
20	29514900	rs1166196201	-1.036	0.0004519	-1.026	0.0008598	-0.9991	1.02e-06	-1.098	7.43e-06	intergenic	FRG1EP;FAM242	NA	NA	NA	NA	NA	NA	NA	NA	NA
10	79316950	rs5786392	-0.7438	0.0004524	-0.9551	4.7e-05	-0.3374	0.01569	-0.5473	0.001104	downstream	ZMIZ1	81076707	TG	T	0.519569	0.4484	0.562	0.6959	0.3628	0.4857
4	12737481	rs73093526	1.359	0.0004527	1.469	0.0001895	0.6597	0.02576	1.134	0.0008408	intergenic	LINC02270;RAB28	12739105	A	C	0.123003	0.0169	0.0706	0.3555	0.0249	0.0562
17	43869145	rs186720	0.8836	0.0004537	1.037	8.76e-05	0.3606	0.05732	0.4827	0.02614	intergenic	CD300LG;MPP2	41946513	G	A	0.789137	1	0.8602	0.5076	0.8012	0.8896
14	27458957	rs141841271	-0.7547	0.0004549	-0.8256	0.0003096	-0.2665	0.05754	-0.2758	0.08777	ncRNA_intronic	LOC728755	27928163	GTTTTC	G	0.441893	0.3909	0.621	0.1778	0.6302	0.5307
13	66047356	rs149508947	2.001	0.0004557	2.135	0.0001873	1.357	0.009011	1.679	0.001875	intergenic	IR548X2;MIR470	66621488	T	G	0.0123802	0	0.0187	0.0076	0.0258	0.0133
2	181710849	rs77873799	2.804	0.0004561	3.18	0.0002049	1.152	0.0704	1.801	0.00679	intergenic	NEUROD1;ITPRID	182575576	T	C	0.0129792	0.0635	0	0	0	0.001
4	130561135	rs145880576	-0.8051	0.0004575	-0.8286	0.000633	-0.4241	0.007003	-0.8397	2.3e-05	intergenic	INC02479;SNHG2	131482290	C	CT	0.218251	0.0962	0.3631	0.1203	0.3996	0.1871
5	14880103	rs863880	-0.6955	0.0004605	-0.6251	0.002476	-0.5678	5.97e-05	-0.687	4.01e-05	intergenic	ANKH;LINC02149	14880212	A	G	0.654153	0.6716	0.5692	0.7912	0.5557	0.6125
12	98162811	rs12296483	1.576	0.0004605	1.733	0.0001335	0.6731	0.07492	1.023	0.01201	intergenic	IR4303;SLC9A7F	98556589	A	G	0.0726837	0.0327	0.0259	0.1884	0.0129	0.0521
2	164683151	rs6717858	0.7026	0.0004627	0.7823	0.0002209	0.5123	0.0005464	0.6636	8.02e-05	UTR3	COBLL1	165539661	T	C	0.395168	0.0982	0.2579	0.7958	0.4423	0.2086
2	73276147	rs76530306	1.727	0.0004628	1.702	0.0008226	0.994	0.003412	1.734	1.89e-05	intronic	FBXO41	73503275	C	T	0.0700879	0.1905	0.0086	0.003	0.0328	0.1186
12	98059539	rs76873074	2.81	0.0004639	3.012	0.0001662	2.195	0.004563	2.64	0.001181	intergenic	IR4303;SLC9A7F	98453317	A	G	0.0259585	0	0.0317	0.0711	0.0139	0
15	99164881	rs2602028	0.8367	0.0004643	0.9567	0.000117	0.5738	0.001301	0.8316	5.02e-05	intronic	TTC23	99705086	G	A	0.188898	0.0992	0.2262	0.2526	0.1819	0.1759
2	212434988	rs13390934	0.7431	0.0004651	0.7751	0.0005109	0.5613	0.0002999	0.7032	8.25e-05	intronic	ERBB4	213299712	G	A	0.440695	0.12	0.3934	0.7292	0.4404	0.4151
2	161037841	rs12470210	0.8397	0.0004658	0.841	0.001013	0.7199	6.38e-05	0.804	0.000104	intergenic	RBMS1;TANK	161894352	T	C	0.144968	0.249	0.0937	0.0772	0.1909	0.1186
7	142753319	rs930452615	-0.9821	0.000466	-1.219	5.63e-05	-0.4866	0.01365	-0.8368	0.0003308	intronic	TCAF2	NA	NA	NA	NA	NA	NA	NA	NA	NA
6	166319162	rs9355572	-0.9617	0.0004664	-0.9232	0.001212	-0.7295	5.86e-05	-0.7409	0.0006275	downstream	SFT2D1	166732650	A	G	0.784145	0.9742	0.7363	0.5673	0.7555	0.9448
14	27519289	rs58452315	-0.744	0.0004669	-0.8372	0.0002422	-0.2831	0.04082	-0.2709	0.09229	ncRNA_intronic	LOC728755	27988495	TATTTG	T	0.469249	0.3948	0.6196	0.2837	0.6173	0.5378

2	56546838	rs190896416	3.858	0.0004671	3.627	0.00111	2.867	0.00633	3.156	0.003039	intergenic	CCDC85A;VRK2	56773973	G	A	0.00539137	0.0089	0.0029	0.0008	0.008	0.0072
4	12742680	rs7692270	1.312	0.0004676	1.424	0.0001896	0.5693	0.05755	1.06	0.001441	intergenic	LINC02270;RAB2	12744304	G	T	0.0870607	0.0169	0.0562	0.2284	0.0239	0.0552
19	54593217	rs1238188073	1.744	0.0004688	1.802	0.0003563	1.162	0.006528	1.695	0.0001693	upstream	LILRA1	NA	NA	NA	NA	NA	NA	NA	NA	NA
1	231871385	rs61836970	0.6784	0.0004692	0.6565	0.001072	0.2306	0.08688	0.5327	0.0007562	ncRNA_intronic	TSNAX-DISC1	232007131	C	T	0.401957	0.5387	0.4193	0.2859	0.3956	0.4121
1	231871948	rs1015101	0.6784	0.0004692	0.6565	0.001072	0.2309	0.08653	0.5414	0.0005971	ncRNA_intronic	TSNAX-DISC1	232007694	A	G	0.403954	0.5417	0.4207	0.2912	0.3936	0.4131
17	54866521	rs2934899	1.162	0.0004703	1.324	9.69e-05	0.9412	0.0002891	0.8981	0.002962	intergenic	KIF2B;TOM1L1	52943882	T	C	0.939696	0.9216	0.9611	0.947	0.9374	0.9356
9	20305162	rs112034652	1.353	0.0004707	1.328	0.000737	0.4598	0.1675	0.7447	0.03827	intergenic	SLC24A2;MLLT3	20305160	G	A	0.0255591	0.001	0.0677	0.003	0.0497	0.0266
16	25585174	rs72775085	1.156	0.000471	0.9902	0.004887	0.55	0.05103	0.6441	0.03602	intergenic	ZKSCAN2;HS3ST4	25596495	T	C	0.0233626	0.001	0.0173	0.0038	0.0845	0.0143
10	38635801	rs375963143	0.9775	0.0004764	1.088	0.0002174	0.6457	0.001404	0.901	9.79e-05	intergenic	NC00999;ACTR3B	NA	NA	NA	NA	NA	NA	NA	NA	NA
7	53015390	rs11772764	0.7605	0.0004793	0.9041	7.02e-05	0.4591	0.004468	0.7121	9.84e-05	intergenic	07986794;POM1	53083083	G	A	0.220647	0.1587	0.304	0.2065	0.2893	0.1738
10	88509553	rs2765441	0.7018	0.0004793	0.6975	0.000908	0.03878	0.7811	0.2514	0.1178	intronic	RNLS	90269310	G	A	0.388978	0.5327	0.4942	0.261	0.327	0.4029
19	52915317	rs757771823	2.818	0.0004811	2.972	0.0002339	2.214	0.003808	2.529	0.001135	exonic	ZNF888	NA	NA	NA	NA	NA	NA	NA	NA	NA
4	94281224	rs76855076	1.133	0.0004828	1.139	0.000925	0.2717	0.235	0.4428	0.09089	intronic	SMARCAD1	95202375	G	C	0.106629	0.2024	0.1902	0.0635	0.0129	0.1033
17	362159	rs140751740	1.016	0.0004847	1.118	0.0002916	0.7454	0.0005205	1.009	4.43e-05	intergenic	H3AL;LOC105371	211950	C	T	0.000998403	0	0	0	0.003	0.002
2	161071756	rs56070685	0.8343	0.0004883	0.8401	0.0009448	0.7262	8.42e-05	0.7077	0.0007085	intergenic	RBMS1;TANK	161928267	T	G	0.117412	0.2163	0.1009	0.0068	0.1809	0.1115
2	161071757	rs55971385	0.8343	0.0004883	0.8401	0.0009448	0.7262	8.42e-05	0.7077	0.0007085	intergenic	RBMS1;TANK	161928268	T	C	0.117612	0.2163	0.1009	0.0068	0.1819	0.1115
2	161076837	rs60800681	0.8343	0.0004883	0.8401	0.0009448	0.7345	6.94e-05	0.7077	0.0007085	intergenic	RBMS1;TANK	161933348	G	A	0.117812	0.2173	0.1009	0.0076	0.1809	0.1115
2	161087763	rs16845615	0.8343	0.0004883	0.8401	0.0009448	0.7345	6.94e-05	0.7077	0.0007085	intergenic	RBMS1;TANK	161944274	A	G	0.117013	0.2163	0.1009	0.0068	0.1809	0.1094
2	161088264	rs16845618	0.8343	0.0004883	0.8401	0.0009448	0.7345	6.94e-05	0.7077	0.0007085	intergenic	RBMS1;TANK	161944775	A	T	0.117013	0.2163	0.1009	0.0068	0.1809	0.1094
2	161088766	rs12465850	0.8343	0.0004883	0.8401	0.0009448	0.7345	6.94e-05	0.7077	0.0007085	intergenic	RBMS1;TANK	161945277	T	C	0.117013	0.2163	0.1009	0.0068	0.1809	0.1094
1	75433439	rs1249850	-0.7837	0.0004899	-0.8976	0.0001771	-0.4792	0.002323	-0.7723	4.56e-05	intronic	SLC44A5	75899124	G	A	0.691494	0.9722	0.6153	0.7269	0.5338	0.5706
3	153540483	rs6764190	0.7556	0.0004916	0.7132	0.00173	0.5327	0.000452	0.727	4.1e-05	ncRNA_intronic	LINC02006	153258272	G	A	0.549521	0.3978	0.5519	0.9054	0.2932	0.4867
6	5770953	rs1206673	1.073	0.0004928	1.126	0.0003821	0.6024	0.007742	1.01	0.0001108	intronic	FARS2	5771186	G	T	0.820288	0.994	0.8862	0.5756	0.8847	0.8589
6	24689773	rs2143337	0.7748	0.0004959	0.8934	0.0001266	0.42	0.01201	0.5428	0.004219	intronic	ACOT13	24690001	G	T	0.805112	0.8651	0.8372	0.9304	0.661	0.6994
4	66763596	rs1839419	0.9941	0.0004972	1.202	7.55e-05	0.5433	0.009662	0.7692	0.001279	intergenic	1269A;LOC10192	67629314	C	T	0.783347	0.6052	0.8746	0.6861	0.9612	0.8507
2	161091177	rs56324352	0.8338	0.0004978	0.8395	0.0009581	0.7336	7.11e-05	0.7077	0.0007131	intergenic	RBMS1;TANK	161947688	G	A	0.116813	0.2163	0.1009	0.0068	0.1799	0.1094
4	12736566	rs59930899	1.35	0.0004984	1.459	0.0002091	0.6602	0.02589	1.141	0.0007977	intergenic	LINC02270;RAB2	12738190	G	T	0.118411	0.0169	0.0706	0.3381	0.0249	0.0562
17	43843624	rs7209751	0.8949	0.000499	1.065	8.66e-05	0.3104	0.1065	0.4472	0.04267	intergenic	MPP3;CD300LG	41920992	T	C	0.226238	0.001	0.1369	0.5575	0.1958	0.1053
1	75366973	rs11163217	-0.7874	0.0005023	-0.9008	0.0001848	-0.5142	0.001276	-0.8	3.02e-05	intronic	SLC44A5	75832658	T	C	0.261781	0.0278	0.3602	0.1248	0.4662	0.408
1	212467640	rs734976	-0.7098	0.0005046	-0.7882	0.0002834	-0.5639	7.16e-05	-0.6423	0.0001082	intergenic	NENF;LINC01740	212640982	T	G	0.538938	0.6875	0.5014	0.5469	0.5	0.4417
20	30855983	rs1176125915	0.9395	0.0005047	1.037	0.0002464	0.8229	3.06e-05	0.9129	5.23e-05	intergenic	FRG1BP;DEFB11	NA	NA	NA	NA	NA	NA	NA	NA	NA
4	130559672	rs4643822	-0.7983	0.0005052	-0.8224	0.0006894	-0.4214	0.007147	-0.8264	2.89e-05	intergenic	INC02479;SNHG2	131480827	G	A	0.218251	0.0962	0.3631	0.1203	0.3996	0.1871
4	130561718	rs10857145	-0.7983	0.0005052	-0.8224	0.0006894	-0.4214	0.007147	-0.8264	2.89e-05	intergenic	INC02479;SNHG2	131482873	A	G	0.218251	0.0962	0.3631	0.1203	0.3996	0.1871
14	27513740	rs34816607	-0.747	0.0005074	-0.8195	0.0003341	-0.2559	0.06793	-0.2465	0.129	ncRNA_intronic	LOC728755	27982946	C	G	0.451677	0.3948	0.6196	0.2194	0.6173	0.5348
15	85843000	rs62023043	0.8664	0.0005083	0.7601	0.00378	0.2153	0.2509	0.2747	0.208	intergenic	MIR548AP;AGBL	86386231	C	T	0.20028	0.2431	0.1542	0.2179	0.2266	0.138
13	21501408	rs4265674	0.784	0.0005084	0.9384	0.0001159	0.4402	0.007266	0.71	0.0001637	intronic	MICU2	22075547	A	G	0.384784	0.6935	0.3919	0.2413	0.2097	0.4356
4	6402573	rs11722264	-0.8637	0.0005092	-1.098	9.66e-05	-0.6577	8.8e-05	-0.5877	0.002453	intronic	PPP2R2C	6404300	G	A	0.290535	0.6835	0.1484	0.0915	0.2416	0.3057
4	12743405	rs6448941	1.303	0.0005097	1.424	0.0001896	0.565	0.05938	1.056	0.001508	intergenic	LINC02270;RAB2	12745029	C	G	0.0876597	0.0169	0.0562	0.23	0.0239	0.0562
2	161082701	rs16845606	0.8312	0.0005115	0.8377	0.0009721	0.7316	7.45e-05	0.7042	0.0007535	intergenic	RBMS1;TANK	161939212	A	G	0.116813	0.2163	0.1009	0.0068	0.1799	0.1094
2	161082722	rs16845609	0.8312	0.0005115	0.8377	0.0009721	0.7389	6.45e-05	0.7184	0.0006134	intergenic	RBMS1;TANK	161939233	T	C	0.117013	0.2163	0.1009	0.0068	0.1809	0.1094
14	27532610	rs34655877	-0.778	0.000513	-0.8427	0.0004116	-0.2828	0.04837	-0.2593	0.1179	ncRNA_intronic	LOC728755	28001816	A	G	0.545527	0.3958	0.6744	0.5076	0.6412	0.5613
3	34530460	rs6781566	-1.463	0.0005131	-1.327	0.001662	-0.958	3.41e-05	-1.173	0.0001099	intergenic	D6IP;LOC10192	34571952	T	G	0.208866	0.1815	0.1037	0.3366	0.0875	0.2638
13	21606017	rs1200042	0.845	0.0005131	1.017	8.28e-05	0.5135	0.002626	0.7076	0.000341	intergenic	MICU2;FGF9	22180156	C	T	0.36861	0.6885	0.3646	0.2519	0.1521	0.4223
13	21607270	rs11462211	0.845	0.0005131	1.017	8.28e-05	0.5263	0.002057	0.7299	0.0002247	intergenic	MICU2;FGF9	22181409	T	TC	0.371206	0.6935	0.3646	0.2572	0.1521	0.4233
4	130560133	rs72682411	-0.7979	0.0005163	-0.8214	0.0007119	-0.4197	0.007409	-0.8265	2.95e-05	intergenic	INC02479;SNHG2	131481288	T	G	0.218251	0.0962	0.3631	0.1203	0.3996	0.1871
7	53015379	rs11772763	0.7557	0.0005193	0.8988	7.72e-05	0.4504	0.00519	0.7064	0.0001101	intergenic	07986794;POM1	53083072	G	C	0.222644	0.1587	0.304	0.2141	0.2893	0.1738
13	21469642	rs9552430	0.8409	0.0005197	1.035	8.56e-05	0.4897	0.005239	0.7265	0.0002934	intergenic	ZDHHC20;MICU2	22043781	A	C	0.349441	0.7391	0.3646	0.0794	0.2028	0.453
2	113583829	rs2418694	-0.9366	0.0005225	-1.047	0.0002281	-0.4699	0.01941	-0.6665	0.003282	ncRNA_exonic	WASH2P	NA	NA	NA	NA	NA	NA	NA	NA	NA
17	21645610	rs79109536	-0.933	0.0005225	-1.027	0.0002848	-0.8656	1e-05	-0.8902	7e-05	intergenic	INC02693;KCNJ1	NA	NA	NA	NA	NA	NA	NA	NA	NA
4	68806437	rs1466952762	-0.9449	0.0005228	-1.054	0.0002328	-0.8449	1.6e-05	-0.8747	0.0001102	intergenic	GT2B15;UGT2B1	NA	NA	NA	NA	NA	NA	NA	NA	NA
15	63855899	rs4777239	0.8412	0.0005243	0.9571	0.0001638	0.3472	0.06457	0.5509	0.00919	intergenic	HERC1;MIR422A	64148098	T	A	0.788339	0.9673	0.7608	0.5651	0.837	0.8753
17	43884421	rs231517	0.9123	0.0005271	0.9708	0.0005134	0.2666	0.1828	0.3884	0.08781	intronic	MPP2	41961789	C	T	0.814097	1	0.9006	0.5477	0.832	0.9029
8	15110704	rs56795275	2.093	0.0005289	1.808	0.005089	0.8882	0.05155	0.9407	0.06423	intronic	SGCZ	14968213	C	T	0.0359425	0.1379	0.0014	0	0.007	0.0337
5	105279641	rs78248314	0.8425	0.000531	0.9881	8.87e-05	0.1609	0.3472	0.415	0.03215	intergenic	AB9BP1;LINC019	104615342	A	G	0.210863	0.5754	0.2536	0.0091	0.1123	0.1789
2	3837268	rs79056241	3.131	0.0005311	3.403	0.0003256	1.673	0.03201	2.159	0.008571	intronic	DCDC2C	3884858	C	T	0.00858626	0.0407	0	0	0	0.002
22	49835056	rs11090999	2.401	0.0005317	2.487	0.0003563	1.097	0.02309	1.567	0.004788	intergenic	BRD1;ZBED4	50228704	C	T	0.0499201	0.0149	0.0259	0.1536	0.003	0.0112
4	189895563	rs1040277627	-1.026	0.000533	-1.123	0.0004126	-0.6943	0.0004588	-0.9482	7.09e-05	ncRNA_intronic	FRG1-DT	NA	NA	NA	NA	NA	NA	NA	NA	NA
4	94232782	rs113735971	1.122	0.0005343	1.127	0.001036	0.2728	0.2301	0.4223	0.1055	intronic	SMARCAD1	95153933	A	G	0.106829	0.2034	0.1902	0.0635	0.0129	0.1033
18	8843645	rs2889786	0.7027	0.0005351	0.6477	0.002312	0.2953	0.05413	0.4059	0.02048	intergenic	MTCL1;NDUFV2	8843643	T	G	0.187899	0.3095	0.1484	0.0847	0.2634	0.1524
9	41075381	rs541020328	-0.9655	0.0005355	-0.9673	0.0009215	-0.8303	2.37e-05	-0.7846	0.0005497	upstream	PGM5P2	69148610	C	T	0.00359425	0	0.0029	0.0121	0	0
6	24675096	rs9356942	0.77	0.0005368	0.9037	0.0001018	0.4141	0.01318	0.5461	0.003961	intronic	ACOT13	24675324	A	G	0.805911	0.8651	0.8386	0.9312	0.661	0.7014
14	18250396	rs28822101	0.9775	0.0005383	1.112	0.0001784	0.4378	0.03966	0.6029	0.01202	intergenic	NONE;OR11H12	NA	NA	NA	NA	NA	NA	NA	NA	NA
22	49833857	rs6009891	2.392	0.00054	2.492	0.0003486	1.102	0.02244	1.571	0.00471	intergenic	BRD1;ZBED4	50227505	G	A	0.0491214	0.0149	0.0245	0.1513	0.003	0.0112
22	49837101	rs6009326	2.392	0.00054	2.492	0.0003486	1.102	0.02244	1.571	0.00471	intergenic	BRD1;ZBED4	50230749	C	T	0.0497204	0.0149	0.0245	0.1536	0.003	0.0112
15	63852977	rs2414829	0.8441	0.0005428	0.946	0.0002054	0.329	0.08062	0.5172	0.01494	intergenic	HERC1;MIR422A	64145176	T	C	0.783347	0.9673	0.7579	0.5545	0.836	0.8671
1	75392333	rs1249822	-0.7997	0.0005451	-0.9138	0.000197	-0.5099	0.00159	-0.8306	2.1e-05	intronic	SLC44A5	75858018	T	C	0.735423	0.9722	0.6354	0.8623	0.5398	0.592
1	16535369	rs6659375	1.062	0.0005452	1.245	0.0001004	0.6938	0.002698	0.927	0.0003764	ncRNA_exonic	LINC01783	NA	NA	NA	NA	NA	NA	NA	NA	NA
14	27523717	rs35242503	-0.7735	0.0005454	-0.8757	0.0002702	-0.2972	0.03833	-0.2729	0.09867	ncRNA_intronic	LOC728755	27992923	A	T	0.534545	0.4018	0.6657	0.4887	0.6262	0.546
13	21472139	rs1590531	0.833	0.0005468	1.02	9.44e-05	0.4817	0.005681	0.712	0.000343	intergenic	ZDHHC20;MICU2	22046278	C	T	0.347644	0.7391	0.3631	0.0787	0.2028	0.4458
1	5669875	rs77614557	-1.096	0.0005482	-1.157	0.0006002	-0.8773	3.95e-05	-0.883	0.0004211	intergenic	AJAP1;MIR4689	NA	NA	NA	NA	NA	NA	NA	NA	NA
2	161020506	rs4664029	0.792	0.0005483	0.7888	0.001196	0.6822	8e-05	0.7049	0.000369	intergenic	RBMS1;TANK	161877017	T	C	0.138578	0.253	0.1095	0.0144	0.2197	0.1258
15	22774619	rs2347179	-2.312	0.0005496	-2.383	0.0003144	-0.9449	0.07596	-1.645	0.00599	exonic	GOLGA6L7	NA	NA	NA	NA	NA	NA	NA	NA	NA
12	129314264	rs117589149	2.231	0.0005499	2.286	0.0006141	0.8016	0.1362	1.334	0.01788	intronic	TMEM132D	129798809	C	G	0.00958466	0.0228	0.036	0	0	0
3	34529669	rs9837790	-1.454	0.0005504	-1.317	0.001784	-0.9413	4.57e-05	-1.161	0.0001282	intergenic	D6IP;LOC10192	34571161	G	A	0.206869	0.1815	0.1023	0.3306	0.0875	0.2628
18	58740922	rs9946417	-0.784	0.0005543	-0.7594	0.001353	-0.4104	0.004297	-0.7314	5.58e-05	intronic	MALT1	56408154	C	T	0.420727	0.3879	0.3602	0.6029	0.2604	0.4162
3	713181	rs2729088	-0.7904	0.0005549	-0.8713	0.0004127	-0.6428	4.36e-05	-0.7367	8.37e-05	ncRNA_intronic	LINC01266	754865	C	T	0.557907	0.6012	0.6455	0.2973	0.7326	0.6237
6	24669244	rs2223589	0.7686	0.0005559	0.9011	0.0001078	0.4349	0.009407	0.5593	0.00327	intronic	ACOT13	24669472	C	G	0.80611	0.8651	0.8386	0.9319	0.661	0.7014
6	24681427	rs7752101	0.7686	0.0005559	0.9011	0.0001078	0.4137	0.01331	0.545	0.00405	intronic	ACOT13	24681655	T	C	0.805511	0.8651	0.8386	0.9304	0.661	0.7004
6	24681507	rs34254928	0.7686	0.0005559	0.9011	0.0001078	0.4177	0.01255	0.5324	0.005061	intronic	ACOT13	24681735	A	AT	0.817492	0.8661	0.8588	0.9334	0.6918	0.7106
6	24684549	rs60466800	0.7686	0.0005559	0.9011	0.0001078	0.426	0.01083	0.545	0.00405	intronic	ACOT13	24684777	A	AGAAATACAGAG	0.805711	0.8651	0.8386	0.9312	0.661	0.7004
6	24684568	rs1555088	0.7686	0.0005559	0.9011	0.0001078	0.426	0.01083	0.545	0.00405	intronic	ACOT13	24684796	C	T	0.805711	0.8651	0.8386	0.9312	0.661	0.7004
6	24684591	rs1555087	0.7686	0.0005559	0.9011	0.0001078	0.426	0.01083	0.545	0.00405	intronic	ACOT13	24684819	A	T	0.805711	0.8651	0.8386	0.9312	0.661	0.7004
6	24684793	rs1555086	0.7686	0.0005559	0.9011	0.0001078	0.426	0.01083	0.545	0.00405	intronic	ACOT13	24685021	A	G	0.805711	0.8651	0.8386	0.9312	0.661	0.7004
6	24688730	rs77035629	0.7686	0.0005559	0.9011	0.0001078	0.426	0.01083	0.545	0.00405	intronic	ACOT13	24688958	A	G	0.80611	0.8651	0.8386	0.9319	0.661	0.7014
6	24689084	rs4397216	0.7686	0.0005559	0.9011	0.0001078	0.426	0.01083	0.545	0.00405	intronic	ACOT13	24689312	A	G	0.80631	0.8651	0.8386	0.9312	0.663	0.7014
19	31073060	rs8101125	1.411	0.0005566	1.39	0.0009967	0.7074	0.02379	1.303	0.0001615	intergenic	NF536;LINC0179	31563966	T	C	0.129992	0.0337	0.0807	0.2905	0.0437	0.136
17	72857544	rs9894922	-0.66	0.0005608	-0.7867	0.0001294	-0.2578	0.0506	-0.4626	0.002709	intronic	SLC39A11	70853683	T	C	0.45647	0.3294	0.5418	0.3956	0.5905	0.4714
7	101289427	rs140010494	1.312	0.0005612	1.312	0.001494	0.2681	0.4343	0.6484	0.08117	intergenic	FIS1;LNCPRESS1	100932708	G	A	0.0221645	0.001	0.0331	0.0008	0.0557	0.0307
6	70617588	rs2691476	0.6827	0.0005636	0.8834	3.4e-05	0.3541	0.01237	0.4168	0.009375	intergenic	SDHAF4;SMAP1	71327291	A	G	0.674521	0.5337	0.7767	0.8464	0.5915	0.6002
3	126171755	rs77626547	1.607	0.0005638	1.759	0.0002533	1.05	0.008868	1.573	0.0002198	intronic	ALDH1L1	125890598	A	T	0.0251597	0	0.0317	0.0136	0.0805	0.0051
6	64386463	rs6902751	-0.8348	0.0005646	-0.7371	0.002975	-0.6967	2.17e-05	-0.6158	0.0009873	intronic	EYS	65096356	A	T	0.228834	0.1022	0.1945	0.1543	0.326	0.3845
9	82651015	rs11139669	-2.024	0.0005651	-2.625	0.0007663	-0.2839	0.2135	-1.177	0.000598	intergenic	PATA31D1;RASE	85265930	G	A	0.157748	0.3998	0.0288	0.0129	0.0517	0.3047
23	112569784	rs2188164	1.179	0.0005693	1.121	0.001702	0.6225	0.0271	0.8489	0.008779	intergenic	RTL4;LHFPL1	NA	NA	NA	NA	NA	NA	NA	NA	NA
11	7882952	rs111384066	1.797	0.0005699	1.607	0.003555	1.039	0.02398	1.254	0.01153	ncRNA_intronic	LOC283299	7904499	G	A	0.0403355	0.001	0.0274	0.0567	0.0408	0.0675
22	16062888	rs1244136183	-1.688	0.0005713	-1.773	0.00102	-0.8885	0.0003489	-1.368	5.77e-05	intergenic	DUXAP8;CCT8L2	NA	NA	NA	NA	NA	NA	NA	NA	NA
3	150648028	rs6783018	0.7478	0.0005726	0.8578	0.0001895	0.3634	0.01934	0.462	0.01235	intergenic	SELENOT;ERICH6	150365815	G	A	0.381989	0.4931	0.2968	0.475	0.2177	0.3712
18	14406201	rs2669369	1.527	0.0005735	1.658	0.0002172	0.7584	0.04537	0.9974	0.01333	intergenic	YP4F35P;CXADR	NA	NA	NA	NA	NA	NA	NA	NA	NA
4	190007259	rs200526989	-0.9923	0.0005743	-1.008	0.000937	-0.7641	0.0001239	-0.9379	7.58e-05	intergenic	FRG1;FRG2	NA	NA	NA	NA	NA	NA	NA	NA	NA
18	10309472	rs9948140	1.072	0.000575	1.133	0.0003947	0.9329	2.46e-05	1.066	1.93e-05	intergenic	VAPA;LINC01254	10309469	C	A	0.153554	0.252	0.2291	0.0885	0.0994	0.1421

19	1021328	rs2107367	1.682	0.0005752	1.609	0.001357	0.7368	0.1033	0.9935	0.03718	upstream	RNU6-7;RNU6-8;F	1021327	A	G	0.0333466	0.0159	0.0115	0.0129	0.0527	0.0746
3	36067502	rs6795407	0.6847	0.0005758	0.7839	0.0002114	0.2386	0.1004	0.3747	0.02356	intergenic	ARPP21;STAC	36108994	A	G	0.249002	0.1062	0.3329	0.2012	0.3907	0.2556
15	78662826	rs77687110	-0.9533	0.0005761	-0.9042	0.001705	-0.5413	0.005421	-0.884	0.000128	intergenic	HRNB4;LOC6469	NA	NA	NA	NA	NA	NA	NA	NA	NA
15	78662827	rs74851770	-0.9533	0.0005761	-0.9042	0.001705	-0.5413	0.005421	-0.884	0.000128	intergenic	HRNB4;LOC6469	NA	NA	NA	NA	NA	NA	NA	NA	NA
2	161058715	rs2358013	0.8145	0.0005793	0.8106	0.001206	0.7172	8.92e-05	0.6852	0.0009301	intergenic	RBMS1;TANK	161915226	T	C	0.119609	0.2222	0.1009	0.0068	0.1809	0.1166
2	161058718	rs2358014	0.8145	0.0005793	0.8106	0.001206	0.7172	8.92e-05	0.6852	0.0009301	intergenic	RBMS1;TANK	161915229	C	G	0.119609	0.2222	0.1009	0.0068	0.1809	0.1166
14	77242461	rs61731611	0.7917	0.0005802	0.9102	0.0001354	0.3141	0.07035	0.5859	0.002593	exonic	TMEM63C	77708804	C	T	0.146366	0.1042	0.1902	0.0106	0.2555	0.2301
6	71936688	rs9341357	0.7335	0.0005808	0.7668	0.0006415	0.5957	0.0001889	0.7532	4.63e-05	intronic	RIMS1	72646391	G	A	0.786941	0.9891	0.7608	0.798	0.6292	0.7444
17	22053814	rs9909212	-0.8262	0.0005811	-1.049	7.35e-05	-0.4192	0.006882	-0.4438	0.01366	intergenic	KCNJ18;UBBP4	NA	NA	NA	NA	NA	NA	NA	NA	NA
14	106038199	rs1394149836	-0.9861	0.0005816	-1.036	0.000716	-0.7832	6.27e-05	-0.5843	0.008814	intergenic	ADAM6;LINC0022	NA	NA	NA	NA	NA	NA	NA	NA	NA
21	30329547	rs13049297	0.678	0.0005848	0.6876	0.0008036	0.1545	0.2684	0.3954	0.01495	intergenic	TAP26-1;KRTAP2	31701865	C	A	0.324081	0.2639	0.3473	0.1884	0.4513	0.4223
6	166181207	rs6924516	-1.269	0.0005853	-1.281	0.001298	-0.8999	7.57e-05	-1.032	0.0002332	intergenic	CDAT;LOC101929	166594695	A	T	0.147564	0.0575	0.1009	0.2269	0.1471	0.1667
3	77784354	rs78157285	-1.573	0.0005857	-1.742	0.0001766	-0.5722	0.1423	-0.7487	0.07867	intergenic	ROBO2;LINC0207	NA	NA	NA	NA	NA	NA	NA	NA	NA
14	27467377	rs1954667	-0.7369	0.0005861	-0.8235	0.0003254	-0.2905	0.04043	-0.3001	0.06556	ncRNA_intronic	LOC728755	27936583	T	C	0.454673	0.6071	0.3458	0.4508	0.3688	0.4683
18	7479205	rs117235270	1.592	0.0005872	1.732	0.0001851	0.7098	0.08836	0.9971	0.02108	intergenic	C112577592;PTP	7479203	G	C	0.0135783	0	0.0317	0.0038	0.0358	0.0051
15	63853901	rs72752925	0.9185	0.0005905	0.9586	0.0005201	0.4833	0.02348	0.6503	0.005615	intergenic	HERC1;MIR422A	64146100	C	G	0.0744808	0.002	0.2032	0.0257	0.1511	0.045
6	135227298	rs72976223	2.164	0.0005912	2.27	0.0003119	1.242	0.02955	1.675	0.004332	intergenic	MYB;MIR548A2	135548436	A	G	0.0141773	0.0397	0.0058	0.0008	0.0239	0.002
6	17487275	rs9477456	1.683	0.0005918	1.746	0.0006947	0.6369	0.08072	0.8917	0.04089	intronic	CAP2	17487506	A	G	0.205671	0.0198	0.072	0.7201	0.007	0.001
20	28784621	rs1416442670	-0.9351	0.0005935	-0.9655	0.0007776	-0.8776	1.16e-05	-0.9625	2.63e-05	intergenic	FRG1CP;FRG1DP	NA	NA	NA	NA	NA	NA	NA	NA	NA
9	43186620	rs1250387557	0.9618	0.0005953	0.9043	0.002174	0.8794	2.72e-05	0.6628	0.005921	intergenic	C102724904;NO	NA	NA	NA	NA	NA	NA	NA	NA	NA
18	13192073	rs77854977	1.83	0.0005954	2.049	0.0002505	0.5954	0.1136	1.34	0.003171	intergenic	CEP192;LDLRAD4	13192072	G	A	0.0527157	0.0823	0.0187	0.1263	0	0.001
18	13192383	rs78635717	1.83	0.0005954	2.049	0.0002505	0.5954	0.1136	1.34	0.003171	intergenic	CEP192;LDLRAD4	13192382	G	A	0.0527157	0.0823	0.0187	0.1263	0	0.001
18	13192527	rs112560408	1.806	0.0005956	2.008	0.0002369	0.7158	0.08297	1.279	0.004972	intergenic	CEP192;LDLRAD4	13192526	G	A	0.0329473	0.0823	0.0115	0.0552	0	0.001
1	241240547	rs118131772	2.016	0.0005958	1.792	0.003685	1.001	0.04162	1.247	0.01636	intronic	RGS7	241403847	T	C	0.0227636	0.0556	0.072	0	0.003	0.0051
6	146456439	rs9390388	0.7692	0.0005985	0.8166	0.000485	0.3177	0.03685	0.6664	0.0002499	intergenic	GRM1;RAB32	146777575	G	A	0.294529	0.5	0.2478	0.0658	0.2714	0.4489
13	21477065	rs3070123	0.7054	0.000599	0.8193	0.0002207	0.51	0.000438	0.6927	4.44e-05	intergenic	ZDHHC20;MICU2	22051204	A	AC	0.366214	0.3264	0.4481	0.0938	0.5974	0.4796
13	65964497	rs73199816	1.578	0.0005991	1.724	0.0001926	1.045	0.01143	1.308	0.002395	intergenic	NC01052;MIR548	66538629	G	A	0.0141773	0.001	0.0159	0.0076	0.0348	0.0143
17	43890215	rs231508	0.9047	0.0006005	0.9521	0.0006524	0.2451	0.225	0.3935	0.0866	intronic	MPP2	41967583	G	A	0.82528	1	0.8991	0.5862	0.837	0.9039
6	63048583	rs4710399	-0.7231	0.0006012	-0.643	0.0033	-0.5825	6.2e-05	-0.5493	0.001161	intergenic	KHDRBS2;LGSN	63758488	T	C	0.65615	0.7728	0.732	0.525	0.6074	0.7096
5	156978231	rs34507698	-0.9238	0.0006055	-1.266	9.88e-05	-0.4915	0.003272	-0.6736	0.001098	intergenic	TIMD4;HAVCR1	156405242	C	CT	0.244808	0.2153	0.1888	0.3427	0.1988	0.2301
6	135222699	rs72976217	2.159	0.0006069	2.266	0.0003204	1.239	0.03002	1.67	0.004434	intergenic	MYB;MIR548A2	135543837	T	C	0.0145767	0.0407	0.0058	0.0008	0.0249	0.002
6	24653148	rs1129644	0.762	0.0006076	0.8896	0.0001256	0.4274	0.01075	0.5527	0.003599	exonic	TDP2	24653376	G	A	0.805112	0.8661	0.8372	0.9319	0.662	0.6953
4	59295743	rs111980450	2.474	0.0006109	2.597	0.0003123	1.175	0.04021	1.657	0.01006	intergenic	C02429;MIR548A	60161461	G	T	0.0447284	0.0605	0.0418	0.0802	0.005	0.0235
6	24687771	rs926528	0.7631	0.0006112	0.8964	0.0001177	0.3913	0.01922	0.5395	0.004449	intronic	ACOT13	24687999	T	C	0.805711	0.8651	0.8386	0.9312	0.661	0.7004
13	66187720	rs3938553	1.851	0.0006112	2.02	0.0002125	1.35	0.008787	1.61	0.002222	intergenic	MIR548X2;MIR47(	66761852	G	A	0.0121805	0	0.0173	0.0091	0.0229	0.0143
3	75663767	rs8180065	0.9186	0.0006127	1.04	0.0002824	0.7537	7.89e-05	0.8245	0.0002003	upstream	FRG2C	NA	NA	NA	NA	NA	NA	NA	NA	NA
23	912289	rs139065290	0.8154	0.0006131	0.7672	0.002034	0.7795	7.78e-06	0.7282	0.0002874	intergenic	SHOX;CRLF2	NA	NA	NA	NA	NA	NA	NA	NA	NA
10	106403355	rs35713152	-0.7112	0.0006141	-0.6903	0.001668	-0.6576	4.03e-05	-0.6736	0.000187	intergenic	INC02624;SORCS	108163113	TAG	T	0.275759	0.129	0.3458	0.1354	0.5427	0.2924
14	42789952	rs78791031	1.413	0.0006184	1.423	0.0008552	0.6175	0.07826	0.9275	0.01478	intergenic	LRFN5;FSCB	43259155	G	A	0.0379393	0	0.0288	0.0961	0.0417	0.001
22	22349342	rs1328565625	-1.12	0.0006187	-1.037	0.00213	-0.9438	1.54e-05	-0.9131	0.0003517	intergenic	MS1P20;ZNF280	NA	NA	NA	NA	NA	NA	NA	NA	NA
23	86406873	rs6617227	0.6728	0.0006191	0.7056	0.0008152	0.485	0.0008605	0.6924	2.63e-05	intronic	DACH2	NA	NA	NA	NA	NA	NA	NA	NA	NA
3	126168107	rs115875394	1.589	0.0006225	1.743	0.0002769	1.033	0.009939	1.553	0.000257	intronic	ALDH1L1	125886950	C	G	0.0257588	0	0.0317	0.0136	0.0835	0.0051
10	79314006	rs2997468	-0.7198	0.0006225	-0.9227	6.99e-05	-0.3272	0.01897	-0.554	0.0009905	UTR3	ZMIZ1	81073763	G	C	0.523562	0.4494	0.5648	0.7042	0.3638	0.4908
4	130560477	rs72682412	-0.7873	0.0006236	-0.8077	0.000886	-0.4238	0.007044	-0.828	3.04e-05	intergenic	INC02479;SNHG2	131481632	G	T	0.218251	0.0962	0.3631	0.1203	0.3996	0.1871
17	12470105	rs7211433	0.6629	0.0006237	0.8312	6.73e-05	0.291	0.03324	0.4523	0.004352	intergenic	MAP2K4;LINC0067	12373422	C	T	0.630391	0.6548	0.5375	0.6657	0.5557	0.7004
3	126174609	rs79058125	1.601	0.0006239	1.733	0.0003166	1.047	0.00914	1.568	0.0002345	intronic	ALDH1L1	125893452	A	T	0.0209665	0	0.0259	0.0008	0.0805	0.0051
4	23845317	rs57256690	1.363	0.0006261	1.416	0.0004685	1.09	0.0005023	1.394	5.41e-05	intronic	PPARGC1A	23846940	C	T	0.0455272	0.001	0.036	0.0847	0.0487	0.0419
6	24666977	rs17243164	0.7613	0.0006313	0.8938	0.0001235	0.41	0.01416	0.5398	0.004428	upstream	ACOT13;TDP2	24667205	T	TCC	0.805312	0.8661	0.8386	0.9312	0.661	0.6973
21	30359479	rs10446082	0.6753	0.0006328	0.6863	0.0008435	0.1657	0.2364	0.4095	0.01205	intergenic	TAP23-1;KRTAP1	31731797	A	T	0.329273	0.2649	0.3501	0.2103	0.4503	0.4172
20	29805435	rs1427919180	1.143	0.0006331	1.267	0.0002355	1.012	0.0001547	1.239	1.81e-05	intergenic	AM242B;MIR447	NA	NA	NA	NA	NA	NA	NA	NA	NA
2	13191182	rs79119888	1.707	0.0006351	1.703	0.001092	0.9788	0.0102	1.091	0.01166	intergenic	100506474;LINC0	13331307	G	A	0.0950479	0.123	0.072	0.2163	0.004	0.0123
4	12759061	rs73105449	1.262	0.0006378	1.369	0.000269	0.5149	0.07816	1.004	0.002049	intergenic	LINC02270;RAB2	12760685	A	G	0.086861	0.0169	0.0548	0.2277	0.0229	0.0573
4	12759267	rs112846957	1.262	0.0006378	1.369	0.000269	0.5149	0.07816	1.004	0.002049	intergenic	LINC02270;RAB2	12760891	A	G	0.0872604	0.0169	0.0548	0.2284	0.0239	0.0573
20	29498385	rs1328678817	1.042	0.0006381	1.125	0.0005568	0.8428	2.91e-05	1.088	1.09e-05	intergenic	FRG1EP;FAM242	NA	NA	NA	NA	NA	NA	NA	NA	NA
6	24690799	rs3822901	0.7608	0.000643	0.8936	0.0001259	0.4266	0.01042	0.5523	0.00345	intronic	ACOT13	24691027	A	G	0.805711	0.8651	0.8386	0.9312	0.661	0.7004
5	134986146	rs35542434	0.8167	0.0006437	0.7284	0.003845	0.2153	0.2249	0.3198	0.1117	intronic	CATSPER3	134321836	C	CT	0.166534	0.0397	0.2075	0.1452	0.2654	0.1953
11	47246999	rs7129661	-0.7339	0.000644	-0.9268	8.87e-05	-0.4678	0.002054	-0.6152	0.0004311	intronic	ACP2	47268550	T	G	0.50599	0.7768	0.3473	0.5734	0.3022	0.4581
1	231875197	rs999710	0.6676	0.0006455	0.6415	0.001525	0.2235	0.09962	0.5333	0.0007891	ncRNA_intronic	TSNAX-DISC1	232010943	G	A	0.403554	0.5427	0.4207	0.2905	0.3926	0.4121
10	38660786	rs61839340	0.9212	0.0006514	1.071	0.0001732	0.3513	0.07825	0.5102	0.02457	intergenic	NC00999;ACTR3B	NA	NA	NA	NA	NA	NA	NA	NA	NA
19	1021512	rs1985546	1.662	0.0006527	1.604	0.001418	0.7272	0.1079	0.984	0.03903	upstream	RNU6-7;RNU6-8;F	1021511	C	T	0.0339457	0.0159	0.0115	0.0129	0.0517	0.0787
14	77232936	rs7143692	-0.8904	0.0006543	-0.9981	0.0004637	-0.4998	0.002681	-0.8196	9.59e-05	intronic	TMEM63C	77699279	A	G	0.264377	0.256	0.1398	0.4024	0.2187	0.2219
16	9153901	rs4471682	0.7068	0.0006543	0.8015	0.0002386	0.5085	0.0007191	0.695	5.52e-05	intergenic	16orf72;LINC021	9247758	A	T	0.312899	0.3393	0.2637	0.2209	0.3708	0.3855
4	12732647	rs16887512	1.294	0.0006556	1.408	0.0002685	0.5997	0.03977	1.061	0.001441	intergenic	LINC02270;RAB2	12734271	C	T	0.123403	0.0169	0.0706	0.3555	0.0258	0.0573
4	12736412	rs60877882	1.294	0.0006556	1.408	0.0002685	0.5997	0.03977	1.061	0.001441	intergenic	LINC02270;RAB2	12738036	AAAC	A	0.122604	0.0169	0.0706	0.354	0.0249	0.0562
4	12737246	rs59772912	1.294	0.0006556	1.408	0.0002685	0.6008	0.03948	1.062	0.001428	intergenic	LINC02270;RAB2	12738870	A	G	0.122604	0.0169	0.0706	0.354	0.0249	0.0562
4	12739638	rs73093535	1.294	0.0006556	1.408	0.0002685	0.6008	0.03948	1.062	0.001428	intergenic	LINC02270;RAB2	12741262	A	G	0.123203	0.0169	0.0706	0.357	0.0249	0.0552
4	12740783	rs6836849	1.294	0.0006556	1.408	0.0002685	0.6008	0.03948	1.062	0.001428	intergenic	LINC02270;RAB2	12742407	G	T	0.123003	0.0169	0.0692	0.357	0.0249	0.0552
6	24661283	rs2056999	0.7566	0.0006581	0.8703	0.0001805	0.3992	0.01696	0.5392	0.004458	intronic	TDP2	24661511	T	C	0.804912	0.8661	0.8386	0.9319	0.661	0.6943
10	38969137	rs1842290	0.9996	0.0006596	1.291	9.18e-05	0.4431	0.02207	0.7358	0.001427	intergenic	ACTR3BP5;NONE	15165892	A	T	0.000199681	0.001	0	0	0	0
3	34528295	rs6785939	-1.429	0.0006622	-1.291	0.002137	-0.9322	5.7e-05	-1.149	0.0001477	intergenic	CD6IP;LOC101928	34569787	A	G	0.204872	0.1835	0.1037	0.3185	0.0885	0.2648
18	14257545	rs62081993	0.9383	0.0006655	0.9508	0.0009053	0.3808	0.05862	0.4937	0.03018	intergenic	KRD20A5P;CYP4F	14257544	G	T	0.198083	0.4157	0.1801	0.2012	0.0298	0.1554
14	27448876	rs1379143	-0.7313	0.0006671	-0.8312	0.0003263	-0.2599	0.06398	-0.2676	0.09933	ncRNA_intronic	LOC728755	27918082	G	C	0.458466	0.3919	0.6254	0.23	0.6362	0.5348
4	81212110	rs989445	0.8635	0.0006688	0.8405	0.0014	0.5577	0.002232	0.8696	2.92e-05	intronic	PRKG2	82133264	C	A	0.216254	0.3353	0.2622	0.2617	0.0408	0.18
6	32587971	rs4713547	1.142	0.0006758	1.244	0.0003642	0.9107	0.0002562	1.162	3.78e-05	intronic	HLA-DRB1	NA	NA	NA	NA	NA	NA	NA	NA	NA
6	158688649	rs11753677	1.25	0.0006773	1.172	0.002103	0.5479	0.07925	0.785	0.01952	intronic	SYTL3	159109681	A	C	0.019369	0	0.0288	0.003	0.0676	0.0051
10	42269838	rs200056850	-0.9333	0.000678	-1.002	0.0005705	-0.9408	1.52e-06	-1.034	5.72e-06	intergenic	NONE;LOC44166(	NA	NA	NA	NA	NA	NA	NA	NA	NA
15	63852614	rs7174738	0.8404	0.000678	0.942	0.0002615	0.3489	0.06752	0.518	0.01591	intergenic	HERC1;MIR422A	64144813	A	T	0.783347	0.9673	0.7579	0.5545	0.836	0.8671
15	99187057	rs2602019	0.8041	0.0006812	0.9374	0.0001451	0.626	0.0003936	0.8026	7.52e-05	intronic	TTC23	99727262	A	T	0.210863	0.0913	0.2334	0.3215	0.1809	0.1994
21	30352879	rs2832819	0.6713	0.0006823	0.6825	0.0009032	0.1512	0.2801	0.3875	0.01732	intergenic	TAP23-1;KRTAP1	31725197	C	G	0.321885	0.2679	0.3473	0.1793	0.4503	0.4202
3	174932140	rs12488801	0.814	0.0006837	0.7305	0.004462	0.1467	0.3951	0.3906	0.04756	intronic	NAALADL2	174649930	G	A	0.261581	0.4583	0.3602	0.0749	0.162	0.3436
2	91860167	rs74443020	-0.9335	0.0006879	-0.9519	0.001069	-0.806	3.86e-05	-0.8204	0.0002983	intergenic	GGT8P;ACTR3BP2	NA	NA	NA	NA	NA	NA	NA	NA	NA
2	161038529	rs55654087	0.8189	0.0006907	0.8069	0.00175	0.7291	5.87e-05	0.8112	0.000102	intergenic	RBMS1;TANK	161895040	A	AAAACAAAC	0.144369	0.249	0.0937	0.0756	0.1909	0.1176
7	28514975	rs11392816	1.166	0.0006984	1.337	0.0001165	0.8911	0.002009	0.9543	0.002139	intronic	CREB5	28554593	A	AT	0.954273	0.9435	0.9452	0.9939	0.9026	0.9714
12	98032006	rs75260981	2.756	0.0006994	2.933	0.0002911	1.929	0.01323	2.257	0.004835	intergenic	MIR4303;SLC9A7F	98425784	T	C	0.0127796	0	0.0274	0.0234	0.0139	0
5	8774231	rs13178537	-0.8761	0.0007013	-0.9024	0.0009764	-0.6699	8.61e-05	-0.636	0.001522	intergenic	R4458HG;LINC02	8774343	C	T	0.291334	0.2897	0.245	0.2307	0.3221	0.3763
8	103134798	rs2454012	0.6383	0.0007024	0.7692	0.0001699	0.3279	0.01619	0.4647	0.00325	ncRNA_exonic	LINC01181	104147026	C	G	0.474641	0.4375	0.4798	0.5106	0.4453	0.4908
15	85841413	rs62023033	0.8373	0.0007032	0.7483	0.004219	0.2419	0.196	0.3026	0.1652	intergenic	MIR548AP;AGBL	86384644	G	A	0.206669	0.2391	0.1571	0.2383	0.2306	0.1411
15	85841582	rs60822461	0.8373	0.0007032	0.7483	0.004219	0.2419	0.196	0.3026	0.1652	intergenic	MIR548AP;AGBL	86384813	G	T	0.206869	0.2401	0.1585	0.239	0.2306	0.1391
15	85841640	rs59528508	0.8373	0.0007032	0.7483	0.004219	0.2419	0.196	0.3026	0.1652	intergenic	MIR548AP;AGBL	86384871	A	T	0.195687	0.2212	0.1542	0.2201	0.2266	0.1339
18	58689624	rs9944971	-0.7754	0.0007039	-0.7575	0.001591	-0.4137	0.004553	-0.7044	0.0001184	intronic	MALT1	56356856	A	G	0.332668	0.3869	0.3256	0.2814	0.2594	0.4264
4	130559015	rs4068888	-0.7744	0.0007044	-0.8074	0.0008908	-0.4341	0.005929	-0.8209	3.39e-05	intergenic	INC02479;SNHG2	131480170	C	CTG	0.218051	0.0952	0.3631	0.1203	0.3996	0.1871
7	62301998	rs79241325	1.009	0.0007049	1.12	0.0004903	0.8579	2.01e-05	0.918	0.0001249	intergenic	NONE;ZNF733P	NA	NA	NA	NA	NA	NA	NA	NA	NA
10	38643144	rs1297852527	-0.9698	0.0007073	-0.9904	0.001092	-0.7933	6.01e-05	-0.8559	0.0002276	intergenic	NC00999;ACTR3B	NA	NA	NA	NA	NA	NA	NA	NA	NA
21	30353514	rs2832820	0.6708	0.0007081	0.6863	0.0008435	0.1544	0.2698	0.3896	0.01671	intergenic	TAP23-1;KRTAP1	31725832	A	G	0.320887	0.2629	0.3473	0.1793	0.4503	0.4202
21	30356435	rs2245296	0.6708	0.0007081	0.6863	0.0008435	0.1544	0.2698	0.3896	0.01671	intergenic	TAP23-1;KRTAP1	31728753	C	A	0.320887	0.2649	0.3473	0.18	0.4503	0.4172
21	30356513	rs2898165	0.6708	0.0007081	0.6863	0.0008435	0.1544	0.2698	0.3896	0.01671	intergenic	TAP23-1;KRTAP1	31728831	T	C	0.320887	0.2649	0.3473	0.18	0.4503	0.4172
21	30356730	rs2832825	0.6708	0.0007081	0.6863	0.0008435	0.1544	0.2698	0.3896	0.01671	intergenic	TAP23-1;KRTAP1	31729048	T	C	0.320887	0.2649	0.3473	0.18	0.4503	0.4172
12	98079433	rs2216021	0.6634	0.0007093	0.7824	0.0001669	0.1019	0.4565	0.2695	0.08674	intergenic	MIR4303;SLC9A7F	98473211	C	T	0.590256	0.7649	0.4914	0.4955	0.504	0.6973
4	12736179	rs73093517	1.286	0.0007095	1.408	0.0002685	0.6021	0.03903	1.067	0.001372	intergenic	LINC02270;RAB2	12737803	G	A	0.122604	0.0169	0.0706	0.354	0.0249	0.0562

4	12738190	rs56790423	1.286	0.0007095	1.408	0.0002685	0.5959	0.04104	1.057	0.001507	intergenic	LINC02270;RAB2	12739814	G	A	0.123203	0.0169	0.0706	0.3563	0.0249	0.0562
1	5034643	rs1459763	0.7455	0.0007103	0.7842	0.0006042	0.6554	5.37e-05	0.6016	0.001308	intergenic	AJAP1;MIR4689	5094703	A	G	0.285343	0.1597	0.2493	0.3911	0.3002	0.2822
17	43868401	rs231532	0.8467	0.0007122	0.9961	0.0001434	0.3369	0.07476	0.4545	0.03547	intergenic	CD300LG;MPP2	41945769	C	T	0.786741	1	0.8573	0.5068	0.7932	0.8885
8	110600130	rs138593398	2.252	0.000714	1.995	0.004274	1.104	0.07182	1.43	0.02542	intergenic	KCNV1;LINC0160	111612359	A	G	0.0105831	0	0.0144	0.0008	0.0199	0.0225
1	63179544	rs11208148	-0.7999	0.0007145	-0.9046	0.000446	-0.4354	0.004198	-0.7204	0.0001427	ncRNA_intronic	LINC00466	63645215	A	C	0.33726	0.4563	0.3458	0.2905	0.2455	0.3661
20	30843499	rs1392018980	2.872	0.0007145	2.897	0.0007472	2.222	0.004993	2.853	0.0004961	intergenic	FRG1BP;DEFB11	NA	NA	NA	NA	NA	NA	NA	NA	NA
4	66773838	rs4860779	0.9408	0.0007156	1.122	0.0001357	0.4452	0.03081	0.6849	0.003443	intergenic	1269A;LOC10192	67639556	C	T	0.78754	0.6071	0.8761	0.7057	0.9602	0.8436
4	66773854	rs11358571	0.9408	0.0007156	1.122	0.0001357	0.4275	0.03843	0.6849	0.003443	intergenic	1269A;LOC10192	67639572	AG	A	0.78754	0.6071	0.8761	0.7057	0.9602	0.8436
7	123442856	rs7805957	0.9494	0.000717	0.9155	0.00177	0.4494	0.03133	0.7922	0.000593	intergenic	SLC13A1;IQUB	123082910	C	T	0.148163	0.0556	0.1009	0.1846	0.1342	0.2423
1	5034375	rs6693990	0.7451	0.0007179	0.7843	0.0006103	0.6601	4.69e-05	0.6025	0.001283	intergenic	AJAP1;MIR4689	5094435	G	A	0.285743	0.1597	0.2507	0.3911	0.3012	0.2822
11	120497586	rs12365193	-0.6872	0.0007192	-0.6176	0.003517	-0.4546	0.0009798	-0.6432	8.81e-05	intergenic	ARHGEF12;GRIK	120368295	A	T	0.317093	0.5119	0.3934	0.0673	0.4811	0.2311
4	12736966	rs57746976	1.284	0.0007193	1.407	0.0002709	0.595	0.04134	1.055	0.001537	intergenic	LINC02270;RAB2	12738590	A	G	0.122604	0.0169	0.0706	0.354	0.0249	0.0562
20	29662420	rs1429184444	-0.9777	0.0007194	-0.9767	0.001329	-0.9172	5.73e-06	-0.9995	3.09e-05	intergenic	AM242B;MIR447	NA	NA	NA	NA	NA	NA	NA	NA	NA
11	28591790	rs7118207	0.7777	0.000724	0.863	0.0002989	0.3208	0.06845	0.5128	0.009049	intergenic	IIR8068;LINC025	28613337	A	G	0.127396	0.1776	0.1182	0.0628	0.165	0.1309
22	26539641	rs113615864	2.888	0.0007253	2.98	0.000428	1.949	0.01408	2.248	0.005099	intronic	TPST2	26935607	C	T	0.00978435	0.002	0.0101	0.0008	0.0249	0.0143
13	40144585	rs145151791	1.327	0.0007277	1.388	0.0004807	1.275	3.09e-05	1.309	0.0001481	intergenic	COG6;LINC00332	40718722	GGAAGGGGAGA	T	0.914537	0.997	0.9524	0.7731	0.9215	0.9867
14	27507409	rs12887064	-0.7259	0.0007309	-0.7975	0.0004839	-0.2505	0.07298	-0.2486	0.1242	ncRNA_intronic	LOC728755	27976615	C	G	0.45627	0.3958	0.6254	0.2231	0.6302	0.5348
12	97933203	rs78732422	2.097	0.0007352	2.29	0.0002429	0.5813	0.2288	1.319	0.01612	intergenic	FAH1B2P2;MIR4	98326981	C	A	0.0445288	0	0.0432	0.1324	0.0169	0.001
16	36100859	rs1238425945	0.9371	0.0007359	1.086	0.0002126	0.9591	2.77e-06	0.9019	0.0001151	intergenic	LINC02167;NONE	NA	NA	NA	NA	NA	NA	NA	NA	NA
17	43853201	rs4793040	0.8604	0.0007372	1.042	0.0001098	0.3708	0.05237	0.452	0.03987	intronic	CD300LG	41930569	A	G	0.226637	0.001	0.1369	0.559	0.1958	0.1053
20	30594869	rs1239482392	-1.213	0.0007375	-1.333	0.0006281	-0.8892	5.57e-05	-0.9702	0.0002768	intergenic	FRG1BP;DEFB11	NA	NA	NA	NA	NA	NA	NA	NA	NA
6	152225295	rs201573874	0.8724	0.0007385	0.9405	0.0005609	0.7513	7.67e-05	0.8252	0.0001475	intronic	SYNE1	NA	NA	NA	NA	NA	NA	NA	NA	NA
4	94191117	rs17021378	1.08	0.0007399	1.101	0.001224	0.2751	0.2238	0.4363	0.09239	ncRNA_intronic	LOC101929210	95112268	A	G	0.107029	0.2024	0.1902	0.0635	0.0129	0.1053
4	12732186	rs77048240	1.261	0.000744	1.361	0.0003397	0.6774	0.02574	1.075	0.00126	intergenic	LINC02270;RAB2	12733810	A	T	0.0770767	0.0169	0.0533	0.1906	0.0249	0.0562
6	146464848	rs9403788	0.7571	0.0007447	0.8082	0.0005753	0.2974	0.05161	0.6619	0.0002854	intergenic	GRM1;RAB32	146785984	A	G	0.299521	0.5198	0.2493	0.0651	0.2724	0.453
12	32916914	rs80116589	2.076	0.0007451	2.334	0.0002526	0.945	0.05454	1.431	0.006884	intergenic	PKP2;SYT10	33069848	G	A	0.0241613	0.0734	0.0014	0	0.001	0.046
1	64638087	rs71645597	2.438	0.0007482	2.615	0.00029	1.564	0.01786	1.99	0.003325	intronic	CACHD1	65103770	C	T	0.0117812	0	0.0101	0	0.0368	0.0153
14	27495393	rs12894202	-0.7262	0.0007498	-0.7989	0.0004898	-0.2508	0.07429	-0.2456	0.1295	ncRNA_intronic	LOC728755	27964599	T	G	0.444489	0.3958	0.6239	0.177	0.6312	0.5368
15	85841662	rs55861516	0.8327	0.0007518	0.7433	0.004482	0.2264	0.227	0.2781	0.2041	intergenic	MIR548AP;AGBL	86384893	C	T	0.206869	0.2401	0.1585	0.239	0.2306	0.1391
8	102721404	rs9642793	-1.142	0.0007529	-1.132	0.001585	-1.034	7.43e-06	-0.8634	0.0008041	intergenic	C101927245;GAS	103733632	G	A	0.150359	0.0962	0.2882	0.1384	0.1083	0.1677
6	146425526	rs5880676	0.695	0.0007547	0.7707	0.0004175	0.544	0.0003738	0.6971	5.88e-05	intronic	GRM1	146746662	CA	C	0.198283	0.0833	0.2939	0.0507	0.4115	0.229
4	154148485	rs1010428108	-0.9771	0.0007565	-1.098	0.0004605	-0.9193	6.26e-06	-0.8684	0.0002135	intergenic	SFRP2;DCHS2	NA	NA	NA	NA	NA	NA	NA	NA	NA
19	31077772	rs10500249	1.426	0.000758	1.445	0.0008138	0.8579	0.009032	1.277	0.0003534	intergenic	NF536;LINC0179	31568678	T	C	0.11901	0.0278	0.0793	0.2496	0.0437	0.1421
22	36841148	rs5750323	0.7802	0.000758	0.6089	0.01257	0.2859	0.123	0.4121	0.04331	intergenic	PVALB;NCF4-AS1	37237191	T	G	0.114217	0.0774	0.1268	0.1293	0.1421	0.0941
7	62301570	rs79435125	-0.9435	0.00076	-0.9864	0.0009509	-0.7362	0.0001446	-0.9036	8.59e-05	intergenic	NONE;ZNF733P	NA	NA	NA	NA	NA	NA	NA	NA	NA
20	29830819	rs1333236166	1.168	0.0007609	1.109	0.002054	0.9494	2.19e-05	1.015	0.0001494	intergenic	AM242B;MIR447	NA	NA	NA	NA	NA	NA	NA	NA	NA
21	30361611	rs11702321	0.667	0.0007613	0.6825	0.0009032	0.149	0.2874	0.3841	0.01838	intergenic	TAP23-1;KRTAP1	31733929	A	C	0.320288	0.2649	0.3473	0.1778	0.4503	0.4172
21	30361612	rs11702344	0.667	0.0007613	0.6825	0.0009032	0.149	0.2874	0.3841	0.01838	intergenic	TAP23-1;KRTAP1	31733930	C	A	0.320288	0.2649	0.3473	0.1778	0.4503	0.4172
1	64552895	rs77805650	0.9151	0.000763	0.9944	0.0005989	0.3593	0.09419	0.5569	0.02116	intronic	CACHD1	65018578	G	T	0.103435	0.0982	0.111	0.1346	0.0895	0.0757
16	9156159	rs62034331	0.7012	0.0007676	0.8362	0.0001558	0.522	0.0005646	0.727	3.08e-05	intergenic	16orf72;LINC021	9250016	T	G	0.247404	0.2966	0.2291	0.0817	0.3241	0.3548
3	34539179	rs6794147	-1.407	0.0007698	-1.261	0.002655	-0.9131	7.76e-05	-1.131	0.0001808	intergenic	D6IP;LOC10192	34580671	T	C	0.196286	0.1825	0.098	0.2927	0.0875	0.2618
8	110545877	rs117249190	2.208	0.0007714	1.965	0.004572	1.114	0.06852	1.424	0.02561	intergenic	KCNV1;LINC0160	111558106	A	G	0.0109824	0	0.0173	0	0.0199	0.0235
8	110555593	rs116868706	2.208	0.0007714	1.965	0.004572	1.114	0.06852	1.424	0.02561	intergenic	KCNV1;LINC0160	111567822	G	A	0.0107827	0	0.0159	0	0.0199	0.0235
8	110589628	rs117738230	2.208	0.0007714	1.965	0.004572	1.114	0.06852	1.424	0.02561	intergenic	KCNV1;LINC0160	111601857	T	C	0.0107827	0	0.0159	0	0.0199	0.0235
14	27529637	rs12882804	-0.7142	0.0007718	-0.7957	0.0004514	-0.2582	0.06411	-0.2774	0.08501	ncRNA_intronic	LOC728755	27998843	G	T	0.493011	0.6032	0.353	0.6059	0.3668	0.456
3	156624763	rs2089735	0.8113	0.0007829	0.8438	0.0007243	0.6535	0.0003461	0.8028	9.54e-05	intergenic	SSR3;TIPARP-AS1	156342552	G	C	0.189097	0.1667	0.2046	0.2322	0.167	0.1656
3	156625194	rs6762762	0.8113	0.0007829	0.8438	0.0007243	0.6535	0.0003461	0.8028	9.54e-05	intergenic	SSR3;TIPARP-AS1	156342983	G	A	0.18131	0.1657	0.2017	0.2042	0.167	0.1667
1	61349741	rs74913171	2.044	0.0007855	2.136	0.0003885	0.9698	0.07741	1.474	0.007891	intronic	NFIA	61815413	A	G	0.00698882	0	0.0072	0.0008	0.0179	0.0112
17	43863144	rs151019121	0.89	0.0007874	1.082	0.0001015	0.3535	0.09251	0.504	0.02931	UTR3	CD300LG	41940512	G	A	0.0754792	0	0.0965	0.0113	0.1918	0.1053
12	103307487	rs78602813	1.717	0.0007922	1.723	0.0009247	0.7128	0.0435	0.9069	0.02286	intronic	C12orf42	103701265	G	A	0.0543131	0.1964	0.036	0.0136	0.001	0.0307
20	30175338	rs1390437361	-0.9134	0.0007926	-0.8845	0.001826	-0.8607	1.36e-05	-0.8322	0.000264	intergenic	IR4477A;LINC015	NA	NA	NA	NA	NA	NA	NA	NA	NA
20	29635193	rs1329985963	-1.244	0.0007965	-1.335	0.0009751	-0.9299	5.73e-05	-1.024	0.0002699	intergenic	AM242B;MIR447	NA	NA	NA	NA	NA	NA	NA	NA	NA
6	5782952	rs17145857	1.062	0.0007981	1.087	0.0008504	0.5778	0.01704	1.001	0.0002671	intergenic	FARS2;NRN1	5783185	G	T	0.152955	0.0089	0.1066	0.3419	0.0934	0.1401
3	41378162	rs186609898	1.214	0.0007991	1.19	0.001434	0.8559	0.004089	1.188	0.0001762	intronic	ULK4	41419653	G	A	0.0183706	0.001	0.0389	0	0.0577	0.0061
19	15507910	rs10417873	0.6717	8e-04	0.8477	8.94e-05	0.3375	0.0188	0.5476	0.001109	upstream	CYP4F22	15618721	G	A	0.302117	0.2083	0.3732	0.2587	0.4781	0.226
8	103078140	rs77342006	1.745	0.0008008	1.913	0.0002333	0.8106	0.08882	1.117	0.02243	intergenic	P6V1C1;LINC011	104090368	T	G	0.0125799	0	0.0303	0.0015	0.0348	0.0051
9	40982147	rs878973429	1.574	0.0008033	1.731	0.0002263	0.9971	0.01471	1.414	0.001027	intergenic	MIR1299;FRG1H	NA	NA	NA	NA	NA	NA	NA	NA	NA
13	101588335	rs1436269	-0.7493	0.000805	-0.7156	0.002294	-0.4105	0.005406	-0.769	4.24e-05	intronic	ITGBL1	102240685	A	G	0.313498	0.4425	0.3256	0.1619	0.3042	0.3865
1	75398844	rs1249781	-0.7482	0.0008071	-0.8504	0.0003279	-0.5113	0.001301	-0.7774	4.16e-05	intronic	SLC44A5	75864529	G	C	0.672923	0.9722	0.6311	0.6293	0.5368	0.593
6	24682663	rs1923188	0.7365	0.0008113	0.8669	0.0001602	0.4069	0.01422	0.5219	0.005476	intronic	ACOT13	24682891	T	C	0.805711	0.8651	0.8386	0.9312	0.661	0.7004
14	27464022	rs7146037	-0.7075	0.0008175	-0.7821	0.0005137	-0.2655	0.05836	-0.2706	0.09307	ncRNA_intronic	LOC728755	27933228	A	G	0.504992	0.6091	0.3631	0.6301	0.3698	0.4683
18	67741605	rs72950666	1.277	0.0008176	1.258	0.001656	0.1419	0.6401	0.6702	0.04576	ncRNA_intronic	LOC643542	65408842	G	A	0.0792732	0.0675	0.0476	0.1452	0.0388	0.0665
9	74553576	rs59567401	-1.106	0.0008212	-1.766	5.97e-05	-0.3844	0.04234	-0.7441	0.00254	intronic	RORB	77168492	T	TA	0.186901	0.0595	0.2406	0.3245	0.1322	0.1503
17	43870930	rs231529	0.9135	0.0008219	0.9641	0.0007713	0.2652	0.2094	0.4357	0.07077	intergenic	CD300LG;MPP2	41948298	A	G	0.873802	1	0.9121	0.7648	0.834	0.9049
14	27508063	rs12897025	-0.7201	0.0008222	-0.7922	0.0005399	-0.2403	0.08622	-0.2382	0.1418	ncRNA_intronic	LOC728755	27977269	T	A	0.45627	0.3968	0.6254	0.2224	0.6302	0.5348
19	47967130	rs2386988	0.6622	0.0008232	0.8181	0.0001328	0.4568	0.001146	0.7048	2.65e-05	downstream	BSPH1	48470387	T	C	0.365415	0.3393	0.4957	0.0802	0.5527	0.4928
14	27752022	rs12435247	-0.6763	0.0008238	-0.813	0.0002031	-0.5694	9.63e-05	-0.4893	0.003176	intergenic	C728755;FOXG1-	28221228	T	C	0.561302	0.497	0.3718	0.879	0.4076	0.4908
4	40270289	rs150273962	2.469	0.0008242	2.575	0.0004277	1.375	0.0489	1.821	0.009516	intergenic	RHOH;LINC0226	40271909	T	A	0.00519169	0	0.013	0	0.0149	0.002
3	713686	rs12715600	-0.7798	0.0008271	-0.7959	0.001219	-0.6076	0.0001463	-0.7487	9.14e-05	ncRNA_intronic	LINC01266	755370	T	C	0.402955	0.3988	0.3444	0.5628	0.2644	0.3753
2	91863206	rs76773210	0.9947	0.0008294	1.042	0.0007954	1.06	2.43e-06	1.06	2.87e-05	intergenic	GGT8P;ACTR3BP	NA	NA	NA	NA	NA	NA	NA	NA	NA
4	179597924	rs2716805	0.7329	0.0008318	0.82	0.0003565	0.601	8.32e-05	0.6192	0.000423	intergenic	NC01098;LINC002	180519077	T	C	0.57508	0.3125	0.6571	0.5272	0.7575	0.6646
10	78373920	rs75596673	1.235	0.0008329	1.215	0.00156	0.4819	0.1314	0.738	0.03312	intergenic	NC00595;ZMIZ1-	80133677	G	A	0.0219649	0.001	0.072	0	0.0427	0.0164
7	53017580	rs34663451	0.7241	0.0008473	0.8612	0.0001402	0.4374	0.006539	0.6857	0.0001663	intergenic	07986794;POM1	53085273	C	CA	0.221645	0.1567	0.304	0.2141	0.2863	0.1738
3	34539526	rs6781445	-1.396	0.0008488	-1.262	0.002615	-0.9123	7.95e-05	-1.127	0.000191	intergenic	D6IP;LOC10192	34581018	C	A	0.200879	0.1806	0.1009	0.3101	0.0875	0.2618
14	27489624	rs10147744	-0.7171	0.0008499	-0.7885	0.0005595	-0.2382	0.08892	-0.2368	0.1439	ncRNA_intronic	LOC728755	27958830	T	C	0.457069	0.3958	0.6268	0.2247	0.6302	0.5358
9	74548966	rs4745339	-1.108	0.0008509	-1.755	7.13e-05	-0.3833	0.04716	-0.7384	0.002923	intronic	RORB	77163882	A	G	0.175919	0.0595	0.2392	0.2837	0.1322	0.1503
12	103324277	rs17033698	1.701	0.000851	1.708	0.0009852	0.7032	0.04609	0.8968	0.02417	intronic	C12orf42	103718055	C	A	0.0545128	0.1964	0.036	0.0136	0.001	0.0317
5	2335297	rs1873950	0.6657	0.0008517	0.6507	0.001754	0.4109	0.003808	0.6214	0.0001526	intergenic	C100506858;LSIN	2335411	C	G	0.329872	0.1915	0.3646	0.2436	0.4374	0.454
1	229216261	rs115896969	2.012	0.0008544	2.048	0.0008682	1.292	0.02743	1.625	0.005563	intergenic	4454;LOC10537	229352008	G	A	0.0125799	0	0.0288	0.0008	0.0209	0.0215
1	75364753	rs1249849	-0.7619	0.000855	-0.8649	0.0003659	-0.501	0.001877	-0.7832	5.28e-05	intronic	SLC44A5	75830438	C	T	0.733427	0.9722	0.6369	0.8646	0.5338	0.5838
16	8119364	rs79323309	1.814	0.0008562	1.924	0.0006587	0.8316	0.06057	1.396	0.003455	intergenic	RBFOX1;TMEM11	8169366	G	A	0.0267572	0.0972	0.0461	0	0	0.0041
6	32156766	rs432953	1.018	0.0008563	1.037	0.001218	0.8899	6.47e-05	0.7785	0.00261	ncRNA_intronic	PPT2-EGFL8	32124543	T	A	0.159345	0.1528	0.0965	0.1899	0.0934	0.2372
14	91380549	rs10665247	0.8763	0.0008577	0.7876	0.003719	0.2318	0.2146	0.4884	0.02336	intronic	CCDC88C	91846893	C	CATCTATCT	0.778355	0.5595	0.804	0.8487	0.9433	0.7209
6	24657254	rs3212233	0.7421	0.0008579	0.8703	0.0001805	0.404	0.01581	0.5288	0.005336	intronic	TDP2	24657482	C	G	0.805112	0.8661	0.8386	0.9319	0.661	0.6953
14	27501208	rs10137632	-0.7357	0.0008583	-0.834	0.0004309	-0.2524	0.0709	-0.2272	0.1638	ncRNA_intronic	LOC728755	27970414	C	T	0.490415	0.3958	0.6527	0.3298	0.6392	0.5368
17	43868196	rs231534	0.8283	0.0008605	1.002	0.000131	0.3353	0.07571	0.45	0.03696	intergenic	CD300LG;MPP2	41945564	C	T	0.786941	1	0.8588	0.5068	0.7932	0.8885
17	43868812	rs231531	0.8283	0.0008605	1.002	0.000131	0.3353	0.07571	0.45	0.03696	intergenic	CD300LG;MPP2	41946180	G	T	0.786741	1	0.8573	0.5068	0.7932	0.8885
21	30362531	rs8130778	0.6853	0.0008613	0.7213	0.0007213	0.1366	0.3403	0.3863	0.02091	intergenic	TAP23-1;KRTAP1	31734849	G	A	0.317891	0.2649	0.3473	0.1687	0.4503	0.4172
7	53019411	rs6957020	0.7233	0.0008616	0.8604	0.0001426	0.4533	0.005164	0.6865	0.0001677	intergenic	07986794;POM1	53087104	A	T	0.221246	0.1567	0.3026	0.2141	0.2853	0.1738
7	123460409	rs36004403	0.9525	0.0008635	0.9148	0.002208	0.4819	0.02406	0.8175	0.0005279	intronic	IQUB	123100463	CT	C	0.124401	0.0536	0.0965	0.1362	0.1431	0.182
5	176696853	rs74428436	2.529	0.0008658	2.468	0.001535	0.6808	0.3237	1.45	0.04005	intergenic	SPAN17;LINC015	176123854	T	C	0.014377	0.0327	0.0014	0.028	0	0.001
22	49782382	rs72490251	4.896	0.000866	4.89	0.0006476	2.857	0.008139	3.666	0.002006	intronic	BRD1	50176030	G	T	0.0315495	0.0169	0.0231	0.0295	0.0119	0.0757
12	68456699	rs79990588	1.806	0.0008661	1.917	0.0008392	0.3879	0.292	0.6608	0.1086	intergenic	LINC02384;RAP1	68850479	T	C	0.0365415	0.1419	0.0476	0.0008	0.002	0.0041
4	66772569	rs7679077	0.9582	0.0008671	1.148	0.0001684	0.4707	0.02503	0.7333	0.002352	intergenic	1269A;LOC10192	67638287	G	A	0.78754	0.6071	0.8761	0.7057	0.9602	0.8436
8	8242032	rs201322979	-1.353	0.0008681	-1.336	0.001854	-1.116	1.47e-05	-1.18	0.0001326	ncRNA_intronic	FAM86B3P	NA	NA	NA	NA	NA	NA	NA	NA	NA
8	8242033	rs199902738	-1.353	0.0008681	-1.336	0.001854	-1.116	1.47e-05	-1.18	0.0001326	ncRNA_intronic	FAM86B3P	NA	NA	NA	NA	NA	NA	NA	NA	NA
8	8242034	rs201303297	-1.353	0.0008681	-1.336	0.001854	-1.116	1.47e-05	-1.18	0.0001326	ncRNA_intronic	FAM86B3P	NA	NA	NA	NA	NA	NA	NA	NA	NA
3	150644879	rs13096003	0.7169	0.0008711	0.8448	0.0002207	0.3324	0.03094	0.4168	0.02258	intergenic	SELENOT;ERICH6	150362666	G	T	0.381789	0.495	0.2968	0.4713	0.2197	0.3712
3	150644885	rs13096005	0.7169	0.0008711	0.8448	0.0002207	0.3324	0.03094	0.4168	0.02258	intergenic	SELENOT;ERICH6	150362672	G	T	0.381789	0.495	0.2968	0.4713	0.2197	0.3712

7	53005724	rs57067196	0.7226	0.0008712	0.8599	0.0001437	0.4289	0.007882	0.6865	0.0001681	intergenic	07986794;POM1	53073417	A	G	0.221645	0.1577	0.3026	0.2141	0.2863	0.1738
23	86415110	rs5968922	0.652	0.0008751	0.6649	0.001288	0.5025	0.0006852	0.6671	6.14e-05	intronic	DACH2	NA	NA	NA	NA	NA	NA	NA	NA	NA
20	29483830	rs1159948585	0.956	0.0008875	1.035	0.0005212	0.9386	9.88e-06	0.8244	0.0006446	ncRNA_intronic	FRG1EP	NA	NA	NA	NA	NA	NA	NA	NA	NA
3	75685734	rs75028712	1.777	0.000888	1.926	0.0002832	0.7599	0.1209	1.149	0.02394	intergenic	INC00960;ZNF71	NA	NA	NA	NA	NA	NA	NA	NA	NA
9	41075371	rs1260798390	-0.9249	0.0008924	-0.9518	0.001133	-0.7762	6.78e-05	-0.7845	0.0005245	upstream	PGM5P2	NA	NA	NA	NA	NA	NA	NA	NA	NA
6	146454349	rs13214509	0.7453	0.000902	0.8006	0.000646	0.2827	0.06583	0.6349	0.0005184	intergenic	GRM1;RAB32	146775485	C	T	0.297724	0.499	0.2493	0.0764	0.2724	0.4499
9	74547243	rs11144004	-1.101	0.0009024	-1.76	6.55e-05	-0.3978	0.03877	-0.7353	0.002979	intronic	RORB	77162159	A	C	0.177516	0.0595	0.2363	0.2897	0.1322	0.1524
17	12464827	rs2322504	0.6599	0.0009055	0.8476	6.61e-05	0.3427	0.01856	0.5292	0.001527	intergenic	1AP2K4;LINC0067	12368144	G	A	0.735623	0.7153	0.5173	0.9259	0.6103	0.7832
3	174932371	rs9830008	0.7246	0.0009059	0.6902	0.003188	0.09081	0.5632	0.3251	0.07159	intronic	NAALADL2	174650161	G	T	0.334465	0.4583	0.3991	0.2284	0.2296	0.4121
6	154450021	rs143159747	1.666	0.0009086	1.821	0.0003025	0.8096	0.07359	1.239	0.007645	intronic	CNKSR3	154771155	A	C	0.00439297	0	0.0029	0.0008	0.0149	0.0041
9	20303117	rs190053476	1.313	0.0009089	1.301	0.001282	0.5217	0.1323	0.7682	0.03978	intergenic	SLC24A2;MLLT3	20303115	T	A	0.023762	0.001	0.0663	0.0038	0.0467	0.0204
16	50952978	rs4467072	0.7944	0.000909	0.8349	0.0007443	0.5749	0.001052	0.7951	6.79e-05	ncRNA_intronic	MIR548AI	50986889	G	T	0.223642	0.2688	0.1556	0.1702	0.2078	0.3139
2	94557721	rs1306988324	-0.9858	0.0009095	-1.032	0.000858	-0.9621	1.66e-05	-0.9116	0.000297	intergenic	i1P14;LOC10013	NA	NA	NA	NA	NA	NA	NA	NA	NA
14	27488583	rs28642378	-0.7091	0.0009108	-0.8024	0.0004704	-0.2511	0.07166	-0.2489	0.1261	ncRNA_intronic	LOC728755	27957789	G	A	0.462061	0.3978	0.6254	0.2458	0.6302	0.5317
6	166178676	rs62439025	-1.397	0.0009118	-1.4	0.001823	-1.208	1.03e-05	-1.295	0.0001102	intergenic	CDAT;LOC101929	166592164	G	C	0.0706869	0.0308	0.0706	0.0083	0.1392	0.1258
7	43487722	rs77338530	0.8101	0.0009123	0.8242	0.001244	0.7856	3.01e-05	0.6951	0.001284	intronic	HECW1	43527321	C	T	0.128195	0.0079	0.1628	0.0756	0.2177	0.2065
4	66765942	rs10010165	0.946	0.0009155	1.131	0.0001814	0.5004	0.01719	0.7599	0.001547	intergenic	1269A;LOC10192	67631660	G	C	0.788339	0.6081	0.8761	0.702	0.9612	0.8507
5	2335467	rs17586674	0.6527	0.0009159	0.6288	0.002102	0.3866	0.00481	0.6032	0.000198	intergenic	C100506858;LSIN	2335581	A	G	0.372804	0.372	0.3646	0.2587	0.4423	0.4622
1	5033932	rs11580542	0.7311	0.0009206	0.7709	0.0007719	0.6431	7.34e-05	0.5864	0.001738	intergenic	AJAP1;MIR4689	5093992	A	G	0.294928	0.1597	0.2507	0.4259	0.3012	0.2822
8	6300873	rs74826807	1.85	0.0009253	2.049	0.0002895	1.222	0.01824	1.624	0.002354	intergenic	VD1;LOC100287	6158394	C	G	0.0141773	0.001	0.0259	0.0008	0.0298	0.0215
8	6300874	rs76807954	1.85	0.0009253	2.049	0.0002895	1.222	0.01824	1.624	0.002354	intergenic	VD1;LOC100287	6158395	C	G	0.0141773	0.001	0.0259	0.0008	0.0298	0.0215
17	43896139	rs231499	0.9037	0.0009263	0.9562	0.0008411	0.2124	0.3125	0.4088	0.08822	intronic	MPP2	41973507	A	G	0.861621	1	0.9135	0.7103	0.8429	0.9059
14	49963388	rs17121917	-0.9617	0.0009323	-1.212	0.0003018	-0.74	7.13e-05	-0.7481	0.0007397	intergenic	ARF6;MIR6076	50430106	T	C	0.230431	0.12	0.1888	0.2882	0.2406	0.2853
1	885006	rs112590444	1.236	0.0009331	1.176	0.002458	0.4722	0.135	0.8025	0.01709	intergenic	AM41C;LINC025	NA	NA	NA	NA	NA	NA	NA	NA	NA
14	27530197	rs2333048	-0.7234	0.0009334	-0.8413	0.000372	-0.245	0.08606	-0.2352	0.1525	ncRNA_intronic	LOC728755	27999403	G	A	0.532947	0.3948	0.6657	0.4894	0.6272	0.5429
14	27503910	rs12433862	-0.7135	0.0009342	-0.7867	0.0006013	-0.2391	0.08937	-0.2355	0.1465	ncRNA_intronic	LOC728755	27973116	T	G	0.443291	0.3958	0.6254	0.1725	0.6302	0.5368
9	20302984	rs75858952	1.308	0.0009353	1.298	0.001306	0.52	0.1337	0.7667	0.04013	intergenic	SLC24A2;MLLT3	20302982	T	C	0.0261581	0.002	0.072	0.0038	0.0477	0.0266
17	43864540	rs79851944	0.828	0.0009372	0.9785	0.0001901	0.3072	0.104	0.429	0.04726	downstream	CD300LG	41941908	C	A	0.784145	0.999	0.8602	0.5008	0.7932	0.8824
17	43867497	rs231535	0.8295	0.0009387	0.972	0.0001972	0.3384	0.08143	0.4995	0.02256	intergenic	CD300LG;MPP2	41944865	T	G	0.872604	1	0.8833	0.8094	0.8032	0.8906
18	10310198	rs1123784	1.032	0.0009387	1.098	0.0006137	0.8922	4.98e-05	1.018	4.26e-05	intergenic	VAPA;LINC01254	10310195	A	C	0.154752	0.255	0.2291	0.09	0.1004	0.1421
14	27510299	rs8007793	-0.707	0.0009396	-0.7759	0.0006274	-0.2347	0.09242	-0.2042	0.209	ncRNA_intronic	LOC728755	27979505	G	A	0.465655	0.3958	0.6254	0.2587	0.6302	0.5348
22	49811098	rs74280003	4.889	0.0009433	4.886	0.0007117	2.648	0.01672	3.618	0.002627	intronic	BRD1	50204746	G	A	0.0147764	0.0159	0.0144	0.0272	0.002	0.0102
14	27446879	rs2096306	-0.7218	0.0009527	-0.8005	0.0006103	-0.2613	0.0659	-0.2583	0.1151	ncRNA_intronic	LOC728755	27916085	A	G	0.459265	0.3919	0.6239	0.23	0.6372	0.5389
4	94812518	rs117961662	3.397	0.0009582	3.563	0.0005837	1.292	0.1364	2.011	0.02277	intronic	BMPR1B	95733669	A	G	0.00239617	0.0099	0	0	0	0.002
2	161069351	rs2161846	0.7828	0.0009693	0.79	0.00173	0.7158	9.64e-05	0.6878	0.0009531	intergenic	RBMS1;TANK	161925862	C	T	0.119609	0.2222	0.1009	0.0068	0.1809	0.1166
17	43897522	rs58713010	0.871	0.0009753	0.9153	0.001077	0.2418	0.2335	0.3818	0.09836	intronic	MPP2	41974890	TACTC	T	0.825679	1	0.8991	0.5862	0.838	0.9049
2	161108361	rs2194506	0.8074	0.0009775	0.7988	0.002188	0.7394	9.66e-05	0.7128	0.0009323	intergenic	RBMS1;TANK	161964872	G	A	0.103834	0.2063	0.0965	0.0053	0.171	0.0675
15	62610634	rs28588975	0.9057	0.0009846	0.7613	0.007971	0.234	0.2904	0.5254	0.02952	intergenic	IR6085;MGC158	62902833	G	T	0.0954473	0.1161	0.0331	0.1452	0.0954	0.0511
15	23543183	rs11853016	0.6943	0.0009891	0.7217	0.001064	0.6158	7.55e-05	0.725	5.05e-05	intergenic	OLGA6L2;MIR45	23788330	A	G	0.264377	0.1974	0.2262	0.2322	0.3877	0.2771
11	88048807	rs182992810	1.478	0.0009893	1.446	0.001796	0.7699	0.05303	1.039	0.01374	intergenic	LINC02711;RAB3	87781975	G	A	0.0165735	0.003	0.0504	0.0023	0.0189	0.0235
10	55854096	rs35870924	1.597	0.0009961	1.763	0.0002889	0.9207	0.03427	1.35	0.002977	intergenic	PCDH15;ZWINT	57613856	T	C	0.0303514	0.0079	0.0231	0.003	0.0318	0.0941
1	231876203	rs1009587	0.6379	0.0009975	0.6076	0.002536	0.2231	0.0986	0.5198	0.00103	ncRNA_intronic	TSNAX-DISC1	232011949	G	A	0.404353	0.5446	0.4207	0.2905	0.3926	0.4141
8	114487970	rs117265998	1.753	0.001003	1.891	0.0003429	1.118	0.01956	1.294	0.009973	intergenic	CSMD3;TRPS1	115500199	C	T	0.0221645	0	0.0447	0.0136	0.0567	0.0051
4	189610603	rs2923344	-0.9006	0.001006	-0.9701	0.0008435	-0.8671	8.83e-06	-0.9019	7.34e-05	intergenic	IC02508;LINC012	NA	NA	NA	NA	NA	NA	NA	NA	NA
9	41250014	rs1367642451	1.003	0.001006	0.9707	0.002371	0.8461	4e-05	0.9559	0.0001028	intergenic	4477A;LOC10798	NA	NA	NA	NA	NA	NA	NA	NA	NA
4	12747914	rs7692152	1.228	0.001007	1.332	0.0004634	0.6338	0.02796	1.028	0.00179	intergenic	LINC02270;RAB2	12749538	C	T	0.123203	0.0169	0.0648	0.3601	0.0229	0.0573
17	68764577	rs9915487	0.7378	0.001009	0.8972	0.0001236	0.3498	0.0377	0.3816	0.04457	intergenic	LINC01482;ABCA	66760718	C	T	0.17472	0.0933	0.1873	0.1271	0.2793	0.2065
4	12752151	rs184884467	1.253	0.00101	1.365	0.0004474	0.4987	0.0952	1.027	0.002102	intergenic	LINC02270;RAB2	12753775	T	C	0.0866613	0.0169	0.0548	0.2269	0.0229	0.0573
12	98096488	rs7314378	0.6424	0.001014	0.7594	0.0002396	0.1018	0.4562	0.2432	0.1202	intergenic	MIR4303;SLC9A7F	98490266	C	T	0.590256	0.7669	0.4798	0.503	0.499	0.6984
15	63351325	rs11637751	0.8457	0.001014	0.9465	0.000384	0.2006	0.3169	0.3616	0.1087	intronic	CA12	63643524	G	A	0.141773	0.003	0.2622	0.1732	0.1461	0.1524
1	231878572	rs61836980	0.638	0.00102	0.6102	0.002393	0.2217	0.1014	0.52	0.001011	ncRNA_intronic	TSNAX-DISC1	232014318	G	A	0.400958	0.5427	0.4193	0.2791	0.3936	0.4141
5	118392868	rs116732062	1.542	0.001022	1.726	0.0002315	0.9312	0.02762	1.058	0.01737	ncRNA_intronic	LINC02208	117728563	T	A	0.014976	0	0.0288	0.0008	0.0477	0.0061
18	10500701	rs522420	0.6587	0.001025	0.6496	0.001977	0.6696	3.59e-06	0.7545	8.55e-06	intergenic	APCDD1;NAPG	10500698	T	C	0.564896	0.6399	0.5115	0.7179	0.4384	0.4489
17	12466022	rs7218628	0.648	0.001027	0.8157	9.82e-05	0.3365	0.0205	0.5147	0.001807	intergenic	1AP2K4;LINC0067	12369339	G	A	0.747204	0.7431	0.5274	0.9259	0.6362	0.7802
22	37015166	rs4821584	0.6433	0.001032	0.7494	0.0003578	0.2848	0.04208	0.4447	0.006589	intronic	TST	37411207	C	T	0.541334	0.5952	0.5418	0.5923	0.4811	0.4785
3	741351	rs2729094	-0.7504	0.001033	-0.716	0.00269	-0.625	7.37e-05	-0.6896	0.0001777	ncRNA_intronic	LINC01266	783035	T	C	0.603634	0.62	0.6599	0.4145	0.7435	0.6585
18	10506212	rs500029	-0.6488	0.001033	-0.6325	0.002357	-0.627	1.02e-05	-0.7068	2.33e-05	intergenic	APCDD1;NAPG	10506209	T	C	0.569489	0.6696	0.5043	0.7156	0.4364	0.4519
20	8351174	rs2175041	0.9409	0.001033	1.064	0.0002996	0.7744	0.0005305	0.9681	8.44e-05	intronic	PLCB1	8331821	C	T	0.117212	0	0.0764	0.211	0.1849	0.0706
11	70619912	rs60654869	-0.7192	0.001034	-0.7259	0.001553	-0.5904	7.36e-05	-0.6304	0.0003045	intronic	SHANK2	70466017	C	T	0.255791	0.3671	0.3847	0.0318	0.2853	0.3221
14	27500452	rs8022659	-0.6997	0.001039	-0.7922	0.0005399	-0.2345	0.0938	-0.2322	0.1513	ncRNA_intronic	LOC728755	27969658	C	T	0.455871	0.3958	0.6254	0.2216	0.6302	0.5348
14	34244860	rs60402373	0.9675	0.001045	1.066	0.0004337	0.4376	0.05226	0.487	0.06159	intergenic	EGLN3;SPTSSA	34714066	G	A	0.104832	0.1597	0.062	0.1135	0.0954	0.0767
14	34244977	rs60866059	0.9675	0.001045	1.066	0.0004337	0.4441	0.04903	0.487	0.06159	intergenic	EGLN3;SPTSSA	34714183	T	C	0.105232	0.1607	0.062	0.1135	0.0954	0.0777
10	38643136	rs879656274	-0.9294	0.001046	-0.9418	0.001677	-0.81	4.23e-05	-0.8031	0.0004831	intergenic	IC00999;ACTR3B	NA	NA	NA	NA	NA	NA	NA	NA	NA
8	6293953	rs34140352	1.743	0.001047	1.929	0.0003353	1.146	0.01751	1.5	0.002964	intergenic	VD1;LOC100287	6151474	G	A	0.014377	0.001	0.0259	0.0008	0.0308	0.0215
20	3388465	rs75466028	1.782	0.00105	1.854	0.0007522	1.534	0.001723	1.905	0.0001386	intronic	C20orf194	3369112	A	C	0.0157748	0	0.0303	0.0008	0.0527	0.0041
17	21688263	rs1187382269	0.9714	0.001051	1.086	0.0006899	0.8992	1.63e-05	0.9661	8.92e-05	intergenic	INC02693;KCNJ1	NA	NA	NA	NA	NA	NA	NA	NA	NA
20	29247755	rs1423535535	-1.236	0.001054	-1.229	0.002059	-1.13	8.14e-06	-1.14	0.0001607	intergenic	FRG1DP;FRG2EP	NA	NA	NA	NA	NA	NA	NA	NA	NA
17	43897883	rs231498	0.8622	0.001055	0.9071	0.001149	0.2395	0.2359	0.3751	0.1033	intronic	MPP2	41975251	C	G	0.826078	1	0.8991	0.5877	0.838	0.9049
17	43898579	rs231497	0.8622	0.001055	0.9071	0.001149	0.2395	0.2359	0.3751	0.1033	intronic	MPP2	41975947	A	G	0.825679	1	0.8991	0.5862	0.838	0.9049
4	130545002	rs6851021	-0.7366	0.001058	-0.7503	0.001567	-0.4545	0.003565	-0.8263	2.47e-05	intergenic	INC02479;SNHG2	131466157	G	A	0.229433	0.0933	0.3818	0.1467	0.4056	0.1922
21	30361124	rs2832832	0.643	0.001058	0.6863	0.0008435	0.1455	0.2974	0.376	0.0205	intergenic	TAP23-1;KRTAP1	31733442	T	G	0.321086	0.2649	0.3473	0.1785	0.4503	0.4202
4	66769590	rs1375887	0.9311	0.001064	1.131	0.0001814	0.4945	0.01847	0.7521	0.001715	intergenic	1269A;LOC10192	67635308	G	C	0.788339	0.6081	0.8761	0.702	0.9612	0.8507
17	8109193	rs3027205	2.672	0.001064	2.887	0.0004053	1.342	0.05431	2.024	0.00643	exonic	ALOXE3	8012511	T	C	0.0453275	0.0179	0.0072	0.1127	0.001	0.0552
2	143954195	rs9678811	1.134	0.001067	1.252	0.0004002	0.5807	0.03029	0.8707	0.003991	ncRNA_intronic	LOC101928386	144711762	C	T	0.14397	0.1637	0.1095	0.2307	0.0408	0.137
17	43894071	rs231503	0.8817	0.001069	0.9303	0.0009871	0.2495	0.2286	0.3893	0.1003	intronic	MPP2	41971439	G	A	0.840655	1	0.9135	0.6316	0.8419	0.9059
4	59471517	rs73820168	1.039	0.001071	1.231	0.0001338	0.3985	0.1238	0.5014	0.08094	intergenic	C02429;MIR548	60337235	C	T	0.0557109	0.1161	0.0634	0.0189	0.0586	0.0348
14	27515317	rs8018341	-0.7191	0.001074	-0.8168	0.0005341	-0.2389	0.09175	-0.2262	0.1683	ncRNA_intronic	LOC728755	27984523	G	A	0.534545	0.3948	0.6686	0.4955	0.6282	0.5399
17	43868220	rs231533	0.853	0.001074	1.038	0.0001416	0.321	0.1215	0.4906	0.03195	intergenic	CD300LG;MPP2	41945588	A	G	0.904353	1	0.9006	0.9107	0.8101	0.8967
14	22461046	rs1882705	-0.6484	0.001089	-0.6186	0.002662	-0.6374	1.73e-05	-0.5952	0.0003736	ncRNA_intronic	LOC105370401	22930038	C	T	0.469848	0.1687	0.4006	0.6861	0.5209	0.4847
17	43885837	rs231516	0.8561	0.00109	0.899	0.001198	0.2666	0.1859	0.3634	0.1122	intronic	MPP2	41963205	G	A	0.815296	1	0.8991	0.5477	0.837	0.9049
2	89667821	rs1264130132	1.342	0.001094	1.279	0.003003	0.5252	0.1505	0.8012	0.04052	intergenic	C101927050;LSP	NA	NA	NA	NA	NA	NA	NA	NA	NA
4	130571772	rs62319787	-0.8115	0.001096	-0.8699	0.001066	-0.5354	0.001723	-0.8696	3.92e-05	intergenic	INC02479;SNHG2	131492927	T	C	0.161941	0.0308	0.1916	0.0499	0.3966	0.1861
6	166330774	rs7757549	-0.8661	0.001098	-0.8069	0.003302	-0.6889	9.94e-05	-0.6517	0.001896	intronic	SFT2D1	166744262	C	G	0.759984	0.9554	0.7305	0.5613	0.7545	0.8538
13	101600045	rs139110039	-0.7924	0.0011	-0.7563	0.002787	-0.4286	0.005887	-0.9473	4.69e-06	intronic	ITGBL1	102252395	G	A	0.240815	0.4286	0.2406	0.0446	0.2644	0.2883
17	21386798	rs113533177	1.147	0.001101	1.34	0.0001926	0.6352	0.03181	0.9517	0.002788	intronic	KCNJ12	21290110	G	A	0.033147	0.001	0.0476	0.0197	0.0606	0.046
18	10505681	rs656067	-0.6517	0.001101	-0.643	0.002089	-0.6576	5.02e-06	-0.7571	8.7e-06	intergenic	APCDD1;NAPG	10505678	T	C	0.567492	0.6667	0.5014	0.7156	0.4334	0.4499
20	30848652	rs1407099197	0.8851	0.001101	1.026	0.0004226	0.892	3.89e-06	0.7566	0.0006922	intergenic	FRG1BP;DEFB11	NA	NA	NA	NA	NA	NA	NA	NA	NA
22	16427955	rs78355888	-1.016	0.001101	-1.016	0.001891	-0.8648	4.01e-05	-0.8296	0.0007417	intergenic	DUXAP8;CCT8L2	NA	NA	NA	NA	NA	NA	NA	NA	NA
2	161046916	rs16845509	0.7855	0.001102	0.7736	0.002454	0.7215	9.77e-05	0.6766	0.001276	intergenic	RBMS1;TANK	161903427	C	T	0.109026	0.2103	0.1009	0.0068	0.1809	0.0746
4	66761971	rs11732387	0.9165	0.001109	1.121	0.00017	0.5099	0.01417	0.7082	0.002692	intergenic	1269A;LOC10192	67627689	C	A	0.774161	0.5982	0.8689	0.6649	0.9612	0.8436
18	10505909	rs657024	0.649	0.001109	0.637	0.002182	0.6357	9.15e-06	0.7272	1.54e-05	intergenic	APCDD1;NAPG	10505906	G	A	0.560703	0.6409	0.4942	0.7148	0.4314	0.4499
18	74224967	rs74502708	1.169	0.001109	1.262	0.0005155	0.3764	0.2251	0.7225	0.02742	intergenic	TIMM21;CYB5A	71892202	T	A	0.0401358	0.0258	0.0231	0.0204	0.0527	0.0808
4	81210266	rs57051776	0.8341	0.00111	0.8405	0.0014	0.5496	0.002708	0.8571	4.13e-05	intronic	PRKG2	NA	NA	NA	NA	NA	NA	NA	NA	NA
4	82482410	rs13114300	0.6501	0.001116	0.6678	0.001268	0.4504	0.001291	0.6597	8.21e-05	downstream	TMEM150C	83403563	G	T	0.515375	0.4633	0.647	0.4932	0.4523	0.5706
18	10492979	rs493201	-0.644	0.001116	-0.5768	0.004986	-0.6481	5.52e-06	-0.7365	1.09e-05	intergenic	APCDD1;NAPG	10492976	T	C	0.531749	0.6478	0.4971	0.5938	0.4324	0.455
15	99163567	rs2602027	0.7767	0.001121	0.9159	0.0002328	0.5575	0.001786	0.7997	9.52e-05	intronic	TTC23	99703772	C	T	0.205871	0.0962	0.232	0.3079	0.1809	0.1881
22	37015139	rs4821582	-0.6364	0.001123	-0.7451	0.0003699	-0.2826	0.04287	-0.4525	0.005521	intronic	TST	37411180	T	A	0.510783	0.5952	0.5346	0.4803	0.4811	0.4785
22	37015149	rs4821583	-0.6364	0.001123	-0.7451	0.0003699	-0.2826	0.04287	-0.4525	0.005521	intronic	TST	37411190	T	C	0.510783	0.5952	0.5346	0.4803	0.4811	0.4785
17	43873915	rs231525	0.8592	0.001125	0.9037	0.001232	0.2287	0.2587	0.3686	0.11	intergenic	CD300LG;MPP2	41951283	C	T	0.825879	1	0.8991	0.587	0.838	0.9049

17	43882975	rs124719	0.8592	0.001125	0.9037	0.001232	0.2287	0.2587	0.3686	0.11	exonic	MPP2	41960343	T	C	0.826278	1	0.9006	0.587	0.838	0.9059
17	43886715	rs231515	0.8592	0.001125	0.9037	0.001232	0.2287	0.2587	0.3686	0.11	intronic	MPP2	41964083	A	G	0.825679	1	0.8991	0.5862	0.838	0.9049
17	43889102	rs231510	0.8592	0.001125	0.9037	0.001232	0.2287	0.2587	0.3686	0.11	intronic	MPP2	41966470	T	C	0.825679	1	0.8991	0.5862	0.838	0.9049
17	43890820	rs34694583	0.8592	0.001125	0.9037	0.001232	0.2287	0.2587	0.3686	0.11	intronic	MPP2	41968188	A	AG	0.825679	1	0.8991	0.5862	0.838	0.9049
17	43891663	rs231507	0.8592	0.001125	0.9037	0.001232	0.2287	0.2587	0.3686	0.11	intronic	MPP2	41969031	A	G	0.825679	1	0.8991	0.5862	0.838	0.9049
17	43892900	rs231505	0.8592	0.001125	0.9037	0.001232	0.2287	0.2587	0.3686	0.11	intronic	MPP2	41970268	A	T	0.825679	1	0.8991	0.5862	0.838	0.9049
17	43894837	rs76696580	0.8592	0.001125	0.9037	0.001232	0.2287	0.2587	0.3686	0.11	intronic	MPP2	41972205	AGGCAGGAG	A	0.825679	1	0.8991	0.5862	0.838	0.9049
17	43894935	rs231501	0.8592	0.001125	0.9037	0.001232	0.2287	0.2587	0.3686	0.11	intronic	MPP2	41972303	C	T	0.825679	1	0.8991	0.5862	0.838	0.9049
17	43896006	rs231500	0.8592	0.001125	0.9037	0.001232	0.2286	0.2591	0.3669	0.1118	intronic	MPP2	41973374	T	C	0.825679	1	0.8991	0.5862	0.838	0.9049
17	43899368	rs537165	0.8592	0.001125	0.9037	0.001232	0.2287	0.2587	0.3686	0.11	intronic	MPP2	41976736	C	T	0.825479	1	0.8991	0.5855	0.838	0.9049
17	43899372	rs537169	0.8592	0.001125	0.9037	0.001232	0.2287	0.2587	0.3686	0.11	intronic	MPP2	41976740	G	A	0.825479	1	0.8991	0.5855	0.838	0.9049
17	43899380	rs231495	0.8592	0.001125	0.9037	0.001232	0.2287	0.2587	0.3686	0.11	intronic	MPP2	41976748	G	C	0.825479	1	0.8991	0.5855	0.838	0.9049
17	43899392	rs58467247	0.8592	0.001125	0.9037	0.001232	0.2287	0.2587	0.3686	0.11	intronic	MPP2	41976760	CCA	C	0.825479	1	0.8991	0.5855	0.838	0.9049
17	43899560	rs231494	0.8592	0.001125	0.9037	0.001232	0.2287	0.2587	0.3686	0.11	intronic	MPP2	41976928	G	C	0.825479	1	0.8991	0.5855	0.838	0.9049
17	43899630	rs231493	0.8592	0.001125	0.9037	0.001232	0.2287	0.2587	0.3686	0.11	intronic	MPP2	41976998	G	C	0.825479	1	0.8991	0.5855	0.838	0.9049
17	43865489	rs231537	0.8147	0.001133	0.9652	0.0002328	0.2984	0.1139	0.4182	0.05306	intergenic	CD300LG;MPP2	41942857	C	T	0.784744	1	0.8602	0.5015	0.7932	0.8834
7	53007569	rs1919941	0.7028	0.001139	0.8612	0.0001402	0.4296	0.007476	0.6751	0.0002034	intergenic	07986794;POM1	53075262	C	T	0.221645	0.1577	0.3026	0.2141	0.2863	0.1738
1	118678154	rs76046637	1.672	0.001149	1.635	0.00199	1.026	0.03149	1.252	0.01301	intergenic	SPAG17;TBX15	119220777	A	G	0.0155751	0.002	0.0576	0	0.0328	0.0031
4	12743530	rs6448944	1.233	0.001153	1.341	0.0005166	0.5347	0.07674	0.9996	0.002849	intergenic	LINC02270;RAB2	12745154	A	G	0.0874601	0.0169	0.0562	0.2292	0.0239	0.0562
4	12743675	rs6448946	1.233	0.001153	1.341	0.0005166	0.5347	0.07674	0.9996	0.002849	intergenic	LINC02270;RAB2	12745299	C	T	0.0874601	0.0169	0.0562	0.2292	0.0239	0.0562
16	84400370	rs9927653	-0.6661	0.001153	-0.6696	0.001899	-0.09671	0.4707	-0.3114	0.05015	intronic	ATP2C2	84433976	A	G	0.368411	0.4673	0.2954	0.2821	0.4414	0.3599
3	99653554	rs141155559	1.982	0.001155	2.111	0.0005342	1.123	0.04958	1.549	0.007351	intronic	COL8A1	99372398	T	C	0.00219649	0	0.0014	0	0.0099	0
20	30175330	rs1355317420	-0.8852	0.001159	-0.8067	0.004294	-0.8069	3.92e-05	-0.8123	0.0003639	intergenic	IR4477A;LINC015	NA	NA	NA	NA	NA	NA	NA	NA	NA
2	233217295	rs111345303	0.8668	0.001161	0.8927	0.001241	0.5462	0.00777	0.8462	0.0001879	intergenic	INPP5D;ATG16L1	234125941	G	T	0.0876597	0.1032	0.0865	0.0522	0.1551	0.0511
4	12760079	rs77540974	1.191	0.001161	1.295	0.0005137	0.5478	0.067	0.9609	0.00338	intergenic	LINC02270;RAB2	12761703	T	A	0.0764776	0.0179	0.0504	0.1899	0.0229	0.0573
17	66954602	rs4791002	1.17	0.001161	1.365	0.0002351	0.7579	0.01249	0.836	0.01105	intergenic	CACNG5;CACNG	64950718	G	A	0.0577077	0.0377	0.072	0.0998	0.0447	0.0245
4	12743467	rs7689041	1.232	0.001163	1.34	0.0005199	0.5339	0.07727	0.9991	0.002866	intergenic	LINC02270;RAB2	12745091	A	G	0.0874601	0.0169	0.0562	0.2292	0.0239	0.0562
4	66768969	rs6552032	0.9259	0.001163	1.124	0.0002014	0.4905	0.01976	0.7444	0.001936	intergenic	1269A;LOC10192	67634687	C	A	0.788538	0.6081	0.8761	0.7027	0.9612	0.8507
13	101605638	rs146546354	-0.8371	0.001163	-0.8087	0.002691	-0.4148	0.01136	-0.9665	9.34e-06	intronic	ITGBL1	102257988	ATG	A	0.246006	0.4504	0.2378	0.0461	0.2644	0.2924
9	33799920	rs1302906086	-1.472	0.00117	-1.387	0.003484	-1.134	4.45e-05	-1.036	0.001463	downstream	PRSS3	NA	NA	NA	NA	NA	NA	NA	NA	NA
6	32182519	rs184003	0.9979	0.001173	1.035	0.001409	0.8973	9.06e-05	0.7603	0.003767	intronic	AGER	32150296	C	A	0.155551	0.1458	0.1009	0.1899	0.0865	0.229
8	110991850	rs59714343	1.175	0.001173	1.26	0.0006109	0.3666	0.2324	0.8427	0.008953	ncRNA_intronic	LINC01608	112004079	G	A	0.0507188	0.0079	0.0476	0.0431	0.0527	0.1053
12	70941795	rs913785340	-0.8971	0.001174	-0.9227	0.001593	-0.6723	0.0004682	-0.9374	4.19e-05	intergenic	PTPRR;TSPAN8	NA	NA	NA	NA	NA	NA	NA	NA	NA
17	43899218	rs231496	0.8555	0.001178	0.9	0.001287	0.2251	0.2665	0.365	0.1135	intronic	MPP2	41976586	T	G	0.825479	1	0.8991	0.5855	0.838	0.9049
2	161088153	rs13426915	0.7482	0.001181	0.7622	0.00183	0.6952	7.26e-05	0.6555	0.00112	intergenic	RBMS1;TANK	161944664	C	G	0.203674	0.2163	0.1441	0.3056	0.1889	0.1104
22	49852953	rs7285615	1.964	0.001184	2.143	0.0005705	0.8546	0.0493	1.419	0.004035	upstream	ZBED4	50246601	G	A	0.0944489	0.0278	0.0432	0.3018	0.005	0.0112
18	10510579	rs565913	0.6461	0.001186	0.6355	0.002372	0.6561	5.17e-06	0.741	1.13e-05	intergenic	APCDD1;NAPG	10510576	T	C	0.570088	0.6677	0.5115	0.7141	0.4384	0.4519
1	175455723	rs859451	-0.6735	0.001188	-0.7849	0.0005234	-0.1164	0.3956	-0.3472	0.03187	intronic	TNR	175424859	G	C	0.634784	0.3442	0.6441	0.9024	0.5775	0.6247
17	43892823	rs231506	0.8797	0.001188	0.9562	0.0008411	0.2044	0.3307	0.3965	0.09772	intronic	MPP2	41970191	T	C	0.861222	1	0.9135	0.7095	0.8419	0.9059
1	66801636	rs6588220	0.8799	0.001192	0.9158	0.001291	0.8824	3.82e-05	0.9558	5.49e-05	upstream	INSL5	67267319	A	G	0.864018	0.9901	0.889	0.7564	0.83	0.8967
1	66804425	rs1353716	0.8799	0.001192	0.9158	0.001291	0.8824	3.82e-05	0.9558	5.49e-05	intergenic	INSL5;DNAI4	67270108	C	G	0.86262	0.9901	0.889	0.7504	0.831	0.8967
12	98117067	rs57634848	2.122	0.001193	2.339	0.0003961	1.512	0.01255	1.895	0.003424	intergenic	IR4303;SLC9A7	98510845	A	T	0.0441294	0	0.0259	0.1415	0.0159	0
23	100767703	rs1343215	0.6505	0.001193	0.717	0.001128	0.06743	0.5913	0.245	0.09986	intergenic	SYTL4;CSTF2	NA	NA	NA	NA	NA	NA	NA	NA	NA
18	10509963	rs519783	0.6462	0.001194	0.6359	0.00238	0.658	4.85e-06	0.7425	1.09e-05	intergenic	APCDD1;NAPG	10509960	G	A	0.570288	0.6677	0.5115	0.7148	0.4384	0.4519
18	10510230	rs607946	0.6462	0.001194	0.6359	0.00238	0.658	4.85e-06	0.7425	1.09e-05	intergenic	APCDD1;NAPG	10510227	A	G	0.570288	0.6677	0.5115	0.7148	0.4384	0.4519
4	66771568	rs7672554	0.924	0.001198	1.112	0.0002322	0.4349	0.03714	0.6737	0.004735	intergenic	1269A;LOC10192	67637286	G	A	0.787939	0.6091	0.8761	0.7057	0.9602	0.8436
14	86465249	rs7140308	-1.178	0.00121	-1.225	0.001797	-0.8447	0.0002317	-1.205	6.36e-05	intergenic	NC02328;LINC011	86931593	G	A	0.757588	0.8502	0.8069	0.4175	0.9354	0.9039
1	125070260	rs1226978594	-1.242	0.001212	-1.277	0.0009472	-1.357	2.18e-06	-1.366	2.02e-05	intergenic	NONE;NONE	NA	NA	NA	NA	NA	NA	NA	NA	NA
1	4756976	rs77759903	1.642	0.001221	1.776	0.0004904	0.6027	0.1875	1.075	0.02654	intronic	AJAP1	4817036	C	G	0.0371406	0.0179	0.0072	0.0008	0.0189	0.1462
1	4757615	rs34072519	1.642	0.001221	1.776	0.0004904	0.6027	0.1875	1.075	0.02654	intronic	AJAP1	4817675	T	G	0.0383387	0.0228	0.0072	0.0023	0.0179	0.1462
1	4758543	rs117179206	1.642	0.001221	1.776	0.0004904	0.6027	0.1875	1.075	0.02654	intronic	AJAP1	4818603	G	A	0.0383387	0.0228	0.0072	0.0023	0.0179	0.1462
1	4759105	rs3753719	1.642	0.001221	1.776	0.0004904	0.6027	0.1875	1.075	0.02654	intronic	AJAP1	4819165	T	C	0.0383387	0.0228	0.0072	0.0023	0.0179	0.1462
1	4760989	rs3766974	1.642	0.001221	1.776	0.0004904	0.6027	0.1875	1.075	0.02654	intronic	AJAP1	4821049	A	G	0.0385383	0.0228	0.0072	0.0023	0.0179	0.1472
1	4763021	rs3835542	1.642	0.001221	1.776	0.0004904	0.6027	0.1875	1.075	0.02654	intronic	AJAP1	4823081	AC	A	0.0371406	0.0179	0.0072	0.0008	0.0179	0.1472
1	4763079	rs111622387	1.642	0.001221	1.776	0.0004904	0.6027	0.1875	1.075	0.02654	intronic	AJAP1	4823139	C	T	0.0375399	0.0179	0.0072	0.0023	0.0179	0.1472
4	59463041	rs57970893	1.023	0.001221	1.236	0.0001258	0.4226	0.1014	0.4975	0.08315	intergenic	C02429;MIR548	60328759	C	A	0.0561102	0.1171	0.0634	0.0189	0.0596	0.0348
17	43878634	rs124722	0.8473	0.001221	0.8891	0.001355	0.2615	0.1952	0.3563	0.1199	intronic	MPP2	41956002	T	G	0.815695	1	0.9006	0.5484	0.837	0.9049
22	22323586	rs796804659	-1.101	0.001223	-1.196	0.001166	-0.6134	0.004227	-1.098	7.47e-05	downstream	BMS1P20	NA	NA	NA	NA	NA	NA	NA	NA	NA
17	43886039	rs170766	0.8483	0.001225	0.8917	0.001325	0.2578	0.2014	0.3515	0.1251	intronic	MPP2	41963407	C	G	0.815695	1	0.8991	0.5492	0.837	0.9049
3	141819980	rs78193590	-0.9008	0.001229	-0.86	0.003156	-0.7197	0.0002276	-0.9012	8.61e-05	downstream	GRK7	NA	NA	NA	NA	NA	NA	NA	NA	NA
4	66772252	rs7679859	0.9226	0.001229	1.114	0.0002315	0.473	0.0244	0.7457	0.002049	intergenic	1269A;LOC10192	67637970	C	G	0.787141	0.6062	0.8761	0.705	0.9602	0.8436
13	101558291	rs74119856	-0.7297	0.001229	-0.6944	0.003483	-0.3942	0.008546	-0.7722	4.91e-05	intronic	ITGBL1	102210642	A	G	0.288139	0.4524	0.3156	0.0598	0.3052	0.3906
13	101581956	rs8001356	-0.7192	0.00123	-0.6829	0.003487	-0.3799	0.01011	-0.7461	6.44e-05	intronic	ITGBL1	102234306	C	T	0.306909	0.4583	0.3228	0.1225	0.3062	0.3896
19	47966825	rs12983148	0.6334	0.001231	0.789	0.0001965	0.4428	0.001535	0.6812	4.19e-05	intergenic	SNAR-C2;BSPH1	48470082	T	C	0.366613	0.3423	0.5	0.0802	0.5507	0.4949
19	47966837	rs12981784	0.6334	0.001231	0.789	0.0001965	0.4428	0.001535	0.6812	4.19e-05	intergenic	SNAR-C2;BSPH1	48470094	G	A	0.366613	0.3423	0.5	0.0802	0.5507	0.4949
19	47966840	rs12981328	0.6334	0.001231	0.789	0.0001965	0.4428	0.001535	0.6812	4.19e-05	intergenic	SNAR-C2;BSPH1	48470097	C	G	0.366613	0.3423	0.5	0.0802	0.5507	0.4949
19	47966964	rs12611226	0.6334	0.001231	0.789	0.0001965	0.4428	0.001535	0.6812	4.19e-05	intergenic	SNAR-C2;BSPH1	48470221	G	A	0.36881	0.3403	0.5014	0.0809	0.5626	0.4939
2	176552260	rs10084388	0.6306	0.001234	0.5679	0.004849	0.3818	0.007324	0.603	0.0002605	intergenic	MTX2;MIR1246	177416988	G	A	0.566494	0.8214	0.6427	0.3971	0.5348	0.5112
22	22349441	rs1226946387	-1.061	0.001234	-0.8874	0.007357	-0.9274	3.04e-05	-0.9773	0.0002316	intergenic	MS1P20;ZNF280	NA	NA	NA	NA	NA	NA	NA	NA	NA
6	15339631	rs7763132	0.8089	0.001237	0.869	0.0009528	0.7602	6.71e-05	0.8493	8.9e-05	intronic	JARID2	15339862	G	A	0.177716	0.2381	0.1239	0.2315	0.1252	0.135
5	170460906	rs114980607	2.094	0.00124	2.091	0.001629	1.066	0.01609	1.797	0.0004959	intronic	KCNIP1	169887910	C	A	0.061901	0.0456	0.0115	0.1483	0.0099	0.0511
1	4758323	rs113226922	1.553	0.001243	1.674	0.0005267	0.355	0.3903	0.9621	0.03244	intronic	AJAP1	4818383	A	G	0.0559105	0.0228	0.0173	0.0635	0.0179	0.1462
1	66808406	rs1498348	0.8763	0.001243	0.9126	0.001338	0.8771	4.27e-05	0.9514	5.95e-05	intergenic	INSL5;DNAI4	67274089	A	G	0.863019	0.9901	0.889	0.7519	0.83	0.8978
1	118685168	rs80134218	1.663	0.001243	1.654	0.001834	1.015	0.0334	1.238	0.01405	intergenic	SPAG17;TBX15	119227791	G	A	0.0157748	0.003	0.0576	0	0.0328	0.0031
17	68764007	rs142743450	0.7273	0.001247	0.8812	0.0001689	0.3244	0.05751	0.3622	0.05866	intergenic	INC01482;ABCA	66760148	G	A	0.164137	0.0595	0.1873	0.1127	0.2793	0.2065
5	129185363	rs567992074	1.31	0.001249	1.306	0.001983	0.3896	0.2329	0.447	0.2288	intergenic	ISOC1;MIR4460	128521056	T	G	0.0794728	0.1637	0.0418	0.1074	0.0348	0.0276
9	33799915	rs1387017778	-1.463	0.00125	-1.379	0.00367	-1.159	3.51e-05	-1.091	0.0009892	downstream	PRSS3	NA	NA	NA	NA	NA	NA	NA	NA	NA
1	4758862	rs3753717	1.638	0.001255	1.772	0.0005047	0.5996	0.1899	1.072	0.02699	intronic	AJAP1	4818922	G	A	0.0383387	0.0228	0.0072	0.0023	0.0179	0.1462
1	4759673	rs3820256	1.638	0.001255	1.772	0.0005047	0.5996	0.1899	1.072	0.02699	intronic	AJAP1	4819733	G	A	0.0375399	0.0179	0.0072	0.0023	0.0179	0.1472
12	98095490	rs11109346	0.6235	0.001258	0.7353	0.0002605	0.1572	0.2647	0.2478	0.12	intergenic	IR4303;SLC9A7	98489268	A	C	0.29373	0.2103	0.4294	0.295	0.3181	0.2566
3	129885635	rs150789881	2.945	0.00126	3.027	0.0007405	1.693	0.0373	2.217	0.01008	intronic	TMCC1	129604478	A	G	0.00898562	0	0.0115	0	0.0308	0.0061
4	130582643	rs11731711	-0.788	0.00127	-0.8545	0.001019	-0.53	0.001808	-0.8667	3.51e-05	intergenic	INC02479;SNHG	131503798	A	T	0.16873	0.0317	0.2032	0.0507	0.4165	0.1902
4	130582661	rs11723497	-0.788	0.00127	-0.8545	0.001019	-0.53	0.001808	-0.8667	3.51e-05	intergenic	INC02479;SNHG	131503816	C	T	0.16873	0.0317	0.2032	0.0507	0.4165	0.1902
4	130583050	rs62317038	-0.788	0.00127	-0.8545	0.001019	-0.53	0.001808	-0.8667	3.51e-05	intergenic	INC02479;SNHG	131504205	C	T	0.16873	0.0317	0.2032	0.0507	0.4165	0.1902
4	130583369	rs55680610	-0.788	0.00127	-0.8545	0.001019	-0.53	0.001808	-0.8667	3.51e-05	intergenic	INC02479;SNHG	131504524	T	C	0.169129	0.0317	0.2032	0.0514	0.4175	0.1902
4	130583697	rs34454626	-0.788	0.00127	-0.8545	0.001019	-0.53	0.001808	-0.8667	3.51e-05	intergenic	INC02479;SNHG	131504852	C	T	0.000998403	0	0	0.0038	0	0
4	130583697	rs34454626	-0.788	0.00127	-0.8545	0.001019	-0.53	0.001808	-0.8667	3.51e-05	intergenic	INC02479;SNHG	131504852	C	CCTAA	0.168131	0.0317	0.2017	0.0514	0.4145	0.1892
4	130584586	rs11730029	-0.788	0.00127	-0.8545	0.001019	-0.53	0.001808	-0.8667	3.51e-05	intergenic	INC02479;SNHG	131505741	A	G	0.169728	0.0317	0.2032	0.0537	0.4175	0.1902
18	10509632	rs485686	0.644	0.001272	0.6359	0.00238	0.6628	3.96e-06	0.7417	1.13e-05	intergenic	APCDD1;NAPG	10509629	A	G	0.571486	0.6687	0.5115	0.7186	0.4384	0.4519
18	10510718	rs610092	0.644	0.001272	0.6359	0.00238	0.6628	3.96e-06	0.7417	1.13e-05	intergenic	APCDD1;NAPG	10510715	T	A	0.570088	0.6677	0.5115	0.7141	0.4384	0.4519
20	30855996	rs1235004438	0.9126	0.001274	1.006	0.0006253	0.831	9.78e-05	0.9511	7.11e-05	intergenic	FRG1BP;DEFB11	NA	NA	NA	NA	NA	NA	NA	NA	NA
17	43897400	rs140405783	0.9555	0.001275	1.048	0.0007312	0.2621	0.2652	0.4781	0.06639	intronic	MPP2	41974768	G	C	0.0567093	0	0.0605	0.003	0.1501	0.089
4	12743445	rs6448942	1.221	0.001278	1.34	0.0005199	0.5279	0.08052	0.9926	0.003059	intergenic	LINC02270;RAB2	12745069	C	T	0.0874601	0.0169	0.0562	0.2292	0.0239	0.0562
2	122613830	rs59115935	2.663	0.001279	2.805	0.0007842	0.5127	0.3976	1.635	0.01895	intergenic	TSN;LINC01826	123371406	C	CA	0.0509185	0.0417	0.0101	0.1551	0	0.001
5	170464780	rs75094236	2.086	0.00128	2.092	0.001619	1.063	0.01637	1.793	0.0005107	intronic	KCNIP1	169891784	C	T	0.0694888	0.0456	0.013	0.1785	0.0099	0.0481
18	10507967	rs80128778	0.6402	0.001283	0.6358	0.002286	0.6366	8.03e-06	0.7195	1.78e-05	intergenic	APCDD1;NAPG	10507964	T	C	0.569688	0.6687	0.5043	0.7186	0.4334	0.453
17	43893538	rs231504	0.8411	0.001287	0.8828	0.00142	0.2614	0.1933	0.3523	0.1229	intronic	MPP2	41970906	C	G	0.815296	1	0.8991	0.5477	0.837	0.9049
9	90225627	rs73492436	0.875	0.001288	0.8904	0.001715	0.795	5.34e-05	0.7859	0.0004954	intergenic	R4290HG;LINC01	NA	NA	NA	NA	NA	NA	NA	NA	NA
23	100735707	rs5920847	-0.6324	0.001288	-0.706	0.001008	-0.06494	0.6	-0.2388	0.1055	intergenic	SYTL4;CSTF2	NA	NA	NA	NA	NA	NA	NA	NA	NA

11	120492665	rs11217895	0.6565	0.001293	0.6693	0.001816	0.4757	0.001128	0.7076	4.09e-05	intergenic	ARHGEF12;GRIK4	120363374	C	T	0.477636	0.6458	0.5231	0.1831	0.7366	0.4039
12	98114376	rs74561960	2.104	0.001293	2.322	0.0004275	1.503	0.01299	1.885	0.003568	intergenic	MIR4303;SLC9A7F	98508154	A	G	0.0439297	0	0.0245	0.1407	0.0169	0
20	30881255	rs1447592663	1.044	0.001296	1.165	0.0004316	0.994	5.45e-05	1.152	2.37e-05	intergenic	FRG1BP;DEFB115	NA	NA	NA	NA	NA	NA	NA	NA	NA
20	30848757	rs1406603426	0.9531	0.001298	1.145	0.0004417	0.9333	5.14e-06	0.8779	0.0002136	intergenic	FRG1BP;DEFB115	NA	NA	NA	NA	NA	NA	NA	NA	NA
3	99544186	rs112163462	0.9607	0.001305	0.982	0.001803	0.5813	0.01828	0.8993	0.0007031	intergenic	DCBLD2;COL8A1	99263030	G	A	0.0465256	0.002	0.0807	0.0061	0.1143	0.0532
4	66774003	rs10018716	0.9176	0.001305	1.106	0.0002531	0.4504	0.03162	0.7057	0.003284	intergenic	1269A;LOC10192	67639721	G	C	0.78774	0.6071	0.8761	0.7065	0.9602	0.8436
20	29454736	rs1322381035	1.964	0.001305	2.181	0.0003991	1.271	0.01835	1.611	0.005433	ncRNA_intronic	LOC105379477	NA	NA	NA	NA	NA	NA	NA	NA	NA
3	130174897	rs113440653	1.996	0.001306	2.103	0.0007538	1.482	0.005138	1.948	0.0003785	intergenic	NC02021;COL6A4	NA	NA	NA	NA	NA	NA	NA	NA	NA
20	24670113	rs6036868	0.8268	0.001307	1.016	0.0001261	0.3205	0.1081	0.4421	0.04815	intergenic	SYNDIG1;CST7	24650749	G	C	0.842252	0.7698	0.9063	0.8298	0.9165	0.8119
15	23544157	rs6576439	0.6747	0.001309	0.6979	0.00146	0.5772	0.0001797	0.6983	8.58e-05	intergenic	OLGA6L2;MIR45(	23789304	C	T	0.710264	0.8026	0.7695	0.674	0.6123	0.7229
5	170459846	rs138003756	2.349	0.001313	2.389	0.001622	1.031	0.02344	1.933	0.0004383	intronic	KCNIP1	169886850	C	T	0.0617013	0.0456	0.0115	0.1467	0.0099	0.0521
1	201462483	rs55958748	1.803	0.001314	1.95	0.0004785	0.8796	0.07477	1.112	0.03435	intergenic	TNNI1;PHLDA3	201431611	C	T	0.00399361	0	0.0043	0.0023	0.0129	0.001
1	4761046	rs117666364	1.628	0.001322	1.776	0.0004904	0.5997	0.1897	1.074	0.02675	intronic	AJAP1	4821106	G	A	0.038139	0.0228	0.0072	0.0008	0.0179	0.1472
2	188223107	rs1439910	1.145	0.001328	1.264	0.0005887	0.7529	0.003225	1.227	2.78e-05	ncRNA_intronic	LINC01090	189087834	T	C	0.809704	0.7827	0.9539	0.6755	0.9503	0.772
2	188223745	rs10931341	1.145	0.001328	1.264	0.0005887	0.7529	0.003225	1.227	2.78e-05	ncRNA_intronic	LINC01090	189088472	C	T	0.809904	0.7827	0.9539	0.6762	0.9503	0.772
2	188224998	rs55944600	1.145	0.001328	1.264	0.0005887	0.7893	0.002051	1.292	1.17e-05	ncRNA_intronic	LINC01090	189089725	TGTAA	T	0.809704	0.7827	0.9539	0.6755	0.9503	0.772
2	188225738	rs980065	1.145	0.001328	1.264	0.0005887	0.7893	0.002051	1.292	1.17e-05	ncRNA_intronic	LINC01090	189090465	C	T	0.809904	0.7827	0.9539	0.6762	0.9503	0.772
2	188226786	rs13416775	1.145	0.001328	1.264	0.0005887	0.7893	0.002051	1.292	1.17e-05	ncRNA_intronic	LINC01090	189091513	C	T	0.189696	0.2173	0.0461	0.3238	0.0497	0.226
2	188227622	rs10221554	1.145	0.001328	1.264	0.0005887	0.7893	0.002051	1.292	1.17e-05	ncRNA_intronic	LINC01090	189092349	T	C	0.189497	0.2173	0.0461	0.3222	0.0507	0.226
2	188230414	rs4595930	1.145	0.001328	1.264	0.0005887	0.7893	0.002051	1.292	1.17e-05	ncRNA_intronic	LINC01090	189095141	G	A	0.189497	0.2173	0.0461	0.323	0.0497	0.226
2	188232918	rs66725342	1.145	0.001328	1.264	0.0005887	0.7893	0.002051	1.292	1.17e-05	ncRNA_intronic	LINC01090	189097645	A	G	0.189497	0.2173	0.0461	0.323	0.0497	0.226
2	188233401	rs66579041	1.145	0.001328	1.264	0.0005887	0.7893	0.002051	1.292	1.17e-05	ncRNA_intronic	LINC01090	189098128	A	G	0.189497	0.2173	0.0461	0.323	0.0497	0.226
2	188235498	rs6749586	1.145	0.001328	1.264	0.0005887	0.7893	0.002051	1.292	1.17e-05	ncRNA_intronic	LINC01090	189100225	C	T	0.189497	0.2173	0.0461	0.323	0.0497	0.226
2	188235773	rs6707721	1.145	0.001328	1.264	0.0005887	0.7893	0.002051	1.292	1.17e-05	ncRNA_intronic	LINC01090	189100500	T	C	0.189497	0.2173	0.0461	0.323	0.0497	0.226
2	188238504	rs13403138	1.145	0.001328	1.264	0.0005887	0.8007	0.001785	1.292	1.17e-05	ncRNA_intronic	LINC01090	189103231	T	C	0.189696	0.2173	0.0461	0.3238	0.0497	0.226
2	188241009	rs4337443	1.145	0.001328	1.264	0.0005887	0.7893	0.002051	1.292	1.17e-05	ncRNA_intronic	LINC01090	189105736	T	C	0.189696	0.2183	0.0461	0.323	0.0497	0.226
2	188244568	rs68056256	1.145	0.001328	1.264	0.0005887	0.7893	0.002051	1.292	1.17e-05	ncRNA_intronic	LINC01090	189109295	A	G	0.189497	0.2173	0.0461	0.323	0.0497	0.226
1	241937945	rs10926602	-0.6929	0.001329	-0.5764	0.009008	-0.5135	0.0006307	-0.6903	0.0001104	intergenic	EXO1;BECN2	242101247	T	G	0.378395	0.3819	0.3458	0.2564	0.4364	0.5031
9	100747435	rs370001347	1.235	0.001339	1.271	0.001305	0.5115	0.1312	0.8542	0.01718	intergenic	CAVIN4;PLPPR1	NA	NA	NA	NA	NA	NA	NA	NA	NA
8	113971059	rs7843577	0.628	0.001341	0.5615	0.006503	0.1013	0.4725	0.2938	0.06717	intergenic	CSMD3;TRPS1	114983288	C	T	0.340855	0.1825	0.2983	0.4448	0.4185	0.3139
2	176541498	rs11900388	0.6447	0.001342	0.6077	0.003653	0.4421	0.001773	0.6677	7.35e-05	intergenic	MTX2;MIR1246	177406226	A	T	0.484026	0.2014	0.415	0.6241	0.5775	0.5389
6	95924447	rs2799638	0.6817	0.001342	0.7362	0.0009126	0.5519	0.0001773	0.6802	8.44e-05	intergenic	MANEA;FUT9	96372323	G	A	0.359625	0.5843	0.1744	0.2655	0.3549	0.3916
17	43865334	rs231538	0.8355	0.001342	1.022	0.0001772	0.3187	0.1232	0.4958	0.02985	intergenic	CD300LG;MPP2	41942702	A	G	0.901358	1	0.902	0.8986	0.8111	0.8957
5	109170246	rs73779050	0.8899	0.001346	0.9368	0.001007	0.7658	0.0002471	1.007	1.73e-05	intronic	FER	108505947	C	A	0.139377	0.0694	0.111	0.1581	0.2107	0.1329
1	231879744	rs11122356	0.6274	0.001349	0.5933	0.00339	0.2244	0.09937	0.5171	0.0012	ncRNA_intronic	TSNAX-DISC1	232015490	G	C	0.400559	0.5397	0.4193	0.2791	0.3946	0.4141
6	52259746	rs12199650	-1.587	0.001352	-1.648	0.002344	-1.229	3.42e-05	-1.172	0.0008429	intergenic	IL17F;MCM3	52124544	T	C	0.0900559	0.0208	0.0476	0.18	0.0676	0.093
15	71567537	rs28517754	0.6549	0.001352	0.5782	0.007506	0.4082	0.004594	0.6684	9.32e-05	intronic	THSD4	71859876	C	T	0.447684	0.7054	0.4553	0.2791	0.2853	0.5716
1	4759820	rs3753722	1.624	0.001358	1.772	0.0005047	0.5966	0.1921	1.071	0.02721	intronic	AJAP1	4819880	C	T	0.0371406	0.0179	0.0072	0.0008	0.0179	0.1472
23	100830459	rs4827880	0.6136	0.00136	0.6453	0.001689	0.03426	0.7801	0.1779	0.2196	intronic	CSTF2	NA	NA	NA	NA	NA	NA	NA	NA	NA
15	17060130	rs1222431693	-0.9382	0.001361	-0.9173	0.002995	-0.9104	7.31e-06	-0.8613	0.0002671	intergenic	NONE;NONE	NA	NA	NA	NA	NA	NA	NA	NA	NA
17	43852739	rs4792921	0.807	0.001361	0.9887	0.0002012	0.3318	0.08079	0.4139	0.05828	intronic	CD300LG	41930107	T	G	0.230631	0.001	0.1383	0.5726	0.1968	0.1053
8	103119262	rs2515177	0.6124	0.001367	0.7715	0.0001985	0.2625	0.05946	0.3908	0.01548	intergenic	'P6V1C1;LINC011	104131490	G	A	0.443091	0.4296	0.4424	0.3843	0.4493	0.5307
17	43875844	rs231524	0.8384	0.001369	0.8799	0.001517	0.2512	0.2125	0.3461	0.1305	UTR3	MPP2	41953212	G	A	0.815695	1	0.8991	0.5484	0.837	0.9059
5	14876460	rs835154	-0.6355	0.001375	-0.6177	0.003312	-0.5699	8.55e-05	-0.6212	0.0002054	intergenic	ANKH;LINC0214(	14876569	G	A	0.701677	0.7063	0.5865	0.9244	0.5586	0.6247
4	66771463	rs10001624	0.9092	0.001388	1.112	0.0002322	0.4365	0.03636	0.6737	0.004621	intergenic	1269A;LOC10192	67637181	A	G	0.788139	0.6091	0.8761	0.7065	0.9602	0.8436
10	38746724	rs202050976	0.9365	0.001388	0.9562	0.001865	0.9016	6.74e-05	0.8452	0.0008178	intergenic	ACTR3BP5;NONE	NA	NA	NA	NA	NA	NA	NA	NA	NA
18	10504271	rs628896	0.6333	0.001388	0.6263	0.002521	0.6194	1.48e-05	0.7006	2.91e-05	intergenic	APCDD1;NAPG	10504268	C	T	0.56889	0.6677	0.5014	0.7186	0.4334	0.4519
4	144000137	rs80219495	-1.163	0.00139	-1.181	0.002567	-0.9596	4.29e-05	-0.8346	0.001822	intronic	GYPB	NA	NA	NA	NA	NA	NA	NA	NA	NA
18	10500116	rs527948	0.6336	0.001392	0.6269	0.002522	0.6299	1.04e-05	0.7152	1.94e-05	intergenic	APCDD1;NAPG	10500113	G	A	0.568091	0.6677	0.5029	0.7148	0.4334	0.4519
18	10500864	rs34238474	0.6336	0.001392	0.6269	0.002522	0.6299	1.04e-05	0.7152	1.94e-05	intergenic	APCDD1;NAPG	10500861	GT	G	0.569089	0.6677	0.5029	0.7186	0.4334	0.4519
18	10502161	rs622870	0.6336	0.001392	0.6269	0.002522	0.6322	9.11e-06	0.7106	2.19e-05	intergenic	APCDD1;NAPG	10502158	A	G	0.569688	0.6677	0.5029	0.7209	0.4334	0.4519
18	10504903	rs642133	0.6336	0.001392	0.6269	0.002522	0.6316	9.88e-06	0.7197	1.78e-05	intergenic	APCDD1;NAPG	10504900	G	A	0.567692	0.6677	0.5029	0.7141	0.4334	0.4509
18	10506082	rs525137	0.6336	0.001392	0.6269	0.002522	0.6352	8.34e-06	0.7152	1.94e-05	intergenic	APCDD1;NAPG	10506079	C	T	0.567093	0.6627	0.5	0.7171	0.4314	0.453
18	10506816	rs669434	0.6336	0.001392	0.6269	0.002522	0.6299	1.04e-05	0.7152	1.94e-05	intergenic	APCDD1;NAPG	10506813	A	G	0.569888	0.6687	0.5029	0.7209	0.4334	0.4519
18	10507082	rs13381734	0.6336	0.001392	0.6269	0.002522	0.6414	7.58e-06	0.7354	1.28e-05	intergenic	APCDD1;NAPG	10507079	C	G	0.569289	0.6687	0.5029	0.7186	0.4334	0.4519
7	81658659	rs117774075	1.718	0.001394	1.881	0.0004656	1.008	0.03422	1.328	0.007586	ncRNA_intronic	LOC100128317	81287975	G	T	0.00958466	0	0.0173	0.0023	0.0318	0.001
12	98118757	rs75431452	2.086	0.001399	2.322	0.0004275	1.496	0.01338	1.879	0.003687	intergenic	MIR4303;SLC9A7F	98512535	C	T	0.0441294	0	0.0259	0.1415	0.0159	0
2	188243275	rs4541223	1.134	0.001405	1.28	0.0004947	0.8154	0.001519	1.323	7.87e-06	ncRNA_intronic	LINC01090	189108002	C	T	0.189297	0.2173	0.0461	0.3222	0.0497	0.226
14	27432896	rs12898080	-0.6867	0.00141	-0.7808	0.0007245	-0.2167	0.1238	-0.2373	0.1437	ncRNA_intronic	LOC728755	27902102	A	T	0.486022	0.3889	0.6311	0.3079	0.6352	0.5706
2	215411319	rs13021377	0.7275	0.001411	0.6797	0.004488	0.7125	2.39e-05	0.7324	0.0001685	intronic	FN1	216276042	G	A	0.732228	0.999	0.7795	0.5129	0.6521	0.8027
18	10507345	rs492820	0.6322	0.001412	0.625	0.002569	0.6349	8.58e-06	0.7174	1.89e-05	intergenic	APCDD1;NAPG	10507342	C	T	0.567292	0.6677	0.4971	0.7186	0.4324	0.4479
22	16415043	rs79299078	-0.8807	0.001412	-1.096	0.0001542	-0.595	0.003929	-0.7466	0.001362	intergenic	DUXAP8;CCT8L2	NA	NA	NA	NA	NA	NA	NA	NA	NA
4	66769751	rs1375885	0.8959	0.001416	1.093	0.000246	0.4954	0.01578	0.7252	0.002018	intergenic	1269A;LOC10192	67635469	A	G	0.717053	0.6101	0.8487	0.447	0.9612	0.8476
2	161046013	rs4664033	0.7134	0.001422	0.7235	0.002182	0.7167	3.34e-05	0.7161	0.0002725	intergenic	RBMS1;TANK	161902524	G	C	0.20008	0.2252	0.1412	0.2534	0.2028	0.1411
1	75388518	rs12127575	-0.6884	0.001426	-0.8581	0.0001881	-0.3463	0.02617	-0.6514	0.0003094	intronic	SLC44A5	75854203	T	C	0.623602	0.9563	0.3876	0.7496	0.4404	0.4663
16	6298295	rs4441272	1.083	0.001426	1.102	0.001561	0.3312	0.2196	0.5337	0.07997	intronic	RBFOX1	6348296	C	A	0.111621	0.126	0.1037	0.1498	0.0219	0.1431
2	181495562	rs77229452	3	0.001433	3.224	0.0008001	1.313	0.1226	2.043	0.01795	intronic	ITGA4	182360289	T	C	0.0125799	0.0615	0	0	0	0.001
2	181546606	rs117436143	3	0.001433	3.224	0.0008001	1.313	0.1226	2.043	0.01795	intronic	CERKL	182411333	C	T	0.0119808	0.0585	0	0	0	0.001
17	43852506	rs8081396	0.802	0.001438	0.9814	0.0002186	0.3183	0.09219	0.4063	0.06222	intronic	CD300LG	41929874	A	G	0.230431	0.001	0.1383	0.5719	0.1968	0.1053
6	17486893	rs58937589	1.515	0.001439	1.56	0.001877	0.4226	0.2277	0.7052	0.1017	intronic	CAP2	NA	NA	NA	NA	NA	NA	NA	NA	NA
18	10507351	rs671624	0.6303	0.00144	0.6233	0.002604	0.6355	8.53e-06	0.7165	1.9e-05	intergenic	APCDD1;NAPG	10507348	T	C	0.567492	0.6677	0.4971	0.7186	0.4334	0.4479
8	102723851	rs62523332	-1.07	0.001444	-1.059	0.002822	-1.057	8.09e-06	-0.9345	0.0004706	intergenic	C101927245;GAS	103736079	G	A	0.105032	0.0982	0.2723	0.0809	0.0924	0.0389
2	108994641	rs6750059	0.7575	0.001445	0.8321	0.0009106	0.3909	0.02698	0.7448	0.0002243	intergenic	EDAR;SH3RF3-AS:	109611097	C	T	0.762979	0.871	0.8228	0.5946	0.7485	0.8517
2	188241543	rs7601023	1.137	0.001449	1.258	0.0006295	0.7831	0.002222	1.286	1.3e-05	ncRNA_intronic	LINC01090	189106270	G	A	0.189497	0.2173	0.0461	0.323	0.0497	0.226
2	188241585	rs9653312	1.137	0.001449	1.258	0.0006295	0.7831	0.002222	1.286	1.3e-05	ncRNA_intronic	LINC01090	189106312	T	G	0.189497	0.2173	0.0461	0.323	0.0497	0.226
8	103119231	rs2454035	0.6096	0.00145	0.7683	0.0002127	0.2626	0.05901	0.3873	0.01648	intergenic	'P6V1C1;LINC011	104131459	C	T	0.442891	0.4296	0.4438	0.3828	0.4493	0.5307
17	21320546	rs73985635	-0.9997	0.001452	-1.048	0.001333	-0.8752	0.0003051	-1.09	5.26e-05	intergenic	MAP2K3;KCNJ12	NA	NA	NA	NA	NA	NA	NA	NA	NA
2	188245955	rs6708455	1.13	0.001461	1.275	0.0005169	0.7964	0.001944	1.284	1.3e-05	ncRNA_intronic	LINC01090	189110682	T	C	0.189297	0.2173	0.0461	0.323	0.0497	0.2249
2	11077349	rs2005507	0.6789	0.001469	0.6437	0.003431	0.4616	0.002255	0.7466	2.11e-05	intergenic	KCNF1;FLJ33534	11217475	A	T	0.29972	0.377	0.3429	0.0408	0.3887	0.4479
2	161121762	rs10181831	0.7461	0.001474	0.7385	0.002985	0.7017	8.33e-05	0.6715	0.001049	intergenic	RBMS1;TANK	161978273	T	C	0.180911	0.2153	0.1297	0.2352	0.1789	0.1104
6	52258422	rs10456155	-1.575	0.00148	-1.634	0.002549	-1.199	5.14e-05	-1.157	0.0009772	intergenic	IL17F;MCM3	52123220	C	T	0.0806709	0.0208	0.0447	0.1475	0.0666	0.092
2	113511843	rs112334306	-1.155	0.001482	-1.121	0.00294	-0.9921	1.61e-05	-1.2	3.53e-05	intergenic	XD4L1;PGM5P3-/	NA	NA	NA	NA	NA	NA	NA	NA	NA
13	101584212	rs17685968	-0.7075	0.001489	-0.669	0.004174	-0.3967	0.007254	-0.7466	6.93e-05	intronic	ITGBL1	102236562	G	A	0.303714	0.4454	0.3285	0.1195	0.3042	0.3885
1	108369145	rs79171355	-1.129	0.001493	-1.025	0.005212	-0.972	5.01e-05	-0.9069	0.0011	intergenic	NBPF4;NBPF6	NA	NA	NA	NA	NA	NA	NA	NA	NA
9	20309135	rs76649715	1.057	0.001493	0.999	0.003305	0.3057	0.2852	0.4234	0.1743	intergenic	SLC24A2;MLLT3	20309133	G	A	0.0399361	0.002	0.072	0.0189	0.0686	0.0552
9	20312802	rs112317016	1.18	0.001498	1.165	0.002064	0.3171	0.3275	0.5921	0.08907	intergenic	SLC24A2;MLLT3	20312800	C	G	0.0265575	0.001	0.072	0.003	0.0517	0.0266
13	101575144	rs9557704	-0.7071	0.001498	-0.6714	0.004102	-0.372	0.01222	-0.7562	5.91e-05	intronic	ITGBL1	102227495	A	T	0.286941	0.4524	0.3156	0.0605	0.3002	0.3885
17	43873442	rs53907	0.835	0.001522	0.8802	0.001626	0.2074	0.3051	0.3463	0.1328	intergenic	CD300LG;MPP2	41950810	C	A	0.822684	1	0.8948	0.5855	0.829	0.9029
4	130582492	rs36065827	-0.7682	0.001535	-0.8545	0.001019	-0.5237	0.002015	-0.8553	4.14e-05	intergenic	INC02479;SNHG2	131503647	T	TG	0.16873	0.0317	0.2032	0.0507	0.4165	0.1902
4	130583103	rs62317039	-0.7682	0.001535	-0.8545	0.001019	-0.5237	0.002015	-0.8553	4.14e-05	intergenic	INC02479;SNHG2	131504258	G	A	0.16873	0.0317	0.2032	0.0507	0.4165	0.1902
4	130583331	rs55664222	-0.7682	0.001535	-0.8545	0.001019	-0.5237	0.002015	-0.8553	4.14e-05	intergenic	INC02479;SNHG2	131504486	T	C	0.16893	0.0317	0.2032	0.0507	0.4175	0.1902
4	12747681	rs7674264	1.183	0.001537	1.287	0.0007145	0.4797	0.1033	0.9429	0.004018	intergenic	LINC02270;RAB28	12749305	T	A	0.0874601	0.0169	0.0548	0.23	0.0229	0.0573
4	144000131	rs77152472	-1.144	0.001541	-1.085	0.003985	-0.9126	7.25e-05	-0.8363	0.001689	intronic	GYPB	NA	NA	NA	NA	NA	NA	NA	NA	NA
18	10499646	rs581369	0.6288	0.001551	0.623	0.002757	0.6253	1.23e-05	0.7091	2.36e-05	intergenic	APCDD1;NAPG	10499643	A	T	0.567891	0.6677	0.5014	0.7148	0.4334	0.4519
16	78424567	rs79064575	3.585	0.001553	3.691	0.001002	2.797	0.008525	3.102	0.004334	intronic	WWOX	78458464	A	T	0.0297524	0	0.0043	0.0749	0.0099	0.0378
8	76785553	rs28577115	0.9181	0.001556	1.062	0.0003709	0.5636	0.01382	0.8094	0.001308	intronic	ZFHX4	77697789	G	T	0.133387	0.1696	0.1441	0.1641	0.0915	0.09
8	76785554	rs28693494	0.9181	0.001556	1.062	0.0003709	0.5636	0.01382	0.8094	0.001308	intronic	ZFHX4	77697790	C	A	0.133387	0.1706	0.1441	0.1634	0.0915	0.09
18	10505899	rs523761	0.6306	0.001556	0.6126	0.003211	0.6374	9e-06	0.7159	2.19e-05	intergenic	APCDD1;NAPG	10505896	T	C	0.57528	0.6409	0.4971	0.7723	0.4314	0.4448
5	24870003	rs115086836	1.82	0.001557	1.976	0.0005897	0.6806	0.2152	1.254	0.02344	intergenic	NC02239;LINC022	24870112	A	G	0.00539137	0	0.0058	0.0008	0.0189	0.0031

17	43887148	rs231514	0.8861	0.001562	0.9772	0.0007989	0.1598	0.4714	0.3961	0.1072	intronic	MPP2	41964516	C	T	0.920327	1	0.928	0.9251	0.8439	0.9049
17	43888463	rs231512	0.8861	0.001562	0.9772	0.0007989	0.1598	0.4714	0.3961	0.1072	intronic	MPP2	41965831	G	A	0.920128	1	0.928	0.9251	0.8429	0.9049
20	3392922	rs74338704	1.854	0.001565	1.92	0.001203	1.626	0.002096	1.986	0.0002313	intronic	C20orf194	3373569	T	C	0.013778	0	0.0259	0.0008	0.0467	0.0031
10	59354233	rs284609	0.6724	0.001566	0.7529	0.0007375	0.1826	0.2478	0.3804	0.033	intronic	FAM13C	61113993	G	A	0.348442	0.1776	0.451	0.5303	0.2336	0.3241
4	130582385	rs62317037	-0.7658	0.001569	-0.8521	0.00104	-0.5224	0.002061	-0.8534	4.26e-05	intergenic	INC02479;SNHG2	131503540	T	C	0.16873	0.0317	0.2032	0.0507	0.4165	0.1902
7	81654997	rs75306112	1.694	0.00157	1.84	0.0005726	1	0.03553	1.315	0.008099	ncRNA_intronic	LOC100128317	81284313	C	A	0.0185703	0	0.0187	0.0356	0.0318	0.001
7	81660858	rs74766322	1.694	0.00157	1.84	0.0005726	1	0.03553	1.315	0.008099	ncRNA_intronic	LOC100128317	81290174	G	A	0.0185703	0	0.0187	0.0356	0.0318	0.001
8	72024193	rs76583847	0.8448	0.001571	1.036	0.0001847	0.463	0.0276	0.5424	0.01918	ncRNA_intronic	MSC-AS1	72936428	T	C	0.151158	0.1597	0.2867	0.0605	0.0775	0.2444
18	10506974	rs500342	0.6232	0.001575	0.6164	0.002838	0.6301	9.4e-06	0.7048	2.3e-05	intergenic	APCDD1;NAPG	10506971	C	T	0.568291	0.6687	0.5029	0.7148	0.4334	0.4519
2	188236229	rs73039929	1.128	0.001582	1.247	0.0007069	0.751	0.003091	1.27	1.54e-05	ncRNA_intronic	LINC01090	189100956	T	C	0.195687	0.2173	0.0476	0.3457	0.0497	0.226
2	188238417	rs13389609	1.128	0.001582	1.247	0.0007069	0.751	0.003091	1.27	1.54e-05	ncRNA_intronic	LINC01090	189103144	C	A	0.195887	0.2173	0.0476	0.3464	0.0497	0.226
9	38998221	rs202009022	-1.457	0.001584	-1.488	0.002841	-1.049	9.55e-05	-1.058	0.001081	intergenic	AM201A;CNTNAP	NA	NA	NA	NA	NA	NA	NA	NA	NA
1	66802655	rs4433364	0.8526	0.001592	0.9158	0.001291	0.8729	4.48e-05	0.9405	6.87e-05	intergenic	INSL5;DNAI4	67268338	A	G	0.864217	0.9901	0.889	0.7564	0.83	0.8978
1	66808318	rs1498347	0.8526	0.001592	0.9158	0.001291	0.8729	4.48e-05	0.9405	6.87e-05	intergenic	INSL5;DNAI4	67274001	C	T	0.864018	0.9901	0.889	0.7557	0.83	0.8978
4	60036626	rs144545480	3.948	0.001592	4.154	0.0008723	2.258	0.03946	2.522	0.02668	intergenic	C02429;MIR548A	60902344	A	G	0.00938498	0.0456	0	0	0	0.001
4	60067612	rs149131530	3.948	0.001592	4.154	0.0008723	2.258	0.03946	2.522	0.02668	intergenic	C02429;MIR548A	60933330	G	T	0.00978435	0.0466	0	0	0	0.002
20	29403966	rs1317690206	-0.9291	0.001593	-0.8415	0.006545	-0.8658	8.59e-05	-0.8567	0.0006427	intergenic	FRG1DP;FRG2EP	NA	NA	NA	NA	NA	NA	NA	NA	NA
20	3299200	rs117905149	1.85	0.001598	1.916	0.001225	1.62	0.002159	1.979	0.0002419	intronic	C20orf194	3279847	A	C	0.0133786	0	0.0259	0.0008	0.0447	0.0031
15	63349162	rs35763222	0.6969	0.001601	0.7482	0.00118	0.1746	0.3086	0.2422	0.2104	intronic	CA12	63641361	C	G	0.20028	0.0149	0.3127	0.2617	0.2346	0.1933
4	12750723	rs201531146	1.181	0.001603	1.289	0.0007171	0.4734	0.1088	0.9387	0.004236	intergenic	LINC02270;RAB28	12752347	G	T	0.0866613	0.0169	0.0548	0.2269	0.0229	0.0573
7	84014922	rs74421748	1.303	0.001607	1.289	0.002466	0.539	0.1395	0.6878	0.08042	intronic	SEMA3A	83644238	A	T	0.0888578	0.0367	0.0403	0.1989	0.0318	0.0869
8	113968778	rs988735	0.6192	0.001609	0.5527	0.007551	0.07974	0.573	0.2846	0.07674	intergenic	CSMD3;TRPS1	114981007	G	T	0.331669	0.1845	0.2925	0.4123	0.4175	0.3139
8	77935476	rs28658457	-1.433	0.00161	-1.505	0.002157	-0.423	0.06061	-1.244	0.0002406	intergenic	OC102724874;PK	78847711	G	T	0.0848642	0.0446	0.0692	0.0204	0.1531	0.1544
12	98130077	rs117011044	2.553	0.001615	2.767	0.0006514	1.668	0.03461	2.003	0.01227	intergenic	MIR4303;SLC9A7P	98523855	T	A	0.00379393	0	0.0101	0	0.0119	0
6	31341778	rs6924925	-0.6316	0.001617	-0.5992	0.004564	-0.5538	8.3e-05	-0.5617	0.0008093	intergenic	LINC02571;HLA-B	31309555	G	T	0.545927	0.4157	0.4899	0.6884	0.5378	0.5358
2	188224223	rs12614592	1.12	0.001635	1.264	0.0005887	0.7819	0.002239	1.28	1.37e-05	ncRNA_intronic	LINC01090	189088950	C	T	0.809704	0.7827	0.9539	0.6755	0.9503	0.772
2	188226941	rs13432874	1.12	0.001635	1.264	0.0005887	0.7819	0.002239	1.28	1.37e-05	ncRNA_intronic	LINC01090	189091668	T	C	0.189696	0.2173	0.0461	0.3238	0.0497	0.226
2	188232129	rs7586536	1.12	0.001635	1.264	0.0005887	0.7819	0.002239	1.28	1.37e-05	ncRNA_intronic	LINC01090	189096856	C	A	0.189497	0.2173	0.0461	0.323	0.0497	0.226
2	188236835	rs7596399	1.12	0.001635	1.264	0.0005887	0.7819	0.002239	1.28	1.37e-05	ncRNA_intronic	LINC01090	189101562	T	G	0.189497	0.2173	0.0461	0.323	0.0497	0.226
2	188238939	rs58838228	1.12	0.001635	1.264	0.0005887	0.7819	0.002239	1.28	1.37e-05	ncRNA_intronic	LINC01090	189103666	TA	T	0.189497	0.2173	0.0461	0.323	0.0497	0.226
20	756478	rs67385369	0.9454	0.001649	0.975	0.001583	0.7282	0.0003674	0.9499	7.38e-05	intergenic	SCRT2;SLC52A3	737122	T	C	0.204273	0.3681	0.0764	0.1422	0.1322	0.2843
3	36129117	rs13094705	0.8705	0.001652	0.9833	0.0006579	0.3163	0.1681	0.4842	0.05509	intergenic	ARPP21;STAC	36170609	G	A	0.04373	0	0.085	0.0061	0.1123	0.0399
20	30179092	rs1258112712	-0.864	0.001653	-0.8539	0.002996	-0.8831	1.11e-05	-0.878	0.000139	intergenic	IR4477A;LINC015	NA	NA	NA	NA	NA	NA	NA	NA	NA
20	30179096	rs1316807282	-0.864	0.001653	-0.8539	0.002996	-0.8831	1.11e-05	-0.878	0.000139	intergenic	IR4477A;LINC015	NA	NA	NA	NA	NA	NA	NA	NA	NA
2	161085203	rs13429707	0.7277	0.001658	0.7395	0.002605	0.6856	9.12e-05	0.6407	0.001465	intergenic	RBMS1;TANK	161941714	T	C	0.203674	0.2163	0.1441	0.3056	0.1889	0.1104
1	151150013	rs7554936	0.6278	0.00166	0.5729	0.006373	0.5902	8.9e-05	0.5083	0.002265	intergenic	A6C;TNFAIP8L2-S	151122489	C	T	0.560903	0.8562	0.6801	0.0303	0.666	0.7812
7	62421794	rs1189815716	0.8627	0.00166	0.9523	0.001128	0.7805	5.61e-05	0.9035	7.56e-05	intergenic	NONE;ZNF733P	NA	NA	NA	NA	NA	NA	NA	NA	NA
7	81654074	rs77119348	1.681	0.001662	1.84	0.0005726	0.9961	0.03618	1.308	0.008394	ncRNA_intronic	LOC100128317	81283390	T	C	0.0185703	0	0.0187	0.0356	0.0318	0.001
7	81662729	rs74731369	1.681	0.001662	1.84	0.0005726	0.9961	0.03618	1.308	0.008394	ncRNA_intronic	LOC100128317	81292045	A	G	0.0183706	0	0.0187	0.0356	0.0308	0.001
11	120499825	rs2000563	-0.6372	0.001667	-0.5625	0.00772	-0.4672	0.0007432	-0.6622	6.37e-05	intergenic	ARHGEF12;GRIK4	120370534	A	T	0.311302	0.4931	0.389	0.0673	0.4742	0.2311
12	97973805	rs75326067	2.593	0.001668	2.76	0.0007669	1.625	0.03939	2.077	0.01025	intergenic	MIR4495;MIR430	98367583	C	A	0.0123802	0	0.0259	0.0204	0.0169	0
2	188233778	rs72901581	1.117	0.00167	1.262	0.0006011	0.7786	0.002335	1.276	1.46e-05	ncRNA_intronic	LINC01090	189098505	C	T	0.189497	0.2173	0.0461	0.323	0.0497	0.226
2	188237460	rs67324207	1.117	0.00167	1.262	0.0006011	0.7786	0.002335	1.276	1.46e-05	ncRNA_intronic	LINC01090	189102187	C	T	0.189696	0.2173	0.0461	0.323	0.0497	0.227
6	5788112	rs59386661	0.9831	0.001673	1.003	0.001798	0.6028	0.01157	0.9869	0.000248	intergenic	FARS2;NRN1	5788345	C	G	0.151957	0.0139	0.1066	0.3487	0.0934	0.1207
18	10506252	rs658365	-0.6	0.001677	-0.5954	0.003056	-0.5732	2.56e-05	-0.6446	6.07e-05	intergenic	APCDD1;NAPG	10506249	T	C	0.568091	0.6687	0.5043	0.7126	0.4344	0.4519
15	49838658	rs138390292	0.7612	0.001696	0.8421	0.001138	0.5895	0.0007589	0.8093	6.56e-05	intergenic	DTWD1;ATP8B4	50130855	C	T	0.196086	0.4058	0.2075	0.0703	0.1531	0.1861
4	12754735	rs76419513	1.172	0.001697	1.287	0.0007145	0.4737	0.1076	0.9361	0.004297	intergenic	LINC02270;RAB28	12756359	T	G	0.0870607	0.0169	0.0548	0.2277	0.0239	0.0573
1	227480480	rs2819522	0.7433	0.001702	0.9186	0.0001831	0.4711	0.01153	0.6003	0.003883	intergenic	CDC42BPA;ZNF67	227668181	G	A	0.796725	0.9117	0.8732	0.6853	0.8131	0.7577
1	66793425	rs4655652	0.8427	0.001707	0.8876	0.001651	0.8347	7.14e-05	0.9009	0.0001121	intergenic	TCTEX1D1;INSL5	67259108	G	A	0.86222	0.9901	0.879	0.7648	0.8191	0.8947
16	36095681	rs1395572932	0.9021	0.00171	0.8367	0.005273	0.9431	2.65e-06	0.9631	4.52e-05	intergenic	LINC02167;NONE	NA	NA	NA	NA	NA	NA	NA	NA	NA
19	47973089	rs11878324	-0.6126	0.001713	-0.6718	0.001115	-0.4168	0.002465	-0.6354	9.78e-05	intronic	BSPH1	48476346	C	T	0.501597	0.3383	0.5735	0.4599	0.6282	0.545
12	98116998	rs146927925	2.538	0.001721	2.732	0.0007524	1.84	0.0191	2.035	0.01123	intergenic	MIR4303;SLC9A7P	98510776	CATAT	C	0.0107827	0	0.0216	0.0182	0.0149	0
4	12747923	rs7690933	1.172	0.001723	1.275	0.0008106	0.4689	0.1111	0.9304	0.004538	intergenic	LINC02270;RAB28	12749547	G	C	0.0864617	0.0169	0.0548	0.2262	0.0229	0.0573
6	64297637	rs6917544	-0.765	0.001723	-0.7244	0.004296	-0.6822	5.24e-05	-0.6262	0.001188	intronic	EYS	65007530	C	T	0.194489	0.1071	0.2003	0.0265	0.3221	0.3763
17	43868408	rs72836567	0.8371	0.001724	0.9947	0.0003413	0.3409	0.1079	0.4763	0.04154	intergenic	CD300LG;MPP2	41945776	G	A	0.0750799	0	0.0994	0.0113	0.1899	0.1033
12	98177858	rs79707681	2.193	0.00173	2.401	0.0006152	1.437	0.03143	1.63	0.01765	intergenic	MIR4303;SLC9A7P	98571636	A	T	0.0081869	0	0.013	0.0144	0.0129	0
15	23546643	rs11635011	0.6641	0.001731	0.694	0.001791	0.5626	0.0002955	0.7002	9.16e-05	intergenic	OLGA6L2;MIR45	23791790	A	G	0.245008	0.1964	0.2176	0.1929	0.3767	0.2495
4	94793003	rs59859017	2.062	0.001735	2.291	0.000675	0.4999	0.3737	1.156	0.04412	intronic	BMPR1B	95714154	A	ACTT	0.98722	0.9782	0.9856	0.997	0.9742	0.998
12	98122565	rs79603557	2.192	0.001737	2.373	0.0006933	1.443	0.03076	1.635	0.01739	intergenic	MIR4303;SLC9A7P	98516343	T	G	0.0101837	0	0.0187	0.0182	0.0139	0
12	98175758	rs75417287	2.192	0.001737	2.373	0.0006933	1.443	0.03076	1.635	0.01739	intergenic	MIR4303;SLC9A7P	98569536	C	T	0.0207668	0	0.0144	0.0613	0.0129	0
18	10499951	rs653239	0.6221	0.001743	0.616	0.003083	0.624	1.27e-05	0.7068	2.49e-05	intergenic	APCDD1;NAPG	10499948	T	G	0.569489	0.6677	0.5014	0.7209	0.4334	0.4519
22	22323593	rs796465348	-1.039	0.00175	-1.116	0.001773	-0.6091	0.004272	-1.068	9.37e-05	downstream	BMS1P20	NA	NA	NA	NA	NA	NA	NA	NA	NA
22	22323605	rs796650754	-1.039	0.00175	-1.116	0.001773	-0.6091	0.004272	-1.068	9.37e-05	downstream	BMS1P20	NA	NA	NA	NA	NA	NA	NA	NA	NA
18	7461883	rs74928586	1.4	0.001752	1.575	0.0004571	0.4765	0.247	0.8762	0.03774	upstream	LOC112577592	7461881	G	A	0.0131789	0	0.0274	0.0038	0.0368	0.0051
19	47966876	rs66860685	0.6133	0.001763	0.7632	0.0003083	0.4352	0.001876	0.6688	5.83e-05	intergenic	SNAR-C2;BSPH1	NA	NA	NA	NA	NA	NA	NA	NA	NA
17	43795128	rs149876847	1.293	0.001766	1.448	0.0005677	0.6322	0.07713	0.819	0.03154	intergenic	CFAP97D1;MPP3	41872496	T	A	0.0173722	0	0.0202	0.0023	0.0507	0.0194
17	43804973	rs117930744	1.293	0.001766	1.448	0.0005677	0.6337	0.07662	0.8168	0.0321	intronic	MPP3	41882341	G	A	0.0171725	0	0.0202	0.0023	0.0497	0.0194
17	43824715	rs78553806	1.293	0.001766	1.448	0.0005677	0.6322	0.07713	0.819	0.03154	intronic	MPP3	41902083	A	G	0.0173722	0	0.0173	0.0023	0.0517	0.0204
2	235399290	rs7583365	1.152	0.00177	1.42	0.0002178	0.4416	0.09571	0.7228	0.01588	intergenic	SH3BP4;AGAP1	236307934	C	T	0.136981	0.2639	0.0173	0.093	0.0398	0.2505
13	101573868	rs9557703	-0.6788	0.001772	-0.6368	0.005129	-0.3572	0.01287	-0.7356	6.65e-05	intronic	ITGBL1	102226219	C	T	0.332668	0.4673	0.3228	0.1899	0.3161	0.411
9	42086160	rs3905600	0.892	0.001778	1.14	0.0001878	0.6536	0.001512	0.8719	0.0002315	intronic	CNTNAP3B	NA	NA	NA	NA	NA	NA	NA	NA	NA
18	10496988	rs509416	0.6224	0.001781	0.6133	0.003245	0.6058	2.24e-05	0.6926	3.67e-05	intergenic	APCDD1;NAPG	10496985	A	G	0.564696	0.6468	0.5	0.7171	0.4334	0.455
18	10492751	rs577418	0.6191	0.001791	0.5768	0.004986	0.6402	7.39e-06	0.7248	1.56e-05	intergenic	APCDD1;NAPG	10492748	A	T	0.54393	0.6478	0.5	0.6384	0.4324	0.455
2	188234628	rs73039927	1.111	0.001793	1.258	0.0006319	0.775	0.002446	1.273	1.55e-05	ncRNA_intronic	LINC01090	189099355	A	C	0.189497	0.2173	0.0461	0.323	0.0497	0.226
2	188235155	rs79524138	1.111	0.001793	1.258	0.0006319	0.775	0.002446	1.273	1.55e-05	ncRNA_intronic	LINC01090	189099882	A	G	0.189497	0.2173	0.0461	0.323	0.0497	0.226
10	55870063	rs11005084	1.426	0.001798	1.586	0.0005304	0.7487	0.06242	1.185	0.004675	intergenic	PCDH15;ZWINT	57629823	G	A	0.0307508	0.0089	0.0231	0.0038	0.0318	0.0941
2	215408729	rs940740	0.7163	0.001805	0.7144	0.002764	0.7342	2.89e-05	0.6793	0.0005865	intronic	FN1	216273452	G	A	0.203075	0.001	0.1758	0.3396	0.3022	0.1442
16	34162001	rs112922093	1.168	0.001811	1.145	0.002897	1.018	0.0003847	1.295	4.03e-05	intergenic	NPP7P13;MIR99	NA	NA	NA	NA	NA	NA	NA	NA	NA
20	30174943	rs1353553916	0.937	0.001811	0.9912	0.001852	0.6355	0.001691	1.006	5.67e-05	intergenic	IR4477A;LINC015	NA	NA	NA	NA	NA	NA	NA	NA	NA
16	27431531	rs3091235	0.7078	0.001816	0.7871	0.0008679	0.2218	0.2191	0.3412	0.08788	intronic	IL21R	27442852	A	G	0.861422	0.9891	0.7637	0.8169	0.7793	0.9438
18	10500365	rs525254	0.6219	0.001816	0.6131	0.003354	0.6575	5.16e-06	0.7234	1.89e-05	intergenic	APCDD1;NAPG	10500362	C	T	0.569289	0.6677	0.5043	0.7186	0.4334	0.4519
16	46412675	rs796284954	0.9891	0.001827	1.14	0.0004238	0.5379	0.03193	0.8141	0.003146	intergenic	NONE;ANKRD26P	NA	NA	NA	NA	NA	NA	NA	NA	NA
5	118275215	rs6860479	0.7173	0.001828	0.8361	0.0004488	0.217	0.2205	0.4	0.04589	intergenic	NC02147;LINC021	117610910	A	G	0.201877	0.0248	0.1859	0.3601	0.2336	0.1493
17	22054172	rs73313358	-0.993	0.00183	-0.9412	0.004697	-0.915	2.54e-05	-0.903	0.0004435	intergenic	KCNJ18;UBBP4	NA	NA	NA	NA	NA	NA	NA	NA	NA
7	81663831	rs11771992	1.658	0.001838	1.823	0.0006045	1.006	0.03365	1.281	0.009719	ncRNA_intronic	LOC100128317	81293147	T	C	0.0207668	0	0.0187	0.0431	0.0328	0.001
20	28784580	rs1473358056	-0.852	0.001841	-0.9213	0.001497	-0.8689	8.01e-06	-0.9086	6.56e-05	intergenic	FRG1CP;FRG1DP	NA	NA	NA	NA	NA	NA	NA	NA	NA
6	129535763	rs9375634	-0.6498	0.001843	-0.7467	0.00072	-0.4802	0.0007991	-0.7434	2.47e-05	intergenic	LAMA2;ARHGAP1	129856908	A	G	0.374601	0.1756	0.5648	0.3495	0.494	0.3558
18	10513004	rs62093530	-0.6319	0.001844	-0.6392	0.002825	-0.647	8.49e-06	-0.7188	2.77e-05	intergenic	APCDD1;NAPG	10513001	A	G	0.565296	0.6726	0.4986	0.6997	0.4344	0.455
20	53603526	rs3916631	-0.9665	0.001845	-0.8499	0.007423	-0.8469	5.73e-05	-0.742	0.002392	intergenic	F217;LOC105372	NA	NA	NA	NA	NA	NA	NA	NA	NA
12	97925303	rs80027012	2.255	0.001847	2.429	0.0007631	1.247	0.06524	1.747	0.01328	intergenic	FAH1B2P2;MIR44	98319081	G	A	0.0139776	0	0.0245	0.0272	0.0169	0
2	113773243	rs76106163	-0.7347	0.001848	-0.6822	0.005197	-0.668	6.94e-05	-0.6858	0.0004145	intergenic	LC35F5;ACTR3-AS	114530820	G	A	0.226637	0.127	0.2176	0.1808	0.3767	0.2434
12	98181432	rs79821503	2.18	0.001852	2.36	0.0007415	1.435	0.03177	1.622	0.01823	intergenic	MIR4303;SLC9A7P	98575210	T	G	0.0207668	0	0.0144	0.0613	0.0129	0
16	27431972	rs8057464	0.7403	0.001855	0.7706	0.001779	0.2657	0.1582	0.3758	0.07259	intronic	IL21R	27443293	A	C	0.867212	0.9881	0.7579	0.8427	0.7803	0.9427
1	75384043	rs11485322	0.7289	0.001856	0.7832	0.001354	0.4777	0.007276	0.7314	0.0002789	intronic	SLC44A5	75849728	G	C	0.237021	0.1359	0.1427	0.3933	0.2237	0.2106
18	10504977	rs556347	0.6183	0.001856	0.6087	0.003441	0.6289	1.04e-05	0.7057	2.54e-05	intergenic	APCDD1;NAPG	10504974	T	G	0.570887	0.6677	0.5029	0.7254	0.4334	0.4519
8	102746801	rs867903	-1.081	0.001875	-0.9712	0.00643	-0.9915	3.22e-05	-0.8116	0.002193	intergenic	C101927245;GAS	103759029	G	T	0.0846645	0.1081	0.2594	0.0045	0.0905	0.0389
20	25928874	rs73350121	0.9633	0.001882	1.046	0.001162	0.9668	5.45e-05	0.9019	0.0008195	intergenic	101926935;LINC0	NA	NA	NA	NA	NA	NA	NA	NA	NA
17	43915282	rs11654245	0.8821	0.001889	0.9742	0.0009935	0.2254	0.3234	0.4322	0.0856	intergenic	MPP2;FAM215A	41992650	T	C	0.0589058	0.001	0.0576	0.0038	0.1511	0.0992
4	9718747	rs200623457	-0.9804	0.001902	-0.886	0.006476	-0.8281	9.12e-05	-0.8845	0.0004532	intergenic	MIR548I2;DRD5	NA	NA	NA	NA	NA	NA	NA	NA	NA

8	54042034	rs148768519	1.17	0.001905	1.176	0.002286	0.4275	0.1513	0.6297	0.06913	intergenic	TCEA1;LYPLA1	54954594	\AGCCTGTATTGG	A	0.135583	0.0119	0.1124	0.4206	0.0278	0.0051
9	64029381	rs1375091269	-1.693	0.00192	-1.528	0.005101	-1.539	5.62e-06	-1.612	0.0001375	ncRNA_intronic	FLJ43315	NA	NA	NA	NA	NA	NA	NA	NA	NA
14	86461526	rs8011240	-1.132	0.001927	-1.212	0.002383	-0.9317	5.92e-05	-1.161	9.06e-05	intergenic	\C02328;LINC011	86927870	G	A	0.796725	0.8403	0.8199	0.5719	0.9304	0.9018
7	43507005	rs7810051	0.6769	0.001932	0.6338	0.005472	0.6929	4.11e-05	0.4842	0.01222	intronic	HECW1	43546604	A	G	0.191693	0.0863	0.1888	0.1664	0.2276	0.2996
1	66806867	rs2221082	0.832	0.001942	0.8763	0.001886	0.8207	9.13e-05	0.8877	0.0001402	intergenic	INSL5;DNAI4	67272550	G	A	0.858427	0.9901	0.879	0.7496	0.8191	0.8957
6	129526659	rs2018906	-0.6408	0.001954	-0.7211	0.001015	-0.4591	0.001272	-0.7093	5.23e-05	intergenic	.AMA2;ARHGAP1	129847804	A	C	0.363818	0.1786	0.4251	0.3616	0.5149	0.3589
14	81714270	rs4613000	-0.7004	0.001957	-0.9442	0.0002084	-0.2461	0.1002	-0.3922	0.02555	intergenic	\C01467;LINC023	82180614	A	C	0.633586	0.3373	0.6585	0.7791	0.7247	0.6309
6	143780250	rs28546310	0.6044	0.001959	0.6686	0.001185	0.06262	0.663	0.1455	0.3802	intronic	PHACTR2	144101387	A	G	0.273962	0.3284	0.3156	0.1884	0.2863	0.2914
17	21655983	rs74943269	0.8525	0.001959	1.092	0.0002686	0.7202	0.0002012	0.9606	3.02e-05	intergenic	.INC02693;KCNJ1	NA	NA	NA	NA	NA	NA	NA	NA	NA
23	313646	rs188048111	1.495	0.001962	1.745	0.0004202	1.094	0.01097	1.187	0.009888	intronic	GTPBP6	NA	NA	NA	NA	NA	NA	NA	NA	NA
2	215410537	rs12991003	0.6973	0.001968	0.6666	0.004713	0.6934	3.43e-05	0.7037	0.0002552	intronic	FN1	216275260	T	C	0.733427	0.999	0.781	0.5182	0.6511	0.8016
18	10495126	rs568812	0.6138	0.001972	0.6091	0.003471	0.6048	2.26e-05	0.6897	3.85e-05	intergenic	APCDD1;NAPG	10495123	G	A	0.564097	0.6468	0.5	0.7156	0.4324	0.455
18	10498201	rs658288	0.6138	0.001972	0.6091	0.003471	0.6048	2.26e-05	0.6897	3.85e-05	intergenic	APCDD1;NAPG	10498198	G	A	0.564097	0.6468	0.5014	0.7141	0.4334	0.455
18	10493277	rs545857	-0.6144	0.001974	-0.5721	0.005433	-0.6264	1.24e-05	-0.7178	1.96e-05	intergenic	APCDD1;NAPG	10493274	G	C	0.53155	0.6478	0.4971	0.593	0.4324	0.455
16	34698564	rs1434984324	-1.077	0.001975	-0.9063	0.00981	-1.046	6.66e-06	-1.154	5.33e-05	intergenic	CCNYL3;CCNYL3	NA	NA	NA	NA	NA	NA	NA	NA	NA
23	100740828	rs5921637	0.603	0.001975	0.6552	0.001899	0.05932	0.6289	0.1973	0.1747	intergenic	SYTL4;CSTF2	NA	NA	NA	NA	NA	NA	NA	NA	NA
16	34698574	rs1192820473	-1.201	0.001976	-1.034	0.008075	-1.05	2.32e-05	-1.213	9.91e-05	intergenic	CCNYL3;CCNYL3	NA	NA	NA	NA	NA	NA	NA	NA	NA
12	98091992	rs2110755	0.6013	0.001979	0.7354	0.0003559	0.05527	0.6889	0.2148	0.1743	intergenic	/IIR4303;SLC9A7F	98485770	T	A	0.613419	0.7669	0.6628	0.4947	0.5	0.6973
14	27529849	rs2022900	-0.6509	0.00198	-0.7544	0.0008976	-0.2173	0.1194	-0.2412	0.1339	ncRNA_intronic	LOC728755	27999055	C	G	0.49381	0.6032	0.3501	0.6097	0.3648	0.4591
17	1167104	rs10902642	0.5937	0.001987	0.6066	0.002514	0.2407	0.0805	0.5678	0.0004753	intronic	ABR	1070398	C	T	0.275958	0.2014	0.3919	0.0484	0.4841	0.364
13	101590913	rs9557709	-0.6759	0.001991	-0.7125	0.002415	-0.3721	0.0109	-0.7208	9.72e-05	intronic	ITGBL1	102243263	G	T	0.311701	0.4365	0.3256	0.1611	0.3032	0.3855
17	1165869	rs60742899	0.5937	0.002	0.6059	0.002556	0.2467	0.07379	0.5668	0.0004884	intronic	ABR	1069163	G	A	0.27496	0.2143	0.3948	0.0295	0.4861	0.3671
6	36984450	rs3822972	0.7168	0.002005	0.7857	0.001065	0.1531	0.3701	0.2162	0.2688	intronic	MTCH1	36952226	G	A	0.193091	0.38	0.1499	0.1997	0.1581	0.0583
1	75376380	rs145173449	-0.6589	0.002008	-0.8211	0.0002945	-0.3605	0.01966	-0.6034	0.0006367	intronic	SLC44A5	75842065	G	A	0.589657	0.9563	0.3804	0.6861	0.4175	0.407
4	190022617	rs1579725222	-0.9249	0.002011	-0.9446	0.002892	-0.9648	4.91e-06	-1.146	8.33e-06	intergenic	FRG1;FRG2	NA	NA	NA	NA	NA	NA	NA	NA	NA
18	10495395	rs484349	0.6137	0.002015	0.6084	0.003477	0.6028	2.42e-05	0.6883	4.01e-05	intergenic	APCDD1;NAPG	10495392	T	G	0.563898	0.6468	0.5	0.7148	0.4324	0.455
18	10495083	rs568934	0.6141	0.002016	0.6091	0.003471	0.6049	2.26e-05	0.69	3.87e-05	intergenic	APCDD1;NAPG	10495080	G	A	0.563898	0.6468	0.5	0.7148	0.4324	0.455
18	10497409	rs505665	0.6141	0.002016	0.6091	0.003471	0.6119	1.7e-05	0.6924	3.48e-05	intergenic	APCDD1;NAPG	10497406	A	G	0.563099	0.6468	0.5	0.711	0.4334	0.455
18	10497829	rs660030	0.6141	0.002016	0.6091	0.003471	0.6105	1.83e-05	0.69	3.87e-05	intergenic	APCDD1;NAPG	10497826	G	A	0.564097	0.6468	0.5	0.7148	0.4334	0.455
5	148765101	rs2400704	-0.6147	0.002025	-0.5511	0.009371	-0.547	0.0001259	-0.6664	8.69e-05	intergenic	HTR4;ADRB2	148144664	C	T	0.53135	0.3472	0.5303	0.6808	0.5109	0.5409
7	32728864	rs118001891	0.7406	0.002025	0.8366	0.0007636	0.6899	7.48e-05	0.7832	9.62e-05	ncRNA_exonic	ZNRF2P1	32768476	T	C	0.238618	0.1399	0.1729	0.3079	0.1769	0.3569
5	90278537	rs72781008	1.011	0.002026	1.158	0.000567	0.8808	0.0009319	1.111	0.0001278	ncRNA_intronic	LINC01339	89574354	G	C	0.0451278	0	0.0591	0.0015	0.0746	0.1104
17	12465885	rs1585412	0.61	0.002029	0.7728	0.0002255	0.315	0.03175	0.4757	0.004334	intergenic	/IAP2K4;LINC0067	12369202	A	C	0.752995	0.7609	0.5331	0.9281	0.6372	0.7832
13	45578354	rs3014922	0.6202	0.002038	0.791	0.0002566	0.2225	0.1212	0.3882	0.01917	intronic	ERICH6B	46152489	A	G	0.665335	0.8006	0.5692	0.7262	0.501	0.681
5	2335823	rs62330978	0.6046	0.00205	0.5902	0.003656	0.3638	0.008041	0.5872	0.0002726	intergenic	:100506858;LSIN	2335937	T	C	0.364617	0.372	0.3429	0.2504	0.4334	0.456
4	130575662	rs17481053	-0.7496	0.002051	-0.8146	0.00164	-0.4927	0.003804	-0.8276	7.19e-05	intergenic	INC02479;SNHG2	131496817	C	G	0.159345	0.0298	0.2017	0.0212	0.4115	0.1902
12	42105985	rs78536352	0.8176	0.002065	0.8859	0.001604	0.6422	0.0009279	0.8976	5.9e-05	exonic	GXYLT1	NA	NA	NA	NA	NA	NA	NA	NA	NA
2	90370561	rs796550071	-1.206	0.002069	-1.047	0.007757	-1.065	2.39e-05	-1.042	0.0005008	intergenic	C101927050;LSP:	NA	NA	NA	NA	NA	NA	NA	NA	NA
8	102739842	rs62524037	-1.039	0.002074	-0.8983	0.009125	-1.052	1.27e-05	-0.8937	0.0008692	intergenic	:101927245;GAS	103752070	G	C	0.0836661	0.1081	0.2594	0.0045	0.0885	0.0358
18	10492404	rs638170	-0.6071	0.00208	-0.5657	0.005754	-0.5835	3.49e-05	-0.6961	2.98e-05	intergenic	APCDD1;NAPG	10492401	G	A	0.510982	0.6478	0.4827	0.5227	0.4324	0.455
5	132520835	rs11242118	-1.044	0.00209	-1.091	0.002819	-0.9093	7.89e-05	-0.9387	0.0006002	intergenic	IRF1;IL5	131856527	G	A	0.0545128	0	0.1066	0.0076	0.164	0.0245
4	66774343	rs13120688	0.8661	0.002093	1.068	0.0003621	0.3981	0.054	0.6299	0.007407	intergenic	1269A;LOC10192	67640061	G	T	0.78774	0.6071	0.8761	0.7065	0.9602	0.8436
4	66774502	rs13122551	0.8661	0.002093	1.068	0.0003621	0.3981	0.054	0.6299	0.007407	intergenic	1269A;LOC10192	67640220	C	T	0.78754	0.6071	0.8761	0.7057	0.9602	0.8436
15	49842063	rs34693309	0.7506	0.002093	0.8326	0.001399	0.5706	0.001166	0.796	9.49e-05	intergenic	DTWD1;ATP8B4	50134260	C	T	0.196286	0.4067	0.2075	0.0703	0.1531	0.1861
17	43857696	rs72836564	0.8043	0.002098	0.9608	0.0004004	0.2881	0.1654	0.4421	0.05368	UTR3	CD300LG	41935064	G	A	0.0946486	0	0.098	0.0885	0.1899	0.1002
5	2336599	rs1545901	0.6051	0.002118	0.6023	0.003185	0.3985	0.004852	0.6097	0.0001745	intergenic	:100506858;LSIN	2336713	G	A	0.33107	0.2202	0.3429	0.2481	0.4304	0.4468
7	32763067	rs12672074	0.7116	0.00212	0.7495	0.002128	0.6709	7.85e-05	0.7803	6.63e-05	downstream	LINC00997	32802679	C	G	0.235224	0.1399	0.17	0.298	0.1779	0.3538
7	32766744	rs73094218	0.7116	0.00212	0.7495	0.002128	0.6709	7.85e-05	0.7803	6.63e-05	intergenic	\C00997;DPY19L1	32806356	C	T	0.235224	0.1399	0.17	0.298	0.1779	0.3538
17	12467764	rs1460319	0.6029	0.00212	0.7712	0.000211	0.331	0.02224	0.4747	0.003794	intergenic	/IAP2K4;LINC0067	12371081	G	A	0.720248	0.7371	0.5115	0.8419	0.6312	0.7781
19	15517012	rs10419316	0.6344	0.00212	0.7475	0.0005861	0.1658	0.2839	0.3799	0.03124	intronic	CYP4F22	15627823	G	A	0.161342	0.0704	0.2133	0.1135	0.336	0.1033
10	24520873	rs7916984	-0.6102	0.002121	-0.5695	0.005867	-0.4404	0.001372	-0.672	5.64e-05	intronic	KIAA1217	24809802	A	C	0.607029	0.6181	0.5086	0.8744	0.4881	0.4264
3	91526546	rs1342605245	-0.8208	0.002124	-0.8614	0.002264	-0.7703	6.33e-05	-0.8132	0.0002539	intergenic	NONE;NONE	NA	NA	NA	NA	NA	NA	NA	NA	NA
19	17407735	rs4808632	-0.823	0.002125	-0.8399	0.003168	-0.5604	0.001621	-0.8662	0.0001107	ncRNA_intronic	BISPR	17518544	C	T	0.334465	0.2153	0.2291	0.6051	0.1968	0.3078
11	25679443	rs1493664	-0.6074	0.002126	-0.6045	0.004003	-0.4272	0.002137	-0.6508	8.93e-05	intergenic	LUZP2;ANO3	25700989	T	C	0.428514	0.5605	0.5418	0.1437	0.5616	0.4601
20	18464624	rs62218708	0.7687	0.00213	0.9452	0.0002805	0.6367	0.0005412	0.7964	9.61e-05	intronic	DZANK1	18445268	G	T	0.139976	0.0585	0.1859	0.084	0.2316	0.1728
12	98169817	rs7294783	2.144	0.00214	2.346	0.0007968	1.417	0.03404	1.6	0.02	intergenic	/IIR4303;SLC9A7F	98563595	A	G	0.0211661	0	0.0144	0.0628	0.0129	0
5	2335114	rs1873952	0.6104	0.002157	0.5961	0.003817	0.4418	0.002076	0.6455	8.69e-05	intergenic	:100506858;LSIN	2335228	C	T	0.301118	0.1736	0.3285	0.2163	0.4175	0.408
19	47965610	rs11671919	0.5992	0.00216	0.75	0.0003758	0.4286	0.002161	0.6593	7.1e-05	intergenic	SNAR-C2;BSPH1	48468867	G	T	0.363019	0.3393	0.4914	0.0734	0.5507	0.4949
19	47965983	rs7253895	0.5992	0.00216	0.75	0.0003758	0.4286	0.002161	0.6593	7.1e-05	intergenic	SNAR-C2;BSPH1	48469240	A	G	0.363419	0.3393	0.4957	0.0734	0.5517	0.4928
2	94557745	rs1439317531	-0.9154	0.00217	-0.9568	0.002086	-0.9166	4.93e-05	-0.8911	0.0004477	intergenic	;1P14;LOC10013:	NA	NA	NA	NA	NA	NA	NA	NA	NA
5	109168900	rs10477440	0.846	0.002172	0.911	0.001354	0.7376	0.0003856	0.9712	3.06e-05	intronic	FER	108504601	C	A	0.139577	0.0704	0.111	0.1581	0.2107	0.1329
18	10500723	rs34508908	0.6108	0.002172	0.6009	0.003982	0.6152	1.76e-05	0.697	3.35e-05	intergenic	APCDD1;NAPG	NA	NA	NA	NA	NA	NA	NA	NA	NA
10	112806389	rs10885385	-0.7273	0.002175	-0.9111	0.0005951	-0.5383	0.0007305	-0.8978	1.1e-05	intronic	VTI1A	114566148	G	A	0.172125	0.2688	0.2248	0.0144	0.2624	0.1554
20	30594851	rs1414587902	-0.9837	0.002177	-1.204	0.0007931	-0.9295	1.4e-05	-1.062	5e-05	intergenic	FRG1BP;DEFB115	NA	NA	NA	NA	NA	NA	NA	NA	NA
2	113511844	rs113046100	-1.116	0.002178	-1.085	0.004085	-0.966	2.69e-05	-1.174	5.32e-05	intergenic	XD4L1;PGM5P3-/	NA	NA	NA	NA	NA	NA	NA	NA	NA
9	19810956	rs143574528	1.314	0.002192	1.401	0.001377	0.6192	0.07228	0.7451	0.05091	intergenic	SLC24A2;MLLT3	19810954	ATTC	A	0.0441294	0.1438	0.0663	0.0053	0.0119	0.0112
22	22349323	rs200395532	-0.8992	0.002192	-0.8408	0.005973	-0.8086	7.83e-05	-0.6414	0.006124	intergenic	3MS1P20;ZNF280	NA	NA	NA	NA	NA	NA	NA	NA	NA
6	129526828	rs2006724	-0.635	0.002193	-0.7232	0.00105	-0.4391	0.002021	-0.6853	8.69e-05	intergenic	.AMA2;ARHGAP1	129847973	A	G	0.352835	0.1766	0.4063	0.3578	0.492	0.3466
6	52255012	rs578360	-1.418	0.002217	-1.438	0.003842	-1.35	1.25e-05	-1.186	0.0009097	intergenic	IL17F;MCM3	52119810	C	A	0.161941	0.001	0.0562	0.4977	0.0586	0.0552
1	75409863	rs1249792	0.6535	0.002219	0.62	0.005787	0.5295	0.0009106	0.7267	7.98e-05	intronic	SLC44A5	75875548	A	G	0.335463	0.1538	0.3948	0.4402	0.334	0.3405
22	25014928	rs34259162	-0.7977	0.002236	-0.8216	0.003048	-0.3807	0.01658	-0.7377	0.0003347	intergenic	VIEM211;KIAA16	25410895	A	G	0.264976	0.4722	0.2925	0.1573	0.2087	0.2352
4	114557336	rs111424248	1.313	0.002246	1.373	0.001887	0.5278	0.1755	0.8319	0.04331	intergenic	ARSJ;UGT8	115478492	A	G	0.0341454	0.005	0.0187	0.0635	0.0308	0.0389
4	114557420	rs116399090	1.313	0.002246	1.373	0.001887	0.5278	0.1755	0.8319	0.04331	intergenic	ARSJ;UGT8	115478576	C	G	0.0341454	0.005	0.0187	0.0635	0.0308	0.0389
2	97149653	rs146031240	-0.9794	0.002247	-1.141	0.001243	-0.953	2.14e-05	-1.023	0.0001481	intronic	ANKRD36	NA	NA	NA	NA	NA	NA	NA	NA	NA
12	37485693	rs200137328	-1.137	0.002247	-1.077	0.005221	-0.989	4.55e-05	-0.7241	0.007591	intergenic	NONE;ALG10B	NA	NA	NA	NA	NA	NA	NA	NA	NA
17	21736655	rs1413640535	1.013	0.002268	0.954	0.005347	0.861	8.45e-05	0.8833	0.0006854	intergenic	KCNJ18;UBBP4	NA	NA	NA	NA	NA	NA	NA	NA	NA
12	98071833	rs7970872	0.5958	0.00227	0.7291	0.0004399	0.07138	0.6068	0.2334	0.1426	intergenic	/IIR4303;SLC9A7F	98465611	A	T	0.608427	0.7649	0.6744	0.4531	0.504	0.7178
17	43859474	rs76557360	0.7981	0.002277	0.9549	0.0004364	0.2808	0.1766	0.4337	0.05858	intronic	CD300LG	41936842	G	A	0.091853	0	0.098	0.0779	0.1899	0.1002
17	43859475	rs80053172	0.7981	0.002277	0.9549	0.0004364	0.2808	0.1766	0.4337	0.05858	intronic	CD300LG	41936843	C	T	0.0920527	0	0.098	0.0779	0.1909	0.1002
23	121511922	rs141705124	0.7307	0.002284	0.8089	0.0008156	0.2522	0.1943	0.4368	0.03813	intergenic	MIR3672;GRIA3	NA	NA	NA	NA	NA	NA	NA	NA	NA
20	29864619	rs1172885425	-0.9902	0.002288	-0.8946	0.007666	-0.8438	7.09e-05	-0.8124	0.00107	intergenic	\M242B;MIR447	NA	NA	NA	NA	NA	NA	NA	NA	NA
1	13685745	rs7545576	-0.7168	0.002305	-0.7436	0.002894	-0.6531	9.3e-05	-0.8137	4.62e-05	intergenic	PDPN;PRDM2	14012240	T	C	0.783946	0.7748	0.7161	0.8616	0.7535	0.7679
20	29866900	rs1186706158	2.262	0.002307	2.118	0.004323	1.287	8.68e-05	1.537	0.0003644	intergenic	\M242B;MIR447	NA	NA	NA	NA	NA	NA	NA	NA	NA
14	100420312	rs916154687	1.976	0.00231	2.028	0.001642	1.145	0.01792	1.925	0.0003988	intronic	WDR25	100886649	A	AT	0.047524	0	0.0216	0.1437	0.0209	0.0123
1	75385590	rs143991175	-0.6479	0.002325	-0.804	0.0003706	-0.3464	0.02467	-0.5914	0.000787	intronic	SLC44A5	75851275	C	T	0.584065	0.9563	0.3761	0.6664	0.4175	0.408
2	188246517	rs4552156	1.124	0.002334	1.291	0.0006043	0.7874	0.002773	1.308	1.48e-05	ncRNA_intronic	LINC01090	189111244	C	T	0.14976	0.2173	0.0375	0.1762	0.0487	0.228
1	125070257	rs1295606189	-1.186	0.002353	-1.212	0.002045	-1.338	3.32e-06	-1.334	3.59e-05	intergenic	NONE;NONE	NA	NA	NA	NA	NA	NA	NA	NA	NA
2	90292499	rs1344435743	-0.8154	0.002361	-0.8291	0.003491	-0.79	3.96e-05	-0.797	0.0003248	intergenic	C101927050;LSP:	NA	NA	NA	NA	NA	NA	NA	NA	NA
19	35151748	rs36072726	1.131	0.002362	1.298	0.0006681	0.7211	0.01121	1.119	0.0002595	intronic	FXYD7	35642651	G	T	0.0485224	0.002	0.0706	0.0015	0.0755	0.1166
13	85237597	rs59015168	0.8413	0.002368	0.9977	0.0004523	0.2706	0.2253	0.5425	0.02629	intergenic	\C00375;LINC003	85811732	G	A	0.0756789	0.0665	0.049	0.062	0.0676	0.1309
12	98095056	rs6538839	0.5954	0.002373	0.7296	0.0004407	0.06445	0.6427	0.22	0.1672	intergenic	/IIR4303;SLC9A7F	98488834	A	G	0.620607	0.7669	0.6628	0.5083	0.5	0.7157
5	118275574	rs34434678	0.7019	0.002374	0.7991	0.0007884	0.1739	0.3199	0.3663	0.06456	intergenic	\C02147;LINC021	117611269	G	C	0.233427	0.0248	0.1873	0.4796	0.2346	0.1472
8	102723100	rs4734665	-0.9944	0.002383	-0.9461	0.005906	-1.03	1.15e-05	-0.8931	0.0007054	intergenic	:101927245;GAS	103735328	C	T	0.105032	0.0982	0.2738	0.0809	0.0915	0.0389
1	66793601	rs474409	0.8324	0.002389	0.8659	0.002632	0.8421	0.0001046	0.9362	9.28e-05	intergenic	TCTEX1D1;INSL5	67259284	A	C	0.877596	0.9901	0.8948	0.8048	0.83	0.8967
12	98151936	rs78315733	1.934	0.00239	2.115	0.0009023	1.169	0.05279	1.403	0.02516	intergenic	/IIR4303;SLC9A7F	98545714	C	T	0.0223642	0	0.0173	0.0658	0.0129	0
12	98156662	rs74344830	1.934	0.00239	2.115	0.0009023	1.169	0.0527	1.404	0.02507	intergenic	/IIR4303;SLC9A7F	98550440	C	A	0.0223642	0	0.0173	0.0658	0.0129	0
16	34698567	rs1402649164	-1.056	0.002391	-0.8887	0.0113	-1.032	1.02e-05	-1.189	4.31e-05	intergenic	CCNYL3;CCNYL3	NA	NA	NA	NA	NA	NA	NA	NA	NA
1	66808227	rs958938	0.8246	0.002394	0.8539	0.002712	0.8559	6.26e-05	0.9184	0.0001015	intergenic	INSL5;DNAI4	67273910	A	G	0.863019	0.9901	0.889	0.7519	0.83	0.8978
12	42106007	rs75194020	0.81	0.002396	0.8626	0.002167	0.7025	0.0002972	0.8943	6.93e-05	exonic	GXYLT1	NA	NA	NA	NA	NA	NA	NA	NA	NA

2	78934537	rs72814629	0.7448	0.002397	0.8888	0.0004132	0.2151	0.2563	0.3631	0.08345	intergenic	C105374820;REG	79161663	C	T	0.135783	0.1339	0.1182	0.1097	0.1113	0.2106
13	110458327	rs7325334	0.6471	0.002403	0.5919	0.007845	0.5361	0.0003016	0.689	9.68e-05	intronic	)L4A2;COL4A2-A	111110674	C	G	0.482029	0.2232	0.4856	0.6407	0.5924	0.4182
4	75835087	rs10856861	0.6349	0.002408	0.6821	0.001734	0.295	0.05025	0.5885	0.0007115	intergenic	USO1;PPEF2	76756240	C	T	0.36861	0.6458	0.4481	0.1581	0.2525	0.4305
12	59534851	rs1345484485	-1.326	0.002411	-1.161	0.008149	-1.137	4.09e-05	-1.067	0.001217	intergenic	LRIG3;SLC16A7	NA	NA	NA	NA	NA	NA	NA	NA	NA
3	195593596	rs7635027	-0.6234	0.002414	-0.7448	0.0008487	-0.2856	0.03927	-0.5921	0.0003923	intergenic	'OD;LOC1053742	195320467	G	A	0.423922	0.7143	0.4193	0.3464	0.3708	0.2873
3	195594972	rs9886971	-0.6234	0.002414	-0.7448	0.0008487	-0.2856	0.03927	-0.5921	0.0003923	intergenic	'OD;LOC1053742	195321843	T	C	0.430511	0.7153	0.4222	0.3684	0.3708	0.2883
7	43497217	rs76764444	0.7459	0.002418	0.7227	0.005174	0.7945	4.19e-05	0.6772	0.001964	intronic	HECW1	43536816	G	A	0.10623	0.0079	0.1542	0.0083	0.2127	0.1963
7	32772484	rs12667060	0.7029	0.002422	0.7417	0.002386	0.6662	8.83e-05	0.7747	7.51e-05	intergenic	IC00997;DPY19L1	32812096	A	G	0.236222	0.1399	0.1715	0.298	0.1789	0.3569
3	195590018	rs9886984	-0.6289	0.002428	-0.7644	0.0007841	-0.2575	0.06174	-0.5563	0.0008235	intergenic	'OD;LOC1053742	195316889	G	T	0.355631	0.5486	0.3948	0.3555	0.3032	0.183
5	118272727	rs6595106	0.6932	0.00243	0.8093	0.0006235	0.1949	0.2641	0.3961	0.04508	intergenic	\C02147;LINC021	117608422	C	T	0.233027	0.0248	0.1859	0.4803	0.2326	0.1472
1	75389932	rs1249828	-0.6553	0.002432	-0.8266	0.0003318	-0.3429	0.02951	-0.6431	0.0003714	intronic	SLC44A5	75855617	A	C	0.617612	0.9563	0.3818	0.7489	0.4225	0.4591
12	98153988	rs78571273	1.931	0.002437	2.112	0.0009209	1.166	0.05332	1.399	0.02551	intergenic	/IIR4303;SLC9A7F	98547766	C	T	0.0223642	0	0.0173	0.0658	0.0129	0
17	57281644	rs10515137	1.022	0.002444	1.114	0.001154	0.1378	0.62	0.4042	0.1854	intronic	MSI2	55359005	C	T	0.0597045	0.0208	0.0605	0.0915	0.0845	0.0307
15	99077909	rs1703774	-0.7279	0.002451	-0.5677	0.01947	-0.4776	0.002248	-0.7767	8.95e-05	intergenic	LUNAR1;SYNM	99621138	G	A	0.314696	0.3571	0.3141	0.441	0.2256	0.1922
18	10512629	rs563331	0.5936	0.002459	0.6177	0.002706	0.6427	6.41e-06	0.7157	1.81e-05	intergenic	APCDD1;NAPG	10512626	T	C	0.564696	0.6716	0.4986	0.6982	0.4344	0.455
5	2336289	rs56262971	0.6046	0.002464	0.6076	0.00316	0.4281	0.003055	0.6499	8.47e-05	intergenic	:100506858;LSIN	2336403	C	T	0.260982	0.2024	0.3084	0.0514	0.4195	0.408
1	83358677	rs4112801	1.176	0.002469	1.021	0.008848	0.9401	0.0001126	1.294	5.19e-05	intergenic	\C01362;LINC017	NA	NA	NA	NA	NA	NA	NA	NA	NA
4	130565686	rs62319784	-0.736	0.002473	-0.783	0.002498	-0.5055	0.002976	-0.8177	9.04e-05	intergenic	INC02479;SNHG2	131486841	G	T	0.161142	0.0308	0.1916	0.0499	0.3926	0.1861
13	22992164	rs35977743	0.69	0.002478	0.8359	0.000434	0.2301	0.1722	0.3678	0.05363	intergenic	LINC00621;SGCG	NA	NA	NA	NA	NA	NA	NA	NA	NA
13	101600111	rs9585734	0.5875	0.002483	0.5527	0.00694	0.3442	0.01225	0.6673	5.92e-05	intronic	ITGBL1	NA	NA	NA	NA	NA	NA	NA	NA	NA
2	230898714	rs1465834	0.6704	0.002504	0.696	0.002885	0.6853	3.83e-05	0.6437	0.0007035	ncRNA_intronic	GCSIR	231763429	G	A	0.327875	0.1577	0.2478	0.5658	0.2783	0.2894
12	98099214	rs10777901	0.5777	0.002508	0.729	0.0003571	0.02471	0.8551	0.1673	0.2798	intergenic	/IIR4303;SLC9A7F	98492992	C	A	0.580272	0.7669	0.4755	0.4561	0.497	0.7157
1	75392814	rs1249820	-0.643	0.002509	-0.814	0.0003423	-0.3401	0.02845	-0.617	0.0005515	intronic	SLC44A5	75858499	A	G	0.601438	0.9563	0.3833	0.6717	0.4404	0.4611
11	67780112	rs1403582266	-0.9165	0.002515	-0.784	0.01184	-1.082	8.3e-07	-1.074	3.19e-05	intergenic	LDH3B2;FAM86C	NA	NA	NA	NA	NA	NA	NA	NA	NA
5	2336408	rs55671606	0.5907	0.002521	0.5765	0.004419	0.361	0.008556	0.58	0.0003204	intergenic	:100506858;LSIN	2336522	A	AC	0.364217	0.372	0.3415	0.2496	0.4334	0.456
15	23180332	rs62010781	-1.206	0.002524	-1.015	0.0114	-1	8.85e-05	-1.077	0.0004865	intronic	GOLGA6L22	NA	NA	NA	NA	NA	NA	NA	NA	NA
1	13698233	rs4662019	-0.697	0.002525	-0.6788	0.00537	-0.5914	0.0002746	-0.7729	7.51e-05	intergenic	PDPN;PRDM2	NA	NA	NA	NA	NA	NA	NA	NA	NA
17	21736634	rs1389500236	1.03	0.002529	0.9824	0.005346	0.9247	3.66e-05	0.925	0.0005151	intergenic	KCNJ18;UBBP4	NA	NA	NA	NA	NA	NA	NA	NA	NA
1	13691325	rs6692099	-0.7017	0.002533	-0.6996	0.004559	-0.6393	0.0001129	-0.809	4.34e-05	intergenic	PDPN;PRDM2	14017820	T	C	0.769169	0.7798	0.7104	0.8056	0.7525	0.7679
4	130566384	rs62319786	-0.7292	0.002546	-0.7972	0.002146	-0.5154	0.00252	-0.8211	8.4e-05	intergenic	INC02479;SNHG2	131487539	A	G	0.161342	0.0317	0.1916	0.0499	0.3926	0.1861
5	14886765	rs13172779	0.5819	0.002553	0.5426	0.007246	0.5582	7.5e-05	0.6138	0.000162	intergenic	ANKH;LINC0214S	14886874	A	G	0.389976	0.3194	0.3905	0.3722	0.5378	0.3344
18	10513245	rs645477	0.5909	0.002554	0.6147	0.002896	0.6433	6.28e-06	0.7119	2.07e-05	intergenic	APCDD1;NAPG	10513242	A	G	0.565096	0.6726	0.4986	0.6989	0.4344	0.455
18	10506327	rs6505569	-0.5768	0.00257	-0.5846	0.004069	-0.5797	2.44e-05	-0.691	2.09e-05	intergenic	APCDD1;NAPG	10506324	A	G	0.567891	0.6687	0.4971	0.7141	0.4354	0.453
12	98154920	rs77714907	1.915	0.002574	2.115	0.0009023	1.162	0.05408	1.397	0.02585	intergenic	/IIR4303;SLC9A7F	NA	NA	NA	NA	NA	NA	NA	NA	NA
4	130565332	rs62319782	-0.7333	0.002575	-0.7811	0.002573	-0.5158	0.002521	-0.8419	6.33e-05	intergenic	INC02479;SNHG2	131486487	T	C	0.161142	0.0308	0.1916	0.0499	0.3926	0.1861
4	130565641	rs62319783	-0.7333	0.002575	-0.7811	0.002573	-0.4993	0.003306	-0.8119	0.0001001	intergenic	INC02479;SNHG2	131486796	T	G	0.161142	0.0308	0.1916	0.0499	0.3926	0.1861
4	130566858	rs142922043	-0.7333	0.002575	-0.7811	0.002573	-0.4993	0.003306	-0.8119	0.0001001	intergenic	INC02479;SNHG2	131488013	T	TA	0.161342	0.0317	0.1916	0.0499	0.3926	0.1861
4	130568706	rs76781910	-0.7333	0.002575	-0.7811	0.002573	-0.4993	0.003306	-0.8119	0.0001001	intergenic	INC02479;SNHG2	131489861	C	CCAGT	0.16234	0.0317	0.1916	0.0499	0.3976	0.1861
4	130574004	rs56023524	-0.7333	0.002575	-0.7811	0.002573	-0.4993	0.003306	-0.8119	0.0001001	intergenic	INC02479;SNHG2	131495159	T	C	0.161741	0.0298	0.1916	0.0499	0.3966	0.1861
18	10505221	rs562584	0.6016	0.002581	0.6088	0.003499	0.6287	1.26e-05	0.7112	2.65e-05	intergenic	APCDD1;NAPG	10505218	T	C	0.56889	0.6677	0.5029	0.7179	0.4334	0.4519
6	166203741	rs13206417	-1.09	0.002586	-1.439	0.0008501	-0.9612	5.67e-05	-1.19	0.000118	intergenic	:DAT;LOC101929	166617229	A	G	0.148163	0.12	0.1988	0.1755	0.0905	0.1636
18	69040123	rs140591155	1.547	0.002598	1.724	0.0008483	0.8559	0.07238	1.221	0.01271	intronic	CCDC102B	66707360	G	A	0.00978435	0	0.0187	0	0.0268	0.0092
18	69040124	rs145713216	1.547	0.002598	1.724	0.0008483	0.8559	0.07238	1.221	0.01271	intronic	CCDC102B	66707361	G	A	0.00978435	0	0.0187	0	0.0268	0.0092
1	75392961	rs1249819	-0.643	0.0026	-0.814	0.0003423	-0.3391	0.02898	-0.6167	0.0005601	intronic	SLC44A5	75858646	C	T	0.601038	0.9563	0.3833	0.671	0.4404	0.4601
5	118274579	rs6880921	0.691	0.002603	0.8138	0.0006228	0.1814	0.2976	0.3893	0.04936	intergenic	\C02147;LINC021	117610274	T	A	0.236821	0.0248	0.1873	0.4924	0.2346	0.1472
7	60951377	rs1428529190	0.8819	0.002604	0.9067	0.003367	0.7901	8.66e-05	0.7566	0.001248	intergenic	NONE;NONE	NA	NA	NA	NA	NA	NA	NA	NA	NA
1	212446872	rs483954	0.5964	0.002606	0.6158	0.002617	0.432	0.002606	0.6144	0.0002387	downstream	NENF	212620214	A	G	0.384185	0.5625	0.4524	0.3782	0.3648	0.18
19	21733965	rs4319879	0.8576	0.002607	0.8181	0.005715	0.6254	0.001677	0.9444	0.0001018	intronic	ZNF100	21916767	G	A	0.691294	0.7718	0.8127	0.5598	0.8549	0.5317
1	66794876	rs12037488	0.8241	0.002609	0.8564	0.002904	0.8543	7.58e-05	0.9259	0.0001074	intergenic	TCTEX1D1;INSL5	67260559	T	G	0.871805	0.9901	0.8919	0.7844	0.83	0.8967
1	66797141	rs558983	0.8241	0.002609	0.8564	0.002904	0.8422	9.79e-05	0.9259	0.0001074	downstream	INSL5	67262824	T	G	0.871805	0.9901	0.8919	0.7844	0.83	0.8967
1	66800473	rs575850	0.8241	0.002609	0.8564	0.002904	0.8422	9.79e-05	0.9259	0.0001074	intronic	INSL5	67266156	C	G	0.871206	0.9901	0.8919	0.7821	0.83	0.8967
1	66801073	rs549148	0.8241	0.002609	0.8564	0.002904	0.8422	9.79e-05	0.9259	0.0001074	exonic	INSL5	67266756	T	A	0.871206	0.9901	0.8919	0.7821	0.83	0.8967
2	91967751	rs77080916	0.8325	0.002611	0.9488	0.001062	0.8012	0.0001074	0.9063	9.16e-05	intergenic	ACTR3BP2;NONE	NA	NA	NA	NA	NA	NA	NA	NA	NA
5	118281937	rs41456648	0.6934	0.002617	0.8144	0.0006399	0.185	0.2953	0.3755	0.06101	upstream	LINC02148	117617632	A	G	0.211661	0.0278	0.1902	0.3896	0.2356	0.1513
3	195590100	rs9886981	-0.6239	0.002618	-0.7589	0.0008528	-0.2588	0.0609	-0.5757	0.0006023	intergenic	'OD;LOC1053742	195316971	C	T	0.355232	0.5486	0.3948	0.354	0.3032	0.183
5	14890768	rs863118	0.5824	0.002627	0.4924	0.01504	0.572	7.96e-05	0.6041	0.0002502	intergenic	ANKH;LINC0214S	14890877	T	C	0.44988	0.3542	0.4063	0.5961	0.506	0.3241
1	75397382	rs1249812	-0.6408	0.002639	-0.7984	0.0004307	-0.3413	0.0283	-0.6334	0.0004075	intronic	SLC44A5	75863067	G	A	0.591653	0.9563	0.3775	0.6513	0.4225	0.4611
11	16917805	rs17699635	1.543	0.002656	1.744	0.0007113	0.8041	0.09716	1.172	0.01777	intronic	PLEKHA7	16939352	C	T	0.013778	0.001	0.0259	0.003	0.0328	0.0133
4	70550054	rs28770574	0.6519	0.002664	0.5952	0.008768	0.6955	5.59e-06	0.6691	0.0001442	intergenic	AMTN;AMBN	71415771	A	T	0.719249	0.5923	0.7378	0.8147	0.7406	0.6861
1	101805303	rs1327593	0.5905	0.002668	0.5425	0.009075	0.5781	5.29e-05	0.6394	0.0001065	intronic	OLFM3	102270859	C	T	0.528355	0.6488	0.4179	0.6021	0.5875	0.3221
4	130581059	rs4469098	-0.7202	0.002668	-0.7979	0.001871	-0.5087	0.002633	-0.8245	6.77e-05	intergenic	INC02479;SNHG2	131502214	G	A	0.168131	0.0317	0.2032	0.0492	0.4155	0.1902
10	42272451	rs201392097	-1.474	0.002671	-1.453	0.004333	-1.481	0.0002276	-1.739	5.77e-05	intergenic	\ONE;LOC44166	NA	NA	NA	NA	NA	NA	NA	NA	NA
6	166203702	rs13206393	-1.086	0.002688	-1.435	0.0008824	-0.9563	6.15e-05	-1.184	0.0001264	intergenic	:DAT;LOC101929	166617190	A	G	0.148363	0.121	0.1988	0.1755	0.0905	0.1636
6	166203835	rs34863185	-1.086	0.002688	-1.435	0.0008824	-0.9563	6.15e-05	-1.184	0.0001264	intergenic	:DAT;LOC101929	166617323	A	G	0.147963	0.12	0.1988	0.1747	0.0905	0.1636
15	17060143	rs1229410741	-1.025	0.002689	-0.8389	0.01492	-1.032	1.04e-05	-0.9901	0.0003181	intergenic	NONE;NONE	NA	NA	NA	NA	NA	NA	NA	NA	NA
2	78948545	rs17015937	0.7363	0.002714	0.8711	0.0005286	0.2016	0.2896	0.3698	0.07882	intergenic	C105374820;REG	79175671	G	A	0.139177	0.1349	0.111	0.1346	0.1054	0.2045
13	110874892	rs34736615	0.6225	0.002715	0.6395	0.002898	0.5784	6.82e-05	0.659	0.0001076	intergenic	'RECSIT;ANKRD1	111527239	G	GA	0.484824	0.8026	0.4424	0.4516	0.4622	0.2556
2	97149627	rs112097945	-0.8403	0.002719	-1.002	0.001008	-0.7332	0.0001949	-0.9215	8.28e-05	intronic	ANKRD36	NA	NA	NA	NA	NA	NA	NA	NA	NA
1	75372536	rs57187191	-0.6404	0.002722	-0.7977	0.00043	-0.3767	0.01631	-0.6352	0.0003983	intronic	SLC44A5	75838221	A	AGT	0.600839	0.9563	0.3818	0.6884	0.4225	0.455
1	75396841	rs1249813	-0.6404	0.002722	-0.7977	0.00043	-0.3618	0.02071	-0.6352	0.0003983	intronic	SLC44A5	75862526	C	A	0.594449	0.9563	0.3761	0.6687	0.4225	0.453
1	5033626	rs7533014	0.6523	0.002728	0.6926	0.002172	0.6529	4.75e-05	0.616	0.0007994	intergenic	AJAP1;MIR4689	5093686	T	C	0.322085	0.1597	0.2608	0.5144	0.3091	0.2863
5	109171545	rs73779051	0.8261	0.002728	0.8787	0.001915	0.7168	0.0005318	0.952	4.06e-05	intronic	FER	108507246	G	A	0.139776	0.0704	0.111	0.1589	0.2107	0.1329
18	10511305	rs559412	0.6044	0.00274	0.5854	0.005533	0.6921	2.12e-06	0.7317	1.89e-05	intergenic	APCDD1;NAPG	10511302	G	A	0.616014	0.6677	0.5245	0.8812	0.4384	0.4519
17	43864741	rs231539	0.7781	0.002754	0.9608	0.0004004	0.2795	0.178	0.4298	0.06026	intergenic	CD300LG;MPP2	41942109	T	C	0.907548	1	0.902	0.9221	0.8111	0.8957
8	5614374	rs73182234	0.8433	0.002765	1.086	0.0001973	0.3251	0.1134	0.4046	0.08813	intergenic	VID1;LOC100287	5471896	G	A	0.116813	0.2421	0.1816	0.0635	0.0974	0.0337
18	10507225	rs13381221	0.5995	0.002766	0.5819	0.005451	0.652	7.15e-06	0.7098	2.91e-05	intergenic	APCDD1;NAPG	10507222	T	C	0.612819	0.6657	0.5159	0.879	0.4334	0.4519
10	112801104	rs4918779	-0.6965	0.002771	-0.8592	0.0008742	-0.5069	0.001172	-0.8683	1.41e-05	intronic	VTI1A	114560863	A	G	0.182508	0.2579	0.2277	0.0545	0.2594	0.1667
8	67503910	rs147175072	2.776	0.002786	3.005	0.00102	1.418	0.08438	2.104	0.01129	intronic	CPA6	68416145	C	A	0.0135783	0.0327	0.0461	0	0	0.0031
5	2335691	rs62330976	0.5817	0.00282	0.5669	0.00493	0.3465	0.01156	0.572	0.0003664	intergenic	:100506858;LSIN	2335805	G	A	0.363019	0.372	0.3386	0.2496	0.4304	0.455
10	112798600	rs4918777	-0.7056	0.002825	-0.8823	0.000791	-0.5346	0.0007783	-0.8705	1.67e-05	intronic	VTI1A	114558359	A	G	0.181709	0.2619	0.2248	0.0507	0.2565	0.1687
17	21383493	rs4247138	1.156	0.002847	1.341	0.0006473	0.3043	0.3759	0.8382	0.01788	intronic	KCNJ12	21286805	G	A	0.0163738	0.001	0.0346	0.003	0.0368	0.0164
7	43509258	rs9639877	0.6622	0.002852	0.6372	0.006082	0.6825	6.78e-05	0.4888	0.01304	ncRNA_exonic	LUARIS	43548857	T	C	0.183506	0.0863	0.1657	0.1649	0.2147	0.2894
23	140925685	rs1948488	0.6128	0.002856	0.7288	0.000595	0.2232	0.1576	0.4358	0.01345	downstream	MIR320D2	NA	NA	NA	NA	NA	NA	NA	NA	NA
1	13672791	rs12039802	-0.6892	0.002859	-0.697	0.00427	-0.607	0.0001866	-0.8087	3.79e-05	intergenic	PDPN;PRDM2	13999286	C	T	0.721845	0.754	0.7032	0.7731	0.7336	0.6207
1	13672822	rs12745515	-0.6892	0.002859	-0.697	0.00427	-0.5988	0.0002309	-0.7976	4.95e-05	intergenic	PDPN;PRDM2	13999317	C	A	0.721246	0.754	0.7032	0.7716	0.7326	0.6207
1	13674523	rs4662010	-0.6892	0.002859	-0.697	0.00427	-0.607	0.0001866	-0.8087	3.79e-05	intergenic	PDPN;PRDM2	14001018	C	T	0.720048	0.754	0.7017	0.7693	0.7306	0.6207
1	13675372	rs4612622	-0.6892	0.002859	-0.697	0.00427	-0.607	0.0001866	-0.8087	3.79e-05	intergenic	PDPN;PRDM2	14001867	G	A	0.720048	0.754	0.7017	0.7693	0.7306	0.6207
1	13675927	rs4431839	-0.6892	0.002859	-0.697	0.00427	-0.607	0.0001866	-0.8087	3.79e-05	intergenic	PDPN;PRDM2	14002422	T	A	0.720048	0.754	0.7017	0.7693	0.7306	0.6207
2	130649502	rs61702261	-0.8119	0.002863	-0.8101	0.00492	-0.7501	0.0001187	-0.952	2.62e-05	intronic	POTEJ	NA	NA	NA	NA	NA	NA	NA	NA	NA
6	15205435	rs9396571	0.5669	0.002863	0.5678	0.004277	0.5524	4.66e-05	0.6336	6.18e-05	intergenic	.INC01108;JARID	15205666	A	G	0.514976	0.5437	0.4957	0.6558	0.336	0.4928
7	43456859	rs55893602	0.756	0.002863	0.7337	0.005099	0.7556	8.15e-05	0.6653	0.002122	intronic	HECW1	43496458	G	A	0.110423	0.0387	0.1499	0.0091	0.2028	0.1984
18	10486309	rs570738	0.5963	0.002876	0.572	0.005877	0.6187	1.66e-05	0.6759	6.37e-05	intronic	APCDD1	10486306	A	G	0.525359	0.6488	0.5447	0.5484	0.4592	0.4213
4	70541668	rs4146576	0.6494	0.002879	0.6112	0.007583	0.6865	1.01e-05	0.6848	0.000114	intergenic	AMTN;AMBN	71407385	A	G	0.719848	0.5923	0.7392	0.8169	0.7406	0.6851
6	166181600	rs34463169	-1.461	0.002892	-1.529	0.004541	-1.232	5.76e-05	-1.324	0.000506	intergenic	:DAT;LOC101929	166595088	T	C	0.0599042	0.0248	0.0591	0.0106	0.1024	0.1196
7	138018590	rs11971315	0.8984	0.002896	1.047	0.0007056	0.1876	0.4171	0.5278	0.03761	intergenic	CREB3L2;AKR1D1	137703336	G	A	0.125599	0.1756	0.0418	0.208	0.0606	0.089
18	10491444	rs500222	-0.5875	0.002898	-0.5405	0.008268	-0.5693	5.65e-05	-0.6905	3.58e-05	intergenic	APCDD1;NAPG	10491441	A	G	0.523363	0.6478	0.4986	0.5643	0.4354	0.4479
1	75372801	rs1249809	-0.6371	0.002902	-0.7971	0.0004664	-0.3772	0.01638	-0.6331	0.0004272	intronic	SLC44A5	75838486	G	A	0.622604	0.9563	0.3862	0.7685	0.4225	0.455
13	24384754	rs138669346	-1.864	0.002904	-1.714	0.006204	-1.593	2.56e-05	-1.807	0.0002705	intergenic	.INC00566;PARP	24958892	G	A	0.00439297	0	0.0058	0.0015	0.0119	0.0041
17	21320532	rs79937502	-0.9448	0.002913	-1.044	0.001402	-0.8683	0.0003418	-1.062	8.8e-05	intergenic	MAP2K3;KCNJ12	NA	NA	NA	NA	NA	NA	NA	NA	NA

4	130588387	rs11734630	-0.7419	0.002914	-0.8342	0.001838	-0.467	0.006289	-0.8069	0.0001366	intergenic	INC02479;SNHG2	131509542	A	C	0.159744	0.0308	0.1902	0.0507	0.3807	0.1912
8	102723335	rs12544182	-0.9727	0.002914	-0.9499	0.005731	-1.028	1.21e-05	-0.8866	0.0007698	intergenic	C101927245;GAS	103735563	C	G	0.105032	0.0982	0.2738	0.0809	0.0915	0.0389
8	102722463	rs112534099	-0.9687	0.002925	-0.9463	0.005703	-0.9838	1.93e-05	-0.8819	0.0007853	intergenic	C101927245;GAS	103734691	T	C	0.111821	0.0982	0.2752	0.1059	0.0915	0.0389
8	102722536	rs111706747	-0.9687	0.002925	-0.9463	0.005703	-0.9838	1.93e-05	-0.8819	0.0007853	intergenic	C101927245;GAS	103734764	G	T	0.111821	0.0982	0.2752	0.1059	0.0915	0.0389
8	102723949	rs78481800	-0.9687	0.002925	-0.9463	0.005703	-0.9838	1.93e-05	-0.8819	0.0007853	intergenic	C101927245;GAS	103736177	A	AGTAG	0.110623	0.0982	0.2723	0.1029	0.0915	0.0389
4	189924748	rs1264871212	-0.8082	0.00293	-0.9617	0.0008526	-0.7628	7.95e-05	-0.7905	0.000407	ncRNA_intronic	FRG1-DT	NA	NA	NA	NA	NA	NA	NA	NA	NA
5	118277059	rs5000371	0.6805	0.002933	0.8267	0.0005191	0.2072	0.2413	0.3774	0.05893	intergenic	NC02147;LINC021	117612754	T	C	0.204673	0.0248	0.1888	0.3631	0.2366	0.1544
4	130565780	rs62319785	-0.7168	0.002946	-0.783	0.002498	-0.4992	0.003308	-0.8064	0.0001059	intergenic	INC02479;SNHG2	131486935	A	G	0.161142	0.0308	0.1916	0.0499	0.3926	0.1861
4	94151091	rs28761031	0.6445	0.002947	0.6265	0.00598	0.149	0.3512	0.2025	0.2646	ncRNA_intronic	LOC101929210	95072242	G	A	0.198083	0.2063	0.3055	0.1899	0.175	0.1483
17	43873909	rs231526	0.8356	0.002951	0.925	0.001552	0.1397	0.531	0.3679	0.137	intergenic	CD300LG;MPP2	41951277	T	C	0.920527	1	0.928	0.9251	0.8439	0.9059
17	43877234	rs231522	0.8356	0.002951	0.925	0.001552	0.1397	0.531	0.3679	0.137	UTR3	MPP2	41954602	A	G	0.920927	1	0.9294	0.9251	0.8439	0.907
17	43882440	rs124721	0.8356	0.002951	0.925	0.001552	0.1397	0.531	0.3679	0.137	exonic	MPP2	41959808	T	C	0.920927	1	0.9294	0.9251	0.8439	0.907
17	43882773	rs124720	0.8356	0.002951	0.925	0.001552	0.1397	0.531	0.3679	0.137	intronic	MPP2	41960141	A	G	0.920927	1	0.9294	0.9251	0.8439	0.907
17	43884804	rs35901677	0.8356	0.002951	0.925	0.001552	0.1397	0.531	0.3679	0.137	intronic	MPP2	41962172	A	AT	0.920727	1	0.9294	0.9251	0.8439	0.9059
17	43888967	rs231511	0.8356	0.002951	0.925	0.001552	0.1397	0.531	0.3679	0.137	intronic	MPP2	41966335	T	C	0.920527	1	0.928	0.9251	0.8439	0.9059
9	19131933	rs7854328	-0.6137	0.00296	-0.5874	0.006684	-0.309	0.0271	-0.6612	0.0001305	intergenic	PLIN2;DENND4C	19131931	A	G	0.319289	0.3075	0.3372	0.1747	0.4225	0.408
18	10507146	rs670749	0.5945	0.00297	0.5772	0.005804	0.647	8.17e-06	0.7048	3.23e-05	intergenic	APCDD1;NAPG	10507143	A	G	0.613419	0.6677	0.5144	0.8805	0.4334	0.4519
15	72836052	rs117432728	1.927	0.002972	2.158	0.001164	0.5531	0.2921	0.9874	0.07786	intergenic	ADPGK-AS1;NEO1	73128393	A	G	0.0227636	0.0952	0.0231	0.0008	0	0.001
5	2337079	rs13169221	0.5853	0.002976	0.5798	0.004553	0.4047	0.004346	0.6053	0.0002001	intergenic	C100506858;LSIN	2337193	C	T	0.328674	0.2173	0.3401	0.2458	0.4284	0.4448
6	52268721	rs17240175	-1.847	0.002983	-2.076	0.005139	-1.515	4.54e-05	-1.228	0.002421	intronic	MCM3	52133519	A	G	0.0263578	0	0.0274	0.0023	0.0616	0.0491
5	2349525	rs4866634	0.6027	0.002991	0.6047	0.003869	0.4612	0.001697	0.659	8.64e-05	intergenic	C100506858;LSIN	2349639	A	T	0.25619	0.1994	0.3069	0.0537	0.4105	0.3937
13	101594489	rs9557710	0.5676	0.002998	0.5281	0.00876	0.3669	0.007412	0.657	6.06e-05	intronic	ITGBL1	102246839	C	T	0.542532	0.6726	0.5259	0.2927	0.6978	0.5982
7	43501288	rs17339479	0.7205	0.003003	0.7156	0.004809	0.7832	4.18e-05	0.6419	0.00333	exonic	HECW1	43540887	A	C	0.116414	0.0079	0.1499	0.0696	0.2068	0.1748
6	15280025	rs11754102	0.5845	0.00301	0.6441	0.001841	0.5369	0.0001764	0.6419	9.97e-05	intronic	JARID2	15280256	G	A	0.253594	0.3145	0.3631	0.0756	0.3181	0.2873
17	4454478	rs12601416	-0.6311	0.003011	-0.8439	0.0002748	-0.3245	0.03199	-0.6198	0.0004827	intronic	SPNS3	4357773	A	G	0.515375	0.2371	0.6744	0.4274	0.5974	0.7239
5	100391922	rs62389062	0.7372	0.003016	0.7162	0.006021	0.7592	3.39e-05	0.8803	3.12e-05	intergenic	100133050;FAM1	99727626	T	C	0.398562	0.6905	0.3588	0.0787	0.3648	0.593
7	9268993	rs10229614	-0.9954	0.003017	-1.045	0.004264	-0.9488	2.02e-05	-0.8568	0.0008584	intergenic	NXPH1;PER3P1	9308623	C	A	0.167332	0.2232	0.1931	0.211	0.1093	0.092
1	13681063	rs10927929	-0.6874	0.003018	-0.7039	0.004105	-0.5834	0.000316	-0.7927	5.24e-05	intergenic	PDPN;PRDM2	14007558	T	C	0.730431	0.754	0.7061	0.7973	0.7396	0.6237
13	101595621	rs9554809	0.5664	0.003022	0.5274	0.008753	0.3589	0.008748	0.6379	9.15e-05	intronic	ITGBL1	102247971	G	A	0.543331	0.6726	0.5274	0.2943	0.6968	0.6002
1	13684159	rs10803423	-0.6851	0.003025	-0.6921	0.004537	-0.6009	0.000216	-0.8044	4.18e-05	intergenic	PDPN;PRDM2	14010654	C	T	0.726637	0.753	0.7061	0.7958	0.7306	0.6166
1	13684323	rs149154504	-0.6851	0.003025	-0.6921	0.004537	-0.6009	0.000216	-0.8044	4.18e-05	intergenic	PDPN;PRDM2	14010818	AAAAT	A	0.727636	0.751	0.7046	0.7965	0.7316	0.6227
1	13684356	rs10803424	-0.6851	0.003025	-0.6921	0.004537	-0.6009	0.000216	-0.8044	4.18e-05	intergenic	PDPN;PRDM2	14010851	A	C	0.726637	0.753	0.7075	0.7958	0.7306	0.6155
1	13689395	rs35118327	-0.6851	0.003025	-0.6921	0.004537	-0.6176	0.0001499	-0.8044	4.18e-05	intergenic	PDPN;PRDM2	14015890	G	A	0.726438	0.7579	0.7075	0.792	0.7296	0.6155
1	13691935	rs4661439	-0.6851	0.003025	-0.6921	0.004537	-0.6176	0.0001499	-0.8044	4.18e-05	intergenic	PDPN;PRDM2	14018430	C	A	0.727436	0.7579	0.7075	0.795	0.7296	0.6166
12	113737048	rs73203080	-0.9341	0.003035	-1.247	0.0006554	-0.8969	7.77e-05	-0.9574	0.0003977	intergenic	HX5-AS1;LINC012	114174853	G	A	0.0952476	0.002	0.1081	0.0076	0.2137	0.1789
1	13697373	rs10803425	-0.6888	0.003037	-0.6859	0.005401	-0.6247	0.0001623	-0.7977	5.61e-05	intergenic	PDPN;PRDM2	14023868	T	C	0.760982	0.7599	0.7147	0.8116	0.7505	0.7372
7	32755541	rs61472961	0.6956	0.003042	0.7354	0.00302	0.6687	9.25e-05	0.7749	8.78e-05	intergenic	IR550A2;LINC009	32795153	G	A	0.221046	0.1399	0.1643	0.2443	0.1779	0.3579
1	13684102	rs10754900	-0.6987	0.003048	-0.7243	0.003818	-0.6506	0.0001116	-0.8054	5.77e-05	intergenic	PDPN;PRDM2	14010597	A	G	0.804113	0.7768	0.7233	0.9327	0.7535	0.7679
1	75383889	rs7522348	-0.6334	0.003061	-0.7936	0.0004698	-0.3573	0.02213	-0.6305	0.0004337	intronic	SLC44A5	75849574	G	C	0.616014	0.9563	0.3818	0.7481	0.4215	0.453
4	130568157	rs7681380	-0.714	0.003069	-0.7811	0.002573	-0.493	0.003674	-0.8005	0.0001173	intergenic	INC02479;SNHG2	131489312	A	C	0.16234	0.0317	0.1916	0.0499	0.3976	0.1861
10	112829271	rs28561703	-0.6106	0.003077	-0.6793	0.001992	-0.4187	0.00298	-0.7172	3.4e-05	intronic	VTI1A	114589030	C	T	0.605232	0.5546	0.4914	0.5681	0.663	0.729
2	53566244	rs7588861	0.6551	0.003081	0.8334	0.0003628	0.3589	0.0289	0.5951	0.001653	intergenic	MIR4431;ASB3	53793381	T	G	0.293331	0.4683	0.17	0.3003	0.167	0.3211
7	43500914	rs78224160	0.7289	0.003096	0.6945	0.007288	0.7992	4.79e-05	0.6569	0.003094	intronic	HECW1	43540513	A	G	0.0992412	0.0079	0.1441	0.0076	0.2068	0.1748
8	72024847	rs41504846	0.7738	0.003107	0.9964	0.000257	0.4775	0.01947	0.5008	0.02824	ncRNA_intronic	MSC-AS1	72937082	T	G	0.172324	0.1647	0.2968	0.1301	0.0775	0.2464
10	59079861	rs73306371	1.163	0.003108	1.314	0.001122	1.141	0.0001588	1.341	7.73e-05	intergenic	LINC00844;CCEPI	60839621	C	T	0.0844649	0.1528	0.0447	0.1059	0.0169	0.0828
6	52255045	rs79609001	-1.837	0.003116	-2.062	0.005426	-1.62	2.77e-05	-1.361	0.001437	intergenic	IL17F;MCM3	52119843	C	T	0.0265575	0.001	0.0245	0.0068	0.0567	0.0501
6	52259784	rs12205964	-1.837	0.003116	-2.062	0.005426	-1.62	2.77e-05	-1.361	0.001437	intergenic	IL17F;MCM3	52124582	A	G	0.0265575	0	0.0245	0.0068	0.0577	0.0501
4	70541437	rs28408923	0.6383	0.003119	0.6026	0.007806	0.638	4.47e-05	0.683	0.0001041	intergenic	AMTN;AMBN	71407154	T	C	0.715855	0.5843	0.745	0.7874	0.7515	0.6973
8	102720511	rs12677521	-0.9491	0.003141	-0.918	0.006512	-0.9715	2.2e-05	-0.797	0.001689	intergenic	C101927245;GAS	103732739	T	C	0.0872604	0.0982	0.2752	0.0068	0.1014	0.0368
4	70551504	rs138639165	0.6419	0.003148	0.604	0.0083	0.6941	7.1e-06	0.6766	0.0001508	intergenic	AMTN;AMBN	71417221	T	C	0.719848	0.5942	0.7392	0.8147	0.7406	0.6861
20	29762132	rs1386627332	0.8029	0.003148	0.7025	0.01308	0.8006	3.37e-05	0.6121	0.005683	intergenic	AM242B;MIR447	NA	NA	NA	NA	NA	NA	NA	NA	NA
22	25012268	rs141922405	-0.7627	0.003151	-0.7757	0.004514	-0.387	0.01572	-0.7466	0.000314	intergenic	MEM211;KIAA16	25408235	TTTC	T	0.266973	0.4722	0.2983	0.1581	0.2147	0.2342
2	188231178	rs4371324	1.113	0.003164	1.295	0.0007608	0.8349	0.002273	1.284	3.11e-05	ncRNA_intronic	LINC01090	189095905	C	T	0.11222	0.2173	0.0274	0.0408	0.0487	0.226
5	118269270	rs1947803	0.6755	0.003172	0.795	0.0007953	0.182	0.2975	0.3807	0.05451	intergenic	NC02147;LINC021	117604965	A	G	0.233826	0.0248	0.1844	0.4818	0.2326	0.1503
5	118272182	rs4555864	0.6755	0.003172	0.795	0.0007953	0.182	0.2975	0.3807	0.05451	intergenic	NC02147;LINC021	117607877	T	C	0.233027	0.0248	0.1859	0.4803	0.2326	0.1472
11	95044250	rs11021024	0.5709	0.003196	0.6045	0.00325	0.5539	8.51e-05	0.5532	0.000603	intergenic	KDM4E;SRSF8	94777414	A	C	0.495607	0.7937	0.451	0.1846	0.5408	0.5941
4	70549106	rs28801727	0.6365	0.003203	0.5988	0.008308	0.6894	7e-06	0.6714	0.0001359	intergenic	AMTN;AMBN	71414823	C	T	0.719249	0.5923	0.7378	0.8147	0.7406	0.6861
6	52239066	rs11465551	-1.833	0.003211	-2.062	0.005426	-1.494	5.55e-05	-1.2	0.00297	intronic	IL17F	52103864	T	C	0.0279553	0	0.0245	0.0129	0.0577	0.0491
6	52259067	rs12190957	-1.833	0.003211	-2.062	0.005426	-1.618	2.84e-05	-1.358	0.001475	intergenic	IL17F;MCM3	52123865	G	C	0.0263578	0	0.0245	0.0068	0.0567	0.0501
6	52259769	rs71820026	-1.833	0.003211	-2.062	0.005426	-1.619	2.84e-05	-1.358	0.001472	intergenic	IL17F;MCM3	52124567	AAAAG	A	0.0263578	0	0.0245	0.0068	0.0567	0.0501
1	13680998	rs10803422	-0.6802	0.003212	-0.6614	0.006691	-0.5903	0.0002925	-0.7894	5.86e-05	intergenic	PDPN;PRDM2	14007493	G	A	0.748203	0.754	0.7118	0.8707	0.7306	0.6207
7	43485728	rs75870777	0.7222	0.003215	0.7332	0.004293	0.7455	7.75e-05	0.6349	0.003468	intronic	HECW1	43525327	G	C	0.128594	0.0079	0.1628	0.0756	0.2187	0.2076
19	54593218	rs1280747808	1.323	0.003228	1.382	0.002574	0.8631	0.02123	1.392	0.0004099	upstream	LILRA1	NA	NA	NA	NA	NA	NA	NA	NA	NA
6	52278541	rs17246338	-1.829	0.003246	-2.054	0.005573	-1.498	5.18e-05	-1.217	0.002581	intronic	MCM3	52143339	TATTAAA	T	0.0375399	0	0.0346	0.0408	0.0616	0.0491
6	52281298	rs12214090	-1.829	0.003246	-2.054	0.005573	-1.498	5.18e-05	-1.217	0.002581	intronic	MCM3	52146096	G	A	0.0379393	0	0.036	0.0408	0.0616	0.0501
13	101611901	rs9557712	0.5613	0.003252	0.5169	0.01005	0.3734	0.006505	0.6543	6.28e-05	intronic	ITGBL1	102264251	G	A	0.548722	0.6855	0.5274	0.3056	0.6958	0.6002
7	57977211	rs1380171254	1.77	0.003264	1.905	0.001696	1.858	0.0003292	2.163	5.27e-05	intergenic	ZNF716;NONE	NA	NA	NA	NA	NA	NA	NA	NA	NA
12	107680220	rs73187387	0.9769	0.003302	0.9364	0.006309	0.8332	0.001503	1.134	0.0001028	intergenic	BTBD11;PWP1	108073997	G	T	0.0998403	0.0823	0.0576	0.1641	0.0606	0.1012
2	188243840	rs34477786	1.108	0.00331	1.29	0.0007995	0.832	0.00236	1.281	3.27e-05	ncRNA_intronic	LINC01090	189108567	A	G	0.11222	0.2173	0.0274	0.0408	0.0487	0.226
1	125149178	rs1340686695	-1.003	0.00332	-1.01	0.005487	-0.838	0.0001838	-1.137	6.26e-05	intergenic	NONE;NONE	NA	NA	NA	NA	NA	NA	NA	NA	NA
22	16408844	rs199636598	0.8681	0.003323	1.121	0.0006715	0.9325	7.8e-06	1.013	4.57e-05	intergenic	DUXAP8;CCT8L2	NA	NA	NA	NA	NA	NA	NA	NA	NA
12	98005593	rs140719341	3.154	0.003327	3.378	0.00172	2.349	0.02701	2.682	0.01232	intergenic	MIR4303;SLC9A7F	98399371	C	T	0.00479233	0	0.0159	0	0.0129	0
8	102721590	rs9642794	-0.9564	0.00333	-0.9348	0.006366	-0.9642	2.83e-05	-0.8638	0.001013	intergenic	C101927245;GAS	103733818	A	G	0.110623	0.0952	0.2752	0.1029	0.0924	0.0389
8	102721794	rs9642981	-0.9564	0.00333	-0.9348	0.006366	-0.9642	2.83e-05	-0.8638	0.001013	intergenic	C101927245;GAS	103734022	G	C	0.111022	0.0952	0.2752	0.1044	0.0924	0.0389
8	102721878	rs9642982	-0.9564	0.00333	-0.9348	0.006366	-0.9642	2.83e-05	-0.8638	0.001013	intergenic	C101927245;GAS	103734106	T	C	0.111422	0.0952	0.2752	0.1051	0.0924	0.0399
10	16518536	rs6602131	0.9195	0.003331	0.9557	0.003471	0.897	9.33e-05	0.881	0.0007893	intronic	C1QL3	16560535	C	T	0.170527	0.2569	0.0735	0.2481	0.0755	0.1431
7	43496166	rs10487676	0.7201	0.003342	0.6943	0.007122	0.7677	6.88e-05	0.6556	0.002691	intronic	HECW1	43535765	G	A	0.10643	0.0079	0.1542	0.0091	0.2127	0.1963
18	10487046	rs622760	0.5849	0.003342	0.5602	0.006765	0.6149	1.71e-05	0.6646	7.95e-05	intronic	APCDD1	10487043	A	G	0.522963	0.6488	0.5432	0.5386	0.4602	0.4223
2	188245122	rs11676580	1.1	0.003345	1.251	0.00106	0.8091	0.002159	1.287	2.22e-05	ncRNA_intronic	LINC01090	189109849	A	G	0.190296	0.2192	0.0461	0.323	0.0497	0.228
1	13686686	rs4662016	-0.6713	0.003347	-0.6471	0.00736	-0.5909	0.0002669	-0.7881	5.4e-05	intergenic	PDPN;PRDM2	14013181	G	T	0.727037	0.752	0.7075	0.7965	0.7306	0.6176
1	114350228	rs9308265	-0.6711	0.003349	-0.8145	0.001096	-0.3666	0.01269	-0.6996	0.0002203	intergenic	SYT6;TRIM33	114892850	A	G	0.466653	0.5169	0.3386	0.6604	0.2545	0.4622
1	75375979	rs4554668	-0.6267	0.003351	-0.7871	0.0005393	-0.3632	0.02044	-0.6227	0.0005159	intronic	SLC44A5	75841664	T	C	0.619609	0.9544	0.3847	0.767	0.4155	0.4519
5	109166590	rs73779047	0.8052	0.003363	0.8601	0.002316	0.7087	0.0005649	0.923	6.22e-05	intronic	FER	108502291	A	G	0.139177	0.0704	0.111	0.1558	0.2107	0.1339
1	13692691	rs4662018	-0.6767	0.003371	-0.6841	0.004975	-0.6068	0.0001955	-0.7865	6.14e-05	intergenic	PDPN;PRDM2	14019186	C	T	0.727436	0.7579	0.7075	0.795	0.7306	0.6155
8	102724501	rs62525076	-0.9637	0.003371	-0.9451	0.006158	-1.006	2.05e-05	-0.864	0.001097	intergenic	C101927245;GAS	103736729	T	A	0.0846645	0.0982	0.2709	0.0053	0.0915	0.0389
17	43871066	rs231528	0.8122	0.003372	0.9043	0.001681	0.1269	0.5638	0.3554	0.1441	intergenic	CD300LG;MPP2	41948434	T	C	0.917931	1	0.9236	0.9251	0.835	0.9049
10	4389712	rs72766495	1.325	0.003377	1.525	0.0007424	0.8002	0.03684	1.153	0.004804	ncRNA_intronic	LINC00703	4431904	G	T	0.0213658	0	0.0418	0.0023	0.0666	0.0082
1	75393107	rs1249818	-0.6271	0.003388	-0.8013	0.0004396	-0.326	0.03589	-0.6046	0.0007293	intronic	SLC44A5	75858792	G	A	0.622404	0.9563	0.3876	0.7496	0.4404	0.4601
4	70549979	rs28818830	0.6329	0.0034	0.5952	0.008768	0.6862	7.61e-06	0.6691	0.0001442	intergenic	AMTN;AMBN	71415696	T	C	0.719249	0.5923	0.7378	0.8147	0.7406	0.6861
4	70549989	rs28868615	0.6329	0.0034	0.5952	0.008768	0.6862	7.61e-06	0.6691	0.0001442	intergenic	AMTN;AMBN	71415706	G	A	0.719249	0.5923	0.7378	0.8147	0.7406	0.6861
4	70550162	rs28782495	0.6329	0.0034	0.5952	0.008768	0.6983	5.62e-06	0.6902	9.67e-05	intergenic	AMTN;AMBN	71415879	G	A	0.719249	0.5923	0.7378	0.8147	0.7406	0.6861
4	70550337	rs28826394	0.6329	0.0034	0.5952	0.008768	0.6862	7.61e-06	0.6691	0.0001442	intergenic	AMTN;AMBN	71416054	G	A	0.71865	0.5923	0.7349	0.8139	0.7406	0.6861
4	70550708	rs28827921	0.6329	0.0034	0.5952	0.008768	0.6862	7.61e-06	0.6691	0.0001442	intergenic	AMTN;AMBN	71416425	T	C	0.719249	0.5923	0.7378	0.8147	0.7406	0.6861
4	70551178	rs4277751	0.6329	0.0034	0.5952	0.008768	0.6862	7.61e-06	0.6691	0.0001442	intergenic	AMTN;AMBN	71416895	T	G	0.719848	0.5942	0.7392	0.8147	0.7406	0.6861
4	70551635	rs28840110	0.6329	0.0034	0.5952	0.008768	0.6862	7.61e-06	0.6691	0.0001442	intergenic	AMTN;AMBN	71417352	A	G	0.720048	0.5942	0.7392	0.8147	0.7406	0.6871
6	143710056	rs2488100	0.5993	0.003404	0.6851	0.001575	0.1941	0.1644	0.5375	0.0009995	intronic	PHACTR2	144031193	T	G	0.360623	0.3323	0.3674	0.1641	0.5636	0.4417
7	62347973	rs78954521	-1.018	0.003412	-1.375	0.000844	-1.041	1.35e-05	-1.148	9.19e-05	intergenic	NONE;ZNF733P	61792224	G	A	0.000199681	0	0	0	0	0.001
6	52270751	rs17246492	-1.821	0.003426	-2.043	0.005902	-1.493	5.9e-05	-1.205	0.002945	intronic	MCM3	52135549	G	C	0.0263578	0	0.0274	0.0023	0.0616	0.0491
5	2337883	rs13183775	0.5805	0.003433	0.6076	0.00316	0.4211	0.003523	0.6388	0.0001075	intergenic	C100506858;LSIN	2337997	C	T	0.261981	0.2024	0.3084	0.0537	0.4195	0.41

7	43499179	rs111615751	0.7218	0.003435	0.7227	0.005174	0.7887	5.62e-05	0.6387	0.003902	intronic	HECW1	43538778	G	T	0.103834	0.0079	0.1542	0.0083	0.2137	0.183
13	85229762	rs9575840	0.8018	0.003445	0.9937	0.0003734	0.3371	0.1246	0.5267	0.02921	intergenic	NC00375;LINC003	85803897	C	T	0.117412	0.0645	0.0591	0.2163	0.0676	0.1309
13	101568514	rs9554806	-0.6521	0.003463	-0.6946	0.003827	-0.3552	0.01731	-0.7026	0.0001979	intronic	ITGBL1	102220865	G	A	0.279153	0.4335	0.3141	0.0598	0.3002	0.3701
13	101569435	rs9554807	-0.6521	0.003463	-0.6946	0.003827	-0.3549	0.01736	-0.7047	0.0001933	intronic	ITGBL1	102221786	C	T	0.283946	0.4375	0.3141	0.0598	0.3022	0.3885
8	102730993	rs62525077	-0.9531	0.003482	-0.9276	0.00687	-0.9789	2.67e-05	-0.869	0.0009777	intergenic	C101927245;GAS	103743221	G	A	0.0866613	0.1081	0.2723	0.0045	0.0915	0.0389
4	40267603	rs139609934	1.693	0.003491	1.856	0.001306	0.6803	0.2172	1.141	0.04007	intergenic	RHOH;LINC0226	40269223	G	A	0.00778754	0	0.0115	0.0008	0.0209	0.0092
8	102722472	rs111477364	-0.9526	0.003508	-0.9268	0.006931	-1.004	1.93e-05	-0.8687	0.0009809	intergenic	C101927245;GAS	103734700	A	G	0.0846645	0.0982	0.2709	0.0053	0.0915	0.0389
6	52260473	rs12194284	-1.817	0.003525	-2.043	0.005902	-1.492	6.08e-05	-1.202	0.003018	intergenic	IL17F;MCM3	52125271	C	T	0.0273562	0	0.0245	0.0068	0.0616	0.0501
6	52263123	rs17240301	-1.817	0.003525	-2.043	0.005902	-1.492	6.08e-05	-1.202	0.003018	downstream	MCM3	52127921	G	A	0.0265575	0	0.0274	0.003	0.0616	0.0491
6	52271028	rs17246471	-1.817	0.003525	-2.043	0.005902	-1.492	6.08e-05	-1.202	0.003018	intronic	MCM3	52135826	C	G	0.0263578	0	0.0274	0.0023	0.0616	0.0491
6	52279423	rs2307331	-1.814	0.003555	-2.033	0.006158	-1.466	8.13e-05	-1.194	0.003218	exonic	MCM3	52144221	C	T	0.0263578	0	0.0274	0.0023	0.0616	0.0491
1	13689432	rs56248481	-0.678	0.003566	-0.6996	0.004559	-0.6318	0.0001372	-0.7981	5.55e-05	intergenic	PDPN;PRDM2	14015927	C	T	0.768371	0.7798	0.7089	0.8041	0.7515	0.7679
8	102724343	rs62525075	-0.951	0.003573	-0.9256	0.007026	-0.9962	2.24e-05	-0.8579	0.001132	intergenic	C101927245;GAS	103736571	G	A	0.0846645	0.0982	0.2709	0.0053	0.0915	0.0389
8	102733316	rs75291442	-0.951	0.003573	-0.9256	0.007026	-1.039	1.24e-05	-0.8661	0.001019	intergenic	C101927245;GAS	103745544	C	T	0.0832668	0.0982	0.2695	0.0045	0.0865	0.0389
8	102733347	rs79738137	-0.951	0.003573	-0.9256	0.007026	-1.039	1.24e-05	-0.8661	0.001019	intergenic	C101927245;GAS	103745575	C	T	0.0832668	0.0982	0.2695	0.0045	0.0865	0.0389
1	75373460	rs1249808	-0.6243	0.003588	-0.7857	0.0005519	-0.3603	0.02154	-0.6207	0.0005442	intronic	SLC44A5	75839145	T	C	0.621406	0.9563	0.3862	0.7655	0.4225	0.453
1	75375249	rs77754220	-0.6243	0.003588	-0.7857	0.0005519	-0.3603	0.02154	-0.6207	0.0005442	intronic	SLC44A5	75840934	A	G	0.622005	0.9563	0.3862	0.7678	0.4225	0.453
1	75375895	rs80030530	-0.6243	0.003588	-0.7857	0.0005519	-0.3603	0.02154	-0.6207	0.0005442	intronic	SLC44A5	75841580	T	C	0.622005	0.9563	0.3862	0.7678	0.4225	0.453
1	75376093	rs1313031	-0.6243	0.003588	-0.7857	0.0005519	-0.3603	0.02154	-0.6207	0.0005442	intronic	SLC44A5	75841778	T	C	0.621805	0.9563	0.3862	0.7678	0.4225	0.4519
2	78920802	rs72814617	0.7563	0.00359	0.9146	0.0006119	0.1679	0.4065	0.3501	0.1207	intergenic	C105374820;REG	79147928	G	A	0.133586	0.1319	0.1023	0.146	0.0875	0.1881
6	52211640	rs12214740	-0.9196	0.003592	-0.9196	0.005974	-0.8598	7.97e-05	-0.6936	0.00424	intergenic	IL17A;IL17F	52076438	C	T	0.103435	0.0427	0.0836	0.0734	0.1551	0.1677
6	52253596	rs75748419	-1.599	0.003593	-1.718	0.005491	-1.459	3.99e-05	-1.276	0.001545	intergenic	IL17F;MCM3	52118394	C	T	0.0587061	0	0.0303	0.121	0.0586	0.0552
17	43884083	rs231518	0.814	0.003617	0.925	0.001552	0.1324	0.5523	0.3571	0.1484	splicing	MPP2	41961451	T	C	0.921126	1	0.9294	0.9251	0.8439	0.908
17	43887832	rs231513	0.814	0.003617	0.925	0.001552	0.1324	0.5523	0.3571	0.1484	intronic	MPP2	41965200	C	G	0.919928	1	0.928	0.9244	0.8429	0.9049
15	28711769	rs57230055	-1.107	0.003626	-1.148	0.004021	-0.7181	0.001087	-1.137	9.97e-05	ncRNA_intronic	LOC101928042	28956915	T	C	0.177716	0.5149	0.2421	0.0363	0.0249	0.1329
10	112792508	rs10787461	-0.5866	0.003627	-0.6917	0.001308	-0.4576	0.001327	-0.691	5.44e-05	intronic	VTI1A	114552267	A	G	0.520966	0.5635	0.4697	0.3048	0.6213	0.7025
4	9501015	rs144435848	1.82	0.003649	1.515	0.02087	0.7648	0.05849	1.639	0.0007205	intergenic	EFB131A;MIR548	9502659	T	C	0.0543131	0.0655	0.0159	0.1029	0	0.0603
3	118044935	rs117026268	2.462	0.003655	2.734	0.001645	0.5814	0.447	1.332	0.08683	intergenic	02024;LOC10537	117763782	C	A	0.0061901	0.0308	0	0	0	0
6	52274621	rs10456156	-1.809	0.003667	-2.033	0.006158	-1.463	8.42e-05	-1.191	0.00331	intronic	MCM3	52139419	C	T	0.0353435	0	0.0346	0.0325	0.0616	0.0491
6	166202164	rs4709104	-1.05	0.0037	-1.399	0.001196	-0.9608	6.85e-05	-1.215	0.0001261	intergenic	DAT;LOC101929	166615652	C	T	0.151358	0.1181	0.2003	0.1876	0.0915	0.1636
6	166202480	rs35299243	-1.05	0.0037	-1.399	0.001196	-0.9654	6.35e-05	-1.222	0.0001147	intergenic	DAT;LOC101929	166615968	A	T	0.149161	0.121	0.1988	0.1778	0.0915	0.1636
7	43497285	rs76922785	0.7154	0.003701	0.7136	0.00567	0.7891	5.01e-05	0.6631	0.002511	intronic	HECW1	43536884	G	A	0.10643	0.0079	0.1556	0.0083	0.2127	0.1963
18	10491284	rs653575	-0.5757	0.003701	-0.5307	0.009772	-0.5608	7.45e-05	-0.6811	4.75e-05	intergenic	APCDD1;NAPG	10491281	A	G	0.522963	0.6478	0.4986	0.5643	0.4334	0.4479
18	10491307	rs529749	-0.5757	0.003701	-0.5307	0.009772	-0.5608	7.45e-05	-0.6811	4.75e-05	intergenic	APCDD1;NAPG	10491304	G	C	0.522963	0.6478	0.4986	0.5635	0.4344	0.4479
3	102427330	rs76612923	1.512	0.003702	1.728	0.001149	0.6442	0.1371	1.004	0.02998	intergenic	C101929411;ZPL	102146174	T	C	0.0207668	0.0417	0.0821	0.0008	0.001	0.0031
6	143784266	rs7742964	0.5543	0.003724	0.6413	0.001507	0.07615	0.5908	0.1471	0.3627	intronic	PHACTR2	144105403	C	T	0.245008	0.3095	0.3012	0.1074	0.2863	0.2822
4	82480792	rs56708731	1.294	0.003726	1.186	0.01078	1.382	6.81e-05	1.611	2.45e-05	intergenic	NOPH1;TMEM15	83401945	A	G	0.0780751	0.0536	0.2795	0.0151	0.0189	0.1063
11	95043361	rs34389030	0.5647	0.003734	0.5978	0.003704	0.5538	8.5e-05	0.546	0.0007256	intergenic	KDM4E;SRSF8	94776525	G	C	0.494209	0.7937	0.4496	0.1838	0.5378	0.592
5	118276745	rs77500281	0.6622	0.003739	0.8022	0.0007286	0.2108	0.2346	0.3798	0.05825	intergenic	NC02147;LINC021	117612440	C	CA	0.202077	0.0248	0.1873	0.3601	0.2336	0.1493
13	45668495	rs144161815	2.079	0.003743	2.042	0.005821	1.865	0.00469	2.341	0.0004359	intergenic	RICH6B;LINC010	46242630	A	G	0.0125799	0	0.0173	0.0015	0.0298	0.0194
19	15510522	rs2060262	0.5864	0.003755	0.6982	0.00105	0.07731	0.6073	0.3216	0.06116	intronic	CYP4F22	15621333	A	G	0.241414	0.0734	0.2406	0.4009	0.337	0.1012
4	82479736	rs58183119	1.29	0.0038	1.182	0.01094	1.351	9.56e-05	1.557	4.9e-05	intergenic	NOPH1;TMEM15	83400889	G	A	0.0822684	0.0546	0.2867	0.0242	0.0229	0.1053
2	188224270	rs72901564	1.088	0.003808	1.295	0.0007608	0.8277	0.002459	1.273	3.57e-05	ncRNA_intronic	LINC01090	189088997	T	C	0.113019	0.2173	0.0274	0.0424	0.0487	0.228
2	188246268	rs72903517	1.088	0.003808	1.295	0.0007608	0.8277	0.002459	1.273	3.57e-05	ncRNA_intronic	LINC01090	189110995	A	C	0.11222	0.2173	0.0274	0.0408	0.0487	0.226
7	142463122	rs769051792	0.7911	0.003814	0.808	0.004848	0.5343	0.005801	0.8439	0.0002106	intronic	TCAF2	NA	NA	NA	NA	NA	NA	NA	NA	NA
18	10488742	rs505893	-0.5724	0.003852	-0.5339	0.00945	-0.6039	2.42e-05	-0.6894	4.08e-05	UTR3	APCDD1	10488739	G	C	0.530152	0.6498	0.5043	0.6157	0.4334	0.409
18	10488784	rs35454872	-0.5724	0.003852	-0.5339	0.00945	-0.5982	3e-05	-0.6894	4.08e-05	UTR3	APCDD1	10488781	T	TA	0.530751	0.6498	0.5043	0.618	0.4334	0.409
18	10489016	rs593078	-0.5724	0.003852	-0.5339	0.00945	-0.6039	2.42e-05	-0.6894	4.08e-05	UTR3	APCDD1	10489013	A	G	0.530551	0.6478	0.5043	0.6188	0.4334	0.409
7	43509958	rs10253986	0.644	0.003853	0.6372	0.006082	0.6751	8.12e-05	0.4762	0.01577	ncRNA_exonic	LUARIS	43549557	A	G	0.183906	0.0863	0.1671	0.1657	0.2147	0.2894
23	100745511	rs5920848	-0.5406	0.003854	-0.6014	0.002808	-0.01725	0.8865	-0.1801	0.2054	intergenic	SYTL4;CSTF2	NA	NA	NA	NA	NA	NA	NA	NA	NA
4	70549910	rs28881884	0.6249	0.003861	0.5874	0.009777	0.6938	6.47e-06	0.6837	0.0001136	intergenic	AMTN;AMBN	71415627	T	C	0.719249	0.5923	0.7378	0.8147	0.7406	0.6861
4	70550175	rs28750399	0.6249	0.003861	0.5874	0.009777	0.6938	6.47e-06	0.6837	0.0001136	intergenic	AMTN;AMBN	71415892	A	G	0.719249	0.5923	0.7378	0.8147	0.7406	0.6861
15	89316763	rs3087374	-1.786	0.003874	-1.632	0.008366	-1.451	8.45e-05	-1.521	0.0008305	exonic	POLG	89859994	C	A	0.0269569	0	0.0403	0.0053	0.0855	0.0143
7	58116118	rs1222632174	-1.04	0.003896	-0.9687	0.009782	-0.968	5.42e-05	-1.149	0.0001301	intergenic	ZNF716;NONE	NA	NA	NA	NA	NA	NA	NA	NA	NA
13	101615074	rs7139608	0.5442	0.003906	0.5014	0.01155	0.3472	0.01067	0.6171	0.0001331	intronic	ITGBL1	102267424	C	A	0.565096	0.6845	0.5317	0.3646	0.6948	0.6033
10	112795376	rs34562081	-0.6784	0.003912	-0.8257	0.001425	-0.5527	0.0006195	-0.8881	1.36e-05	intronic	VTI1A	114555135	A	T	0.169529	0.25	0.2161	0.0484	0.2336	0.1513
19	1698640	rs59725842	-0.8245	0.003935	-0.8919	0.003591	-0.6658	0.0005303	-1.01	5.22e-05	intergenic	TCF3;ONECUT3	1698639	G	A	0.1877	0.0486	0.1354	0.3275	0.1918	0.1748
3	195598669	rs13303294	-0.5758	0.003944	-0.6623	0.0022	-0.2546	0.06244	-0.5645	0.000562	intergenic	POD;LOC1053742	195325540	C	G	0.4377	0.7163	0.4265	0.3722	0.3907	0.2955
13	101553989	rs9557700	-0.6387	0.003948	-0.5985	0.01004	-0.3408	0.02083	-0.6921	0.0002254	intronic	ITGBL1	102206340	G	A	0.294928	0.4345	0.3156	0.1006	0.3032	0.3906
6	166202801	rs34512108	-1.04	0.003958	-1.369	0.001462	-0.9527	7.9e-05	-1.208	0.0001361	intergenic	DAT;LOC101929	166616289	T	C	0.148962	0.12	0.1988	0.177	0.0915	0.1646
18	10492054	rs11367178	-0.5675	0.00396	-0.5246	0.0101	-0.535	0.0001414	-0.6566	7.76e-05	intergenic	APCDD1;NAPG	10492051	TG	T	0.520168	0.6478	0.4899	0.5605	0.4304	0.4479
2	53565455	rs12053563	0.6339	0.003976	0.8334	0.0003628	0.352	0.0319	0.5853	0.001929	intergenic	MIR4431;ASB3	53792592	A	G	0.293131	0.4683	0.1729	0.3026	0.167	0.3149
5	2352616	rs10036054	0.5724	0.003983	0.5664	0.005991	0.3884	0.006507	0.6001	0.0002416	intergenic	C100506858;LSIN	2352730	C	T	0.314896	0.2192	0.3242	0.2171	0.4205	0.4305
7	63014879	rs796389001	-0.9019	0.00399	-1.104	0.001592	-0.5906	0.004496	-1	0.000127	intergenic	NONE;ZNF733P	NA	NA	NA	NA	NA	NA	NA	NA	NA
17	21645626	rs112595202	-0.7699	0.003995	-0.8385	0.002784	-0.8023	4.11e-05	-0.8059	0.0003158	intergenic	LINC02693;KCNJ1	NA	NA	NA	NA	NA	NA	NA	NA	NA
13	101552483	rs885304	-0.6278	0.003997	-0.663	0.00467	-0.3243	0.02549	-0.6619	0.0003152	intronic	ITGBL1	102204834	C	T	0.315495	0.4415	0.3213	0.171	0.3032	0.3896
13	101615796	rs996507	-0.6738	0.004038	-0.6709	0.006703	-0.3722	0.01543	-0.8411	2.58e-05	intronic	ITGBL1	102268146	A	G	0.2498	0.4573	0.2421	0.0514	0.2674	0.2914
3	75759571	rs796819888	0.8448	0.004041	0.8065	0.008753	0.8155	6.98e-05	0.9046	0.0001915	intronic	ZNF717	NA	NA	NA	NA	NA	NA	NA	NA	NA
6	15280753	rs9396579	0.5623	0.004054	0.6208	0.002491	0.5347	0.0001788	0.6423	9.75e-05	intronic	JARID2	15280984	C	T	0.259984	0.3264	0.389	0.0756	0.3181	0.2894
1	13674378	rs1832803	-0.6618	0.004058	-0.6705	0.005834	-0.5797	0.0003428	-0.7792	6.7e-05	intergenic	PDPN;PRDM2	14000873	A	G	0.720248	0.754	0.7017	0.77	0.7306	0.6207
7	58116114	rs1293268665	-1.004	0.004066	-0.9213	0.01105	-0.9844	3.33e-05	-1.083	0.0001852	intergenic	ZNF716;NONE	NA	NA	NA	NA	NA	NA	NA	NA	NA
4	70544159	rs58722739	0.6204	0.004071	0.5828	0.01028	0.6747	1.04e-05	0.6574	0.0001851	intergenic	AMTN;AMBN	71409876	T	TTAA	0.713259	0.5923	0.7349	0.8109	0.7406	0.6626
10	112795381	rs36108730	-0.6782	0.004084	-0.8277	0.001451	-0.5097	0.001503	-0.8818	1.73e-05	intronic	VTI1A	114555140	T	C	0.170927	0.25	0.2161	0.0545	0.2326	0.1513
11	128745591	rs1238566	0.5618	0.004097	0.5922	0.004572	0.5344	0.0001851	0.6733	5.52e-05	intronic	FLI1	128615486	A	G	0.390974	0.3859	0.353	0.3396	0.4503	0.4315
22	16894877	rs199952169	-0.8474	0.004105	-0.8779	0.004291	-0.8674	9.07e-05	-1.038	3.16e-05	intergenic	HSFY1P1;GAB4	NA	NA	NA	NA	NA	NA	NA	NA	NA
13	101626430	rs74120603	-0.6645	0.004167	-0.6575	0.007244	-0.4173	0.007105	-0.865	1.45e-05	intronic	ITGBL1	102278780	A	T	0.252196	0.4702	0.2406	0.0514	0.2684	0.2904
13	85237000	rs61960253	0.7995	0.004171	0.9955	0.0004677	0.2522	0.2604	0.5237	0.03292	intergenic	NC00375;LINC003	85811135	T	C	0.0752796	0.0665	0.0461	0.062	0.0676	0.1309
7	43498698	rs1019971	0.7051	0.004183	0.7031	0.006371	0.7756	6.27e-05	0.6494	0.003002	intronic	HECW1	43538297	T	C	0.106829	0.0079	0.1542	0.0083	0.2137	0.1984
7	43499150	rs111475932	0.7051	0.004183	0.7031	0.006371	0.7706	7.89e-05	0.6269	0.004547	intronic	HECW1	43538749	G	A	0.103834	0.0079	0.1542	0.0083	0.2137	0.183
8	102741483	rs113663274	-0.9382	0.004198	-0.7928	0.01777	-0.9714	3.69e-05	-0.7802	0.002706	intergenic	C101927245;GAS	103753711	T	C	0.104633	0.1171	0.2752	0.0605	0.0944	0.0409
13	101597275	rs1583526	0.5438	0.004214	0.5281	0.00876	0.3469	0.01102	0.623	0.0001268	intronic	ITGBL1	102249625	G	T	0.54353	0.6726	0.5274	0.2965	0.6978	0.5971
4	130548361	rs4532248	-0.6979	0.004237	-0.7375	0.004516	-0.4591	0.007047	-0.8352	8.22e-05	intergenic	INC02479;SNHG2	131469516	T	A	0.179313	0.0288	0.1931	0.1195	0.3956	0.183
7	8303147	rs199719022	-1.483	0.004255	-1.314	0.01163	-1.989	4.73e-07	-1.666	0.0001567	ncRNA_intronic	LOC100505938	NA	NA	NA	NA	NA	NA	NA	NA	NA
3	195599681	rs4677701	-0.579	0.004269	-0.6905	0.00166	-0.2757	0.04598	-0.5906	0.0003841	intergenic	POD;LOC1053742	195326552	G	A	0.436901	0.7163	0.428	0.3737	0.3877	0.2914
18	10497939	rs501069	0.5737	0.00427	0.5578	0.007926	0.6208	1.83e-05	0.678	6.48e-05	intergenic	APCDD1;NAPG	10497936	A	G	0.609026	0.6478	0.5144	0.8775	0.4334	0.454
18	10498623	rs502237	0.5737	0.00427	0.5578	0.007926	0.6208	1.83e-05	0.678	6.48e-05	intergenic	APCDD1;NAPG	10498620	A	T	0.609026	0.6468	0.5144	0.8775	0.4334	0.455
1	179937570	rs1281386	0.6123	0.004275	0.568	0.01038	0.6388	7.42e-05	0.6325	0.0004865	intergenic	TOR1AIP1;CEP35	179906705	G	A	0.705072	0.8224	0.804	0.5083	0.7087	0.7761
4	189924720	rs111584967	-0.782	0.004289	-0.827	0.003874	-0.8399	2.27e-05	-0.799	0.0004213	ncRNA_intronic	FRG1-DT	NA	NA	NA	NA	NA	NA	NA	NA	NA
5	118270822	rs1382729	0.6503	0.004297	0.795	0.0007953	0.1741	0.3184	0.368	0.06257	intergenic	NC02147;LINC021	117606517	T	C	0.233027	0.0248	0.1859	0.4803	0.2326	0.1472
3	12256490	rs13078017	-0.6692	0.004332	-1.081	9.31e-05	-0.195	0.2143	-0.4644	0.01499	intergenic	SYN2;PPARG	12297989	T	C	0.254992	0.0466	0.2723	0.3457	0.4076	0.1779
12	98083066	rs7134234	0.5572	0.004333	0.6882	0.0008969	0.06632	0.6346	0.2193	0.1705	intergenic	MIR4303;SLC9A7F	98476844	T	A	0.617612	0.7639	0.6744	0.5053	0.504	0.6953
12	98104445	rs10777902	0.5477	0.00434	0.7132	0.0005241	0.03281	0.8114	0.1903	0.2268	intergenic	MIR4303;SLC9A7F	98498223	A	C	0.622005	0.7659	0.6628	0.5121	0.501	0.7178
9	10279606	rs1544056	0.5955	0.004369	0.572	0.009792	0.6276	7.77e-05	0.5546	0.002038	intronic	PTPRD	10279606	C	A	0.463458	0.3929	0.4625	0.674	0.1909	0.5327
8	40241765	rs28858171	-1.057	0.004406	-0.9047	0.0151	-0.9944	6.09e-05	-0.7615	0.006561	intergenic	TCIM;SIRLNT	40099284	A	G	0.119609	0.0268	0.0951	0.1188	0.1362	0.2168
6	166202424	rs4710026	-1.022	0.004408	-1.353	0.001488	-0.9659	5.78e-05	-1.206	0.0001272	intergenic	DAT;LOC101929	166615912	C	T	0.159744	0.119	0.2046	0.2163	0.0915	0.1636
17	507211	rs9748016	0.5747	0.004412	0.6304	0.002822	0.5151	0.0002648	0.6865	4.29e-05	intergenic	RFLNB;VPS53	410451	C	A	0.4373	0.502	0.4654	0.3147	0.4245	0.5297
13	45680614	rs113604798	2.02	0.004422	2.042	0.005821	1.859	0.004812	2.341	0.0004359	downstream	LINC01055	46254749	C	T	0.0119808	0	0.0173	0.0008	0.0298	0.0174
6	52247172	rs12216475	-1.771	0.004435	-1.993	0.007222	-1.567	5.3e-05	-1.303	0.002302	intergenic	IL17F;MCM3	52111970	C	T	0.0255591	0	0.0231	0.0068	0.0557	0.0481
4	70550426	rs56895716	0.6115	0.004437	0.5952	0.008768	0.6798	8.98e-06	0.6588	0.0001768	intergenic	AMTN;AMBN	71416143	TG	T	0.718251	0.5923	0.7363	0.8139	0.7376	0.6861

4	70550470	rs28889392	0.6115	0.004437	0.5952	0.008768	0.6798	8.98e-06	0.6588	0.0001768	intergenic	AMTN;AMBN	71416187	C	G	0.719249	0.5923	0.7378	0.8147	0.7406	0.6861
4	70552582	rs60421347	0.6115	0.004437	0.5952	0.008768	0.6798	8.98e-06	0.6588	0.0001768	intergenic	AMTN;AMBN	71418299	G	GC	0.719449	0.5933	0.7392	0.8147	0.7396	0.6861
4	70553471	rs4389630	0.6115	0.004437	0.5952	0.008768	0.6798	8.98e-06	0.6588	0.0001768	intergenic	AMTN;AMBN	71419188	A	G	0.719249	0.5923	0.7392	0.8147	0.7406	0.6851
7	43500333	rs113043572	0.7034	0.004441	0.6945	0.007288	0.7881	6.14e-05	0.6397	0.004036	intronic	HECW1	43539932	C	T	0.0992412	0.0079	0.1441	0.0076	0.2068	0.1748
10	38501942	rs1254097075	-1.488	0.004473	-1.337	0.01073	-1.292	6.28e-05	-1.732	0.000188	intergenic	NC00999;ACTR3B	NA	NA	NA	NA	NA	NA	NA	NA	NA
17	21835889	rs1231613951	-0.8404	0.00448	-0.8588	0.005212	-0.8708	9.3e-05	-0.8443	0.0007886	intergenic	KCNJ18;UBBP4	NA	NA	NA	NA	NA	NA	NA	NA	NA
13	85237774	rs9635065	0.7904	0.004485	0.9841	0.0005191	0.2671	0.2309	0.5179	0.03458	intergenic	NC00375;LINC003	85811909	A	T	0.0890575	0.0665	0.0519	0.1097	0.0676	0.1329
3	195597805	rs4677618	-0.5714	0.004506	-0.6591	0.002476	-0.2486	0.06972	-0.5612	0.0006448	intergenic	POD;LOC1053742	195324676	G	A	0.430112	0.7173	0.4251	0.3472	0.3877	0.2935
8	27530682	rs10503812	0.6445	0.004506	0.7446	0.001626	-0.03913	0.8195	0.1842	0.3482	intronic	EPHX2	27388199	G	A	0.275958	0.1885	0.1715	0.5431	0.1461	0.2127
1	101805273	rs1327594	0.5613	0.00451	0.5425	0.009075	0.5685	7.31e-05	0.6255	0.0001577	intronic	OLFM3	102270829	C	T	0.528355	0.6488	0.4179	0.6021	0.5875	0.3221
6	52233462	rs67216096	-1.759	0.00451	-1.981	0.007349	-1.561	5.01e-05	-1.298	0.002195	intergenic	IL17A;IL17F	52098260	TTAAA	T	0.0255591	0	0.0231	0.0045	0.0606	0.046
17	22445008	rs138786273	1.38	0.004522	1.54	0.001446	0.3875	0.382	0.8116	0.07759	intergenic	J36000;MTRNR2	21944338	G	A	0.0133786	0	0.0317	0.0015	0.0348	0.0082
4	70541607	rs4146575	0.6148	0.004528	0.5985	0.008926	0.6599	2.18e-05	0.6469	0.0002719	intergenic	AMTN;AMBN	71407324	T	C	0.715455	0.5923	0.7378	0.8154	0.7416	0.6646
5	2338251	rs4505958	0.5553	0.004553	0.5746	0.004934	0.3897	0.005924	0.5867	0.0002998	intergenic	C100506858;LSIN	2338365	G	A	0.327077	0.2222	0.3429	0.2315	0.4284	0.4489
5	118271040	rs1382731	0.646	0.004563	0.7908	0.0008483	0.1588	0.3637	0.3503	0.07694	intergenic	NC02147;LINC021	117606735	T	C	0.233027	0.0248	0.1859	0.4803	0.2326	0.1472
5	118271114	rs4635967	0.646	0.004563	0.7908	0.0008483	0.1669	0.3393	0.3614	0.06758	intergenic	NC02147;LINC021	117606809	A	G	0.236222	0.0248	0.1902	0.4902	0.2326	0.1472
19	54593223	rs900783153	1.254	0.004572	1.311	0.003731	0.9403	0.00897	1.439	0.0001573	upstream	LILRA1	NA	NA	NA	NA	NA	NA	NA	NA	NA
1	75509134	rs6658825	-0.6682	0.004585	-0.7597	0.002476	-0.3892	0.01646	-0.7366	0.0002407	intronic	SLC44A5	75974819	C	T	0.223442	0.0387	0.3012	0.177	0.3559	0.2853
3	195600142	rs6795576	-0.568	0.004606	-0.6755	0.001848	-0.2707	0.04888	-0.5779	0.0004576	intergenic	POD;LOC1053742	195327013	G	A	0.436302	0.7143	0.4251	0.3729	0.3877	0.2935
18	74587962	rs7505143	-0.566	0.004619	-0.6188	0.003208	-0.5511	9.32e-05	-0.6168	0.0001767	downstream	CNDP1	72255198	G	A	0.49381	0.5744	0.6066	0.2436	0.5895	0.5706
1	101806218	rs4907955	0.5592	0.00463	0.543	0.00895	0.5668	8.05e-05	0.6162	0.0001988	intronic	OLFM3	102271774	G	A	0.524361	0.6478	0.415	0.5908	0.5845	0.3231
20	29525395	rs1198671183	-0.8633	0.004638	-0.9277	0.004077	-0.8463	5.19e-05	-0.8874	0.0003461	intergenic	FRG1EP;FAM242I	NA	NA	NA	NA	NA	NA	NA	NA	NA
5	14900413	rs2860288	0.5471	0.004664	0.5012	0.01393	0.5799	9.05e-05	0.4915	0.003292	intergenic	ANKH;LINC0214S	14900522	C	A	0.711661	0.748	0.7161	0.7133	0.6322	0.7505
6	52245196	rs11465536	-1.761	0.004696	-1.989	0.007349	-1.558	5.82e-05	-1.295	0.002459	upstream	IL17F	52109994	A	G	0.0241613	0	0.0231	0.0023	0.0557	0.047
6	52249220	rs12210153	-1.761	0.004696	-1.989	0.007349	-1.558	5.82e-05	-1.295	0.002459	intergenic	IL17F;MCM3	52114018	C	T	0.0253594	0	0.0231	0.0068	0.0557	0.047
23	100748226	rs2154370	0.5443	0.004696	0.6182	0.002881	-0.005541	0.9648	0.1508	0.3034	intergenic	SYTL4;CSTF2	NA	NA	NA	NA	NA	NA	NA	NA	NA
6	52227090	rs77749223	-1.752	0.004705	-1.973	0.007644	-1.55	5.82e-05	-1.29	0.002378	intergenic	IL17A;IL17F	52091888	G	A	0.0249601	0	0.0231	0.0023	0.0606	0.046
6	52229113	rs79744878	-1.752	0.004705	-1.973	0.007644	-1.55	5.82e-05	-1.29	0.002378	intergenic	IL17A;IL17F	52093911	G	C	0.0249601	0	0.0231	0.0023	0.0606	0.046
18	10503666	rs508439	0.568	0.004709	0.56	0.007603	0.6189	2.08e-05	0.6369	0.0001715	intergenic	APCDD1;NAPG	10503663	A	G	0.639577	0.6687	0.5389	0.9266	0.4722	0.4652
7	43495658	rs17208350	0.6955	0.004732	0.6943	0.007122	0.7569	8.76e-05	0.639	0.003504	intronic	HECW1	43535257	G	A	0.107029	0.0079	0.1542	0.0091	0.2127	0.1994
7	43495753	rs75282349	0.6955	0.004732	0.6943	0.007122	0.7569	8.76e-05	0.639	0.003504	intronic	HECW1	43535352	C	A	0.107029	0.0079	0.1542	0.0091	0.2127	0.1994
5	118276713	rs1871366	0.6412	0.00475	0.7841	0.0009018	0.1639	0.3453	0.36	0.06771	intergenic	NC02147;LINC021	117612408	C	G	0.234425	0.0248	0.1873	0.4811	0.2356	0.1493
5	118276829	rs73243413	0.6412	0.00475	0.7841	0.0009018	0.1639	0.3453	0.36	0.06771	intergenic	NC02147;LINC021	117612524	C	A	0.234026	0.0248	0.1888	0.4796	0.2346	0.1493
5	118276877	rs1382735	0.6412	0.00475	0.7841	0.0009018	0.1639	0.3453	0.36	0.06771	intergenic	NC02147;LINC021	117612572	A	C	0.233826	0.0248	0.1888	0.4788	0.2346	0.1493
5	118276894	rs1382736	0.6412	0.00475	0.7841	0.0009018	0.1639	0.3453	0.36	0.06771	intergenic	NC02147;LINC021	117612589	C	T	0.235024	0.0248	0.1888	0.4834	0.2346	0.1493
7	43500518	rs17208513	0.6983	0.004772	0.69	0.007709	0.7854	6.52e-05	0.6369	0.004222	intronic	HECW1	43540117	C	T	0.0992412	0.0079	0.1441	0.0076	0.2068	0.1748
7	43478056	rs76632102	0.7021	0.004786	0.7061	0.005861	0.7691	6.62e-05	0.6334	0.00337	intronic	HECW1	43517655	T	G	0.107628	0.0069	0.1513	0.0091	0.2167	0.2014
7	32743771	rs56218467	0.668	0.00482	0.7516	0.002166	0.6738	8.37e-05	0.7612	0.0001183	intergenic	IR550A2;LINC00S	32783383	T	C	0.230431	0.1389	0.1686	0.2799	0.1779	0.3558
2	76426173	rs7423748	-0.7108	0.004848	-0.675	0.01069	-0.394	0.01628	-0.7052	0.0004854	intergenic	GCFC2;LRRTM4	76653299	A	G	0.264577	0.5089	0.3012	0.062	0.2177	0.3088
8	102727476	rs12679376	-0.8933	0.004849	-0.8347	0.01183	-0.994	1.63e-05	-0.8354	0.001232	intergenic	C101927245;GAS	103739704	G	A	0.111821	0.0982	0.2824	0.0998	0.0944	0.0389
6	52235942	rs11465561	-1.748	0.004857	-1.973	0.007644	-1.549	5.98e-05	-1.287	0.002436	downstream	IL17F	52100740	C	T	0.0241613	0	0.0231	0.0023	0.0567	0.046
15	24212851	rs7178856	-0.8421	0.004875	-1.051	0.001393	-0.8476	4.7e-05	-0.9463	0.0001398	intergenic	PWRN2;PWRN3	NA	NA	NA	NA	NA	NA	NA	NA	NA
10	59141576	rs12268935	1.259	0.004882	1.292	0.005224	0.8303	0.01385	1.453	0.0001626	intergenic	LINC00844;CCEPI	60901336	T	C	0.0884585	0.0327	0.0519	0.2133	0.0119	0.0818
1	13681154	rs4662013	-0.6483	0.00492	-0.6686	0.006179	-0.5963	0.00029	-0.7742	8.25e-05	intergenic	PDPN;PRDM2	14007649	A	G	0.771366	0.7768	0.7089	0.8162	0.7535	0.7679
9	138148935	rs1396392235	-1.305	0.004931	-1.346	0.007151	-1.445	9.19e-06	-1.354	0.0004808	intergenic	CACNA1B;TUBBP	NA	NA	NA	NA	NA	NA	NA	NA	NA
17	4451878	rs954524	-0.6019	0.004974	-0.8177	0.0004743	-0.2573	0.09258	-0.6387	0.0004494	intronic	SPNS3	4355173	T	C	0.521366	0.2252	0.683	0.5008	0.5974	0.6616
9	111086018	rs58041395	-0.5817	0.004984	-0.4809	0.02589	-0.6028	4.22e-05	-0.4008	0.01589	intergenic	LPAR1;MIR7702	113848298	GA	G	0.425919	0.6319	0.3689	0.4939	0.2604	0.3323
2	91979050	rs9678157	-0.7945	0.004986	-0.8329	0.005399	-0.8451	3.1e-05	-0.8476	0.0003199	intergenic	ACTR3BP2;NONE	NA	NA	NA	NA	NA	NA	NA	NA	NA
20	30186028	rs77635548	0.7575	0.004994	0.8476	0.002693	0.8399	1.63e-05	0.9259	4.54e-05	intergenic	IR4477A;LINC015	NA	NA	NA	NA	NA	NA	NA	NA	NA
20	30186030	rs77434032	0.7575	0.004994	0.8476	0.002693	0.8399	1.63e-05	0.9259	4.54e-05	intergenic	IR4477A;LINC015	NA	NA	NA	NA	NA	NA	NA	NA	NA
6	166192873	rs36072720	-1.257	0.005012	-1.274	0.008103	-1.146	9.89e-05	-1.198	0.0008866	intergenic	CDAT;LOC101929	166606361	A	G	0.0732827	0.0337	0.0634	0.0537	0.1054	0.1145
16	34140083	rs995854250	-1.755	0.005012	-1.614	0.009729	-1.443	5.56e-05	-1.537	0.0007859	intergenic	NPP7P13;MIR99	NA	NA	NA	NA	NA	NA	NA	NA	NA
11	27715576	rs10219241	0.5244	0.005016	0.623	0.002065	0.2537	0.05688	0.5612	0.0003733	intronic	BDNF	27737123	G	A	0.523562	0.5833	0.4236	0.4304	0.5278	0.6544
7	43495085	rs76205128	0.6902	0.005076	0.6886	0.007626	0.7519	9.77e-05	0.6337	0.003796	intronic	HECW1	43534684	A	G	0.107029	0.0079	0.1542	0.0091	0.2127	0.1994
6	166184985	rs62439030	-1.278	0.005081	-1.296	0.008264	-1.21	7.89e-05	-1.308	0.0006028	intergenic	CDAT;LOC101929	166598473	G	T	0.0648962	0.0248	0.0634	0.028	0.1054	0.1155
7	20859911	rs11768686	-0.7634	0.005087	-0.8051	0.005779	-0.7625	6.25e-05	-0.7147	0.0012	ncRNA_intronic	LINC01162	20899530	G	A	0.149161	0.1815	0.1556	0.0151	0.1431	0.2986
18	10486370	rs550247	-0.5569	0.005091	-0.5293	0.01021	-0.5628	6.96e-05	-0.6329	0.0001608	intronic	APCDD1	10486367	A	G	0.518371	0.6488	0.5403	0.5242	0.4592	0.4213
2	188325081	rs12151502	1.03	0.005094	1.143	0.002596	0.568	0.03014	1.069	0.0003431	intronic	GULP1	189189808	G	A	0.155152	0.2123	0.036	0.2262	0.0417	0.2014
3	195599574	rs150186602	-0.5607	0.005117	-0.6663	0.002093	-0.2643	0.05404	-0.5717	0.0005177	intergenic	POD;LOC1053742	195326445	A	G	0.436901	0.7163	0.428	0.3729	0.3887	0.2914
5	14887100	rs835144	0.5459	0.005119	0.4385	0.03209	0.5713	7.67e-05	0.5951	0.0003195	intergenic	ANKH;LINC0214S	14887209	T	C	0.461861	0.3353	0.4294	0.6006	0.5477	0.3395
5	14887138	rs2441024	0.5459	0.005119	0.4385	0.03209	0.5713	7.67e-05	0.5951	0.0003195	intergenic	ANKH;LINC0214S	14887247	T	C	0.462859	0.3353	0.4308	0.6006	0.5477	0.3436
5	14887140	rs2441023	0.5459	0.005119	0.4385	0.03209	0.5713	7.67e-05	0.5951	0.0003195	intergenic	ANKH;LINC0214S	14887249	T	A	0.462859	0.3353	0.4308	0.6006	0.5477	0.3436
7	152287767	rs77806526	-1.039	0.005121	-0.8804	0.01842	-1.079	1.56e-05	-1.118	0.000229	intronic	KMT2C	NA	NA	NA	NA	NA	NA	NA	NA	NA
6	166196832	rs6941781	-1.185	0.005134	-1.376	0.004684	-1.127	7.79e-05	-1.288	0.00037	intergenic	CDAT;LOC101929	166610320	A	C	0.0730831	0.0248	0.0663	0.0582	0.1074	0.1125
2	78894738	rs138982464	0.68	0.005143	0.8316	0.0008622	0.07141	0.7026	0.2937	0.1607	intergenic	C105374820;REG	79121864	T	A	0.151158	0.1329	0.1153	0.1717	0.1352	0.184
2	188244156	rs4372844	1.196	0.005146	1.397	0.001445	0.9245	0.002426	1.357	9.18e-05	ncRNA_intronic	LINC01090	189108883	G	A	0.100439	0.2113	0.0216	0.0159	0.0378	0.2209
7	43504313	rs75399458	0.6943	0.005169	0.6854	0.008497	0.8182	3.82e-05	0.6755	0.002566	intronic	HECW1	43543912	G	A	0.0982428	0.0079	0.1398	0.0076	0.2038	0.1759
17	4451197	rs4790194	-0.602	0.005172	-0.8107	0.0005362	-0.3129	0.04249	-0.6279	0.0005622	intronic	SPNS3	4354492	C	G	0.48143	0.2262	0.6715	0.3691	0.5924	0.6472
2	188241689	rs4536617	1.197	0.00518	1.395	0.00148	0.9349	0.002184	1.369	8.15e-05	ncRNA_intronic	LINC01090	189106416	C	T	0.0998403	0.2113	0.0216	0.0159	0.0348	0.2209
10	112799020	rs941826	-0.6587	0.005187	-0.842	0.001386	-0.4923	0.002032	-0.8122	5.38e-05	intronic	VTI1A	114558779	C	T	0.175519	0.2421	0.2233	0.0492	0.2515	0.1656
8	102744237	rs4734666	-0.9139	0.005203	-0.7928	0.01777	-0.9289	6.73e-05	-0.7202	0.004985	intergenic	C101927245;GAS	103756465	C	G	0.0858626	0.1091	0.2594	0.0083	0.0895	0.0399
2	227659594	rs4321359	0.5508	0.005228	0.6349	0.002285	0.0514	0.7275	0.2805	0.08949	intergenic	C2orf83;SLC19A3	228524310	T	C	0.634585	0.6657	0.7579	0.4887	0.6978	0.6472
18	10487446	rs538795	0.557	0.005253	0.5256	0.01112	0.5978	3.3e-05	0.6683	7.83e-05	intronic	APCDD1	10487443	T	C	0.533147	0.6498	0.549	0.5666	0.4692	0.4223
5	118256758	rs17143923	0.6493	0.005274	0.7965	0.001013	0.2013	0.2634	0.4121	0.04191	ncRNA_intronic	LINC02147	117592453	G	A	0.223442	0.0258	0.183	0.4463	0.2326	0.1452
8	40244253	rs115751409	-1.03	0.005277	-0.8838	0.01683	-0.9998	6.43e-05	-0.7829	0.005607	intergenic	TCIM;SIRLNT	40101772	G	A	0.11901	0.0268	0.0951	0.118	0.1362	0.2147
2	188245199	rs76067012	1.193	0.005312	1.391	0.001523	0.9319	0.002256	1.366	8.47e-05	ncRNA_intronic	LINC01090	189109926	T	G	0.0996406	0.2113	0.0216	0.0151	0.0348	0.2209
4	130539637	rs7689653	-0.6686	0.005342	-0.7012	0.005894	-0.4351	0.00944	-0.7794	0.0001693	intergenic	INC02479;SNHG2	131460792	G	A	0.179113	0.0288	0.1931	0.1195	0.3946	0.183
19	1698725	rs55654895	-0.7862	0.005378	-0.8951	0.003358	-0.6343	0.001034	-0.9596	9.18e-05	intergenic	TCF3;ONECUT3	1698724	C	G	0.184105	0.0496	0.1441	0.2988	0.1879	0.1922
19	29712130	rs10407128	-0.5749	0.005398	-0.6641	0.002085	-0.4419	0.002026	-0.6739	8.09e-05	intronic	C19orf12	30203037	C	G	0.567292	0.5655	0.536	0.8207	0.3728	0.4489
6	166182463	rs71029590	-1.266	0.005406	-1.286	0.008644	-1.19	9.99e-05	-1.08	0.002124	intergenic	CDAT;LOC101929	166595951	C	CAAAT	0.0628994	0.0248	0.062	0.0219	0.1054	0.1145
6	166186688	rs118062743	-1.266	0.005406	-1.286	0.008644	-1.201	8.78e-05	-1.197	0.001054	intergenic	CDAT;LOC101929	166600176	G	A	0.063099	0.0248	0.0634	0.0318	0.1054	0.1012
5	135494179	rs17168468	1.303	0.005408	1.575	0.001079	0.4495	0.2214	0.805	0.05071	intergenic	TIFAB;NEUROG1	134829869	A	C	0.0579073	0.0486	0.0692	0.1384	0.004	0.0061
4	130549532	rs150284329	-0.6641	0.005475	-0.6947	0.006133	-0.4318	0.00957	-0.7754	0.0001689	intergenic	INC02479;SNHG2	131470687	C	CA	0.179912	0.0308	0.196	0.1195	0.3946	0.183
2	50568758	rs72835372	1.407	0.005506	1.536	0.002367	0.2993	0.5232	0.7167	0.1388	intronic	NRXN1	50795896	G	C	0.028754	0.0188	0.0793	0.0008	0.0169	0.0532
2	50569921	rs72835375	1.407	0.005506	1.536	0.002367	0.2993	0.5232	0.7167	0.1388	intronic	NRXN1	50797059	G	C	0.0307508	0.0159	0.0793	0.0008	0.0179	0.0654
12	42874714	rs4768457	-0.5964	0.005507	-0.6407	0.004123	-0.51	0.0005515	-0.722	4.67e-05	intergenic	NC02450;LINC024	43268517	G	A	0.647764	0.8026	0.5533	0.702	0.5239	0.6094
2	188228849	rs139110719	1.188	0.005511	1.386	0.00158	0.9246	0.002443	1.359	9.1e-05	ncRNA_intronic	LINC01090	189093576	A	G	0.0994409	0.2113	0.0216	0.0144	0.0348	0.2209
5	118247589	rs11739862	0.6463	0.005518	0.7936	0.001065	0.1952	0.2776	0.4087	0.0437	ncRNA_intronic	LINC02147	117583284	T	G	0.221046	0.0258	0.1801	0.4372	0.2326	0.1472
2	188258060	rs6735839	1.018	0.00552	1.144	0.002506	0.7725	0.003378	1.167	8.54e-05	ncRNA_intronic	LINC01090	189122787	T	A	0.151558	0.2083	0.0317	0.1876	0.0497	0.2342
3	75673710	rs62248031	0.939	0.005525	0.8744	0.01256	0.89	9.02e-05	0.9263	0.0006024	ncRNA_intronic	LINC00960	NA	NA	NA	NA	NA	NA	NA	NA	NA
4	130551066	rs62318256	-0.6666	0.005534	-0.7001	0.006031	-0.4384	0.009011	-0.801	0.0001236	intergenic	INC02479;SNHG2	131472221	G	A	0.180911	0.0308	0.1931	0.1203	0.3976	0.1861
7	43502753	rs17208534	0.682	0.00556	0.6409	0.01325	0.7852	6.38e-05	0.6321	0.004418	intronic	HECW1	43542352	T	C	0.0980431	0.0079	0.1398	0.0076	0.2038	0.1748
7	43507018	rs117689113	0.682	0.00556	0.6409	0.01325	0.7852	6.38e-05	0.6321	0.004418	intronic	HECW1	43546617	C	T	0.0980431	0.0079	0.1412	0.0076	0.2028	0.1748
6	52224761	rs6941084	-0.8542	0.005586	-0.8557	0.008355	-0.8465	9.14e-05	-0.6667	0.005448	intergenic	IL17A;IL17F	52089559	G	T	0.105032	0.0427	0.0836	0.0802	0.1551	0.1667
4	24486797	rs16875707	0.5432	0.005608	0.5067	0.01409	0.4731	0.0006401	0.6609	7.59e-05	intergenic	PARGC1A;MIR57	24488420	A	G	0.562101	0.5962	0.5821	0.5393	0.5895	0.5153
4	70495397	rs13148900	0.6033	0.00563	0.6298	0.00568	0.6622	2.41e-05	0.6823	0.0001194	intergenic	MUC7;AMTN	71361114	T	G	0.729433	0.6091	0.732	0.8593	0.7117	0.6943
4	130542305	rs11734424	-0.6638	0.005654	-0.6946	0.006291	-0.4333	0.009707	-0.7953	0.0001334	intergenic	INC02479;SNHG2	131463460	C	T	0.179313	0.0288	0.1945	0.1195	0.3946	0.183
3	721902	rs9883263	-0.793	0.005657	-0.8217	0.007311	-0.8058	3.23e-05	-0.8099	0.0005665	ncRNA_intronic	LINC01266	763586	C	G	0.307708	0.3393	0.1787	0.5446	0.0934	0.2669
10	59161045	rs12255741	1.239	0.00566	1.274	0.005975	0.825	0.0145	1.453	0.0001706	intergenic	LINC00844;CCEPI	60920805	A	G	0.0848642	0.0327	0.049	0.2012	0.0119	0.0818

2	76429600	rs10196661	-0.6909	0.005697	-0.6366	0.01528	-0.3704	0.02331	-0.6821	0.0006846	intergenic	GCFC2;LRRTM4	76656726	A	G	0.735623	0.4931	0.6988	0.938	0.7823	0.6902
11	70635141	rs489717	0.526	0.005709	0.4217	0.03353	0.5868	3.25e-05	0.5839	0.0002386	ncRNA_exonic	SHANK2-AS1	70481246	T	C	0.386581	0.3056	0.4078	0.3064	0.497	0.4499
3	195594723	rs9886959	-0.555	0.005727	-0.6301	0.003572	-0.2556	0.06467	-0.5695	0.0005825	intergenic	OD;LOC1053742	195321594	A	G	0.488618	0.7143	0.4409	0.5794	0.3708	0.2883
5	141914555	rs62379961	1.058	0.005763	1.234	0.001436	0.3683	0.2502	0.6186	0.07788	intergenic	OC729080;DELE	141294120	C	G	0.0676917	0.1002	0.0317	0.0749	0.0398	0.0787
17	75689579	rs2053508	0.5558	0.005788	0.6768	0.001496	0.4736	0.0007635	0.676	5.67e-05	intronic	SAP30BP	73685659	A	G	0.413139	0.4732	0.4712	0.1384	0.5954	0.4939
1	216431026	rs114736194	1.856	0.005878	1.943	0.003391	1.77	0.0009401	2.107	0.0001187	intergenic	USH2A;ESRRG	216604368	G	C	0.0173722	0	0.0317	0.003	0.0457	0.0153
4	130563529	rs11099063	-0.666	0.005897	-0.7076	0.005881	-0.4325	0.01024	-0.7633	0.0002388	intergenic	INC02479;SNHG2	131484684	C	T	0.179513	0.0308	0.1931	0.1188	0.3926	0.1861
2	188220131	rs16829913	1.174	0.0059	1.395	0.00148	0.9283	0.002331	1.36	9.02e-05	ncRNA_intronic	LINC01090	189084858	G	A	0.095647	0.2083	0.0202	0.0023	0.0348	0.2219
2	188233811	rs77518981	1.174	0.0059	1.395	0.00148	0.9283	0.002331	1.36	9.02e-05	ncRNA_intronic	LINC01090	189098538	T	C	0.0994409	0.2113	0.0216	0.0144	0.0348	0.2209
20	3188331	rs117398381	0.9098	0.005922	1.076	0.001594	0.6758	0.01136	1.053	0.0002377	intergenic	LZTS3;DDRGK1	3168977	G	A	0.0335463	0	0.062	0.0038	0.1054	0.0143
7	43516989	rs77962581	0.7259	0.005924	0.7428	0.005989	0.8137	9.05e-05	0.705	0.002322	ncRNA_intronic	LUARIS	43556588	G	A	0.0890575	0.0089	0.1196	0.0068	0.1809	0.1667
20	25916494	rs76267272	0.8153	0.005927	0.8743	0.005685	0.9748	3.9e-06	0.8571	0.000419	intergenic	101926935;LINC0	NA	NA	NA	NA	NA	NA	NA	NA	NA
5	2345571	rs7734490	0.5515	0.005938	0.5589	0.006914	0.395	0.00619	0.5961	0.0003043	intergenic	C100506858;LSIN	2345685	G	A	0.290535	0.2192	0.3156	0.1316	0.4175	0.4305
5	2347169	rs958510	0.5515	0.005938	0.5589	0.006914	0.395	0.00619	0.5961	0.0003043	intergenic	C100506858;LSIN	2347283	A	G	0.290535	0.2192	0.3156	0.1316	0.4175	0.4305
2	188257195	rs57795591	1.005	0.005953	1.157	0.00225	0.7358	0.005312	1.147	0.0001132	ncRNA_intronic	LINC01090	189121922	T	TCTG	0.151558	0.2073	0.0331	0.1884	0.0497	0.2331
7	32585487	rs3801332	0.6126	0.005972	0.6646	0.004514	0.6098	9.23e-05	0.7388	4.93e-05	ncRNA_intronic	DPY19L1P1	32625099	C	T	0.272963	0.1329	0.2061	0.3707	0.2406	0.3661
6	52213186	rs1999673	-0.8451	0.006029	-0.8489	0.00879	-0.8566	7.39e-05	-0.6639	0.005569	intergenic	IL17A;IL17F	52077984	C	A	0.117212	0.0427	0.0937	0.1203	0.1551	0.1677
5	135492426	rs73791408	1.236	0.006053	1.482	0.00142	0.4921	0.1767	0.789	0.05074	intergenic	TIFAB;NEUROG1	134828116	T	C	0.0513179	0.0476	0.0706	0.1135	0.004	0.0061
2	188258268	rs6749420	1.002	0.006071	1.154	0.002295	0.7753	0.00323	1.166	8.46e-05	ncRNA_intronic	LINC01090	189122995	A	T	0.151558	0.2083	0.0317	0.1876	0.0497	0.2342
2	91937812	rs62154670	-0.7923	0.006089	-0.7299	0.01523	-0.8403	3.47e-05	-0.8833	0.0002171	intergenic	GGT8P;ACTR3BP2	NA	NA	NA	NA	NA	NA	NA	NA	NA
3	195597739	rs4677617	-0.5525	0.006109	-0.6591	0.002476	-0.2409	0.07922	-0.551	0.0008166	intergenic	OD;LOC1053742	195324610	G	A	0.430112	0.7173	0.4251	0.3472	0.3877	0.2935
12	107747711	rs73189570	0.972	0.00612	0.9976	0.006453	0.8416	0.002272	1.184	0.0001017	ncRNA_intronic	LOC101929162	108141488	A	G	0.0842652	0.1091	0.0375	0.1067	0.0547	0.092
9	111083889	rs10980748	-0.5744	0.006126	-0.4407	0.04136	-0.5789	8.93e-05	-0.3858	0.02111	intergenic	LPAR1;MIR7702	113846169	T	G	0.382588	0.6359	0.3617	0.32	0.2724	0.3344
17	4451053	rs6502788	-0.5887	0.006137	-0.7962	0.0006519	-0.2785	0.06894	-0.614	0.0007227	intronic	SPNS3	4354348	A	C	0.481829	0.2212	0.6686	0.3752	0.5934	0.6472
4	130549472	rs62318254	-0.6562	0.006139	-0.6875	0.006775	-0.4196	0.01197	-0.7648	0.000212	intergenic	INC02479;SNHG2	131470627	T	A	0.179513	0.0308	0.1931	0.1195	0.3946	0.183
4	130552624	rs12152613	-0.6562	0.006139	-0.6875	0.006775	-0.4196	0.01197	-0.7648	0.000212	intergenic	INC02479;SNHG2	131473779	T	C	0.180911	0.0308	0.1931	0.1203	0.3976	0.1861
4	130556256	rs11514609	-0.6562	0.006139	-0.6875	0.006775	-0.4196	0.01197	-0.7648	0.000212	intergenic	INC02479;SNHG2	131477411	T	C	0.180911	0.0308	0.1931	0.1203	0.3976	0.1861
4	130556675	rs3034836	-0.6562	0.006139	-0.6875	0.006775	-0.4196	0.01197	-0.7648	0.000212	intergenic	INC02479;SNHG2	131477830	CAT	C	0.180911	0.0308	0.1931	0.1203	0.3976	0.1861
2	188250185	rs72903530	1.001	0.006166	1.152	0.002341	0.7689	0.003514	1.158	9.46e-05	ncRNA_intronic	LINC01090	189114912	A	G	0.151558	0.2083	0.0317	0.1876	0.0497	0.2342
11	71217225	rs12222935	1.071	0.006181	1.023	0.01062	1.166	4.29e-05	1.056	0.0008759	intronic	SHANK2	70928271	G	T	0.0521166	0.126	0.0346	0.0008	0.0348	0.0757
10	4618063	rs72770156	1.176	0.0062	1.03	0.0247	0.8796	0.01458	1.349	0.0003382	intergenic	INC00703;MANC	4660255	T	C	0.0419329	0.0179	0.0274	0.0038	0.0577	0.1125
13	101585101	rs1925990	-0.5773	0.006352	-0.5903	0.008804	-0.3367	0.01981	-0.6615	0.0002367	intronic	ITGBL1	102237451	T	C	0.334864	0.4673	0.3329	0.2209	0.3042	0.3855
4	130544668	rs62318253	-0.6534	0.006378	-0.6854	0.006969	-0.4133	0.01321	-0.759	0.0002339	intergenic	INC02479;SNHG2	131465823	G	A	0.179313	0.0288	0.1945	0.1195	0.3946	0.183
4	130545621	rs7672122	-0.6534	0.006378	-0.6854	0.006969	-0.4133	0.01321	-0.759	0.0002339	intergenic	INC02479;SNHG2	131466776	T	C	0.179912	0.0308	0.1945	0.1203	0.3946	0.183
4	130549303	rs140100053	-0.6534	0.006378	-0.6854	0.006969	-0.4133	0.01321	-0.759	0.0002339	intergenic	INC02479;SNHG2	131470458	A	AT	0.18131	0.0357	0.1945	0.1218	0.3936	0.184
4	130559279	rs7657378	-0.6534	0.006378	-0.6854	0.006969	-0.4284	0.01052	-0.7883	0.0001507	intergenic	INC02479;SNHG2	131480434	C	T	0.18151	0.0337	0.1931	0.1195	0.3986	0.1861
4	130559809	rs7662917	-0.6534	0.006378	-0.6854	0.006969	-0.4133	0.01321	-0.759	0.0002339	intergenic	INC02479;SNHG2	131480964	C	T	0.180511	0.0288	0.1931	0.1195	0.3986	0.1861
10	59102939	rs138505624	1.834	0.006465	1.794	0.008778	1.684	0.001564	2.254	9.26e-05	intergenic	LINC00844;CCEPI	60862699	G	A	0.048722	0.0317	0.0375	0.0877	0.0099	0.0613
10	59110336	rs80323272	1.834	0.006465	1.794	0.008778	1.686	0.001535	2.257	8.9e-05	intergenic	LINC00844;CCEPI	60870096	A	T	0.0489217	0.0317	0.0375	0.0885	0.0099	0.0613
10	59110828	rs74436219	1.834	0.006465	1.794	0.008778	1.684	0.001564	2.254	9.26e-05	intergenic	LINC00844;CCEPI	60870588	T	C	0.0489217	0.0317	0.0375	0.0885	0.0099	0.0613
7	20856443	rs17143423	-0.7306	0.006467	-0.7668	0.007562	-0.8006	2.89e-05	-0.7417	0.0008001	ncRNA_intronic	LINC01162	20896062	A	G	0.14996	0.1677	0.1715	0.0151	0.1451	0.3037
12	107717158	rs73189504	0.9658	0.006474	0.9976	0.006453	0.836	0.00242	1.179	0.0001086	intergenic	PWP1;PRDM4	108110935	A	G	0.0844649	0.1091	0.0389	0.1067	0.0547	0.092
12	107740255	rs73189568	0.9658	0.006474	0.9976	0.006453	0.836	0.00242	1.179	0.0001086	ncRNA_intronic	LOC101929162	108134032	G	A	0.0830671	0.1091	0.0375	0.1021	0.0547	0.092
12	107759794	rs3741791	0.9658	0.006474	0.9976	0.006453	0.836	0.00242	1.179	0.0001086	ncRNA_exonic	LOC101929162	108153571	G	C	0.0836661	0.1091	0.0389	0.1029	0.0557	0.092
4	70551808	rs28847396	0.5728	0.006487	0.5384	0.01487	0.6267	3.14e-05	0.6156	0.0003599	intergenic	AMTN;AMBN	71417525	A	T	0.695487	0.5942	0.7349	0.7247	0.7406	0.6861
3	691848	rs34013295	-0.8153	0.006528	-0.8074	0.01075	-0.8062	5.4e-05	-0.9025	0.0002373	ncRNA_intronic	LINC01266	733532	G	GA	0.701278	0.6677	0.8228	0.4788	0.9175	0.728
7	32774846	rs6972178	0.6301	0.006538	0.7083	0.003063	0.6397	0.0001366	0.7595	8.72e-05	intergenic	IC00997;DPY19L1	32814458	T	C	0.248802	0.1399	0.1744	0.3464	0.1789	0.3538
5	75897666	rs200191498	-0.8153	0.006572	-0.8712	0.00503	-0.923	2.64e-05	-0.8702	0.0004708	intergenic	LOC441087;SV2C	NA	NA	NA	NA	NA	NA	NA	NA	NA
8	40216272	rs6989028	-0.974	0.006588	-0.8098	0.02455	-0.9545	9.96e-05	-0.7131	0.009748	intergenic	TCIM;SIRLNT	40073791	T	C	0.119808	0.0268	0.0965	0.1188	0.1362	0.2168
8	40216666	rs7816927	-0.974	0.006588	-0.8098	0.02455	-0.9545	9.96e-05	-0.7131	0.009748	intergenic	TCIM;SIRLNT	40074185	A	G	0.120008	0.0268	0.0965	0.1195	0.1362	0.2168
8	40218419	rs10108159	-0.974	0.006588	-0.8098	0.02455	-0.9545	9.96e-05	-0.7131	0.009748	intergenic	TCIM;SIRLNT	40075938	G	A	0.119808	0.0258	0.0965	0.1188	0.1372	0.2168
1	13695599	rs2487657	-0.6548	0.006593	-0.6598	0.01004	-0.7036	6.79e-05	-0.8641	3.99e-05	intergenic	PDPN;PRDM2	14022094	A	G	0.827875	0.7827	0.732	0.9917	0.7684	0.7822
11	16936474	rs72865735	1.428	0.006596	1.634	0.001958	0.7134	0.1471	1.065	0.03447	intronic	PLEKHA7	16958021	C	T	0.00958466	0	0.0101	0.0023	0.0268	0.0112
5	135492927	rs740657	0.7759	0.0066	0.93	0.00184	0.1703	0.4152	0.3923	0.1081	intergenic	TIFAB;NEUROG1	134828617	G	T	0.200479	0.1865	0.1153	0.3949	0.0974	0.1186
8	40254042	rs10504020	-0.9719	0.00665	-0.821	0.02217	-0.9694	7.34e-05	-0.7199	0.008759	intergenic	TCIM;SIRLNT	40111561	T	G	0.120208	0.0268	0.0965	0.1195	0.1342	0.2198
19	54595586	rs1219524329	0.7612	0.00668	0.6276	0.03053	0.8283	5.7e-05	0.8374	0.0003608	intronic	LILRA1	NA	NA	NA	NA	NA	NA	NA	NA	NA
11	24735779	rs4923207	-0.6829	0.00669	-0.7298	0.007949	-0.7703	5.43e-05	-0.84	0.0002018	intronic	LUZP2	24757325	T	G	0.134385	0.1151	0.2565	0.0144	0.2197	0.1421
17	75712749	rs9898904	0.5619	0.006706	0.6602	0.002586	0.4592	0.001434	0.684	6.9e-05	intergenic	SAP30BP;ITGB4	73708829	G	A	0.388379	0.4663	0.4582	0.0325	0.5984	0.5235
10	59117884	rs148853067	1.683	0.006721	1.838	0.002802	1.367	0.003489	2.015	0.0001115	intergenic	LINC00844;CCEPI	60877644	G	T	0.0595048	0.0327	0.0403	0.1248	0.0119	0.0613
11	70637537	rs536637	0.5182	0.006725	0.421	0.03412	0.5857	3.82e-05	0.5778	0.0002893	intronic	SHANK2	70483642	C	G	0.371206	0.3036	0.3862	0.2912	0.4662	0.4407
9	10202567	rs35839517	-1.075	0.006738	-0.9127	0.02196	-1.268	2.05e-05	-1.419	0.0001401	intronic	PTPRD	10202567	CTA	C	0.160144	0.0278	0.1023	0.3994	0.1163	0.0593
3	195599349	rs7650134	-0.5437	0.006743	-0.6504	0.002708	-0.2617	0.05839	-0.5578	0.0007243	intergenic	OD;LOC1053742	195326220	A	G	0.437101	0.7163	0.428	0.3737	0.3887	0.2914
17	1168330	rs11655015	0.5146	0.006797	0.5671	0.004755	0.1923	0.1588	0.5363	0.0009315	intronic	ABR	1071624	T	C	0.284944	0.2173	0.4107	0.031	0.5149	0.3722
9	10196275	rs294831	-1.059	0.006819	-0.9005	0.0219	-1.13	6.41e-05	-1.387	0.0001645	intronic	PTPRD	10196275	C	T	0.826478	0.9692	0.8963	0.5802	0.8847	0.9029
8	40254396	rs78445425	-0.9666	0.006852	-0.8009	0.02562	-0.9666	7.67e-05	-0.7134	0.009393	intergenic	TCIM;SIRLNT	40111915	G	A	0.120208	0.0268	0.0965	0.1195	0.1342	0.2198
23	119753720	rs2782217	0.5213	0.006995	0.714	0.0007936	0.06197	0.6481	0.2636	0.09004	intergenic	SEPTIN6;SOWAHI	NA	NA	NA	NA	NA	NA	NA	NA	NA
4	190022563	rs58598341	-0.7343	0.007013	-0.7394	0.0097	-0.7696	6.91e-05	-0.8527	0.0001593	intergenic	FRG1;FRG2	NA	NA	NA	NA	NA	NA	NA	NA	NA
5	95850932	rs112128951	1.217	0.007061	1.377	0.002379	0.228	0.5856	0.652	0.1307	intergenic	102724720;LINC0	95186636	C	T	0.00938498	0.002	0.0101	0.0015	0.0298	0.0061
8	27493702	rs59162874	0.6191	0.007067	0.7179	0.002792	-0.0666	0.7103	0.1811	0.3701	intronic	EPHX2	27351219	T	TG	0.301118	0.1597	0.1585	0.6573	0.1501	0.2219
8	27496954	rs6999685	0.6191	0.007067	0.7179	0.002792	-0.0666	0.7103	0.1811	0.3701	intronic	EPHX2	27354471	A	G	0.302117	0.1607	0.1585	0.6589	0.1501	0.2239
8	27497613	rs112452787	0.6191	0.007067	0.7179	0.002792	-0.0666	0.7103	0.1811	0.3701	intronic	EPHX2	27355130	T	C	0.301917	0.1607	0.1585	0.6589	0.1501	0.2229
8	40217541	rs77082986	-0.9666	0.00708	-0.8139	0.02381	-0.9561	9.88e-05	-0.712	0.009937	intergenic	TCIM;SIRLNT	40075060	T	A	0.119808	0.0268	0.0965	0.1188	0.1362	0.2168
4	130553995	rs1824372	-0.647	0.007157	-0.6796	0.00773	-0.3905	0.01988	-0.7344	0.0004018	intergenic	INC02479;SNHG2	131475150	T	G	0.180911	0.0308	0.1931	0.1203	0.3976	0.1861
2	78901458	rs56136828	0.6071	0.007159	0.785	0.0007906	0.1057	0.5445	0.2504	0.1977	intergenic	C105374820;REG	79128584	G	C	0.146366	0.127	0.1254	0.1278	0.1561	0.1963
4	130541665	rs56163617	-0.6388	0.007173	-0.6875	0.006775	-0.414	0.01303	-0.7543	0.0002456	intergenic	INC02479;SNHG2	131462820	T	A	0.179313	0.0288	0.1945	0.1195	0.3946	0.183
4	130541888	rs11726055	-0.6388	0.007173	-0.6875	0.006775	-0.414	0.01303	-0.7543	0.0002456	intergenic	INC02479;SNHG2	131463043	A	C	0.179313	0.0288	0.1945	0.1195	0.3946	0.183
2	158944741	rs7560785	0.6266	0.007179	0.5946	0.01296	0.2958	0.06676	0.6376	0.0008327	intergenic	DAPL1;TANC1	159801253	G	A	0.36901	0.0635	0.3516	0.6127	0.4503	0.2832
17	27250128	rs4795527	-0.713	0.007205	-0.6741	0.01462	-0.76	5.61e-05	-0.5989	0.004775	intergenic	105371703;MIR4	25577154	C	G	0.873203	0.8839	0.8069	0.9811	0.8191	0.819
11	27725444	rs7931247	0.5063	0.007206	0.6094	0.002846	0.292	0.02959	0.565	0.0003779	intergenic	BDNF;KIF18A	27746991	T	C	0.435104	0.4157	0.5663	0.4349	0.4771	0.319
4	130550322	rs62318255	-0.6415	0.007228	-0.6914	0.006797	-0.4163	0.01324	-0.7586	0.0002498	intergenic	INC02479;SNHG2	131471477	C	T	0.179712	0.0308	0.1931	0.1203	0.3946	0.183
8	27514029	rs7000388	0.6177	0.007269	0.7163	0.002895	-0.07943	0.6588	0.1769	0.3824	intronic	EPHX2	27371546	T	C	0.301717	0.1597	0.1585	0.6589	0.1511	0.2219
4	102901031	rs1483160089	-0.747	0.007273	-1.067	0.0005668	-0.08796	0.6435	-0.4211	0.05999	UTR3	SLC9B1	NA	NA	NA	NA	NA	NA	NA	NA	NA
23	25737511	rs5986464	-0.7272	0.007279	-0.7926	0.007562	-0.2187	0.1454	-0.7131	0.000943	intergenic	ARX;MAGEB18	NA	NA	NA	NA	NA	NA	NA	NA	NA
11	70636967	rs529310	0.5102	0.007298	0.407	0.04023	0.5767	4.37e-05	0.5733	0.0003069	intronic	SHANK2	70483072	A	C	0.386182	0.3056	0.4092	0.3056	0.497	0.4479
3	75772583	rs74803589	-0.8051	0.00732	-0.9485	0.002031	0.0856	0.7229	-0.2411	0.3676	intronic	ZNF717	NA	NA	NA	NA	NA	NA	NA	NA	NA
7	133389429	rs145579503	1.593	0.007323	1.806	0.002452	0.816	0.1501	1.109	0.05633	intronic	EXOC4	133074183	C	T	0.00579073	0	0.0043	0	0.0199	0.0061
2	73280144	rs77788622	1.042	0.007353	0.9848	0.01645	0.5538	0.05166	1.137	0.0004279	intronic	FBXO41	73507272	A	G	0.109625	0.1756	0.0375	0.115	0.0577	0.1391
11	70636139	rs533484	0.5126	0.007364	0.4152	0.03671	0.5751	5.17e-05	0.5706	0.0003432	intronic	SHANK2	70482244	C	A	0.375799	0.3056	0.3862	0.3048	0.4672	0.4427
1	13683228	rs2487651	-0.6401	0.007429	-0.6489	0.01021	-0.6845	7.88e-05	-0.9143	1.51e-05	intergenic	PDPN;PRDM2	14009723	C	G	0.777157	0.7579	0.7233	0.9281	0.7495	0.6595
4	130553920	rs62319780	-0.6359	0.007455	-0.6854	0.006969	-0.4225	0.01151	-0.7769	0.0001765	intergenic	INC02479;SNHG2	131475075	A	G	0.18111	0.0317	0.1931	0.1203	0.3976	0.1861
4	130562000	rs922078	-0.6359	0.007455	-0.6854	0.006969	-0.4077	0.01437	-0.7484	0.000271	intergenic	INC02479;SNHG2	131483155	A	C	0.180312	0.0288	0.1931	0.1188	0.3986	0.1861
11	70636697	rs67852913	0.5109	0.007515	0.4143	0.0372	0.5799	4.42e-05	0.5712	0.000334	intronic	SHANK2	70482802	ACGTGTG	A	0.373802	0.3046	0.3876	0.3026	0.4632	0.4397
12	14943406	rs11375521	0.6645	0.007589	0.7871	0.002249	0.7458	3.64e-05	0.7664	0.0002164	intronic	ARHGDIB	NA	NA	NA	NA	NA	NA	NA	NA	NA
23	121375449	rs149825612	0.6655	0.00761	0.7441	0.002843	0.1246	0.5593	0.3318	0.1459	intergenic	MIR3672;GRIA3	NA	NA	NA	NA	NA	NA	NA	NA	NA
18	10487356	rs580258	0.5264	0.007688	0.4954	0.01557	0.5883	3.62e-05	0.6233	0.000193	intronic	APCDD1	10487353	A	G	0.529553	0.6478	0.5432	0.5613	0.4602	0.4264
3	702374	rs1385194	-0.7903	0.007703	-0.7833	0.01253	-0.8162	4.29e-05	-0.8895	0.0002703	ncRNA_intronic	LINC01266	744058	C	G	0.716454	0.6667	0.8372	0.5356	0.9185	0.7188
10	59154467	rs16913243	1.212	0.007739	1.361	0.003597	0.7355	0.03568	1.354	0.0006031	intergenic	LINC00844;CCEPI	60914227	A	G	0.0796725	0.0327	0.049	0.1815	0.0119	0.0818
6	103394397	rs2179783	-0.5789	0.007747	-0.5839	0.01144	-0.6343	4.08e-05	-0.7157	9.17e-05	intergenic	GRIK2;HACE1	103842272	T	C	0.372204	0.3442	0.3401	0.5802	0.2972	0.2198
8	7121517	rs10046609	-0.5904	0.007768	-0.5648	0.01479	-0.6234	5e-05	-0.6633	0.0002903	intergenic	FA5;LOC101928(	6979039	A	C	0.335463	0.5496	0.3487	0.2231	0.2734	0.3211

9	138148914	rs1464935173	-0.9775	0.007773	-0.9224	0.01567	-1.31	2.08e-06	-1.078	0.0005687	intergenic	CACNA1B;TUBBP	NA	NA	NA	NA	NA	NA	NA	NA	NA
3	195602283	rs13303137	-0.5228	0.007787	-0.5841	0.005641	-0.2566	0.06038	-0.554	0.0006723	intergenic	POD;LOC1053742	195329154	G	A	0.449681	0.7163	0.4467	0.3775	0.4205	0.3047
11	70638298	rs581395	0.5085	0.00779	0.4111	0.03845	0.5746	5.26e-05	0.5692	0.0003535	intronic	SHANK2	70484403	A	G	0.377196	0.3056	0.3876	0.3071	0.4672	0.4458
14	92211553	rs71123325	0.5542	0.007868	0.5823	0.007754	0.6104	6.41e-05	0.5976	0.0006621	intergenic	CPSF2;SLC24A4	92677897	G	GGT	0.727835	0.9077	0.7118	0.795	0.5596	0.636
11	70635983	rs559452	0.5082	0.007887	0.4107	0.0389	0.5737	5.4e-05	0.5686	0.000361	intronic	SHANK2	70482088	T	C	0.375998	0.3056	0.3862	0.3048	0.4672	0.4438
11	70636119	rs533547	0.5082	0.007887	0.4107	0.0389	0.5737	5.4e-05	0.5686	0.000361	intronic	SHANK2	70482224	T	C	0.375998	0.3056	0.3862	0.3048	0.4672	0.4438
2	188215360	rs1595763	0.9844	0.007926	1.15	0.002316	0.6251	0.01525	1.131	0.0001373	ncRNA_intronic	LINC01090	189080087	C	T	0.851038	0.7847	0.9611	0.8253	0.9513	0.773
11	24562345	rs11028019	0.7618	0.007958	0.848	0.004967	0.4356	0.05337	0.7992	0.001072	intronic	LUZP2	24583891	T	C	0.0509185	0.0258	0.1398	0.0091	0.0885	0.0317
9	116901245	rs10817957	0.8277	0.00799	0.9269	0.004483	0.7596	0.0004444	1.039	2.88e-05	intronic	ASTN2	119663524	C	T	0.187899	0.2817	0.0836	0.2905	0.0527	0.1656
9	138148899	rs375381055	-0.9256	0.007996	-0.8406	0.01996	-1.253	2.31e-06	-0.9926	0.0008202	intergenic	CACNA1B;TUBBP	NA	NA	NA	NA	NA	NA	NA	NA	NA
11	70636307	rs568678	0.5079	0.008036	0.4099	0.03983	0.5726	5.57e-05	0.5675	0.0003726	intronic	SHANK2	70482412	A	G	0.376398	0.3056	0.3876	0.3048	0.4672	0.4448
10	38688408	rs61842174	-0.9868	0.008044	-0.9973	0.01015	-0.7706	0.009565	-1.216	0.0001546	intergenic	NC00999;ACTR3B	NA	NA	NA	NA	NA	NA	NA	NA	NA
8	27505619	rs4149239	0.606	0.008086	0.7129	0.00271	-0.1166	0.5093	0.1401	0.4842	intronic	EPHX2	27363136	A	G	0.266174	0.1607	0.1398	0.5401	0.1461	0.2178
4	70545795	rs28532597	0.5552	0.008127	0.5399	0.01481	0.6522	1.58e-05	0.6142	0.0003736	intergenic	AMTN;AMBN	71411512	A	C	0.718251	0.5923	0.7334	0.8147	0.7386	0.6861
8	105032636	rs117542308	1.132	0.008145	1.308	0.002229	0.3633	0.3455	0.7603	0.05533	intergenic	LRP12;ZFPM2	106044864	G	A	0.0125799	0.001	0.0231	0.0015	0.0328	0.0112
4	130529281	rs2926414	-0.6307	0.008151	-0.6578	0.009158	-0.4509	0.007917	-0.752	0.0002602	intergenic	INC02479;SNHG2	131450436	A	G	0.851038	0.9782	0.8098	0.9781	0.6123	0.8231
10	112793283	rs10787465	-0.5197	0.008249	-0.6247	0.003025	-0.4231	0.002499	-0.6767	5.65e-05	intronic	VTI1A	114553042	C	T	0.559505	0.5685	0.4784	0.4955	0.5795	0.6738
11	27725002	rs10767667	0.4952	0.008284	0.597	0.003308	0.2884	0.03105	0.5566	0.0004382	intergenic	BDNF;KIF18A	27746549	C	T	0.434704	0.4157	0.5663	0.4342	0.4771	0.318
22	20650189	rs7285501	0.5784	0.008286	0.6699	0.00317	0.4467	0.008202	0.7039	0.000202	intergenic	1ED15;POM121L	21004476	G	A	0.221046	0.0804	0.134	0.2148	0.2048	0.453
6	103395694	rs12211639	-0.571	0.008297	-0.5861	0.01099	-0.6447	3.35e-05	-0.7246	7.44e-05	intergenic	GRIK2;HACE1	103843569	T	C	0.401757	0.3462	0.3429	0.6861	0.3002	0.2209
9	63765454	rs3009631	-0.7051	0.00833	-0.7426	0.008702	-0.7503	9.68e-05	-0.7634	0.0006214	intergenic	100996643;LINC0	NA	NA	NA	NA	NA	NA	NA	NA	NA
4	70553482	rs28492877	0.5521	0.008348	0.5384	0.01487	0.6202	3.69e-05	0.6053	0.000439	intergenic	AMTN;AMBN	71419199	A	G	0.695088	0.5923	0.7349	0.7247	0.7406	0.6861
1	150542271	rs71622702	0.9207	0.008387	0.6795	0.07965	1.14	9.15e-05	1.08	0.0008461	intergenic	FALEC;ADAMTSL	150514747	C	T	0.0766773	0.001	0.049	0.1952	0.0706	0.0204
2	77252421	rs75101710	1.071	0.008447	1.082	0.0101	0.9691	0.003162	1.279	0.0002297	intronic	LRRTM4	77479547	C	A	0.0367412	0.001	0.0591	0.0038	0.0924	0.045
7	62347939	rs79280802	-0.915	0.008601	-1.272	0.002026	-0.931	9.21e-05	-1.009	0.0004803	intergenic	NONE;ZNF733P	NA	NA	NA	NA	NA	NA	NA	NA	NA
7	28483085	rs12671763	1.07	0.008644	1.14	0.006782	1.085	8.9e-05	0.982	0.001796	intronic	CREB5	28522703	C	A	0.0617013	0.2113	0.0202	0.0098	0.0278	0.0419
10	59141378	rs12244430	1.186	0.008698	1.327	0.00418	0.7462	0.03283	1.355	0.0005741	intergenic	LINC00844;CCEP	60901138	G	C	0.0802716	0.0327	0.049	0.1846	0.0119	0.0808
10	59149011	rs10491018	1.186	0.008698	1.327	0.00418	0.7462	0.03283	1.355	0.0005741	intergenic	LINC00844;CCEP	60908771	G	A	0.0770767	0.0327	0.0461	0.174	0.0119	0.0808
23	2818462	rs2316290	0.5065	0.00877	0.5426	0.007497	0.3794	0.005352	0.597	0.00021	intergenic	XG;GYG2	NA	NA	NA	NA	NA	NA	NA	NA	NA
20	12653478	rs6041514	0.5862	0.008774	0.6322	0.006464	0.5485	0.0008741	0.779	4.31e-05	intergenic	3TBD3;LINC0172	12634125	A	G	0.251398	0.3492	0.1282	0.2784	0.2038	0.2505
9	111090569	rs10980755	-0.5457	0.00878	-0.4335	0.04481	-0.5959	6.06e-05	-0.3959	0.01849	intergenic	LPAR1;MIR7702	113852849	A	G	0.355232	0.6339	0.3458	0.2375	0.2624	0.3292
19	30666846	rs7508481	0.6707	0.008845	0.8377	0.001534	0.1577	0.413	0.3134	0.1543	intronic	ZNF536	31157753	A	G	0.171725	0.0615	0.1052	0.3411	0.1918	0.0828
1	13689141	rs2495047	-0.6307	0.008959	-0.6598	0.01004	-0.6963	8.1e-05	-0.8533	4.99e-05	intergenic	PDPN;PRDM2	14015636	T	A	0.827875	0.7817	0.732	0.9917	0.7694	0.7822
4	70475005	rs10011835	0.5789	0.008964	0.5918	0.01055	0.6793	2.15e-05	0.638	0.0003547	intronic	MUC7	71340722	G	A	0.247604	0.3879	0.2622	0.0628	0.2873	0.3016
19	34518916	rs182556925	1.3	0.008979	1.135	0.03437	1.564	8.73e-05	1.67	0.0001152	intergenic	WTIP;ZNF807P	35009821	A	G	0.0435304	0.0714	0.0202	0.0393	0.0467	0.0337
4	70551556	rs28846795	0.5732	0.008982	0.526	0.02307	0.6527	2.35e-05	0.6125	0.0005957	intergenic	AMTN;AMBN	71417273	A	C	0.719848	0.5942	0.7392	0.8147	0.7406	0.6861
3	704408	rs1484515	-0.7576	0.009022	-0.8105	0.006858	-0.7994	4.92e-05	-0.8731	0.0002792	ncRNA_intronic	LINC01266	746092	G	T	0.703674	0.6667	0.8242	0.5008	0.9155	0.7127
11	70635879	rs572470	0.4998	0.009045	0.403	0.04286	0.5688	6.31e-05	0.5618	0.000429	intronic	SHANK2	70481984	A	G	0.376198	0.3056	0.3876	0.3048	0.4672	0.4438
11	70635905	rs535359	0.4998	0.009045	0.403	0.04286	0.5688	6.31e-05	0.5618	0.000429	intronic	SHANK2	70482010	A	C	0.375998	0.3056	0.3862	0.3048	0.4672	0.4438
8	27514672	rs7018249	0.5994	0.009072	0.6976	0.003662	-0.09572	0.5942	0.1586	0.4328	intronic	EPHX2	27372189	G	A	0.301518	0.1587	0.1585	0.6604	0.1491	0.2219
12	121162726	rs1186055	-0.5513	0.009073	-0.5881	0.008879	-0.3396	0.01861	-0.6828	0.0001662	intronic	P2RX7	121600529	C	A	0.386781	0.3472	0.317	0.5825	0.2654	0.3374
11	111218010	rs6589213	0.5564	0.009137	0.6903	0.001877	0.05657	0.7174	0.2528	0.1516	intergenic	NC02550;C11orf	111088734	T	C	0.744209	0.6637	0.6282	0.7905	0.7674	0.8231
11	132517326	rs494050	-0.5726	0.009145	-0.6404	0.005966	-0.4413	0.004267	-0.6817	0.0002163	intronic	OPCML	132387220	T	C	0.745607	0.996	0.598	0.8207	0.4592	0.7853
10	4608958	rs17297024	1.094	0.009175	0.9509	0.03409	0.7994	0.02312	1.268	0.0005521	intergenic	INC00703;MANC	4651150	C	T	0.0423323	0.0188	0.0274	0.0053	0.0577	0.1115
10	4613497	rs72770150	1.094	0.009175	0.9509	0.03409	0.7994	0.02312	1.268	0.0005521	intergenic	INC00703;MANC	4655689	T	C	0.0417332	0.0169	0.0274	0.0053	0.0567	0.1115
17	75706173	rs2305529	0.5327	0.009268	0.6436	0.002894	0.4872	0.0007137	0.689	5.45e-05	intronic	SAP30BP	73702253	G	A	0.386981	0.4663	0.4611	0.0325	0.5954	0.5174
13	22342948	rs6490728	0.7463	0.009277	0.5197	0.08926	0.8362	5.2e-05	0.4985	0.04623	intergenic	4C00540;LINC00	22917087	T	A	0.191294	0.131	0.1686	0.3525	0.1143	0.1309
4	130553682	rs72682410	-0.6202	0.009338	-0.6524	0.009951	-0.3846	0.02234	-0.7409	0.000362	intergenic	INC02479;SNHG2	131474837	A	G	0.180911	0.0308	0.1931	0.1203	0.3976	0.1861
11	25668943	rs7104152	-0.5104	0.009374	-0.5105	0.01359	-0.4552	0.001133	-0.6704	5.74e-05	intergenic	LUZP2;ANO3	25690489	G	A	0.398562	0.5109	0.5692	0.0802	0.4891	0.499
20	12653505	rs6041515	0.5817	0.009388	0.6284	0.006858	0.5475	0.0008975	0.7783	4.44e-05	intergenic	3TBD3;LINC0172	12634152	T	C	0.251797	0.3492	0.1282	0.2791	0.2048	0.2505
7	104873867	rs6963987	0.6469	0.009464	0.8113	0.00162	0.08094	0.6868	0.2603	0.2425	intronic	LHFPL3	104514314	C	A	0.0890575	0	0.1081	0.0938	0.1938	0.0532
9	10205088	rs12342306	-1.019	0.00956	-0.9885	0.01697	-1.244	2.66e-05	-1.495	0.000107	intronic	PTPRD	10205088	T	C	0.123403	0.0298	0.0937	0.2595	0.1153	0.0654
4	70464817	rs11932207	0.574	0.009598	0.5871	0.01125	0.6753	2.38e-05	0.6345	0.0003826	intronic	MUC7	71330534	G	A	0.248003	0.3889	0.2637	0.0628	0.2873	0.3016
4	70475444	rs11249502	0.574	0.009598	0.5871	0.01125	0.6753	2.38e-05	0.6345	0.0003826	intronic	MUC7	71341161	T	G	0.247604	0.3879	0.2622	0.0628	0.2873	0.3016
4	70476588	rs28454888	0.574	0.009598	0.5871	0.01125	0.6753	2.38e-05	0.6345	0.0003826	intronic	MUC7	71342305	A	G	0.247604	0.3879	0.2622	0.0628	0.2873	0.3016
3	717274	rs34467265	-0.7617	0.009606	-0.7567	0.0151	-0.7965	6.22e-05	-0.8615	0.0004088	ncRNA_intronic	LINC01266	758958	A	G	0.300319	0.3462	0.1787	0.5045	0.0845	0.2853
17	75710182	rs9908331	0.5217	0.009706	0.6118	0.003983	0.4506	0.001364	0.6507	0.0001064	intergenic	SAP30BP;ITGB4	73706262	C	T	0.410144	0.4643	0.4712	0.1082	0.6004	0.5235
1	13677086	rs2487648	-0.6234	0.00972	-0.6516	0.01089	-0.6549	0.0001945	-0.8329	7.29e-05	intergenic	PDPN;PRDM2	14003581	T	C	0.827276	0.7788	0.7305	0.9917	0.7704	0.7822
8	7124587	rs6605586	-0.5786	0.009766	-0.5519	0.01841	-0.6128	7.63e-05	-0.6498	0.0004327	intergenic	FA5;LOC101928	6982109	A	T	0.668331	0.4524	0.6499	0.792	0.7237	0.68
23	121375427	rs139736606	0.6394	0.009827	0.7494	0.002695	0.1112	0.6019	0.339	0.1375	intergenic	MIR3672;GRIA3	NA	NA	NA	NA	NA	NA	NA	NA	NA
6	15147374	rs9464750	0.4799	0.009904	0.5193	0.007924	0.5101	0.0001891	0.6178	8.83e-05	intergenic	LINC01108;JARID	NA	NA	NA	NA	NA	NA	NA	NA	NA
8	27540166	rs17057330	0.8625	0.009962	1.074	0.00156	0.2478	0.3771	0.4283	0.1679	intronic	EPHX2	27397683	C	T	0.0533147	0.001	0.0764	0.0507	0.0845	0.0624
10	19047049	rs12769020	0.7668	0.009984	0.8728	0.004389	0.9587	1.33e-05	1.008	4.74e-05	intergenic	ARL5B;MALRD1	19335978	G	C	0.102037	0.2063	0.0432	0.0166	0.0924	0.1616
20	61557843	rs16985218	-0.9174	0.01011	-1.109	0.006453	-0.9994	3.92e-05	-0.7777	0.005143	intronic	CDH4	60132899	A	G	0.116414	0.1329	0.0908	0.0764	0.0885	0.2004
7	47637534	rs114972253	-1.926	0.01016	-1.775	0.01788	-1.597	6.71e-05	-1.275	0.004118	intergenic	INC01447;C7orf	47677132	G	A	0.0896565	0.1052	0.0159	0.171	0.0268	0.0808
3	691907	rs2729168	-0.7566	0.01017	-0.7392	0.01706	-0.7832	7.85e-05	-0.863	0.0003728	ncRNA_intronic	LINC01266	733591	C	T	0.701278	0.6677	0.8228	0.4788	0.9175	0.728
3	701406	rs13080860	-0.7566	0.01017	-0.7392	0.01706	-0.8194	3.96e-05	-0.863	0.0003728	ncRNA_intronic	LINC01266	743090	A	G	0.289736	0.3343	0.1628	0.4864	0.0815	0.2822
13	63036299	rs532918704	-1.085	0.01019	-1.056	0.01826	-1.11	8.2e-05	-1.391	0.0002146	intergenic	4C00448;LINC00	63610432	T	A	0.00519169	0.0069	0.0043	0.0068	0.001	0.0061
15	54468244	rs12438759	1.968	0.01035	2.278	0.003842	0.7866	0.2648	1.378	0.0547	intronic	UNC13C	54760442	A	G	0.00758786	0.003	0.049	0	0.001	0
4	164886540	rs28430890	-1.421	0.01038	-1.261	0.02271	-1.577	4.62e-05	-1.617	0.001003	intronic	APELA	165807692	A	G	0.0826677	0.0804	0.0317	0.1384	0.0427	0.0869
5	51884204	rs4331856	0.5616	0.01041	0.5483	0.01538	0.4355	0.005517	0.6897	0.0001643	intergenic	ISL1;LINC02118	NA	NA	NA	NA	NA	NA	NA	NA	NA
2	188329302	rs7574262	0.4962	0.01042	0.508	0.01181	0.2614	0.06151	0.5813	0.0003729	intronic	GULP1	189194029	C	T	0.417732	0.2827	0.2536	0.5318	0.4006	0.5368
3	191363363	rs293803	0.5857	0.01046	0.6769	0.004811	0.4854	0.002527	0.7568	7.63e-05	intronic	CCDC50	191081152	C	T	0.34405	0.4405	0.245	0.4939	0.163	0.2986
8	27539846	rs10283341	0.8566	0.01049	1.062	0.001754	0.1905	0.5002	0.4168	0.1794	intronic	EPHX2	27397363	C	T	0.0543131	0.001	0.0778	0.053	0.0855	0.0624
10	59114574	rs75088866	1.629	0.01061	1.586	0.01465	1.333	0.004802	1.974	0.0001888	intergenic	LINC00844;CCEP	60874334	T	C	0.0589058	0.0317	0.0403	0.1248	0.0099	0.0613
2	76430624	rs4264582	-0.585	0.01073	-0.5357	0.02773	-0.3541	0.02017	-0.6413	0.0006573	intergenic	GCFC2;LRRTM4	76657750	G	C	0.610224	0.4921	0.6499	0.5182	0.7376	0.6973
6	151649504	rs1293960	-0.5331	0.01095	-0.6239	0.005349	-0.6164	4.77e-05	-0.6056	0.0005655	intergenic	CCDC170;ESR1	151970639	C	G	0.614816	0.629	0.7233	0.5295	0.5954	0.6585
14	62708242	rs4902176	0.5769	0.0111	0.6387	0.007864	0.5567	0.0005668	0.749	9.13e-05	exonic	KCNH5	63174960	C	T	0.221246	0.1478	0.2997	0.1936	0.3479	0.1483
20	61559449	rs71323513	-0.9092	0.01113	-1.099	0.007165	-1.032	2.99e-05	-0.7607	0.006507	intronic	CDH4	60134505	G	A	0.115016	0.1329	0.0893	0.0764	0.0885	0.1943
4	70474710	rs10003641	0.5623	0.01118	0.5757	0.01297	0.6638	3.2e-05	0.6234	0.0004813	intronic	MUC7	71340427	T	C	0.247604	0.3879	0.2622	0.0628	0.2873	0.3016
15	54508459	rs142628188	1.944	0.01126	2.252	0.004229	0.7673	0.2772	1.353	0.05926	intronic	UNC13C	54800657	A	C	0.00838658	0.004	0.0533	0	0.001	0
7	142753897	rs944498537	-0.7286	0.01135	-0.8129	0.007933	-0.8494	4.44e-05	-0.9618	0.0001051	intronic	TCAF2	NA	NA	NA	NA	NA	NA	NA	NA	NA
20	61549454	rs35587576	-0.9438	0.01156	-1.033	0.01209	-1.087	3.17e-05	-0.7897	0.006417	intronic	CDH4	60124510	CT	C	0.114018	0.1032	0.0778	0.1104	0.0795	0.1912
10	59097089	rs78721894	1.612	0.01158	1.573	0.01583	1.364	0.004271	1.988	0.0001869	intergenic	LINC00844;CCEP	60856849	A	G	0.0543131	0.0317	0.0432	0.1044	0.0119	0.0613
3	717236	rs9843857	-0.7388	0.01164	-0.7253	0.01883	-0.7974	6.23e-05	-0.8465	0.0004909	ncRNA_intronic	LINC01266	758920	A	G	0.305911	0.3462	0.1801	0.5242	0.0845	0.2863
5	132291708	rs6878654	-0.5015	0.01166	-0.6327	0.002978	-0.5898	4.93e-05	-0.6353	0.0001537	intergenic	PDLIM4;SLC22A	131627401	T	A	0.31849	0.2817	0.4885	0.1082	0.4583	0.3763
3	695585	rs2729171	-0.7425	0.01168	-0.7431	0.01639	-0.7932	7.34e-05	-0.8213	0.0007505	ncRNA_intronic	LINC01266	737269	T	A	0.70627	0.6935	0.83	0.4818	0.9165	0.7188
15	22559385	rs200005565	-1.01	0.01177	-0.9429	0.02662	-1.03	0.0001775	-1.346	7.29e-05	ncRNA_intronic	HERC2P2	NA	NA	NA	NA	NA	NA	NA	NA	NA
7	60950830	rs1251710946	-0.9889	0.01184	-1.224	0.007426	-0.9249	0.0003515	-1.442	7.82e-05	intergenic	NONE;NONE	NA	NA	NA	NA	NA	NA	NA	NA	NA
12	70938923	rs1350401734	-0.9182	0.01187	-0.7477	0.04235	-1.023	7.92e-05	-0.8202	0.0005762	intergenic	PTPRR;TSPAN8	NA	NA	NA	NA	NA	NA	NA	NA	NA
1	186224086	rs584976	0.5461	0.01202	0.6489	0.005018	0.6178	0.0001249	0.7285	8.64e-05	intergenic	HMCN1;PRG4	186193218	C	T	0.21865	0.1528	0.3141	0.0726	0.3608	0.2699
4	70565458	rs28833600	0.5537	0.01205	0.5409	0.02044	0.6136	9.3e-05	0.6594	0.0002343	intergenic	AMTN;AMBN	71431175	C	T	0.250998	0.3661	0.2392	0.0968	0.2594	0.3405
3	691641	rs2729167	-0.739	0.01217	-0.7392	0.01706	-0.7765	9.05e-05	-0.854	0.0004297	ncRNA_intronic	LINC01266	733325	C	T	0.701478	0.6667	0.8228	0.4796	0.9165	0.7301
3	691652	rs2627944	-0.739	0.01217	-0.7392	0.01706	-0.7765	9.05e-05	-0.854	0.0004297	ncRNA_intronic	LINC01266	733336	G	T	0.700879	0.6667	0.8228	0.4773	0.9165	0.7301
3	692305	rs1611004	-0.739	0.01217	-0.7392	0.01706	-0.7765	9.05e-05	-0.854	0.0004297	ncRNA_intronic	LINC01266	733989	TAGTC	T	0.701078	0.6696	0.8228	0.4781	0.9175	0.726
10	112802685	rs10509965	-0.5719	0.01219	-0.7921	0.002118	-0.429	0.005992	-0.764	0.0001049	intronic	VTI1A	114562444	T	C	0.179313	0.256	0.2205	0.0537	0.2565	0.1616
18	10515702	rs3910734	0.4785	0.01219	0.5033	0.01167	0.5694	4.72e-05	0.6088	0.0001737	intergenic	APCDD1;NAPG	10515699	C	T	0.372804	0.3353	0.4755	0.1036	0.5288	0.5419
11	27705368	rs2030324	0.4704	0.01229	0.5955	0.003304	0.2797	0.03703	0.5323	0.0007477	intronic	BDNF	27726915	A	G	0.434105	0.4147	0.5677	0.4342	0.4751	0.317
11	27710436	rs7934165	0.4704	0.01229	0.5955	0.003304	0.2797	0.03703	0.5323	0.0007477	intronic	BDNF	27731983	G	A	0.433706	0.4147	0.5677	0.4357	0.4712	0.317

4	174383470	rs1165551416	-0.795	0.01241	-0.8599	0.01172	-0.6566	0.001808	-1.024	0.0001364	intergenic	CEP44;MIR4276	NA	NA	NA	NA	NA	NA	NA	NA	NA
10	112792633	rs10787462	-0.495	0.01247	-0.6058	0.004511	-0.4067	0.003937	-0.6575	0.0001133	intronic	VTI1A	114552392	G	A	0.560703	0.5585	0.4841	0.4917	0.5934	0.6769
4	70463773	rs1109501	0.5504	0.01254	0.5871	0.01125	0.6678	2.85e-05	0.623	0.0004741	intronic	MUC7	71329490	G	A	0.248003	0.3889	0.2637	0.0628	0.2873	0.3016
4	130550633	rs877892	-0.6089	0.01256	-0.6397	0.01349	-0.4	0.01856	-0.7513	0.0003527	intergenic	INC02479;SNHG2	131471788	T	A	0.180711	0.0308	0.1931	0.1195	0.3976	0.1861
3	191368621	rs2307647	0.5762	0.01269	0.6714	0.005784	0.4714	0.003389	0.767	7.09e-05	intronic	CCDC50	191086410	ATTC	A	0.354034	0.4385	0.2478	0.5333	0.161	0.2986
13	22338487	rs7986375	0.7173	0.01297	0.5311	0.08251	0.8174	9.38e-05	0.4864	0.053	intergenic	NC00540;LINC006	22912626	A	G	0.190296	0.131	0.1686	0.348	0.1143	0.1319
10	112793203	rs10787464	-0.4921	0.01315	-0.6087	0.004078	-0.4056	0.004084	-0.6457	0.0001328	intronic	VTI1A	114552962	G	A	0.525759	0.5675	0.4611	0.3888	0.5646	0.6738
3	705264	rs1484518	-0.7264	0.01344	-0.7833	0.01253	-0.7752	9.11e-05	-0.8492	0.0004541	ncRNA_intronic	LINC01266	746948	G	T	0.703275	0.6667	0.8242	0.4955	0.9155	0.7178
5	135488858	rs76860778	1.176	0.0137	1.453	0.003345	0.4302	0.305	0.8414	0.0568	intergenic	TIFAB;NEUROG1	134824548	G	A	0.0205671	0.0496	0.0605	0.0015	0.003	0.0061
5	135492508	rs75583160	1.176	0.0137	1.453	0.003345	0.4891	0.2423	0.8418	0.05671	intergenic	TIFAB;NEUROG1	134828198	T	A	0.0199681	0.0476	0.0605	0.0015	0.003	0.0051
5	135490238	rs117155811	1.174	0.01381	1.451	0.003384	0.4274	0.3082	0.8409	0.05699	intergenic	TIFAB;NEUROG1	134825928	A	G	0.0201677	0.0476	0.0605	0.0015	0.003	0.0061
5	52968323	rs246509	0.4848	0.01386	0.4916	0.01674	0.6244	3.69e-05	0.6701	7.3e-05	intergenic	ITGA1;ITGA2	52264153	A	C	0.293131	0.2728	0.2421	0.2852	0.2853	0.3691
2	188282184	rs147312864	0.4674	0.01391	0.4728	0.01786	0.285	0.03688	0.609	0.0001702	ncRNA_intronic	LINC01090	189146911	AAAG	A	0.46865	0.2857	0.281	0.643	0.4334	0.591
2	188282188	rs55676189	0.4674	0.01391	0.4728	0.01786	0.285	0.03688	0.609	0.0001702	ncRNA_intronic	LINC01090	189146915	A	T	0.46865	0.2857	0.281	0.6437	0.4324	0.591
11	67780081	rs374059167	-0.6545	0.01392	-0.7135	0.01081	-0.8193	2.04e-05	-0.7697	0.0005396	intergenic	LDH3B2;FAM86C	NA	NA	NA	NA	NA	NA	NA	NA	NA
12	121138849	rs656612	-0.5252	0.01395	-0.6354	0.006356	-0.4105	0.006035	-0.7087	0.00014	intronic	P2RX7	121576652	C	A	0.565495	0.6091	0.7738	0.2935	0.7177	0.5838
12	107538402	rs11113385	0.5018	0.014	0.6064	0.004729	0.6011	5.05e-05	0.3639	0.03242	intronic	BTBD11	107932179	A	G	0.359625	0.3363	0.4481	0.4644	0.3002	0.2403
13	22339188	rs34703184	0.7089	0.01408	0.5197	0.08926	0.8244	6.72e-05	0.4766	0.05766	intergenic	NC00540;LINC006	22913327	G	A	0.190695	0.131	0.1686	0.351	0.1133	0.1309
13	22341668	rs12873807	0.7089	0.01408	0.5197	0.08926	0.8244	6.72e-05	0.4766	0.05766	intergenic	NC00540;LINC006	22915807	A	G	0.191294	0.131	0.1686	0.3525	0.1143	0.1309
6	146499391	rs9403791	0.5358	0.01427	0.5798	0.01073	0.2719	0.07663	0.6293	0.0004565	intergenic	GRM1;RAB32	146820527	A	G	0.320887	0.5754	0.2176	0.1657	0.2018	0.4642
12	107532305	rs11113380	0.5004	0.01438	0.605	0.004883	0.5963	5.97e-05	0.3614	0.03384	intronic	BTBD11	107926082	A	T	0.358626	0.3363	0.4467	0.4644	0.2982	0.2382
18	9067947	rs12457383	0.6906	0.01439	0.7887	0.006364	-0.04119	0.864	0.2698	0.2975	intergenic	MTCL1;NDUFV2	9067945	C	T	0.0756789	0.0784	0.1037	0.0333	0.0626	0.1237
12	121149550	rs208287	-0.5186	0.01464	-0.5887	0.01025	-0.4226	0.004739	-0.7088	0.0001469	intronic	P2RX7	121587353	T	C	0.471645	0.3948	0.2349	0.8154	0.2833	0.4479
3	706237	rs2729181	-0.7104	0.01479	-0.7392	0.01706	-0.7994	5.92e-05	-0.886	0.0002918	ncRNA_intronic	LINC01266	747921	C	T	0.69988	0.6667	0.8242	0.4826	0.9165	0.7168
5	132339031	rs4551060	-0.6945	0.01485	-0.7319	0.01588	-0.837	8.23e-05	-0.7677	0.001581	ncRNA_intronic	MIR3936HG	131674724	G	A,T	658946,0.00119	0,0	0.1196,0	0.0272,0.0045	0.172,0	0.0389,0
19	9563158	rs78449212	1.165	0.01497	1.288	0.007298	1.112	0.004547	1.474	0.0002972	UTR3	ZNF121	9673834	C	T	0.0409345	0.0139	0.0346	0.0015	0.0636	0.1033
22	36455160	rs139961	-0.5335	0.01499	-0.8621	0.0006106	-0.1493	0.3311	-0.4245	0.01969	intergenic	MYH9;TXN2	36851207	C	T	0.56869	0.2044	0.7666	0.8321	0.6998	0.3129
11	128749880	rs626816	0.4784	0.01506	0.5664	0.006779	0.5721	6.25e-05	0.7071	3.14e-05	intronic	FLI1	128619775	G	A	0.516573	0.4385	0.402	0.5469	0.5149	0.6391
23	119750268	rs2782214	0.468	0.0154	0.7576	0.0005694	0.06447	0.6423	0.2275	0.1536	intergenic	SEPTIN6;SOWAHI	NA	NA	NA	NA	NA	NA	NA	NA	NA
18	10514709	rs612452	0.4708	0.01543	0.4837	0.01754	0.5606	8.24e-05	0.6076	0.0002462	intergenic	APCDD1;NAPG	10514706	G	A	0.612021	0.6567	0.5101	0.885	0.4344	0.4519
7	56335130	rs1456703276	-0.7156	0.01575	-0.8557	0.007466	-0.686	0.0007568	-1.003	8.07e-05	intergenic	NUPR2;LOC65022	NA	NA	NA	NA	NA	NA	NA	NA	NA
8	7116987	rs7831556	-0.5283	0.01576	-0.4884	0.03263	-0.5991	9.16e-05	-0.6229	0.0005806	intergenic	FA5;LOC101928	6974509	G	C	0.66873	0.4514	0.6499	0.7905	0.7276	0.681
19	1037767	rs78386506	-0.8293	0.01612	-0.7825	0.02783	-0.9321	6.09e-05	-0.8128	0.002774	exonic	CNN2	NA	NA	NA	NA	NA	NA	NA	NA	NA
12	107537277	rs7960037	0.4922	0.01617	0.5979	0.005441	0.6025	5.24e-05	0.3542	0.03776	intronic	BTBD11	107931054	C	A	0.358427	0.3353	0.4467	0.4637	0.2992	0.2382
12	107532887	rs78544106	0.4919	0.01624	0.597	0.005557	0.6053	4.76e-05	0.3571	0.03611	intronic	BTBD11	107926664	TA	T	0.359026	0.3373	0.4481	0.4652	0.2992	0.2362
18	74586341	rs4892248	-0.492	0.01628	-0.5788	0.00737	-0.5857	6.24e-05	-0.5937	0.0004337	UTR3	CNDP1	72253577	G	T	0.465855	0.5893	0.6326	0.1127	0.5865	0.5736
12	121150742	rs169632	-0.5088	0.0163	-0.5777	0.01146	-0.4197	0.004922	-0.7096	0.0001364	intronic	P2RX7	121588545	T	C	0.472045	0.3929	0.2349	0.8177	0.2843	0.4479
10	112793838	rs10885383	-0.4716	0.01636	-0.5568	0.007736	-0.4333	0.00204	-0.6705	6.84e-05	intronic	VTI1A	114553597	G	A	0.553714	0.5446	0.4798	0.4924	0.5795	0.6718
8	40197886	rs77472320	-0.7674	0.0164	-0.6497	0.04655	-0.9907	2.19e-05	-0.8065	0.002661	intergenic	TCIM;SIRLNT	40055405	C	A	0.146965	0.0298	0.1023	0.2345	0.1322	0.1963
10	59107984	rs76350599	1.904	0.01646	2.066	0.008838	2.107	0.001285	2.646	0.0001597	intergenic	LINC00844;CCEPI	60867744	G	A	0.0315495	0.0317	0.0245	0.0295	0.0099	0.0613
1	13669583	rs2495042	-0.5849	0.0166	-0.6226	0.01685	-0.6335	0.0003298	-0.8707	5.16e-05	intergenic	PDPN;PRDM2	13996078	A	G	0.828275	0.7629	0.7363	0.9917	0.7823	0.7873
12	121139975	rs671684	-0.5153	0.0168	-0.6235	0.008011	-0.4494	0.003001	-0.7337	0.0001041	intronic	P2RX7	121577778	G	A	0.468051	0.3899	0.2334	0.8306	0.2823	0.4162
23	119750257	rs2528718	0.4619	0.0168	0.7657	0.0005787	0.07496	0.5901	0.2255	0.158	intergenic	SEPTIN6;SOWAHI	NA	NA	NA	NA	NA	NA	NA	NA	NA
5	135483695	rs61563823	1.068	0.01691	1.31	0.004483	0.3713	0.3117	0.684	0.09115	intergenic	TIFAB;NEUROG1	134819385	A	G	0.0501198	0.0476	0.0663	0.1104	0.004	0.0072
5	135491566	rs73791407	1.068	0.01691	1.31	0.004483	0.4187	0.2522	0.684	0.09115	intergenic	TIFAB;NEUROG1	134827256	C	G	0.058107	0.0476	0.0692	0.1399	0.004	0.0061
17	1169192	rs67861065	0.4486	0.01693	0.5012	0.01141	0.22	0.1077	0.5347	0.0009295	intronic	ABR	1072486	A	T	0.28095	0.2222	0.4006	0.0295	0.501	0.3701
5	135483280	rs73791403	1.068	0.01697	1.31	0.004499	0.3714	0.3115	0.6835	0.09137	intergenic	TIFAB;NEUROG1	134818970	T	C	0.0501198	0.0476	0.0663	0.1104	0.004	0.0072
5	135488018	rs17168438	1.068	0.01697	1.31	0.004499	0.3714	0.3115	0.6835	0.09137	intergenic	TIFAB;NEUROG1	134823708	A	C	0.0501198	0.0476	0.0663	0.1104	0.004	0.0072
5	135492615	rs17168462	1.068	0.01697	1.31	0.004499	0.4186	0.2522	0.6835	0.09137	intergenic	TIFAB;NEUROG1	134828305	G	A	0.0515176	0.0486	0.0706	0.1135	0.004	0.0061
7	100953133	rs1419524538	0.7193	0.01697	0.5408	0.07679	0.9282	1.8e-05	0.8903	0.0004132	exonic	MUC3A	NA	NA	NA	NA	NA	NA	NA	NA	NA
5	135491536	rs73791406	1.067	0.01704	1.309	0.004516	0.4176	0.2533	0.6824	0.0918	intergenic	TIFAB;NEUROG1	134827226	T	C	0.0497204	0.0476	0.0677	0.1089	0.004	0.0061
5	135491545	rs77076864	1.067	0.01704	1.309	0.004516	0.4176	0.2533	0.6824	0.0918	intergenic	TIFAB;NEUROG1	134827235	C	T	0.0579073	0.0476	0.0692	0.1392	0.004	0.0061
19	30667560	rs58769168	0.6098	0.01705	0.7813	0.003102	0.08599	0.6566	0.2547	0.2462	intronic	ZNF536	31158467	A	G	0.171526	0.0625	0.1052	0.3389	0.1928	0.0828
12	107519515	rs759520	0.4876	0.01719	0.5926	0.005934	0.6057	4.76e-05	0.3565	0.03651	intronic	BTBD11	107913292	G	A	0.371206	0.3373	0.451	0.4622	0.3002	0.2996
7	60950862	rs1185857187	-0.6919	0.01721	-0.7176	0.02075	-0.8291	6.95e-05	-0.9587	0.0001384	intergenic	NONE;NONE	NA	NA	NA	NA	NA	NA	NA	NA	NA
3	2715593	rs11711025	0.5899	0.0173	0.6937	0.006532	0.7917	2.67e-05	0.8043	0.0001285	intronic	CNTN4	2757277	C	G	0.178914	0.1706	0.1398	0.2126	0.1382	0.2117
1	80411027	rs79428324	0.9586	0.01746	1.179	0.004486	-0.1264	0.673	0.2558	0.4559	intergenic	ADGRL4;LINC0178	80876712	T	C	0.0804712	0.1637	0.0418	0.1218	0.0199	0.0286
10	59206493	rs2440867	1.096	0.01747	1.216	0.009961	0.673	0.05031	1.3	0.001092	intronic	PHYHIPL	60966253	G	A	0.109026	0.0337	0.0663	0.2874	0.0139	0.0736
22	36456643	rs139965	-0.5205	0.01747	-0.8477	0.0007409	-0.1397	0.3622	-0.4151	0.02245	intergenic	MYH9;TXN2	36852690	C	T	0.567891	0.2034	0.7651	0.8313	0.6998	0.3119
3	694432	rs2729169	-0.6936	0.01748	-0.7392	0.01706	-0.7786	8.43e-05	-0.8235	0.0006206	ncRNA_intronic	LINC01266	736116	A	G	0.700679	0.6667	0.8256	0.4781	0.9175	0.7249
10	59154293	rs16913241	1.6	0.01749	1.524	0.02702	1.406	0.005767	2.038	0.0002414	intergenic	LINC00844;CCEPI	60914053	T	C	0.0583067	0.0317	0.0375	0.1225	0.0099	0.0634
10	59099824	rs16913129	1.65	0.0175	1.794	0.008778	1.627	0.0024	2.201	0.0001561	intergenic	LINC00844;CCEPI	60859584	G	A	0.048722	0.0317	0.0375	0.0877	0.0099	0.0613
1	236754528	rs2297858	-0.4639	0.01784	-0.5184	0.01297	-0.5457	0.0001317	-0.6968	3.74e-05	intronic	ACTN2	236917828	T	C	0.371406	0.3938	0.4899	0.0363	0.6521	0.4284
10	59100667	rs145393994	1.64	0.01802	1.785	0.009027	1.624	0.002456	2.189	0.0001675	intergenic	LINC00844;CCEPI	60860427	G	A	0.0501198	0.0317	0.0375	0.093	0.0099	0.0613
17	68910738	rs1080499	0.8413	0.01834	0.6954	0.06781	1.097	8.33e-05	0.8767	0.005637	intronic	ABCA8	66906879	T	G	0.170527	0.1944	0.0504	0.4327	0.0408	0.0102
6	166201879	rs13198053	-0.7072	0.01839	-0.86	0.00993	-0.8524	6.88e-05	-0.9754	0.0002731	intergenic	CDAT;LOC101929	166615367	A	C	0.19349	0.1181	0.232	0.2874	0.1352	0.1769
6	146503561	rs9485092	0.5102	0.01841	0.5384	0.01626	0.2703	0.07594	0.5894	0.0008921	intergenic	GRM1;RAB32	146824697	C	T	0.677516	0.4246	0.7795	0.8328	0.7922	0.5378
4	70549188	rs28770292	0.482	0.01842	0.4365	0.04186	0.6188	2.02e-05	0.5972	0.0003071	intergenic	AMTN;AMBN	71414905	G	C	0.635982	0.5923	0.6859	0.6097	0.662	0.6544
4	70549393	rs28867378	0.482	0.01842	0.4365	0.04186	0.6188	2.02e-05	0.5972	0.0003071	intergenic	AMTN;AMBN	71415110	G	A	0.635982	0.5923	0.6859	0.6097	0.662	0.6544
12	107705505	rs148638337	0.9869	0.01876	1.101	0.01055	0.9189	0.004218	1.354	9.12e-05	intronic	PWP1	108099282	T	C	0.0523163	0.1071	0.0274	0.0129	0.0348	0.0849
7	96059863	rs6965020	0.5441	0.01886	0.4379	0.08056	0.7061	2.38e-05	0.5023	0.008759	intronic	DYNC1I1	95689175	C	T	0.227436	0.3075	0.2046	0.2912	0.2356	0.0665
6	44066665	rs11375670	-0.5183	0.01888	-0.6009	0.01448	-0.6281	7.68e-05	-0.5794	0.001989	ncRNA_intronic	LOC101929705	44034402	T	TCC	0.416334	0.3254	0.4424	0.5938	0.3857	0.2832
5	135489471	rs73791405	1.047	0.01892	1.31	0.004499	0.365	0.3198	0.6726	0.09645	intergenic	TIFAB;NEUROG1	134825161	G	C	0.0499201	0.0476	0.0663	0.1097	0.004	0.0072
12	121144437	rs183283	-0.5025	0.01902	-0.6047	0.009509	-0.4456	0.00321	-0.7189	0.0001298	intronic	P2RX7	121582240	G	A	0.46845	0.3899	0.2334	0.8328	0.2823	0.4151
12	121145948	rs169631	-0.4977	0.01923	-0.6148	0.008394	-0.4576	0.002471	-0.7267	0.0001061	intronic	P2RX7	121583751	C	G	0.476438	0.3899	0.2334	0.8328	0.2823	0.456
7	96072036	rs7796183	0.5375	0.01931	0.4436	0.07312	0.6668	7.27e-05	0.4548	0.01786	intronic	DYNC1I1	95701348	C	A	0.207069	0.3085	0.2003	0.208	0.2376	0.0746
4	3549634	rs62275373	-0.6518	0.02015	-0.6223	0.03371	-0.7973	7.93e-05	-0.9746	5.66e-05	intergenic	RPAP1;LINC0095	NA	NA	NA	NA	NA	NA	NA	NA	NA
12	107516818	rs1558782	0.47	0.02025	0.5365	0.01212	0.5792	9.16e-05	0.349	0.03966	intronic	BTBD11	107910595	C	G	0.382588	0.3373	0.4539	0.5045	0.3002	0.2986
10	126953876	rs985873474	-0.4875	0.02026	-0.4015	0.0649	-0.6212	5.03e-05	-0.3624	0.03439	intronic	DOCK1	NA	NA	NA	NA	NA	NA	NA	NA	NA
12	107508855	rs11113355	0.4756	0.02035	0.5805	0.006991	0.6198	3.54e-05	0.3741	0.0296	intronic	BTBD11	107902632	T	G	0.403554	0.38	0.4611	0.5477	0.2992	0.2996
19	30668007	rs17543087	0.5857	0.02059	0.7776	0.003085	0.08117	0.6733	0.2458	0.2602	intronic	ZNF536	31158914	C	G	0.171925	0.0635	0.1052	0.3396	0.1928	0.0828
23	25687640	rs5986260	0.4485	0.02098	0.5088	0.01395	0.368	0.006693	0.6446	8.03e-05	intergenic	ARX;MAGEB18	NA	NA	NA	NA	NA	NA	NA	NA	NA
4	70555195	rs138049929	0.472	0.02111	0.4265	0.04705	0.6094	2.66e-05	0.5881	0.0003803	intergenic	AMTN;AMBN	71420912	A	G	0.631789	0.5923	0.6873	0.6089	0.662	0.6329
10	59128789	rs78542399	1.548	0.0212	1.686	0.01093	1.459	0.003717	2.064	0.0001968	intergenic	LINC00844;CCEPI	60888549	T	C	0.0567093	0.0317	0.0375	0.118	0.0099	0.0613
6	151677008	rs1293941	0.5198	0.02129	0.5163	0.02907	0.4864	0.004249	0.6911	0.0002833	intronic	ESR1	151998143	T	G	0.150359	0.0585	0.1527	0.0371	0.2833	0.2597
7	58088579	rs1382946423	-0.6748	0.02137	-0.5564	0.06526	-0.851	4.75e-05	-0.7888	0.001246	intergenic	ZNF716;NONE	NA	NA	NA	NA	NA	NA	NA	NA	NA
2	188277029	rs7598279	0.4333	0.02175	0.4633	0.02041	0.2727	0.04635	0.6014	0.0002121	ncRNA_intronic	LINC01090	189141756	T	C	0.468051	0.2857	0.2752	0.6452	0.4314	0.591
23	119754661	rs2528721	0.4469	0.02236	0.7632	0.0007157	0.05223	0.7158	0.1928	0.2344	intergenic	SEPTIN6;SOWAHI	NA	NA	NA	NA	NA	NA	NA	NA	NA
23	119756640	rs2782221	0.4175	0.02339	0.6277	0.001698	0.07937	0.564	0.1977	0.2072	intergenic	SEPTIN6;SOWAHI	NA	NA	NA	NA	NA	NA	NA	NA	NA
12	107518376	rs9669581	0.4633	0.02342	0.568	0.008206	0.5939	6.78e-05	0.3498	0.04055	intronic	BTBD11	107912153	A	G	0.382588	0.3373	0.4539	0.5045	0.3002	0.2986
6	151680823	rs851999	0.5114	0.02354	0.5086	0.03174	0.4639	0.006258	0.6847	0.0003202	intronic	ESR1	152001958	G	T	0.155152	0.0595	0.1542	0.0371	0.2833	0.2822
7	56335124	rs980899294	-0.6627	0.0236	-0.79	0.01201	-0.6765	0.0008362	-0.989	8.73e-05	intergenic	NUPR2;LOC65022	NA	NA	NA	NA	NA	NA	NA	NA	NA
6	103387583	rs9390997	-0.4926	0.02373	-0.5181	0.02637	-0.6211	8.15e-05	-0.7507	7.67e-05	intergenic	GRIK2;HACE1	103835458	T	C	0.377796	0.3175	0.3141	0.6346	0.2962	0.2219
5	52967867	rs9791048	0.4486	0.0244	0.4916	0.01674	0.6171	4.88e-05	0.6612	9.73e-05	intergenic	ITGA1;ITGA2	52263697	T	G	0.706669	0.7272	0.7565	0.7148	0.7147	0.6309
5	52975417	rs246522	0.4486	0.0244	0.4916	0.01674	0.6171	4.88e-05	0.6612	9.73e-05	intergenic	ITGA1;ITGA2	52271247	G	T	0.294129	0.2738	0.2435	0.2867	0.2853	0.3701
5	52977549	rs246525	0.4486	0.0244	0.4916	0.01674	0.6171	4.88e-05	0.6612	9.73e-05	intergenic	ITGA1;ITGA2	52273379	G	A	0.29393	0.2738	0.2435	0.2867	0.2853	0.3691
5	52979340	rs2067320	0.4486	0.0244	0.4916	0.01674	0.6171	4.88e-05	0.6612	9.73e-05	intergenic	ITGA1;ITGA2	52275170	TA	T	0.29393	0.2738	0.2435	0.2867	0.2853	0.3691
10	19052704	rs35763766	0.6686	0.02446	0.7638	0.01245	0.8931	3.75e-05	0.9292	0.0001413	intronic	MALRD1	19341633	A	AC	0.103235	0.2054	0.0432	0.0151	0.0974	0.1656
6	151676694	rs1738380	0.5052	0.02452	0.5223	0.02713	0.4867	0.004165	0.6877	0.0002951	intronic	ESR1	151997829	C	T	0.150359	0.0585	0.1527	0.0371	0.2833	0.2597

12	107508991	rs4964585	0.463	0.02457	0.5661	0.008795	0.6168	3.95e-05	0.368	0.03291	intronic	BTBD11	107902768	A	T	0.403355	0.38	0.4611	0.5469	0.2992	0.2996
10	59163110	rs78718325	1.02	0.02458	1.14	0.01424	0.6869	0.04173	1.332	0.0006083	intergenic	LINC00844;CCEPI	60922870	G	A	0.101438	0.0337	0.0634	0.2579	0.0169	0.0736
7	56335123	rs1479055336	-0.6577	0.02467	-0.7862	0.01246	-0.6739	0.0008782	-0.9855	9.26e-05	intergenic	NUPR2;LOC65022	NA	NA	NA	NA	NA	NA	NA	NA	NA
8	115067211	rs1353277	-0.5105	0.02539	-0.6508	0.00796	-0.3396	0.03737	-0.6264	0.001191	intergenic	CSMD3;TRPS1	116079440	A	G	0.517772	0.7937	0.4697	0.6021	0.2386	0.4407
7	96072321	rs7796656	0.5034	0.0255	0.4111	0.09053	0.6465	8.58e-05	0.4351	0.0203	intronic	DYNC1I1	95701633	C	G	0.245807	0.3145	0.2133	0.3434	0.2386	0.0736
12	107520047	rs759519	0.4555	0.02602	0.5526	0.01008	0.5926	6.73e-05	0.3367	0.04823	intronic	BTBD11	107913824	A	G	0.370607	0.3373	0.4481	0.4622	0.3002	0.2986
12	107520364	rs1861586	0.4555	0.02602	0.5526	0.01008	0.5926	6.73e-05	0.3367	0.04823	intronic	BTBD11	107914141	C	T	0.370607	0.3373	0.4481	0.4622	0.3002	0.2986
4	12012227	rs10939394	0.4298	0.02605	0.4951	0.01412	0.3977	0.003937	0.6364	8.11e-05	intergenic	IR12113;LINC022	12013851	C	G	0.316094	0.5298	0.3761	0.1074	0.3479	0.3027
7	96075324	rs11293151	0.4904	0.02611	0.4035	0.08889	0.6545	5.83e-05	0.4393	0.01728	intronic	DYNC1I1	95704636	TG	T	0.257987	0.3145	0.2205	0.388	0.2356	0.0736
5	52969926	rs246510	0.443	0.02632	0.4863	0.018	0.6019	7.38e-05	0.6386	0.0001634	intergenic	ITGA1;ITGA2	52265756	G	A	0.294329	0.2718	0.2464	0.2882	0.2853	0.3691
5	52972252	rs246512	0.4432	0.02632	0.4869	0.01789	0.6038	6.96e-05	0.639	0.0001616	intergenic	ITGA1;ITGA2	52268082	G	A	0.29353	0.2718	0.2435	0.2867	0.2853	0.3691
1	4111768	rs77516436	-1.795	0.02698	-1.631	0.04666	-2.166	2.42e-05	-1.903	0.001524	intergenic	NC01346;LINC017	4171828	G	A	0.0467252	0.1389	0.0029	0.0575	0.005	0.0112
1	4109454	rs16840763	-1.794	0.02704	-1.63	0.04678	-2.165	2.45e-05	-1.903	0.001531	intergenic	NC01346;LINC017	4169514	G	C	0.048722	0.1389	0.0029	0.0651	0.005	0.0112
1	4107160	rs16840755	-1.794	0.02708	-1.63	0.04685	-2.164	2.45e-05	-1.902	0.001534	intergenic	NC01346;LINC017	4167220	C	A	0.0497204	0.1409	0.0029	0.0673	0.005	0.0112
1	4107489	rs79863961	-1.794	0.02708	-1.63	0.04685	-2.164	2.45e-05	-1.902	0.001534	intergenic	NC01346;LINC017	4167549	G	C	0.0493211	0.1409	0.0029	0.0658	0.005	0.0112
1	4109949	rs75226241	-1.794	0.02708	-1.63	0.04685	-1.972	5.6e-05	-1.852	0.002068	intergenic	NC01346;LINC017	4170009	C	T	0.048722	0.1389	0.0029	0.0651	0.005	0.0112
1	4110019	rs115631479	-1.794	0.02708	-1.63	0.04685	-1.999	4.37e-05	-1.902	0.001534	intergenic	NC01346;LINC017	4170079	G	A	0.0491214	0.1399	0.0029	0.0651	0.006	0.0112
1	4112557	rs16840780	-1.794	0.02708	-1.63	0.04685	-2.164	2.45e-05	-1.902	0.001534	intergenic	NC01346;LINC017	4172617	C	T	0.0473243	0.1409	0.0029	0.0582	0.005	0.0112
1	4113781	rs10492945	-1.794	0.02708	-1.63	0.04685	-1.978	5.33e-05	-1.863	0.001946	intergenic	NC01346;LINC017	4173841	A	G	0.0489217	0.1399	0.0029	0.0651	0.005	0.0112
1	4114233	rs149610995	-1.794	0.02708	-1.63	0.04685	-1.978	5.33e-05	-1.863	0.001946	intergenic	NC01346;LINC017	4174293	GT	AT,G	0599042,0.0497	0,0.1419	0,0.0029	0.0023,0.0643	0,0.007	0,0.0123
1	4115359	rs79817596	-1.794	0.02708	-1.63	0.04685	-2.164	2.45e-05	-1.902	0.001534	intergenic	NC01346;LINC017	4175419	C	T	0.0441294	0.1399	0.0029	0.0454	0.005	0.0133
1	4116607	rs78451862	-1.794	0.02708	-1.63	0.04685	-2.164	2.45e-05	-1.902	0.001534	intergenic	NC01346;LINC017	4176667	C	A	0.0489217	0.1399	0.0029	0.0651	0.005	0.0112
1	4117838	rs16840802	-1.794	0.02708	-1.63	0.04685	-2.164	2.45e-05	-1.902	0.001534	intergenic	NC01346;LINC017	4177898	G	A	0.0469249	0.1399	0.0029	0.0575	0.005	0.0112
5	52974094	rs57191324	0.4415	0.02747	0.4842	0.01907	0.629	3.83e-05	0.6753	7.72e-05	intergenic	ITGA1;ITGA2	52269924	TG	T	0.286542	0.2738	0.2392	0.2602	0.2853	0.3701
4	12054751	rs5011971	0.4335	0.0282	0.5133	0.01306	0.3865	0.00689	0.621	0.0001611	intergenic	IR12113;LINC022	12056375	C	T	0.284145	0.5238	0.3545	0.034	0.3181	0.2904
20	7937199	rs2294298	0.7134	0.0282	0.6076	0.07911	0.9246	7.72e-05	0.771	0.003919	intronic	HAO1	7917846	C	G	0.191294	0.3343	0.0461	0.1377	0.0646	0.3497
4	70549652	rs4092435	0.4376	0.02837	0.3957	0.05835	0.5668	7.14e-05	0.5536	0.0006682	intergenic	AMTN;AMBN	71415369	C	A	0.612021	0.5923	0.6816	0.5212	0.662	0.6544
4	122582337	rs10212786	-0.5393	0.02854	-0.4916	0.05394	-0.7024	9.97e-05	-0.7297	0.0006319	intergenic	IL2;IL21	123503492	C	G	0.186901	0.1131	0.1686	0.2239	0.2704	0.1401
12	41313268	rs75586953	0.9506	0.02855	1.033	0.02073	0.9307	0.004267	1.292	0.0002324	intronic	PDZRN4	41707070	A	T	0.0926518	0.1875	0.0173	0.0454	0.0318	0.1748
2	188262692	rs12693489	0.4072	0.02861	0.4214	0.03074	0.276	0.03985	0.5801	0.0002771	ncRNA_intronic	LINC01090	189127419	T	C	0.479233	0.2877	0.3084	0.6172	0.4841	0.6063
4	12022097	rs16886565	0.4229	0.02883	0.4826	0.01671	0.3536	0.01217	0.5994	0.0002241	intergenic	IR12113;LINC022	12023721	G	A	0.288339	0.5188	0.3516	0.0545	0.3201	0.2894
7	100953117	rs1215688799	0.6608	0.02888	0.4799	0.1178	0.8746	6.38e-05	0.9433	0.0003073	exonic	MUC3A	NA	NA	NA	NA	NA	NA	NA	NA	NA
20	7919957	rs62200774	0.7261	0.02951	0.5987	0.09293	0.9636	3.99e-05	0.7863	0.003143	intronic	HAO1	7900604	A	G	0.182907	0.3294	0.0476	0.1256	0.0646	0.3272
20	7926739	rs35502451	0.7261	0.02951	0.5987	0.09293	0.9582	4.98e-05	0.7674	0.004438	intronic	HAO1	7907386	C	A	0.182508	0.3284	0.0476	0.1248	0.0636	0.3282
20	7926938	rs13040468	0.7261	0.02951	0.5987	0.09293	0.9582	4.98e-05	0.7674	0.004438	intronic	HAO1	7907585	A	C	0.182907	0.3284	0.0476	0.1248	0.0646	0.3292
20	7935055	rs62198476	0.7261	0.02951	0.5987	0.09293	0.9582	4.98e-05	0.7674	0.004438	intronic	HAO1	7915702	T	C	0.184505	0.3294	0.0461	0.1309	0.0646	0.3292
12	107510438	rs759522	0.4447	0.02962	0.5453	0.01101	0.5917	7.43e-05	0.3377	0.04823	intronic	BTBD11	107904215	A	G	0.383187	0.3363	0.4553	0.5068	0.3002	0.2986
7	96055047	rs4305826	0.4981	0.03045	0.3955	0.1124	0.6801	4.4e-05	0.4625	0.01514	intronic	DYNC1I1	95684359	G	A	0.245807	0.3085	0.2133	0.3502	0.2366	0.0726
4	12019515	rs7660418	0.4233	0.03082	0.4802	0.0187	0.3454	0.01501	0.6234	0.0001525	intergenic	IR12113;LINC022	12021139	C	T	0.288339	0.5228	0.3545	0.0552	0.3131	0.2894
20	7927572	rs3215458	0.7199	0.03092	0.5936	0.09561	0.953	5.47e-05	0.7621	0.004703	intronic	HAO1	7908219	AT	A	0.182708	0.3284	0.0476	0.1248	0.0646	0.3282
6	43110328	rs17516786	0.5263	0.03095	0.6327	0.0117	0.4434	0.008818	0.7182	0.0001878	intronic	PTK7	43078066	T	C	0.256989	0.3413	0.2061	0.3177	0.1153	0.2699
5	52983403	rs33443	0.4284	0.03099	0.4552	0.0268	0.6084	6.1e-05	0.6453	0.0001368	intergenic	ITGA1;ITGA2	52279233	T	A	0.284944	0.2748	0.2378	0.2542	0.2873	0.3681
7	62442884	rs1554387268	-0.6629	0.03105	-0.5846	0.06896	-0.8833	7.19e-05	-0.8516	0.001103	intergenic	NONE;ZNF733P	NA	NA	NA	NA	NA	NA	NA	NA	NA
8	27056758	rs7818141	0.4189	0.03156	0.3555	0.08154	0.5808	4.27e-05	0.5275	0.001133	intergenic	MIR548H4;STMN	26914275	G	T	0.558706	0.4385	0.549	0.559	0.6928	0.5511
20	7942272	rs16994212	0.7145	0.0316	0.5843	0.09977	0.9302	7.13e-05	0.7537	0.00499	intergenic	HAO1;TMX4	7922919	A	G	0.195288	0.3333	0.0476	0.152	0.0646	0.3507
18	14255165	rs200651710	-0.6004	0.03184	-0.695	0.01716	-0.8394	3.82e-05	-0.7123	0.002369	intergenic	KRD20A5P;CYP4F	NA	NA	NA	NA	NA	NA	NA	NA	NA
1	4118930	rs115990293	-1.737	0.03189	-1.573	0.05422	-1.919	8.22e-05	-1.823	0.002315	intergenic	NC01346;LINC017	4178990	G	A	0.0465256	0.1409	0.0029	0.0575	0.004	0.0092
3	83868452	rs11914825	0.5933	0.03189	0.57	0.0519	0.8526	2.45e-05	0.8446	0.0001901	intergenic	NC02008;LINC009	83917603	T	G	0.217452	0.1339	0.2017	0.267	0.1471	0.32
15	96183151	rs62008617	-0.4501	0.03242	-0.4729	0.03542	-0.6455	5.86e-05	-0.5773	0.001449	ncRNA_intronic	NR2F2-AS1	96726380	G	A	0.160942	0.1161	0.1816	0.0703	0.3012	0.1708
23	119777724	rs11797000	0.4009	0.03245	0.6296	0.002139	0.08451	0.5457	0.1652	0.3012	intergenic	SOWAHD;RPL39	NA	NA	NA	NA	NA	NA	NA	NA	NA
2	188250664	rs12619361	0.3985	0.03258	0.4111	0.03554	0.2646	0.04956	0.5732	0.0003407	ncRNA_intronic	LINC01090	189115391	C	G	0.488419	0.2877	0.3098	0.6513	0.4841	0.6063
14	92199597	rs1312650	-0.5275	0.03291	-0.4952	0.05511	-0.7655	5.07e-05	-0.6408	0.002663	intergenic	CPSF2;SLC24A4	92665941	T	C	0.852636	0.9812	0.8112	0.9576	0.667	0.7986
17	22155848	rs1293649278	0.9583	0.03301	1.031	0.02474	0.7028	0.05137	1.24	0.0008305	intergenic	KCNJ18;UBBP4	NA	NA	NA	NA	NA	NA	NA	NA	NA
4	12040444	rs1899501	0.4192	0.03318	0.4706	0.02165	0.3727	0.009022	0.6138	0.0001901	intergenic	IR12113;LINC022	12042068	G	A	0.290535	0.5188	0.3487	0.0673	0.3191	0.2863
4	12028009	rs16886569	0.4185	0.03335	0.4922	0.01682	0.3822	0.007614	0.6366	0.0001166	intergenic	IR12113;LINC022	12029633	T	C	0.290136	0.5179	0.3501	0.0651	0.3181	0.2883
20	7943203	rs35876964	0.744	0.03412	0.7299	0.04556	1.02	4e-05	0.856	0.00225	intergenic	HAO1;TMX4	7923850	C	CA	0.154752	0.3294	0.0375	0.0076	0.0636	0.3507
3	2714684	rs9852788	0.5262	0.03431	0.6597	0.009491	0.7467	6.63e-05	0.7602	0.0002818	intronic	CNTN4	2756368	T	G	0.18131	0.1726	0.1427	0.2118	0.1461	0.2127
6	146495368	rs846413	0.4595	0.03441	0.5331	0.01825	0.2598	0.08896	0.5928	0.0009103	intergenic	GRM1;RAB32	146816504	G	A	0.333866	0.5675	0.2089	0.2194	0.2087	0.4652
12	121162450	rs208294	0.4267	0.03448	0.5099	0.01656	0.5019	0.0005586	0.6787	9.83e-05	exonic	P2RX7	121600253	T	C	0.529952	0.4137	0.3905	0.6944	0.5557	0.5
7	96055652	rs11374590	0.4847	0.03455	0.3801	0.1255	0.6891	3.65e-05	0.4706	0.01353	intronic	DYNC1I1	95684964	T	TA	0.245208	0.3105	0.2176	0.3434	0.2356	0.0746
4	12026408	rs16886566	0.413	0.03484	0.5067	0.01368	0.3613	0.01106	0.6141	0.0001769	intergenic	IR12113;LINC022	12028032	C	T	0.284744	0.5159	0.3458	0.056	0.3072	0.2894
15	62304492	rs114594	0.415	0.03499	0.4417	0.0317	0.3659	0.01228	0.5895	0.0004573	downstream	MIR8067	62596691	A	T	0.644768	0.8869	0.3847	0.7557	0.4324	0.6483
3	178062077	rs9290635	1.432	0.03539	1.535	0.02914	2.031	9.07e-05	1.866	0.002375	intergenic	NC02015;LINC010	177779865	C	A	0.123403	0.003	0.0634	0.3994	0.0089	0.0348
7	96064625	rs79656757	0.4772	0.03559	0.3968	0.1077	0.6609	6.32e-05	0.4537	0.01619	intronic	DYNC1I1	95693937	AC	A	0.229034	0.3075	0.2046	0.2905	0.2376	0.0736
14	92197800	rs1241707	-0.5006	0.03596	-0.4424	0.07503	-0.7657	3.84e-05	-0.6396	0.00221	intergenic	CPSF2;SLC24A4	92664144	A	C	0.851637	0.9821	0.8112	0.9531	0.667	0.7986
1	229104059	rs35371110	-0.5038	0.03618	-0.4699	0.06026	-0.386	0.02014	-0.7724	0.0002543	intergenic	RHOU;MIR4454	229239806	G	A	0.213858	0.2817	0.1772	0.1657	0.2992	0.1472
7	96055584	rs6953841	0.4799	0.03622	0.3757	0.1295	0.6759	5.03e-05	0.4535	0.01702	intronic	DYNC1I1	95684896	T	G	0.246006	0.3095	0.2133	0.3502	0.2366	0.0726
8	27065541	rs4623440	0.4102	0.03628	0.3517	0.08664	0.5503	9.97e-05	0.5048	0.001886	intergenic	MIR548H4;STMN	26923058	T	C	0.555511	0.4385	0.5461	0.5582	0.6809	0.5501
23	119760392	rs2782227	0.4	0.03672	0.6438	0.002311	0.06964	0.6207	0.1687	0.2938	downstream	SOWAHD	NA	NA	NA	NA	NA	NA	NA	NA	NA
7	96054233	rs28600224	0.4791	0.03674	0.3743	0.1315	0.6723	5.24e-05	0.4514	0.01753	intronic	DYNC1I1	95683545	G	A	0.246006	0.3095	0.2133	0.3502	0.2366	0.0726
7	96055106	rs4305827	0.4791	0.03674	0.3743	0.1315	0.6723	5.24e-05	0.4514	0.01753	intronic	DYNC1I1	95684418	G	A	0.245607	0.3075	0.2133	0.3502	0.2366	0.0726
7	96056036	rs10712465	0.4791	0.03674	0.3743	0.1315	0.6723	5.24e-05	0.4514	0.01753	intronic	DYNC1I1	95685348	CT	C	0.246006	0.3095	0.2133	0.3502	0.2366	0.0726
10	19053258	rs34949183	0.6209	0.03808	0.7091	0.02114	0.8729	6.77e-05	0.874	0.0004159	intronic	MALRD1	19342187	T	A	0.100639	0.1905	0.0432	0.0166	0.0984	0.1646
10	123505674	rs7100435	0.4774	0.03808	0.594	0.01227	0.6689	5.89e-05	0.6124	0.001027	intergenic	BUB3;GPR26	125265190	A	G	0.774161	0.8859	0.6614	0.7663	0.7147	0.8108
10	70993327	rs61851216	0.7668	0.03813	0.8166	0.02999	0.7212	0.008324	1.103	0.00019	intergenic	LINC02622;UNC5	72753084	C	T	0.0946486	0.2073	0.0274	0.1172	0.0258	0.0665
10	70993337	rs61851217	0.7668	0.03813	0.8166	0.02999	0.7212	0.008324	1.103	0.00019	intergenic	LINC02622;UNC5	72753094	T	C	0.0946486	0.2073	0.0274	0.1172	0.0258	0.0665
7	96054558	rs72494422	0.4674	0.03834	0.3743	0.1315	0.6729	4.21e-05	0.4368	0.02111	intronic	DYNC1I1	95683870	C	T	0.251198	0.3095	0.2147	0.3691	0.2366	0.0726
5	26770063	rs77690564	0.7344	0.03881	0.6131	0.1011	1.052	9.97e-05	0.9734	0.001083	intergenic	LINC02211;CDH9	26770172	G	T	0.117812	0	0.098	0.289	0.0915	0.0491
18	10479438	rs525355	-0.401	0.03895	-0.4088	0.0454	-0.5552	8.49e-05	-0.5442	0.000963	intronic	APCDD1	10479435	A	G	0.523562	0.5804	0.4798	0.649	0.4463	0.4059
23	12743222	rs56216376	-0.412	0.03906	-0.5135	0.01863	-0.5731	9.55e-05	-0.4898	0.00342	intergenic	FRMPD4;PRPS2	NA	NA	NA	NA	NA	NA	NA	NA	NA
4	70548419	rs28406187	0.4092	0.03907	0.3922	0.06086	0.5572	9.11e-05	0.5391	0.0008884	intergenic	AMTN;AMBN	71414136	G	A	0.609824	0.5923	0.6787	0.5144	0.662	0.6544
19	1037757	rs77830704	-0.6382	0.03938	-0.6297	0.05182	-0.8598	8.29e-05	-0.8111	0.001647	exonic	CNN2	NA	NA	NA	NA	NA	NA	NA	NA	NA
10	19025926	rs12781949	0.6268	0.0395	0.6608	0.03672	0.8963	4.11e-05	0.8801	0.0004187	intergenic	ARL5B;MALRD1	19314855	A	C	0.120807	0.1885	0.0461	0.0915	0.0944	0.1708
20	7963232	rs16994242	0.6897	0.03953	0.6229	0.07748	0.9542	7.97e-05	0.7424	0.007307	intergenic	HAO1;TMX4	7943879	C	T	0.176518	0.3323	0.0432	0.0802	0.0646	0.3558
14	92197144	rs1241706	-0.4858	0.04035	-0.446	0.07028	-0.7488	5.2e-05	-0.6282	0.002474	intergenic	CPSF2;SLC24A4	92663488	C	T	0.859225	0.9821	0.8141	0.9803	0.667	0.7986
5	52954054	rs6450100	0.4077	0.04038	0.4364	0.03401	0.5914	8.8e-05	0.6215	0.0002402	UTR3	ITGA1	52249884	C	T	0.707668	0.7252	0.7608	0.7209	0.7147	0.6268
7	100953138	rs1160111527	0.6152	0.04148	0.4341	0.156	0.8719	7.74e-05	0.8973	0.0005824	exonic	MUC3A	NA	NA	NA	NA	NA	NA	NA	NA	NA
7	96066510	rs7805771	0.4539	0.04316	0.3746	0.1245	0.6385	9.9e-05	0.431	0.02112	intronic	DYNC1I1	95695822	A	T	0.230431	0.3145	0.2046	0.2905	0.2376	0.0736
14	92191304	rs1241697	-0.4809	0.04423	-0.4424	0.07503	-0.7582	4.59e-05	-0.6288	0.002635	intergenic	CPSF2;SLC24A4	92657648	T	C	0.852037	0.9821	0.8127	0.9531	0.668	0.7986
13	66322380	rs17501046	0.464	0.04431	0.5535	0.01987	0.3746	0.03189	0.6907	0.0004154	intronic	PCDH9	66896512	G	A	0.103235	0.1329	0.0865	0.0144	0.1551	0.1513
15	62304647	rs10670473	0.3959	0.04438	0.4454	0.02977	0.3617	0.01323	0.5813	0.0005412	downstream	MIR8067	62596846	C	CTT	0.644169	0.8849	0.3847	0.7557	0.4324	0.6472
10	70993385	rs76619375	0.7373	0.04458	0.7864	0.03535	0.6941	0.01031	1.079	0.0002335	intergenic	LINC02622;UNC5	72753142	C	T	0.0946486	0.2073	0.0274	0.1172	0.0258	0.0665
10	70993386	rs79926639	0.7373	0.04458	0.7864	0.03535	0.6941	0.01031	1.079	0.0002335	intergenic	LINC02622;UNC5	72753143	C	G	0.0946486	0.2073	0.0274	0.1172	0.0258	0.0665
20	7931551	rs2235248	0.679	0.0449	0.6099	0.08778	0.9726	4.64e-05	0.7578	0.005376	intronic	HAO1	7912198	T	A	0.180711	0.3294	0.0447	0.1172	0.0646	0.3292
20	7941844	rs12624982	0.679	0.0449	0.6099	0.08778	0.9465	6.94e-05	0.7578	0.005376	intergenic	HAO1;TMX4	7922491	T	C	0.185104	0.3313	0.0447	0.1172	0.0646	0.3497
7	96055562	rs10229316	0.4616	0.04492	0.3489	0.1617	0.6607	7.5e-05	0.4437	0.01993	intronic	DYNC1I1	95684874	G	A	0.223043	0.3095	0.2046	0.2678	0.2366	0.0726
20	7930886	rs34473022	0.6754	0.04601	0.6066	0.08942	0.9699	4.88e-05	0.7551	0.005542	intronic	HAO1	7911533	A	G	0.180312	0.3294	0.0447	0.1165	0.0646	0.3282
1	92983000	rs3943283	0.4967	0.04629	0.5687	0.02627	0.7023	0.0001278	0.8475	4.25e-05	intergenic	DIPK1A;MTF2	93448557	T	A	0.213059	0.2371	0.196	0.2216	0.165	0.2382
10	70993689	rs12242399	0.7305	0.04666	0.779	0.03716	0.6894	0.01083	1.072	0.000254	intergenic	LINC02622;UNC5	72753446	G	A	0.0952476	0.2073	0.0303	0.1172	0.0258	0.0675

7	96055810	rs2106513	0.4536	0.04681	0.3743	0.1315	0.6621	6.49e-05	0.4368	0.02111	intronic	DYNC1I1	95685122	C	T	0.246006	0.3095	0.2133	0.3502	0.2366	0.0726
14	92190444	rs1241695	-0.4741	0.04728	-0.4353	0.07979	-0.7445	6.27e-05	-0.6214	0.002957	intergenic	CPSF2;SLC24A4	92656788	T	C	0.859425	0.9821	0.8156	0.9796	0.668	0.7986
10	70992715	rs61851214	0.7407	0.04777	0.7832	0.04009	0.6673	0.01475	1.091	0.0002345	intergenic	.INC02622;UNC5	72752472	A	C	0.0808706	0.2054	0.0231	0.0696	0.0249	0.0665
3	83871521	rs72912683	0.5526	0.04786	0.5687	0.05275	0.8432	3.05e-05	0.8356	0.0002422	intergenic	NC02008;LINC009	83920672	T	C	0.21905	0.1339	0.2017	0.2738	0.1471	0.319
10	70993547	rs74144382	0.7242	0.04807	0.7864	0.03535	0.6861	0.01115	1.068	0.0002644	intergenic	.INC02622;UNC5	72753304	A	G	0.0948482	0.2073	0.0288	0.1172	0.0258	0.0665
10	70993282	rs61851215	0.7252	0.04821	0.7749	0.03812	0.685	0.01132	1.068	0.0002677	intergenic	.INC02622;UNC5	72753039	T	C	0.0948482	0.2073	0.0288	0.1172	0.0258	0.0665
10	59165214	rs149096164	1.213	0.04841	1.146	0.06995	1.015	0.02029	1.714	0.0004834	intergenic	LINC00844;CCEP	60924974	TA	T	0.0696885	0.0327	0.0447	0.1657	0.0129	0.0542
8	117356255	rs1695733	0.6407	0.04897	0.5191	0.1449	0.9747	9.85e-05	0.783	0.005558	intergenic	SLC30A8;MED30	118368494	T	C	0.94389	0.9246	0.9395	0.9902	0.9006	0.9489
10	70990917	rs12569958	0.729	0.05119	0.7707	0.04325	0.5951	0.02816	1.069	0.0003015	intergenic	.INC02622;UNC5	72750674	T	C	0.0786741	0.2024	0.0187	0.0666	0.0249	0.0654
6	128963758	rs7770018	-0.6046	0.05212	-0.7088	0.03635	-0.9367	8.93e-05	-0.6589	0.01249	intronic	LAMA2	129284903	A	G	0.191893	0.002	0.121	0.4463	0.1372	0.1503
10	70993734	rs12242280	0.7114	0.05232	0.7721	0.03889	0.6783	0.01204	1.057	0.0003015	intergenic	.INC02622;UNC5	72753491	C	T	0.0952476	0.2073	0.0303	0.1172	0.0258	0.0675
3	83871040	rs62259177	0.5399	0.05265	0.5545	0.05834	0.8248	4.27e-05	0.8174	0.0003113	intergenic	NC02008;LINC009	83920191	A	G	0.21865	0.1339	0.2017	0.2716	0.1471	0.32
3	83871069	rs111962895	0.5399	0.05265	0.5545	0.05834	0.8248	4.27e-05	0.8174	0.0003113	intergenic	NC02008;LINC009	83920220	AC	A	0.21865	0.1339	0.2017	0.2716	0.1471	0.32
3	83871099	rs72912679	0.5399	0.05265	0.5545	0.05834	0.8248	4.27e-05	0.8174	0.0003113	intergenic	NC02008;LINC009	83920250	T	C	0.219249	0.1339	0.2017	0.2738	0.1471	0.32
3	83871296	rs72912681	0.5399	0.05265	0.5545	0.05834	0.8248	4.27e-05	0.8174	0.0003113	intergenic	NC02008;LINC009	83920447	G	A	0.21845	0.1339	0.2017	0.2716	0.1471	0.319
10	122255947	rs4751880	0.3893	0.05271	0.4566	0.02986	0.7041	2.84e-06	0.6778	7.73e-05	intergenic	TACC2;BTBD16	124015462	A	C	0.482428	0.5962	0.5288	0.5159	0.34	0.4335
16	17575509	rs28375623	-0.4272	0.05274	-0.5477	0.02252	-0.6883	3.69e-05	-0.6297	0.001171	intergenic	XYLT1;NPIPA8	17669366	T	C	0.23742	0.1974	0.2089	0.2579	0.2266	0.2822
3	83871688	rs62259178	0.5263	0.05388	0.5367	0.0603	0.8073	4.26e-05	0.7955	0.000295	intergenic	NC02008;LINC009	83920839	C	T	0.193291	0.1379	0.1888	0.1808	0.1461	0.319
20	7934955	rs34334468	0.6484	0.05416	0.5766	0.1049	0.9218	9.08e-05	0.7249	0.007206	intronic	HAO1	7915602	T	A	0.189097	0.3294	0.0461	0.1483	0.0646	0.3292
20	7923932	rs3817774	0.6475	0.05433	0.5987	0.09293	0.9419	6.03e-05	0.7514	0.004852	intronic	HAO1	7904579	G	A	0.182907	0.3294	0.0476	0.1248	0.0646	0.3282
10	70993442	rs73270607	0.6506	0.0563	0.7111	0.04058	0.6915	0.004595	0.9665	0.0002834	intergenic	.INC02622;UNC5	72753199	T	C	0.156749	0.2073	0.0533	0.3359	0.0298	0.0665
3	83872560	rs11919252	0.5231	0.05795	0.5938	0.03844	0.7938	7.89e-05	0.7684	0.0005099	intergenic	NC02008;LINC009	83921711	T	G	0.184105	0.1359	0.1801	0.1831	0.1372	0.2863
20	7923157	rs2294299	0.6378	0.05811	0.5645	0.1123	0.9161	9.48e-05	0.7317	0.007091	intronic	HAO1	7903804	C	G	0.195487	0.3294	0.0548	0.1679	0.0656	0.3282
4	152213807	rs11727661	0.5671	0.05874	0.4236	0.1972	0.5375	0.01515	0.9414	0.0001614	intergenic	.INC02273;FBXW	153134959	C	A	0.133187	0.12	0.0692	0.2443	0.1044	0.0716
4	152214267	rs11727800	0.5671	0.05874	0.4236	0.1972	0.5258	0.01746	0.9173	0.0002288	intergenic	.INC02273;FBXW	153135419	C	T	0.133387	0.12	0.0692	0.2451	0.1044	0.0716
3	83870660	rs11712109	0.5221	0.05988	0.5382	0.06529	0.8022	6.16e-05	0.7945	0.0004257	intergenic	NC02008;LINC009	83919811	A	G	0.221046	0.1339	0.2017	0.2806	0.1471	0.32
10	70992407	rs12254773	0.6831	0.06026	0.735	0.04735	0.6479	0.01522	1.027	0.0003765	intergenic	.INC02622;UNC5	72752164	A	G	0.0936502	0.2054	0.0274	0.115	0.0258	0.0665
3	90570192	rs1316383398	0.5643	0.06063	0.6538	0.03669	0.8748	6.39e-05	0.8072	0.001309	intergenic	EPHA3;NONE	NA	NA	NA	NA	NA	NA	NA	NA	NA
14	92189096	rs1241693	-0.4456	0.06134	-0.4357	0.07892	-0.7416	5.17e-05	-0.5874	0.004513	intergenic	CPSF2;SLC24A4	92655440	T	C	0.786342	0.9821	0.7997	0.7171	0.668	0.7904
10	70993084	rs74144375	0.673	0.06367	0.7383	0.04638	0.6427	0.01596	1.02	0.0004098	intergenic	.INC02622;UNC5	72752841	A	G	0.0946486	0.2063	0.0288	0.1172	0.0258	0.0665
10	18978492	rs34562958	0.5614	0.06777	0.6405	0.04243	0.8531	8.55e-05	0.8443	0.0007021	intergenic	ARL5B;MALRD1	19267421	A	G	0.125799	0.1825	0.0519	0.112	0.0954	0.1697
10	70993673	rs12256932	0.6592	0.06918	0.7057	0.05621	0.6462	0.01539	1.018	0.0004141	intergenic	.INC02622;UNC5	72753430	A	G	0.0952476	0.2073	0.0303	0.1172	0.0258	0.0675
1	92983222	rs2127400	0.4517	0.07048	0.5183	0.04326	0.6876	0.0001737	0.8216	6.94e-05	intergenic	DIPK1A;MTF2	93448779	C	T	0.213059	0.2371	0.196	0.2216	0.165	0.2382
3	83871402	rs72912682	0.503	0.07314	0.5118	0.08352	0.8139	5.45e-05	0.7998	0.0004318	intergenic	NC02008;LINC009	83920553	C	T	0.21845	0.1339	0.2017	0.2716	0.1471	0.319
3	83870479	rs11719954	0.5092	0.07316	0.5302	0.07686	0.7937	7.84e-05	0.791	0.0005113	intergenic	NC02008;LINC009	83919630	C	T	0.21865	0.1339	0.2017	0.2716	0.1471	0.32
4	71123704	rs16845804	0.4331	0.08003	0.4876	0.0587	0.4673	0.009876	0.7294	0.0003482	intergenic	DCK;SLC4A4	71989421	T	C	0.209065	0.252	0.0965	0.2451	0.1322	0.2751
4	71051162	rs12511905	0.3285	0.08388	0.3693	0.06087	0.09598	0.4848	0.4713	0.003156	intergenic	DCK;SLC4A4	71916879	A	G	0.433906	0.622	0.5519	0.0726	0.5497	0.5256
3	83872604	rs34577640	0.4645	0.08527	0.5536	0.04849	0.7644	7.64e-05	0.7027	0.001197	intergenic	NC02008;LINC009	83921755	C	CA	0.187101	0.1349	0.2032	0.171	0.1501	0.2894
3	83872053	rs79294805	0.4601	0.08945	0.473	0.09609	0.7656	8.94e-05	0.7417	0.0007046	intergenic	NC02008;LINC009	83921204	G	A	0.196685	0.1359	0.1873	0.1959	0.1471	0.318
11	19254244	rs793239	-0.4902	0.1069	-0.4752	0.1347	-0.9289	7.96e-05	-0.9446	0.0008494	intergenic	E2F8;NAV2	19275791	C	T	0.13139	0.1071	0.0648	0.2451	0.1103	0.0716
6	13237875	rs602976	0.4773	0.1081	0.4317	0.1718	0.861	5.18e-05	0.7991	0.0009356	intronic	PHACTR1	13238107	T	C	0.155551	0.1319	0.2622	0.2292	0.0596	0.1033
6	36584002	rs148512123	-0.2596	0.2353	-0.4191	0.07733	-0.6543	7.85e-05	-0.6926	0.0003702	intergenic	STK38;SRSF3	36551779	CCACCGCGCCCG	G	0.246406	0.3899	0.196	0.1687	0.2753	0.2096
10	70995727	rs12246540	0.5897	0.2843	0.7564	0.1729	0.8442	0.01847	1.38	0.0005192	intergenic	.INC02622;UNC5	72755484	C	T	0.0628994	0.2073	0.0043	0.0424	0.004	0.044
10	70992670	rs74384138	0.5872	0.3099	0.7531	0.1976	0.8164	0.02689	1.432	0.0005121	intergenic	.INC02622;UNC5	72752427	G	A	0.0513179	0.2054	0	0.003	0.003	0.044
10	70994745	rs4451634	0.5869	0.3102	0.7528	0.1978	0.8164	0.02686	1.433	0.0005078	intergenic	.INC02622;UNC5	72754502	G	A	0.0511182	0.2073	0	0.0008	0.003	0.044
21	27401704	rs2830913	-0.2	0.3233	-0.1932	0.3621	-0.5952	7.58e-05	-0.4918	0.004184	intergenic	DAMTS5;LINC001	28774023	G	A	0.270367	0.131	0.3718	0.0688	0.4304	0.4499
7	122153918	rs279697	0.2319	0.5998	0.2183	0.6339	1.22	8.54e-06	1.11	0.0003616	intergenic	AASS;FEZF1	121793972	T	A	0.124601	0.0972	0.0648	0.2315	0.0805	0.0961
7	122152776	rs279691	0.227	0.6076	0.2136	0.6413	1.216	9.13e-06	1.105	0.000381	intergenic	AASS;FEZF1	121792830	A	T	0.124601	0.0972	0.0648	0.2315	0.0805	0.0961
7	122156981	rs157656	0.2136	0.6285	0.2183	0.6339	1.211	9.61e-06	1.099	0.0004035	intergenic	AASS;FEZF1	121797035	C	A	0.1252	0.0972	0.0648	0.2337	0.0805	0.0961
5	6323777	rs76040944	NA	NA	NA	NA	NA	NA	NA	NA	ncRNA_intronic	LINC02145	6323890	G	C	0.00579073	0.0278	0	0	0	0.001
6	56087877	rs147515421	NA	NA	NA	NA	NA	NA	NA	NA	intronic	COL21A1	55952675	T	A	0.00399361	0.0198	0	0	0	0
6	56215894	rs146151949	NA	NA	NA	NA	NA	NA	NA	NA	intronic	COL21A1	56080692	T	C	0.00439297	0.0218	0	0	0	0
20	29354153	rs1290862766	NA	NA	NA	NA	-1.559	5.12e-05	-2.181	0.0003661	intergenic	FRG1DP;FRG2EP	NA	NA	NA	NA	NA	NA	NA	NA	NA

Supplementary Table S2. Genomic risk loci of interest from large (medium/giant) aneurysm WGS Association

Genomic Loci	uniqID	rs numbers	P-value	chromosome	Position (hg 19)	nSNPs	nGWASSNPs	nIndSigSNPs	IndSigSNPs	nLeadSNPs	LeadSNPs
1	1:30833646:A:G	rs634288	4.377E-06	1	30833646	8	3	1	rs634288	1	rs634288
2	1:63674914:G:T	rs1328589	7.327E-06	1	63674914	1	1	1	rs1328589	1	rs1328589
3	4:155069547:A:G	rs62330192	2.586E-07	4	1.55E+08	13	1	1	rs62330192	1	rs62330192
4	6:53591144:A:G	rs114644621	8.213E-06	6	53591144	2	2	1	rs114644621	1	rs114644621
5	7:81262900:C:T	rs10276547	2.171E-06	7	81262900	18	10	1	rs10276547	1	rs10276547
6	7:123210617:A:G	rs34163760	4.844E-06	7	1.23E+08	21	17	1	rs34163760	1	rs34163760
7	8:40574679:G:T	rs9643846	6.568E-07	8	40574679	30	29	1	rs9643846	1	rs9643846
8	9:10285669:A:G	rs193014517	3.433E-06	9	10285669	15	14				
8	9:10297519:A:G	rs600075	2.358E-06	9	10297519	19	12	2	rs600075;rs193014517	1	rs600075
9	11:45740416:C:CCACTCCT	rs63237761	7.237E-06	11	45740416	4	3	1	rs63237761	1	rs63237761
10	13:22105506:A:T	rs12585631	4.109E-06	13	22105506	99	87	1	rs12585631	1	rs12585631
11	14:50305580:A:G	rs117885021	4.925E-06	14	50305580	3	2	1	rs117885021	1	rs117885021
12	22:48738133:G:T	rs35056946	2.922E-06	22	48738133	5	1	1	rs35056946	1	rs35056946

Genomic Loci : Index of assigned genomic locus matched with "GenomicRiskLoci.txt". Multiple independent lead SNPs can be assigned to the same genomic locus.
uniqID : Unique ID of SNPs consists of chr:position:allele1:allele2 where alleles are alphabetically ordered.
rs numbers : rsID of the SNP.
chromosome : chromosome number
Position (hg 19) : genomic position on hg19
P-value : Association analysis p value
nSNPs : Number of SNPs which are in LD of the independent significant SNP given r2<0.6
nGWASSNPs : Number of WGS-tagged SNPs which are in LD of the ind. sig. SNP given r2<0.6
nIndSigSNPs : Number of independent significant SNPs which are in LD of the lead SNP at r2 0.1
IndSigSNPs : rsID of independent significant SNPs which are in LD of the lead SNP at r2 0.1
nLeadSNPs : The number of lead SNPs in the genomic locus
LeadSNPs : rsID of lead SNPs in the genomic locus

Supplementary Table S3. All genes mapped in SNP-based (FUMA) for large (medium/giant) aneurysm

ensg	symbol	chr	start	end	strand	type	entrezID	HUGO	pLI	ncRVIS	posMapSNPs	posMapMaxCADD	eqtlMapSNPs	eqtlMapminP	eqtlMapminQ	eqtlDirection	ciMap	ciMapts	minGwasP	IndSigSNPs	GenomicLocus
ENSG00000180776	ZDHHC20	13	21950263	22033509	-1	protein_coding	253832	ZDHHC20	0.00233007	NA	2	5.55	3	1.02E-58	0	+	No	NA	4.11E-06	rs12585631	10
ENSG00000165487	MICU2	13	22066836	22178353	-1	protein_coding	221154	MICU2	2.60E-13	0.606595223	93	15.2	3	1.79E-46	0	-	No	NA	4.11E-06	rs12585631	10
ENSG00000128609	NDUFA5	7	1.23E+08	1.23E+08	-1	protein_coding	4698	NDUFA5	0.17396305	-0.39682751	7	14.4	1	4.63E-13	6.09E-26	+	No	NA	5.98E-05	rs34163760	6
ENSG00000106299	WASL	7	1.23E+08	1.23E+08	-1	protein_coding	8976	WASL	0.96196049	-0.22723743	0	0	1	6.50E-08	0.00027767	+	No	NA	0.000195	rs34163760	6
ENSG00000224429	LINC00422	13	21877654	21922860	-1	lincRNA	1.01E+08	NA	NA	NA	0	0	1	1.25E-07	2.15E-06	+	No	NA	4.11E-06	rs12585631	10
ENSG00000272686	RP11-390E23.6	7	1.23E+08	1.23E+08	1	antisense	NA	NA	NA	NA	0	0	1	2.77E-06	2.41E-05	+	No	NA	0.000195	rs34163760	6
ENSG00000235076	GAPDHP52	13	21932512	21933453	1	pseudogene	NA	GAPDHP52	NA	NA	0	0	3	2.91E-06	7.35E-07	+	No	NA	4.11E-06	rs12585631	10
ENSG00000173914	RBM4B	11	66432469	66445392	-1	protein_coding	83759	RBM4B	0.4422467	-1.11377278	0	0	1	4.48E-06	0.03006487	-	No	NA	2.86E-05	rs12585631	10
ENSG00000236953	ZDHHC20-IT1	13	21951116	21952013	-1	sense_intronic	NA	ZDHHC20-IT1	NA	NA	0	0	3	2.47E-05	0.00882319	+	No	NA	4.11E-06	rs12585631	10
ENSG00000165061	ZMAT4	8	40388109	40755352	-1	protein_coding	79698	ZMAT4	0.01932686	-0.44555495	30	15	1	3.27170000000001e-310	0	-	No	NA	6.57E-07	rs9643846	7
ENSG00000224209	LINC00466	1	63655660	63782902	-1	lincRNA	199899	LINC00466	NA	NA	1	3.89	0	NA	NA	NA	No	NA	7.33E-06	rs1328589	2
ENSG00000233491	AC010091.1	7	81205702	81255532	-1	lincRNA	1E+08	NA	NA	NA	8	13.5	0	NA	NA	NA	No	NA	2.17E-06	rs10276547	5
ENSG00000164675	IQUB	7	1.23E+08	1.23E+08	-1	protein_coding	154865	IQUB	3.24E-13	-0.24811521	9	14.4	0	NA	NA	NA	No	NA	0.000108	rs34163760	6
ENSG00000232524	RP11-332K15.1	7	1.23E+08	1.23E+08	1	antisense	NA	NA	NA	NA	1	2.47	0	NA	NA	NA	No	NA	0.000199	rs34163760	6
ENSG00000207338	RNU6-296P	7	1.23E+08	1.23E+08	-1	snRNA	NA	RNU6-296P	NA	NA	1	2.47	0	NA	NA	NA	No	NA	0.000199	rs34163760	6
ENSG00000146809	ASB15	7	1.23E+08	1.23E+08	1	protein_coding	142685	ASB15	6.01E-08	-0.75131263	10	5.34	0	NA	NA	NA	No	NA	4.84E-06	rs34163760	6
ENSG00000230442	RP11-390E23.3	7	1.23E+08	1.23E+08	-1	antisense	NA	NA	NA	NA	5	3.64	0	NA	NA	NA	No	NA	0.00013	rs34163760	6
ENSG00000153707	PTPRD	9	8314246	10612723	-1	protein_coding	5789	PTPRD	0.99999998	-0.57383378	34	14.8	0	NA	NA	NA	No	NA	2.36E-06	rs193014517;rs600075	8
ENSG00000254519	CTD-2210P24.1	11	45743858	45765801	1	lincRNA	1.01E+08	NA	NA	NA	3	4.86	0	NA	NA	NA	No	NA	7.24E-06	rs63237761	9
ENSG00000226908	HIST1H2BPS3	13	22057973	22058313	-1	pseudogene	NA	HIST1H2BPS3	NA	NA	6	9.39	0	NA	NA	NA	No	NA	1.03E-05	rs12585631	10
ENSG00000238878	snoU13	13	22101939	22102039	-1	snoRNA	NA	NA	NA	NA	17	12.9	0	NA	NA	NA	No	NA	4.11E-06	rs12585631	10
ENSG00000230022	FNTAP2	13	22104829	22105462	1	pseudogene	NA	FNTAP2	NA	NA	17	12.9	0	NA	NA	NA	No	NA	4.11E-06	rs12585631	10
ENSG00000207518	RNU6-59P	13	22125443	22125549	-1	snRNA	1.01E+08	RNU6-59P	NA	NA	29	15.2	0	NA	NA	NA	No	NA	1.00E-05	rs12585631	10
ENSG00000165501	LRR1	14	50065415	50081390	1	protein_coding	122769	LRR1	8.61E-07	0.090498624	1	4.35	0	NA	NA	NA	No	NA	NA	rs117885021	11
ENSG00000165502	RPL36AL	14	50085237	50087403	-1	protein_coding	6166	RPL36AL	0.0418332	-0.08365874	1	4.35	0	NA	NA	NA	No	NA	NA	rs117885021	11
ENSG00000168282	MGAT2	14	50087489	50090198	1	protein_coding	4247	MGAT2	0.9550317	-0.61504694	1	4.35	0	NA	NA	NA	No	NA	NA	rs117885021	11
ENSG00000258377	RP11-649E7.5	14	50087533	50090198	-1	antisense	NA	NA	NA	NA	1	4.35	0	NA	NA	NA	No	NA	NA	rs117885021	11
ENSG00000165516	KLHDC2	14	50234326	50249909	1	protein_coding	23588	KLHDC2	0.15795896	NA	1	0.23	0	NA	NA	NA	No	NA	5.70E-06	rs117885021	11
ENSG00000165525	NEMF	14	50249997	50319921	-1	protein_coding	9147	NEMF	0.98170645	-0.01668801	2	0.94	0	NA	NA	NA	No	NA	4.93E-06	rs117885021	11
ENSG00000197502	AL627171.1	14	50300810	50311552	-1	protein_coding	NA	NA	NA	NA	1	0.94	0	NA	NA	NA	No	NA	4.93E-06	rs117885021	11
ENSG00000252474	RNU6-539P	14	50306443	50306545	1	snRNA	NA	RNU6-539P	NA	NA	1	0.94	0	NA	NA	NA	No	NA	4.93E-06	rs117885021	11
ENSG00000145428	RNF175	4	1.55E+08	1.55E+08	-1	protein_coding	285533	RNF175	6.79E-10	0.383163491	0	0	0	NA	NA	NA	Yes	Mesendoderm	NA	rs62330192	3
ENSG00000145423	SFRP2	4	1.55E+08	1.55E+08	-1	protein_coding	6423	SFRP2	0.05388452	-0.28370247	0	0	0	NA	NA	NA	Yes	Mesendoderm	NA	rs62330192	3
ENSG00000187391	MAGI2	7	77646393	79082890	-1	protein_coding	9863	MAGI2	0.88317439	-1.16024605	0	0	0	NA	NA	NA	Yes	Left_Ventricle	2.93E-06	rs10276547	5
ENSG00000234456	MAGI2-AS3	7	79082198	79100524	1	processed_transcript	1.01E+08	MAGI2-AS3	NA	NA	0	0	0	NA	NA	NA	Yes	Left_Ventricle	2.93E-06	rs10276547	5
ENSG00000127955	GNAI1	7	79763271	79848718	1	protein_coding	2770	GNAI1	0.91752824	0.288400901	0	0	0	NA	NA	NA	Yes	Mesendoderm	2.93E-06	rs10276547	5
ENSG00000019991	HGF	7	81328322	81399754	-1	protein_coding	3082	HGF	0.99470103	-0.59413812	0	0	0	NA	NA	NA	Yes	em_Cell:Mesendod	2.93E-06	rs10276547	5
ENSG00000153956	CACNA2D1	7	81575760	82073114	-1	protein_coding	781	CACNA2D1	0.9999958	-1.09342797	0	0	0	NA	NA	NA	Yes	esenchymal_Stem_C	2.93E-06	rs10276547	5
ENSG00000132950	ZMYM5	13	20397622	20437776	-1	protein_coding	9205	ZMYM5	0.20037813	0.302122761	0	0	0	NA	NA	NA	Yes	Fetal_Cortex	0.000151	rs12585631	10
ENSG00000233325	MIPEPP3	13	21872278	21880512	1	pseudogene	NA	MIPEPP3	NA	NA	0	0	0	NA	NA	NA	Yes	Adult_Cortex	0.000151	rs12585631	10
ENSG00000102678	FGF9	13	22245522	22278637	1	protein_coding	2254	FGF9	0.91071591	-0.65247124	0	0	0	NA	NA	NA	Yes	ult_Cortex:Fetal_Co	0.000151	rs12585631	10
ENSG00000232187	FTH1P7	13	23270162	23270713	-1	pseudogene	NA	FTH1P7	NA	NA	0	0	0	NA	NA	NA	Yes	_Cell:Mesendoderm	0.000151	rs12585631	10
ENSG00000226507	IPMKP1	13	23411165	23412019	-1	pseudogene	NA	IPMKP1	NA	NA	0	0	0	NA	NA	NA	Yes	.90:Mesendoderm:h	0.000151	rs12585631	10
ENSG00000102683	SGCG	13	23755091	23899304	1	protein_coding	6445	SGCG	0.00637038	1.253761194	0	0	0	NA	NA	NA	Yes	ymal_Stem_Cell:Me	0.000151	rs12585631	10
ENSG00000151835	SACS	13	23902965	24007841	-1	protein_coding	26278	SACS	6.15E-12	0.125705792	0	0	0	NA	NA	NA	Yes	Fetal_Cortex:IMR9(	0.000151	rs12585631	10
ENSG00000232977	LINC00327	13	24040710	24061603	1	lincRNA	1.01E+08	LINC00327	NA	NA	0	0	0	NA	NA	NA	Yes	IMR90	0.000151	rs12585631	10
ENSG00000127863	TNFRSF19	13	24144509	24250232	1	protein_coding	55504	TNFRSF19	0.01988251	NA	0	0	0	NA	NA	NA	Yes	x:GM12878:IMR9(	0.000151	rs12585631	10
ENSG00000252695	MIR2276	13	24736555	24736643	1	miRNA	1E+08	MIR2276	NA	NA	0	0	0	NA	NA	NA	Yes	esenchymal_Stem_C	0.000151	rs12585631	10

ensg : ENSG ID
symbol : Gene Symbol
chr : chromosome
start : Starting position of the gene
end : Ending position of the gene
strand : Strand of the gene
type : Gene biotype from Ensembl
entrezID : entrez ID (if available)
HUGO : HUGO (HGNC) gene symbol
pLI : pLI score from ExAC database. The probability of being loss-of-function intolerant. The higher the score is, the more intolerant to loss-of-function mutations the gene is.
ncRVIS : Non-coding residual variation intolerance score. The higher the score is, the more intolerant to non-coding variation the gene is.
posMapSNPs (posMap): Number of SNPs mapped to gene based on positional mapping (after functional filtering if parameters are given).
posMapMaxCADD (posMap): The maximum CADD score of mapped SNPs by positional mapping.
eqtlMapSNPs (eqtlMap): Number of SNPs mapped to the gene based on eQTL mapping.
eqtlMapminP (eqtlMap): The minimum eQTL P-value of mapped SNPs.
eqtlMapminQ (eqtlMap): The minimum eQTL FDR of mapped SNPs.
eqtlMapts (eqtlMap): Tissue types of mapped eQTL SNPs.
eqtlDirection (eqtlMap): Consequential direction of mapped eQTL SNPs after aligning risk increasing alleles in GWAS and tested alleles in eQTL data source.
"NA" if risk increase alleles are not defined either because signed effect is not provided in the input GWAS file or all of eQTL SNPs are not in the input GWAS file but included from reference panel for the gene.
ciMap (ciMap): "Yes" if the gene is mapped by chromatin interaction mapping, "No" otherwise.
ciMapts (ciMap): Tissue/cell types of mapped chromatin interactions.
minGwasP : The minimum P-value of mapped SNPs.
IndSigSNPs : rsID of the independent significant SNPs that are in LD with the mapped SNPs.
Note that this column does not represent SNPs that are mapped to the gene.
The actual mapped SNPs are available in gene_snps.txt file.
GenomicLocus : Index of genomic loci where mapped SNPs are from. Multiple loci can be assigned with ":" delimiter.

Supplementary Table S4. Expression values of the genes prioritized by FUMA for large (medium/giant) aneurysm in 54 tissues

ensg	symbol	Adipose_Subcutaneous	Adipose_Visceral_Omentum	Adrenal_Gland	Artery_Aorta	Artery_Coronary	Artery_Tibial	Bladder	Brain_Amygdala	Brain_Anterior_cingulate_cortex_BA24	Brain_Caudate_basal_ganglia	Brain_Cerebellar_Hemisphere	Brain_Cerebellum	Brain_Cortex
ENSG00000224209	LINC00466	0.02643209	0.01749987	0.00498671	0.00823429	0.05025569	0.01230961	0.03856936	0.02175256	0.02448174	0.01828714	0.00447329	0.00460604	0.01743299
ENSG00000145428	RNF175	0.91620973	1.08578252	0.61956131	0.81778263	0.77598814	0.71605331	0.61709067	2.29324261	3.05717471	2.69157692	3.5367776	3.34824503	3.37024868
ENSG00000145423	SFRP2	4.53076203	5.48655725	2.61221389	5.55939669	5.16983261	5.62257272	5.59929473	1.85161971	2.06346234	1.95697033	0.98214419	1.53974561	3.11201098
ENSG00000187391	MAGI2	1.33665895	1.02452922	1.01965489	1.68761657	1.98005572	2.75767788	1.40406691	2.08571439	2.21681753	2.42298184	2.32961763	2.35955981	2.37550399
ENSG00000234456	MAGI2-AS3	4.25061804	3.99764758	3.71509395	4.34342983	4.41625805	4.35808936	3.95453006	2.58098042	2.46827889	2.494682	4.53672841	4.53324071	2.80027467
ENSG00000127955	GNAI1	5.45280686	5.32610047	4.58853183	3.91786802	4.40504533	5.20777087	4.8939913	4.33419505	4.16263953	3.73770566	5.10968486	4.63960306	4.12333713
ENSG00000233491	AC010091.1	0.00361033	0.00376635	0.00312362	0.00930265	0.04570288	0.03718273	0.00075021	0.00071258	0.00039676	0.00064252	0.00087646	0.00047234	0.0012103
ENSG00000019991	HGF	2.31917374	2.71375789	2.99465204	2.94947345	3.47035323	2.9661212	2.69580874	0.55760199	1.19866163	0.38582957	0.25794062	0.32097662	1.23962184
ENSG00000153956	CACNA2D1	3.75973398	3.12434226	3.48423444	3.86444273	3.73579009	4.20419444	3.5772669	2.84347747	3.46682599	3.02937034	3.97735811	3.66495376	3.41438658
ENSG00000164675	IQUB	0.3258829	0.36278978	0.66566466	0.44957487	0.38941902	0.37012959	0.50237408	0.31783298	0.2369875	0.45043677	0.51945224	0.60761506	0.31633418
ENSG00000232524	RP11-332K15.1	0.02376066	0.03602409	0.04729637	0.05290148	0.03110666	0.03302885	0.42419856	0.02682451	0.0300435	0.03157038	0.01189344	0.03712434	0.03317011
ENSG00000207338	RNU6-296P	0	0.00505688	0.00177342	0	0.00188581	0.00170653	0	0	0.00361413	0.00537586	0.00275422	0	0.00119874
ENSG00000128609	NDUFA5	3.98995006	3.92762328	4.86104534	4.37071128	4.42441885	4.65466424	4.34959488	4.81003196	5.13201069	4.71528532	5.25761865	4.94724168	5.05604843
ENSG00000146809	ASB15	0.03914222	0.02590591	0.01849762	0.03864538	0.15969029	0.063053	0.01984887	0.01390831	0.01252737	0.01291605	0.05236369	0.0503958	0.01858449
ENSG00000230442	RP11-390E23.3	0.0045738	0.00337527	0.00499583	0.01168346	0.01546193	0.00978655	0.00742724	0.00165274	0.00271496	0.00302502	0.00319162	0.0034315	0.00140149
ENSG00000106299	WASL	5.35346777	5.0918541	4.74798316	5.63188054	5.48968259	5.66435179	5.48506485	4.55189054	4.86230153	4.57345315	5.29869533	4.88453775	4.71907556
ENSG00000272686	RP11-390E23.6	3.07143164	2.55788933	2.80793423	3.28113378	3.09864115	3.68503622	3.16195158	2.11865509	2.15594703	2.13060778	3.02009703	2.80198698	2.08664599
ENSG00000165061	ZMAT4	0.14361449	0.09920721	0.12762732	0.14966479	0.12150449	0.27850311	0.08116749	1.50306337	2.79836656	3.10339104	0.55831536	0.60238435	3.61732757
ENSG00000153707	PTPRD	1.70743175	1.88564475	0.7314741	3.81260208	3.63940065	2.33169147	2.00860349	3.66121562	3.75535363	3.77536116	5.4871861	5.56756486	4.12678578
ENSG00000254519	CTD-2210P24.1	0.008626	0.00765771	0.00811232	0.0195553	0.01184534	0.01096623	0.01212839	0.00979413	0.01134084	0.0090386	0.0105732	0.01341029	0.01226287
ENSG00000173914	RBM4B	3.02555087	2.95565923	3.61994975	3.55138959	3.48560406	3.35832588	3.93821653	3.31568666	3.42770784	3.44369497	5.40227265	5.33469773	3.5490446
ENSG00000132950	ZMYM5	3.96339555	3.79649622	3.49637937	3.8160746	3.81443519	4.15372153	3.981388	2.21959955	2.20537584	2.26691539	3.50380774	3.29865736	2.39973197
ENSG00000233325	MIPEPP3	1.52087209	1.26268819	0.99469909	1.74464187	1.76076305	1.83153403	1.56646262	0.92514802	0.86094226	1.00594396	1.49486407	1.45055112	0.91204124
ENSG00000224429	LINC00422	0.48640764	0.45182097	0.33878897	0.58293599	0.64243158	0.5817681	0.66036051	0.45485312	0.48125374	0.5342512	1.10716873	1.07049222	0.56947975
ENSG00000235076	GAPDHP52	0.14569893	0.17294742	0.10902858	0.19708503	0.17938766	0.13818658	0.21305958	0.09912465	0.10334181	0.10897753	0.16202877	0.17734606	0.15028514
ENSG00000180776	ZDHHC20	4.24963372	4.09903139	3.615868	4.59106295	4.35391705	4.50349947	4.65004822	3.46336826	3.28687303	3.2450973	4.30341239	4.08323534	3.23099707
ENSG00000236953	ZDHHC20-IT1	0.50336495	0.49581895	0.3538848	0.51970033	0.48556406	0.39788372	0.70505184	0.25188413	0.2202556	0.30394733	0.73720465	0.87166886	0.36607022
ENSG00000226908	HIST1H2BPS3	0.1112689	0.08485735	0.05286777	0.03841847	0.06587986	0.05483827	0.04662937	0.03238982	0.02347497	0.02349042	0.10142314	0.08481983	0.02783302
ENSG00000165487	MICU2	4.56390359	4.43871557	4.95616509	4.54633556	4.65758808	4.70551212	4.59671943	3.92728718	4.09617998	4.08091965	5.48684747	5.42694787	4.19227267
ENSG00000230027	FNTAP2	0.13453266	0.12630892	0.0786857	0.18177584	0.14638235	0.12833797	0.11908646	0.04038759	0.03518706	0.04616191	0.1437674	0.32319735	0.06928077
ENSG00000207518	RNU6-59P	0.01724127	0.01036448	0.00648463	0.01020742	0.01194245	0.00524218	0	0.00474604	0.00228501	0.0053	0.00886409	0.01147508	0.00833371
ENSG00000102678	FGF9	0.67988553	1.99888175	2.5054854	0.55207571	0.79220609	0.36516589	1.66899767	1.15408503	2.11640964	1.70189334	4.79926473	4.58432491	2.51918135
ENSG00000232187	FTH1P7	2.77807886	2.53155495	2.16229484	2.4493162	2.50971371	2.47357146	2.1897211	2.12448973	1.9610883	1.46983102	1.23876133	1.28097197	1.97863189
ENSG00000226507	IPMKP1	0.00459407	0.0044613	0.0064568	0.00258515	0.00350399	0.0035878	0	0.01272607	0.02676041	0.01455017	0.01394014	0.019946	0.03391873
ENSG00000102683	SGCG	2.61460939	2.253637	0.4958849	2.9873533	2.08570117	1.24430442	2.12984501	0.36946814	0.83251539	0.5387636	0.97978037	1.03084991	1.30767423
ENSG00000151835	SACS	2.68407537	2.39537817	1.94880331	3.84407058	3.810015	3.84858845	3.18142363	2.20530258	2.57089683	2.76336141	2.57518302	2.85835427	2.82042074
ENSG00000232977	LINC00327	0.19140331	0.15577825	0.14917492	0.52638233	0.7472217	0.5863582	0.63885342	0.45856466	0.54513741	0.54749909	0.25448522	0.25465892	0.48491715
ENSG00000127863	TNFRSF19	1.82522525	1.7505134	1.4548519	1.19210285	2.86392961	2.0642671	2.9139372	0.9472962	0.96962978	1.16275527	1.02962277	1.23723728	0.97435113
ENSG00000252695	MIR2276	0.00656431	0.00571112	0.01760527	0.0058196	0.00925714	0.0049952	0	0.00243896	0.00199275	0.01468498	0.00577316	0.00802795	0.00129586
ENSG00000165501	LRR1	3.25530104	2.89582101	2.84598236	2.59785049	2.84889746	2.81598037	3.06279986	1.10200186	1.06973295	1.161643	1.81435498	1.74799006	1.19133071
ENSG00000165502	RPL36AL	5.67242534	5.67242534	5.67242534	5.67242534	5.67242534	5.67242534	5.67242534	5.67242534	5.67143994	5.66310217	5.67242534	5.67242534	5.67240423
ENSG00000168282	MGAT2	3.27253846	3.49461968	3.35910282	3.50453111	3.4338708	2.91501505	3.20262297	1.35929644	1.51081045	1.50935172	2.4047883	2.1728445	1.46615569
ENSG00000165516	KLHDC2	5.23420774	4.67704364	4.79838463	5.27334545	5.09361275	5.44613003	5.18805506	3.35314794	3.79525701	3.72301242	5.30868863	5.30130822	4.30832127
ENSG00000165525	NEMF	4.08307999	3.87224087	3.56898099	4.11716874	4.11603336	4.28913991	4.16201491	2.86497411	3.02724629	3.1075012	4.34344618	4.37418085	3.37991265
ENSG00000252474	RNU6-539P	0.1040222	0.06538091	0.03180044	0.08588117	0.05645102	0.07028154	0.03541573	0.01596185	0.00734822	0.01239213	0.03202033	0.04367124	0.01295601

ensg	symbol	Brain_Frontal_Cortex_BA9	Brain_Hippocampus	Brain_Hypothalamus	Brain_Nucleus_accumbens_basal_ganglia	Brain_Putamen_basal_ganglia	Brain_Spinal_cord_cervical_c-1	Brain_Substantia_nigra	Breast_Mammary_Tissue	Cells_Cultured_fibroblasts	Cells_EBV-transformed_lymphocytes	Cervix_Ectocervix	Cervix_Endocervix	Colon_Sigmoid	Colon_Transverse
ENSG00000224209	LINC00466	0.01369752	0.01618092	0.02511129	0.02189317	0.01666232	0.01331481	0.02019128	0.02614764	0.00222249	0.00475137	0.07693911	0.06992672	0.171262	0.0783686
ENSG00000145428	RNF175	3.69205092	2.27977112	2.99327485	3.03949518	2.27628854	1.36910207	1.86316571	0.97369705	0.76924733	0.03123688	0.85693145	1.55696791	0.75329815	0.55944052
ENSG00000145423	SFRP2	2.35820694	3.19190674	2.04064907	1.5122317	0.8513898	2.91832188	1.41038828	5.4095317	2.55972222	0.2996517	4.70531392	4.78108253	5.3363367	4.82132801
ENSG00000187391	MAGI2	2.59910502	2.06950761	2.11270698	2.22864667	2.25050929	2.29042688	1.95246522	1.16266842	0.89304591	0.04052271	1.39118527	1.61684371	1.25618401	0.84485129
ENSG00000234456	MAGI2-AS3	2.80045899	2.68759759	2.83527501	2.34727379	2.30773785	3.69438729	2.93501411	3.74255619	4.70067113	0.0530276	3.91049904	4.27246414	4.00181618	2.77381753
ENSG00000127955	GNAI1	4.73278269	4.67365203	4.70123361	3.97993147	3.83763513	5.54031236	4.7263619	4.90974845	4.97415432	0.2558742	4.72901378	4.38475023	4.95094625	4.34143176
ENSG00000233491	AC010091.1	0.00045721	0.0012307	0.00243733	0.0004267	0.00024987	0.0031465	0.00871007	0.00878948	0.16282778	0.0071377	0.02141222	0.02719565	0.00226479	0.00151117
ENSG00000019991	HGF	1.38905808	0.56924933	0.62080436	0.4322411	0.25581241	0.78057669	0.40815956	2.38306726	3.30695522	0.29571384	2.18980592	2.99930759	1.83224666	1.32620687
ENSG00000153956	CACNA2D1	4.07899976	3.32550684	3.89403942	3.28723869	2.67881465	2.80471846	2.40449997	2.88863444	4.19264296	0.06583128	3.06907628	3.31956304	4.43501634	2.83502174
ENSG00000164675	IQUB	0.27285951	0.39969756	0.57182649	0.31755597	0.15588161	0.27355642	0.29105443	0.7209044	0.33186301	0.11241907	0.66106178	0.99696075	0.31473589	0.18534466
ENSG00000232524	RP11-332K15.1	0.03415038	0.03424459	0.03597339	0.01960033	0.01372907	0.00980975	0.01778835	0.11121763	0.01267451	0.1458942	0.12522403	0.03435784	0.01947897	0.01041458
ENSG00000207338	RNU6-296P	0	0	0.00425284	0.00729903	0.00176691	0	0.00438264	0.0044845	0	0.002273	0	0	0.00125304	0
ENSG00000128609	NDUFA5	5.39067508	4.5832748	4.93053059	4.90854788	4.49685752	4.45493395	4.68177027	4.13545933	4.07479934	4.37433018	4.21432486	4.38118918	4.8302774	4.45043741
ENSG00000146809	ASB15	0.0144151	0.01587407	0.01514108	0.01057376	0.00965935	0.01694665	0.01103875	0.05883414	0.0140745	0.01184284	0.01405075	0.0284491	0.02811653	0.02544129
ENSG00000230442	RP11-390E23.3	0.00173167	0.00254155	0.00295442	0.00176351	0.00055251	0.00128555	0.00098771	0.00945673	0.00218379	0.00189182	0	0	0.00423609	0.00393275
ENSG00000106299	WASL	5.26789497	4.8014087	4.71736924	4.74747509	4.26353974	4.55370414	4.25226488	5.20947522	5.22492971	3.17867216	5.61097612	5.58338327	5.29674875	5.29243533
ENSG00000272686	RP11-390E23.6	2.26056722	2.13253036	2.51796235	2.10606794	1.95964988	2.6555679	2.27309056	3.20145692	2.13246685	2.11775192	3.43578788	3.47712413	2.98445035	2.97339926
ENSG00000165061	ZMAT4	3.76919048	1.86206285	1.87341647	2.92368954	2.88365751	0.74709078	1.01178908	0.19693669	0.1996731	0.01154661	0.46185859	0.70763686	0.93123662	0.45772293
ENSG00000153707	PTPRD	4.40927529	4.07289488	4.01380995	3.333112	3.68732685	5.13878924	4.11534306	1.24889162	0.91423525	0.04052364	3.26117222	3.44751781	1.21680961	2.12376807
ENSG00000254519	CTD-2210P24.1	0.01221212	0.01031725	0.01192164	0.01227772	0.00983921	0.00340649	0.01380852	0.02178055	0.00453407	0.00927425	0	0.03364928	0.0084466	0.00686191
ENSG00000173914	RBM4B	3.69604011	3.30106166	3.76114406	3.41984708	3.15969973	3.86443111	3.40628883	3.38096935	3.88239023	3.72126913	4.46748909	4.82151763	3.90166354	3.48065631
ENSG00000132950	ZMYM5	2.52756449	2.35358862	2.55964003	2.23341516	2.15722592	3.19438544	2.52742507	3.99704452	3.86695522	3.86783111	4.44524506	4.47526222	3.9518097	3.40765616
ENSG00000233325	MIPEPP3	0.98553384	1.03248077	1.27076999	0.99866743	0.79770207	1.36018639	0.99611272	1.68401411	1.51801379	1.22344347	2.23070668	2.12762626	1.45462356	1.17174241
ENSG00000224429	LINC00422	0.58089587	0.60215722	0.73107985	0.5576605	0.45358408	1.17881194	0.76533226	0.5421889	0.3690872	1.88604194	0.85823835	0.98830851	0.57176373	0.54618557
ENSG00000235076	GAPDHP52	0.11293013	0.17960472	0.20452861	0.10579293	0.09746196	0.47657575	0.23817388	0.22672306	0.12593121	0.18291055	0.32178719	0.34863431	0.15748689	0.13533913
ENSG00000180776	ZDHHC20	3.6742892	4.04190249	4.37637842	3.32919509	3.21210542	5.4878093	4.64942325	4.12097936	5.35438294	5.05142532	4.847347	4.59530292	4.15996008	4.16573238
ENSG00000236953	ZDHHC20-IT1	0.26462661	0.35599292	0.4447247	0.3286607	0.28077633	1.10759198	0.54528424	0.6342403	0.27669327	0.4755972	1.32979292	0.80324355	0.45984551	0.45456425
ENSG00000226908	HIST1H2BPS3	0.03614986	0.01204242	0.01625073	0.02186575	0.02387176	0.02620254	0.02335025	0.10937587	0.0454624	0.07596644	0.05123782	0.10492936	0.06058989	0.08356744
ENSG00000165487	MICU2	4.42816971	3.97038363	4.32757986	4.12806149	3.87317258	4.7308339	4.08810024	4.69714324	4.84762173	5.01913261	5.08021034	5.18991169	4.35584062	4.56270871
ENSG00000230027	FNTAP2	0.04617032	0.041492	0.04220331	0.0372921	0.0373124	0.10420811	0.03109511	0.15837379	0.08310895	0.13002236	0.33310722	0.16193175	0.13554765	0.09315889
ENSG00000207518	RNU6-59P	0.00563242	0.00844141	0	0.00411733	0.00252797	0.00152024	0.00226943	0.01211061	0.00466927	0.02430978	0	0.04752925	0.0048368	0.0091386
ENSG00000102678	FGF9	2.84781308	1.31794435	1.56400641	1.89425707	1.55314228	0.83234834	1.1241868	0.64451692	0.08722504	0.29498919	0.99424842	1.78079701	0.93427296	1.12721691
ENSG00000232187	FTH1P7	2.04700727	1.85062972	1.78536477	1.59103332	1.42543748	2.66274326	1.80460006	2.49228812	3.08527344	1.82208414	1.95792355	2.04523827	2.00109183	2.03148068
ENSG00000226507	IPMKP1	0.03112052	0.01425117	0.03409955	0.02691313	0.00633877	0.00683853	0.00708515	0.00489924	0.00579908	0.0049512	0	0.15524211	0.00539769	0.00709562
ENSG00000102683	SGCG	1.47984498	0.6759548	0.58053358	0.57176502	0.43467408	0.40517876	0.38181131	1.72558946	0.70991151	0.0980297	0.70130085	0.63472401	1.47138707	1.02698033
ENSG00000151835	SACS	3.06711832	2.34195332	2.43598745	2.78276915	2.50377331	3.00046556	2.38789639	2.51893173	4.50000829	4.01504321	2.55595768	2.91751123	3.05355244	2.11243661
ENSG00000232977	LINC00327	0.59710117	0.4759295	0.76787012	0.73105817	0.45795805	0.62144032	0.49100576	0.27908923	0.60239204	0.21967235	0.45734416	0.55233665	0.4967723	0.27302419
ENSG00000127863	TNFRSF19	1.02885004	1.26597872	1.45768	1.06282865	0.98156684	1.4745988	1.25477414	2.14329136	2.87605904	2.29851512	3.08726317	3.33273258	2.10530267	1.59071527
ENSG00000252695	MIR2276	0.00859506	0.00272411	0.00274233	0.00358433	0.0093051	0.00884516	0.00851257	0.03415879	0.00429673	0.00255678	0	0	0.00731433	0.01330484
ENSG00000165501	LRR1	1.28449606	1.01508396	1.18244573	1.15609211	1.10304217	1.44846994	1.37992056	3.08020344	3.96924966	4.92818195	2.88817477	2.997322	3.12469577	3.41418625
ENSG00000165502	RPL36AL	5.67242534	5.66952369	5.67242534	5.65602644	5.67114918	5.67242534	5.67242534	5.67242534	5.67242534	5.67242534	5.67242534	5.67242534	5.67242534	5.67242534
ENSG00000168282	MGAT2	1.71670155	1.29111555	1.61541429	1.59943687	1.35543923	1.78055358	1.41013386	3.10507938	5.1684339	5.11458761	3.03815176	3.39203051	2.65321281	3.28674557
ENSG00000165516	KLHDC2	4.38378777	3.36879107	3.80671778	3.8004214	3.44171829	3.89621307	3.51904544	5.14323233	4.68075705	3.89345494	5.40313116	5.5453237	5.26990117	5.01856832
ENSG00000165525	NEMF	3.475113	2.97685854	3.30541255	3.0689121	2.87850056	4.04044038	3.23911206	4.0594291	4.65130773	4.55206281	4.41820388	4.48125443	4.01815904	3.72549389
ENSG00000252474	RNU6-539P	0.01562216	0.01672737	0.01679697	0.01288821	0.01613428	0.03834004	0.01044155	0.11842912	0.03268028	0.06258766	0.25452151	0.11415729	0.04828196	0.05955049

ensg	symbol	Esophagus_Gastroesophageal_Junction	Esophagus_Mucosa	Esophagus_Muscularis	Fallopian_Tube	Heart_Atrial_Appendage	Heart_Left_Ventricle	Kidney_Cortex	Kidney_Medulla	Liver	Lung	Minor_Salivary_Gland	Muscle_Skeletal
ENSG00000224209	LINC00466	0.11130837	0.01579719	0.11545547	0.07583902	0.02764851	0.00705618	0.00958972	0	0.00300113	0.00452283	0.0171426	0.028826
ENSG00000145428	RNF175	0.58231155	0.43063537	0.61350447	0.785118	1.51027492	0.31115707	0.24893466	0.21809471	0.26819437	1.50853371	1.2990429	0.118293
ENSG00000145423	SFRP2	5.47387999	5.4566629	5.38890419	5.34737325	1.9392845	0.75859119	1.5155027	1.43961206	0.29852308	4.89590272	5.49630395	1.753993
ENSG00000187391	MAGI2	1.38452429	0.43175759	1.44741817	1.52552889	0.56975595	0.29264098	0.94832623	0.95761161	0.21173859	0.98733607	0.6084683	0.32222
ENSG00000234456	MAGI2-AS3	4.22423667	1.7824222	4.25648335	3.9737271	3.76957642	2.87612003	3.52857121	2.88515283	2.03659938	3.48838417	2.82240507	1.80258
ENSG00000127955	GNAI1	5.00342562	4.33592482	4.93043088	4.14828404	2.20912987	2.21347176	3.61085217	4.86299302	2.94038703	4.3900423	3.49438745	2.051877
ENSG00000233491	AC010091.1	0.00487017	0.00139033	0.00693576	0.03072622	0.00288624	0.00425116	0.00324083	0.00731643	0.02018869	0.00920545	0.0279037	0.001279
ENSG00000019991	HGF	1.87428073	0.92027612	1.93842834	2.5708214	1.50310112	0.82211891	2.05929296	2.6981665	2.60680339	4.07922581	1.4458628	0.381042
ENSG00000153956	CACNA2D1	3.586119	1.50486908	3.74286786	3.22646422	3.19581792	2.86703454	1.78217866	1.71885382	0.53014154	2.72390835	2.11066386	5.122825
ENSG00000164675	IQUB	1.24750768	0.11063874	1.49853952	1.60879571	0.20366452	0.09643922	1.07375468	1.43587082	0.06533664	0.95513085	0.4459136	0.18419
ENSG00000232524	RP11-332K15.1	0.20683429	0.00209433	0.28919753	0.17853894	0.02313522	0.00594942	0.17424877	0.40268923	0.00127006	0.08440351	0.05144184	0.015295
ENSG00000207338	RNU6-296P	0.00672442	0.00075495	0.00349787	0	0.00321879	0.00119403	0.00962992	0.08677467	0	0.00541618	0.00217473	0
ENSG00000128609	NDUFA5	4.85806461	3.80673783	4.95729776	4.4787025	4.86465157	4.41392888	4.38068526	4.87882402	3.60231819	4.08005095	4.13243589	5.202579
ENSG00000146809	ASB15	0.02607337	0.01366538	0.0412255	0.02332847	3.11137779	3.17284435	0.87682154	0.24379599	0.02195927	0.04646238	0.36024113	3.698432
ENSG00000230442	RP11-390E23.3	0.00610048	0.00204389	0.00673796	0.01264524	0.47179794	0.43663815	0.0409795	0.00492904	0.00471145	0.01141036	0.02681501	0.902595
ENSG00000106299	WASL	5.30070651	5.15029767	5.26617326	5.3653246	4.07791369	3.3140717	4.81894536	4.95962932	4.20427065	5.26627422	4.95263664	4.441164
ENSG00000272686	RP11-390E23.6	3.069224	2.86408302	3.00631288	3.50016676	1.87482558	1.47064385	2.33329603	2.87368993	1.80084856	2.9143961	2.54107004	2.51911
ENSG00000165061	ZMAT4	0.21475046	0.02801136	0.39108056	0.09201836	0.08297872	0.23001428	0.12327983	0.42975745	0.03150728	0.08851804	0.02545921	0.017391
ENSG00000153707	PTPRD	1.93699878	0.80197112	1.63382556	2.32153021	1.19091471	1.13368629	2.57781257	1.45755289	1.56005701	3.09331933	0.71373537	0.658032
ENSG00000254519	CTD-2210P24.1	0.00613724	0.00757211	0.01193983	0.02055378	0.00516517	0.00417161	0.00441116	0.00850109	0.01299292	0.01028409	0.00998079	0.620729
ENSG00000173914	RBM4B	3.61329922	3.48104145	3.48660939	4.34775115	2.43553751	2.02492687	3.05243597	3.54129116	2.59461166	3.36133253	3.72324298	2.368908
ENSG00000132950	ZMYM5	3.6084379	3.75452469	3.56889852	4.09312598	2.72869805	2.02067409	2.86663144	3.21983016	2.45564387	3.932712	3.53915753	2.660068
ENSG00000233325	MIPEPP3	1.35244787	1.81517328	1.34080811	2.07415732	0.84956766	0.4570197	1.08199445	0.9254062	0.36735622	1.46644528	1.65211982	0.418864
ENSG00000224429	LINC00422	0.52026115	0.58257556	0.50471038	1.10261414	0.23760086	0.15697116	0.40653759	0.59343722	0.18531665	0.88673728	0.6310366	0.115443
ENSG00000235076	GAPDHP52	0.13852118	0.32301089	0.13051132	0.31094767	0.13619765	0.08755933	0.14783246	0.26856009	0.0576704	0.43742809	0.22779244	0.061855
ENSG00000180776	ZDHHC20	3.94810534	5.10040072	3.94684737	4.44515867	2.79908936	2.14417211	2.63525114	2.88450478	2.90392663	4.84522726	4.33729913	2.36671
ENSG00000236953	ZDHHC20-IT1	0.44470496	0.75439071	0.40233689	0.91955946	0.35236907	0.18642136	0.37767873	0.43769036	0.29992883	0.97929556	0.83372845	0.163214
ENSG00000226908	HIST1H2BPS3	0.06374161	0.04290564	0.05688193	0.18829588	0.03078682	0.02790402	0.07859147	0.02954009	0.02491602	0.15422839	0.04129102	0.084087
ENSG00000165487	MICU2	4.21071051	4.45586246	4.15882764	4.95252542	4.33037655	3.9114258	4.57116874	5.05128653	3.53055917	4.61918108	4.39358587	4.588963
ENSG00000230027	FNTAP2	0.12787006	0.10092636	0.1239374	0.20518398	0.08913421	0.04150963	0.0714422	0.01443361	0.03997173	0.18579038	0.15257088	0.054523
ENSG00000207518	RNU6-59P	0.00496307	0.00860792	0.00607315	0	0.00567979	0.00753058	0.00896651	0	0.01357507	0.01162942	0.0140777	0.001661
ENSG00000102678	FGF9	1.27970077	0.34140829	1.07706304	1.08594682	1.97277758	1.59433266	2.88588894	3.71987825	0.04321725	1.37147511	0.65639138	0.55901
ENSG00000232187	FTH1P7	2.12139034	1.70078333	2.02255315	1.91594512	1.70065141	1.33201877	1.88934782	1.6606123	1.06538972	2.90387429	1.85622761	1.523669
ENSG00000226507	IPMKP1	0.00422082	0.00651735	0.00556365	0.02939961	0.00146317	0.00238481	0.01088125	0.04671109	0.00274213	0.00440017	0.01529928	0.00341
ENSG00000102683	SGCG	2.34734004	0.78957571	2.16907533	1.65546472	5.29196239	5.01885704	0.29119369	0.45130643	0.0337581	3.01335953	1.88389857	5.236451
ENSG00000151835	SACS	3.68083545	1.94205975	3.73640286	3.14234211	2.23555992	1.79353811	1.12375688	1.44676547	0.77149605	2.62536264	2.82440352	3.04022
ENSG00000232977	LINC00327	0.85113219	0.43004704	0.90678155	0.69060426	0.94438202	0.51729473	0.04203069	0.05514163	0.01877002	0.57697714	1.12379261	0.447991
ENSG00000127863	TNFRSF19	2.7319916	3.08004807	2.3908363	4.16261699	3.96275554	3.07756055	2.7366199	2.50767499	0.4150253	3.92368212	4.80330138	0.958026
ENSG00000252695	MIR2276	0.00879332	0.00201487	0.00075387	0	0.00571859	0.00248164	0.04410151	0	0.01182377	0.03161658	0.00814815	0.001744
ENSG00000165501	LRR1	2.95249739	2.99635307	3.12859257	3.00696966	1.76394117	1.44170947	1.83267229	2.45276817	1.93380149	3.00499821	2.60063876	1.67345
ENSG00000165502	RPL36AL	5.67242534	5.67242534	5.67242534	5.67242534	5.67087876	5.5716065	5.67242534	5.67242534	5.6652433	5.67242534	5.67242534	5.672425
ENSG00000168282	MGAT2	2.82453661	3.05243001	2.88902784	3.55858274	2.16268552	1.64383963	1.97538195	1.98693281	2.94840122	3.07055056	3.1858447	1.327643
ENSG00000165516	KLHDC2	5.09469113	4.36640939	5.11285777	5.34076169	4.29344072	3.71240627	4.425464	4.39223292	4.3714303	4.76691835	5.00945319	4.878482
ENSG00000165525	NEMF	3.99931382	3.97592464	3.99509957	4.32413981	3.06520572	2.48788298	2.9014205	3.36303225	2.31238432	4.08123361	3.93221716	3.685505
ENSG00000252474	RNU6-539P	0.03676416	0.05289552	0.05009356	0.18600486	0.03655555	0.00951437	0.00415397	0	0.01866866	0.12709157	0.08680241	0.019403

ensg	symbol	Nerve_Tibial	Ovary	Pancreas	Pituitary	Prostate	Skin_Not_Sun_Exposed_Suprapubic	Skin_Sun_Exposed_Lower_leg	Small_Intestine_Terminal_Ileum	Spleen	Stomach	Testis	Thyroid	Uterus	Vagina	Whole_Blood
ENSG00000224209	LINC00466	0.811963	0.015648	0.005638	0.016777	0.08819318	0.01984395	0.02605722	0.04783478	0.00309785	0.03643457	2.80173039	0.014218	0.019515	0.09442752	0.00106815
ENSG00000145428	RNF175	1.30335	0.253703	0.153819	2.685961	1.78184339	0.71800482	0.73086429	0.71878998	1.9251386	0.36913915	1.5837953	0.487809	0.346275	1.43003773	2.56540105
ENSG00000145423	SFRP2	5.473745	1.700391	3.760095	5.128153	5.29272335	5.39628978	5.07890407	4.71796564	0.8106693	3.79787505	1.68361059	5.432175	3.403675	5.5228681	1.38127902
ENSG00000187391	MAGI2	2.492315	1.719092	0.305574	1.925654	1.55376967	0.47709755	0.58570963	0.7259817	0.70205894	0.69770113	1.10915382	1.622344	1.515183	1.14751386	0.02032404
ENSG00000234456	MAGI2-AS3	4.540776	4.675358	0.916175	3.722052	3.58763282	1.7226863	1.92202818	2.47413574	3.29788527	2.23901473	1.91700032	3.949971	4.306543	3.36073156	0.15812371
ENSG00000127955	GNAI1	5.249072	4.655006	1.653696	3.837723	3.63472648	5.372573	5.45671311	3.42803543	2.8958763	4.06949608	4.27399358	4.421002	4.521371	4.78376091	0.24311289
ENSG00000233491	AC010091.1	0.005555	0.007702	0.000325	0.014968	0.00795566	0.00914674	0.00392056	0.00154528	0.00091759	0.0013822	0.1449739	0.041921	0.044971	0.00549224	0.00036758
ENSG00000019991	HGF	2.648243	1.196284	0.55889	1.660085	2.58285618	0.59881858	0.71608575	1.40770011	2.17428154	1.28075047	1.25239274	2.851956	2.509853	2.63999347	1.43009657
ENSG00000153956	CACNA2D1	3.292884	3.445567	0.588599	3.683677	3.16595759	2.35243215	2.19424752	2.22014689	2.14193815	2.47532934	2.37873024	3.976294	3.632868	2.80988796	0.0695946
ENSG00000164675	IQUB	0.763293	0.681862	0.230921	1.52133	0.64580052	0.24629727	0.26497353	0.16298498	0.12692139	0.23740067	5.02611261	1.818564	1.116155	0.45583924	0.04262363
ENSG00000232524	RP11-332K15.1	0.084212	0.078038	0.013914	0.133862	0.06505059	0.29065062	0.36981366	0.01209381	0.00671498	0.01301923	0.3609757	0.264688	0.150847	0.05789366	0.00219385
ENSG00000207338	RNU6-296P	0.008891	0.003234	0	0.015614	0.00186537	0.00576839	0.00970335	0	0	0.00203575	0.05267335	0.020877	0	0	0
ENSG00000128609	NDUFA5	4.201506	4.37953	2.818337	4.84622	4.49600578	3.47536441	3.49037304	4.11022764	3.61683806	4.3257653	4.30614518	5.114304	4.560072	4.177385	1.03592929
ENSG00000146809	ASB15	0.045488	0.032409	0.020497	0.0778	0.07004919	0.04334596	0.0502649	0.02978193	0.03385082	0.03639683	1.82012288	0.044371	0.031738	0.02730055	0.01952034
ENSG00000230442	RP11-390E23.3	0.006942	0.006385	0.001203	0.004955	0.01322412	0.00473165	0.00525837	0.00416096	0.00324253	0.02196754	2.2757536	0.005175	0.005282	0.0025608	0.00163077
ENSG00000106299	WASL	5.565554	5.642026	3.640163	4.833751	5.17443288	4.84475676	4.96252157	4.87388506	4.20274258	4.70214507	5.1152329	5.448556	5.551976	5.51912643	1.38545044
ENSG00000272686	RP11-390E23.6	3.477713	3.448984	1.598054	3.456834	3.04225397	2.91611717	2.98955545	2.57523981	2.82302171	2.49737953	3.42782626	3.655393	3.424186	3.28085436	0.64936341
ENSG00000165061	ZMAT4	0.047498	0.71563	0.069477	1.835415	0.86415131	0.13502143	0.1749331	0.15019556	0.47307743	0.24308362	1.11401555	3.796419	0.295581	0.13252504	0.08459932
ENSG00000153707	PTPRD	1.177327	4.283929	0.805908	2.880046	1.94578365	0.79315343	0.98958104	2.68203044	0.7449147	0.98441572	2.12499755	2.70655	2.637646	2.5125789	0.03794621
ENSG00000254519	CTD-2210P24.1	0.008627	0.009087	0.004542	0.012911	0.02839639	0.01450863	0.01513441	0.00926491	0.01093938	0.00799611	5.20996875	0.017869	0.012216	0.01116634	0.00442922
ENSG00000173914	RBM4B	4.071543	4.85682	2.524953	4.664648	4.3818556	3.53608562	3.46722977	3.74959718	3.95889482	3.24734335	4.39625468	4.353321	4.638569	4.12079522	1.86512603
ENSG00000132950	ZMYM5	4.330201	4.289367	2.27738	3.833563	3.96499934	3.51981423	3.517102	3.54821508	3.667479	3.10660396	4.72117257	4.323364	4.184123	4.18049638	2.33389502
ENSG00000233325	MIPEPP3	2.182845	2.10605	0.679087	2.072487	1.80461341	1.50238257	1.45861391	1.11893105	1.09392456	1.04379285	2.06577752	2.315773	2.242254	1.88451718	0.22707526
ENSG00000224429	LINC00422	0.987139	0.930847	0.235693	1.133352	0.76223258	0.51861902	0.50044279	0.79704122	1.45477895	0.49542405	2.39612956	1.147473	0.934078	0.72427693	1.15263367
ENSG00000235076	GAPDHP52	0.463535	0.289093	0.080353	0.383991	0.27340286	0.2735465	0.23555914	0.20973045	0.38063477	0.17146595	3.36778517	0.372793	0.318218	0.26193224	0.41981201
ENSG00000180776	ZDHHC20	4.645686	4.181555	2.655707	4.3533	4.27141694	4.74168954	4.66619203	4.35852585	4.44051383	4.31620499	4.87306061	4.490189	4.662981	4.99156539	4.49695563
ENSG00000236953	ZDHHC20-IT1	1.12605	0.843467	0.320963	0.854994	0.77373485	0.84375433	0.77487838	0.59621671	0.75646947	0.65477771	0.94432832	1.021421	0.843008	1.09383869	0.58653745
ENSG00000226908	HIST1H2BPS3	0.074937	0.057641	0.034679	0.120329	0.11083584	0.07674669	0.08017469	0.15155766	0.20350655	0.05297435	0.39210954	0.095323	0.126956	0.10113907	0.06526328
ENSG00000165487	MICU2	5.045323	4.867282	3.384226	5.196713	4.63001462	4.39239717	4.34686183	4.64666989	4.61283675	4.24176796	4.81780386	5.195669	5.026222	4.82645337	3.10391072
ENSG00000230027	FNTAP2	0.345137	0.331633	0.059455	0.191581	0.1942242	0.23497657	0.22376981	0.12759065	0.09577379	0.08363085	0.21255411	0.226829	0.265373	0.27839507	0.05002741
ENSG00000207518	RNU6-59P	0.031531	0.025148	0.004229	0.017002	0.01851385	0.01963342	0.01817711	0.02562682	0.01660995	0.00826083	0.02273716	0.020017	0.019396	0.02065743	0.01434316
ENSG00000102678	FGF9	1.228062	1.529558	0.425336	2.46358	0.62119233	0.26447186	0.35810445	1.40624275	0.24811374	0.72762731	1.518242	0.537122	1.003829	1.42705721	0.15967573
ENSG00000232187	FTH1P7	3.146864	2.017215	1.180445	1.572877	1.79676477	1.37561816	1.52954977	1.98677413	1.82981524	1.75820421	1.71622266	2.200684	2.011912	1.86688021	3.19032519
ENSG00000226507	IPMKP1	0.014323	0.019086	0.002403	0.017097	0.00798463	0.00354155	0.00481052	0.00410487	0.00331904	0.0054779	0.19454921	0.021545	0.020809	0.00677134	0.00316275
ENSG00000102683	SGCG	1.117326	0.475597	0.199341	0.76621	2.20467685	1.77882431	2.49063487	0.86447476	0.29902292	0.91444333	2.37269142	0.375743	0.787916	1.57723923	0.10652764
ENSG00000151835	SACS	3.127232	2.719783	1.584836	2.891922	2.83584215	1.69918986	1.75819144	2.48404707	3.02003194	2.16162389	3.24970502	2.108941	3.418996	2.46379605	0.40525783
ENSG00000232977	LINC00327	1.572896	0.324767	0.048575	0.757596	0.95066868	1.01338411	0.89020917	0.2309758	0.30866811	0.2690561	1.48813259	0.091671	0.539851	0.42558146	0.01316614
ENSG00000127863	TNFRSF19	4.587492	2.291903	1.359659	3.600813	4.07784607	5.53173718	5.42344136	1.43622669	2.3327526	2.019827	2.10346799	2.578583	2.657685	3.05629081	0.08949109
ENSG00000252695	MIR2276	0.048795	0.04582	0.052317	0.015293	0.07449741	0.0277805	0.02221731	0.03140553	0.01389774	0.0113887	0.00923694	0.026294	0.007921	0	0.00221711
ENSG00000165501	LRR1	3.105759	2.648564	1.851514	1.786563	2.83046707	2.79773369	2.85523642	3.30702586	3.58214057	2.61436269	5.3745897	2.628933	3.081857	2.82957445	1.9725683
ENSG00000165502	RPL36AL	5.672425	5.672425	5.672425	5.672425	5.67242534	5.67242534	5.67242534	5.67242534	5.67242534	5.67242534	5.67242534	5.672425	5.672425	5.67242534	5.66272006
ENSG00000168282	MGAT2	2.69051	3.259294	3.012314	2.567547	2.76910554	2.54669458	2.57072758	3.57419357	3.59645386	3.08382855	4.46681556	2.96306	3.280169	2.98726906	2.16253527
ENSG00000165516	KLHDC2	5.351261	5.560251	4.514374	5.181234	5.04805167	4.97017585	5.07532264	5.08562814	4.6642409	5.08278426	5.3218879	5.601703	5.463166	5.14774119	2.72471119
ENSG00000165525	NEMF	4.448	4.448961	2.572543	4.143315	4.00666695	3.90569376	4.02072214	3.9371701	3.89759895	3.50677253	3.77355268	4.290156	4.548462	4.31749169	1.90520592
ENSG00000252474	RNU6-539P	0.113411	0.100744	0.036252	0.054362	0.03949146	0.19671666	0.16112085	0.09486187	0.08087042	0.05230775	0.03596976	0.088157	0.100303	0.08179302	0.04729009

Tissue expression of prioritized genes are average of log2 transformed (transcripts per million) TPM after winsolizing at 50 per tissue (54 general

Supplementary Table S5. Minor allele frequencies of cases and control design for 4 CAA phenotype

SNP	Chr	Major Allele	Minor Allele	Total Sample (N) - All	Total Sample (MAF) - All	CAA/L (N) - All Cases	CAA/L (MAF) - All Cases	No CAA (N) - Total Controls	No CAA (MAF) - Total Controls	Total Sample (N) - White	Total Sample (MAF) - White	CAA/L (N) - CAU Cases	CAA/L (MAF) - CAU Cases	No CAA (N) - CAU Controls	No CAA (MAF) - CAU Controls	Total Sample (N) - AA	Total Sample (MAF) - AA	CAA/L (N) - AA Cases	CAA/L (MAF) - AA Cases	No CAA (N) - AA Controls	No CAA (MAF) - AA Controls	Total Sample (N) - HIS	Total Sample (MAF) - HIS	CAA/L (N) - His Cases	CAA/L (MAF) - His Cases	No CAA (N) - His Controls	No CAA (MAF) - His Controls	Total Sample (N) - AsPI	Total Sample (MAF) - AsPI	CAA/L (N) - AsPI Cases	CAA/L (MAF) - AsPI Cases	No CAA (N) - AsPI Controls	No CAA (MAF) - AsPI Controls
rs72863358	1	T	A	369	0.1369	91	0.2418	278	0.1025	212	0.1415	52	0.2308	160	0.1125	32	0.07812	3	0.3333	29	0.05172	50	0.25	20	0.325	30	0.2	75	0.07333	16	0.1562	59	0.05085
rs377203	1	T	C	367	0.1253	91	0.2088	276	0.09783	211	0.109	52	0.2019	159	0.07862	31	0.1935	3	0.3333	28	0.1786	50	0.15	20	0.225	30	0.1	75	0.1267	16	0.1875	59	0.1102
rs1754156	1	G	A	369	0.126	91	0.2088	278	0.09892	212	0.1108	52	0.2115	160	0.07812	32	0.2344	3	0.3333	29	0.2241	50	0.13	20	0.2	30	0.08333	75	0.12	16	0.1875	59	0.1017
rs634288	1	A	G	369	0.1247	91	0.2088	278	0.09712	212	0.1108	52	0.2115	160	0.07812	32	0.2188	3	0.3333	29	0.2069	50	0.13	20	0.2	30	0.08333	75	0.12	16	0.1875	59	0.1017
rs1328589	1	T	G	369	0.3591	91	0.2253	278	0.4029	212	0.3302	52	0.1923	160	0.375	32	0.375	3	0	29	0.4138	50	0.36	20	0.35	30	0.3667	75	0.4333	16	0.2188	59	0.4915
rs1614816	1	C	G	369	0.397	91	0.3242	278	0.4209	212	0.4858	52	0.4231	160	0.4562	32	0.2656	3	0	29	0.2931	50	0.41	20	0.325	30	0.4667	75	0.1133	16	0.0625	59	0.1271
rs4353040	1	T	C	369	0.3957	92	0.3207	277	0.4206	213	0.4906	53	0.4151	160	0.4594	32	0.2656	3	0	29	0.2931	50	0.41	20	0.325	30	0.4667	74	0.1149	16	0.0625	58	0.1293
rs4949849	1	A	G	368	0.3913	91	0.3242	277	0.4134	211	0.4858	52	0.4231	159	0.456	32	0.2031	3	0	29	0.2241	50	0.41	20	0.325	30	0.4667	75	0.1133	16	0.0625	59	0.1271
rs1913124	1	C	T	363	0.3884	90	0.3167	273	0.4121	209	0.4904	51	0.4118	158	0.4589	31	0.1935	3	0	28	0.2143	49	0.4082	20	0.325	29	0.4655	74	0.1149	16	0.0625	58	0.1293
rs1249826	1	G	A	367	0.3992	91	0.3242	276	0.4239	210	0.481	52	0.4231	158	0.4494	32	0.2656	3	0	29	0.2931	50	0.41	20	0.325	30	0.4667	75	0.1133	16	0.0625	59	0.1271
rs1249825	1	C	A	366	0.3921	90	0.3167	276	0.4167	209	0.4833	51	0.4118	158	0.4494	32	0.2031	3	0	29	0.2241	50	0.41	20	0.325	30	0.4667	75	0.1133	16	0.0625	59	0.1271
rs1249821	1	T	C	369	0.3902	92	0.3207	277	0.4134	213	0.4906	53	0.4151	160	0.4594	32	0.2031	3	0	29	0.2241	50	0.41	20	0.325	30	0.4667	74	0.1149	16	0.0625	58	0.1293
rs1249816	1	T	A	366	0.3989	91	0.3242	275	0.4236	210	0.4833	52	0.4231	158	0.4525	32	0.2656	3	0	29	0.2931	50	0.41	20	0.325	30	0.4667	74	0.1149	16	0.0625	58	0.1293
rs1249815	1	G	A	369	0.3957	91	0.3242	278	0.4191	212	0.4882	52	0.4231	160	0.4594	32	0.2656	3	0	29	0.2931	50	0.41	20	0.325	30	0.4667	75	0.1133	16	0.0625	59	0.1271
rs1249814	1	C	A	369	0.3943	92	0.3207	277	0.4188	213	0.4906	53	0.4151	160	0.4594	32	0.2656	3	0	29	0.2931	49	0.4082	20	0.325	29	0.4655	75	0.1133	16	0.0625	59	0.1271
rs1249811	1	G	A	369	0.3957	91	0.3242	278	0.4191	212	0.4882	52	0.4231	160	0.4594	32	0.2656	3	0	29	0.2931	50	0.41	20	0.325	30	0.4667	75	0.1133	16	0.0625	59	0.1271
rs6691590	1	G	C	370	0.3878	92	0.3152	278	0.4119	213	0.4906	53	0.4151	160	0.4594	32	0.2031	3	0	29	0.2241	50	0.4	20	0.3	30	0.4667	75	0.1133	16	0.0625	59	0.1271
rs1249780	1	C	T	370	0.3905	92	0.3207	278	0.4137	213	0.4883	53	0.4151	160	0.4562	32	0.2031	3	0	29	0.2241	50	0.41	20	0.325	30	0.4667	75	0.1133	16	0.0625	59	0.1271
rs1249779	1	T	G	369	0.3902	92	0.3207	277	0.4134	213	0.4906	53	0.4151	160	0.4594	32	0.2031	3	0	29	0.2241	50	0.41	20	0.325	30	0.4667	74	0.1149	16	0.0625	58	0.1293
rs1249795	1	C	T	370	0.3892	92	0.3207	278	0.4119	213	0.4906	53	0.4151	160	0.4594	32	0.2031	3	0	29	0.2241	50	0.41	20	0.325	30	0.4667	75	0.1133	16	0.0625	59	0.1271
rs1249801	1	T	G	369	0.3902	92	0.3207	277	0.4134	213	0.4906	53	0.4151	160	0.4594	32	0.2031	3	0	29	0.2241	50	0.41	20	0.325	30	0.4667	74	0.1149	16	0.0625	58	0.1293
rs1249785	1	A	T	370	0.3905	92	0.3207	278	0.4137	213	0.4883	53	0.4151	160	0.4562	32	0.2031	3	0	29	0.2241	50	0.41	20	0.325	30	0.4667	75	0.1133	16	0.0625	59	0.1271
rs6672579	1	A	G	369	0.3875	92	0.3152	277	0.4116	213	0.493	53	0.4151	160	0.4625	32	0.2031	3	0	29	0.2241	50	0.4	20	0.3	30	0.4667	74	0.1149	16	0.0625	58	0.1293
rs1356379875	1	G	C	369	0.2304	91	0.1429	278	0.259	212	0.2524	52	0.1827	160	0.275	32	0.2031	3	0	29	0.2241	50	0.16	20	0.075	30	0.2167	75	0.2267	16	0.125	59	0.2542
rs1383193086	1	G	A	369	0.2249	91	0.1319	278	0.2554	212	0.2429	52	0.1635	160	0.2688	32	0.2031	3	0	29	0.2241	50	0.16	20	0.075	30	0.2167	75	0.2267	16	0.125	59	0.2542
rs1369301042	1	G	T	369	0.2222	91	0.1319	278	0.2518	212	0.2429	52	0.1635	160	0.2688	32	0.1875	3	0	29	0.2069	50	0.16	20	0.075	30	0.2167	75	0.22	16	0.125	59	0.2458
rs1295606189	1	A	G	367	0.436	90	0.3944	277	0.4495	210	0.431	51	0.3922	159	0.4434	32	0.4375	3	0.5	29	0.431	50	0.45	20	0.425	30	0.4667	75	0.44	16	0.3438	59	0.4661
rs1226978594	1	A	T	367	0.436	90	0.3944	277	0.4495	210	0.431	51	0.3922	159	0.4434	32	0.4375	3	0.5	29	0.431	50	0.45	20	0.425	30	0.4667	75	0.44	16	0.3438	59	0.4661
rs151019013	1	G	GGTT	362	0.3108	87	0.1724	275	0.3545	206	0.3471	49	0.2347	157	0.3822	32	0.1562	3	0	29	0.1724	49	0.2755	19	0.1316	30	0.3667	75	0.3	16	0.0625	59	0.3644
rs1255965559	2	G	A	369	0.2778	91	0.1923	278	0.3058	212	0.2642	52	0.1923	160	0.2875	32	0.2969	3	0	29	0.3276	50	0.24	20	0.15	30	0.3	75	0.3333	16	0.2812	59	0.3475
rs76773210	2	T	C	370	0.1135	92	0.1848	278	0.08993	213	0.1197	53	0.1792	160	0.1	32	0.1406	3	0.3333	29	0.1207	50	0.13	20	0.2	30	0.08333	75	0.07333	16	0.1562	59	0.05085
rs4059845	2	G	A	369	0.3347	91	0.4341	278	0.3022	212	0.3349	52	0.4135	160	0.3094	32	0.2812	3	0.5	29	0.2586	50	0.36	20	0.45	30	0.3	75	0.34	16	0.4688	59	0.3051
rs62154092	2	T	C	369	0.3997	92	0.2989	277	0.4332	213	0.3967	53	0.3113	160	0.425	32	0.4062	3	0.1667	29	0.431	50	0.36	20	0.275	30	0.4167	74	0.4324	16	0.3125	58	0.4655
rs1342924635	2	A	G	368	0.2473	90	0.15	278	0.2788	211	0.2488	51	0.1569	160	0.2781	32	0.2656	3	0	29	0.2931	50	0.26	20	0.15	30	0.3333	75	0.2267	16	0.1562	59	0.2458
rs10490562	2	C	T	368	0.1916	91	0.2967	277	0.157	212	0.1934	52	0.2981	160	0.1594	32	0.2031	3	0.5	29	0.1724	50	0.17	20	0.25	30	0.1167	74	0.1959	16	0.3125	58	0.1638
rs4664397	2	A	G	367	0.1689	91	0.2582	276	0.1395	211	0.1943	52	0.3077	159	0.1572	32	0.0625	3	0	29	0.06897	50	0.13	20	0.2	30	0.08333	74	0.1689	16	0.2188	58	0.1552
rs4541223	2	T	C	369	0.07317	92	0.1087	277	0.06137	213	0.05164	53	0.06604	160	0.04688	32	0.1562	3	0.3333	29	0.1379	50	0.01	20	0.025	30	0	74	0.1419	16	0.3125	58	0.09483
rs2628010	3	T	A	363	0.3444	91	0.2363	272	0.3805	209	0.2536	52	0.1731	157	0.2803	31	0.4194	3	0.3333	28	0.4286	50	0.38	20	0.375	30	0.3833	73	0.4795	16	0.1875	57	0.4386
rs10652766	3	AATT	A	369	0.2276	92	0.337	277	0.1913	213	0.277	53	0.4245	160	0.2281	32	0.1875	3	0.3333	29	0.1724	49	0.2347	20	0.3	29	0.1897	75	0.1	16	0.09375	59	0.1017
rs10451979	3	C	G	370	0.2986	92	0.1848	278	0.3363	213	0.2958	53	0.1981	160	0.3281	32	0.3594	3	0.1667	29	0.3793	50	0.2	20	0.1	30	0.2667	75	0.3467	16	0.25	59	0.3729
rs72909180	3	G	A	368	0.1671	90	0.06667	278	0.1996	211	0.109	51	0.05882	160	0.125	32	0.4844	3	0.1667	29	0.4483	50	0.1	20	0.025	30	0.15	75	0.2267	16	0.125	59	0.2542
rs7630177	3	C	A	367	0.1621	90	0.06667	277	0.1931	210	0.1071	51	0.05882	159	0.1226	32	0.4531	3	0.1667	29	0.4828	50	0.1	20	0.025	30	0.15	75	0.2333	16	0.125	59	0.2627
rs377109159	3	T	C	369	0.1165	91	0.2088	278	0.08633	212	0.1085	52	0.1827	160	0.08438	32	0.07812	3	0.1667	29	0.06897	50	0.14	20	0.225	30	0.08333	75	0.14	16	0.2812	59	0.1017
rs796814369	3	C	G	369	0.1179	91	0.2088	278	0.08813	212	0.1108	52	0.1827	160	0.0875	32	0.07812	3	0.1667	29	0.06897	50	0.14	20	0.225	30	0.08333	75	0.14	16	0.2812	59	0.1017
rs536855860	3	A	G	369	0.145	91	0.2253	278	0.1187	212	0.1415	52	0.2115	160	0.1188	32	0.09375	3	0.1667	29	0.08621	50	0.18	20	0.25	30	0.1333	75	0.1533	16	0.25	59	0.1271
rs28801727	4	C	T	367	0.2439	91	0.3242	276	0.2174	212	0.2123	52	0.25	160	0.2	32	0.1875	3	0.5	29	0.1552	50	0.26	20	0.375	30	0.1833	73	0.3493	16	0.4688	57	0.3158
rs28881884	4	T	C	367	0.2452	91	0.3242	276	0.2192	211	0.2133	52	0.25	159	0.2013	32	0.1875	3	0.5	29	0.1552	50	0.26	20	0.375	30	0.1833	74	0.3514	16	0.4688	58	0.319
rs28818830	4	T	C	368	0.2446	91	0.3242	277	0.2184	212	0.2123	52	0.25	160	0.2	32	0.1875	3	0.5	29	0.1552	50	0.26	20	0.375	30	0.1833	74	0.3514	16	0.4688	58	0.319
rs28868615	4	G	A	368	0.2446	91	0.3242	277	0.2184	212	0.2123	52	0.25	160	0.2	32	0.1875	3	0.5	29	0.1552	50	0.26	20	0.375	30	0.1833	74	0.3514	16	0.4688	58	0.319
rs28770574	4	A	T	367	0.2452	90	0.3278	277	0.2184	211	0.2133	51	0.2549	160	0.2	32	0.1875	3	0.5	29	0.1552	50	0.26	20	0.375	30	0.1833	74	0.3514	16	0.4688	58	0.319
rs28782495	4	G	A	369	0.2439	91	0.3242	278	0.2176	212	0.2123	52	0.25	160	0.2	32	0.1875	3	0.5	29	0.1552	50	0.26	20	0.375	30	0.1833	75	0.3467	16	0.4688	59	0.3136
rs28750399	4	A	G	368	0.2446	91	0.3242	277	0.2184	211	0.2133	52	0.25	159	0.2013	32	0.1875	3	0.5	29	0.1552	50	0.26	20	0.375	30	0.1833	75	0.3467	16	0.4688	59	0.3136
rs28826394	4	G	A	368	0.2446	91	0.3242	277	0.2184	212	0.2123	52	0.25	160	0.2	32	0.1875	3	0.5	29	0.1552	50	0.26	20	0.375	30	0.1833	74	0.3514	16	0.4688	58	0.319
rs56895716	4	TG	T	369	0.2439	92	0.3207	277	0.2184	212	0.2123	53	0.2453	159	0.2013	32	0.1875	3	0.5	29	0.1552	50	0.26	20	0.375	30	0.1833	75	0.3467	16	0.4688	59	0.3136
rs28889392	4	C	G	370	0.2432	92	0.3207	278	0.2176	213	0.2113	53	0.2453	160	0.2	32	0.1875	3	0.5	29	0.1552	50	0.26	20	0.375	30	0.1833	75	0.3467	16	0.4688	59	0.3136

rs28827921	4	T	C	369	0.2439	91	0.3242	278	0.2176	212	0.2123	52	0.25	160	0.2	32	0.1875	3	0.5	29	0.1552	50	0.26	20	0.375	30	0.1833	75	0.3467	16	0.4688	59	0.3136
rs4277751	4	T	G	368	0.2446	91	0.3242	277	0.2184	212	0.2123	52	0.25	160	0.2	32	0.1875	3	0.5	29	0.1552	50	0.26	20	0.375	30	0.1833	74	0.3514	16	0.4688	58	0.319
rs138639165	4	T	C	363	0.241	89	0.3258	274	0.2135	209	0.2105	50	0.25	159	0.1981	32	0.1875	3	0.5	29	0.1552	49	0.2551	20	0.375	29	0.1724	73	0.3425	16	0.4688	57	0.307
rs28840110	4	A	G	367	0.2425	91	0.3242	276	0.2156	212	0.2123	52	0.25	160	0.2	32	0.1875	3	0.5	29	0.1552	50	0.26	20	0.375	30	0.1833	73	0.3425	16	0.4688	57	0.307
rs60421347	4	G	GC	369	0.2425	92	0.3207	277	0.2166	212	0.2099	53	0.2453	159	0.1981	32	0.1875	3	0.5	29	0.1552	50	0.26	20	0.375	30	0.1833	75	0.3467	16	0.4688	59	0.3136
rs4389630	4	A	G	370	0.2432	92	0.3207	278	0.2176	213	0.2113	53	0.2453	160	0.2	32	0.1875	3	0.5	29	0.1552	50	0.26	20	0.375	30	0.1833	75	0.3467	16	0.4688	59	0.3136
rs1531319	4	T	C	368	0.3397	92	0.2446	276	0.3714	213	0.446	53	0.3113	160	0.4906	32	0.1875	3	0.1667	29	0.1897	50	0.31	20	0.2	30	0.3833	73	0.1164	16	0.09375	57	0.1228
rs62330192	4	A	G	369	0.2127	91	0.0989	278	0.25	212	0.2241	52	0.1154	160	0.2594	32	0.125	3	0	29	0.1379	50	0.21	20	0.1	30	0.2833	75	0.22	16	0.0625	59	0.2627
rs1207442361	4	C	T	369	0.21	92	0.1196	277	0.2401	212	0.2193	53	0.1321	159	0.2484	32	0.09375	3	0	29	0.1034	50	0.22	20	0.15	30	0.2667	75	0.2267	16	0.0625	59	0.2712
rs1243908229	4	T	A	368	0.2079	91	0.1209	277	0.2365	211	0.218	52	0.1346	159	0.2453	32	0.07812	3	0	29	0.08621	50	0.22	20	0.15	30	0.2667	75	0.2267	16	0.0625	59	0.2712
rs1010428108	4	G	A	367	0.1948	91	0.1264	276	0.2174	211	0.2038	52	0.1538	159	0.2201	32	0.1094	3	0	29	0.1207	50	0.18	20	0.125	30	0.2167	74	0.2162	16	0.0625	58	0.2586
rs2923344	4	G	A	370	0.2514	92	0.1848	278	0.2734	213	0.2535	53	0.1981	160	0.2719	32	0.2188	3	0	29	0.2414	50	0.23	20	0.225	30	0.2333	75	0.2733	16	0.125	59	0.3136
rs1579725222	4	A	C	349	0.1891	87	0.1322	262	0.208	200	0.1925	51	0.1569	149	0.2047	30	0.1167	3	0	27	0.1296	48	0.1667	18	0.1389	30	0.1833	71	0.2254	15	0.06667	56	0.2679
rs41265804	6	G	C	369	0.2236	91	0.3297	278	0.1888	212	0.217	52	0.3462	160	0.175	32	0.25	3	0.3333	29	0.2414	50	0.22	20	0.225	30	0.2167	75	0.2333	16	0.4062	59	0.1864
rs114644621	6	G	A	369	0.05149	91	0.1154	278	0.03058	212	0.07075	52	0.1538	160	0.04375	32	0.01562	3	0	29	0.01724	50	0.03	20	0.075	30	0	75	0.02667	16	0.0625	59	0.01695
rs1524738	6	G	T	369	0.4201	91	0.4945	278	0.3957	212	0.4458	52	0.4615	160	0.4156	32	0.4062	3	0.3333	29	0.3793	50	0.28	20	0.275	30	0.2833	75	0.4467	16	0.4062	59	0.4068
rs11155800	6	G	A	370	0.2068	92	0.1141	278	0.2374	213	0.2723	53	0.1321	160	0.3188	32	0.2031	3	0.1667	29	0.2069	50	0.11	20	0.075	30	0.1333	75	0.08667	16	0.09375	59	0.08475
rs6914432	6	G	C	368	0.2038	91	0.1154	277	0.2329	211	0.2654	52	0.1346	159	0.3082	32	0.2188	3	0.1667	29	0.2241	50	0.11	20	0.075	30	0.1333	75	0.08667	16	0.09375	59	0.08475
rs56681564	6	A	G	364	0.2033	89	0.1236	275	0.2291	208	0.2668	50	0.15	158	0.3038	32	0.2031	3	0.1667	29	0.2069	50	0.11	20	0.075	30	0.1333	74	0.08784	16	0.09375	58	0.08621
rs6904261	6	A	G	370	0.2041	92	0.125	278	0.2302	213	0.2676	53	0.1509	160	0.3062	32	0.2031	3	0.1667	29	0.2069	50	0.11	20	0.075	30	0.1333	75	0.08667	16	0.09375	59	0.08475
rs62434386	6	A	G	369	0.4377	92	0.3696	277	0.4603	213	0.4437	53	0.3774	160	0.4656	32	0.4688	3	0.5	29	0.4655	49	0.3878	20	0.35	29	0.4138	75	0.44	16	0.3438	59	0.4661
rs199719022	7	A	G	368	0.07473	90	0.03333	278	0.08813	211	0.04739	51	0.01961	160	0.05625	32	0.04688	3	0	29	0.05172	50	0.06	20	0.025	30	0.08333	75	0.1733	16	0.09375	59	0.1949
rs77925841	7	T	A	370	0.2216	92	0.1196	278	0.2554	213	0.223	53	0.1509	160	0.2469	32	0.2188	3	0	29	0.2414	50	0.2	20	0.1	30	0.2667	75	0.2333	16	0.0625	59	0.2797
rs74600664	7	A	T	369	0.1396	91	0.04945	278	0.1691	212	0.1321	52	0.05769	160	0.1562	32	0.1406	3	0	29	0.1552	50	0.16	20	0.05	30	0.2333	75	0.1467	16	0.03125	59	0.178
rs1231228837	7	G	A	349	0.2049	82	0.09146	267	0.2397	199	0.196	47	0.08511	152	0.2303	31	0.1774	2	0	29	0.1897	48	0.1458	19	0.02632	29	0.2241	71	0.2817	14	0.2143	57	0.2982
rs1444241839	7	T	C	348	0.2098	83	0.09639	265	0.2453	199	0.201	47	0.08511	152	0.2368	30	0.2	2	0	28	0.2143	49	0.1531	20	0.05	29	0.2241	70	0.2786	14	0.2143	56	0.2946
rs1327573902	7	G	A	347	0.2133	82	0.09146	265	0.2509	199	0.2035	47	0.08511	152	0.2401	30	0.2	2	0	28	0.2143	48	0.1458	19	0.02632	29	0.2241	70	0.2929	14	0.2143	56	0.3125
rs1441248959	7	C	A	347	0.2176	81	0.1049	266	0.2519	201	0.209	47	0.08511	154	0.2468	30	0.1833	2	0	28	0.1964	47	0.1702	18	0.08333	29	0.2241	69	0.2899	14	0.2143	55	0.3091
rs1396220338	7	T	C	348	0.217	81	0.1049	267	0.2509	201	0.2065	47	0.08511	154	0.2435	30	0.1667	2	0	28	0.1786	47	0.1702	18	0.08333	29	0.2241	70	0.3	14	0.2143	56	0.3214
rs1454766342	7	A	G	368	0.1399	91	0.2198	277	0.1137	212	0.1486	52	0.2308	160	0.1219	32	0.125	3	0.1667	29	0.1207	50	0.13	20	0.2	30	0.08333	74	0.1284	16	0.2188	58	0.1034
rs372186635	7	A	G	369	0.2507	91	0.3462	278	0.2194	212	0.2689	52	0.3269	160	0.25	32	0.1875	3	0.3333	29	0.1724	50	0.29	20	0.425	30	0.2	75	0.2	16	0.3125	59	0.1695
rs796840421	7	T	C	366	0.3989	90	0.3389	276	0.4185	212	0.4057	52	0.3558	160	0.4219	32	0.4062	3	0.3333	29	0.4138	49	0.3469	19	0.2895	30	0.3833	73	0.411	16	0.3438	57	0.4298
rs78598581	7	G	A	366	0.3989	90	0.3389	276	0.4185	213	0.3991	53	0.3302	160	0.4219	32	0.4219	3	0.3333	29	0.431	49	0.3673	19	0.3421	30	0.3833	72	0.4097	15	0.3667	57	0.4211
rs138547582	7	A	G	368	0.3601	92	0.2772	276	0.3877	213	0.3615	53	0.2736	160	0.3906	32	0.375	3	0	29	0.4138	50	0.3	20	0.275	30	0.3167	73	0.3904	16	0.3438	57	0.4035
rs77189369	7	A	G	369	0.3469	92	0.2609	277	0.3755	213	0.3521	53	0.2736	160	0.3781	32	0.3594	3	0	29	0.3966	50	0.24	20	0.2	30	0.2667	74	0.3986	16	0.3438	58	0.4138
rs113910059	7	A	G	357	0.3501	87	0.2644	270	0.3778	203	0.3498	50	0.25	153	0.3824	32	0.375	3	0	29	0.4138	50	0.29	20	0.275	30	0.3	72	0.3819	14	0.3571	58	0.3879
rs10280266	7	T	A	368	0.07201	92	0.1413	276	0.04891	213	0.07981	53	0.1792	160	0.04688	32	0.1094	3	0	29	0.1207	49	0.06122	20	0.1	29	0.03448	74	0.04054	16	0.09375	58	0.02586
rs10247514	7	T	C	362	0.07182	91	0.1374	271	0.04982	211	0.0782	52	0.1731	159	0.04717	30	0.1167	3	0	27	0.1296	49	0.06122	20	0.1	29	0.03448	72	0.04167	16	0.09375	56	0.02679
rs10264538	7	G	C	368	0.06386	91	0.1319	277	0.04152	212	0.07783	52	0.1731	160	0.04688	32	0.09375	3	0	29	0.1034	50	0.05	20	0.1	30	0.01667	74	0.02027	16	0.0625	58	0.008621
rs10276547	7	T	C	367	0.07221	91	0.1429	276	0.04891	212	0.08019	52	0.1827	160	0.04688	32	0.1094	3	0	29	0.1207	50	0.06	20	0.1	30	0.03333	73	0.0411	16	0.09375	57	0.02632
rs10276241	7	T	C	367	0.07084	91	0.1374	276	0.04891	212	0.07783	52	0.1731	160	0.04688	32	0.1094	3	0	29	0.1207	49	0.06122	20	0.1	29	0.03448	74	0.04054	16	0.09375	58	0.02586
rs10236154	7	A	G	366	0.07104	92	0.1359	274	0.04927	213	0.07746	53	0.1698	160	0.04688	31	0.1129	3	0	28	0.125	49	0.06122	20	0.1	29	0.03448	73	0.0411	16	0.09375	57	0.02632
rs79702292	7	G	A	370	0.3541	92	0.4565	278	0.3201	213	0.3685	53	0.4717	160	0.3344	32	0.3125	3	0.5	29	0.2931	50	0.37	20	0.45	30	0.3167	75	0.32	16	0.4062	59	0.2966
rs78451619	7	A	T	369	0.3388	91	0.4396	278	0.3058	212	0.3467	52	0.4423	160	0.3156	32	0.3281	3	0.5	29	0.3103	50	0.36	20	0.45	30	0.3	75	0.3067	16	0.4062	59	0.2797
rs279691	7	T	A	366	0.05601	91	0.06593	275	0.05273	210	0.05238	52	0.04808	158	0.0538	32	0.125	3	0	29	0.1379	50	0.04	20	0.05	30	0.03333	74	0.0473	16	0.1562	58	0.01724
rs279697	7	A	T	368	0.05571	91	0.06593	277	0.05235	212	0.05189	52	0.04808	160	0.05312	32	0.125	3	0	29	0.1379	50	0.04	20	0.05	30	0.03333	74	0.0473	16	0.1562	58	0.01724
rs157656	7	A	C	369	0.05556	92	0.06522	277	0.05235	213	0.05164	53	0.04717	160	0.05312	32	0.125	3	0	29	0.1379	50	0.04	20	0.05	30	0.03333	74	0.0473	16	0.1562	58	0.01724
rs34163760	7	A	G	370	0.1243	92	0.2174	278	0.09353	213	0.1221	53	0.2075	160	0.09375	32	0.1875	3	0.5	29	0.1552	50	0.11	20	0.15	30	0.08333	75	0.1133	16	0.2812	59	0.0678
rs1278498984	7	A	C	369	0.187	91	0.2747	278	0.1583	212	0.1934	52	0.2981	160	0.1594	32	0.1719	3	0.5	29	0.1379	50	0.2	20	0.2	30	0.2	75	0.1667	16	0.25	59	0.1441
rs1215719281	7	T	A	369	0.1829	91	0.2692	278	0.1547	212	0.1863	52	0.2885	160	0.1531	32	0.1719	3	0.5	29	0.1379	50	0.2	20	0.2	30	0.2	75	0.1667	16	0.25	59	0.1441
rs1276772253	7	T	C	369	0.1789	91	0.2692	278	0.1493	212	0.1863	52	0.2885	160	0.1531	32	0.1719	3	0.5	29	0.1379	50	0.18	20	0.2	30	0.1667	75	0.16	16	0.25	59	0.1356
rs1218424730	7	G	A	369	0.1775	91	0.2692	278	0.1475	212	0.1863	52	0.2885	160	0.1531	32	0.1719	3	0.5	29	0.1379	50	0.17	20	0.2	30	0.15	75	0.16	16	0.25	59	0.1356
rs189593833	7	A	C	369	0.1341	91	0.2143	278	0.1079	212	0.1509	52	0.2404	160	0.1219	32	0.1094	3	0.3333	29	0.08621	50	0.12	20	0.15	30	0.1	75	0.1067	16	0.1875	59	0.08475
rs371146758	7	G	A	369	0.1314	91	0.2143	278	0.1043	212	0.1439	52	0.2308	160	0.1156	32	0.1094	3	0.3333	29	0.08621	50	0.12	20	0.15	30	0.1	75	0.1133	16	0.2188	59	0.08475
rs6988966	8	A	G	370	0.1649	92	0.2717	278	0.1295	213	0.0892	53	0.1415	160	0.07188	32	0.1562	3	0.3333	29	0.1379	50	0.27	20	0.475	30	0.1333	75	0.3133	16	0.4375	59	0.2797
rs9643846	8	G	T	369	0.1667	91	0.2802	278	0.1295	212	0.08962	52	0.1442	160	0.07188	32	0.1719	3	0.5	29	0.1379	50	0.27	20	0.475	30	0.1333	75	0.3133	16	0.4375	59	0.2797
rs9643847	8	A	G	369	0.1667	92	0.2772	277	0.13	213	0.09155	53	0.1509	160	0.07188	32	0.1562	3	0.3333	29	0.1379	50	0.27	20	0.475	30	0.1333	74	0.3176	16	0.4375	58	0.2845
rs57504215	8	A	G	368	0.1658	91	0.2747	277	0.13	212	0.08962	52	0.1442	160	0.07188	32	0.1562	3	0.3333	29	0.1379	50	0.27	20	0.475	30	0.1333	74	0.3176	16	0.4375	58	0.2845
rs4332118	8	T	C	367	0.1567	91	0.2637	276	0.1214	212	0.08962	52	0.1442	160	0.07188	32	0.09375	3	0.1667	29	0.08621	50	0.26	20	0.45	30	0.1333	73	0.3082	16	0.4375	57	0.2719
rs61320655	8	G	T	368	0.1562	91	0.2637	277	0.1209	212	0.08962	52	0.1442	160	0.07188	32	0.07812	3	0.1667	29	0.06897	50	0.26	20	0.45	30	0.1333	74	0.3108	16	0.4375	58	0.2759
rs12541699	8	T	A	369	0.1558	91	0.2637	278	0.1205	212	0.08962	52	0.1442	160	0.07188	32	0.07812	3	0.1667	29	0.06897	50	0.26	20	0.45	30	0.1333	75	0.3067	16	0.4375	59	0.2712
rs60545202	8	G	A	369	0.1572	92	0.2663	277	0.1209	213	0.09155	53	0.1509	160	0.07188	32	0.07812	3	0.1667	29	0.06897	50	0.26	20	0.45	30	0.1333	74	0.3108	16	0.4375	58	0.2759
rs62497418	8	C	T	369	0.1585	91	0.2637	278	0.1241	212	0.08962	52	0.1442	160	0.07188	32	0.1094	3	0.1667	29	0.1034	50	0.26	20	0.45	30	0.1333	75	0.3067	16	0.4375	59	0.2712
rs12677604	8	A	G	364	0.1593	90	0.2667	274	0.1241	208	0.09135	51	0.1471	157	0.07325	32	0.1094	3	0.1667	29	0.1034	50	0.26	20	0.45	30	0.1333	74	0.3041	16	0.4375	58	0.2672
rs12677682	8	A	G	369	0.1545	91	0.2582	278	0.1205	212	0.08962	52	0.1442	160	0.07188	32	0.09375	3	0.1667	29	0.08621	50	0.26	20	0.45	30	0.1333	75	0.2933	16	0.4062	59	0.2627
rs60311345	8	A	G	369	0.1572	91	0.2637	278	0.1223	212	0.08962	52	0.1442	160	0.07188	32	0.1094	3	0.1667	29	0.1034	50	0.26	20	0.45	30	0.1333	75	0.3	16	0.4375	59	0.2627
rs56847872	8	G	T	368	0.159	91	0.2637	277	0.1245	212	0.08962	52	0.1442	160	0.07188	32	0.125	3	0.1667	29	0.1207	50	0.26	20	0.45	30	0.1333	74	0.3041	16	0.4375	58	0.2672

rs60297253	8	A	G	363	0.1598	90	0.2611	273	0.1264	210	0.09286	52	0.1442	158	0.07595	31	0.1129	3	0.1667	28	0.1071	50	0.26	20	0.45	30	0.1333	72	0.3056	15	0.4333	57	0.2719
rs7832204	8	T	C	369	0.1585	91	0.2637	278	0.1241	212	0.08962	52	0.1442	160	0.07188	32	0.125	3	0.1667	29	0.1207	50	0.26	20	0.45	30	0.1333	75	0.3	16	0.4375	59	0.2627
rs7831620	8	A	G	369	0.1599	91	0.2637	278	0.1259	212	0.09198	52	0.1442	160	0.075	32	0.125	3	0.1667	29	0.1207	50	0.26	20	0.45	30	0.1333	75	0.3	16	0.4375	59	0.2627
rs12679157	8	A	G	369	0.1585	91	0.2637	278	0.1241	212	0.08962	52	0.1442	160	0.07188	32	0.125	3	0.1667	29	0.1207	50	0.26	20	0.45	30	0.1333	75	0.3	16	0.4375	59	0.2627
rs6995809	8	G	C	368	0.159	91	0.2637	277	0.1245	211	0.09005	52	0.1442	159	0.07233	32	0.125	3	0.1667	29	0.1207	50	0.26	20	0.45	30	0.1333	75	0.3	16	0.4375	59	0.2627
rs6995810	8	A	C	368	0.159	91	0.2637	277	0.1245	211	0.09005	52	0.1442	159	0.07233	32	0.125	3	0.1667	29	0.1207	50	0.26	20	0.45	30	0.1333	75	0.3	16	0.4375	59	0.2627
rs12676292	8	T	C	370	0.1595	92	0.2663	278	0.1241	213	0.0939	53	0.1509	160	0.075	32	0.1094	3	0.1667	29	0.1034	50	0.26	20	0.45	30	0.1333	75	0.3	16	0.4375	59	0.2627
rs58008678	8	T	C	368	0.1576	91	0.2637	277	0.1227	212	0.08962	52	0.1442	160	0.07188	31	0.1129	3	0.1667	28	0.1071	50	0.26	20	0.45	30	0.1333	75	0.3	16	0.4375	59	0.2627
rs59556769	8	A	G	369	0.1612	91	0.2692	278	0.1259	212	0.08962	52	0.1442	160	0.07188	32	0.1562	3	0.3333	29	0.1379	50	0.26	20	0.45	30	0.1333	75	0.3	16	0.4375	59	0.2627
rs12678434	8	T	C	368	0.159	91	0.2637	277	0.1245	212	0.08726	52	0.1346	160	0.07188	32	0.1562	3	0.3333	29	0.1379	49	0.2653	20	0.45	29	0.1379	75	0.2933	16	0.4375	59	0.2542
rs6988685	8	T	C	369	0.1585	91	0.2637	278	0.1241	212	0.08726	52	0.1346	160	0.07188	32	0.1562	3	0.3333	29	0.1379	50	0.26	20	0.45	30	0.1333	75	0.2933	16	0.4375	59	0.2542
rs4341150	8	C	A	369	0.1599	91	0.2637	278	0.1259	212	0.08962	52	0.1346	160	0.075	32	0.1562	3	0.3333	29	0.1379	50	0.26	20	0.45	30	0.1333	75	0.2933	16	0.4375	59	0.2542
rs73677451	8	T	G	370	0.1595	92	0.2663	278	0.1241	213	0.0892	53	0.1415	160	0.07188	32	0.1562	3	0.3333	29	0.1379	50	0.26	20	0.45	30	0.1333	75	0.2933	16	0.4375	59	0.2542
rs12549678	8	A	G	368	0.159	91	0.2637	277	0.1245	212	0.08726	52	0.1346	160	0.07188	32	0.1562	3	0.3333	29	0.1379	50	0.26	20	0.45	30	0.1333	74	0.2973	16	0.4375	58	0.2586
rs9642793	8	A	G	369	0.1504	91	0.1154	278	0.1619	212	0.1321	52	0.06731	160	0.1531	32	0.1562	3	0	29	0.1724	50	0.31	20	0.3	30	0.3167	75	0.09333	16	0.0625	59	0.1017
rs62523332	8	A	G	368	0.1413	91	0.1099	277	0.1516	211	0.1232	52	0.06731	159	0.1415	32	0.1094	3	0	29	0.1207	50	0.3	20	0.275	30	0.3167	75	0.1	16	0.0625	59	0.1102
rs193014517	9	A	G	362	0.05249	89	0.1292	273	0.02747	207	0.01691	50	0.04	157	0.009554	31	0.1452	3	0.3333	28	0.125	50	0.14	20	0.3	30	0.03333	74	0.05405	16	0.1562	58	0.02586
rs600075	9	A	G	368	0.1685	91	0.2692	277	0.1354	212	0.1203	52	0.1923	160	0.09688	32	0.2969	3	0.3333	29	0.2931	50	0.23	20	0.375	30	0.1333	74	0.2095	16	0.375	58	0.1638
rs647328	9	A	G	370	0.1676	92	0.2663	278	0.1349	213	0.1174	53	0.1887	160	0.09375	32	0.2969	3	0.3333	29	0.2931	50	0.23	20	0.375	30	0.1333	75	0.2133	16	0.375	59	0.1695
rs5896385	9	CA	C	367	0.1676	91	0.2692	276	0.1341	212	0.1179	52	0.1923	160	0.09375	32	0.2969	3	0.3333	29	0.2931	49	0.2347	20	0.375	29	0.1379	74	0.2095	16	0.375	58	0.1638
rs35433273	9	C	CA	369	0.06775	92	0.1413	277	0.04332	213	0.01878	53	0.03774	160	0.0125	32	0.2656	3	0.3333	29	0.2241	50	0.17	20	0.325	30	0.06667	74	0.05405	16	0.1562	58	0.02586
rs12003726	9	G	A	368	0.05707	92	0.1304	276	0.03261	213	0.01643	53	0.03774	160	0.009375	31	0.1935	3	0.3333	28	0.1786	49	0.1531	20	0.325	29	0.03448	75	0.05333	16	0.1562	59	0.02542
rs1951785	9	C	T	367	0.05586	91	0.1264	276	0.03261	211	0.01659	52	0.03846	159	0.009434	32	0.2031	3	0.3333	29	0.1897	50	0.14	20	0.325	30	0.01667	74	0.0473	16	0.125	58	0.02586
rs1590347	9	T	C	364	0.1731	91	0.2747	273	0.1392	209	0.1292	52	0.2115	157	0.1019	32	0.3281	3	0.5	29	0.3103	50	0.24	20	0.375	30	0.15	73	0.1849	16	0.3125	57	0.1491
rs1463676990	9	A	T	369	0.1762	92	0.2826	277	0.1408	213	0.1854	53	0.2736	160	0.1562	32	0.1094	3	0.3333	29	0.08621	50	0.18	20	0.225	30	0.15	74	0.1757	16	0.375	58	0.1207
rs78491863	9	T	G	369	0.2805	91	0.3901	278	0.2446	212	0.2736	52	0.3846	160	0.2375	32	0.2031	3	0.5	29	0.1724	50	0.38	20	0.4	30	0.3667	75	0.2667	16	0.375	59	0.2373
rs62545432	9	A	T	370	0.3676	92	0.4511	278	0.3399	213	0.3732	53	0.4434	160	0.35	32	0.3125	3	0.5	29	0.2931	50	0.37	20	0.475	30	0.3	75	0.3733	16	0.4375	59	0.3559
rs1375091269	9	C	A	369	0.08266	91	0.02198	278	0.1025	212	0.08726	52	0.02885	160	0.1062	32	0.09375	3	0	29	0.1034	50	0.06	20	0	30	0.1	75	0.08	16	0.03125	59	0.09322
rs375381055	9	T	A	370	0.1284	92	0.07065	278	0.1475	213	0.1338	53	0.07547	160	0.1531	32	0.2188	3	0	29	0.2414	50	0.12	20	0.075	30	0.15	75	0.08	16	0.0625	59	0.08475
rs1464935173	9	G	A	370	0.1176	92	0.05978	278	0.1367	213	0.1197	53	0.0566	160	0.1406	32	0.1875	3	0	29	0.2069	50	0.12	20	0.075	30	0.15	75	0.08	16	0.0625	59	0.08475
rs1396392235	9	C	G	368	0.0856	91	0.03297	277	0.1029	211	0.08768	52	0.02885	159	0.1069	32	0.125	3	0	29	0.1379	50	0.1	20	0.05	30	0.1333	75	0.05333	16	0.03125	59	0.05932
rs7907859	10	G	A	370	0.4743	92	0.3641	278	0.4892	213	0.4695	53	0.434	160	0.4375	32	0.07812	3	0.1667	29	0.06897	50	0.44	20	0.325	30	0.4833	75	0.4933	16	0.2188	59	0.4153
rs7921490	10	C	T	366	0.0724	91	0.1429	275	0.04909	212	0.08019	52	0.1538	160	0.05625	31	0.04839	3	0	28	0.05357	50	0.12	20	0.2	30	0.06667	73	0.0274	16	0.0625	57	0.01754
rs375244917	10	T	G	369	0.2466	91	0.3571	278	0.2104	212	0.2217	52	0.3077	160	0.1938	32	0.1562	3	0.3333	29	0.1379	50	0.33	20	0.425	30	0.2667	75	0.3	16	0.4375	59	0.2627
rs1350106841	10	A	G	369	0.09621	91	0.02198	278	0.1205	212	0.0967	52	0.03846	160	0.1156	32	0.1094	3	0	29	0.1207	50	0.08	20	0	30	0.1333	75	0.1	16	0	59	0.1271
rs200056850	10	C	G	362	0.2735	86	0.186	276	0.3007	209	0.256	49	0.1837	160	0.2781	32	0.3125	3	0	29	0.3448	49	0.2347	19	0.1842	30	0.2667	72	0.3333	15	0.2333	57	0.3596
rs4751880	10	C	A	370	0.3486	92	0.4239	278	0.3237	213	0.2864	53	0.3868	160	0.2531	32	0.375	3	0.3333	29	0.3793	50	0.49	20	0.425	30	0.4667	75	0.4067	16	0.375	59	0.4153
rs36228357	11	C	CCACTCCT	369	0.1274	92	0.212	277	0.09928	213	0.08685	53	0.1509	160	0.06562	31	0.1935	3	0.5	28	0.1607	50	0.25	20	0.3	30	0.2167	75	0.1333	16	0.25	59	0.1017
rs1403582266	11	C	A	369	0.1721	92	0.1141	277	0.1913	213	0.1714	53	0.1038	160	0.1938	32	0.125	3	0	29	0.1379	50	0.16	20	0.15	30	0.1667	74	0.2027	16	0.125	58	0.2241
rs192400155	11	A	G	364	0.1016	89	0.1798	275	0.07636	210	0.09524	50	0.19	160	0.06562	31	0.06452	3	0.1667	28	0.05357	49	0.08163	20	0.15	29	0.03448	74	0.1486	16	0.1875	58	0.1379
rs28730284	12	G	A	348	0.2198	90	0.08333	258	0.2674	200	0.2225	51	0.1078	149	0.2617	30	0.3	3	0	27	0.3333	49	0.1224	20	0.025	29	0.1897	69	0.2464	16	0.09375	53	0.2925
rs7306104	12	A	G	370	0.3365	92	0.2283	278	0.3723	213	0.4225	53	0.2925	160	0.4656	32	0.1562	3	0.1667	29	0.1552	50	0.28	20	0.15	30	0.3667	75	0.2067	16	0.125	59	0.2288
rs1259164930	13	T	C	369	0.4417	91	0.3901	278	0.4586	212	0.4434	52	0.4135	160	0.4531	32	0.4219	3	0.1667	29	0.4483	50	0.43	20	0.35	30	0.4833	75	0.4533	16	0.4062	59	0.4661
rs2801763	13	G	T	369	0.4268	91	0.3626	278	0.4478	212	0.4292	52	0.3942	160	0.4406	32	0.4219	3	0.1667	29	0.4483	50	0.42	20	0.325	30	0.4833	75	0.4267	16	0.3438	59	0.4492
rs1202400626	13	C	T	369	0.4187	91	0.3462	278	0.4424	212	0.4222	52	0.3846	160	0.4344	32	0.4219	3	0.1667	29	0.4483	50	0.4	20	0.3	30	0.4667	75	0.42	16	0.3125	59	0.4492
rs1240713470	13	T	G	369	0.416	91	0.3407	278	0.4406	212	0.4198	52	0.375	160	0.4344	32	0.4062	3	0.1667	29	0.431	50	0.4	20	0.3	30	0.4667	75	0.42	16	0.3125	59	0.4492
rs11148117	13	A	T	353	0.3754	85	0.2529	268	0.4142	204	0.4363	48	0.3229	156	0.4712	31	0.2097	3	0.1667	28	0.2143	48	0.3646	19	0.1842	29	0.4828	70	0.2786	15	0.1333	55	0.3182
rs1199895	13	T	G	370	0.4108	92	0.2935	278	0.4496	213	0.4695	53	0.3585	160	0.4938	32	0.3594	3	0.1667	29	0.3793	50	0.4	20	0.25	30	0.5	75	0.2733	16	0.1562	59	0.3051
rs9552432	13	G	A	369	0.3238	91	0.4505	278	0.2824	212	0.1863	52	0.2885	160	0.1531	32	0.3281	3	0.1667	29	0.2759	50	0.39	20	0.425	30	0.2667	75	0.3333	16	0.25	59	0.3559
rs2874196	13	T	C	369	0.374	91	0.2582	278	0.4119	212	0.4363	52	0.3173	160	0.475	32	0.2031	3	0.1667	29	0.2069	50	0.37	20	0.2	30	0.4833	75	0.2733	16	0.1562	59	0.3051
rs34946673	13	T	C	369	0.393	91	0.2692	278	0.4335	212	0.4505	52	0.3173	160	0.4938	32	0.25	3	0.1667	29	0.2586	50	0.39	20	0.2	30	0.4833	75	0.2933	16	0.2188	59	0.3136
rs11148128	13	T	A	369	0.3957	91	0.2802	278	0.4335	213	0.4507	53	0.3302	160	0.4906	32	0.2188	3	0.1667	29	0.2241	49	0.4082	19	0.2105	30	0.4667	75	0.3067	16	0.2188	59	0.3305
rs2025243	13	G	A	369	0.3902	91	0.2692	278	0.4299	212	0.4528	52	0.3173	160	0.4969	32	0.2031	3	0.1667	29	0.2069	50	0.39	20	0.2	30	0.4833	75	0.2933	16	0.2188	59	0.3136
rs111475357	13	C	CCAA	367	0.4687	91	0.3901	276	0.4221	211	0.3886	52	0.4808	159	0.3459	32	0.4688	3	0.1667	29	0.431	49	0.4796	20	0.3	29	0.3966	75	0.34	16	0.25	59	0.3644
rs2315545	13	T	C	368	0.3886	91	0.2692	277	0.4278	212	0.4458	52	0.3173	160	0.4875	32	0.2031	3	0.1667	29	0.2069	49	0.3878	20	0.2	29	0.4828	75	0.3067	16	0.2188	59	0.3305
rs9550741	13	A	G	370	0.2932	92	0.413	278	0.2536	213	0.1526	53	0.2642	160	0.1156	32	0.2656	3	0.3333	29	0.2241	50	0.39	20	0.45	30	0.2833	75	0.36	16	0.3125	59	0.3729
rs9634371	13	A	G	369	0.294	91	0.4176	278	0.2536	212	0.1533	52	0.2692	160	0.1156	32	0.2656	3	0.3333	29	0.2241	50	0.39	20	0.45	30	0.2833	75	0.36	16	0.3125	59	0.3729
rs718426	13	G	A	368	0.3967	90	0.2778	278	0.4353	212	0.4575	52	0.3173	160	0.4969	32	0.2031	3	0.1667	29	0.2069	49	0.4082	19	0.2368	30	0.4833	75	0.3	16	0.2188	59	0.322
rs1044090	13	C	A	368	0.3967	92	0.2826	276	0.4348	213	0.4554	53	0.3302	160	0.4969	31	0.2258	3	0.1667	28	0.2321	49	0.398	20	0.225	29	0.4828	75	0.3	16	0.2188	59	0.322
rs9552442	13	G	A	368	0.3003	91	0.4176	277	0.2617	212	0.1627	52	0.2692	160	0.1281	32	0.2656	3	0.3333	29	0.2241	49	0.398	20	0.45	29	0.2931	75	0.36	16	0.3125	59	0.3729
rs12861578	13	G	T	369	0.3984	91	0.2802	278	0.4371	212	0.4599	52	0.3269	160	0.4969	32	0.2188	3	0.1667	29	0.2241	50	0.4	20	0.225	30	0.4833	75	0.3	16	0.2188	59	0.322
rs1359190	13	G	T	370	0.4	92	0.288	278	0.4371	213	0.4624	53	0.3396	160	0.4969	32	0.2188	3	0.1667	29	0.2241	50	0.4	20	0.225	30	0.4833	75	0.3	16	0.2188	59	0.322
rs60181111	13	G	A	369	0.3984	91	0.2747	278	0.4388	212	0.4623	52	0.3269	160	0.4938	32	0.2188	3	0.1667	29	0.2241	50	0.39	20	0.2	30	0.4833	75	0.3	16	0.2188	59	0.322
rs10492455	13	G	A	369	0.3984	91	0.2747	278	0.4388	212	0.4623	52	0.3269	160	0.4938	32	0.2188	3	0.1667	29	0.2241	50	0.39	20	0.2	30	0.4833	75	0.3	16	0.2188	59	0.322
rs12869776	13	G	A	366	0.4016	91	0.2747	275	0.4436	211	0.4645	52	0.3269	159	0.4906	31	0.2258	3	0.1667	28	0.2321	50	0.39	20	0.2	30	0.4833	74	0.3041	16	0.2188	58	0.3276
rs2181751	13	C	T	368	0.3859	91	0.467	277	0.3375	212	0.2877	52	0.4423	160	0.2375	31	0.3226	3	0.1667	28	0.2679	50	0.41	20	0.45	30	0.3167	75	0.3267	16	0.25	59	0.3475
rs12585631	13	A	T	369	0.4106	91	0.2747	278	0.455	212	0.4741	52	0.3269	160	0.4781	32	0.25	3	0.1667	29	0.2586	50	0.39	20	0.2	30	0.4833	75	0.3133	16	0.2188	59	0.339

rs2798266	13	C	T	369	0.4106	91	0.2802	278	0.4532	212	0.4646	52	0.3269	160	0.4906	32	0.3125	3	0.1667	29	0.3276	50	0.39	20	0.2	30	0.4833	75	0.3133	16	0.25	59	0.3305
rs72183497	13	A	AGCATCAT	367	0.4183	90	0.2889	277	0.4603	211	0.4645	51	0.3333	160	0.4938	32	0.375	3	0.1667	29	0.3966	50	0.4	20	0.225	30	0.4833	74	0.3176	16	0.25	58	0.3362
rs4769167	13	C	T	369	0.4106	91	0.2912	278	0.4496	212	0.467	52	0.3365	160	0.4906	32	0.25	3	0.1667	29	0.2586	50	0.39	20	0.225	30	0.5	75	0.3333	16	0.25	59	0.3559
rs7983473	13	A	G	368	0.2976	91	0.4176	277	0.2581	211	0.1611	52	0.2692	159	0.1258	32	0.2656	3	0.3333	29	0.2241	50	0.39	20	0.45	30	0.2833	75	0.3667	16	0.3125	59	0.3814
rs9552451	13	T	C	369	0.3008	92	0.4185	277	0.2617	213	0.1643	53	0.2642	160	0.1312	31	0.2742	3	0.3333	28	0.2321	50	0.4	20	0.425	30	0.2833	75	0.3667	16	0.3125	59	0.3814
rs147058896	13	A	ATATCCAACTGCACTAGTACCAT	367	0.4033	91	0.2747	276	0.4457	211	0.455	52	0.3269	159	0.4969	32	0.3125	3	0.1667	29	0.3276	49	0.3776	20	0.175	29	0.4828	75	0.3133	16	0.25	59	0.3305
rs9552453	13	G	A	369	0.3008	91	0.4231	278	0.2608	212	0.1651	52	0.2692	160	0.1312	32	0.2656	3	0.3333	29	0.2241	50	0.4	20	0.425	30	0.2833	75	0.3667	16	0.3125	59	0.3814
rs68090535	13	T	TAC	370	0.2973	92	0.413	278	0.259	213	0.1643	53	0.2642	160	0.1312	32	0.2656	3	0.3333	29	0.2241	50	0.39	20	0.45	30	0.2833	75	0.3733	16	0.3125	59	0.3898
rs11148172	13	T	C	369	0.4201	91	0.2912	278	0.4622	212	0.4882	52	0.3558	160	0.4688	32	0.2656	3	0.1667	29	0.2759	50	0.4	20	0.2	30	0.4667	75	0.3067	16	0.2188	59	0.3305
rs139110039	13	A	G	369	0.2751	91	0.1868	278	0.304	212	0.2618	52	0.1731	160	0.2906	32	0.1719	3	0.1667	29	0.1724	50	0.24	20	0.15	30	0.3	75	0.38	16	0.2812	59	0.4068
rs146546354	13	A	ATG	343	0.2638	87	0.1782	256	0.293	198	0.2424	48	0.1667	150	0.2667	31	0.1774	3	0.1667	28	0.1786	46	0.2283	20	0.15	26	0.2885	68	0.3897	16	0.25	52	0.4327
rs143665261	14	A	G	369	0.05149	91	0.1154	278	0.03058	212	0.07547	52	0.1731	160	0.04375	32	0.01562	3	0	29	0.01724	50	0.03	20	0.05	30	0.01667	75	0.01333	16	0.03125	59	0.008475
rs117885021	14	A	G	367	0.05177	90	0.1167	277	0.03069	211	0.07583	51	0.1765	160	0.04375	32	0.01562	3	0	29	0.01724	49	0.03061	20	0.05	29	0.01724	75	0.01333	16	0.03125	59	0.008475
rs569699407	14	T	C	368	0.125	91	0.06044	277	0.1462	212	0.1321	52	0.07692	160	0.15	31	0.04839	3	0	28	0.05357	50	0.1	20	0	30	0.1667	75	0.1533	16	0.09375	59	0.1695
rs1321063331	14	G	A	368	0.125	91	0.06044	277	0.1462	212	0.1321	52	0.07692	160	0.15	31	0.04839	3	0	28	0.05357	50	0.1	20	0	30	0.1667	75	0.1533	16	0.09375	59	0.1695
rs76832499	14	T	C	368	0.1332	91	0.2253	277	0.1029	212	0.1557	52	0.2885	160	0.1125	32	0.0625	3	0.3333	29	0.03448	50	0.11	20	0.125	30	0.1	74	0.1149	16	0.125	58	0.1121
rs7151803	14	A	G	369	0.1355	91	0.2143	278	0.1097	212	0.1132	52	0.1827	160	0.09062	32	0.3281	3	0.1667	29	0.3448	50	0.14	20	0.25	30	0.06667	75	0.1133	16	0.2812	59	0.0678
rs1222431693	15	G	A	369	0.1911	92	0.1141	277	0.2166	213	0.1972	53	0.09434	160	0.2312	32	0.1562	3	0	29	0.1724	50	0.14	20	0.175	30	0.1167	74	0.223	16	0.125	58	0.25
rs200566609	15	C	A	363	0.2713	90	0.1722	273	0.304	208	0.2812	51	0.2059	157	0.3057	31	0.3065	3	0	28	0.3393	49	0.2347	20	0.125	29	0.3103	75	0.2533	16	0.1562	59	0.2797
rs76495562	16	C	T	368	0.1332	91	0.04396	277	0.1625	212	0.1439	52	0.05769	160	0.1719	32	0.09375	3	0	29	0.1034	49	0.08163	20	0.025	29	0.1207	75	0.1533	16	0.03125	59	0.1864
rs1434984324	16	A	C	360	0.1417	88	0.06818	272	0.1654	208	0.149	49	0.09184	159	0.1667	31	0.129	3	0	28	0.1429	50	0.08	20	0.025	30	0.1167	71	0.169	16	0.0625	55	0.2
rs1395572932	16	A	C	368	0.2935	91	0.3736	277	0.2671	212	0.3019	52	0.3654	160	0.2812	32	0.2812	3	0.3333	29	0.2759	50	0.34	20	0.4	30	0.3	74	0.2432	16	0.375	58	0.2069
rs1238425945	16	C	G	370	0.1838	92	0.2554	278	0.1601	213	0.2136	53	0.283	160	0.1906	32	0.1875	3	0.3333	29	0.1724	50	0.15	20	0.15	30	0.15	75	0.12	16	0.2812	59	0.07627
rs79263592	17	G	C	369	0.2425	91	0.3462	278	0.2086	212	0.2358	52	0.3269	160	0.2062	32	0.2188	3	0.3333	29	0.2069	50	0.27	20	0.375	30	0.2	75	0.2533	16	0.375	59	0.2203
rs62049740	17	T	C	369	0.2846	91	0.1978	278	0.3129	212	0.2594	52	0.125	160	0.3031	32	0.2188	3	0	29	0.2414	50	0.34	20	0.35	30	0.3333	75	0.3467	16	0.2812	59	0.3644
rs1422393541	17	T	C	369	0.2412	91	0.3571	278	0.2032	212	0.2311	52	0.3173	160	0.2031	32	0.2344	3	0.5	29	0.2069	50	0.29	20	0.4	30	0.2167	75	0.24	16	0.4062	59	0.1949
rs1480649071	17	A	G	369	0.1152	91	0.2143	278	0.08273	212	0.1038	52	0.2019	160	0.07188	32	0.1094	3	0.3333	29	0.08621	50	0.15	20	0.225	30	0.1	75	0.1267	16	0.2188	59	0.1017
rs1387678001	17	A	G	370	0.2905	92	0.2065	278	0.3183	213	0.2864	53	0.2264	160	0.3062	32	0.3281	3	0.1667	29	0.3448	50	0.25	20	0.15	30	0.3167	75	0.3133	16	0.2188	59	0.339
rs1157112523	17	G	A	369	0.1883	92	0.1087	277	0.2148	213	0.2042	53	0.1321	160	0.2281	32	0.2969	3	0.1667	29	0.3103	49	0.09184	20	0.05	29	0.1207	75	0.16	16	0.09375	59	0.178
rs73313398	17	C	T	366	0.1107	90	0.03889	276	0.1341	209	0.1053	51	0.04902	158	0.1234	32	0.125	3	0	29	0.1379	50	0.09	20	0.025	30	0.1333	75	0.1333	16	0.03125	59	0.161
rs1293649278	17	T	C	368	0.3451	92	0.25	276	0.3768	212	0.3255	53	0.2075	159	0.3648	32	0.4062	3	0.1667	29	0.431	50	0.35	20	0.375	30	0.3333	74	0.3716	16	0.25	58	0.4052
rs73312311	17	T	G	368	0.2704	91	0.3681	277	0.2383	212	0.2618	52	0.3654	160	0.2281	32	0.2656	3	0.3333	29	0.2586	50	0.3	20	0.375	30	0.25	74	0.277	16	0.375	58	0.25
rs76931425	17	A	G	370	0.1581	92	0.08152	278	0.1835	213	0.1362	53	0.06604	160	0.1594	32	0.1719	3	0.1667	29	0.1724	50	0.12	20	0.05	30	0.1667	75	0.24	16	0.1562	59	0.2627
rs577418	18	A	T	369	0.477	91	0.4286	278	0.446	212	0.4741	52	0.3654	160	0.4906	32	0.375	3	0.5	29	0.3621	50	0.49	20	0.475	30	0.4667	75	0.3733	16	0.4375	59	0.3559
rs493201	18	T	C	370	0.4797	92	0.4239	278	0.4478	213	0.4718	53	0.3585	160	0.4906	32	0.3906	3	0.5	29	0.3793	50	0.49	20	0.475	30	0.4667	75	0.3733	16	0.4375	59	0.3559
rs525254	18	C	T	365	0.4712	90	0.4333	275	0.44	210	0.4738	51	0.3725	159	0.4937	32	0.3438	3	0.5	29	0.3276	49	0.4898	20	0.475	29	0.4655	74	0.3581	16	0.4375	58	0.3362
rs522420	18	T	C	367	0.4632	91	0.4341	276	0.4293	211	0.4905	52	0.375	159	0.4717	32	0.3438	3	0.5	29	0.3276	50	0.5	20	0.475	30	0.4833	74	0.3581	16	0.4375	58	0.3362
rs622870	18	A	G	368	0.4715	91	0.4286	277	0.4386	212	0.4764	52	0.3654	160	0.4875	32	0.3438	3	0.5	29	0.3276	49	0.4898	20	0.475	29	0.4655	75	0.3667	16	0.4375	59	0.3475
rs642133	18	G	A	369	0.4729	91	0.4286	278	0.4406	212	0.4764	52	0.3654	160	0.4875	32	0.3438	3	0.5	29	0.3276	50	0.5	20	0.475	30	0.4833	75	0.3667	16	0.4375	59	0.3475
rs656067	18	T	C	362	0.4765	90	0.4278	272	0.4449	207	0.4686	51	0.3529	156	0.4936	31	0.3387	3	0.5	28	0.3214	50	0.49	20	0.5	30	0.4833	74	0.3716	16	0.4375	58	0.3534
rs523761	18	T	C	365	0.4712	91	0.4341	274	0.4398	209	0.4761	52	0.3654	157	0.4873	32	0.3281	3	0.5	29	0.3103	50	0.5	20	0.475	30	0.4833	74	0.3649	16	0.4062	58	0.3534
rs657024	18	G	A	366	0.4727	91	0.4286	275	0.44	210	0.4786	52	0.3654	158	0.4842	32	0.3438	3	0.5	29	0.3276	50	0.5	20	0.475	30	0.4833	74	0.3716	16	0.4375	58	0.3534
rs525137	18	C	T	369	0.4729	91	0.4286	278	0.4406	212	0.4764	52	0.3654	160	0.4875	32	0.3438	3	0.5	29	0.3276	50	0.5	20	0.475	30	0.4833	75	0.3667	16	0.4375	59	0.3475
rs500342	18	C	T	369	0.4743	91	0.4286	278	0.4424	212	0.4764	52	0.3654	160	0.4875	32	0.3594	3	0.5	29	0.3448	50	0.5	20	0.475	30	0.4833	75	0.3667	16	0.4375	59	0.3475
rs13381734	18	C	G	369	0.4729	91	0.4286	278	0.4406	212	0.4764	52	0.3654	160	0.4875	32	0.3438	3	0.5	29	0.3276	50	0.5	20	0.475	30	0.4833	75	0.3667	16	0.4375	59	0.3475
rs670749	18	A	G	369	0.4593	91	0.4451	278	0.4281	212	0.4788	52	0.375	160	0.4875	32	0.2188	3	0.3333	29	0.2069	50	0.5	20	0.475	30	0.4833	75	0.36	16	0.4062	59	0.3475
rs13381221	18	T	C	368	0.4592	91	0.4451	277	0.4278	212	0.4788	52	0.375	160	0.4875	31	0.2097	3	0.3333	28	0.1964	50	0.5	20	0.475	30	0.4833	75	0.36	16	0.4062	59	0.3475
rs492820	18	C	T	367	0.4755	91	0.4286	276	0.4438	212	0.4741	52	0.3654	160	0.4906	31	0.3387	3	0.5	28	0.3214	50	0.5	20	0.475	30	0.4833	74	0.3716	16	0.4375	58	0.3534
rs671624	18	T	C	366	0.4768	91	0.4286	275	0.4455	212	0.4741	52	0.3654	160	0.4906	31	0.3548	3	0.5	28	0.3393	50	0.5	20	0.475	30	0.4833	73	0.3699	16	0.4375	57	0.3509
rs80128778	18	T	C	368	0.4728	90	0.4278	278	0.4406	211	0.4763	51	0.3627	160	0.4875	32	0.3438	3	0.5	29	0.3276	50	0.5	20	0.475	30	0.4833	75	0.3667	16	0.4375	59	0.3475
rs485686	18	A	G	369	0.4661	91	0.4341	278	0.4335	212	0.4882	52	0.375	160	0.475	32	0.3438	3	0.5	29	0.3276	50	0.5	20	0.475	30	0.4833	75	0.3667	16	0.4375	59	0.3475
rs519783	18	G	A	370	0.4662	92	0.4348	278	0.4335	213	0.4883	53	0.3774	160	0.475	32	0.3438	3	0.5	29	0.3276	50	0.5	20	0.475	30	0.4833	75	0.3667	16	0.4375	59	0.3475
rs607946	18	A	G	370	0.4662	92	0.4348	278	0.4335	213	0.4883	53	0.3774	160	0.475	32	0.3438	3	0.5	29	0.3276	50	0.5	20	0.475	30	0.4833	75	0.3667	16	0.4375	59	0.3475
rs565913	18	T	C	369	0.4661	92	0.4348	277	0.4332	213	0.4883	53	0.3774	160	0.475	32	0.3438	3	0.5	29	0.3276	49	0.5	20	0.475	29	0.4828	75	0.3667	16	0.4375	59	0.3475
rs610092	18	T	A	369	0.4661	91	0.4341	278	0.4335	212	0.4882	52	0.375	160	0.475	32	0.3438	3	0.5	29	0.3276	50	0.5	20	0.475	30	0.4833	75	0.3667	16	0.4375	59	0.3475
rs559412	18	G	A	369	0.4526	91	0.4505	278	0.4209	212	0.4906	52	0.3846	160	0.475	32	0.2188	3	0.3333	29	0.2069	50	0.5	20	0.475	30	0.4833	75	0.36	16	0.4062	59	0.3475
rs563331	18	T	C	369	0.4715	91	0.4341	278	0.4406	212	0.4788	52	0.3654	160	0.4844	32	0.3594	3	0.5	29	0.3448	50	0.5	20	0.475	30	0.4833	75	0.36	16	0.4062	59	0.3475
rs62093530	18	A	G	356	0.4789	86	0.4244	270	0.4481	202	0.4678	47	0.3511	155	0.4968	32	0.3594	3	0.5	29	0.3448	48	0.5	20	0.475	28	0.4821	74	0.3716	16	0.4375	58	0.3534
rs645477	18	A	G	370	0.473	92	0.4348	278	0.4424	213	0.4765	53	0.3679	160	0.4875	32	0.3594	3	0.5	29	0.3448	50	0.5	20	0.475	30	0.4833	75	0.36	16	0.4062	59	0.3475
rs59570228	18	GGATA	G	370	0.3622	92	0.2391	278	0.4029	213	0.2723	53	0.1604	160	0.3094	32	0.25	3	0.5	29	0.2241	50	0.44	20	0.375	30	0.4833	75	0.4	16	0.2812	59	0.4322
rs12961764	18	T	C	368	0.3261	91	0.2198	277	0.361	211	0.2701	52	0.1635	159	0.305	32	0.4219	3	0.1667	29	0.4483	50	0.42	20	0.35	30	0.4667	75	0.38	16	0.25	59	0.4153
rs62212267	20	C	T	366	0.3169	91	0.2253	275	0.3473	210	0.331	52	0.2404	158	0.3608	32	0.2969	3	0	29	0.3276	50	0.25	20	0.2	30	0.2833	74	0.3311	16	0.25	58	0.3534
rs62212269	20	A	G	367	0.3147	91	0.2198	276	0.346	211	0.327	52	0.2308	159	0.3585	32	0.2969	3	0	29	0.3276	50	0.24	20	0.175	30	0.2833	74	0.3378	16	0.2812	58	0.3534
rs76267272	20	G	A	369	0.3171	92	0.3859	277	0.2942	213	0.3239	53	0.3962	160	0.3	32	0.3125	3	0.5	29	0.2931	50	0.32	20	0.4	30	0.2667	74	0.2973	16	0.3125	58	0.2931
rs1301447577	20	A	G	369	0.2927	91	0.4066	278	0.2554	212	0.2854	52	0.4135	160	0.2438	32	0.3438	3	0.3333	29	0.3448	50	0.35	20	0.45	30	0.2833	75	0.2533	16	0.3438	59	0.2288
rs1424309393	20	A	T	369	0.3062	91	0.4341	278	0.2644	212	0.3042	52	0.4423	160	0.2594	32	0.3281	3	0.3333	29	0.3276	50	0.36	20	0.45	30	0.3	75	0.2667	16	0.4062	59	0.2288
rs1258107032	20	T	A	369	0.3035	91	0.4341	278	0.2608	212	0.2995	52	0.4423	160	0.2531	32	0.3125	3	0.3333	29	0.3103	50	0.36	20	0.45	30	0.3	75	0.2733	16	0.4062	59	0.2373

rs1473358056	20	C	T	368	0.2486	91	0.1758	277	0.2726	212	0.2429	52	0.1635	160	0.2688	32	0.25	3	0.1667	29	0.2586	50	0.18	20	0.125	30	0.2167	74	0.3108	16	0.2812	58	0.319
rs1210597027	20	A	G	368	0.1997	91	0.1154	277	0.2274	212	0.2052	52	0.125	160	0.2312	32	0.1562	3	0	29	0.1724	50	0.13	20	0.05	30	0.1833	74	0.25	16	0.1875	58	0.2672
rs1285684358	20	A	C	369	0.1612	92	0.08696	277	0.1859	213	0.1714	53	0.09434	160	0.1969	32	0.09375	3	0	29	0.1034	50	0.08	20	0.025	30	0.1167	74	0.2162	16	0.1562	58	0.2328
rs1436718853	20	A	T	369	0.1911	92	0.1087	277	0.2184	213	0.1972	53	0.1132	160	0.225	32	0.1719	3	0	29	0.1897	50	0.12	20	0.075	30	0.15	74	0.2297	16	0.1562	58	0.25
rs79236426	20	G	A	369	0.1491	91	0.06593	278	0.1763	212	0.1651	52	0.08654	160	0.1906	32	0.1094	3	0	29	0.1207	50	0.08	20	0	30	0.1333	75	0.1667	16	0.09375	59	0.1864
rs1205686479	20	C	T	369	0.2249	91	0.3516	278	0.1835	212	0.2288	52	0.3654	160	0.1844	32	0.1875	3	0.5	29	0.1552	50	0.28	20	0.325	30	0.25	75	0.1933	16	0.3125	59	0.161
rs1262415727	20	C	T	369	0.2182	91	0.3407	278	0.1781	212	0.2241	52	0.3558	160	0.1812	32	0.1875	3	0.5	29	0.1552	50	0.27	20	0.325	30	0.2333	75	0.18	16	0.2812	59	0.1525
rs1423535535	20	T	G	366	0.123	91	0.06593	275	0.1418	211	0.128	52	0.06731	159	0.1478	32	0.09375	3	0	29	0.1034	49	0.1531	20	0.1	29	0.1897	74	0.1014	16	0.03125	58	0.1207
rs1424006606	20	C	T	367	0.188	92	0.07609	275	0.2255	210	0.2262	53	0.09434	157	0.2707	32	0.1875	3	0.1667	29	0.1897	50	0.11	20	0.075	30	0.1333	75	0.1333	16	0	59	0.1695
rs1479209115	20	G	A	366	0.1995	90	0.1	276	0.2319	209	0.2297	51	0.1078	158	0.269	32	0.2188	3	0.1667	29	0.2241	50	0.13	20	0.1	30	0.15	75	0.1533	16	0.0625	59	0.178
rs1418717426	20	A	T	358	0.2402	88	0.1477	270	0.2704	203	0.2562	49	0.1939	154	0.276	30	0.2833	3	0.1667	27	0.2963	50	0.16	20	0.05	30	0.2333	75	0.2333	16	0.125	59	0.2627
rs1159948585	20	A	G	363	0.1501	90	0.2167	273	0.1282	209	0.177	51	0.2549	158	0.1519	30	0.1667	3	0.3333	27	0.1481	49	0.1224	20	0.15	29	0.1034	75	0.08667	16	0.1562	59	0.0678
rs1467766855	20	C	G	368	0.3519	92	0.2554	276	0.3841	212	0.3349	53	0.2264	159	0.3711	32	0.375	3	0.1667	29	0.3966	49	0.3265	20	0.275	29	0.3621	75	0.4067	16	0.3438	59	0.4237
rs1166196201	20	G	C	366	0.2104	90	0.1222	276	0.2391	210	0.2024	51	0.1373	159	0.2233	31	0.2742	3	0	28	0.3036	50	0.19	20	0.075	30	0.2667	75	0.22	16	0.1562	59	0.2373
rs1429184444	20	T	C	368	0.2011	91	0.1209	277	0.2274	211	0.2085	52	0.1346	159	0.2327	32	0.2031	3	0	29	0.2241	50	0.18	20	0.15	30	0.2	75	0.1933	16	0.0625	59	0.2288
rs1396081550	20	C	T	370	0.3257	92	0.2011	278	0.3669	213	0.3357	53	0.2358	160	0.3688	32	0.3594	3	0.1667	29	0.3793	50	0.27	20	0.125	30	0.3667	75	0.32	16	0.1875	59	0.3559
rs1379390981	20	T	G	369	0.3794	91	0.2857	278	0.4101	212	0.3892	52	0.3173	160	0.4125	32	0.2969	3	0	29	0.3276	50	0.34	20	0.275	30	0.3833	75	0.4133	16	0.25	59	0.4576
rs1433249736	20	A	T	369	0.29	92	0.3804	277	0.2599	213	0.2887	53	0.3679	160	0.2625	32	0.2812	3	0.3333	29	0.2759	50	0.36	20	0.375	30	0.35	74	0.25	16	0.4375	58	0.1983
rs878937828	20	A	C	362	0.4171	90	0.3333	272	0.4449	207	0.4275	51	0.3627	156	0.4487	30	0.4667	3	0.3333	27	0.4815	50	0.37	20	0.3	30	0.4167	75	0.4	16	0.2812	59	0.4322
rs1191020582	20	A	G	361	0.4044	90	0.3389	271	0.4262	206	0.4102	51	0.3725	155	0.4226	30	0.4667	3	0.3333	27	0.4815	50	0.37	20	0.325	30	0.4	75	0.3867	16	0.25	59	0.4237
rs1266698617	20	G	A	361	0.4003	90	0.3278	271	0.4244	206	0.4102	51	0.3627	155	0.4258	30	0.4667	3	0.3333	27	0.4815	50	0.36	20	0.3	30	0.4	75	0.3733	16	0.25	59	0.4068
rs62198872	20	C	T	370	0.2892	92	0.2065	278	0.3165	213	0.2981	53	0.2075	160	0.3281	32	0.25	3	0	29	0.2759	50	0.27	20	0.225	30	0.3	75	0.2933	16	0.2188	59	0.3136
rs62196406	20	T	C	370	0.2216	92	0.3207	278	0.1888	213	0.2207	53	0.3208	160	0.1875	32	0.1875	3	0.3333	29	0.1724	50	0.28	20	0.325	30	0.25	75	0.2	16	0.3125	59	0.1695
rs1407099197	20	G	T	370	0.2486	92	0.3315	278	0.2212	213	0.2535	53	0.3302	160	0.2281	32	0.2344	3	0.3333	29	0.2241	50	0.28	20	0.325	30	0.25	75	0.22	16	0.3438	59	0.1864
rs1406603426	20	A	T	370	0.3068	92	0.3913	278	0.2788	213	0.3005	53	0.3774	160	0.275	32	0.2969	3	0.5	29	0.2759	50	0.37	20	0.425	30	0.3333	75	0.2867	16	0.375	59	0.2627
rs1980848880	20	C	T	367	0.1458	91	0.04396	276	0.1793	210	0.1548	52	0.06731	158	0.1835	32	0.1719	3	0.1667	29	0.1724	50	0.11	20	0	30	0.1833	75	0.1333	16	0	59	0.1695
rs1329708049	21	T	A	367	0.1839	90	0.08333	277	0.2166	211	0.1754	51	0.05882	160	0.2125	32	0.1875	3	0	29	0.2069	50	0.16	20	0.075	30	0.2167	74	0.223	16	0.1875	58	0.2328
rs1313570056	22	C	T	364	0.1126	91	0.03297	273	0.1392	211	0.09479	52	0.03846	159	0.1132	31	0.1774	3	0	28	0.1964	49	0.09184	20	0.025	29	0.1379	73	0.1507	16	0.03125	57	0.1842
rs9282911	22	A	G	362	0.3163	91	0.2363	271	0.3432	208	0.3125	52	0.2212	156	0.3429	30	0.3833	3	0.1667	27	0.4074	50	0.29	20	0.25	30	0.3167	74	0.3176	16	0.2812	58	0.3276
rs1373227070	22	T	C	370	0.327	92	0.2337	278	0.3579	213	0.3333	53	0.2547	160	0.3594	32	0.3438	3	0.3333	29	0.3448	50	0.23	20	0.125	30	0.3	75	0.3667	16	0.2812	59	0.3898
rs199636598	22	C	A	370	0.3162	92	0.3859	278	0.2932	213	0.3099	53	0.3868	160	0.2844	32	0.3438	3	0.3333	29	0.3448	50	0.34	20	0.375	30	0.3167	75	0.3067	16	0.4062	59	0.2797
rs796806674	22	T	C	368	0.2405	91	0.3516	277	0.204	212	0.2241	52	0.3462	160	0.1844	32	0.2344	3	0.5	29	0.2069	50	0.3	20	0.375	30	0.25	74	0.25	16	0.3125	58	0.2328
rs796527088	22	G	A	368	0.2432	91	0.3516	277	0.2076	212	0.2264	52	0.3462	160	0.1875	32	0.2344	3	0.5	29	0.2069	50	0.3	20	0.375	30	0.25	74	0.2568	16	0.3125	58	0.2414
rs35056946	22	G	T	368	0.1549	91	0.2418	277	0.1264	211	0.08768	52	0.1731	159	0.05975	32	0.2656	3	0.3333	29	0.2586	50	0.1	20	0.225	30	0.01667	75	0.3333	16	0.4688	59	0.2966
rs139065290	23	A	C	259	0.3707	64.5	0.4884	194.5	0.3316	146	0.3938	35.5	0.4507	110.5	0.3439	23	0.3696	2	0.25	21	0.381	36.5	0.3836	15.5	0.4194	21	0.3571	53.5	0.1869	11.5	0.3478	42	0.1429
rs4134455	23	A	G	247.5	0.2384	65.5	0.3664	182	0.1923	140	0.2607	34.5	0.4348	105.5	0.2038	19.5	0.4615	2.5	0.2	17	0.4118	38	0.1053	16.5	0.1515	21.5	0.06977	50	0.19	12	0.375	38	0.1316
