# Supplementary material for: Pharmacogenomics of coronary artery response to intravenous gamma globulin in kawasaki disease
Source: NPJ Genom Med. 2024 May 30;9:34. doi: 10.1038/s41525-024-00419-7 (PMC11139870; doi:10.1038/s41525-024-00419-7)
Supplement: Supplementary file 2 — Reporting Summary [file 41525_2024_419_MOESM2_ESM.pdf]

nature portfolio

Corresponding author(s): Sadeep Shrestha
Last updated by author(s): 2024/04/08

Reporting Summary

Nature Portfolio wishes to improve the reproducibility of the work that we publish. This form provides structure for consistency and transparency in reporting. For further information on Nature Portfolio policies, see our Editorial Policies and the Editorial Policy Checklist.

Statistics

For all statistical analyses, confirm that the following items are present in the figure legend, table legend, main text, or Methods section.

n/a	Confirmed	
☐	☒	The exact sample size (*n*) for each experimental group/condition, given as a discrete number and unit of measurement
☐	☒	A statement on whether measurements were taken from distinct samples or whether the same sample was measured repeatedly
☐	☒	The statistical test(s) used AND whether they are one- or two-sided<br>*Only common tests should be described solely by name; describe more complex techniques in the Methods section.*
☐	☒	A description of all covariates tested
☐	☒	A description of any assumptions or corrections, such as tests of normality and adjustment for multiple comparisons
☐	☒	A full description of the statistical parameters including central tendency (e.g. means) or other basic estimates (e.g. regression coefficient) AND variation (e.g. standard deviation) or associated estimates of uncertainty (e.g. confidence intervals)
☐	☒	For null hypothesis testing, the test statistic (e.g. *F*, *t*, *r*) with confidence intervals, effect sizes, degrees of freedom and *P* value noted<br>*Give P values as exact values whenever suitable.*
☒	☐	For Bayesian analysis, information on the choice of priors and Markov chain Monte Carlo settings
☒	☐	For hierarchical and complex designs, identification of the appropriate level for tests and full reporting of outcomes
☐	☒	Estimates of effect sizes (e.g. Cohen's *d*, Pearson's *r*), indicating how they were calculated

Our web collection on statistics for biologists contains articles on many of the points above.

Software and code

Policy information about availability of computer code

Data collection	Whole genome sequencing was performed on the MGISEQ-2000 instrument. The raw data was pre-processed according to the Broad Institute’s Genome Analysis Tool Kit (GATK) best practices workflow for small germline variants. Clinical data were collected from the hospital electronic health record (EHR).
Data analysis	PLINK 1.90 was used to perform an association analysis of individual SNPs based on an additive model. Functional annotation was conducted in Functional Mapping and Annotation (FUMA) v1.3.0. All genomic risk loci from FUMA were used to estimate gene risk score (GRS) by a simple risk alleles count method in R package.

For manuscripts utilizing custom algorithms or software that are central to the research but not yet described in published literature, software must be made available to editors and reviewers. We strongly encourage code deposition in a community repository (e.g. GitHub). See the Nature Portfolio guidelines for submitting code & software for further information.

Data

Policy information about availability of data

All manuscripts must include a data availability statement. This statement should provide the following information, where applicable:

- Accession codes, unique identifiers, or web links for publicly available datasets
- A description of any restrictions on data availability
- For clinical datasets or third party data, please ensure that the statement adheres to our policy

The data have been deposited with links to BioProject accession number PRJNA1055092 in the NCBI BioProject database (https://www.ncbi.nlm.nih.gov/bioproject/).

Research involving human participants, their data, or biological material

Policy information about studies with human participants or human data. See also policy information about sex, gender (identity/presentation), and sexual orientation and race, ethnicity and racism.

Reporting on sex and gender	The participants are children and only biological sex has been reported and included in the analyses.
Reporting on race, ethnicity, or other socially relevant groupings	Race and ethnicity were self-reported (by the parents) however, race based on ancestry derived from genomic data has been reported in the manuscript.
Population characteristics	Kawasaki Disease is a pediatric disease so only children have been included in the study.
Recruitment	Through our consortium of Kawasaki Disease in Northwest (based in Seattle's Children) and Childrens' of Alabama, we have developed and established a clinical cohort with a repository of biospecimen collected with parental consent. We have conducted and published multiple studies using the clinical data and biospecimen.
Ethics oversight	The parent cohort/study and this pharmacogenomic study conformed to the procedures for informed consent (parental permission) approved by institutional review boards at all sponsoring organizations and to human-experimentation guidelines set forth by the United States Department of Health and Human Services. The pharmacogenomic data management and analysis procedure was approved by the University of Alabama at Birmingham Institutional Review Board (IRB). The study was conducted in accordance with the local legislation and institutional requirements. Written informed consent for minor participation (children) in this study was provided by the participants' legal guardians/next of kin.

Note that full information on the approval of the study protocol must also be provided in the manuscript.

Field-specific reporting

Please select the one below that is the best fit for your research. If you are not sure, read the appropriate sections before making your selection.

☒ Life sciences ☐ Behavioural & social sciences ☐ Ecological, evolutionary & environmental sciences

For a reference copy of the document with all sections, see nature.com/documents/nr-reporting-summary-flat.pdf

Life sciences study design

All studies must disclose on these points even when the disclosure is negative.

Sample size	Kawasaki Disease is a rare disease. This pharmacogenomic study includes all patients with KD diagnosed using AHA guidelines, who underwent treatment using AHA guidelines and those who developed aneurysm using the valided Z-score. Pharmacogenomic studies, including our own have show OR ranging 2.5-3.0. While no formal power calculation was conducted, our OR ranging 2.5-3.0 show promising associations, consistent with previous reports with similar or smaller sample size. This is the largest WGS study in the field.
Data exclusions	Only patients met the clinical criteria (diagnosis, treatment and available z-score data) were included in the study. No other criteria were included to exclude participants except for the quality of the genomic data. All samples passed the criteria.
Replication	This is a rare disease with treatment outcome study so replication study is not easily available. However, we included 5 duplicate samples, which showed overall high SNP genotype concordance, with a kinship coefficient estimate, $\Phi > 0.497$ between duplicates. For the SNPs we report association, the concordant genotypes were confirmed between all duplicate samples.
Randomization	Randomization is not relevant to the study design to achieve the objective of the pharmacogenomic study.
Blinding	Yes - case-control status of patients (with and without aneurysm) was blinded during the sequencing and genomic data pre-processing phase. Only during the analysis, clinical data was merged.

Reporting for specific materials, systems and methods

We require information from authors about some types of materials, experimental systems and methods used in many studies. Here, indicate whether each material, system or method listed is relevant to your study. If you are not sure if a list item applies to your research, read the appropriate section before selecting a response.

Materials & experimental systems

n/a	Involved in the study
☒	☐ Antibodies
☒	☐ Eukaryotic cell lines
☒	☐ Palaeontology and archaeology
☒	☐ Animals and other organisms
☒	☐ Clinical data
☒	☐ Dual use research of concern
☒	☐ Plants

Methods

n/a	Involved in the study
☒	☐ ChIP-seq
☒	☐ Flow cytometry
☒	☐ MRI-based neuroimaging

Plants

Seed stocks	N/A
Novel plant genotypes	N/A
Authentication	N/A
